# Stable Isotope-Assisted Plant Metabolomics: Investigation of Phenylalanine-Related Metabolic Response in Wheat Upon Treatment With the *Fusarium* Virulence Factor Deoxynivalenol

**DOI:** 10.3389/fpls.2019.01137

**Published:** 2019-10-30

**Authors:** Maria Doppler, Bernhard Kluger, Christoph Bueschl, Barbara Steiner, Hermann Buerstmayr, Marc Lemmens, Rudolf Krska, Gerhard Adam, Rainer Schuhmacher

**Affiliations:** ^1^Department of Agrobiotechnology (IFA-Tulln), Institute of Bioanalytics and Agro-Metabolomics, University of Natural Resources and Life Sciences, Vienna (BOKU), Tulln, Austria; ^2^Department of Agrobiotechnology (IFA-Tulln), Institute for Biotechnology in Plant Production, University of Natural Resources and Life Sciences, Vienna (BOKU), Tulln, Austria; ^3^School of Biological Sciences, Institute for Global Food Security, Queen’s University Belfast, Belfast, United Kingdom; ^4^Department of Applied Genetics and Cell Biology (DAGZ), University of Natural Resources and Life Sciences, Vienna (BOKU), Tulln, Austria

**Keywords:** *Triticum aestivum*, *Fusarium graminearum*, LC-HRMS, *Fhb1*, resistance QTL

## Abstract

The major *Fusarium* mycotoxin deoxynivalenol (DON) is a virulence factor in wheat and has also been shown to induce defense responses in host plant tissue. In this study, global and tracer labeling with ^13^C were combined to annotate the overall metabolome of wheat spikes and to evaluate the response of phenylalanine-related pathways upon treatment with DON. At anthesis, spikes of resistant and susceptible cultivars as well as two related near isogenic wheat lines (NILs) differing in the presence/absence of the major resistance QTL *Fhb1* were treated with 1 mg DON or water (control), and samples were collected at 0, 12, 24, 48, and 96 h after treatment (hat). A total of 172 Phe-derived wheat constituents were detected with our untargeted approach employing ^13^C-labeled phenylalanine and subsequently annotated as flavonoids, lignans, coumarins, benzoic acid derivatives, hydroxycinnamic acid amides (HCAAs), as well as peptides. Ninety-six hours after the DON treatment, up to 30% of the metabolites biosynthesized from Phe showed significantly increased levels compared to the control samples. Major metabolic changes included the formation of precursors of compounds implicated in cell wall reinforcement and presumed antifungal compounds. In addition, also dipeptides, which presumably are products of proteolytic degradation of truncated proteins generated in the presence of the toxin, were significantly more abundant upon DON treatment. An in-depth comparison of the two NILs with correlation clustering of time course profiles revealed some 70 DON-responsive Phe derivatives. While several flavonoids had constitutively different abundance levels between the two NILs differing in resistance, other Phe-derived metabolites such as HCAAs and hydroxycinnamoyl quinates were affected differently in the two NILs after treatment with DON. Our results suggest a strong activation of the general phenylpropanoid pathway and that coumaroyl-CoA is mainly diverted towards HCAAs in the presence of *Fhb1*, whereas the metabolic route to monolignol(-conjugates), lignans, and lignin seems to be favored in the absence of the *Fhb1* resistance quantitative trait loci.

## Introduction

*Fusarium graminearum* (*Fg*) has been ranked among the 10 most important fungal plant pathogens (Dean et al., 2012). The fungus is a causal agent of the *Fusarium* head blight (FHB) disease of small grain cereals. It starts its spread in plants in their floral tissue and, after having established an infection in the plants, finally results in reduced grain quality and yield and most importantly crop contamination with mycotoxins. The major toxins produced by this fungus are—depending on the fungal chemotype—different type B-trichothecenes such as deoxynivalenol (DON), nivalenol and their acetylated derivatives 3-acetyl-DON or 15-acetyl-DON, as well as the estrogenic zearalenone. For the most prevalent of these toxins (DON and zearalenone), maximum legal limits have been established in the EU (EuropeanCommission, 2006) and many other countries (van Egmond et al., 2007).

Application of synthetic fungicides is not well suited to treat FHB efficiently. Fungicide application is limited to the short time of flowering, does not provide complete protection against the fungus or mycotoxin accumulation, and increases the risk of the fungus to become resistant to the fungicides (McMullen et al., 2012; Wegulo et al., 2015; Dweba et al., 2017). Among the different alternative strategies to counteract the disease, such as adaption of soil treatment methods, irrigation and fertilizer application, or crop rotation, resistance breeding offers the most effective and sustainable approach to control FHB and thereby mycotoxin contamination (Steiner et al., 2017). According to Bai et al. (2018), the three general types of resistance against FHB are resistance to initial infection (type I), resistance to fungal spread within a spike (type II), and resistance to mycotoxin accumulation (type III), the latter including both inhibition of mycotoxin production and detoxification of the mycotoxins (Mesterházy et al., 2005; Niwa et al., 2014). Resistance against FHB is quantitatively inherited and further influenced by interactions between genes and environmental factors. More than 100 quantitative trait loci (QTL) have been reported in the literature (Buerstmayr et al., 2009), which have been narrowed to about 50 QTL with unique chromosome locations (Liu et al., 2009; Bai et al., 2018; Ren et al., 2019). Among the few QTL that have been characterized in more detail, most efforts have been put on the resistance locus *Fusarium* head blight 1 (*Fhb1*) (Steiner et al., 2017; Bai et al., 2018). *Fhb1* originates from the Chinese cultivar Sumai 3 and has been shown to be the most effective and stable resistance QTL across different genetic backgrounds. Based on numerous previous studies on wheat, 20–60% of the variation for FHB resistance is accounted by this QTL (summarized in Zhuang et al. (2012)). *Fhb1* confers resistance against fungal spread (type II) and has also been demonstrated to be associated with a reduction in DON content in grain (type III resistance) (Lemmens et al., 2005; Lemmens et al., 2016). Despite considerable efforts to elucidate the genetic basis of *Fhb1*-mediated resistance, the causal gene(s) remain equivocal. Based on the finding that the ability of wheat to detoxify DON into DON-3-glucoside colocalizes with *Fhb1*, it had been speculated that the causal gene might encode a DON glucosyltransferase (Lemmens et al., 2005). However, recently Schweiger and colleagues fine mapped and sequenced a 1-Mb contig comprising *Fhb1* in the resistant line CM-82036 and found 28 genes at the *Fhb1* locus (Schweiger et al., 2016), of which none encoded for a glucosyltransferase with detoxification function. Among the 28 predicted genes, a putative pectin methylesterase inhibitor, which is downregulated in susceptible lines (Zhuang et al., 2012), a putative chimeric lectin with a pore forming toxin-like domain (Rawat et al., 2016) and a loss-of-function mutation of a tentative histidine-rich calcium-binding protein (*TaHRC*) have been suggested as candidate genes of *Fhb1*. Since *TaHRC* has been predicted to encode a functional protein in susceptible genotypes only, this gene has thus been proposed to act as a susceptibility factor of FHB (Su et al., 2018). However, to the present day, none of these genes has been convincingly demonstrated to be the true causal factor of *Fhb1* or the increased resistance against the mycotoxin DON, which can also not be explained by the pore-forming protein.

While the molecular mechanisms of toxicity and the biological role of most secondary metabolites of *F. graminearum* remains largely unknown (Adam et al., 2015), its major mycotoxin DON has been characterized in more detail. DON is a potent inhibitor of eukaryotic protein biosynthesis by binding to the peptidyltransferase of the ribosome (Chain et al., 2017). During the fungal infection of wheat, it is a virulence factor that is necessary for fungal spread into floral tissue (Proctor et al., 1995). *Fusarium* strains deficient in DON production show unhindered growth in the initially infected spikelet but are unable to penetrate the adjacent rachis to colonize further parts of the spike (Bai et al., 2002; Jansen et al., 2005). It is therefore currently believed that DON is not required for initial colonization but suppresses the formation of a wheat cell wall apposition barrier in the rachis by inhibiting or delaying translation of induced defense transcripts. In agreement with this, DON production has been described to be mainly induced in the rachis node and the rachis itself (Ilgen et al., 2009) and was described to be at its peak at the fungal infection front (Hallen-Adams et al., 2011). At low concentrations, however, the toxin has been shown to suppress programmed cell death, and thus, it is believed that it supports the fungus during its initial biotrophic lifestyle of the pathogen in the early phase of infection. Interestingly, when applied at low concentrations (up to ~1 mg/kg), DON shows defense-inducing, elicitor-like effects as has been demonstrated by successful priming of *Brachypodium distachyon* (Blümke et al., 2015) or the increase in production of pathogenesis-related proteins with known antifungal function like glucanase (PR2) and chitinase (PR3) as well as phenylalanine ammonia lyase (PAL). At concentrations of about 100–200 mg/kg, DON can induce the accumulation of reactive oxygen species, which probably trigger cell death thereby facilitating cell penetration and intracellular growth of the fungus (Desmond et al., 2008).

Resistance to DON can be regarded as a component of FHB resistance and has been shown to be associated with DON detoxification by conjugation (Desmond et al., 2008; Kluger et al., 2015; Lemmens et al., 2016) as well as induction of membrane transport proteins (Gunupuru et al., 2017). Over the last decade, many different omics studies have been published that pursued to elucidate the molecular mechanisms underlying *Fhb1*-mediated resistance in wheat. To this end, (near isogenic) wheat lines differing in resistance (e.g., by the presence/absence of *Fhb1*) have been compared with respect to defense-related changes on transcriptomic, proteomic, or metabolomic levels. The role of DON has been studied by treatment with pure toxin versus mock or comparative treatment with toxin producing wild-type strain and nonproducing strains (either via deletion or insertion).

As recently reviewed by Kazan and Gardiner (Kazan and Gardiner, 2018), the pure DON alone is capable of inducing a broadly based defense response in host plants: DON-induced transcripts were associated with processes such as DON detoxification (ATP-binding cassette transporters, UDP glucosyltransferases) (Hofstad et al., 2016), formation of PR proteins (Desmond et al., 2008), plant hormone biosynthesis, carbohydrate metabolism, and phenylpropanoid metabolism (Walter and Doohan, 2011; Chetouhi et al., 2016; Biselli et al., 2018).

Likewise, proteomics studies also suggest that plant defense against *Fg* infection is based on a diversified range of integrated mechanisms. For example, Zhang et al. (2013) report *Fhb1*-dependent increase in levels of wheat proteins that are known to be implicated in strengthening the plant cell wall, in the inhibition of fungal cell wall degrading enzymes, or in minimization of adverse effects on photosynthesis and energy metabolism. Eldakak et al. (2018) reported a broadly impaired defense response and deficiencies in energy metabolism in a *Fhb1*-deficient wheat line. Moreover, Liu et al. (2018) observed pathogen-related proteins to be linked with susceptible as well as with resistant wheat lines.

The effects and role of *Fusarium* and DON on host plant metabolism and the underlying defense and resistance mechanism were subject of several metabolomics studies, mostly wheat and barley, and have been reviewed by Gauthier et al. (2015). Wheat metabolites contributing to resistance were classified in seven groups: phenylpropanoids (more than 50% of the compounds were annotated as such), fatty acids, terpenoids, amino acids and derivatives, carbohydrates, amines and polyamines, and others (Gauthier et al., 2015). Moreover, phenolic acids and derivatives are also known to be involved in the plant defense (Nicholson and Hammerschmidt, 1992). Many polyphenols have been suggested to interfere with the fungus and have antifungal properties (Gunnaiah and Kushalappa, 2014; Atanasova-Penichon et al., 2016) to inhibit trichothecene biosynthesis (Boutigny et al., 2008; Boutigny et al., 2009; Gauthier et al., 2016) or to be used as metabolic building blocks for cell wall reinforcement, thereby hindering fungal spread inside infected tissue (Gunnaiah et al., 2012; Gunnaiah and Kushalappa, 2014). Besides specificity and extent of response, rapidity and spatial localization of plant defense appear to play a role in efficient defense reactions conferring resistance against FHB (Walter et al., 2008; Kluger et al., 2015; Warth et al., 2015b; Liu et al., 2018).

Resistance to the virulence factor DON is known to be a part of resistance against FHB and is also associated with *Fhb1*-mediated defense in wheat. Moreover, since DON is also known to show elicitor like properties including the induction of secondary metabolism in host plants (Gunnaiah et al., 2012), we wanted to investigate the effect of the pure mycotoxin on the metabolism of the central defense precursor phenylalanine (Phe) in wheat. To this end, a global and a tracer-based stable isotope-assisted workflow were combined to follow the *Fhb1*-related defense response of wheat spikes upon treatment with DON over a time period of 96 h. Methodical details on the metabolomics workflow as well as its benefits resulting from the combination of different labeling techniques can be found in the related publication (Doppler et al., 2019).

## Material and Methods

### Chemicals

The uniformly ^13^C-labeled tracer substance ^13^C_9_-phenylalanine (99% isotopic purity) and ^13^CO_2_ (99% purity) were purchased from Euriso-top (St-Aubin, Cedex, France). ELGA water was obtained from an ELGA Purelab Ultra-AN-MK2 system (Veolia Water; Vienna, Austria). Liquid chromatography (LC)-gradient methanol (MeOH; LiChrosolv) was purchased from Merck (Darmstadt, Germany). Formic acid (FA, MS grade) was obtained from Sigma-Aldrich (Steinheim, Germany and Vienna, Austria).

### Biological Experiment

**Figure 1:**
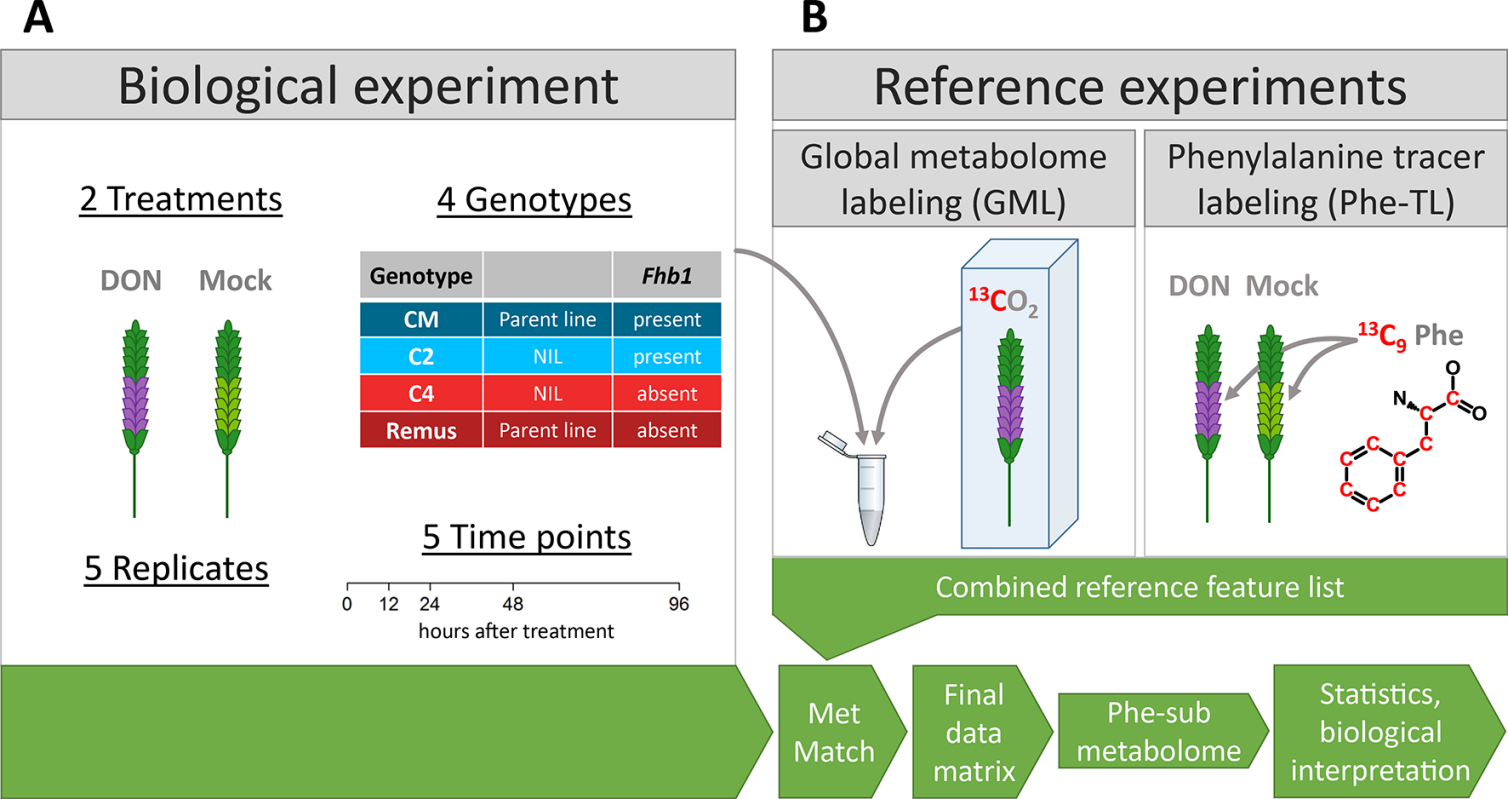
Schematic overview of the experimental setup of **(A)** the biological experiment and **(B)** the two reference experiments. The reference feature list generated from metabolites of the reference experiments was matched with data from the biological experiment into the final data matrix that was used for statistics and biological interpretation.

Four wheat genotypes were grown in a greenhouse until flowering stage. The genotype set consisted of two parent lines with different resistance levels against FHB (CM-82036—resistant, Remus—susceptible) and two lines derived from these two parent lines, both being near isogenic lines (NILs) of Remus but differing in the presence (C2) or absence (C4) of the *Fhb1* QTL (**Figure 1A**). The dataset used in this study is a subset of a large, comprehensive experiment and dataset consisting of two parent wheat cultivars and four NILs (C1, C2, C3, and C4), which allows the investigation of *Fhb1* as well as the *Qfhs.ifa-5A* QTL (not investigated in this manuscript). To keep the terminology coherent across all publications, which made/make use of these wheat lines, the two tested NILs are also termed C2 and C4 here. For more details about these wheat lines, the interested reader is referred to the publications of Kluger et al. (2015), who used this experiment to study the biotransformation of DON, and Warth et al. (2015b), who investigated the effect of DON treatment upon the primary metabolism with the same samples. The development of the NILs is described in Schweiger et al. (2013).

Flowering wheat spikes were treated with DON (DON in water; 1 mg per spike) or water (mock) as control samples. Samples were taken at 0, 12, 24, 48, and 96 h after treatment (hat). For each such condition, five biological replicates were sampled resulting in 200 samples in total. A detailed description of the growth conditions, treatment, and harvest of wheat plants is available in Warth et al. (2015b).

### LC-HRMS Analysis

Sample preparation and liquid chromatography–high-resolution mass spectrometry (LC-HRMS) measurement were carried out as described in Bueschl et al. (2014). Briefly summarized, 100 ± 2 mg of frozen, milled samples was extracted with 1 ml MeOH/H_2_O 3:1 (v/v) + 0.1% FA, vortexed and kept in an ultrasonic bath for 15 min. After centrifugation (14,000 rpm, 10 min), the supernatant was diluted with H_2_O + 0.1% FA to a final ratio of MeOH/H_2_O 1:1 (v/v) + 0.1% FA. Measurements were carried out using reverse-phase ultrahigh-performance liquid chromatography (C18, XBridge) coupled to an ESI-LTQ Orbitrap XL high-resolution mass spectrometer in positive ionization mode. Samples of each genotype were analyzed in separate batches. Control samples (KPX) containing a mixture of all biological samples were prepared beforehand and extracted in parallel and measured within every sample batch of one genotype to be able to account for different matrix effects and for systematic errors due to the long measurement batches. Tandem mass spectrometry (MS/MS) fragment spectra were recorded with a QExactive Orbitrap HF instrument using a data-dependent acquisition method with stepped collision energies (25, 35, and 45 eV), a resolution of 30,000 FWHM (at *m/z* 200), and the same chromatographic settings as for the full-scan measurements. Extraction artifacts due to the use of methanol were searched for but not detected in the experiment (Sauerschnig et al., 2018).

### Reference Experiments (Global Metabolome and Tracer Labeling)

Two individual labeling experiments were carried out to generate a reference list of all detectable metabolites of the phenylalanine-submetabolome. The method of combining global with tracer-based stable isotope labeling is described in detail in the associated manuscript of this combined publication (Doppler et al., 2019).

Global metabolome labeling (GML): For generating the reference metabolite/feature list, extracts of DON-treated samples (96 hat) originating from the biological experiment (Figure 1B) were internally standardized with a 1:1 mixture of extracts of ^13^C-labeled CM and Remus plants (Phytolabelbox). For a detailed description of the cultivation, please refer to the related publication (Doppler et al., 2019). Sample preparation was carried out as described for the biological samples. Samples were mixed 1:1:1 [^12^C (four genotypes DON treatment)/^13^C Remus DON treatment/^13^C CM DON treatment]. Subsequently, the internally standardized reference samples were analyzed with LC-HRMS with the same instrument and method parameters as the samples from the biological experiment. Data analysis was carried out with the AllExtract module of MetExtract II (Bueschl et al., 2017) using the settings listed in the **Supplementary Table 1**.

Tracer labeling (TL): For detecting the phenylalanine-derived submetabolome of wheat, a tracer labeling experiment was carried out (**Figure 1B**). Here, ^13^C_9_-phenylalanine (^13^C enrichment ~99%) was spiked to the flowering wheat spikes together with or without DON (1 mg ^13^C_9_-Phe and 0.2 mg DON per spike in water; wheat cultivar Remus) and harvested 72 hat. Samples were milled, extracted, and measured as described earlier. Data evaluation was carried out using the TracExtract module of MetExtract II (Bueschl et al., 2017) using the settings listed in **Supplementary Table 1**.

Generation of reference list: The resulting Phe-TL feature list containing the metabolites of the Phe-submetabolome was combined with the GML feature list to generate a reference feature list that includes all information from the two labeling experiments. Each metabolite in this reference list is a true metabolite of wheat, and those also detected in the Phe-TL reference experiments are constituents of wheat’s phenylalanine-derived submetabolome. For a detailed description of these steps, please refer to publication (Doppler et al., 2019).

### Matching of the Reference Feature List With the Biological Experiment

The software tool MetMatch (Koch et al., 2016) was then used to match all detected wheat metabolites from the two reference feature list to the samples of the biological experiment. This step was necessary, since this large biological experiment was carried out earlier than the reference experiments, and at this time, the information from the reference experiments and the assignment of the phenylalanine-submetabolome was not available. Parameters used for matching the reference list to the biological experiment are listed in the **Supplementary Table 2**.

### Data Preprocessing

Representative feature selection: The first step of the preprocessing was to select a single feature (i.e., metabolite ion) for each feature group (i.e., metabolite) that consisted of more than one metabolite ion (e.g. [M + H]^+^, [M + Na]^+^, or in-source fragments). The average most abundant feature in such a feature group was used to represent the respective metabolite in the statistical analysis, while the remaining features were not used for it. Moreover, a feature had to be present in at least three of five replicates of a single experimental variant to be considered for further statistical evaluation.

Missing value imputation: If a metabolite was not detected in a particular sample (i.e., missing values, no abundance values could be determined by the peak picking algorithm; e.g., not present or not distinguishable from instrument noise), it was imputed with half of the lowest abundance value present for the respective feature in any sample of the biological experiment (level-of-detection imputation). A random variation of ±15% was added to each such imputed value.

Abundance normalization: To account for shifts in ionization efficiencies or detector sensitivity between the different measurement batches, metabolite abundances in the biological samples were normalized prior to statistical analysis. This normalization was carried out via the KPX sample, which consists of a representative pool of the experimental wheat samples that was periodically measured within each batch.

For each measurement batch, the mean abundance values obtained for the KPX sample aliquots were calculated for each of the 172 phenylalanine-derived metabolites. Each biological sample was then standardized by dividing the individual metabolite ion abundances by the respective KPX mean value of the respective measurement batch to account for batch-specific intensities. Depending whether or not the metabolite was successfully detected in the KPX sample, either the very same metabolite ion was used (direct normalization), or if that particular metabolites was not present in at least 50% of the KPX sample aliquots, the metabolite closest to the target (± 1 min retention time deviation) was used for this measurement batch normalization (indirect normalization). In the rare case that a detected metabolite ion could not be normalized with either of these two methods, no abundance normalization was carried out.

### Annotation and Identification

All Phe-derived metabolites were investigated with the aim of identification (level 1) or annotation (levels 2–3) (Sumner et al., 2007).

Metabolite identification: For putatively identified metabolites in the biological samples, authentic reference standards were used for confirmation (comparison of the retention time, chromatographic peak shape, and the *m/z* values of the observed adducts).

Metabolite annotation: Metabolites, for which no authentic reference standards were available, putative sum formulas were generated from the accurate mass and queried against comprehensive wheat-specific metabolite databases. For metabolite ions with unknown ion species (e.g., only one metabolic feature was present in the LC-HRMS data), the most commonly observed adducts were used to calculate the metabolites’ mass from their ions. These adducts were [M + H]^+^, [M + Na]^+^, [M + K]^+^, [M + NH_4_]^+^, and [M + CH_3_OH + H]^+^.

Putative sum formulas were generated with the help of the Seven Golden Rules (Kind and Fiehn, 2007). However, since the total number of carbon atoms was known from the global labeling experiment, we omitted the check of the isotopic pattern (rule 1) and directly used the determined number of carbon atoms for each metabolite. The sum formula annotation was carried out with the elements C, H, N, O, S, and P and a mass deviation window of ±5 ppm.

In addition, the detected metabolites were queried against a wheat-specific, in-house-compiled metabolite database [e.g., phenylpropanoid-amides, metabolites described by Gauthier et al. (2015), metabolites described by Gunnaiah et al. (2012), the PlantCyc database, metabolomics.jp (http://metabolomics.jp)]. For this search only, the exact (i.e., theoretical) mass of the noncharged metabolites in the databases was compared to the experimentally determined masses of the unknown compounds. A mass deviation window of ±5 ppm was allowed. In addition, the number of carbon atoms was checked to be identical for the database hits and the detected metabolites to reduce the number of database hits. In addition, the core structure of the incorporated Phe-part(s) was used for efficient filtering of database hits. For details, see related publication (Doppler et al., 2019).

To cluster structurally similar phenylalanine-derived metabolites of wheat, we carried out a molecular networking approach (Watrous et al., 2012). Calculations were performed with a self-developed script (Python 2.7). A minimum cosine score of 0.75 for at least five MS/MS-fragment peaks were required for two compounds to be linked in the chemical similarity graph. A maximum mass deviation of 50 ppm was allowed for two fragment peaks to be considered to represent the same fragment.

### Statistics

All statistical analyses were carried out in the R programming language (R Core Team, 2014; version 3.1.0). The script comprised of the following steps and used the data matrix generated after data preprocessing:

Venn diagrams: To select metabolites present in a certain biological condition, Venn diagrams were created for a maximum of four biological conditions. A metabolite was classified as being present in such a group if it was present in at least three out of five replicates (at least 50% or the replicates) of a group. For this analysis, the data matrix without the replaced missing values was used.

Classification of treatment-related induced (TR+) or reduced (TR−) metabolites: Metabolite levels may be more or less abundant after DON treatment relative to the respective mock control. To find and annotate such metabolites in the different experimental conditions, we used the two classifications “treatment-related induced” (TR+) and “treatment-related reduced” (TR). The classification TR+ indicates that a metabolite is more abundant upon DON treatment than in the corresponding control (same genotype and time point after mock treatment), while the classification TR− indicates that a metabolite is significantly less abundant as a consequence of the DON treatment, respectively. For a metabolite to fall into one of the two categories, the following criteria (a)–(c) had to be fulfilled:

- Same abundance level immediately after DON application (0 hat): For the tested genotype, the metabolite’s abundance must not be significantly different (*t* test; critical alpha: 0.05) between the DON- and the mock-treated samples at the time point 0 hat.
- Significantly different abundance at the tested time point: For this, metabolite abundances were compared for every genotype and time point separately. Abundance levels between corresponding DON- and mock-treated samples must be significantly different (*t* test; critical alpha: 0.05).
- For the metabolite under investigation, the corresponding pair of mock- and DON-treated samples must at least differ by a factor of 2: For being assigned to the category TR+, the ratio of the mean abundances (arithmetic mean, *n* = 5 replicates) obtained for the DON-treated samples relative to the controls must be >2 or <0.5 for the category TR−.

For example, the metabolite X is annotated as TR+ in C2 at 48 hat: if its abundance values are not significantly different between the DON and mock treatment 0 hat but significantly different at the time point 48 hat and if, additionally, the mean fold change between these DON-treated and the control samples is more than 2.

Classification of QTL-associated metabolites: Metabolite levels may be different between the two genotypes C2 and C4 as a consequence of the presence or absence of the *Fhb1* QTL. This includes both constitutively differing metabolites as well as TR+ or TR− classified compounds, which are induced (TR+) or reduced (TR−) to a significantly different extent. We have classified such a difference as “QTL effect” (abbreviation “QTL” in Supporting Information 1). For the “QTL effect” classification, the following statistical criteria (a) and (b) must be fulfilled:

- The metabolite abundance levels must be significantly different (*t* test; critical alpha: 0.05) between the C2 and C4 genotype for the tested treatment at the tested time point.
- The ratio of the mean abundances (arithmetic mean, *n* = 5 replicates) for C2 relative to C4 must be >2 or <0.5 for the tested treatment and time point.

Multivariate comparison: The unsupervised statistical method principal component analysis (PCA) was used to investigate the dataset. For this, the missing-value imputed data matrix was autoscaled and mean centered to obtain comparable metabolite levels.

Time course clustering: To cluster metabolites with similar time course profiles, a correlation-based approach was used. All samples of the C2 and C4 genotypes were compared simultaneously with the unsupervised hierarchical cluster analysis employing squared Euclidean distance and Ward linkage. However, instead of pairwise correlating the abundance levels of individual metabolites across different samples, the clustering was carried out by comparing the abundance of the samples across different metabolites. The thereby generated dendrogram consisted of metabolites instead of samples at its leaves. Metabolites closely related in the dendrogram show similar time course profiles, while metabolites that are further apart show less similar or even different time courses. Subsequently, the dendrogram was manually cut into subtrees, each representing a set of metabolites with similar time courses for all metabolites contained within it.

## Results and Discussion

### Overview of the Phe-Submetabolome and the Biological Experiment

After initial data evaluation of the reference datasets with our stable isotope labeling assisted workflows (Kluger et al., 2014), the GML and the Phe-TL derived feature lists were combined as shown in the associated manuscript of this tandem publication (Doppler et al., 2019) This resulted in a reference list that was matched against the LC-HRMS data of the biological experiment with MetMatch (Koch et al., 2016). The final data matrix contained 1,019 plant metabolites.

**Figure 2:**
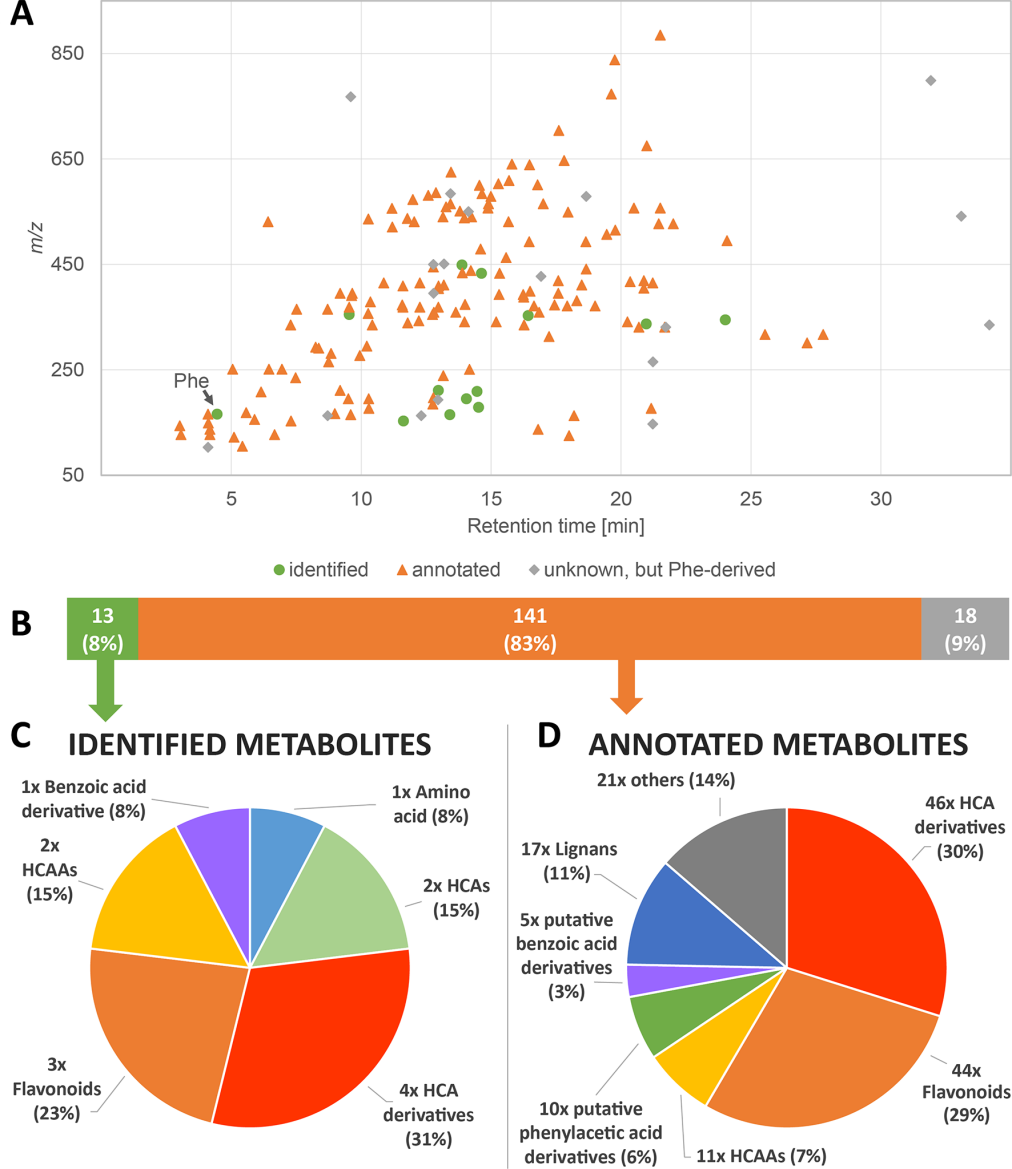
Overview of the detected phenylalanine-submetabolome. **(A)** Each dot represents one of the 172 phenylalanine-derived wheat metabolites detected in the presented study. **(B**–**D)** Overview of identified and annotated compounds and classification of identified **(C)** and annotated **(D)** compounds according to their chemical structures.

To deduce the final set of Phe-derived metabolites from the MetMatch obtained final data matrix, all TL-derived metabolites as well as identified and annotated metabolites that can be linked to the Phe-derived metabolic pathways were collected. This resulted in total of 172 Phe-derived metabolites, which constitute the detectable Phe-submetabolome and correspond to 17% of all detected wheat metabolites in this biological study. **Supplementary Table 3** lists all metabolites of the phenylalanine submetabolome. *m/z* values ranged from *m/z* 103 to *m/z* 885 (**Figure 2A**). The real number of Phe-derived metabolites might even be higher since low abundant compounds may not have been detected or their ^13^C-labeled analogues may not have been produced in large quantities during the rather short time period after application of the ^13^C-labeled tracer. The applied labeling approach, however, still allowed detecting many Phe-derived metabolites in an untargeted manner.

Biochemically, Phe is formed from phosphoenolpyruvate and erythrose-4-phosphate via the shikimate pathway in the chloroplast and is a central intermediate metabolite in plant metabolism. Phe is a precursor of various metabolites with the major biotransformation routes into (i) polymers like lignin and proteins; (ii) aromatic compounds such as simple phenols (C_6_), benzoic acids (C_7_, after ß-oxidation or nonoxidative pathway), phenylacetic acid derivatives (C_8_, after decarboxylation), hydroxycinnamic acids (HCAs, C_9_), and various conjugates of the different classes such as, e.g., HCAAs and other phenylpropanoids like flavonoids, monolignols, lignans, and coumarins; or (iii) degradation via yet unknown pathways in higher plants as reviewed in Hildebrandt et al. (2015).

A comparison with authentic reference standards allowed the identification of the tracer Phe itself as well as 13 other metabolites of the Phe-submetabolome (**Figures 2B, C**). Sum formulas could be generated for 170 of the 172 Phe-metabolites. Unique sum formulas were generated for 82 (48%) such compounds with the help of the total number of carbon atoms derived from the data of the GML reference experiment. Regarding the number of carbon atoms originating from the Phe structure motif, we found compounds that contained 6, 7, 8, 9, 16, and 18 carbons, as can be expected from metabolites of the above-mentioned pathways. Although salicylic acid is well known to be involved in plant defense and potentially can be produced from Phe (Hao et al., 2018), we were neither able to find salicylic acid with the isotope-assisted approach nor by targeted search using a reference standard. For database search, a special focus was put on Phe-derived metabolites that have already been described to play a role in the defense against FHB and DON (Gunnaiah et al., 2012; Gauthier et al., 2015). By comparison of the measured Phe-submetabolome with metabolites reported to be related to *Fusarium* and/or mycotoxin response, 141 compounds could be annotated (**Figures 2B, D**). The Phe-derived submetabolome consists of metabolites of various substance classes (**Figure 2C, D**). Our approach enabled us to identify (level 1) 8% and annotate (levels 2 and 3) 83% of the Phe-submetabolome according to (Sumner et al., 2007).

As discussed in detail in the associated methodical paper (Doppler et al., 2019), most annotated metabolites did not have a unique database hit rendering their annotations ambiguous. The high number of putative database hits without definite proof of their true identity remains a major challenge of any untargeted metabolomics experiment. We have evaluated metabolites of interest with great care and reviewed database hits manually. Incorrect database annotations were removed by combining global and tracer labeling-derived molecular formula and Phe-(sub)structure units. Annotations were assigned to substance classes, and with the help of molecular networking, categorization in an untargeted fashion became possible.

To reduce the technical variance in the dataset and thus allow the comparison between wheat genotypes that were measured in different batches, metabolite abundance normalization within the different LC-HRMS measurement batches was carried out. With this approach, 85 of the 172 metabolites were normalized directly using the average abundance of the very same feature in the pooled wheat QC sample (KPX), and another 72 metabolite abundances were normalized using a close-by eluting Phe-derived metabolite, which was consistently present across all samples (indirect normalization). The remaining 15 metabolites could not be successfully normalized. The success of data normalization has been checked and is demonstrated by a PCA scores plot of the KPX samples (**Supplementary Information 2**).

### Overview of Treatment Related Induced/Reduced Metabolites and Evaluation of the Fhb1-QTL Effect

**Figure 3:**
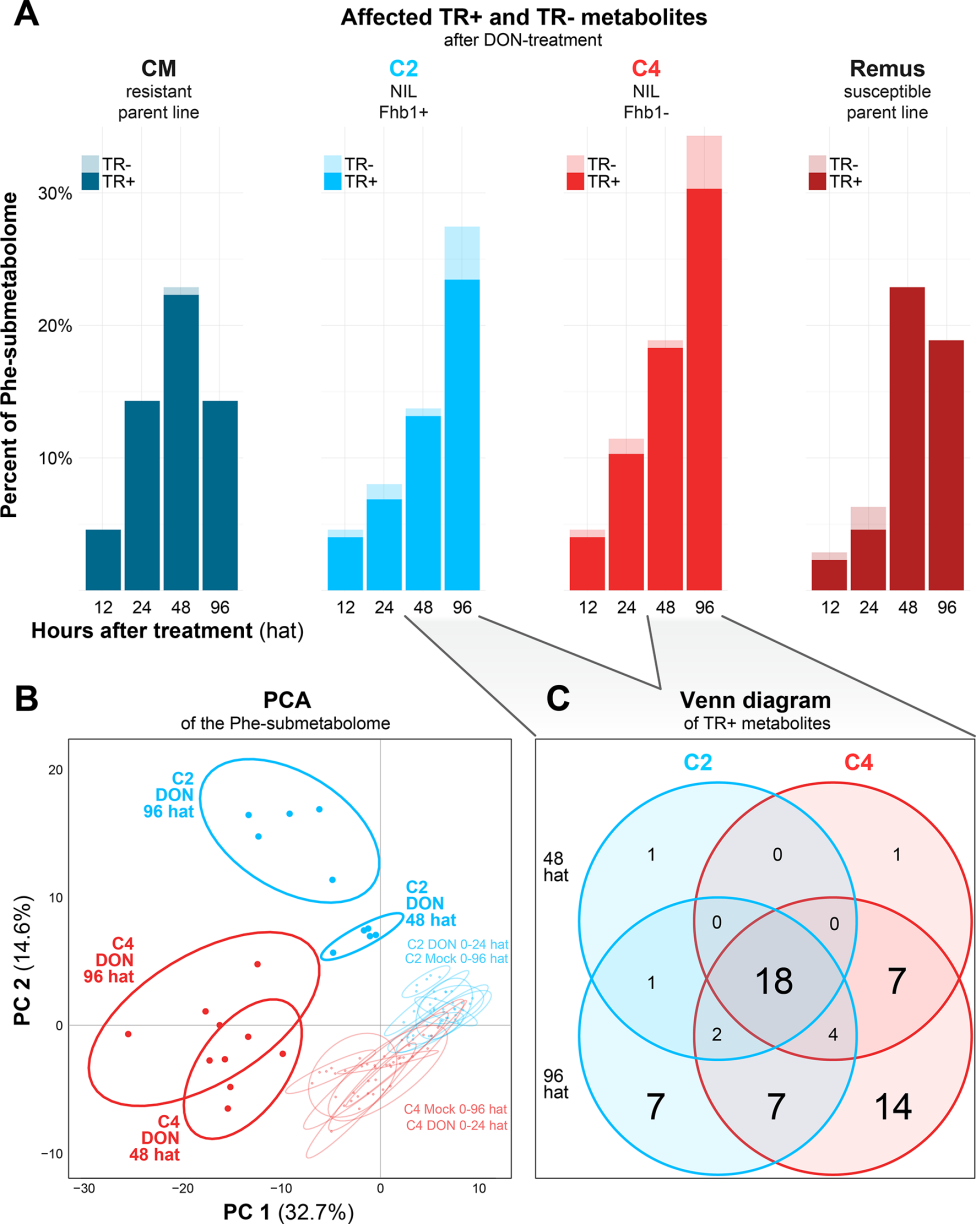
**(A)** Histogram of Phe-derived metabolites with significantly differing abundance levels in the DON-treated groups over time for each of the four tested wheat lines. **(B)** Scores plot of the PCA for the C2 and C4 genotypes based on the metabolite profiles of the DON and mock treatments. **(C)** Venn diagram of metabolites classified as TR+ in C2 and C4 at 48 and 96 h after DON treatment.

To assess whether the tested wheat lines differ in terms of their metabolic response to the DON treatment (TR+/−), the number of metabolites with significantly affected abundance levels after DON treatment were counted for each time point and genotype (**Figure 3A**). We found that at a single time point and genotype, the abundance of up to ~30% of the Phe-derived metabolites were either significantly increased (TR+) or decreased (TR−) compared to mock treatment, including those metabolites that were only detected in DON-treated samples but not in the controls.

While the number of DON-affected metabolites increased in each of the wheat lines over time, the resistant cultivar CM showed a faster increase in the number of TR+ Phe derivatives compared to Remus and the two NILs. The faster response of the resistant cultivar CM is also reflected in a decrease in TR+ metabolites 96 after the DON treatment again. Metabolites with this behavior are marked in the “Maximum CM 48 hat” column in **Supplementary Table 3**.

A similar trend that the number of TR+/− metabolites decreased 96 after DON treatment was observed for the parent genotype Remus. It should be noted, however, that for Remus, the graph does not reflect the observed steady increase in abundance of individual metabolites over time. Instead, only significantly differing metabolites are considered, the number of which decreased due to a somewhat higher variability of metabolite abundances found for the Remus samples 96 hat.

The resistant parent cultivar CM was the only one with distinctly different behavior compared to the other tested wheat lines. Among the significantly differing metabolites, 34 showed constantly lower and 49 constantly higher abundances over all tested time points (columns “CM low” and “CM high” in the **Supplementary Table 3**). A similar, distinct difference between CM and the other tested wheat lines had also been observed for the primary metabolites investigated by Warth et al. (2015b). Another fact, which should be taken into account, is that CM is able to detoxify DON much faster to its major derivative DON-3-glucoside than the remaining genotypes of the presented study (Kluger et al., 2015). It is therefore known that resistance in CM is not only based on the production of certain unique defense metabolites but also mediated by the speed of this defense reaction (Walter and Doohan, 2011; Schweiger et al., 2016).

As the genetic constitution of the two parent lines Remus (susceptible) and CM (resistant) does not only differ in the alleles of the studied *Fhb1* QTL but many other genes, the observed effects on the metabolite profiles cannot directly be related to resistance or the presence of *Fhb1*, respectively. Thus, to evaluate the effect of the *Fhb1* QTL, the NILs C2 and the C4, both being near isogenic of Remus (genomes ca. 98% identical), were investigated in detail. In total, 159 Phe-derived metabolites were detected in C2 and C4 samples with 99 of those being detected in all experimental variants. Nine metabolites were detected solely in the C2 genotype and 24 in C4. Twenty-three metabolites were only formed upon DON treatment, including five uniquely for C2 and nine for C4, respectively (**Supplementary Table 3**). Multivariate comparison of the metabolite profiles by PCA revealed a clear separation of the C2 and C4 NILs 48 and 96 after DON treatment from all other samples and a separation between the respective C2 and C4 samples (**Figure 3B**). Moreover, the general separation between C2 and C4 NILs suggests a constitutive difference between their metabolite profiles. On the contrary, mock-imposed changes in the profile of Phe-derived metabolites seemed to be negligible as mock-treated samples did not separate over time.

Based on that, we checked the C2 and C4 genotypes after DON treatment for the time points 48 and 96 hat and depicted the overlap of TR+ metabolites as a Venn diagram in **Figure 3C**. The number of TR+ metabolites increased from 48 to 96 hat in both genotypes. Interestingly, the C2 genotype, which carries the *Fhb1* resistance QTL, contained less significantly DON-induced metabolites (40) than the susceptible C4 genotype (53). Among these four comparisons, 18 metabolites were consistently classified as TR+. Ninety-six after the DON treatment and another 7 and 14 Phe-derived metabolites were specifically induced in C2 and C4, respectively. The seven C2-specific metabolites may therefore be associated with *Fhb1*-mediated resistance and include compounds that were exclusively detected in the DON-treated and absent in the mock-treated samples. A more detailed discussion of those metabolites is presented below.

### Time Course Kinetics of the Phe-Submetabolome

In this study, 1 mg toxin was added per flowering wheat spike by treating 10 adjacent spikelets (2 florets per spikelet) with 10 µl aliquots of a 5g/l DON solution. This corresponds to a concentration of roughly 1 mg/g fresh weight, which can be assumed to be equally distributed in the main 20 florets of the treated spike. This toxin concentration is approximately in the range of what has been reported for infected spikelets and connected rachis node tissue 4 days after *Fg* treatment (Savard et al., 2000) and may therefore also be expected to prevail at the hyphal tip *in situ* (cellular/molecular level) under infection conditions. At this concentration, DON is acutely toxic and likely to inhibit protein biosynthesis and defense more or less completely. DON, added according the experimental setup applied here, first has to penetrate the plant tissue and diffuse into the cells. Thus, active defense processes will most probably originate from cells at the front of the diffusing toxin, where its concentration is much lower than 1 mg/g.

**Figure 4:**
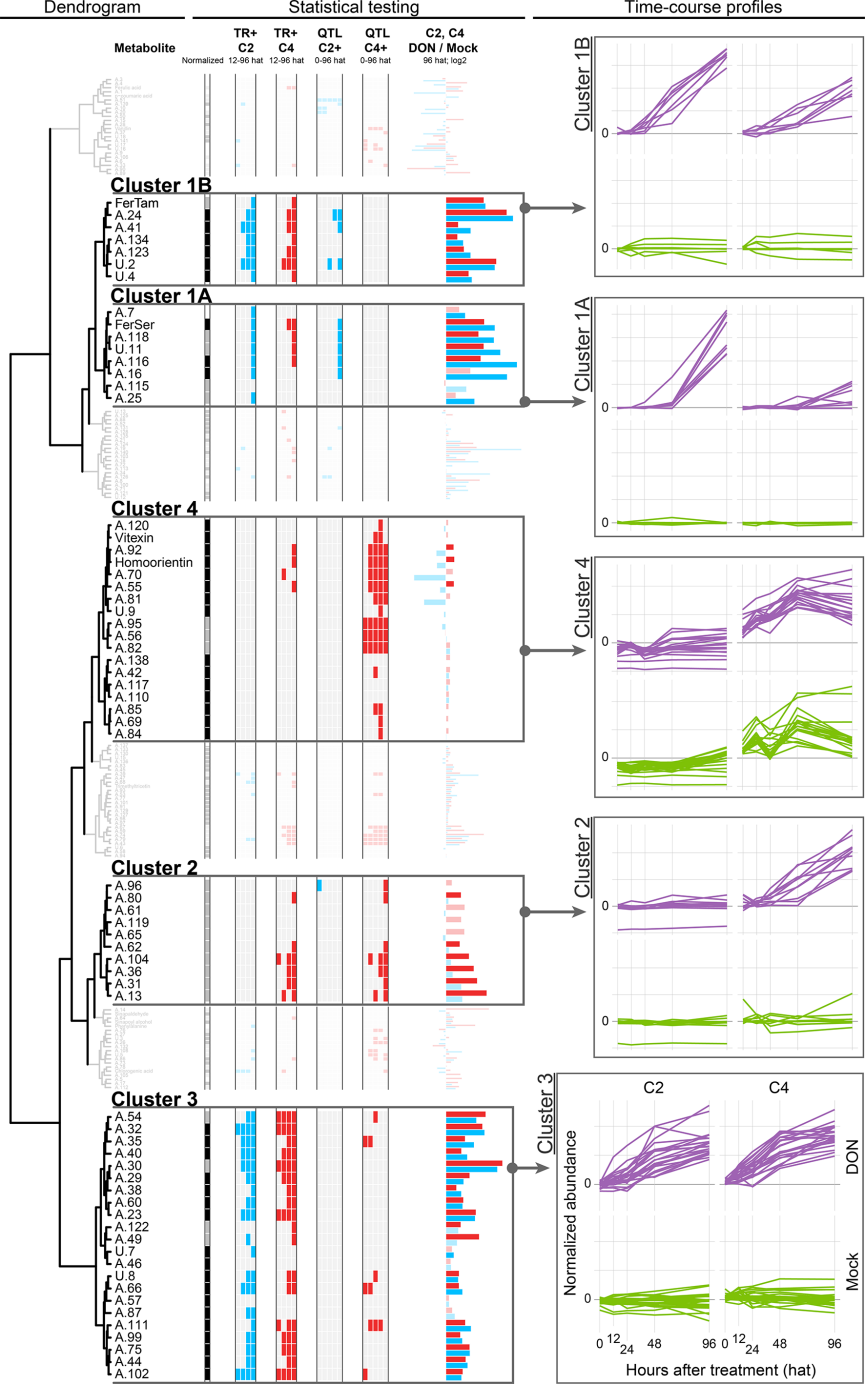
Dendrogram of the time course profiles of the Phe-submetabolome. The results obtained from univariate statistics (TR+ and QTL effect) are depicted as color-coded columns next to each metabolite of the dendrogram. The time courses of all metabolites within a chosen cluster are depicted as overlaid graphs. Each such line in the time course represents the average abundance values obtained for the five replicates per time point and treatment (normalized to the mean value of the 0 hat mock-treated samples). Color code for normalization: black, direct normalization; gray, indirect normalization; and white, no normalization. Color code for statistical tests (TR+ and QTL effect): white, no significant difference/no effect; red/blue, significant difference/effect.

To illustrate metabolite abundances over time, time course profiles of the C2 and C4 genotypes were correlated and subsequently clustered based on their similarity. The result of clustering is illustrated as a dendrogram, in which metabolites with similar time courses were grouped (i.e., small branch in the dendrogram with low height), while metabolites with dissimilar profiles were separated and are further apart in the dendrogram. A fixed cutoff value or number of clusters of similar Phe-derived metabolites proved to be difficult; therefore, the dendrogram was inspected manually for subclusters with similar time courses (**Figure 4** and **Supplementary Table 4**).

We focused on those time courses that showed large differences between the DON- and mock-treated samples (i.e., TR+ metabolites) and selected four subclusters for further discussion. Statistically significant differences were assessed by univariate comparisons to find metabolites with DON-induced (TR+) as well as *Fhb1* QTL-related differences in metabolite abundance (resistant NIL C2 compared to susceptible C4) (for details, see Material and Methods section).

The following clusters have been selected for further discussion:

### Cluster 1

This cluster comprises of 15 metabolites and is likely the most intuitive cluster as one might think that wheat produces new metabolites to counteract the stress imposed by the mycotoxin.

A univariate comparison of the DON- and mock-treated samples confirms that the metabolites’ abundances are significantly higher after DON treatment (TR+ in C2 and C4, 48 and 96 hat). Moreover, the abundances of eight of these metabolites increased to levels which were significantly higher in presence of *Fhb1* (i.e., C2 compared to C4). Visual inspection also revealed that the respective metabolite levels increased faster in the *Fhb1*-harboring C2 genotype, suggesting that *Fhb1* mediates their production.

### Cluster 1A

Cluster 1A represents seven DON-induced metabolites, namely, six HCAAs, one unknown (U.11), and, in addition to these metabolites, the dipeptide (Phe-Asp or Asp-Phe, A.25)(see **Table 1**). For five of those, the abundances increased to a significantly greater extent in the resistant (C2) in comparison to the susceptible NIL (C4), suggesting their involvement in *Fhb1*-mediated defense. When inspecting the time courses manually, QTL effects were also observed for the remaining three metabolites of this cluster (A.7, A.115, and A.25), although they did not pass the strict statistical criteria. The dominating group of HCAAs is derived from conjugation of HCA(s) with biogenic amine(s), such as tryptamine, serotonine, putrescine, agmatine, and cadaverine, that are presumably involved in defense against biotic stress [reviewed in Macoy et al. (2015)]. Besides antifungal activity, cell wall reinforcement as a response to pathogen attack and wounding has been reported (Facchini et al., 2002). The diHCAAs feruloyl-coumaroylputrescine (A.116) and di-feruloylputrescine (A.118) have been reported for maize (Moreau et al., 2001), and the latter has also been isolated from an extract of a *Fusarium*-resistant cultivar (Miller et al., 1996). Atanasova-Penichon et al. (2012) have investigated both of these metabolites in maize after treatment with *Fg* and observed an increase over the observation period from treatment to maturity at 74 days after treatment in both *Fg*-treated and also in nontreated controls.

**Table 1:** Metabolites assigned to cluster 1 of the time course profile clustering.

ID	Putatively Annotated or Identified as	Number of Annotations	Substance Class	Fold-change DON/Mock 96 hat	
				C2	C4
Cluster 1A					
FerSer	Feruloylserotonin *		HCAA	105	38
A.7	(Coumaroylhydroxyputrescine; caffeoylputrescine) isomer 1	2	HCAA	6.2	3.5
A.16	(Coumaroylhydroxyputrescine; caffeoylputrescine) isomer 2	2	HCAA	335	10
A.25	Phenylalanylaspartic acid or aspartylphenylalanine	1	Others (dipeptide)	15	2.5
A.115	Di-coumaroylputrescine	1	HCAA	7	0.9
A.116	Feruloyl-coumaroylputrescine; caffeoyl-coumaroylcadaverine	2	HCAA	863	27
A.118	Di-feruloylputrescine; coumaroyl-sinapoylputrescine; feruloyl-coumaroylhydroxycadaverine; feruloyl-caffeoylcadaverine	4	HCAA	96	22
U.11				177	36
Cluster 1B					
FerTam	Feruloyltryptamine *		HCAA	43	37
A.24	Valylphenylalanine	1	Others (dipeptide)	592	323
A.41	p-Hydroxycinnamyl alcohol 4-D-glucoside isomer 2	1	HCA derivative	11	3.2
A.123	ChemSpider ID 204894; ChemSpider ID 4976380	2	Lignan, HCA derivative	10	5.5
A.134	ChemSpider ID 34497391	1	HCA derivative	5.2	3
U.2				105	121
U.4				12	8.7

*Identified based on comparison with an authentic reference standard

Our findings that HCAAs are toxin induced and significantly more abundant in *Fhb1*-harboring NIL C2 compared to the susceptible C4 are in good agreement with those of Gunnaiah and colleagues who investigated FHB resistance in *Fhb1*-related wheat NILs upon *Fg* treatment. They concluded that the *Fhb1* QTL-derived resistance is mainly associated with HCAA-mediated cell wall reinforcement by thickening of xylem tissue (Gunnaiah et al., 2012). In complementary studies, comparing the effect of trichothecene producing and nonproducing *Fg* isolates on a susceptible wheat genotype, higher levels of HCAAs, especially FerSer, were found in the rachis tissue of infected wheat spikes (Gunnaiah and Kushalappa, 2014). Our observations are in agreement with these findings, and our data demonstrate that this metabolic response can not only be triggered by *Fg* but also by the pure DON. In a very recent study, Brar et al. (2019) have used nondestructive X‐ray imaging and computed tomography and Fourier-transform infrared spectroscopy to investigate the location and type of biopolymers presumably produced/reinforced as a physical barrier against the spread of *Fg* within wheat spikes. While the structural reinforcement was clearly located at the rachilla and rachis nodes, the type of barrier facilitating type II resistance remains elusive. Based on Fourier-transform infrared measurements of the cell wall polymers, lignin (in contrast to cellulose, pectin, or xylan, which all remained unchanged) was found to be the only consistently altered biopolymer. When comparing a susceptible wheat cultivar with several derived NILs carrying resistance alleles (including an *Fhb1*-harboring line), lignification was also observed in the susceptible parent line, suggesting this mechanism to be a basal defense response rather than a specific type II resistance against FHB in wheat (Brar et al., 2019). This conclusion does also agree with our findings, as HCAAs may act as metabolic precursors in lignification and are formed by both NILs, with this response being more pronounced in the resistant line C2. The true metabolic destination of the induced HCAAs might be unraveled by cell wall hydrolyzation experiments in further experiments.

In addition to stress- and defense-related functions, HCAAs are also reported to play a role in plant growth and development including seed development, maturation, organ growth, or to act as signals for flower development [for a review, the interested reader is referred to Bassard et al. (2010)]. Interestingly, we have also detected further HCAAs among the Phe-derived metabolites, which did not fall into cluster 1. Dicoumaroylspermidine (A.78; annotation described in more detail in (Doppler et al., 2019) may serve as an example for such a development- rather than defense-related HCAA, which has been described to play a role in the development of male reproduction organs of tobacco (Facchini et al., 2002). In agreement with the proposed function in organ development, dicoumaroylspermidine did neither show treatment- nor genotype-related response over time.

One major mode of action of DON is the inhibition of protein biosynthesis [reviewed in Chain et al. (2017)], which results in incomplete protein formation and consequently recycling of protein fragments via small peptides. In good agreement with this, we detected compound A.25, which was annotated as the dipeptide Phe-Asp or Asp-Phe. In addition, two more dipeptides (A.24 Phe-Val/Val-Phe in cluster 1B and A.35 Glu-Phe/Phe-Glu in cluster 3) were annotated and found to be significantly more abundant in DON-treated samples. While Phe-Asp/Asp-Phe and Phe-Val/Val-Phe were formed in higher amounts in *Fhb1*-carrying NIL C2, Phe-Glu/Glu-Phe did not show such a QTL effect. Since DON has been demonstrated to be detoxified more rapidly in the resistant line C2 (Kluger et al. (2015), a more efficient protein recycling may be expected for these lines, which was indeed observed in form of higher levels for two of the three detected dipeptides.

### Cluster 1B

This cluster consists of seven wheat compounds, which constitute feruloyltryptamine (HCAA, identified), the dipeptide Phe-Val/Val-Phe (A.24), p-hydroxycinnamyl alcohol glucoside, one compound which has been annotated as either a dicaffeoylquinic acid isomer or succinyl-podophyllotoxin (lignan), and two unknown Phe-derived metabolites (**Table 1**).

All of these compounds are classified as TR+ for at least one time point (96 hat). Compared to cluster 1A, cluster 1B is characterized by a more rapid increase in metabolite abundances over time. Visual inspection of time course profiles additionally revealed that, for the resistant NIL C2, the abundance levels tend to increase faster than for the C4 genotype. Thus, for these DON- and *Fhb1*-responsive metabolites, velocity appears to be decisive, which is additionally supported by a clear increase in abundance levels in the resistant parent line CM (**Supplementary Information 1**). The influence of the defense reaction’s speed in defending against *Fg* and, therefore, higher resistance levels has been shown in several studies before (Walter et al., 2008; Kluger et al., 2015; Warth et al., 2015b; Liu et al., 2018). Hydroxycinnamyl alcohol glucoside (A.41) constitutes a lignin-precursor and putative storage form of the short-lived hydroxycinnamyl alcohol and has also been reported by Gunnaiah and Kushalappa (2014) to be significantly elevated upon treatment with a trichothecene producing *Fg* isolate. In contrast, nontrichothecene-producing *Fg* did not cause an increase in the monolignol glucoside.

Interestingly, within this cluster, three putative annotations, which are derived from a database of already described FHB-associated metabolites, had to be discarded after careful manual inspection. While the mass of metabolite A.134 fits to the annotation of resistance-related metabolite epicatechin 5-O-beta-D-glucopyranoside-3-benzoate described in the study investigating resistance mechanisms of barley against *Fg* (Bollina et al., 2011), our isotope-assisted workflow (Doppler et al., 2019) allowed us to falsify this annotation as we detected 18 Phe-derived carbons (2 Phe moieties) to be incorporated into the metabolite. Applying our workflow and filtering database hits for metabolites with two C_6_–C_3_ moieties resulted in a single database hit (ChemSpider ID 34497391) with a polyketide-like structure, for which, to the best of our knowledge, its biological role or context are yet unknown. Unfortunately, we were not successful in recording MS/MS fragmentation spectra of this compound. The fact that we have presumably detected the same metabolite as Bollina et al. (2011) and classified this compound to be resistance related together with its identification will be rewarding in future experiments.

Initial annotation of the two isomeric compounds U.2 and U.4 as methyl cinnamate was based on matching accurate mass and total number of carbon atoms against our in-house wheat database. Methyl cinnamate was previously described by Kumaraswamy et al. (2011) to be involved in *Fg*–barley interaction. In the presented study, we were able to not only exclude that the metabolite is a methanol-based solvent artifact (Sauerschnig et al., 2018) but also the annotation as methyl cinnamate was excluded, as neither U.2 nor U.4 showed chromatographic coelution with the authentic cinnamic acid methylester standard.

### Cluster 2

Cluster 2 contains 10 metabolites, which have been assigned to a diversity of chemical structure classes including ferulic acid conjugates [feruloyl-quinic acid hexoside (A.13) or ferulic acid hexoside (A.36)], lignans (A.13, A.80, A.96), flavonoids (A.104 and A.119), and the two compounds A.62 and A.96 containing 8 Phe-derived carbon atoms, which were therefore putatively classified as phenylacetic acid derivatives (**Supplementary Table 3**). All of these compounds exhibited increased abundance levels in the susceptible NIL C4 upon DON treatment, but interestingly, their levels did not significantly change in the resistant NIL C2. The observed increase could be caused by the higher stress level, as DON is metabolized less efficiently in C4 compared to C2. Possibly, these metabolites are basal defense metabolites that are formed out of the same pool of building blocks (i.e., activated HCAs, mainly the coumaroyl-coenzyme A thioester) as metabolites of cluster 1A. The low abundance of these metabolites in C2 could be explained by the production of higher amounts of cluster 1A metabolites in C2, which then can result in a limited availability of substrates and resources for the production of the various cluster 2 metabolites in this genotype.

### Cluster 3

This cluster comprises of 22 metabolites, showing increased abundances after the DON treatment. Only two metabolites were not significantly affected by DON treatment at 96 hat. Manual inspection showed that the metabolites have similar abundance levels in the two genotypes C2 and C4; thus, the presence of the *Fhb1* QTL does neither increase nor decrease the abundances of these Phe metabolites.

Metabolites of this cluster have been classified into various chemical structure classes such as flavonoids, HCA alcohols, -aldehydes, or -glycosides, lignans, and acyl-quinic acids. Many of these metabolites represent intermediates of general defense pathways to different types of stress. Interestingly, most of these compounds show a rapid increase in the resistant parent line CM to reach a maximum abundance level around 48 hat before a subsequent decrease towards 96 hat is observed. Four metabolites were annotated as HCA–quinic acid conjugates with one coumaroylquinate (A.49) and three isomeric feruloylquinates (A.30, A.32, A.54) derived from ferulic acid, constituting the most abundant HCA in wheat. The observed DON-responsive increase in these compounds is consistent with an earlier study of Warth et al. (2015b), which has investigated the same biological samples and found quinic acid to be induced upon DON treatment. The role of acyl-quinic acids *in planta* has recently been summarized by Clifford et al. (2017). The authors proposed that feruloylquinic acids are important indirect plant defense compounds, as they may act as lignin precursors (Nuringtyas et al., 2012). Moreover, acyl-quinic acids have been found to enhance tolerance against oxidative stress, and some of them are capable of inhibiting pathogen-derived cell wall hydrolytic enzymes (Clifford et al., 2017). Our observations of acyl-quinates being accumulated are in good agreement with their proposed biological roles as well as the ribotoxic, stress-inducing, and elicitor-like properties of DON. It is worth noting that chlorogenic acid (5-*O*-caffeoylquinic acid) has also been detected and confirmed with an authentic standard. Surprisingly however, although the DON-induced increase in chlorogenic acid was still classified to be statistically significant at early time points, this acyl-quinate was not part of cluster 3, as its induction was less pronounced over time and tended to be higher in the susceptible NIL C4 compared to C2 (**Supplementary Information 1**).

### Cluster 4

Cluster 4 comprises of 18 metabolites, most of them being annotated as flavonoids. This group of Phe-derived metabolites are constitutively more abundant in the susceptible NIL C4 compared to the *Fhb1* QTL-harboring NIL C2. Except for 2 out of the 12 flavonoids, their abundances were not significantly altered by toxin treatment. For each of the flavonoids, the *Fhb1*-associated constitutive differences in metabolite abundances is also supported by the parent lines CM and Remus, which correspondingly showed lower (CM) and higher (Remus) abundance levels as well as no DON response, respectively [only exception: A.110, annotated as (iso)scoparin 7-O-hexoside or another isomer].

Based on the reduction and oxygenation status at the C2, C3, and C4 position of their central C-ring, flavonoids can be divided into chalcones, dihydroflavones (= flavanones), flavones, dihydroflavonols, flavonols, flavan-3,4-diols, flavan3-ols (= catechins), and anthocyan(id)ins. In cereals, flavones and flavonols constitute the major compound classes (Žilić, 2016). For cluster 4, metabolite annotation/identification resulted in (i) five flavones: apigenin-8-C-glucoside (= vitexin), apigenin 7-O-rutinoside (A.85), (iso)vitexin-7-O-hexoside (A.69 and A.84), and homoorientin; (ii) two flavonols: kaempferol-4′-methylether-3-neohesperidoside (A.92) and quercetin 3-O-methyl-O-hexoside (A.81); and (iii) a single anthocyanin (malvidin-3-hexoside A.117). Although vitexin and homoorientin were confirmed by authentic standards, it should be noted that most flavonoid annotations remain ambiguous. The number of commercially available reference standards is limited, and many different structural isomers exist for each of the assigned sum formulas. However, without the availability of standards or high-quality MS/MS spectra (i.e., sufficiently abundant precursor masses), unambiguous characterization of isomers or classification of flavonoids into structure classes is not feasible. Here, we have complemented metabolite annotations by molecular network analysis of MS/MS fragment spectra according to Watrous et al. (2012). Similarity-based correlation of MS/MS spectra connected five of the annotated flavonoids of this cluster to the identified vitexin and homoorientin, which allowed to annotate all respective metabolites as glycosylated flavonoids (**Supplementary Table 3**).

For the selection of single isomers taken for the annotations listed above, we also considered and prioritized former studies, which had reported the respective compounds to be involved in *Fusarium*– or DON–plant interactions [reviewed in Gauthier et al. (2015)]. Surprisingly, 10 of the 12 annotated flavonoids assigned to this cluster have been previously described to be related to plant defense against FHB. Moreover, vitexin was described to be involved in plant defense by Kumaraswamy et al. (2012), who compared the metabolic reaction of barley upon infection with a DON-producing and a non-DON-producing *Fg* strain. In contrast to the literature reports, we did not find these flavonoids to be defense-induced or directly associated with *Fhb1*-mediated resistance against DON. Since the published studies have investigated *Fg*–wheat or *Fg*–barley interactions, it is possible that, in contrast to *Fg*, pure DON does not trigger the accumulation of these compounds in wheat. Alternatively, the flavonoids, contained in cluster 4 have biological functions other than defense.

However, since such a large number of flavonoids were already reported to be involved in resistance against *Fusarium*/DON, we have extended the screening of this class of compounds to additional authentic flavonoid reference standards. With this approach, we were also able to exclude catechin, naringenin, naringenin-7-O-glucoside, and quercetin, which all had been classified as FHB related, to be involved against DON-induced stress, as none of them were detectable under the tested conditions.

Overall, the automated data evaluation according to the described isotope-assisted workflow resulted in identification of three metabolites and annotation of a total of 44 different flavonoids in our study. Interestingly, and in contrast to the annotated anthocyanin, flavones and flavonols of cluster 4, some flavanones (A.119; A.111; A.60 and A.104) were linked to the time course clusters 2 and 3, which contain metabolites that were induced upon toxin treatment. As already pointed out above, while the classification of these compounds as flavonoids can be considered reliable, multiple chemical structures fit the search criteria; thus, biological interpretation has to be handled with care. For example, three different feature groups (i.e., metabolites) corresponded to 5′-prenylhomoeriodictyol (A.100, A.111, and A.119), a metabolite previously described to be related to FHB. The three isomers show different time course profiles (A.100: no cluster; A.111: cluster 3 and A.119: cluster 2), which complicates biological interpretation. As flavonoids represent a highly diverse class of secondary metabolites with different biological functions, further investigations are required to differentiate between the various biological roles and unravel their contribution to plant defense/resistance against DON in more detail.

### Remaining Clusters/Metabolites

Several other identified metabolites can be expected to be related to plant defense upon DON treatment and shall at least briefly be mentioned. Ferulic acid, for example, is known to be the most prominent HCA in wheat (Klepacka and Fornal, 2006). In our experiment, ferulic acid was identified and also monitored over time but did not show clear differences between the treatments or genotypes (**Supplementary Information 1**). This behavior could be explained by (i) the formation of numerous derivatives such as feruloylquinic acid conjugates, ferulic acid glucosides, etc. from this central metabolite (de O. Buanafina, 2009) (ii) its polymerization into cell wall constituents, or (iii) masking of differing but high turnover rates, which are not directly reflected in metabolite abundances. A similar behavior was observed for p-coumaric acid or sinapaldehyde or sinapyl alcohol, which are also likely to have high turnover rates, as they are known intermediates in lignin biosynthesis (Vanholme et al., 2010).

## Summary

Combining metabolomics approaches for global- and tracer-based isotopic labeling allowed an untargeted yet guided view into the Phe-derived submetabolome of wheat plants and its perturbation upon DON stress. We were able to confirm and critically assess previously known metabolites. In addition, we have also detected a number of unknown Phe-derived metabolites, which may at least partly constitute novel biochemical constituents of wheat. Consideration of the number of carbon atoms enabled unambiguous assignment to Phe metabolism and supports annotation although identification remains difficult due to lack of reference standards.

Statistical evaluation of the biological time course experiment showed that many of the Phe-derived wheat metabolites were affected by the tested DON treatment, and our untargeted tracer approach was used to relate individual compounds to the major resistance QTL *Fhb1*. To this end, both kinetics of metabolite formation and the extent of metabolite accumulation have been followed over the first 96 h after toxin treatment.

**Figure 5:**
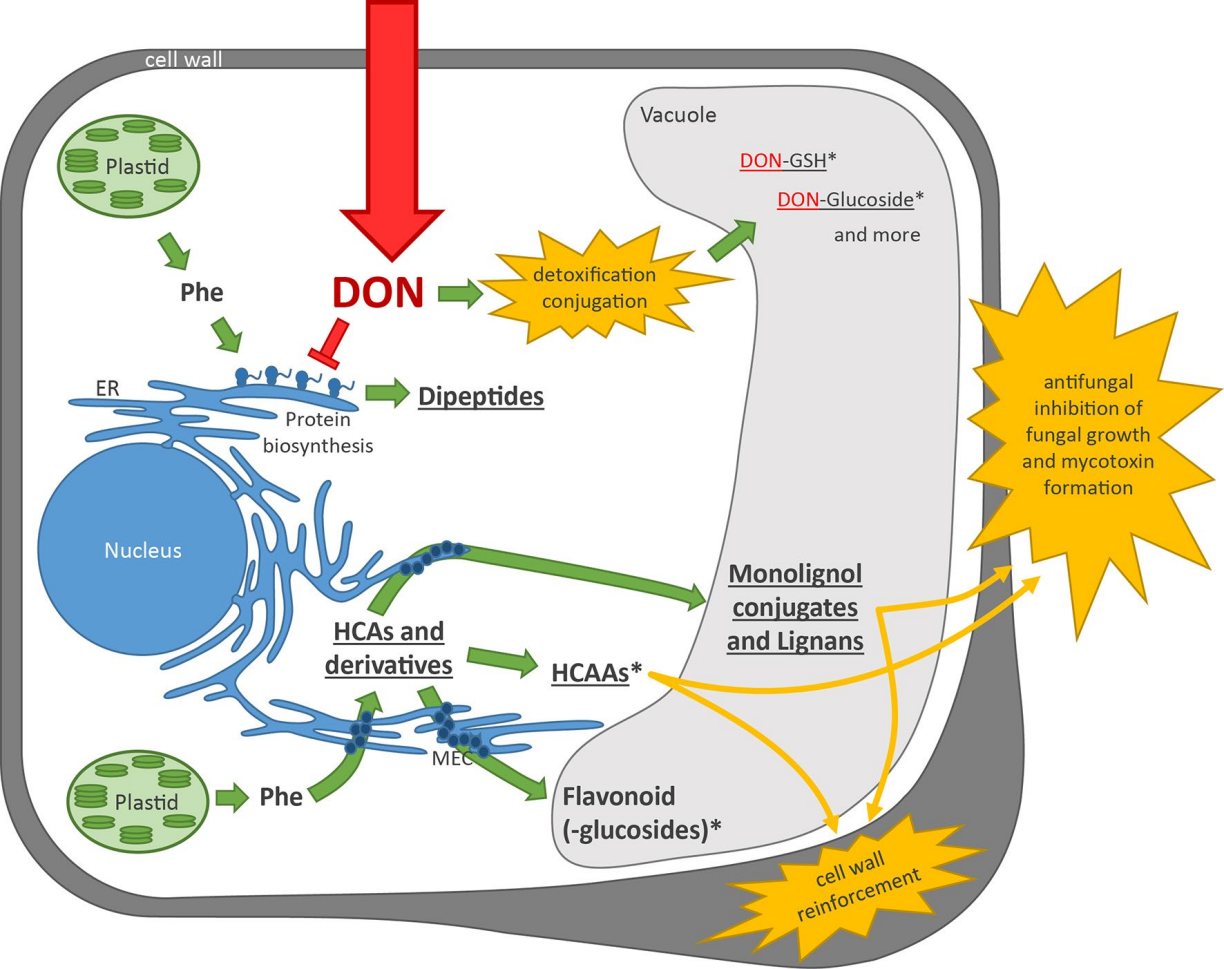
Plant cell model showing the plants’ reaction on DON treatment. Green arrows indicate metabolic activity of the plant, and red indicates DON and its mode of action. Underlined metabolite (classes) indicate upregulation upon DON treatment observed in this experiment and in previous experiments (Kluger et al., 2015). *indicates an Fhb1 QTL effect. Yellow explosion: plant defense responses. DON, deoxynivalenol; Phe, phenylalanine; ER, endoplasmic reticulum; GSH, glutathione; HCA, hydroxycinnamic acid; HCAA, hydroxycinnamic acid amide; MEC, multienzyme complex.

Based on our results, previous studies on the same sample set, and the current literature, we propose the following model for the defense reaction of wheat on DON treatment (**Figure 5**): After DON enters the plant cell, it binds to the ribosome and inhibits protein biosynthesis (Walter et al., 2010). Phe is used as a building block for proteins. DON is—depending on the concentration—*in vivo* most likely primarily acting as a translation elongation inhibitor (Cundliffe and Davies, 1977). Under these conditions, truncated proteins are formed that have to be degraded. It has previously been shown that ubiquitin proteasome-mediated degradation is limiting DON resistance in bakers’ yeast (Abolmaali et al., 2008). The peptides generated by the proteasome are then presumably further degraded by cytosolic and lysosomal endo- and exopeptidases. It is known that the plant proteasome is modified during stress conditions (Kovács et al., 2017), but little is known about peptides released by the proteasome. Some proteasome subunits cleave proteins like chymotrypsin after bulky aromatic amino acids (including Phe), so that presumably, the observed DON-induced dipeptides are expected to have Phe at the C terminus (X-Phe). This could not be fully confirmed. Manual inspection of MS/MS fragmentation patterns as for A.35 fragments of either Glu-Phe (y2 and b1) as well as Phe-Glu (a1) were detected, which suggests coelution of the two peptides. Alternatively, dipeptides could be formed by dipeptidyl-peptidases, acting like mammalian cathepsin C, which removes dipeptides from the N terminus of proteins. Further work is necessary to elucidate the metabolic route(s) of dipeptide formation in detail.

In parallel, the plant detoxifies DON by conjugation to glucose, glutathione (Kluger et al., 2015), and sulfate (Warth et al., 2015a). Presumably, these conjugates can further be modified and incorporated into biopolymers. DON detoxification products were confirmed based on the GML approach in this study. *Fhb1* plays a significant role in this defense reaction by a more rapid and efficient detoxification of DON, as was shown in the studies of Kluger et al. (2015).

Depending on its effective concentration, DON can induce broadly based defense reactions, including the production of pathogenesis-related-proteins, jasmonate signaling, H_2_O_2_ production (Desmond et al., 2008; Gunupuru et al., 2017), and induction of the shikimate pathway (Warth et al., 2015b). Activation of the shikimate pathway leads to an increased formation of the aromatic amino acids Trp, Tyr, and Phe. The latter is the substrate of the enzyme PAL, which catalyzes the first step of the general phenylpropanoid pathway from Phe to cinnamic acid. PAL is generally stress inducible, and for the cultivars Remus and CM, gene transcription has been shown to be triggered by DON (Walter and Doohan, 2011). Numerous secondary metabolites are located downstream of Phe that can potentially be used to mitigate DON-induced stress. In the present study, putative Phe-derived flavonoids, monolignols, lignans, coumarins, HCAAs, and HCA–quinic acid conjugates were found, many of which were accumulating after treatment with DON. Similar to earlier studies, which had employed treatment with the fungal pathogen *Fg* (Gunnaiah et al., 2012), we found numerous HCAAs to be induced in all tested wheat lines by treatment with pure DON as well. In the wheat lines carrying *Fhb1*, HCAAs were both more rapidly induced and accumulating to higher levels, suggesting a role of these compounds in *Fhb1*-mediated type II resistance against DON. The accumulating HCAAs may act as antifungal and antioxidative agents, but most likely, they are mainly transported across the cell membrane and used for lignification of the cell wall (Brar et al., 2019). Conversely, various ferulic acid conjugates and a few flavonoids accumulated in the susceptible NIL C4, while their levels did not significantly change in the resistant NIL C2. Moreover, in contrast to former studies on FHB, which had found many flavonoids such as flavones and flavonols to be involved in resistance against *Fg* in wheat and barley, our data do not support these findings for the pure DON in wheat. Under the applied conditions, some of the formerly reported flavone and flavonol conjugates were even below the limit of detection. In addition, most of the detected flavonoids were not affected by treatment with the pure toxin but were constitutively more abundant in the susceptible NIL C4 (no *Fhb1* present). We therefore conclude that these compounds do not seem to play a major role against DON-induced stress in wheat.

## Note Added In Proof

Very recently, two conflicting studies on *Fhb1* have been published. While both reports found the same gene, they claim different mechanisms underlying *Fhb1* mediated resistance (Lagudah and Krattinger, 2019; Li et al., 2019; Su et al., 2019)

## Data Availability

All datasets generated for this study are included in the article/**Supplementary Material**.

## Author Contributions

MD, BK, CB, RK, GA, ML, and RS contributed to the conception and design of the study. ML, BS, HB, BK, and MD performed the biological experiment. MD, BK, HB, ML, and RS did the plant labeling. MD and BK carried out the measurements. CB, MD, BM, and BK evaluated data. All authors contributed to writing the manuscript and approved the final version.

## Funding

The authors want to thank the Austrian Science Fund (project SFB Fusarium F3715, F3711, and F3701) and the Provincial Government of Lower Austria (projects NoBiTUM, OMICS 4.0).

## Conflict of Interest

The authors declare that the research was conducted in the absence of any commercial or financial relationships that could be construed as a potential conflict of interest.
