# Supplementary material for: Stable Isotope-Assisted Plant Metabolomics: Investigation of Phenylalanine-Related Metabolic Response in Wheat Upon Treatment With the Fusarium Virulence Factor Deoxynivalenol
Source: Front Plant Sci. 2019 Oct 30;10:1137. doi: 10.3389/fpls.2019.01137 (PMC6831647; doi:10.3389/fpls.2019.01137)

Supplementary Information 1:

This supplementary figure shows an overview of all Phe-derived metabolites (n=172) detected in the above shown publication. Each page illustrates the time course (mean values) of the metabolite abundances in the different treatments and/or genotypes respectively. Moreover, “QTL” or “TR+/-” labels indicate if there is a significant difference between the time points and/or genotypes. Please refer to the materials and methods part of the main text for the calculation of these indicator labels.

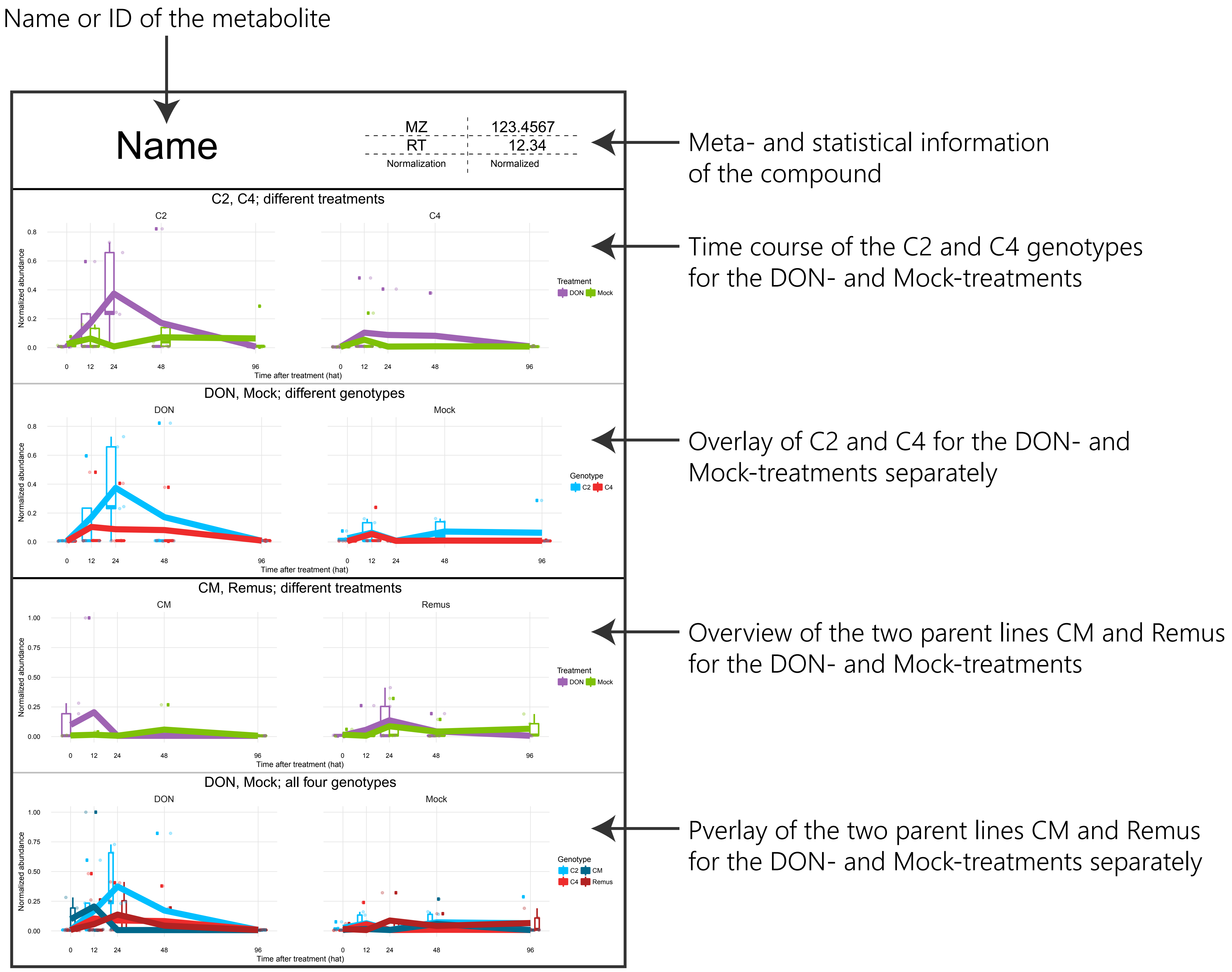

A.3

Annotated as HCA derivative
(3 database hits)

MZ	166.0866
RT	4.11 min
Normalization	Not normalized
Cluster	–
Cn total / Phe	9 / 9

C2, C4; different treatments

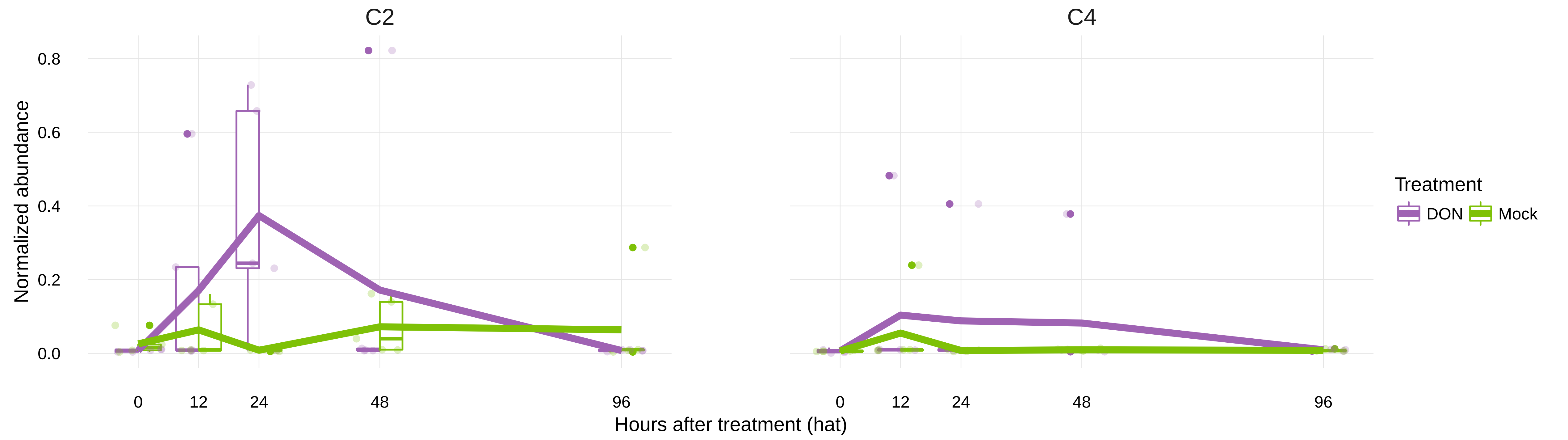

DON, Mock; different genotypes

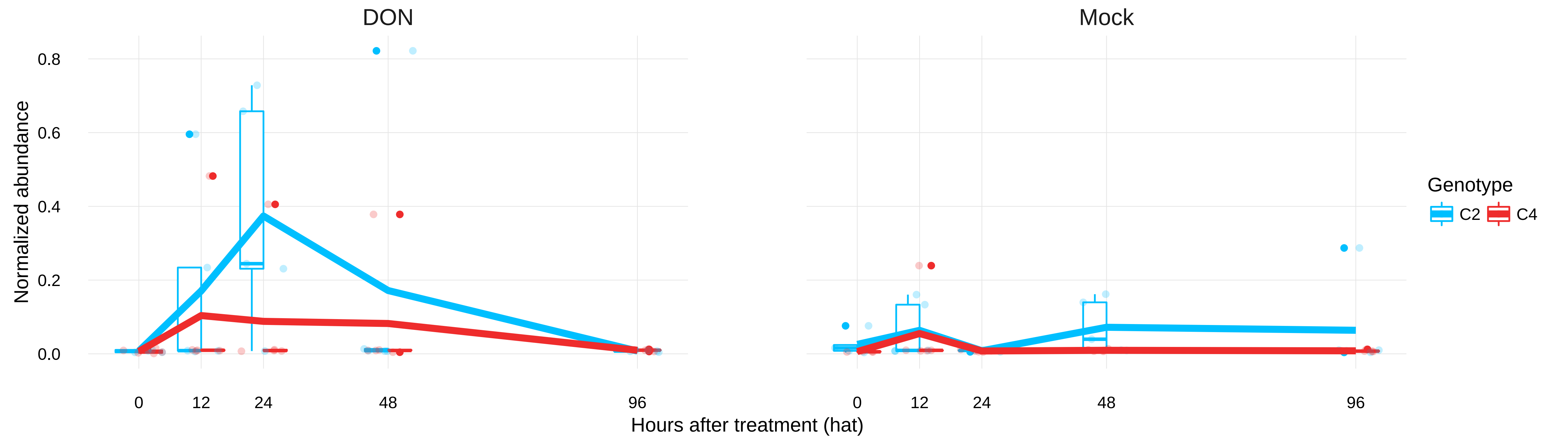

CM, Remus; different treatments

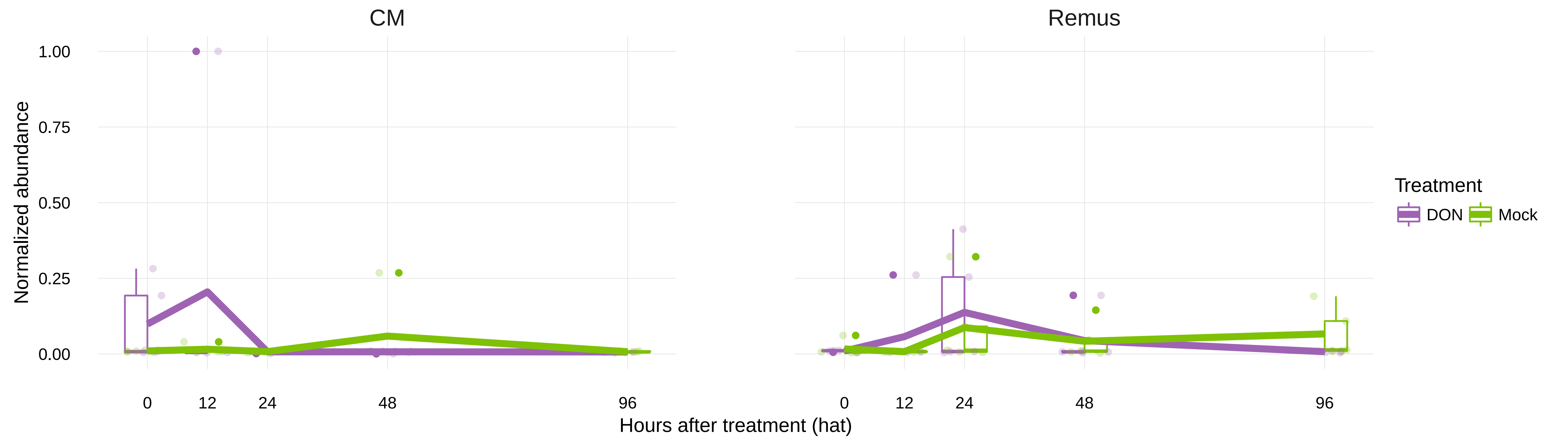

DON, Mock; all four genotypes

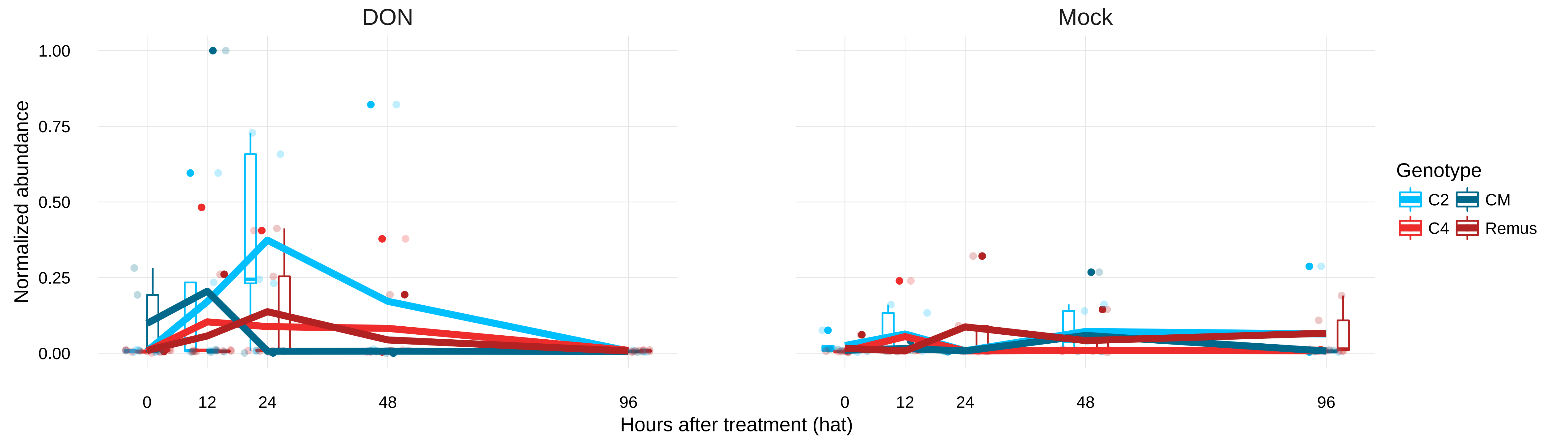

A.4

Annotated as HCA derivative

(3 database hits)

MZ	149.06
RT	4.12 min
Normalization	Not normalized
Cluster	–
Cn total / Phe	9 / 9

C2, C4; different treatments

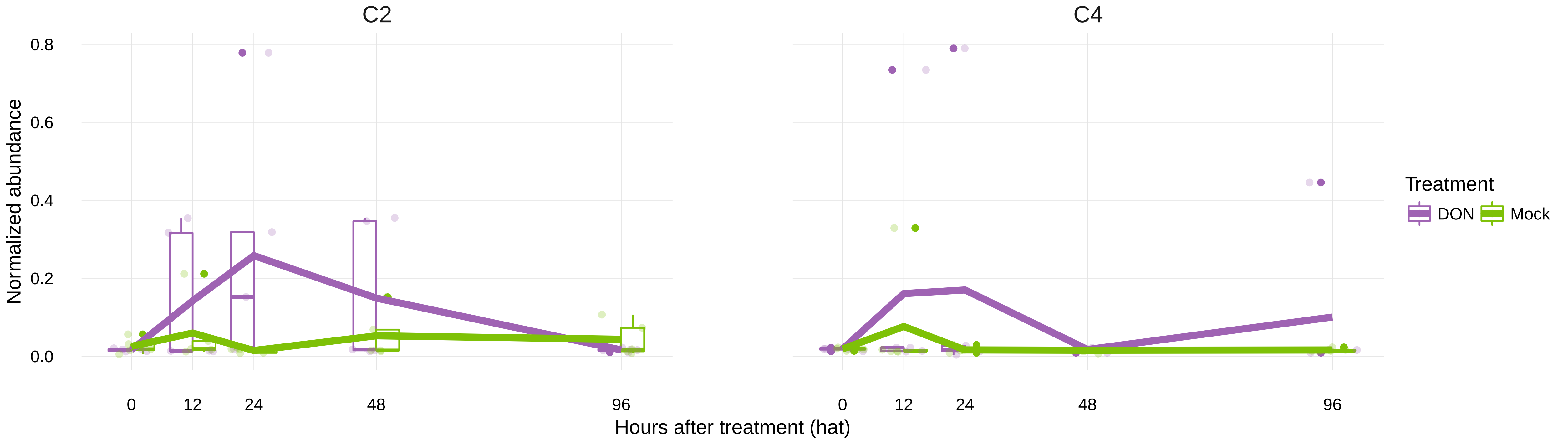

DON, Mock; different genotypes

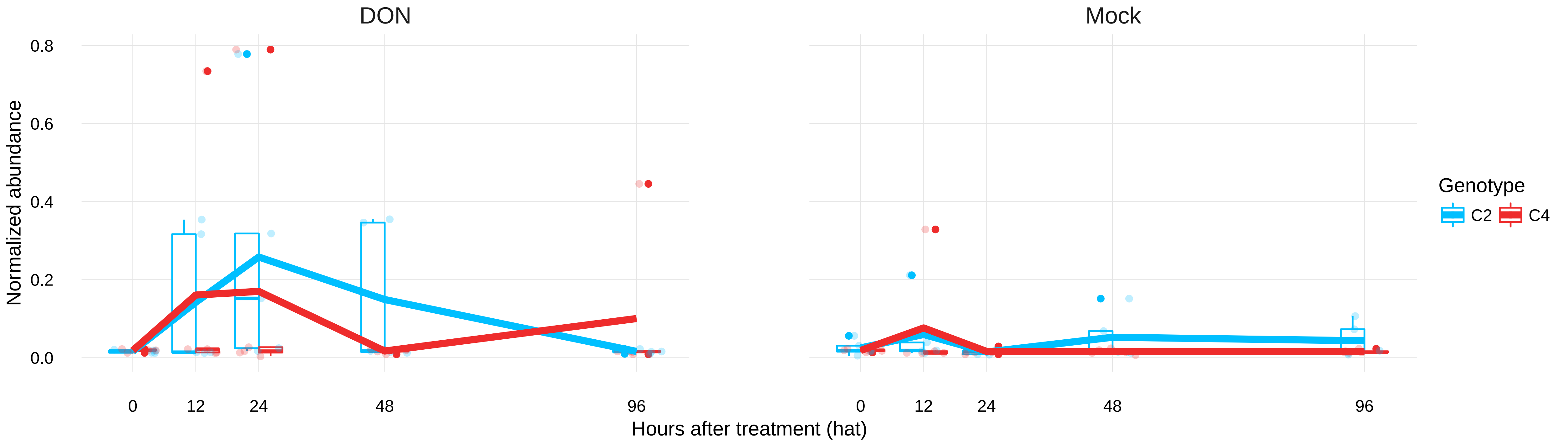

CM, Remus; different treatments

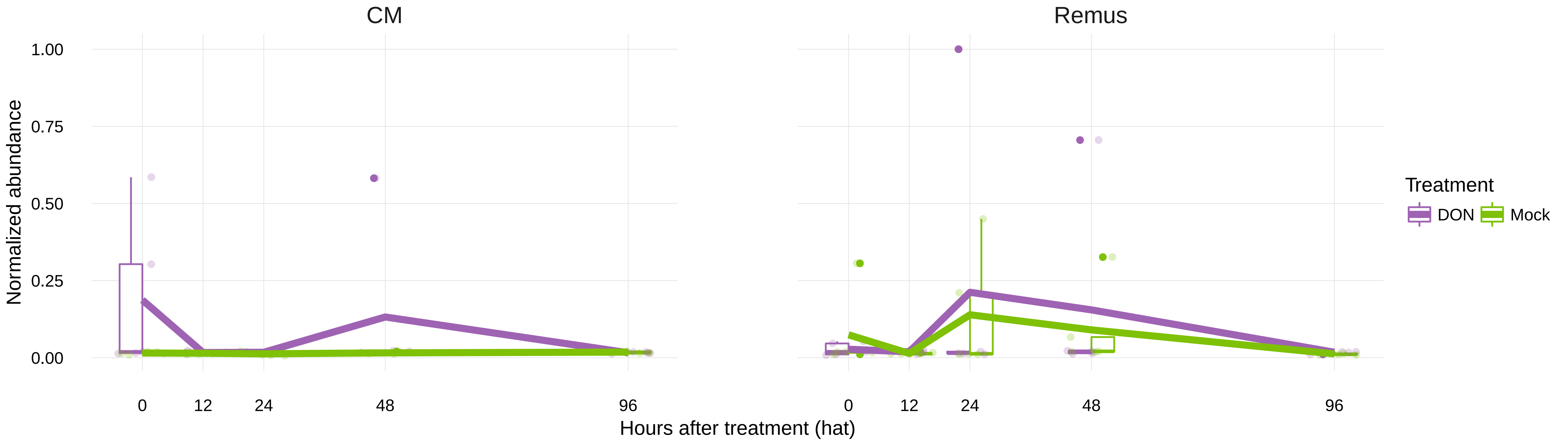

DON, Mock; all four genotypes

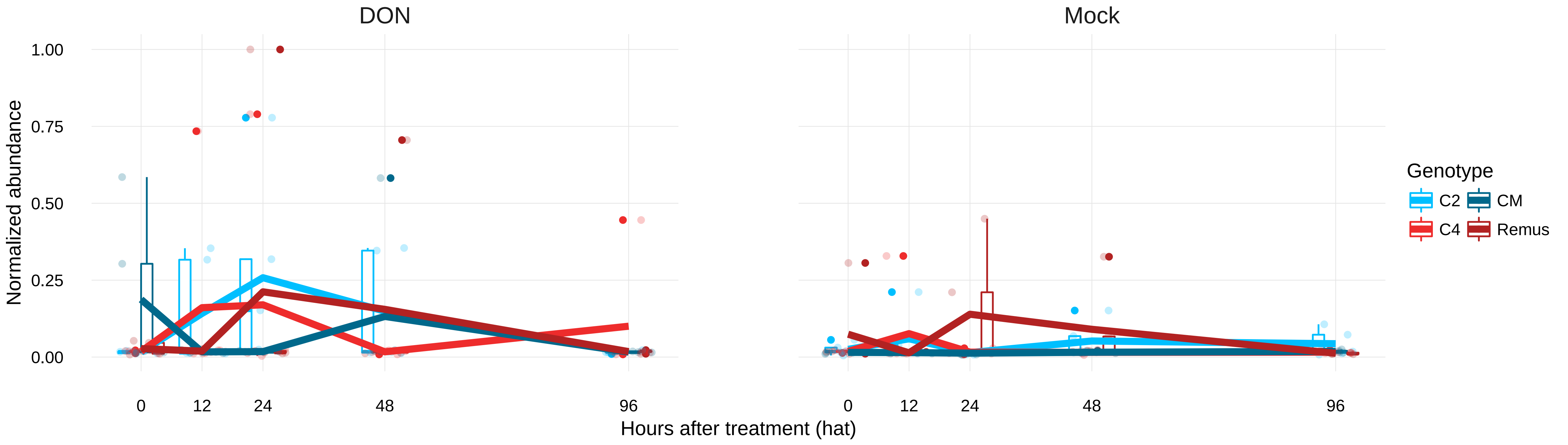

Ferulic acid

Identified metabolite (level 1)

MZ	195.0653
RT	14.05 min
Normalization	Indirectly via surrogate in the KPX samples
Cluster	–
Cn total / Phe	10 /

C2, C4; different treatments

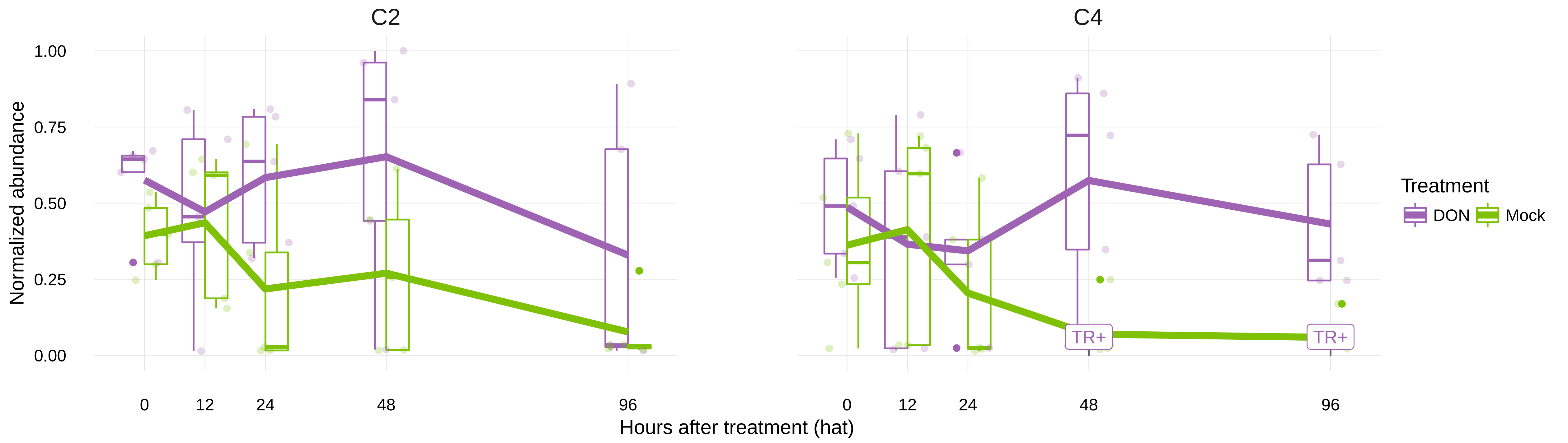

DON, Mock; different genotypes

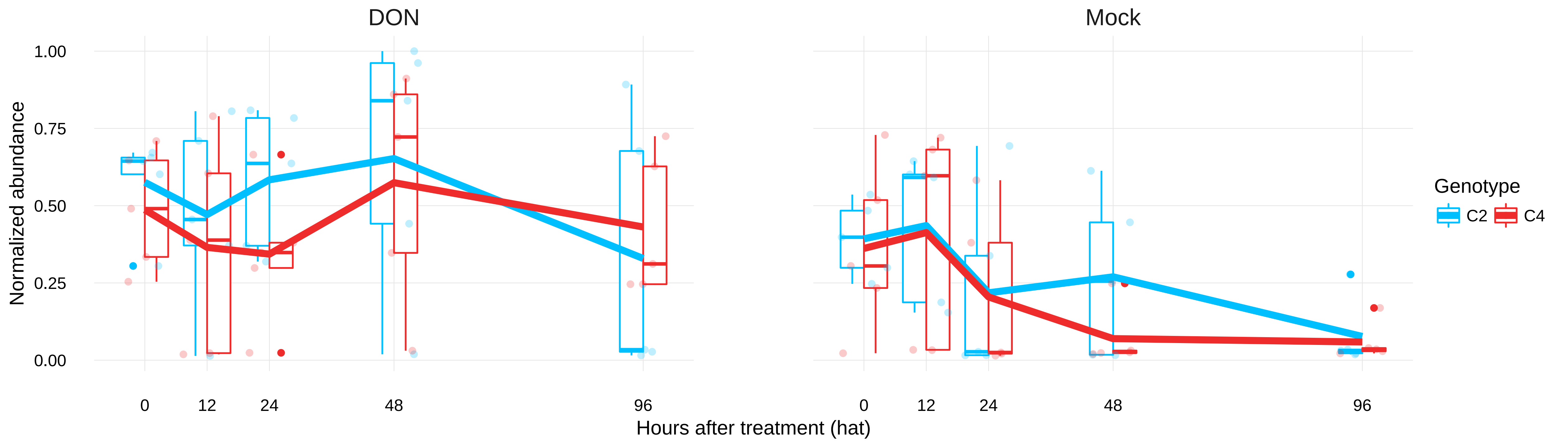

CM, Remus; different treatments

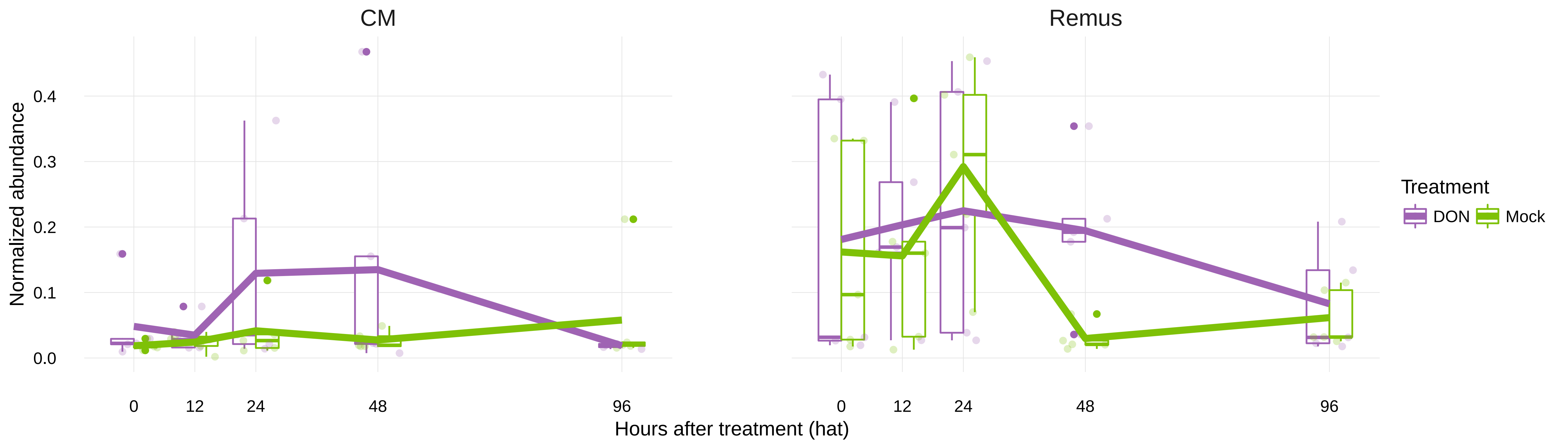

DON, Mock; all four genotypes

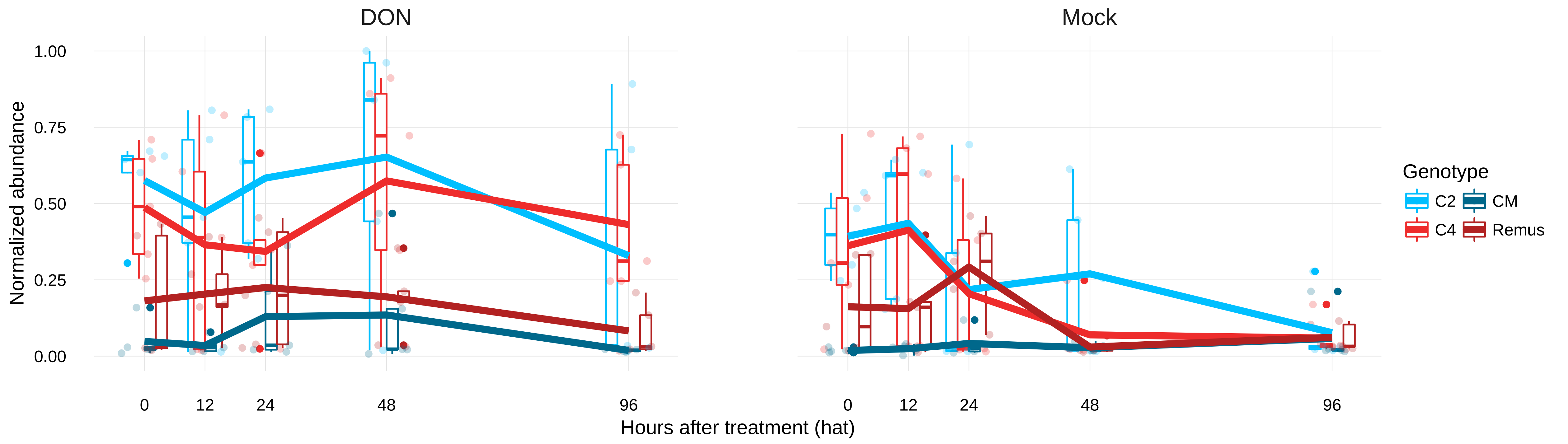

A.1

Annotated as others (Phenol)
(1 database hit)

MZ	144.0656
RT	3.02 min
Normalization	Not normalized
Cluster	–
Cn total / Phe	6 /

C2, C4; different treatments

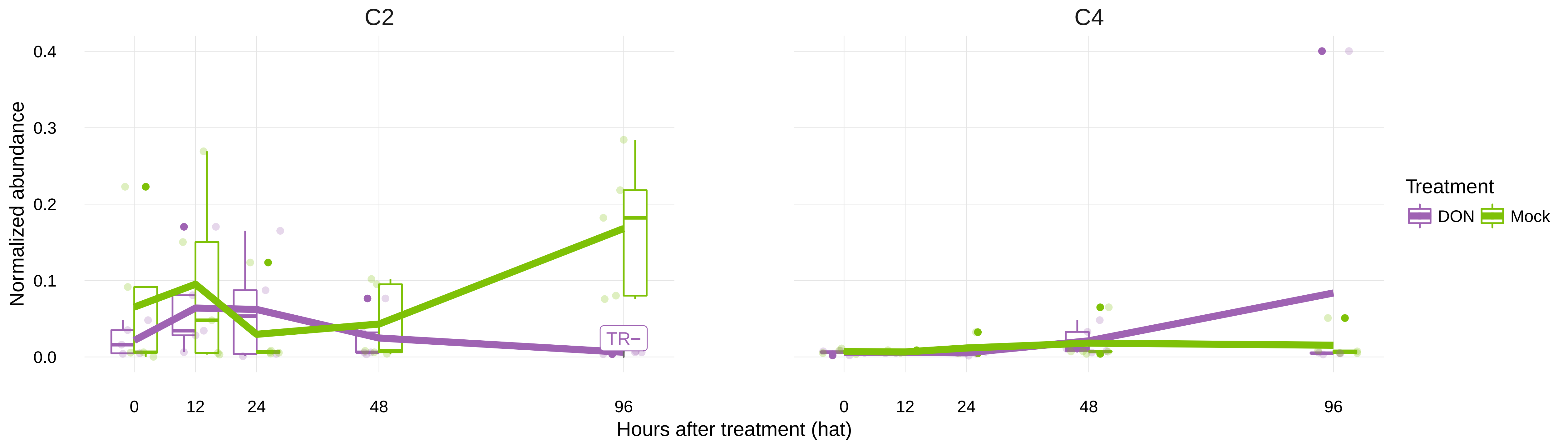

DON, Mock; different genotypes

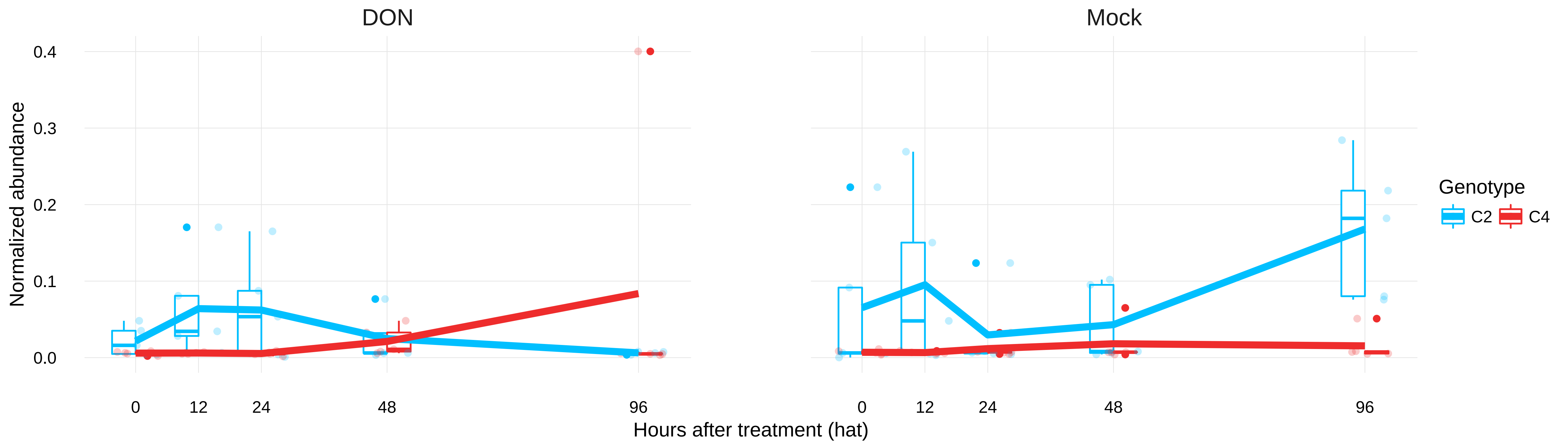

CM, Remus; different treatments

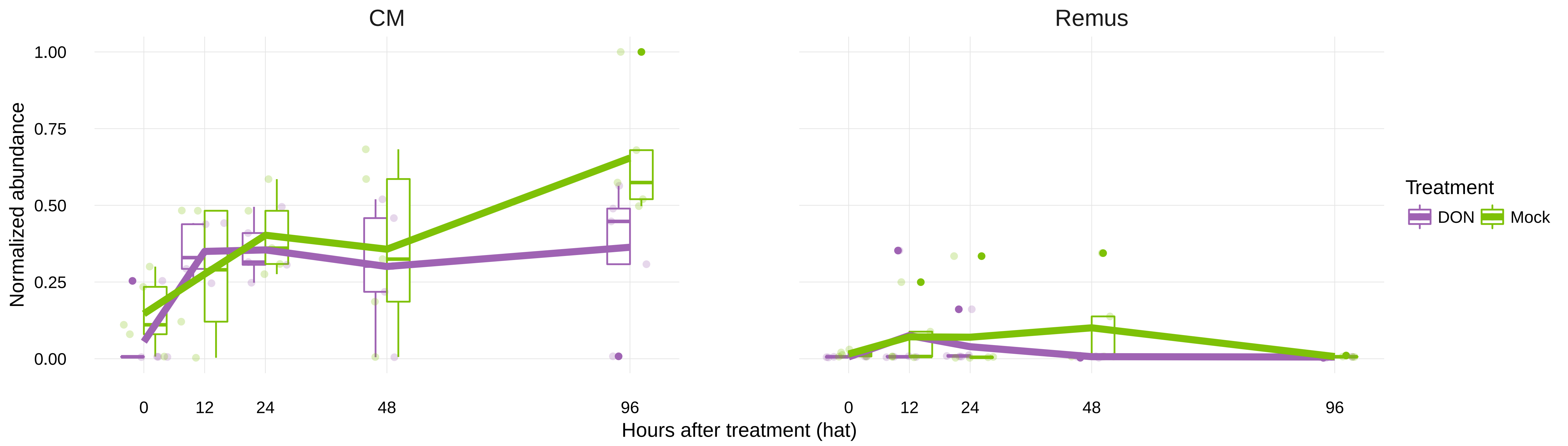

DON, Mock; all four genotypes

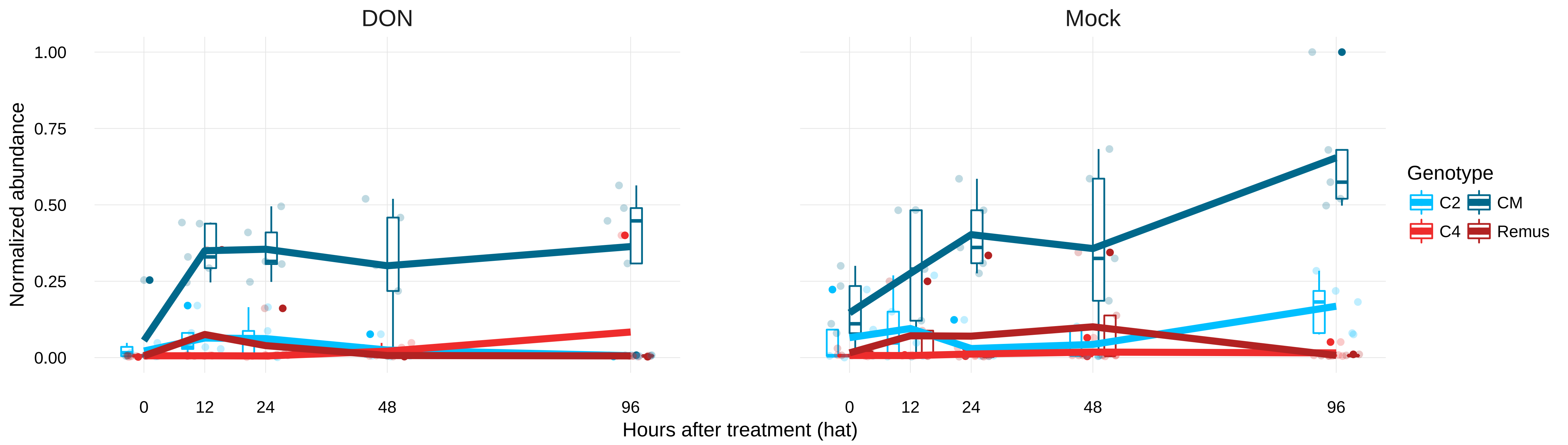

p−coumaric acid

Identified metabolite (level 1)

MZ	165.0546
RT	13.41 min
Normalization	Indirectly via surrogate in the KPX samples
Cluster	−
Cn total / Phe	9 /

C2, C4; different treatments

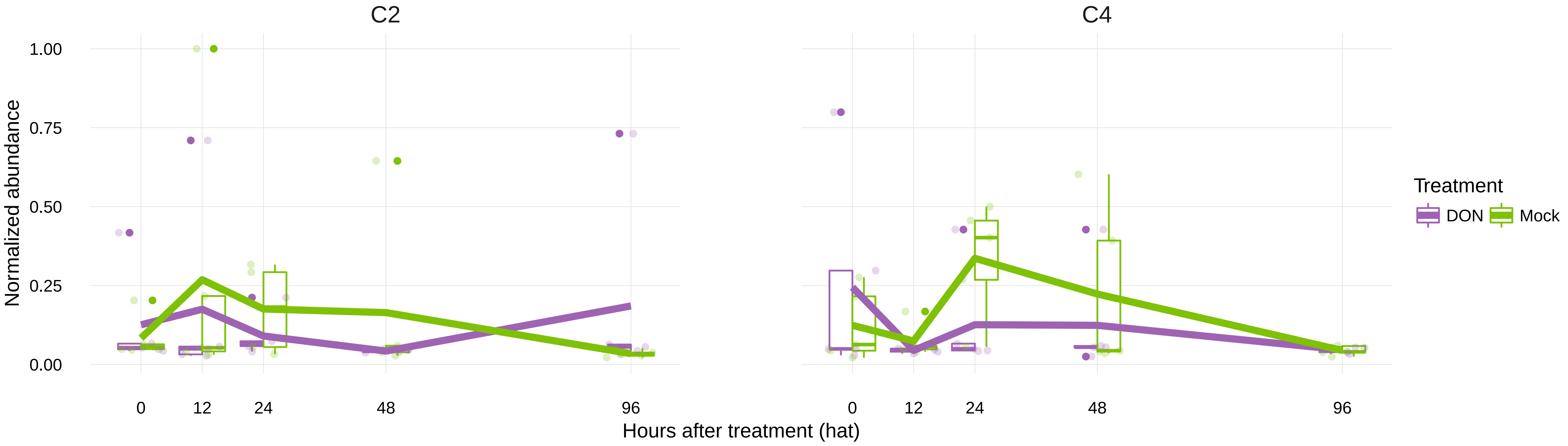

DON, Mock; different genotypes

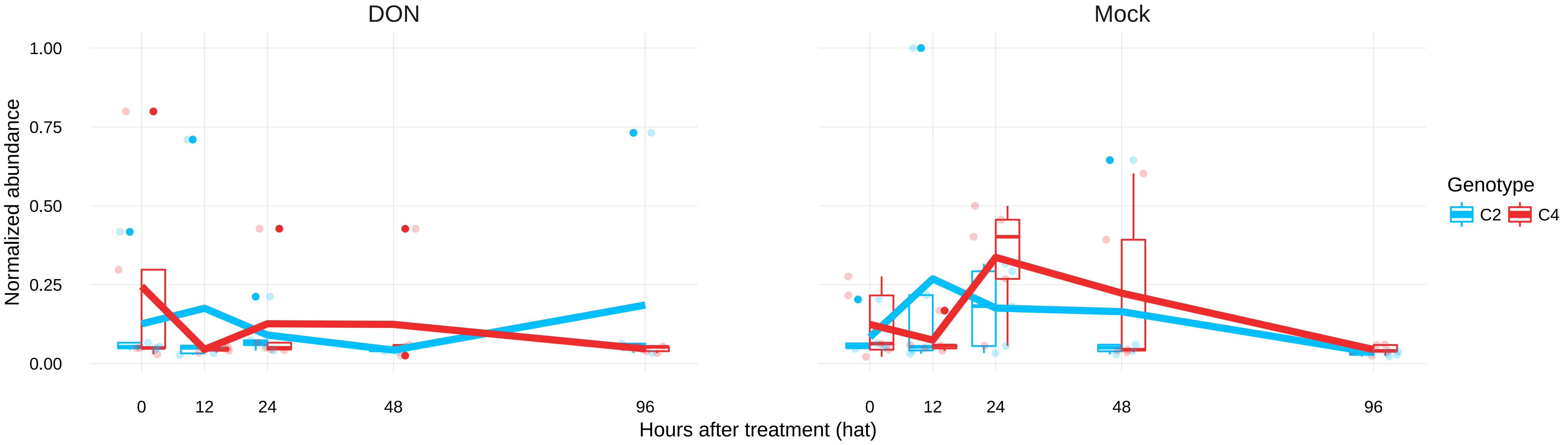

CM, Remus; different treatments

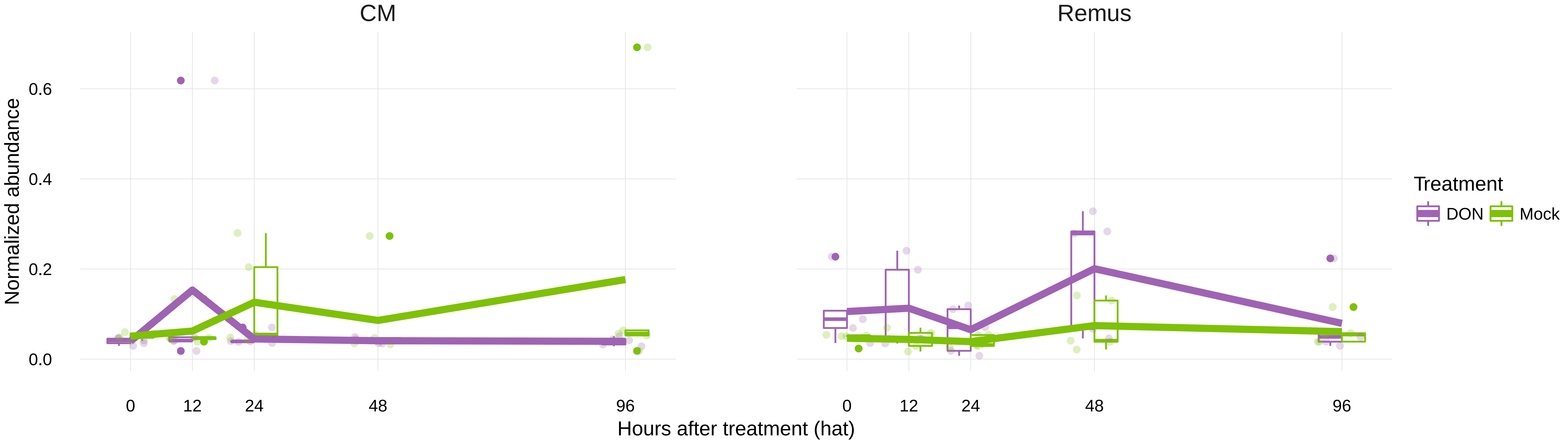

DON, Mock; all four genotypes

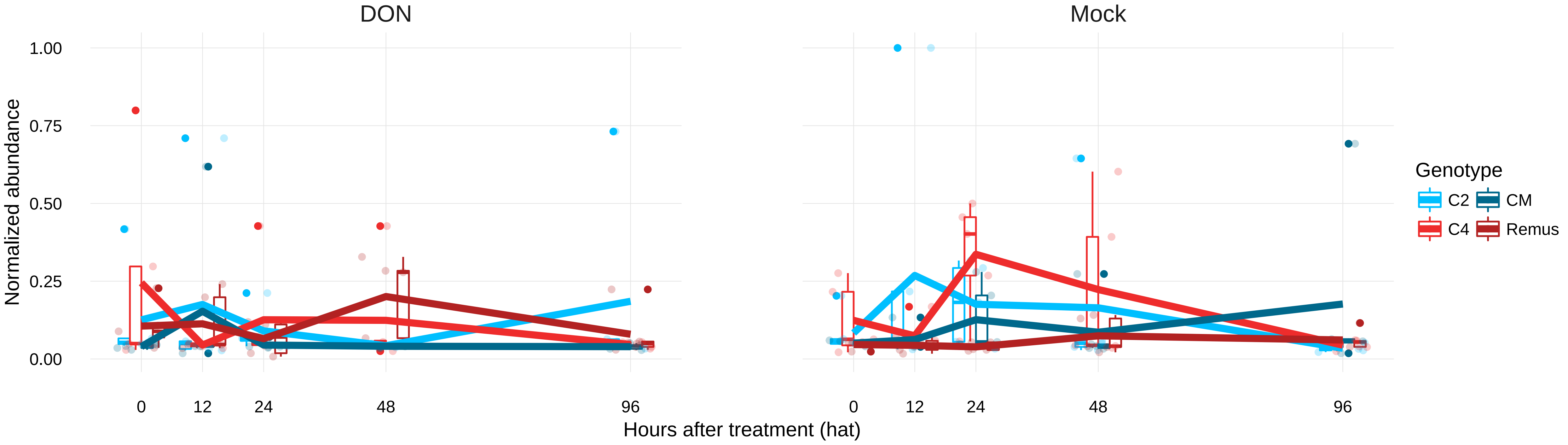

A.51

Annotated as others (Iridoid)
(1 database hit)

MZ	531.1474
RT	12.04 min
Normalization	Indirectly via surrogate in the KPX samples
Cluster	–
Cn total / Phe	24 /

C2, C4; different treatments

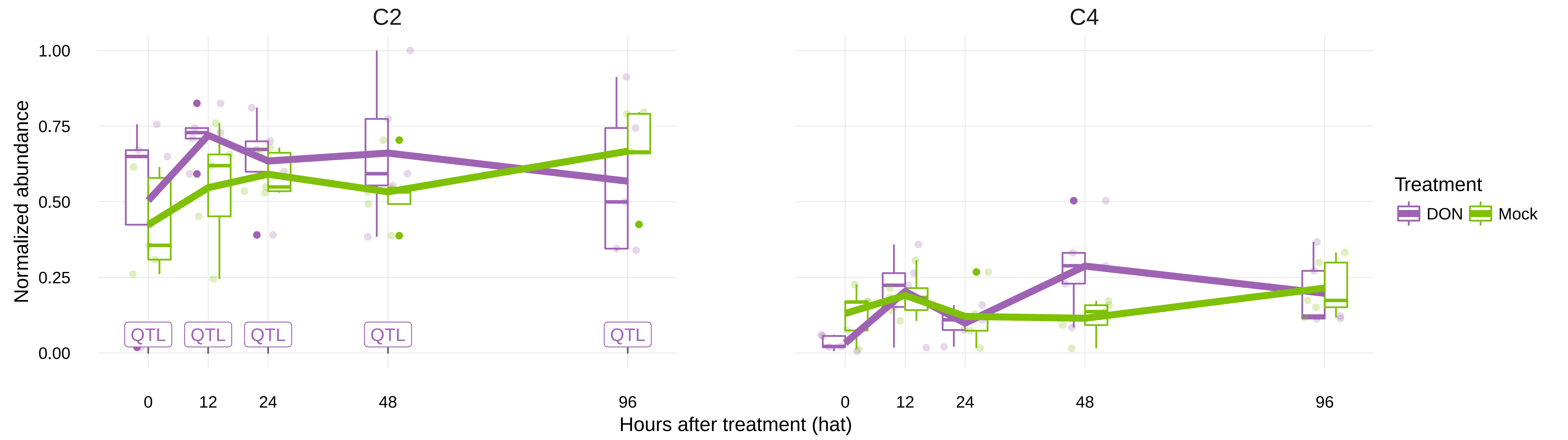

DON, Mock; different genotypes

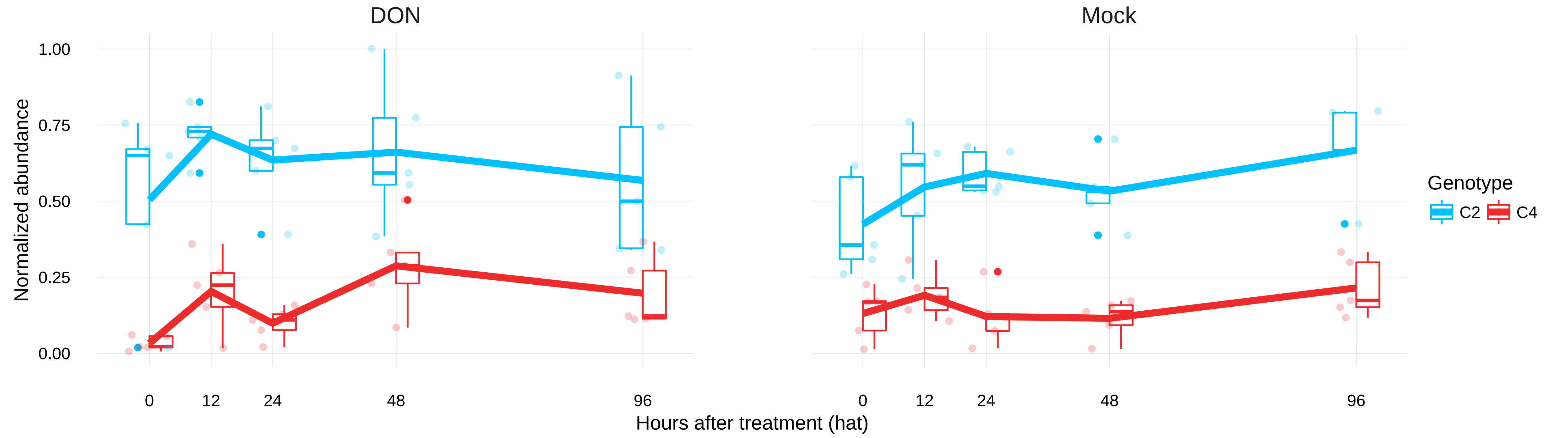

CM, Remus; different treatments

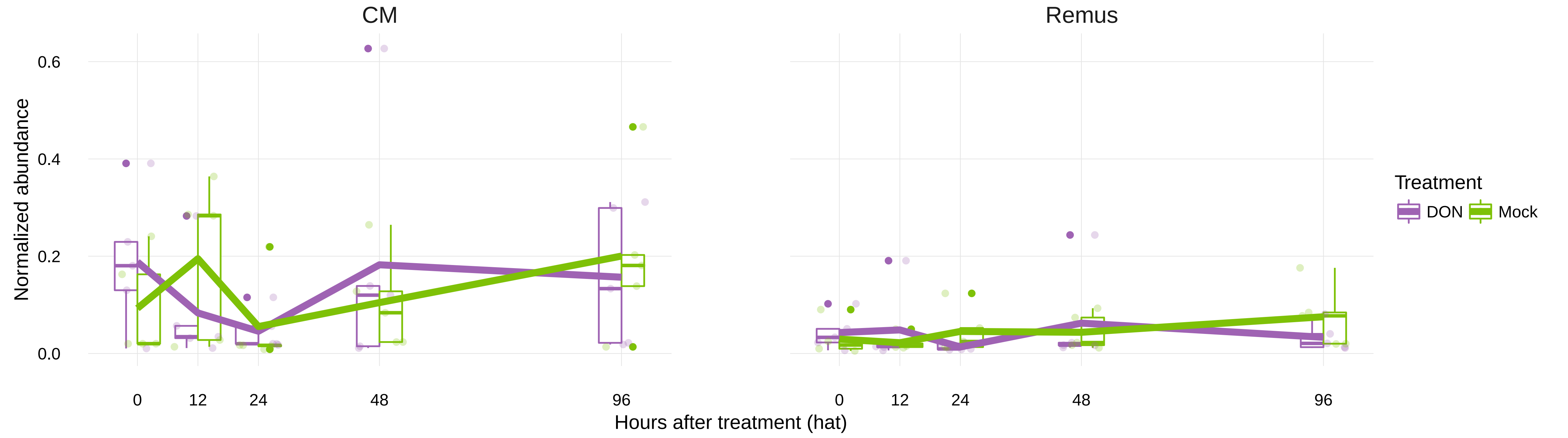

DON, Mock; all four genotypes

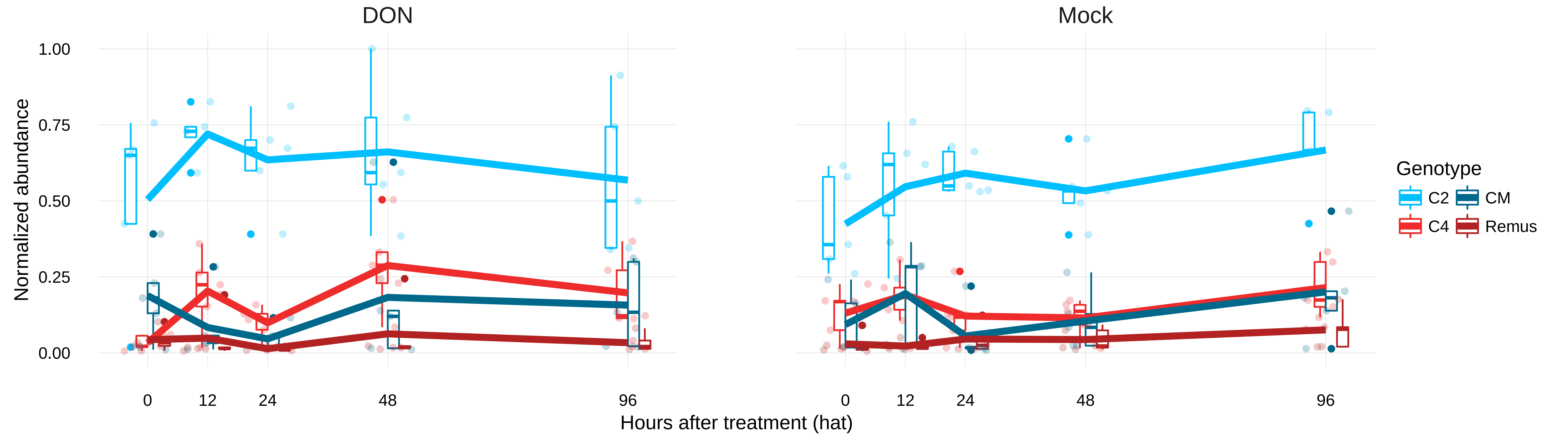

A.139

Annotated as HCA derivative

(1 database hit)

MZ	317.1725
RT	25.54 min
Normalization	Directly via KPX samples
Cluster	–
Cn total / Phe	17 /

C2, C4; different treatments

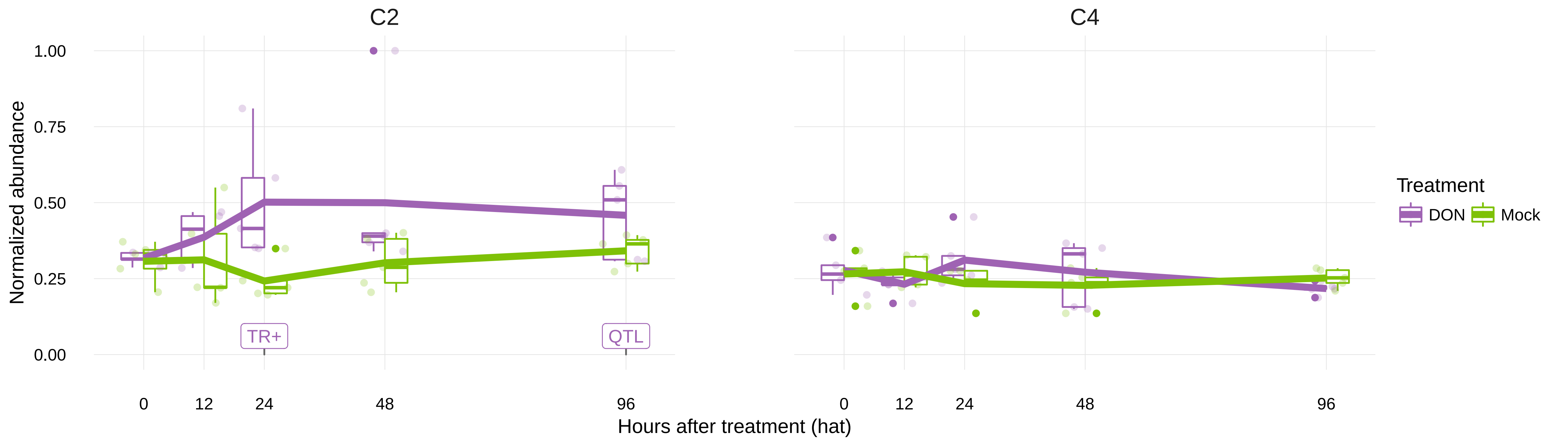

DON, Mock; different genotypes

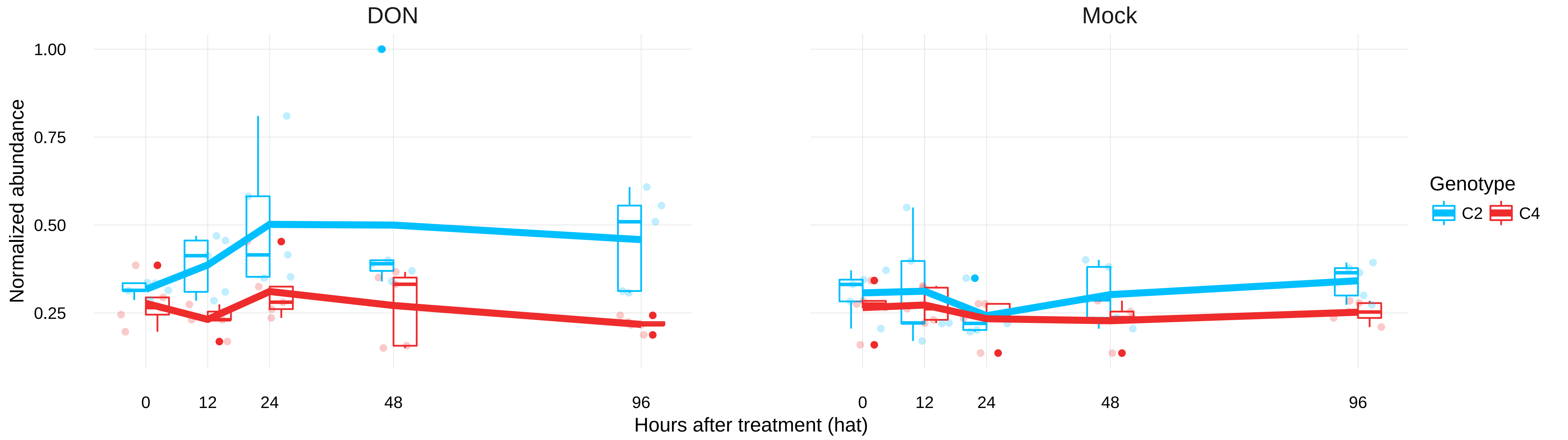

CM, Remus; different treatments

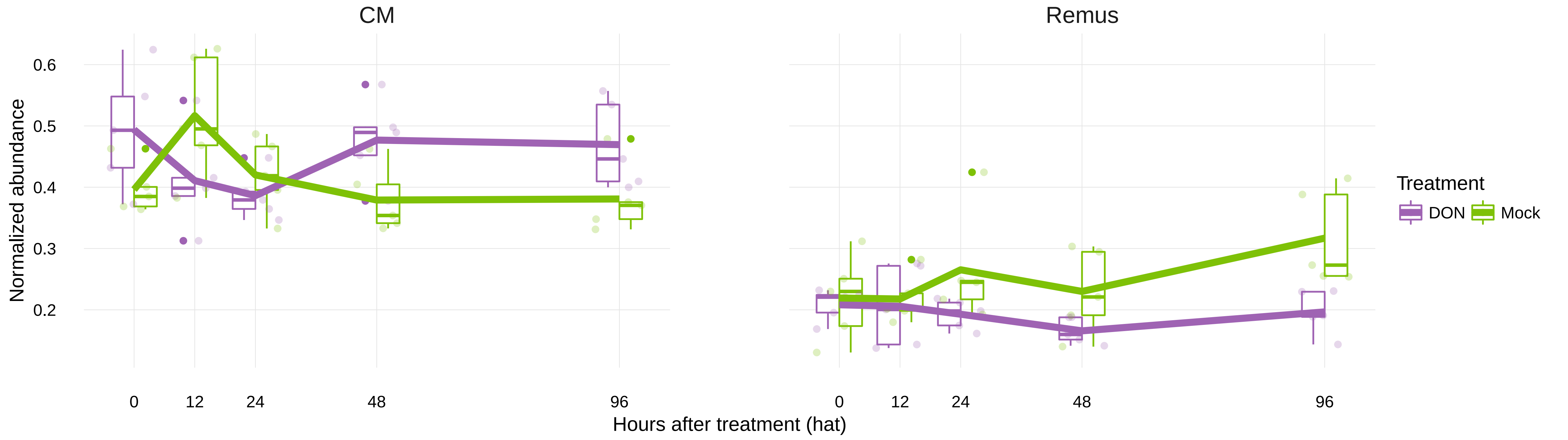

DON, Mock; all four genotypes

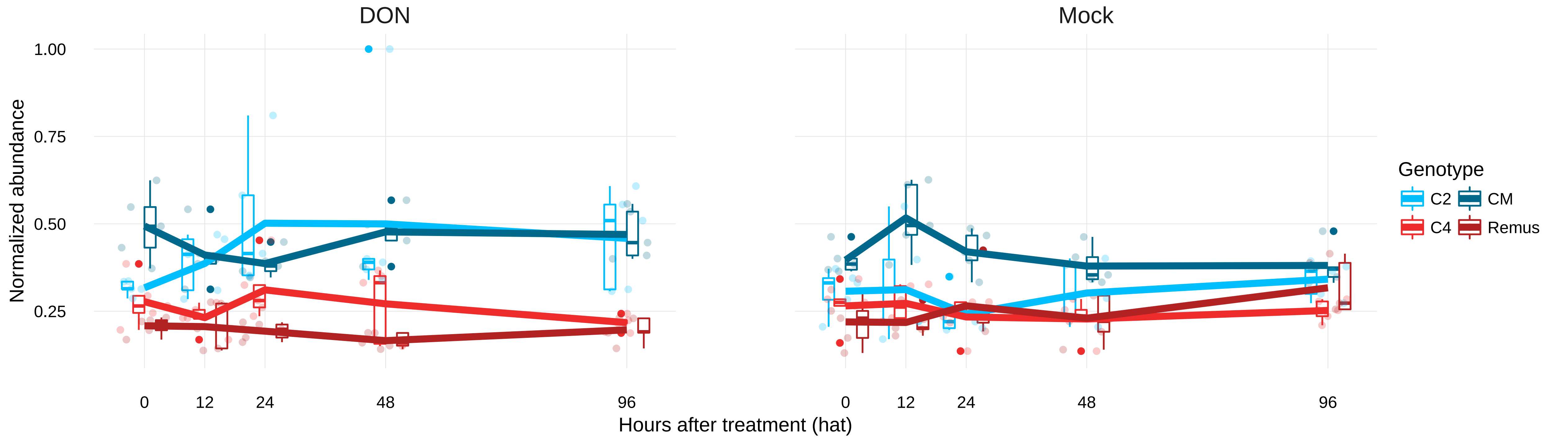

A.10

Annotated as others

(4 database hits)

MZ	169.0495
RT	5.58 min
Normalization	Not normalized
Cluster	–
Cn total / Phe	8 /

C2, C4; different treatments

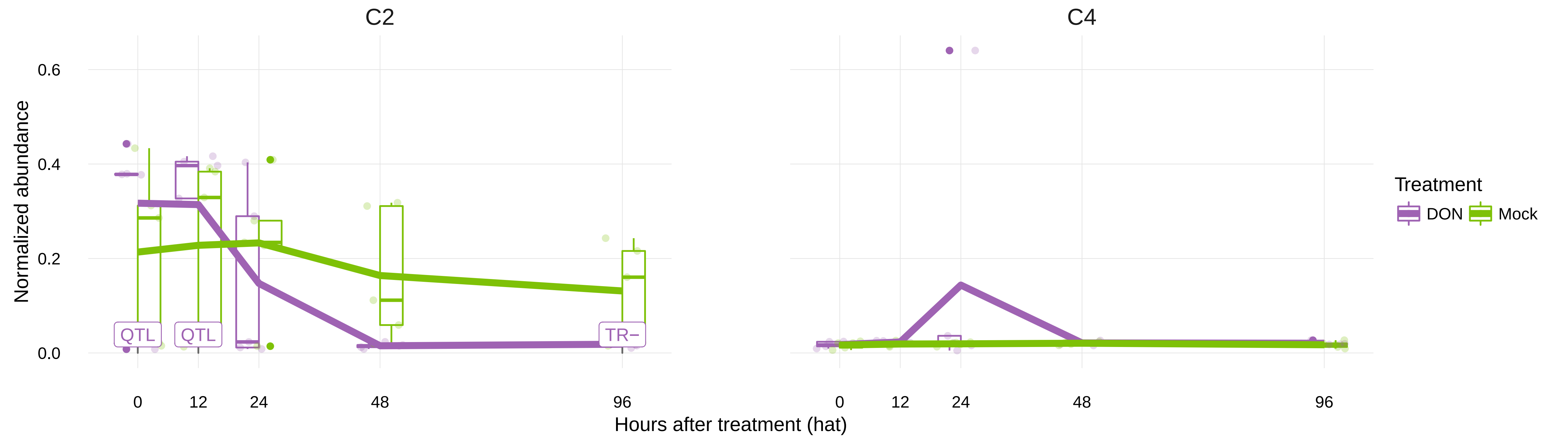

DON, Mock; different genotypes

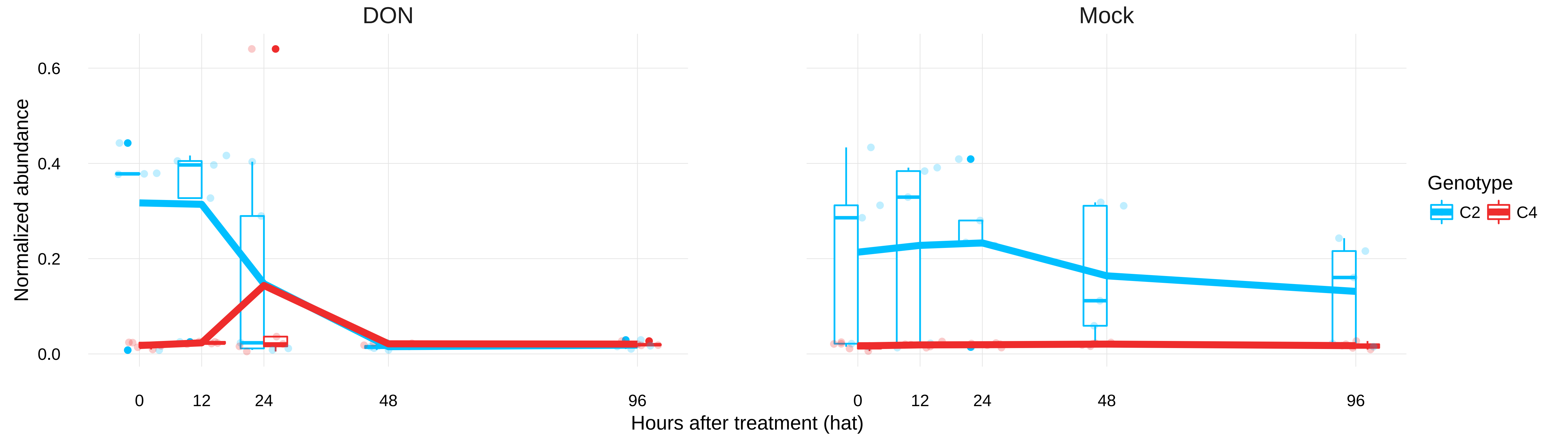

CM, Remus; different treatments

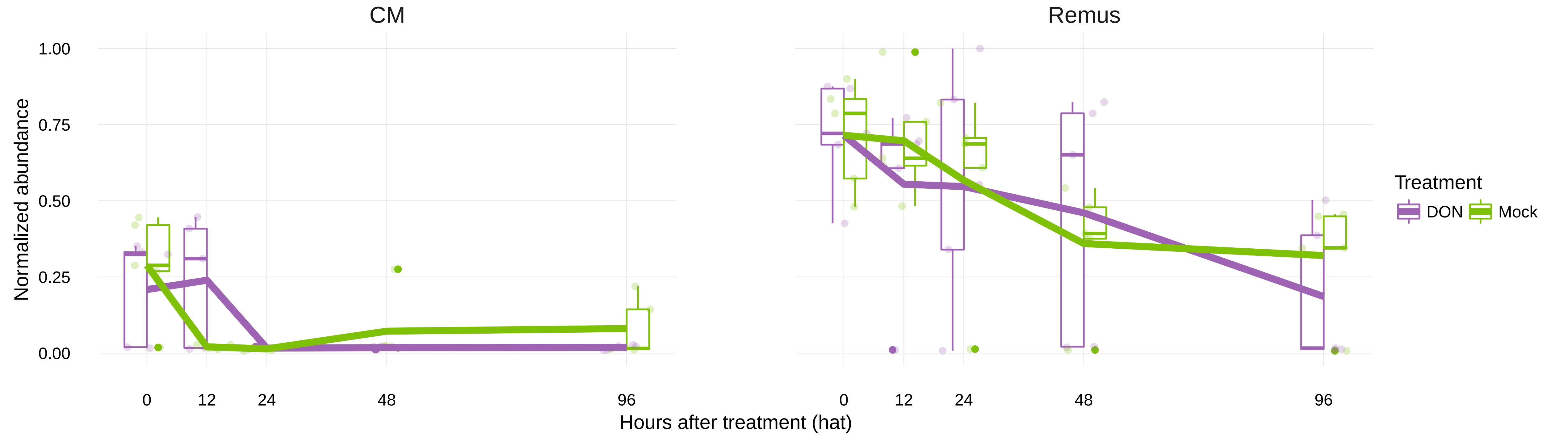

DON, Mock; all four genotypes

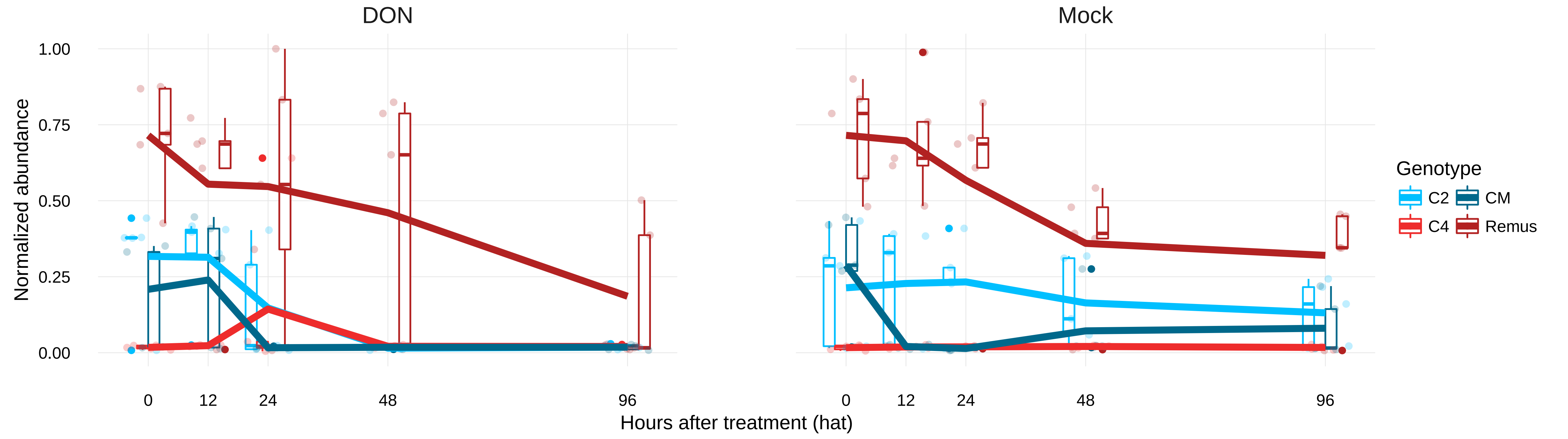

A.20

Annotated as HCA derivative
(1 database hit)

MZ	365.0845
RT	7.52 min
Normalization	Indirectly via surrogate in the KPX samples
Cluster	–
Cn total / Phe	15 /

C2, C4; different treatments

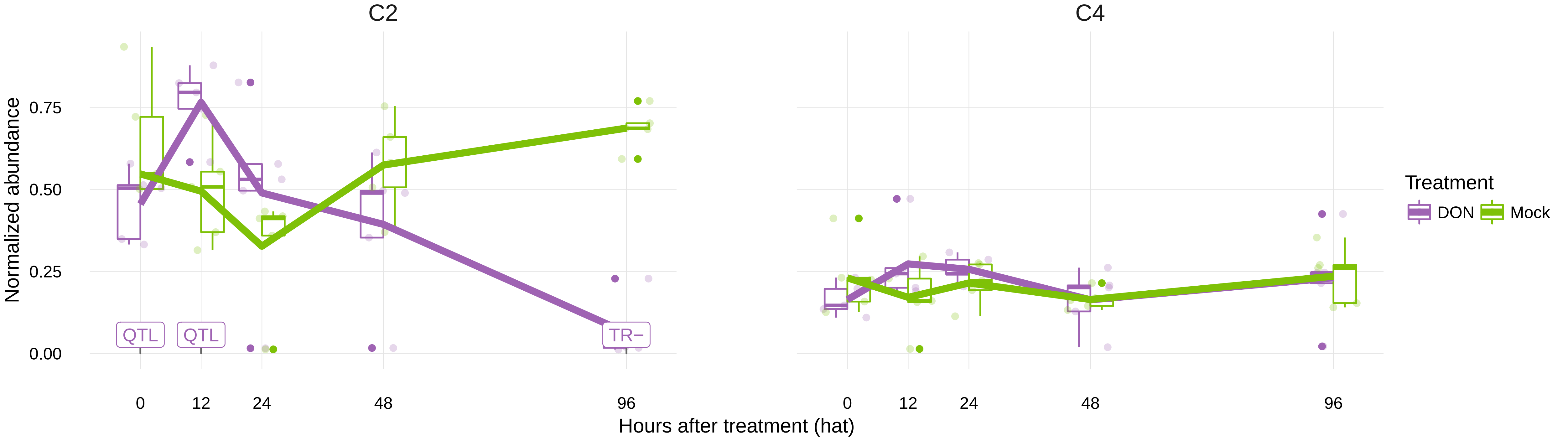

DON, Mock; different genotypes

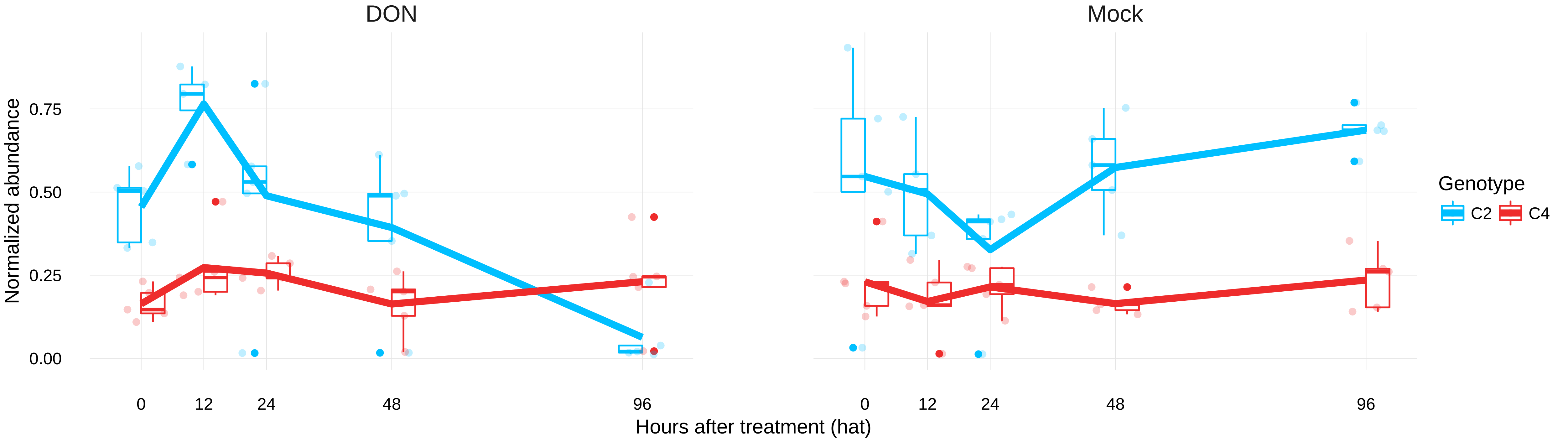

CM, Remus; different treatments

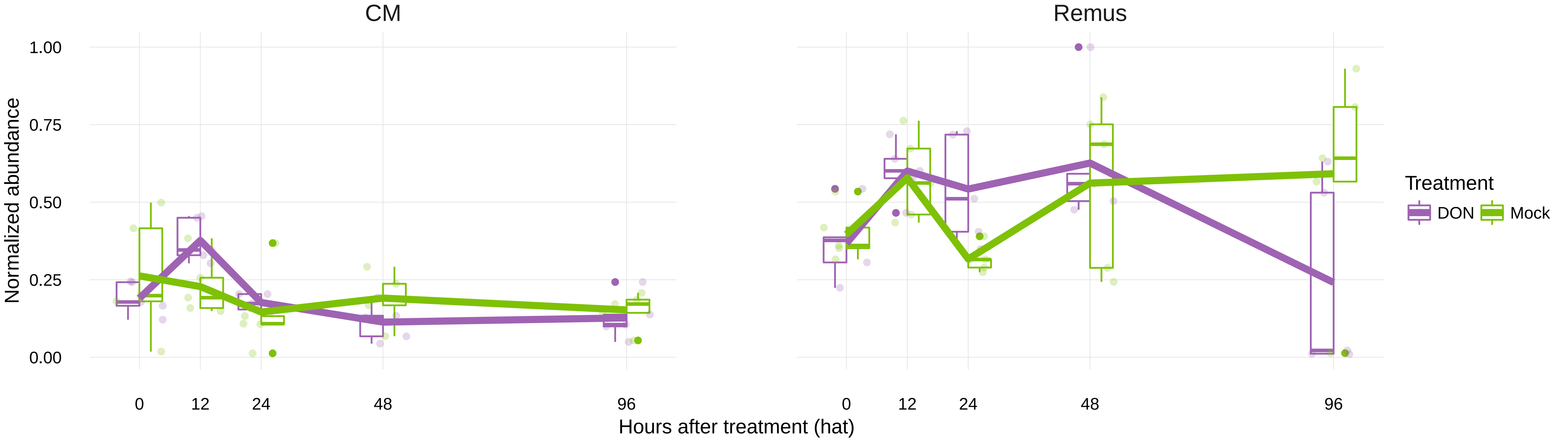

DON, Mock; all four genotypes

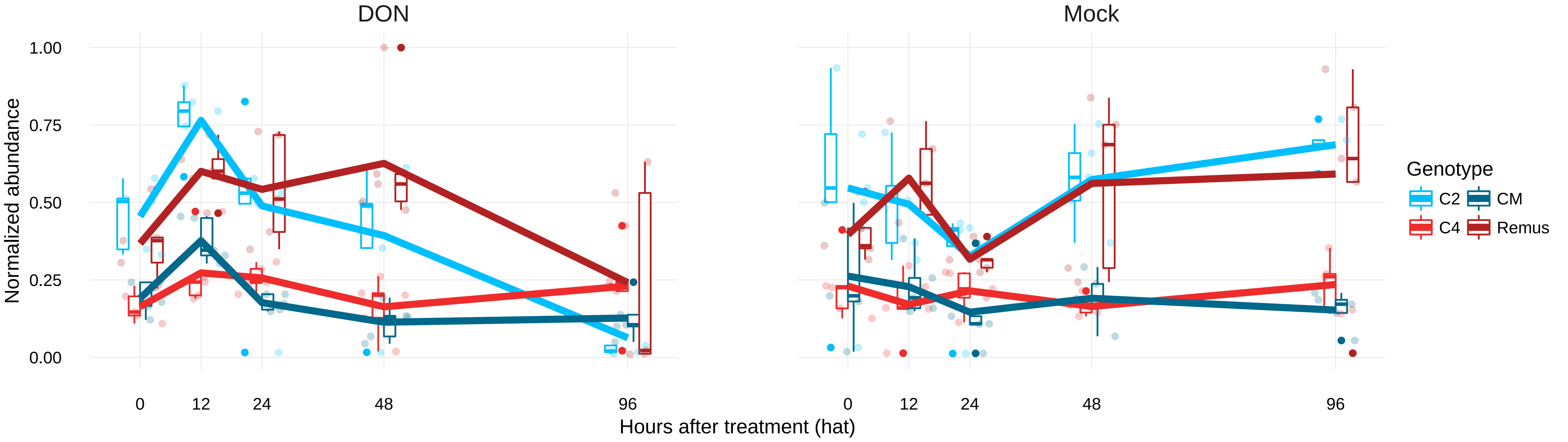

A.50

Annotated as Flavonoid or others (GlcF, Chalkon)
(3 database hits)

MZ	573.1578
RT	11.99 min
Normalization	Directly via KPX samples
Cluster	–
Cn total / Phe	26 /

C2, C4; different treatments

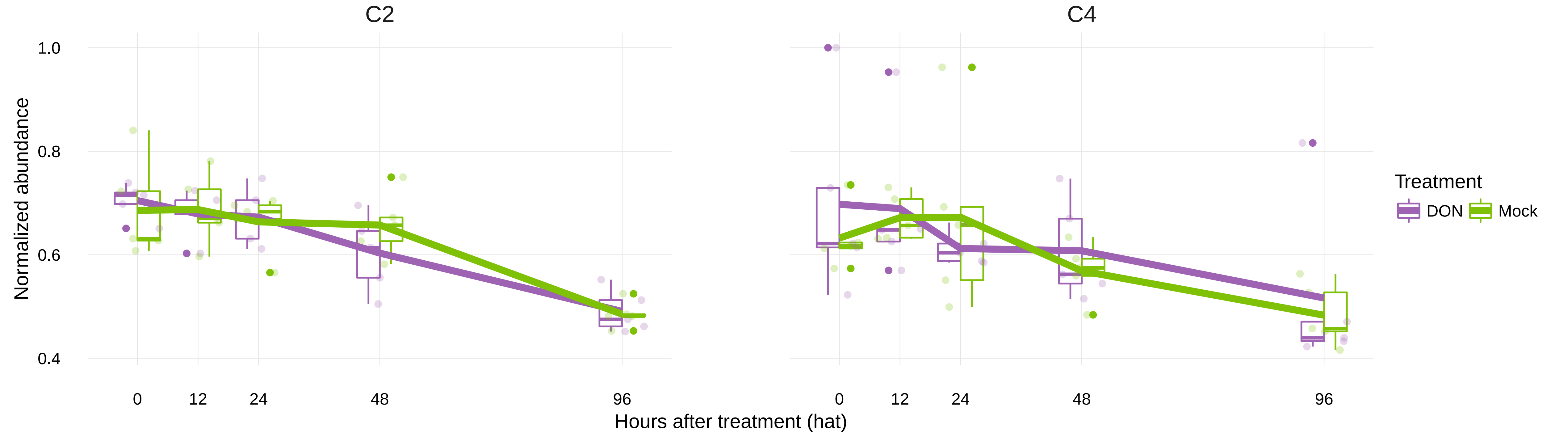

DON, Mock; different genotypes

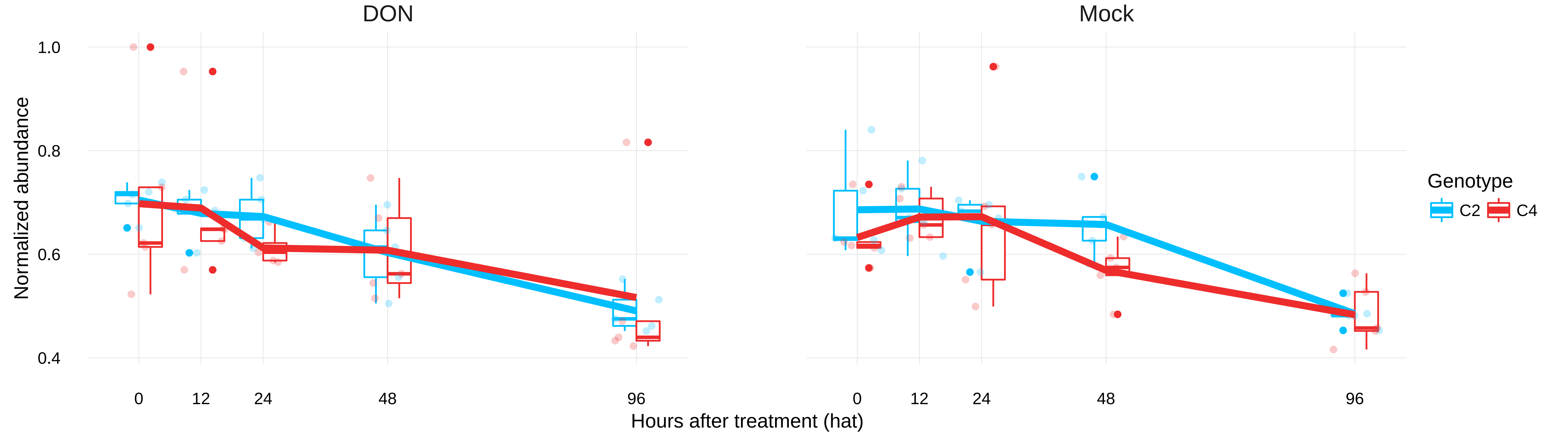

CM, Remus; different treatments

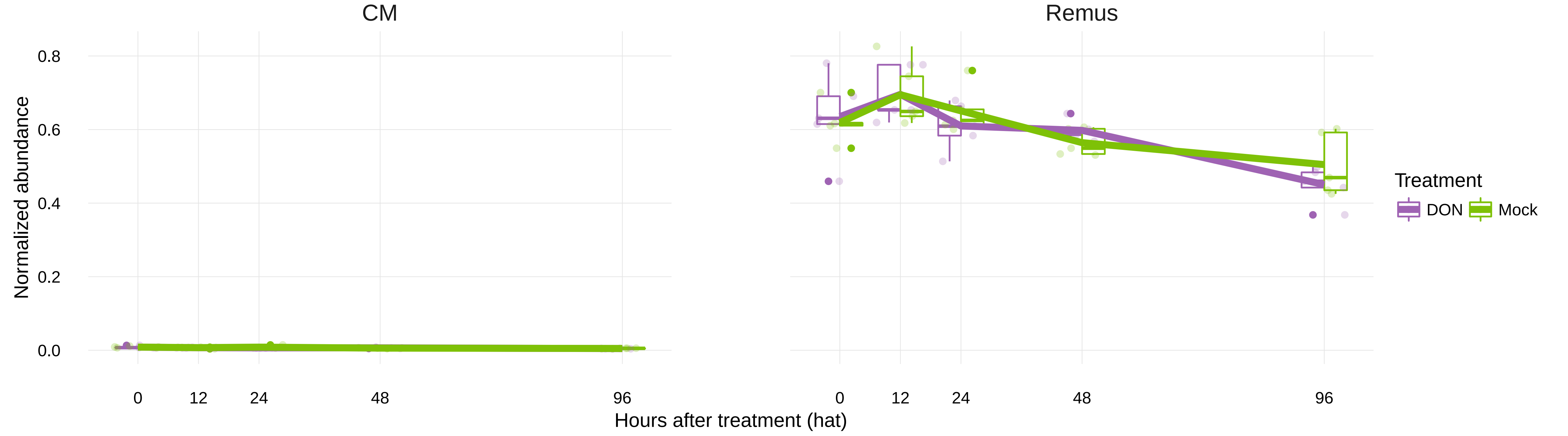

DON, Mock; all four genotypes

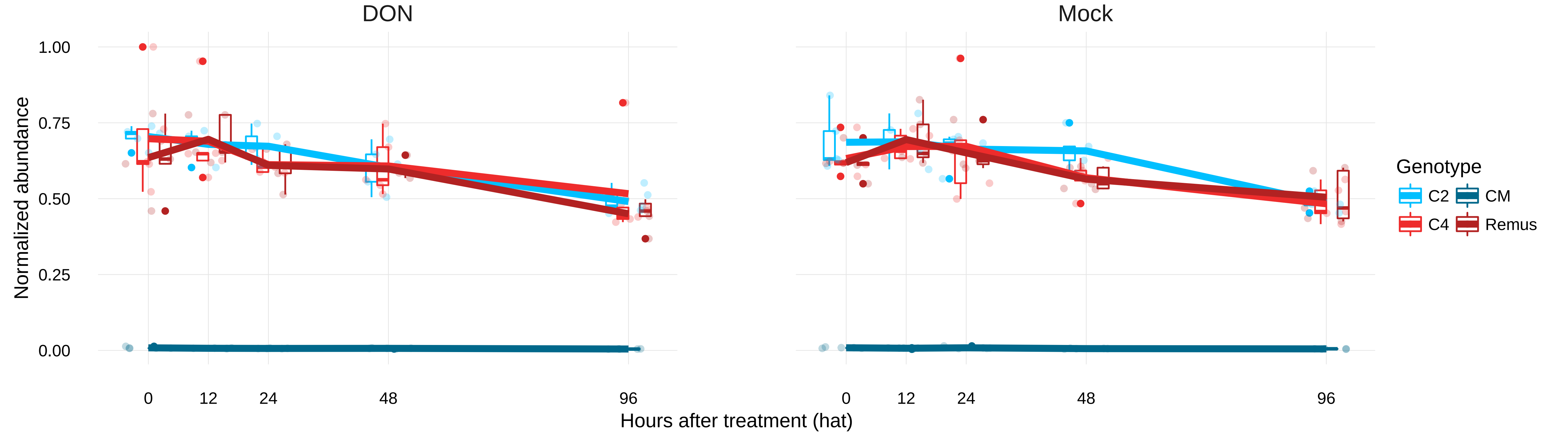

A.15

Annotated as others (Phenol)
(1 database hit)

MZ	127.039
RT	6.67 min
Normalization	Indirectly via surrogate in the KPX samples
Cluster	–
Cn total / Phe	6 /

C2, C4; different treatments

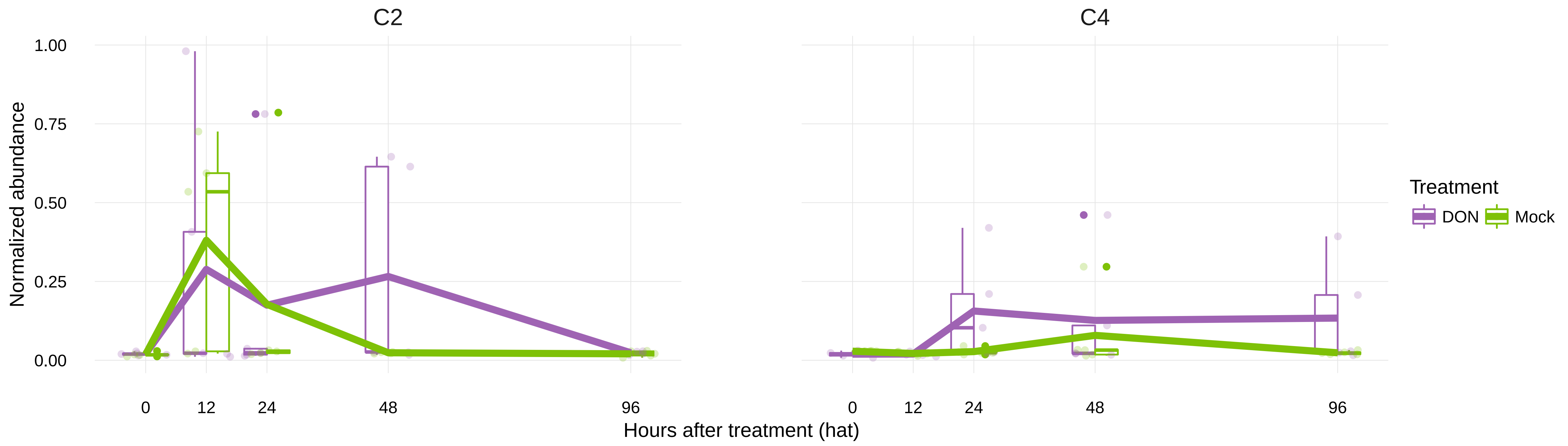

DON, Mock; different genotypes

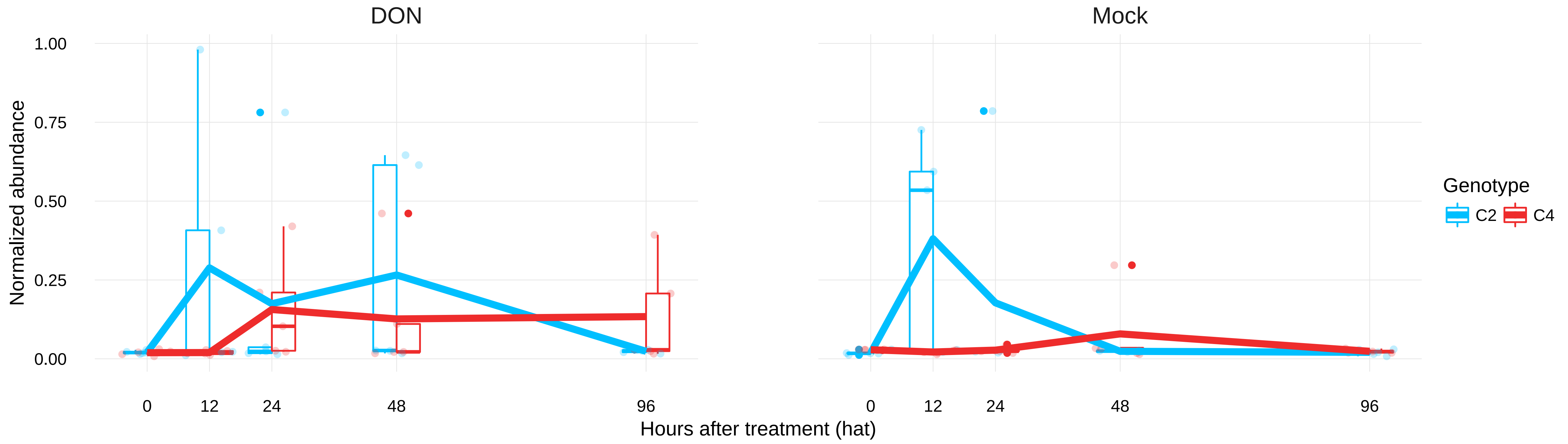

CM, Remus; different treatments

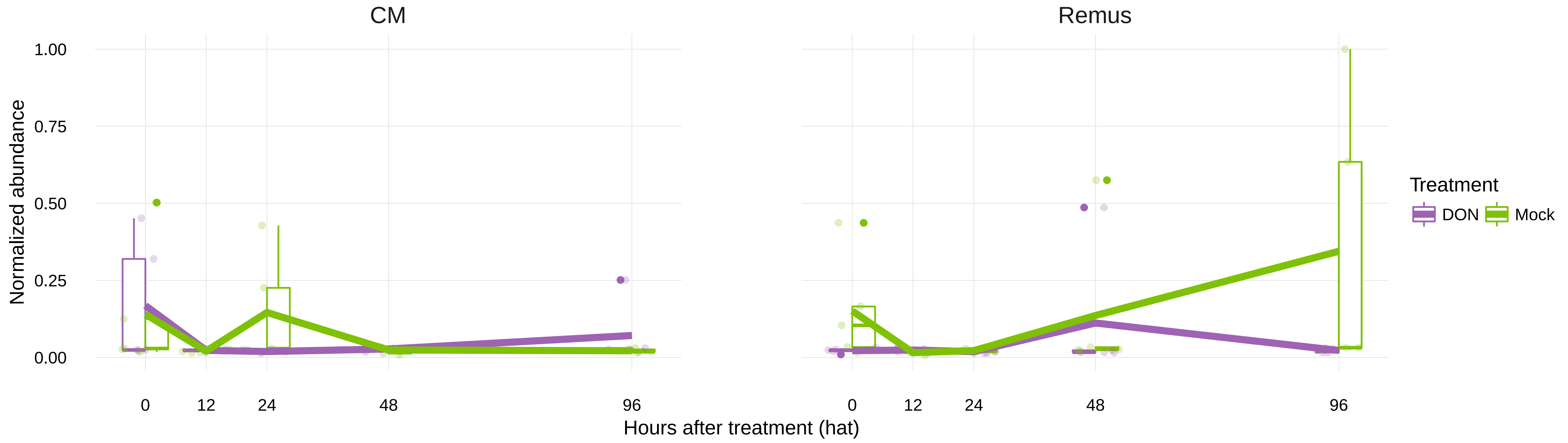

DON, Mock; all four genotypes

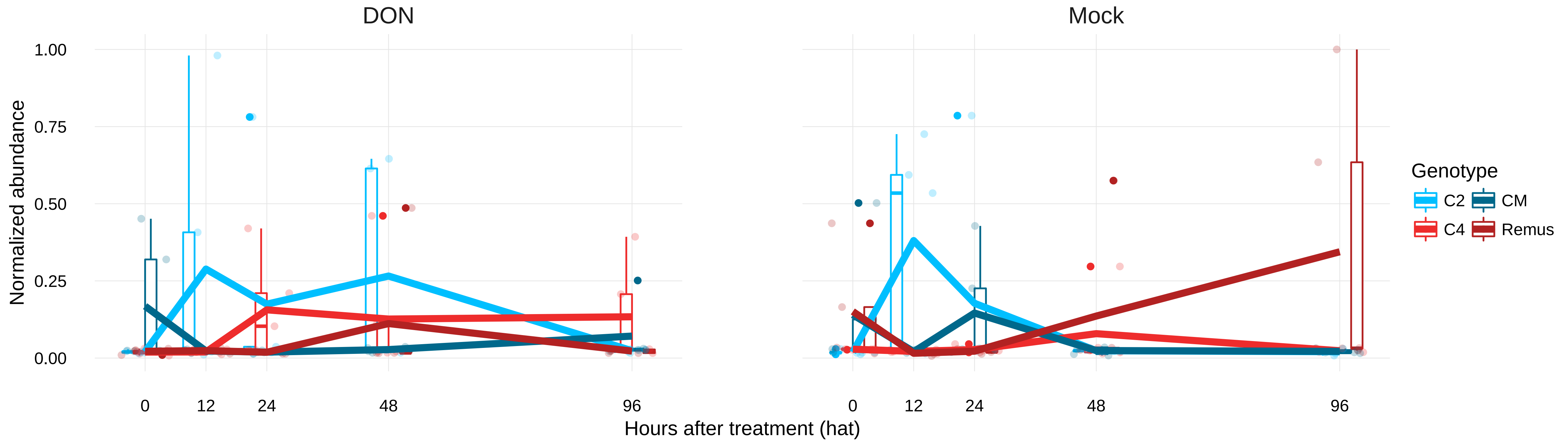

A.28

Annotated as HCA derivative
(1 database hit)

MZ	211.0965
RT	9.19 min
Normalization	Directly via KPX samples
Cluster	–
Cn total / Phe	10 /

C2, C4; different treatments

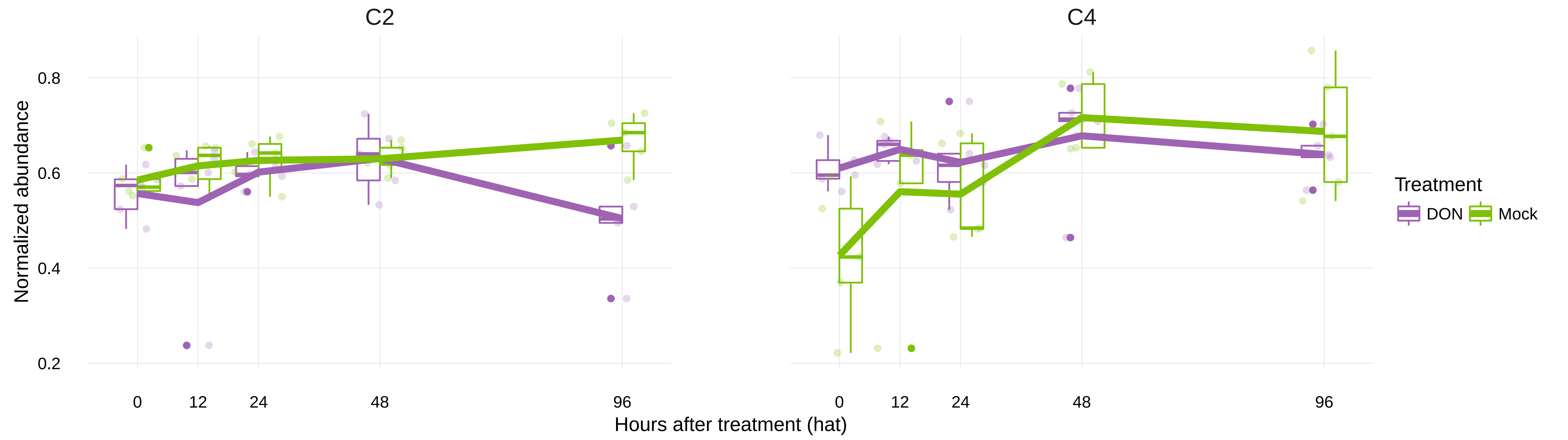

DON, Mock; different genotypes

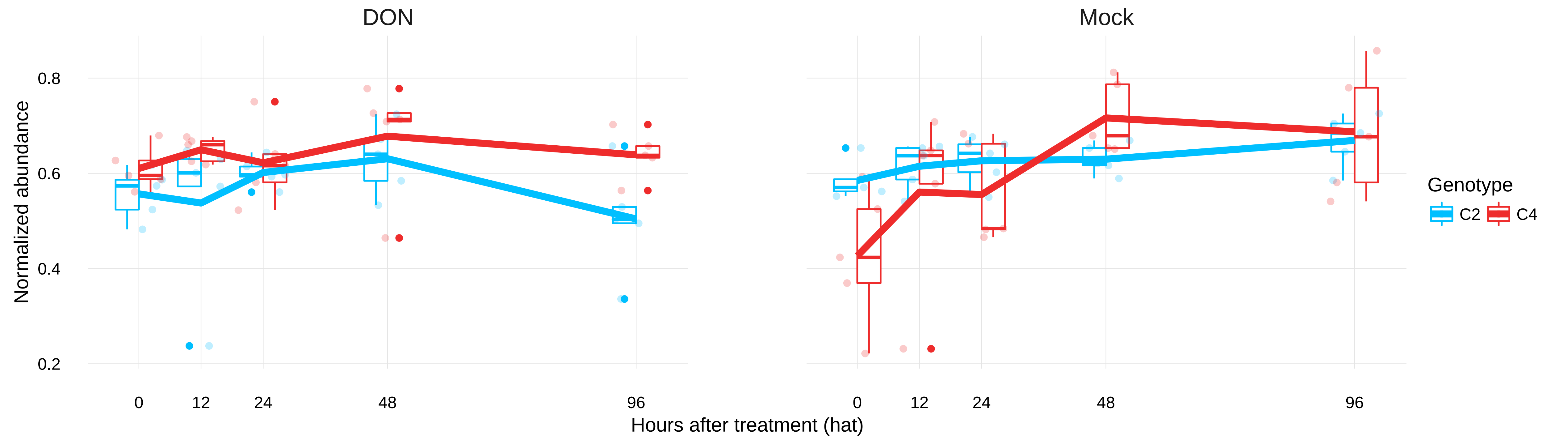

CM, Remus; different treatments

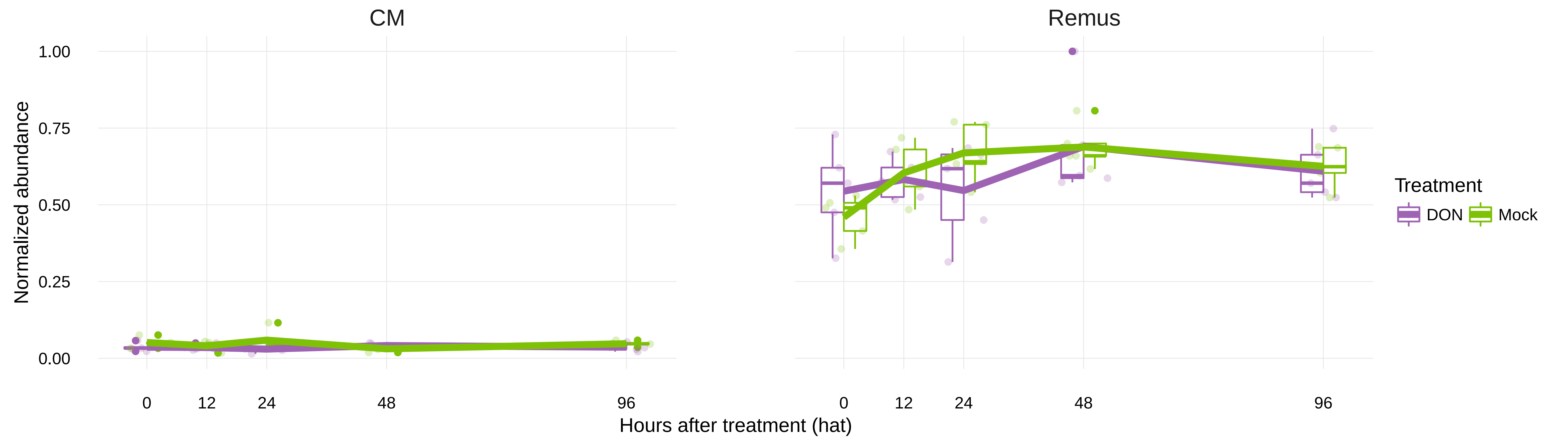

DON, Mock; all four genotypes

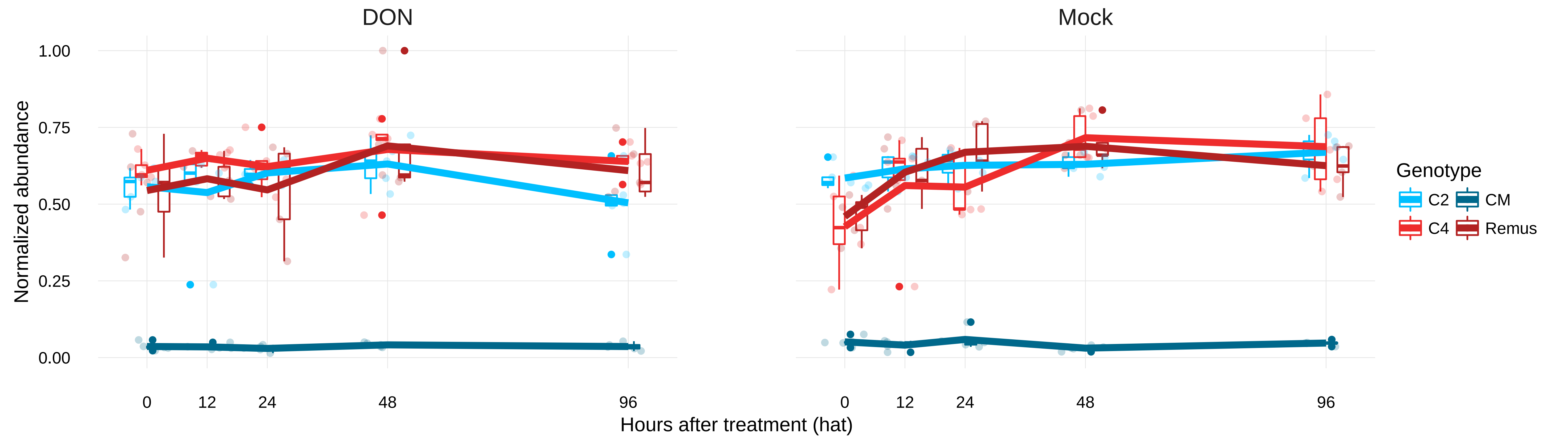

Vanillin

Identified metabolite (level 1)

MZ	153.0546
RT	11.63 min
Normalization	Indirectly via surrogate in the KPX samples
Cluster	–
Cn total / Phe	8 / 7

C2, C4; different treatments

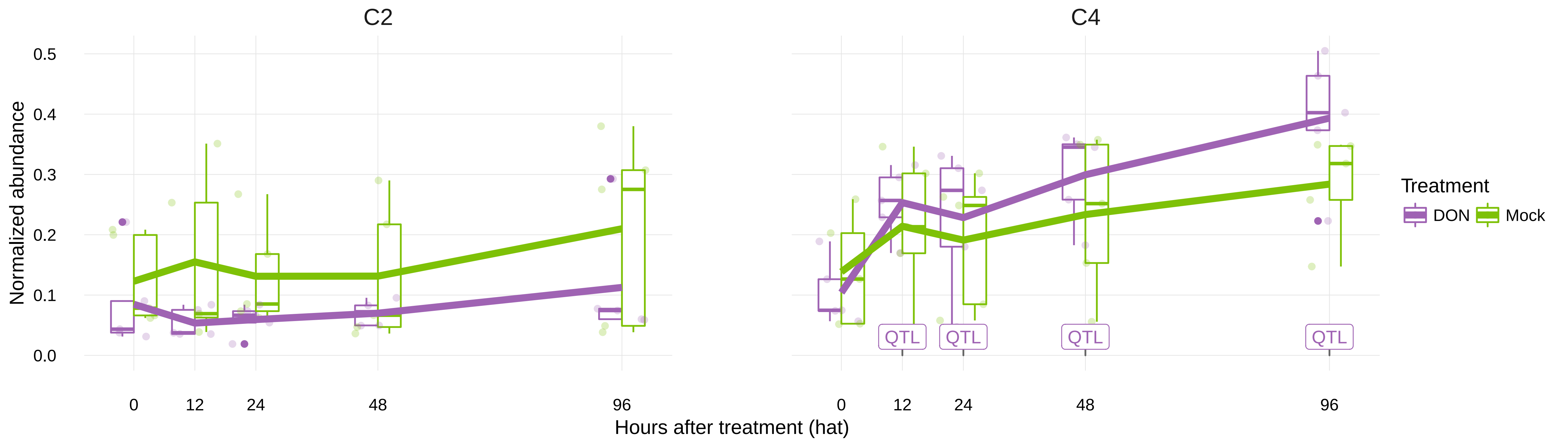

DON, Mock; different genotypes

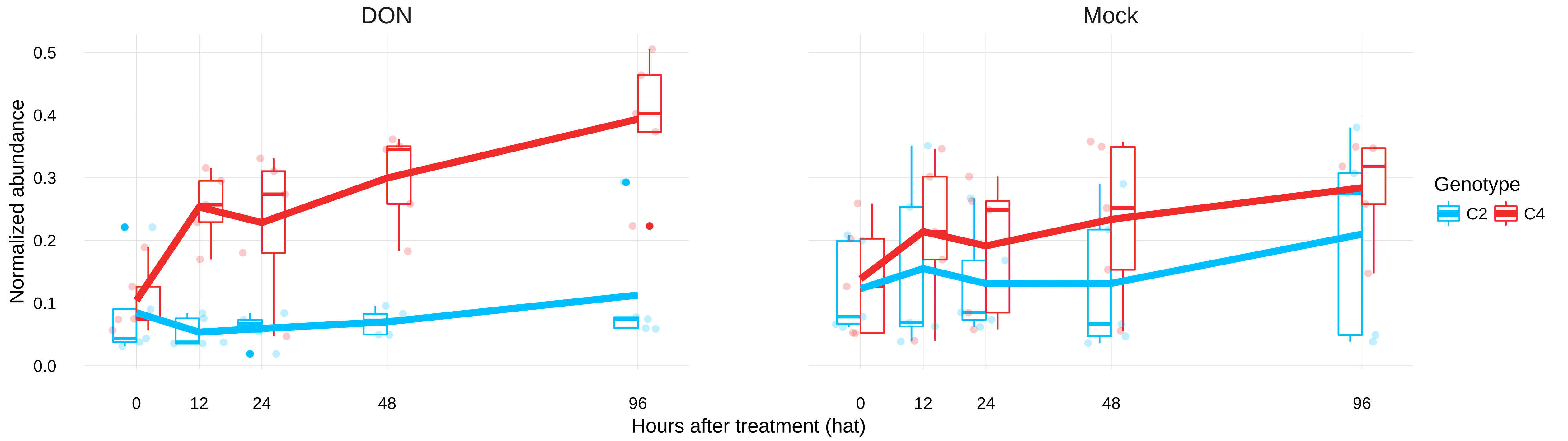

CM, Remus; different treatments

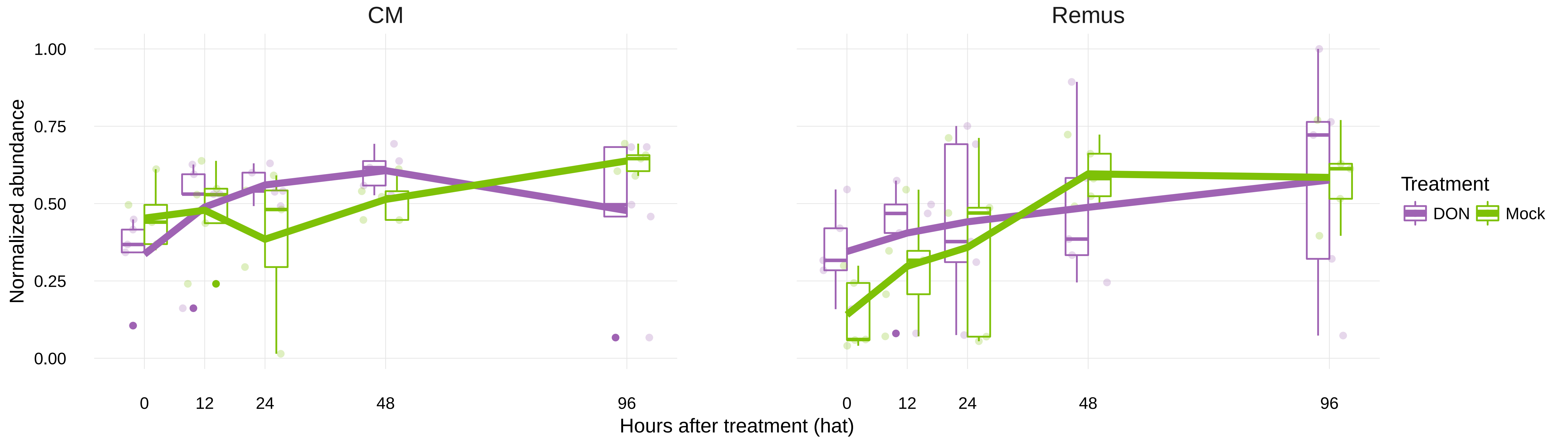

DON, Mock; all four genotypes

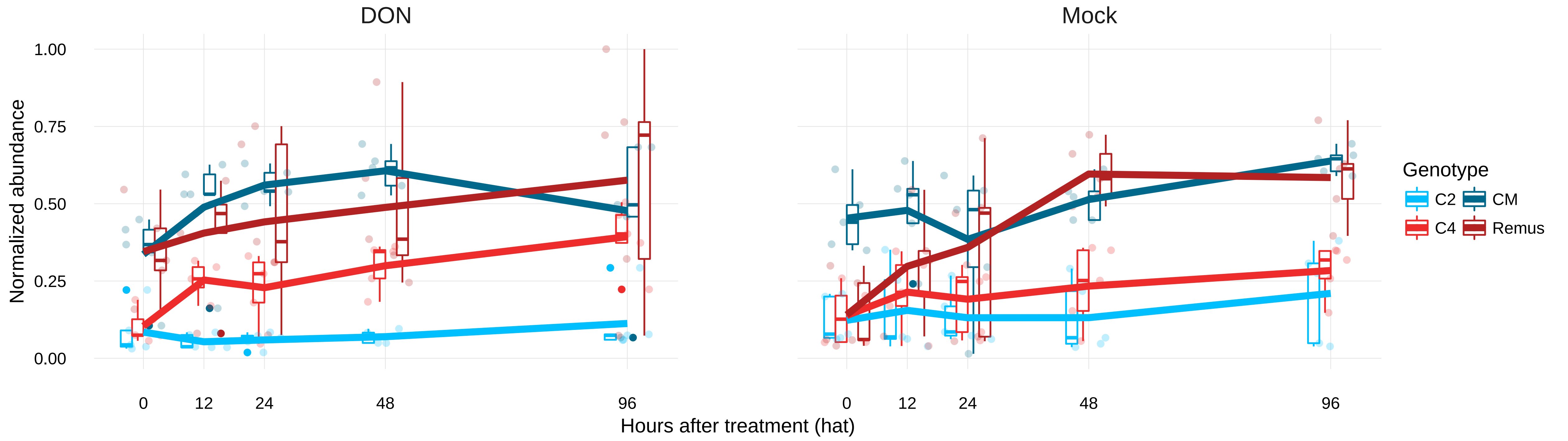

A.37

Annotated as HCA derivative

(1 database hit)

MZ	536.1974
RT	10.28 min
Normalization	Indirectly via surrogate in the KPX samples
Cluster	–
Cn total / Phe	22 / 9

C2, C4; different treatments

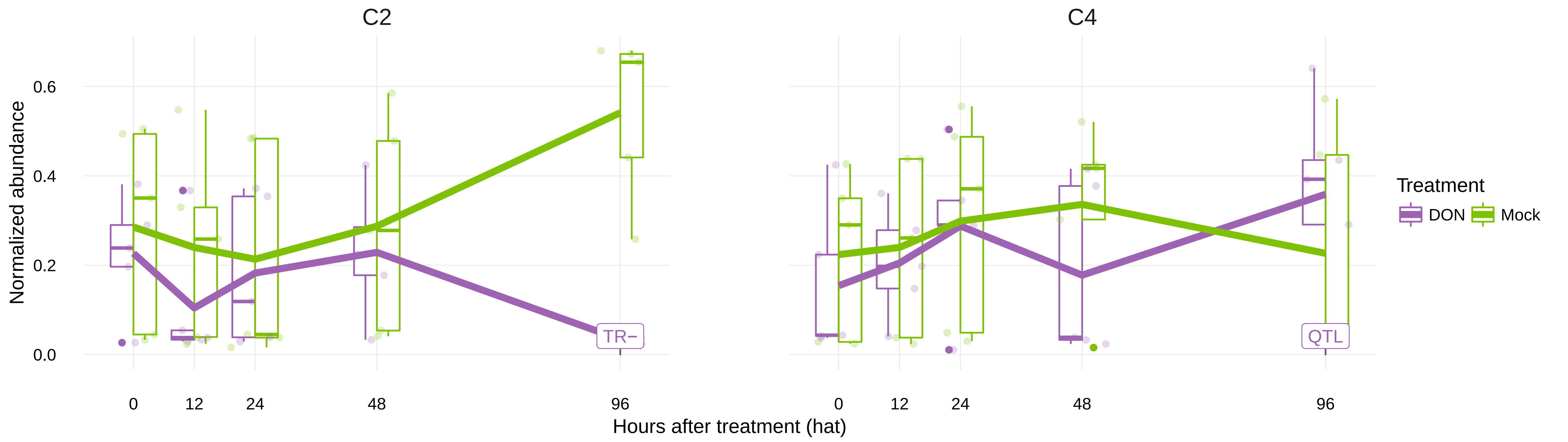

DON, Mock; different genotypes

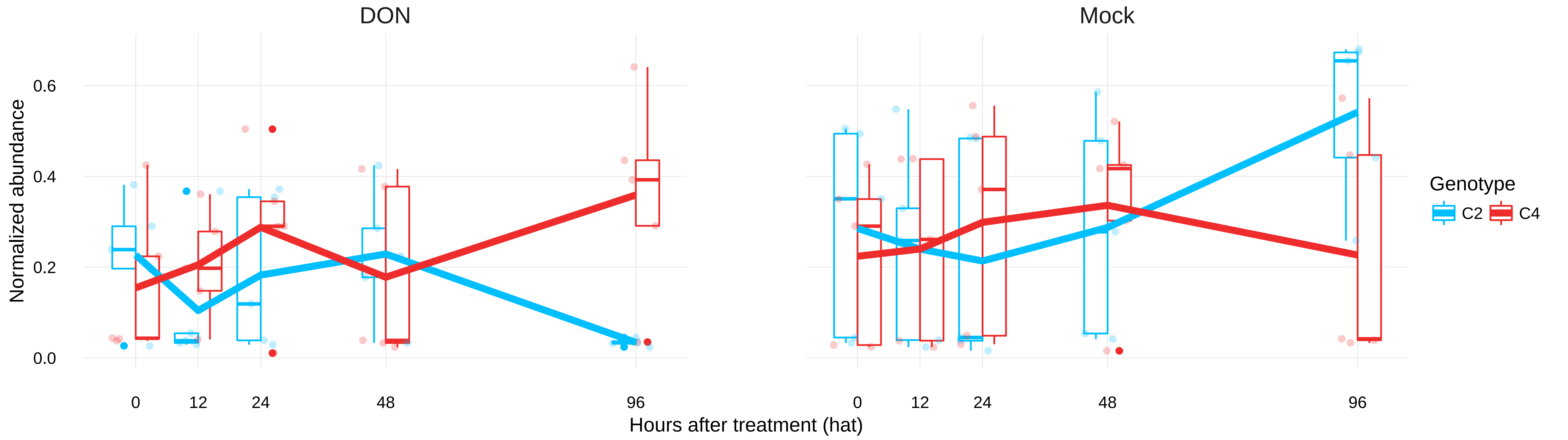

CM, Remus; different treatments

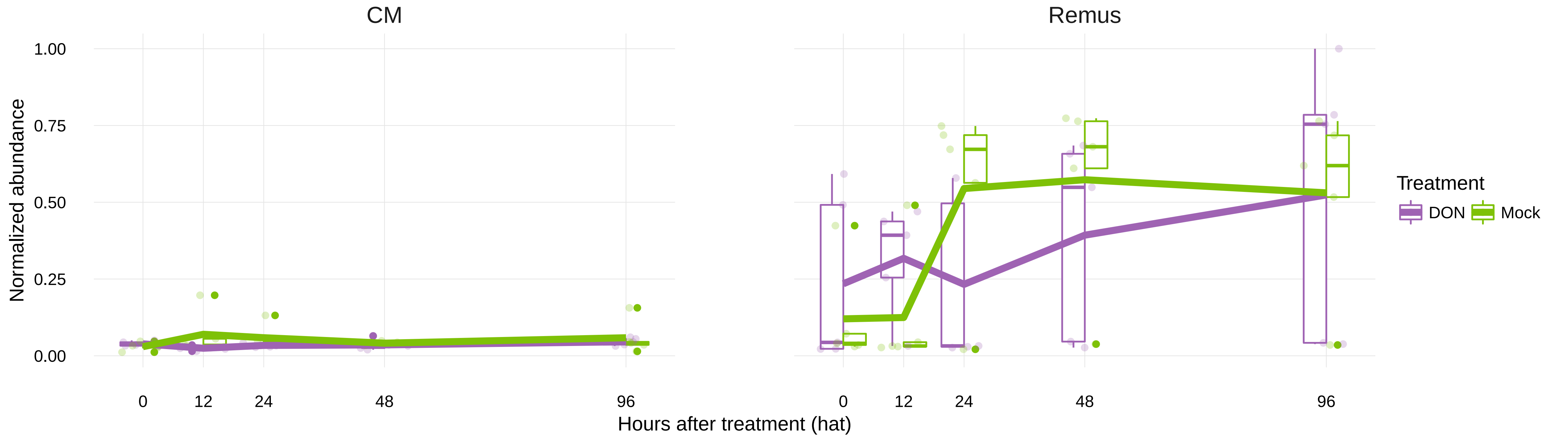

DON, Mock; all four genotypes

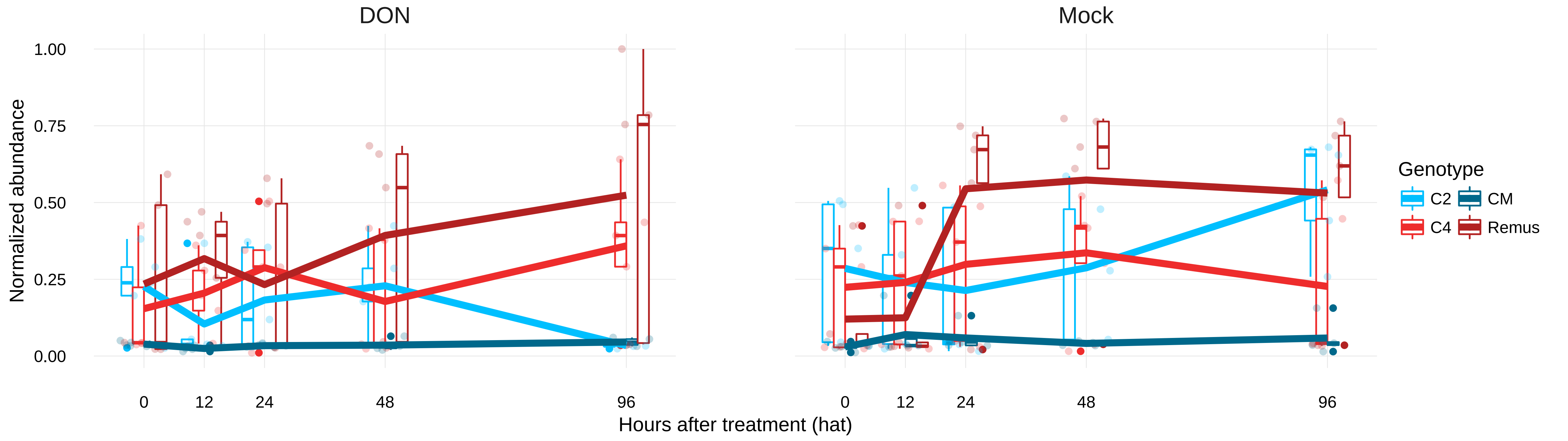

U.18

Unknown Phe−derived wheat−metabolite

MZ	335.1674
RT	34.17 min
Normalization	Directly via KPX samples
Cluster	−
Cn total / Phe	16 / 16

C2, C4; different treatments

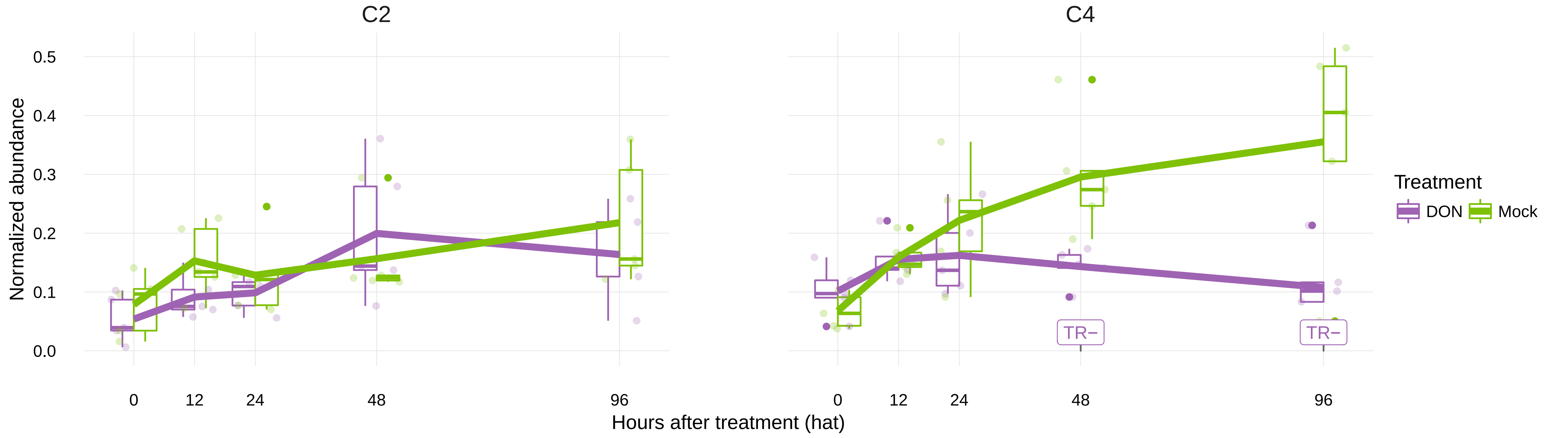

DON, Mock; different genotypes

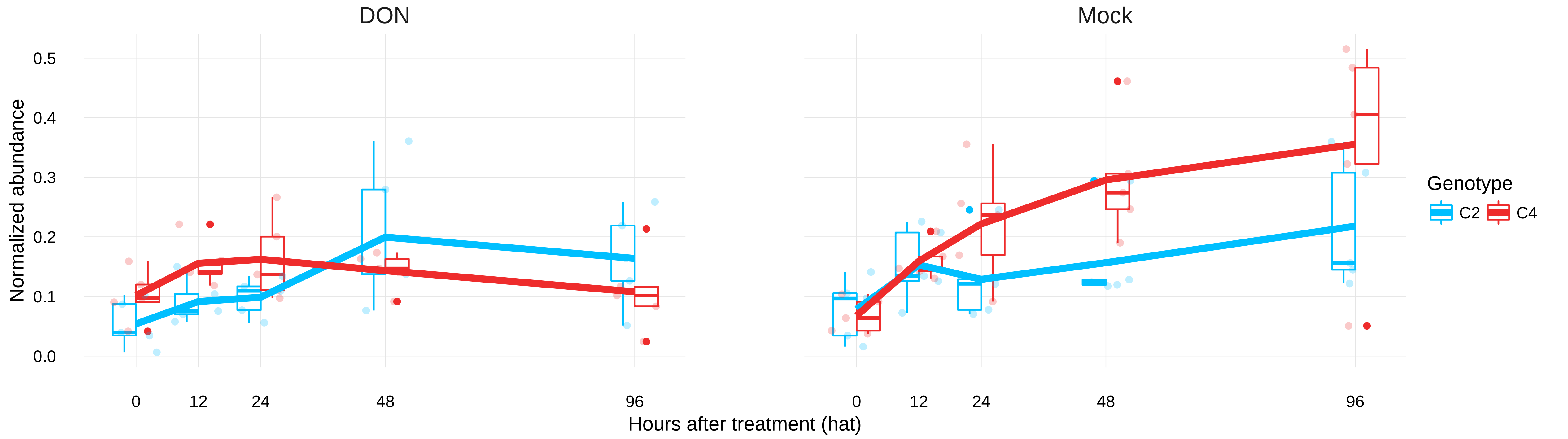

CM, Remus; different treatments

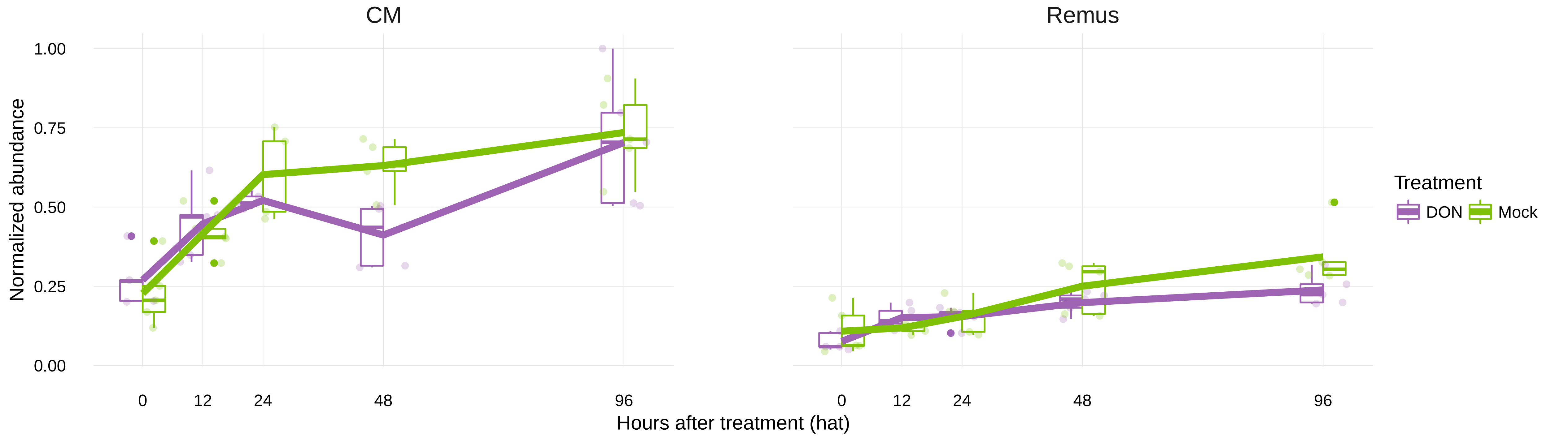

DON, Mock; all four genotypes

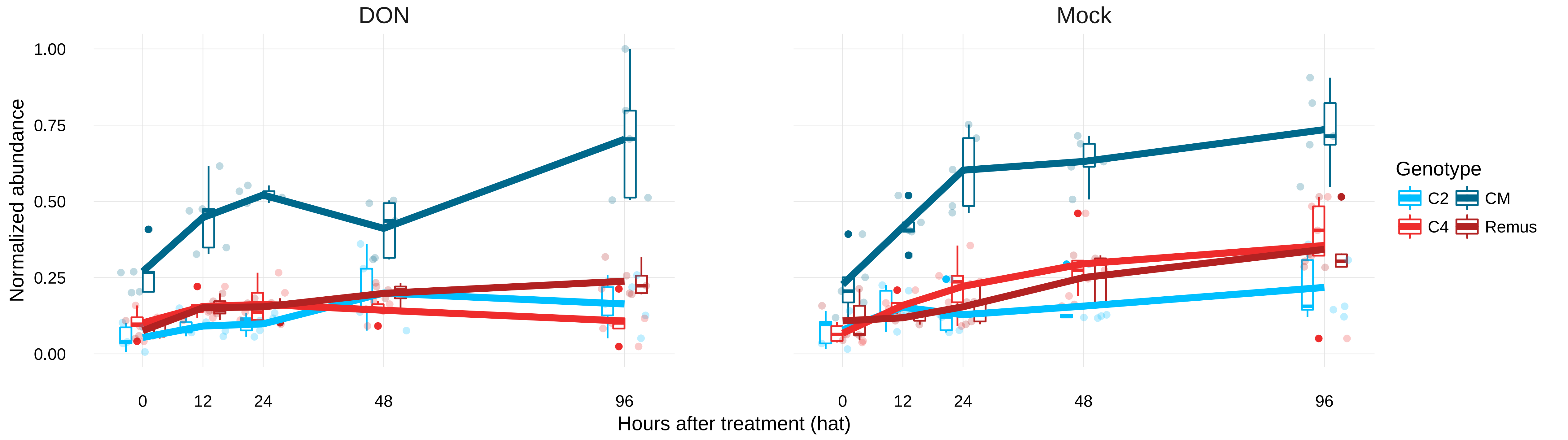

A.141

Annotated as HCA derivative
(1 database hit)

MZ	317.1726
RT	27.78 min
Normalization	Directly via KPX samples
Cluster	–
Cn total / Phe	17 /

C2, C4; different treatments

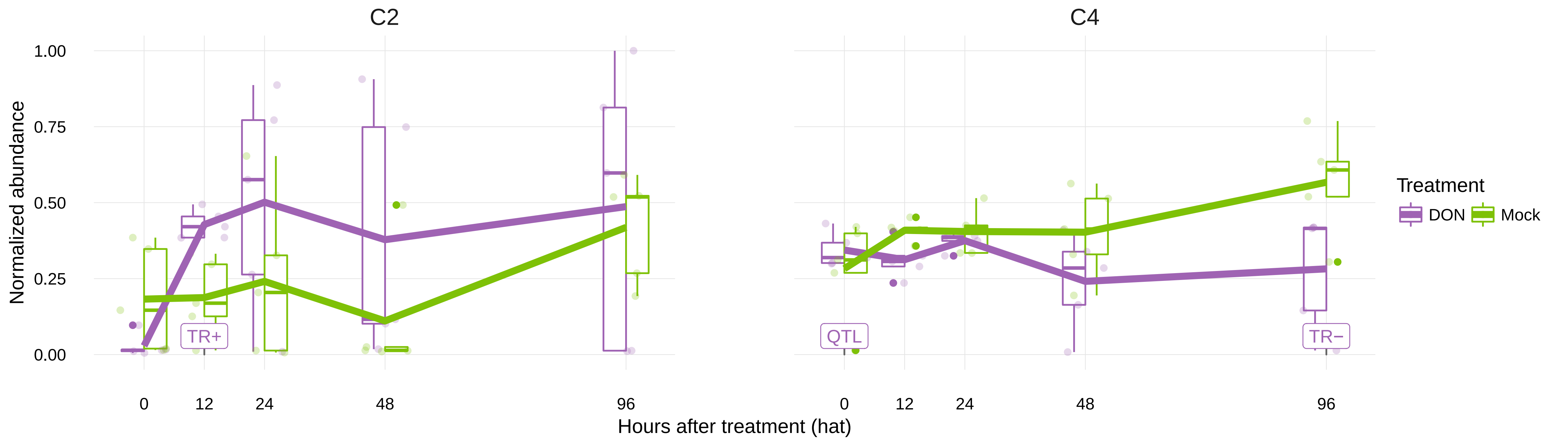

DON, Mock; different genotypes

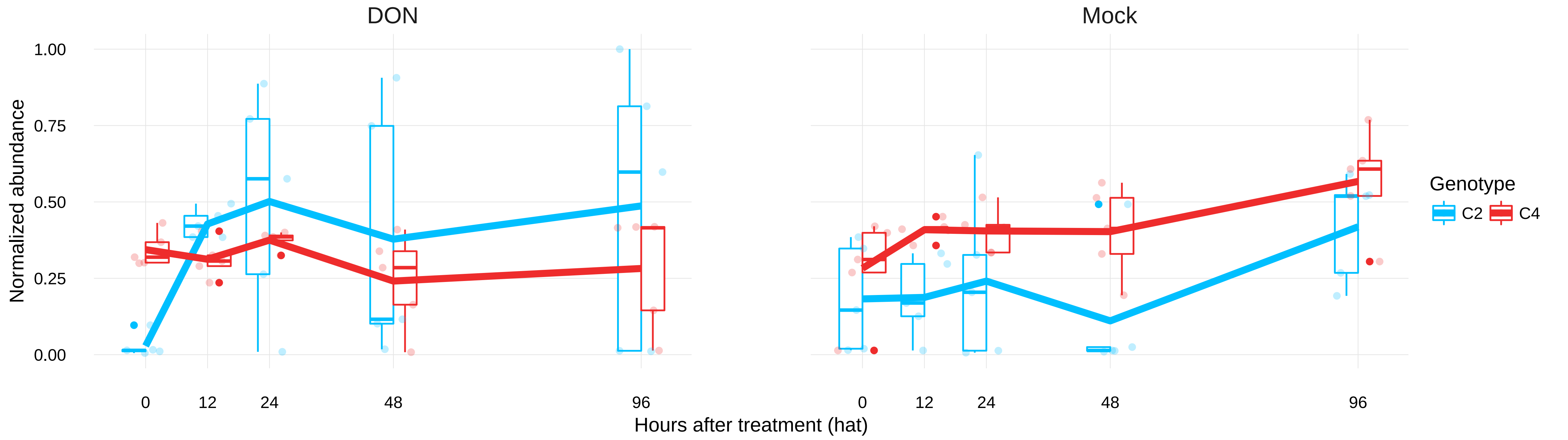

CM, Remus; different treatments

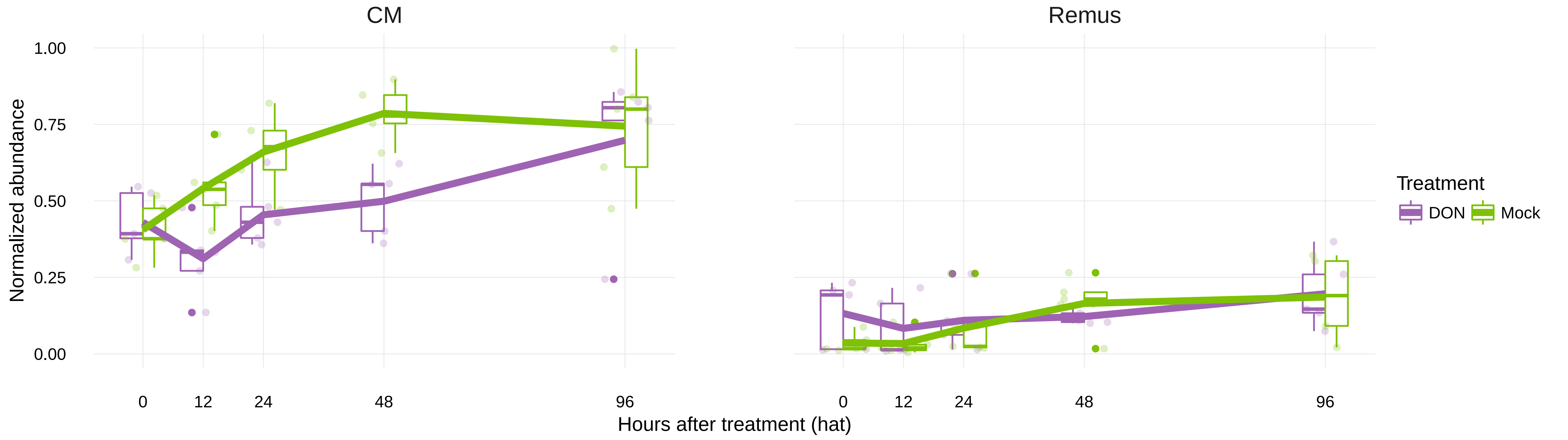

DON, Mock; all four genotypes

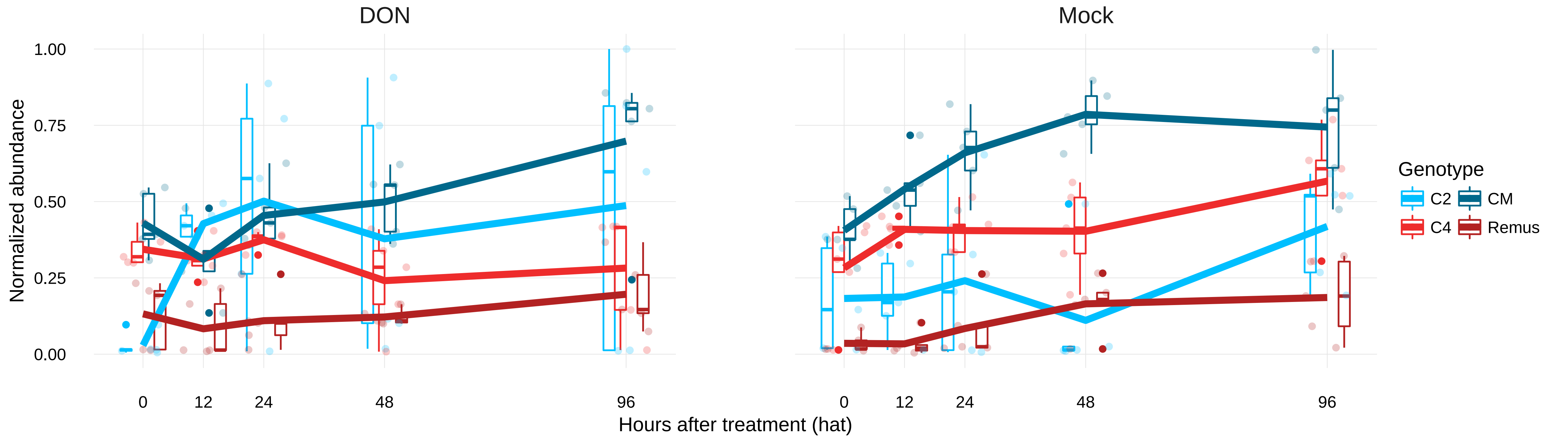

U.17

Unknown Phe−derived wheat−metabolite

MZ	541.3291
RT	33.09 min
Normalization	Not normalized
Cluster	−
Cn total / Phe	8 / 8

C2, C4; different treatments

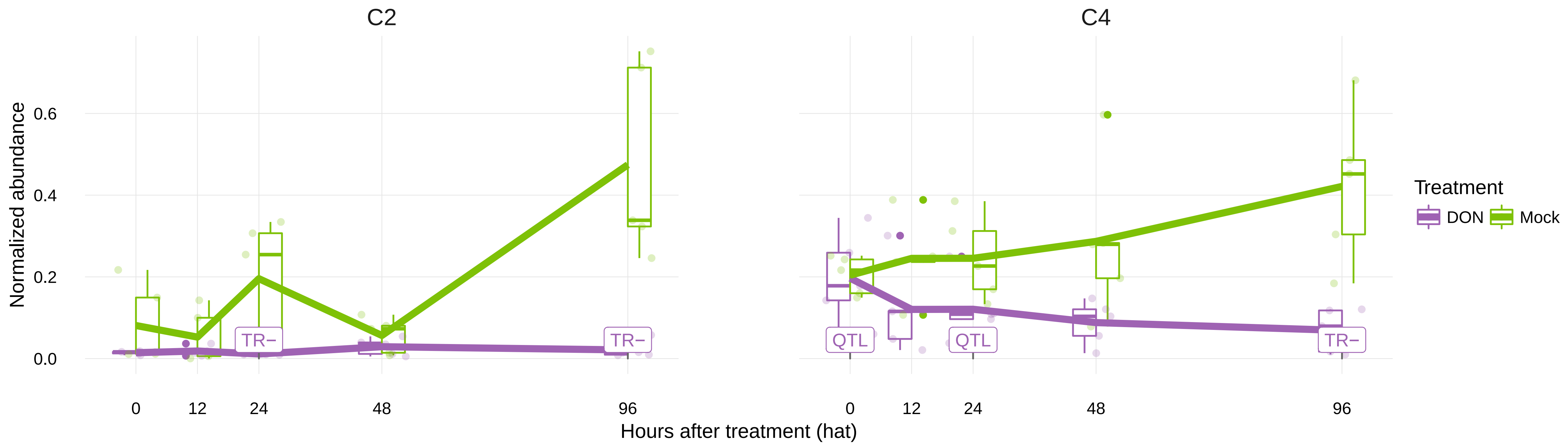

DON, Mock; different genotypes

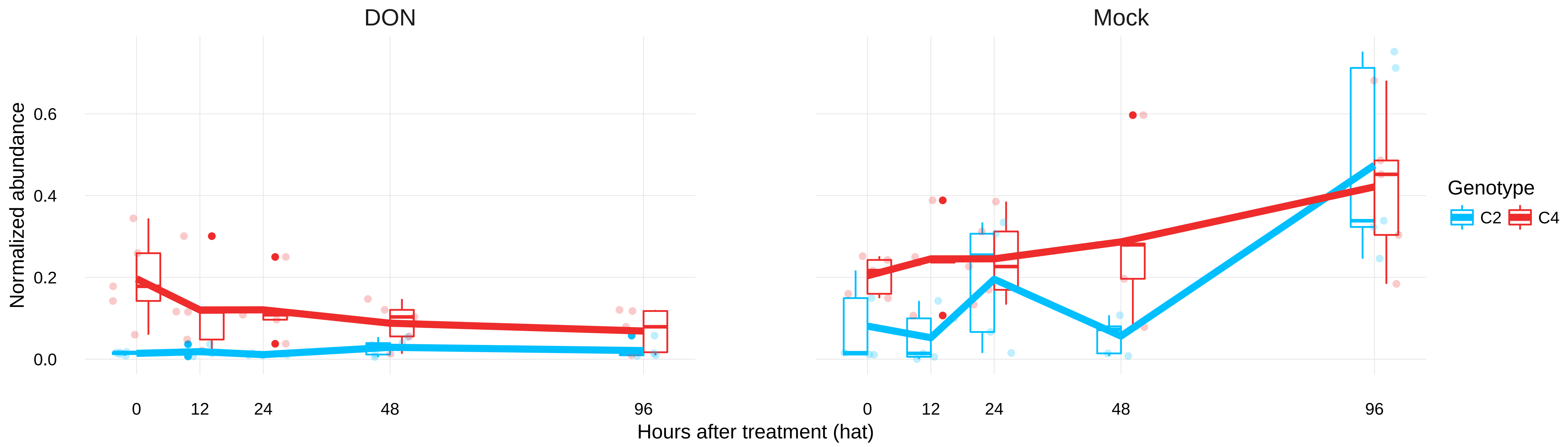

CM, Remus; different treatments

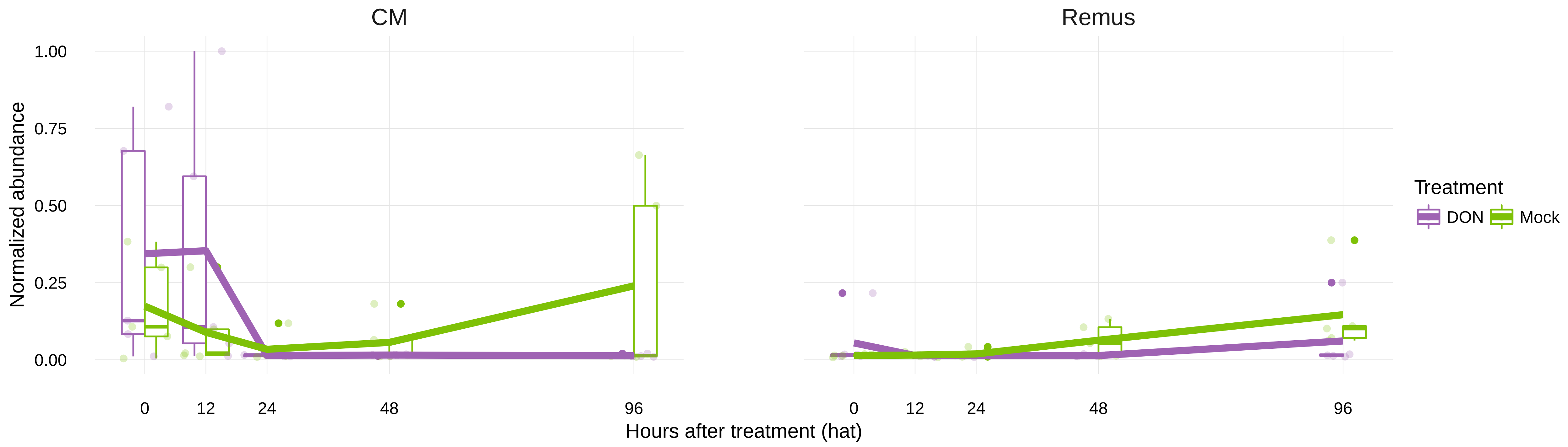

DON, Mock; all four genotypes

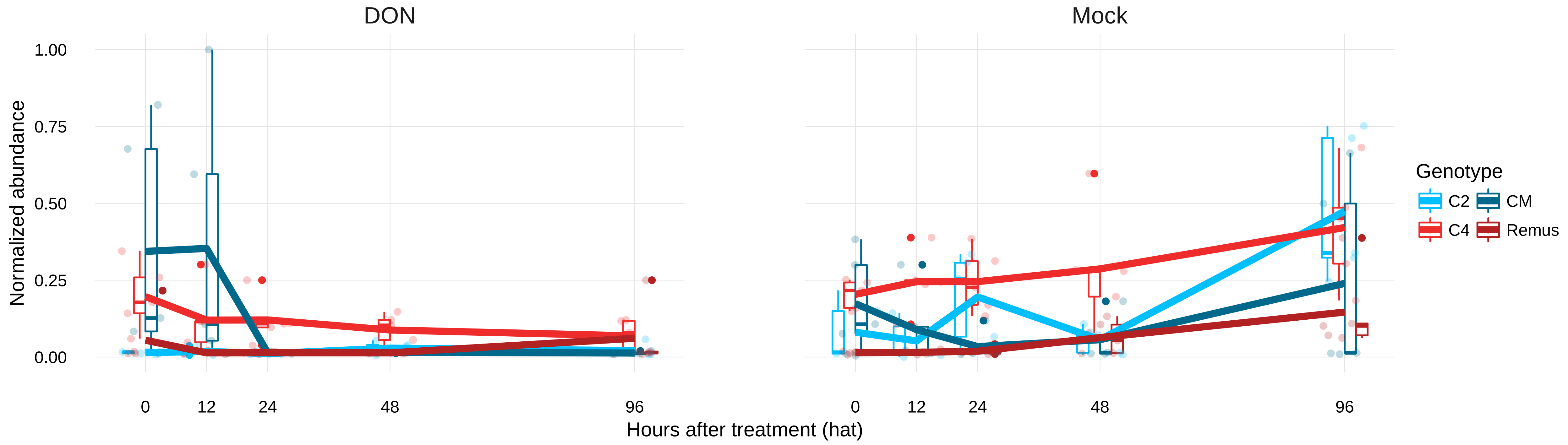

U.16

Unknown Phe−derived wheat−metabolite

MZ	798.9801
RT	31.92 min
Normalization	Not normalized
Cluster	−
Cn total / Phe	6 / 6

C2, C4; different treatments

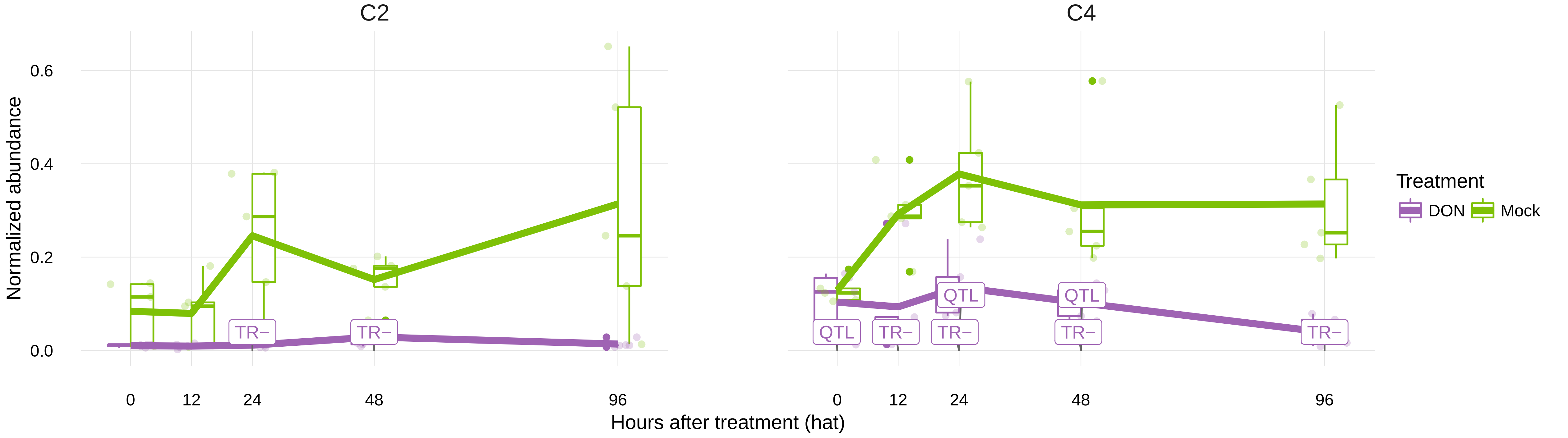

DON, Mock; different genotypes

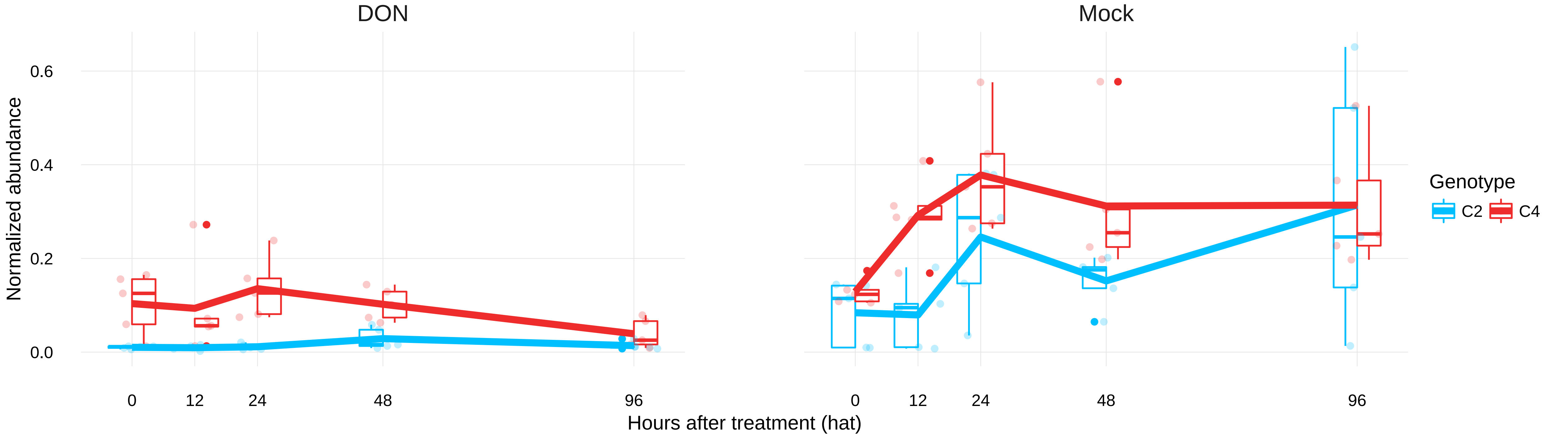

CM, Remus; different treatments

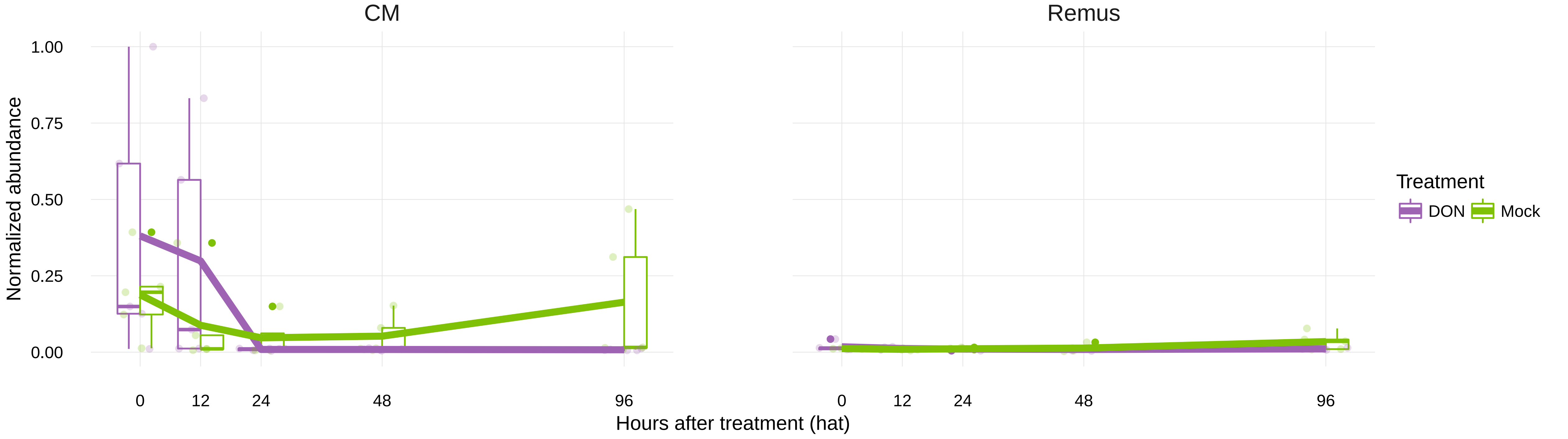

DON, Mock; all four genotypes

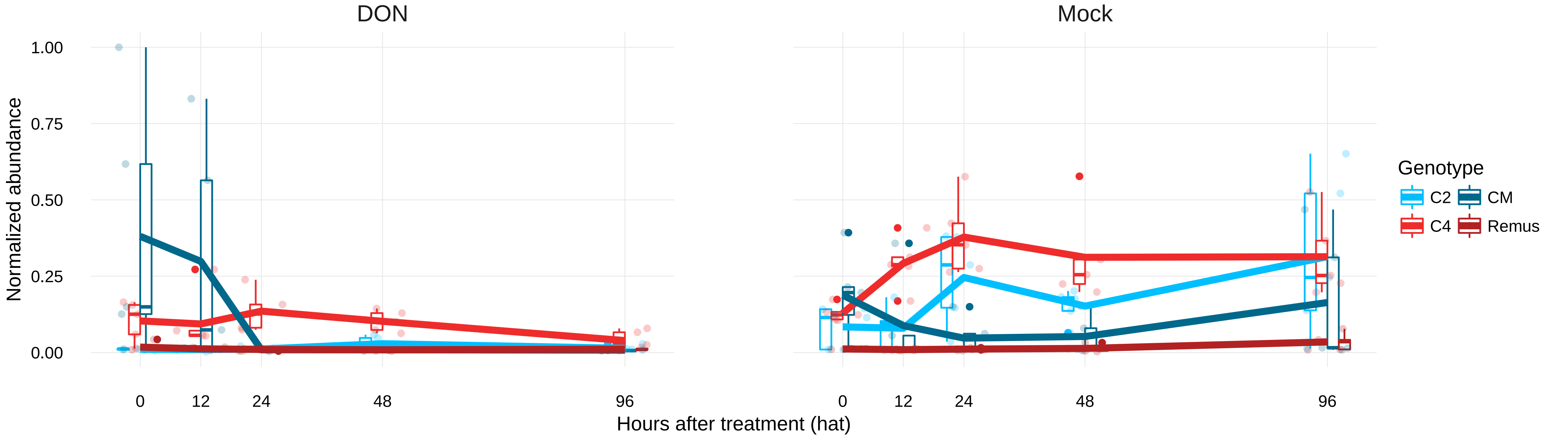

A.9

Annotated as Putative phenylacetic acid derivative
(1 database hit)

MZ	105.0698
RT	5.43 min
Normalization	Not normalized
Cluster	–
Cn total / Phe	8 / 8

C2, C4; different treatments

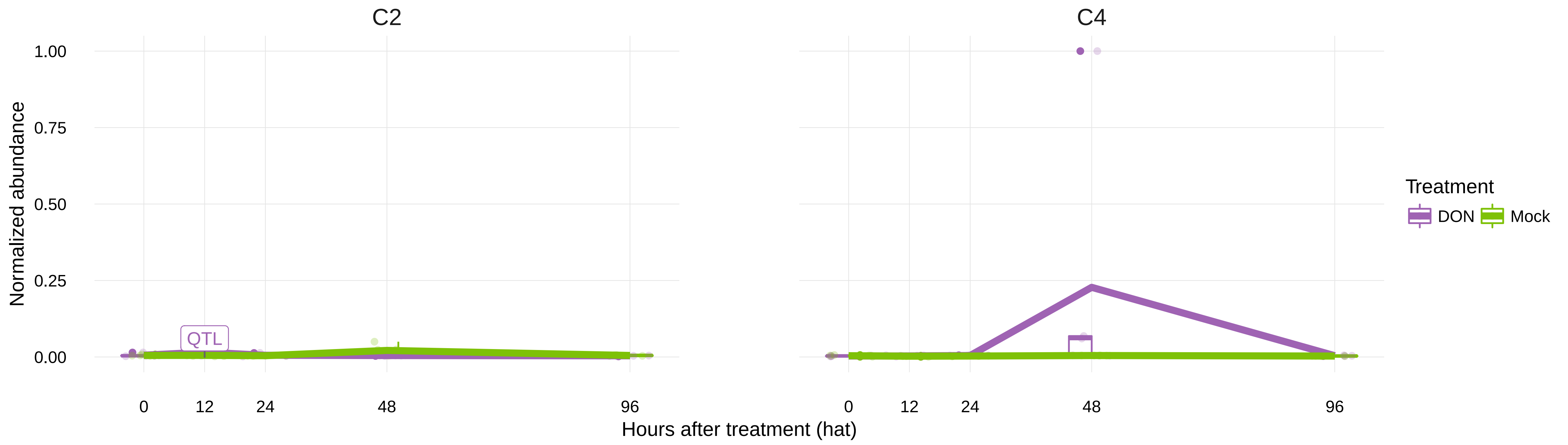

DON, Mock; different genotypes

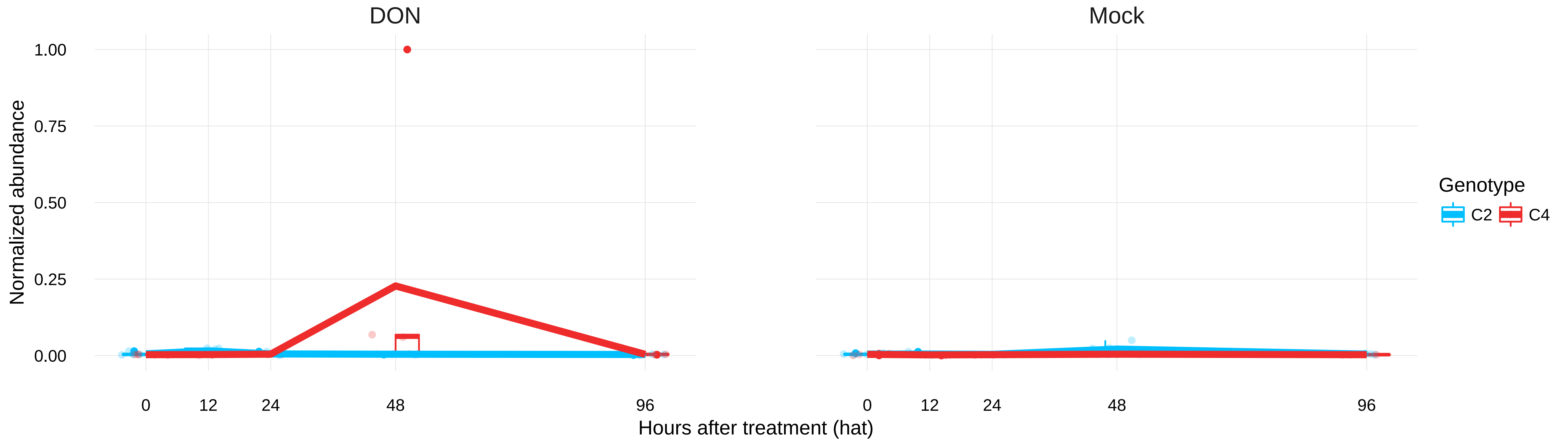

CM, Remus; different treatments

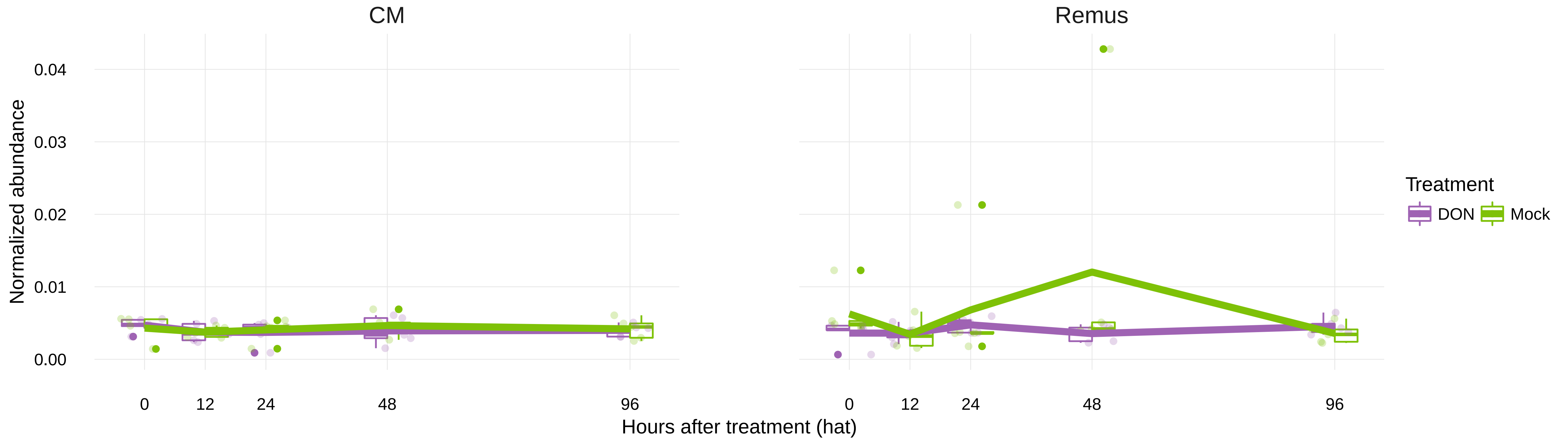

DON, Mock; all four genotypes

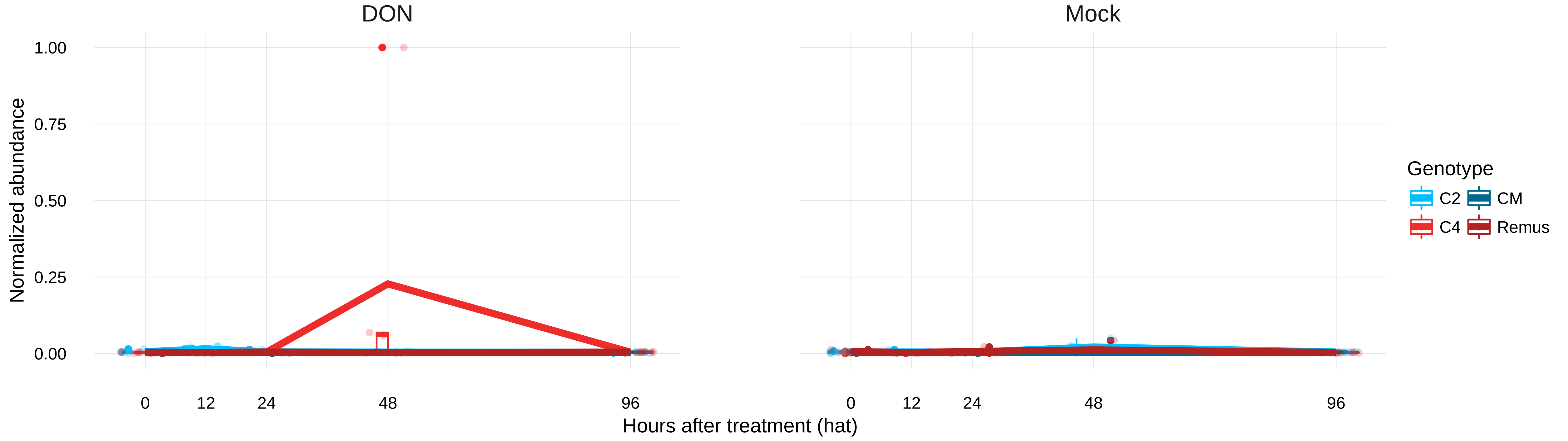

A.106

Annotated as Flavonoid
(30 database hits)

MZ	373.1281
RT	17.44 min
Normalization	Indirectly via surrogate in the KPX samples
Cluster	–
Cn total / Phe	20 / 9

C2, C4; different treatments

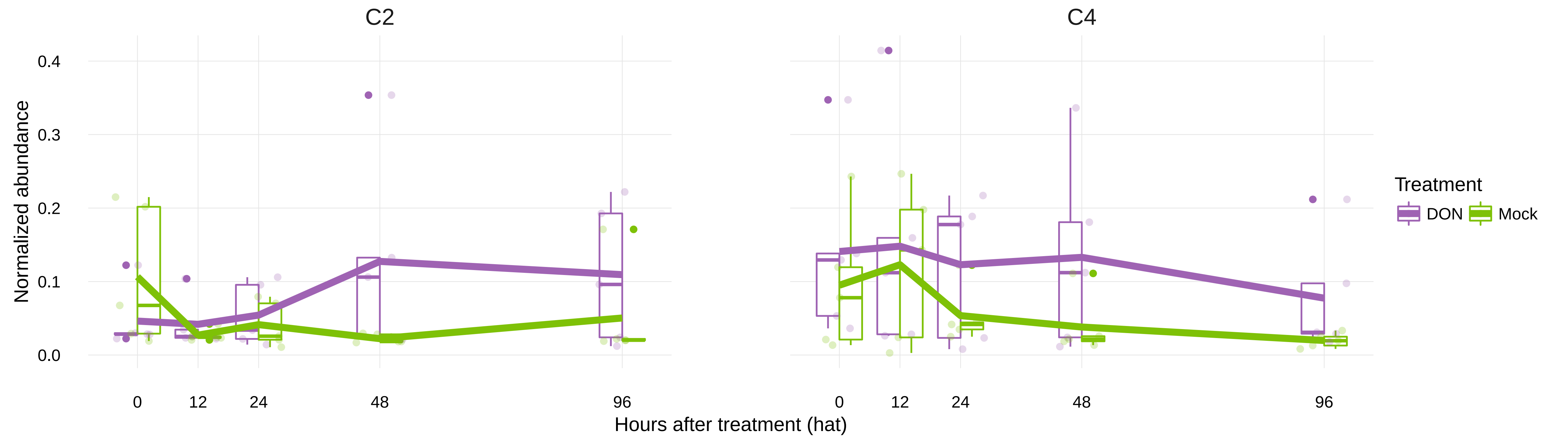

DON, Mock; different genotypes

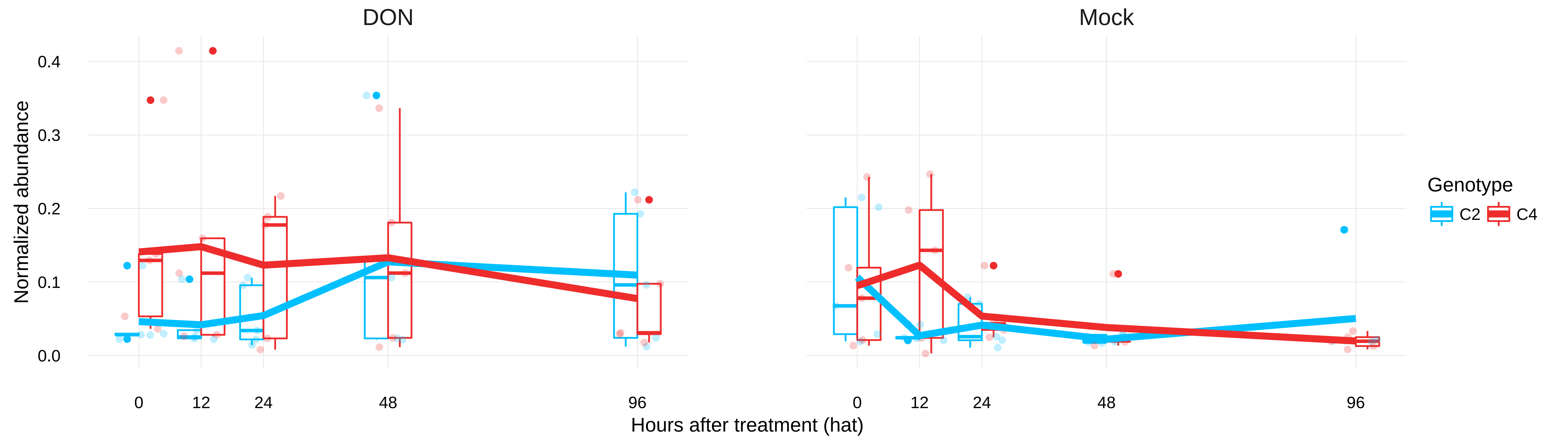

CM, Remus; different treatments

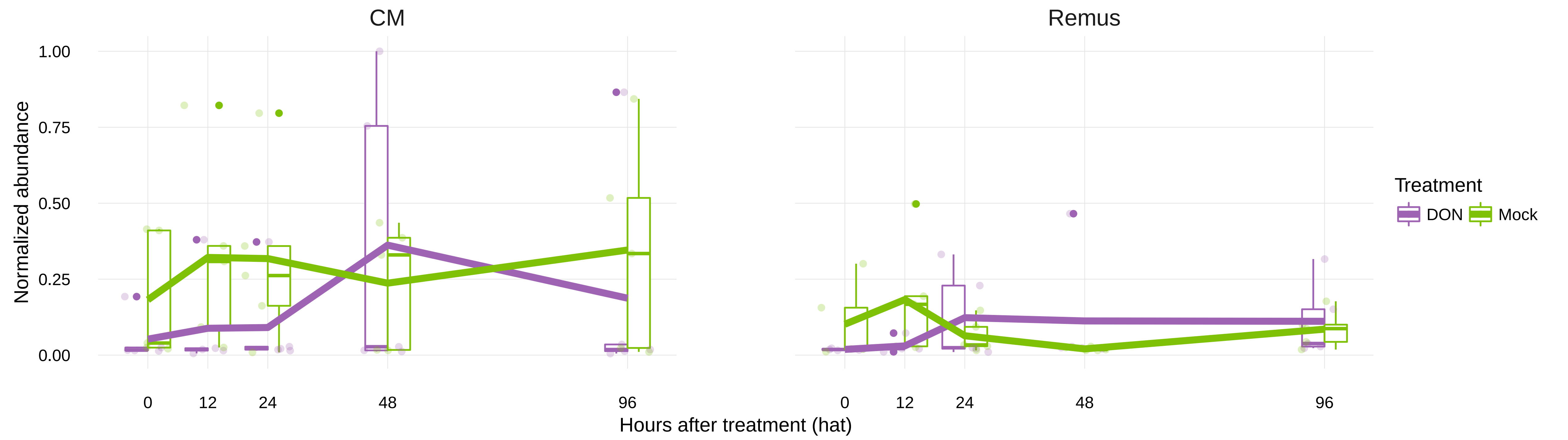

DON, Mock; all four genotypes

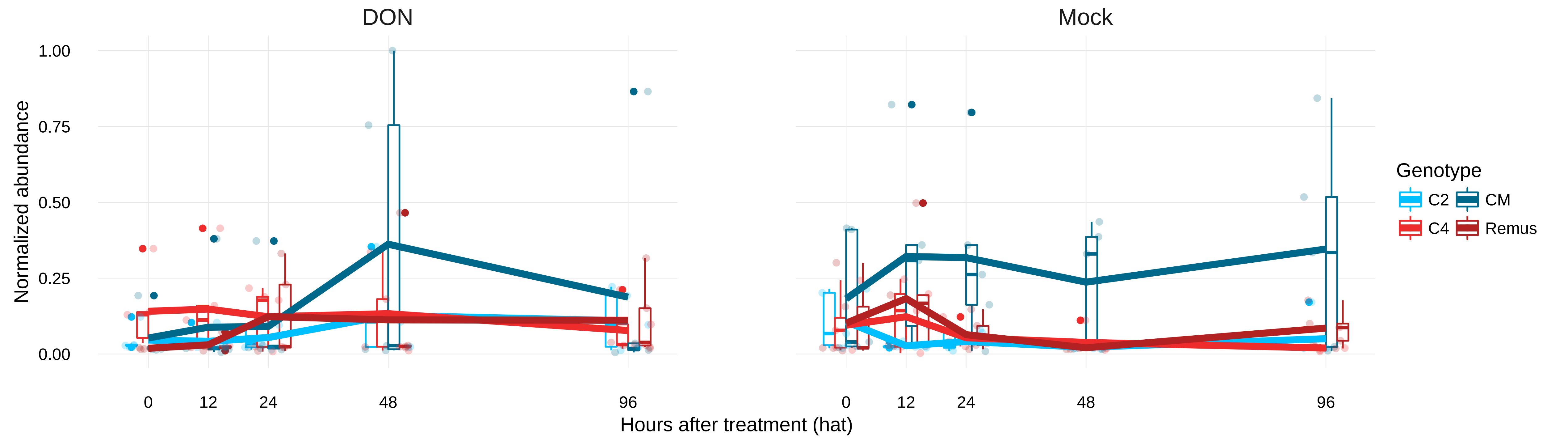

A.6

Annotated as others

(4 database hits)

MZ	137.0597
RT	4.18 min
Normalization	Not normalized
Cluster	–
Cn total / Phe	8 /

C2, C4; different treatments

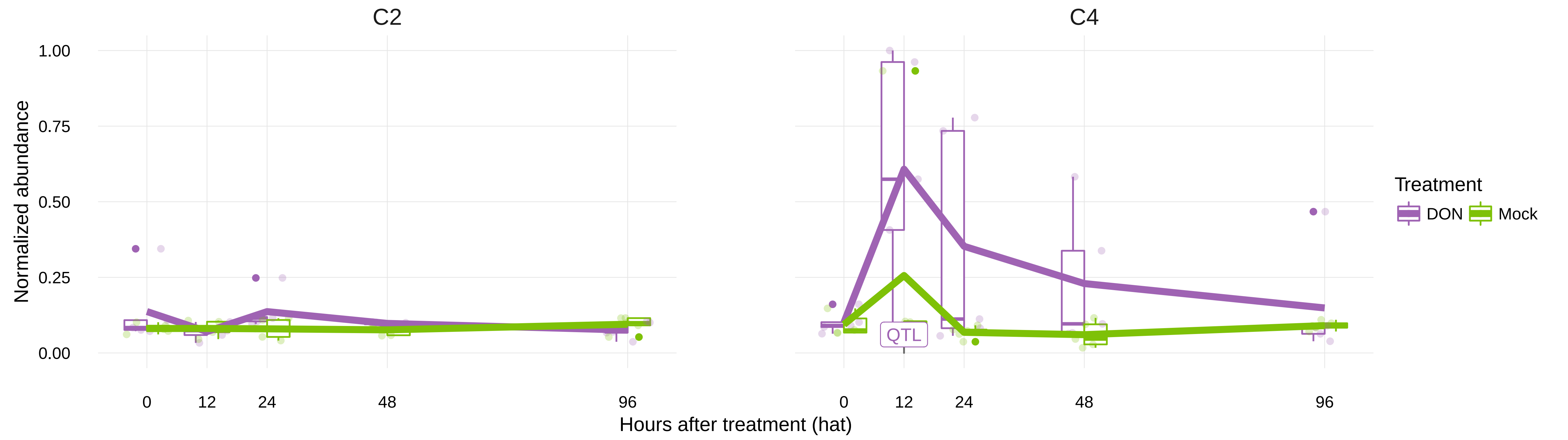

DON, Mock; different genotypes

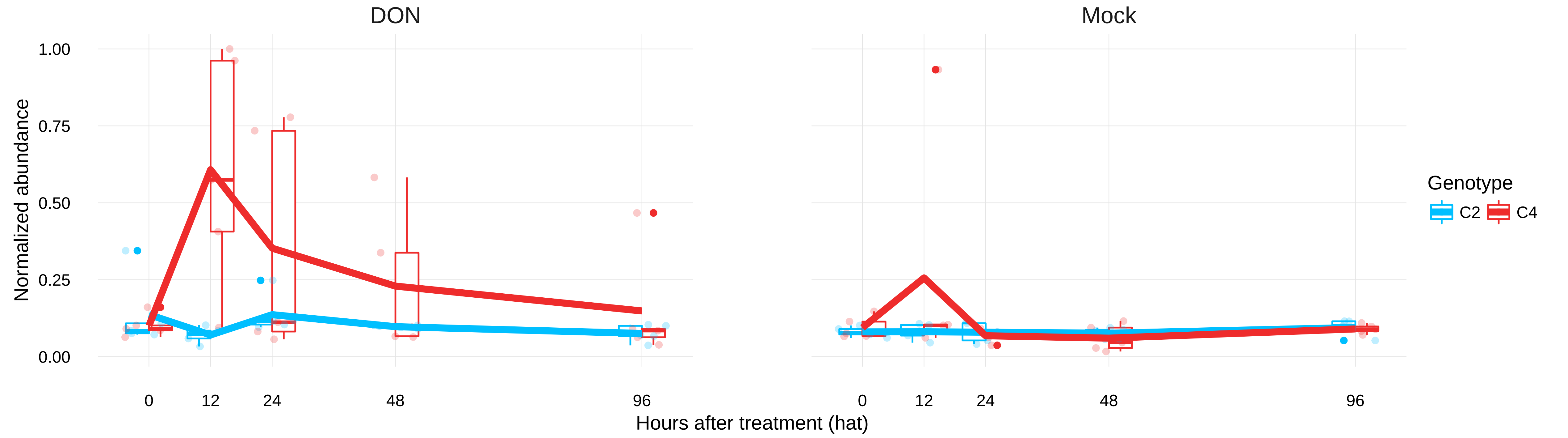

CM, Remus; different treatments

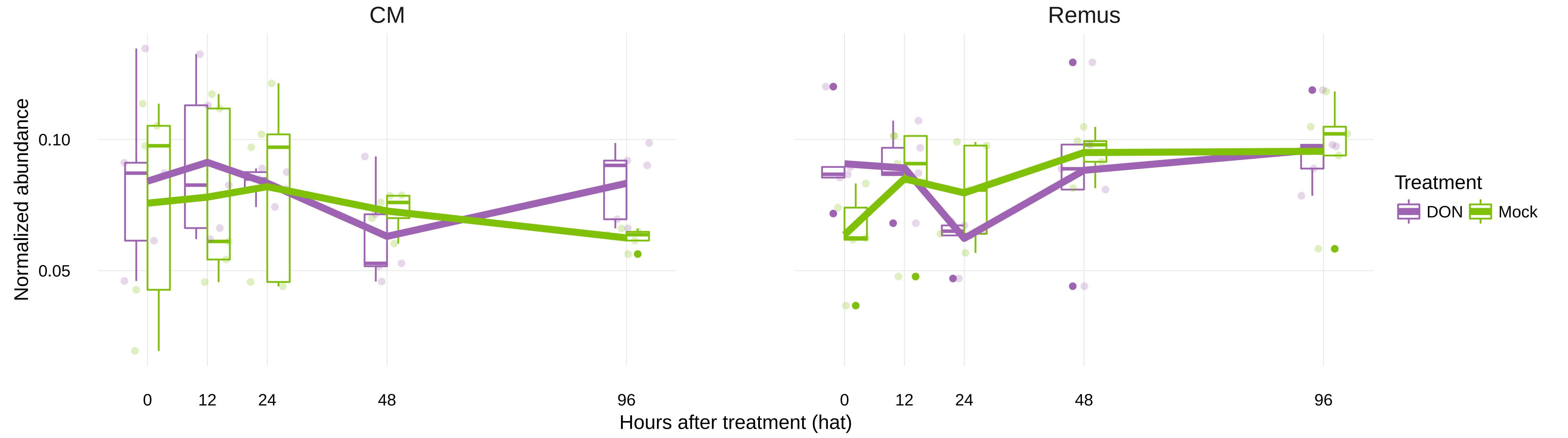

DON, Mock; all four genotypes

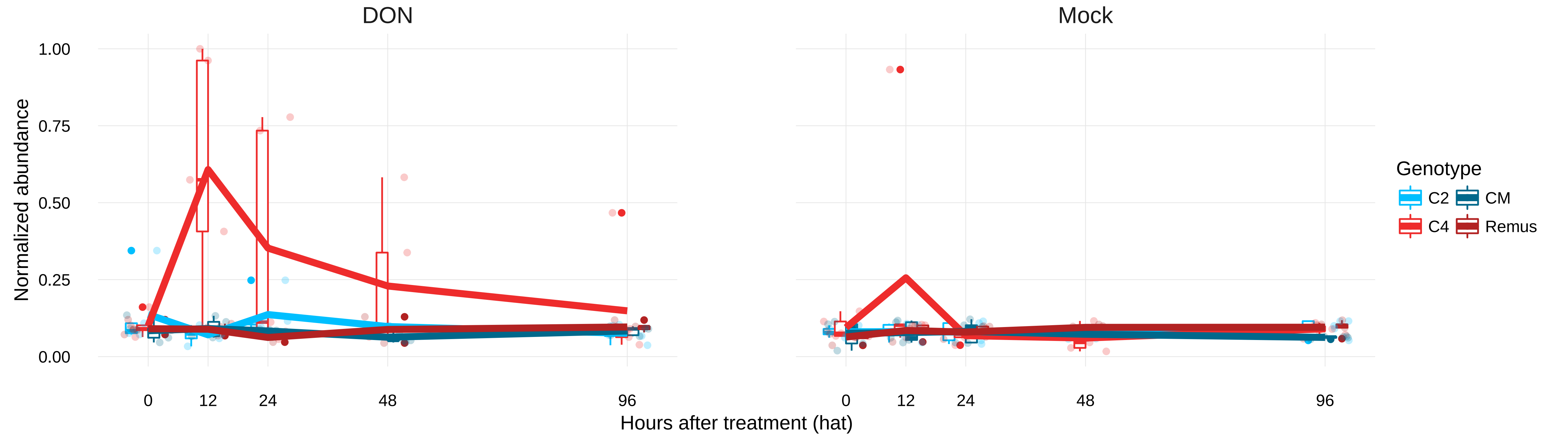

A.33

Annotated as HCA derivative
(1 database hit)

MZ	395.1313
RT	9.66 min
Normalization	Indirectly via surrogate in the KPX samples
Cluster	–
Cn total / Phe	17 /

C2, C4; different treatments

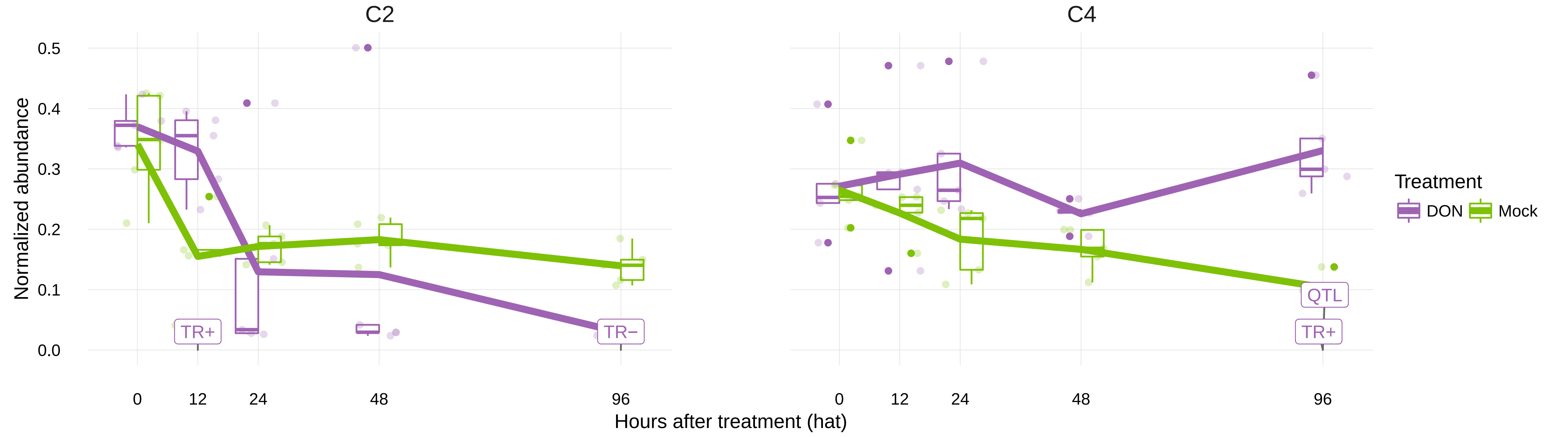

DON, Mock; different genotypes

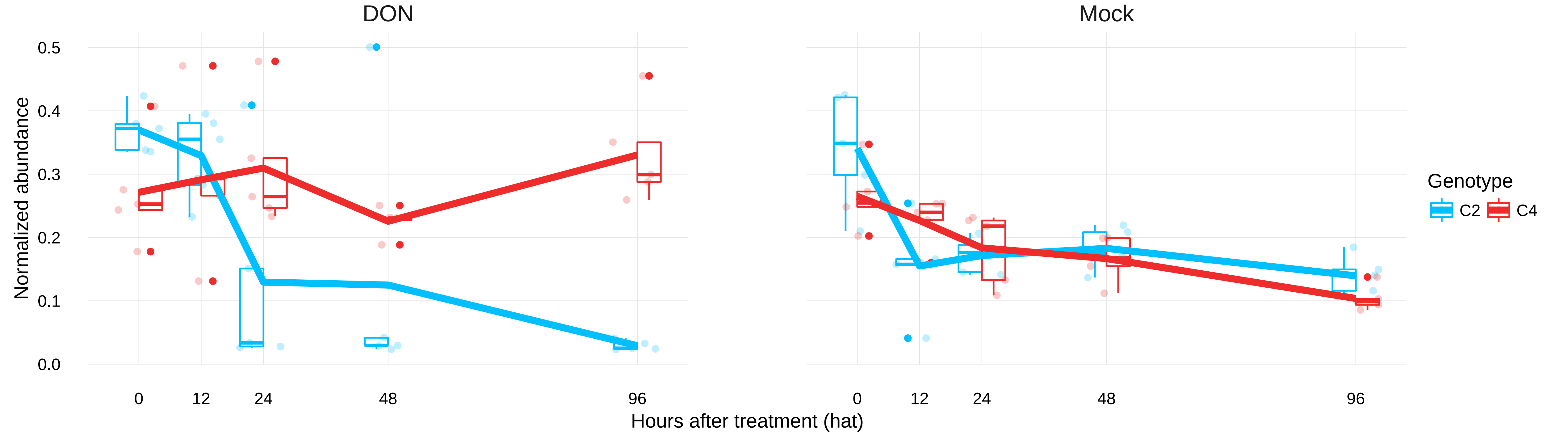

CM, Remus; different treatments

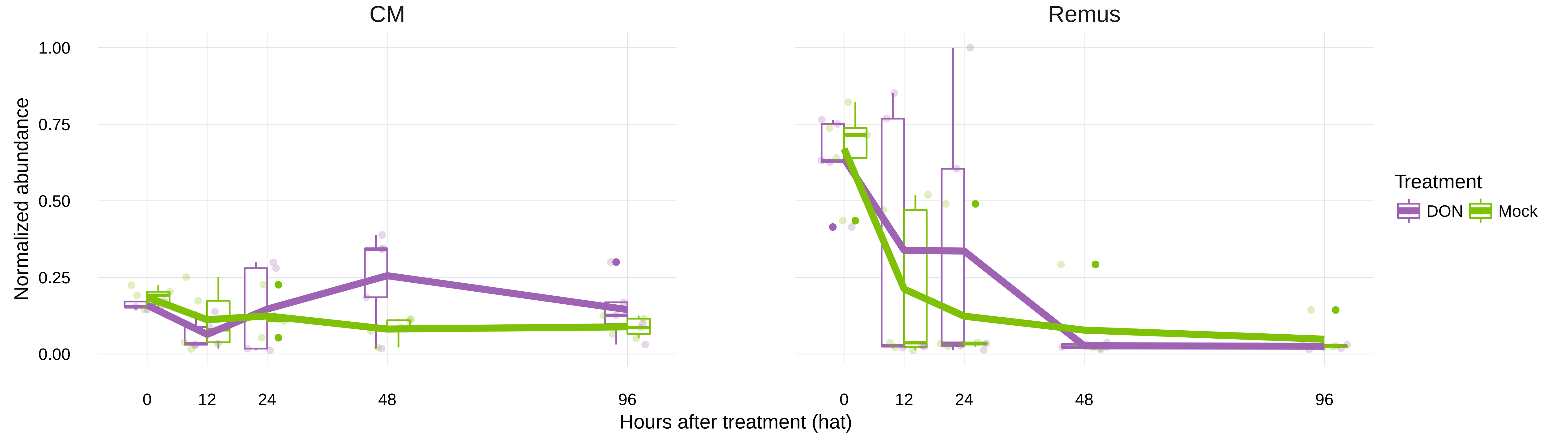

DON, Mock; all four genotypes

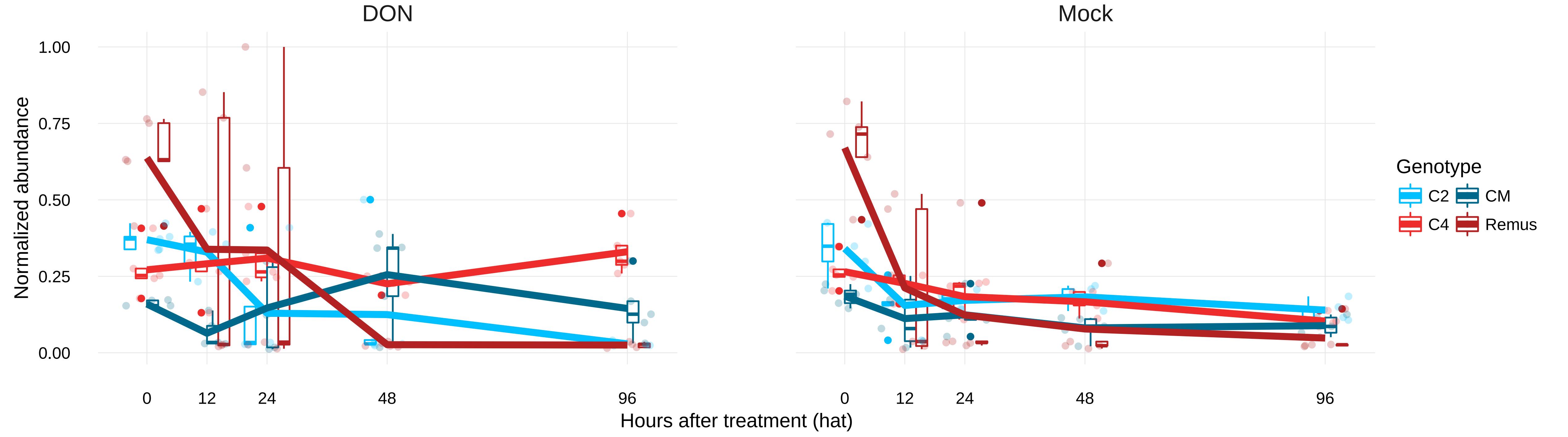

A.90

Annotated as Flavonoid (GlcF)
(69 database hits)

MZ	463.1237
RT	15.58 min
Normalization	Indirectly via surrogate in the KPX samples
Cluster	–
Cn total / Phe	22 /

C2, C4; different treatments

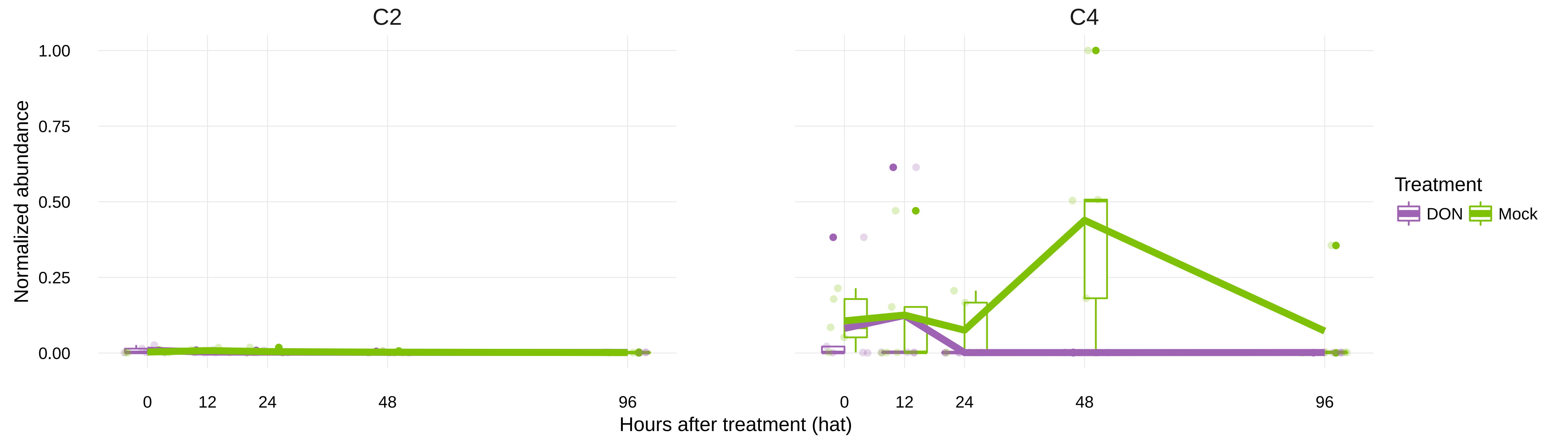

DON, Mock; different genotypes

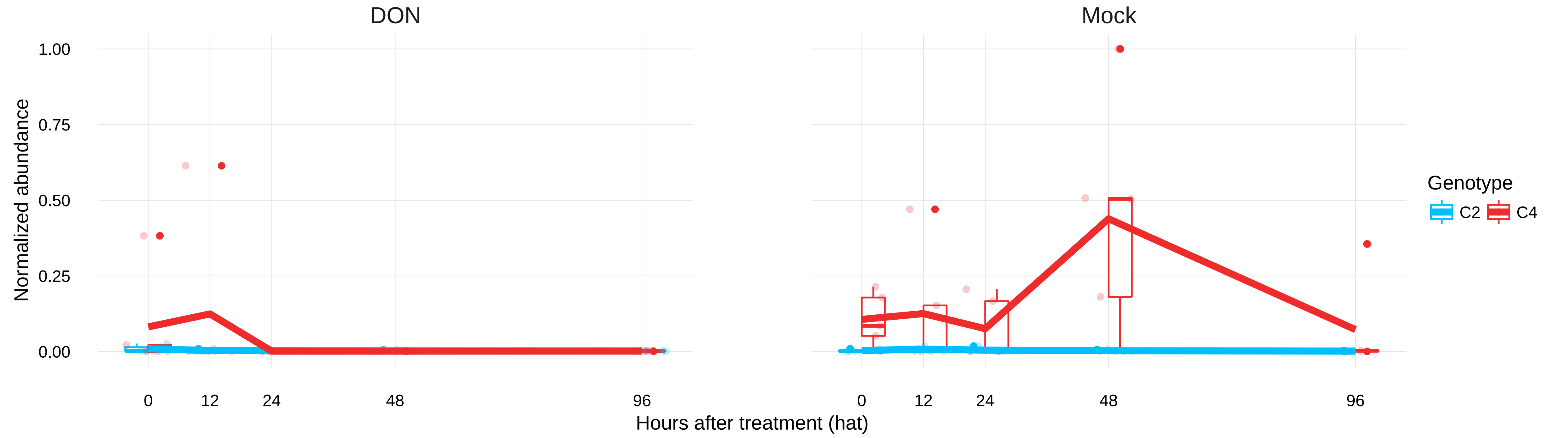

CM, Remus; different treatments

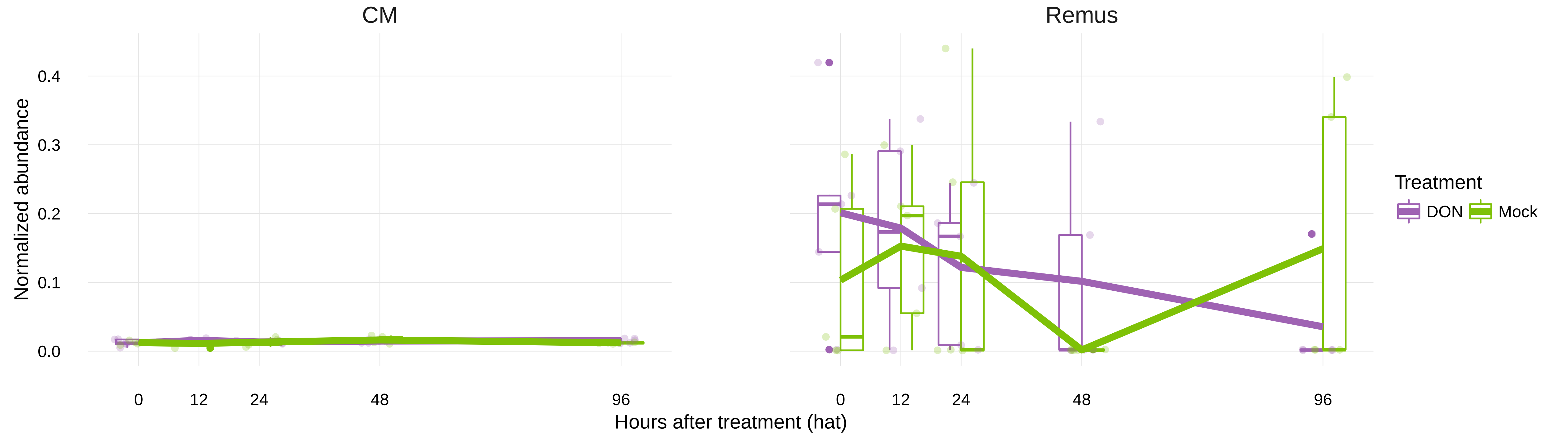

DON, Mock; all four genotypes

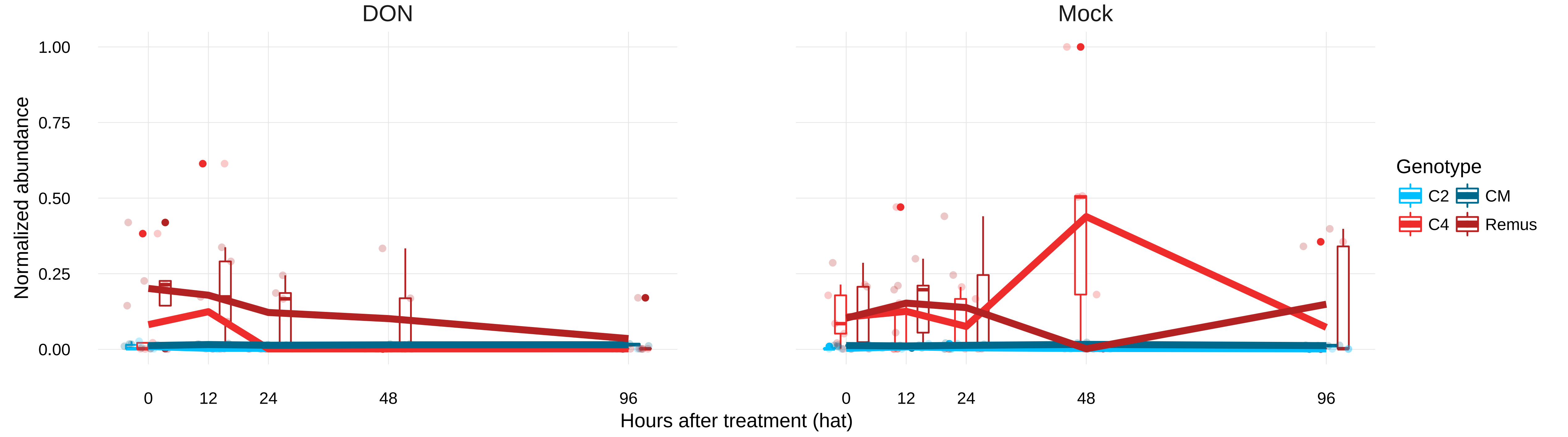

A.89

Annotated as Flavonoid (GlcF)
(68 database hits)

MZ	433.1131
RT	15.33 min
Normalization	Indirectly via surrogate in the KPX samples
Cluster	–
Cn total / Phe	21 /

C2, C4; different treatments

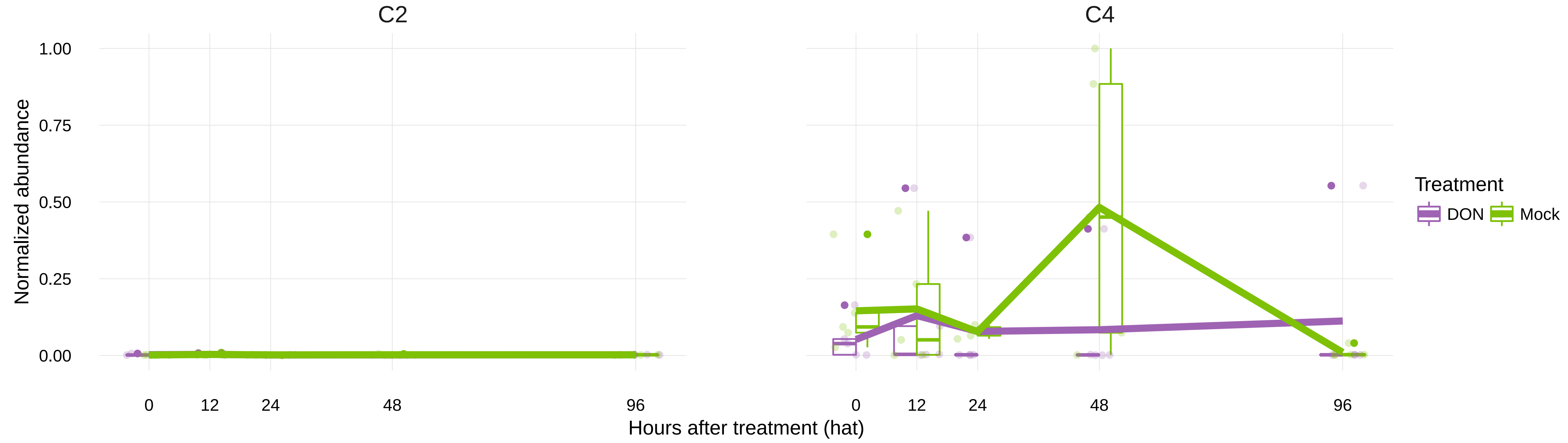

DON, Mock; different genotypes

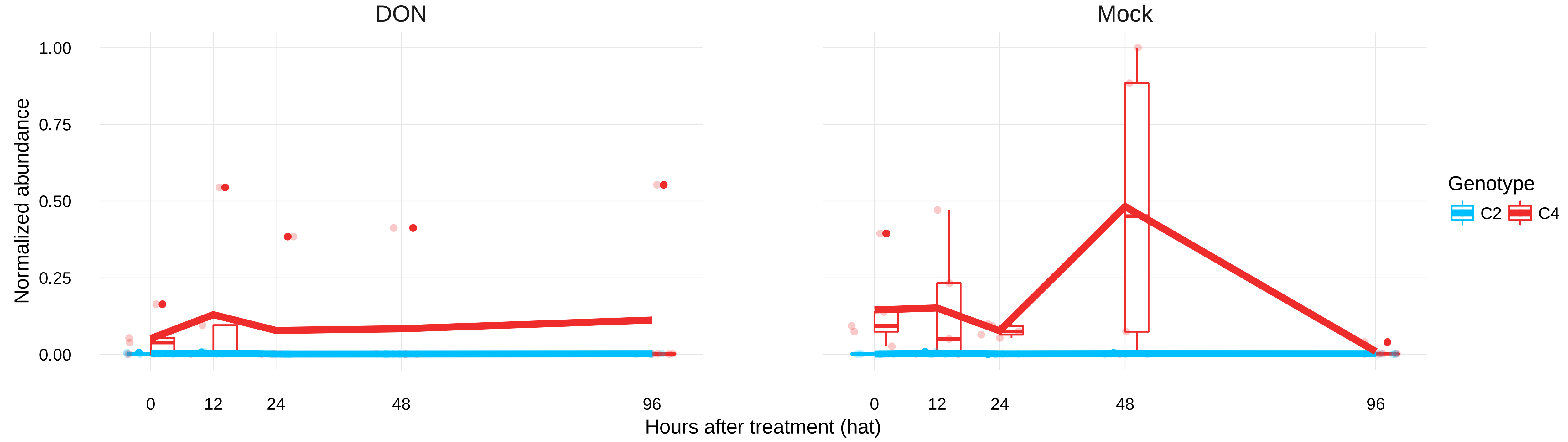

CM, Remus; different treatments

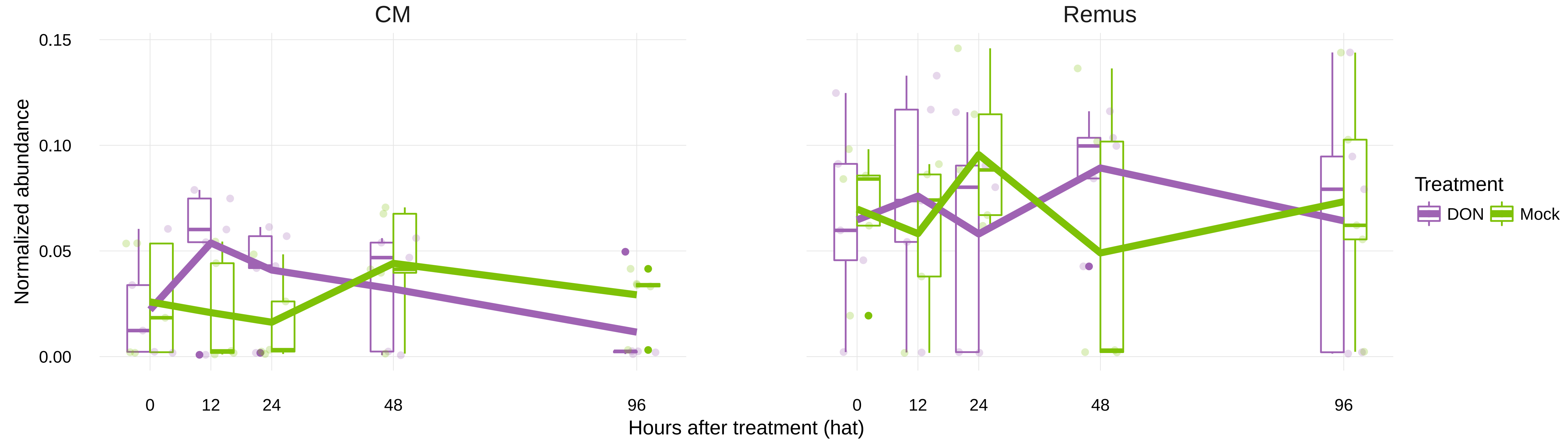

DON, Mock; all four genotypes

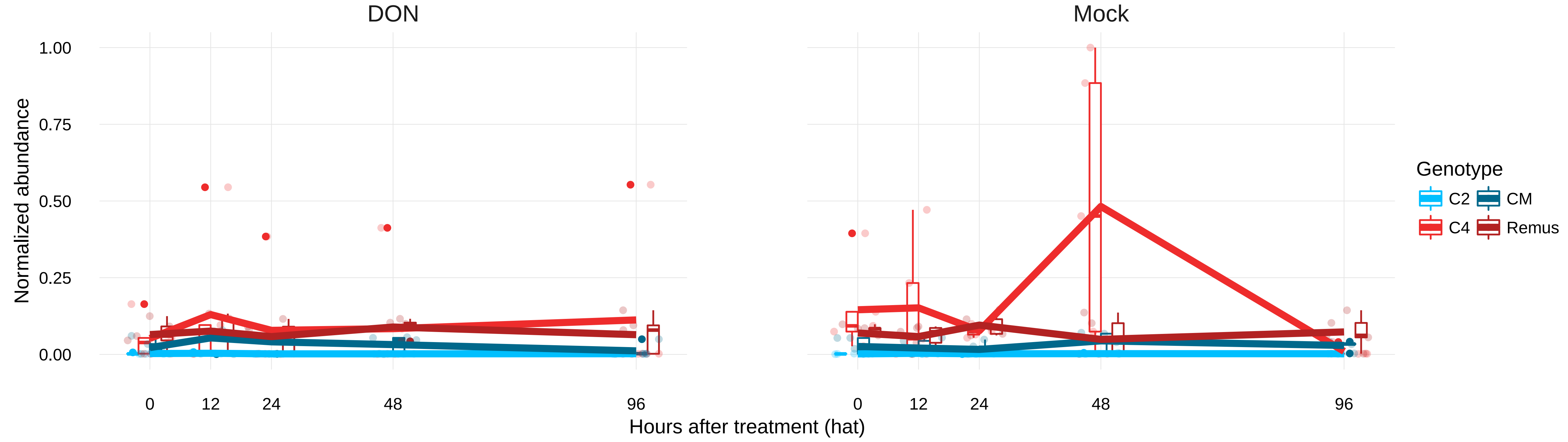

FerTam

Identified metabolite (level 1)

MZ	337.1548
RT	20.97 min
Normalization	Indirectly via surrogate in the KPX samples
Cluster	Cluster 1B
Cn total / Phe	20 / 9

C2, C4; different treatments

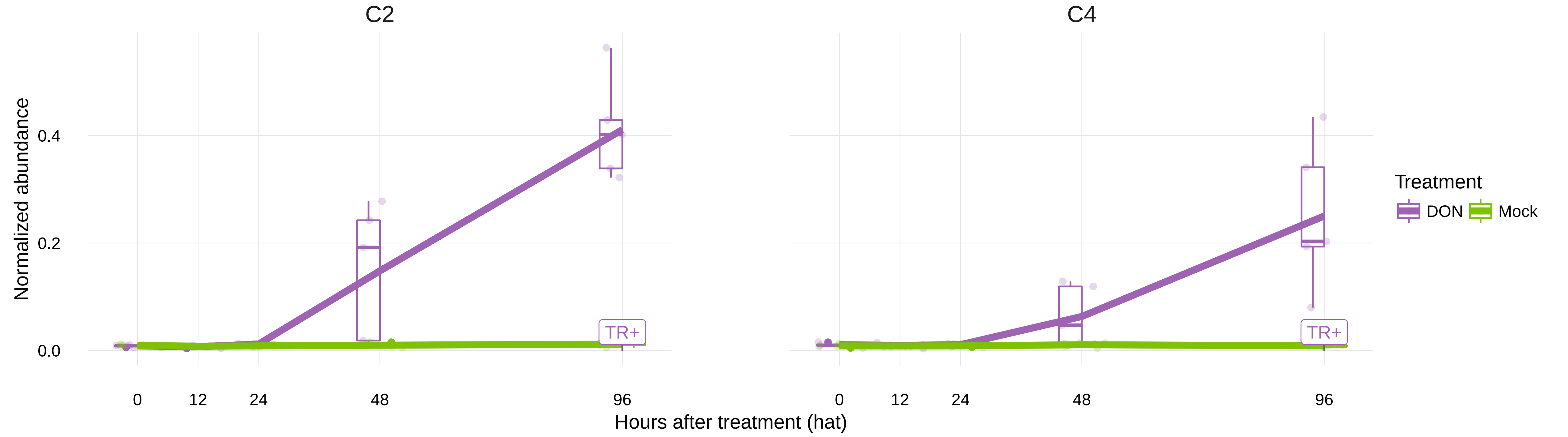

DON, Mock; different genotypes

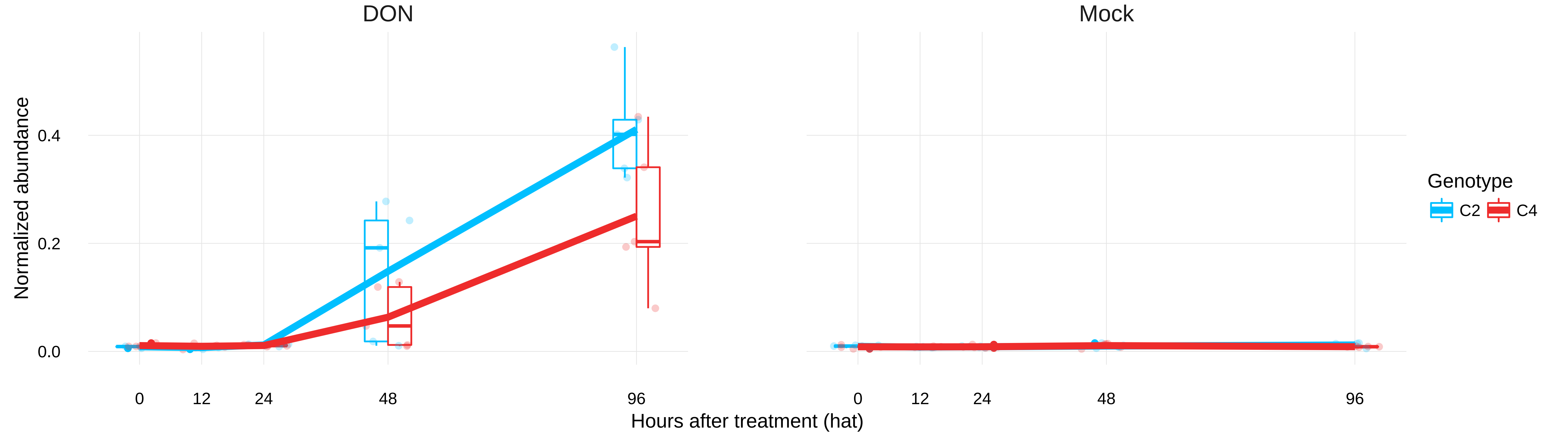

CM, Remus; different treatments

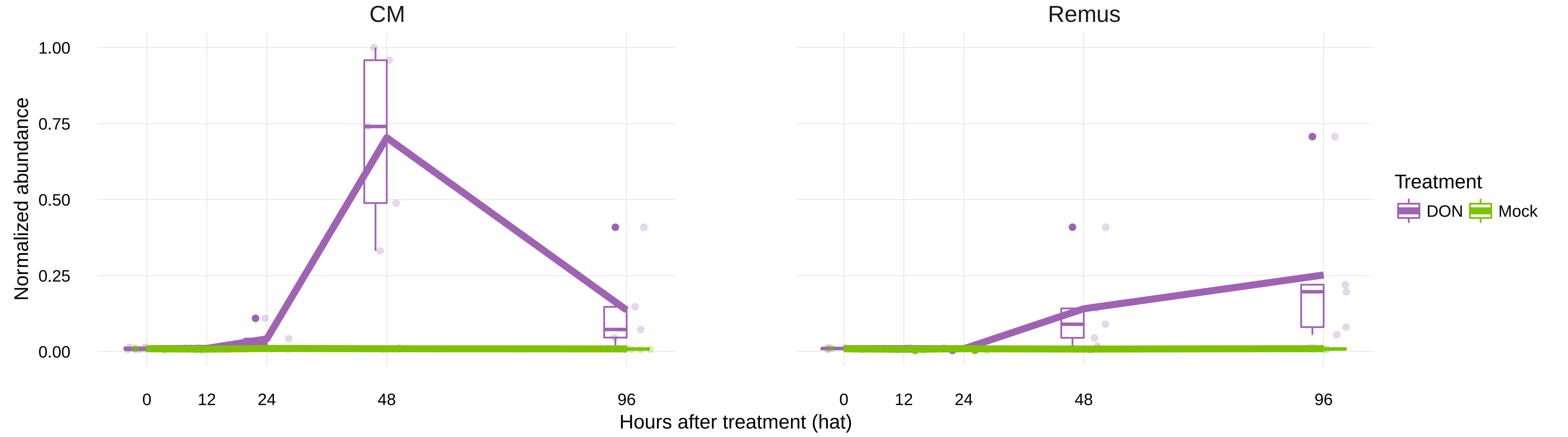

DON, Mock; all four genotypes

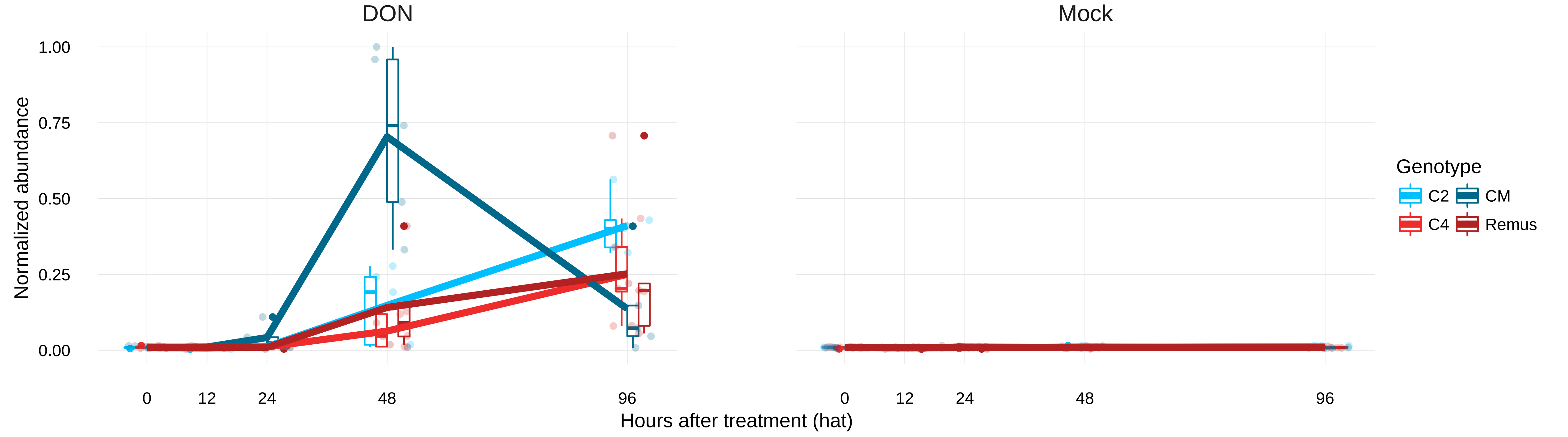

A.24

Annotated as others (dipeptide)

(1 database hit)

MZ	265.1554
RT	8.75 min
Normalization	Directly via KPX samples
Cluster	Cluster 1B
Cn total / Phe	9 / 9

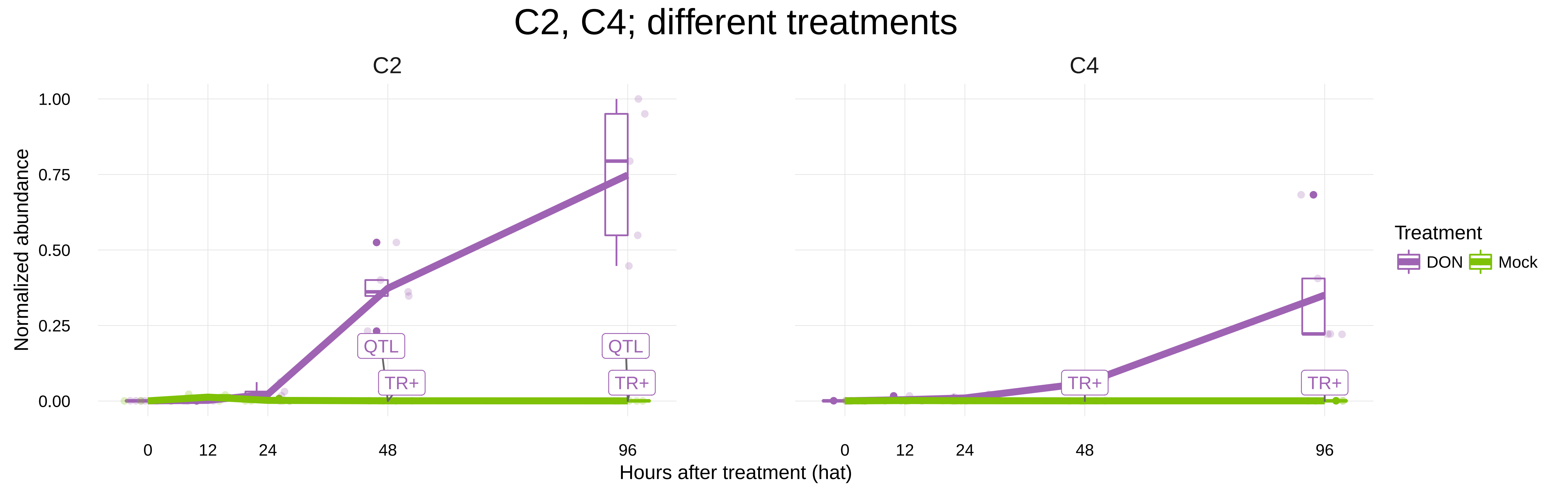

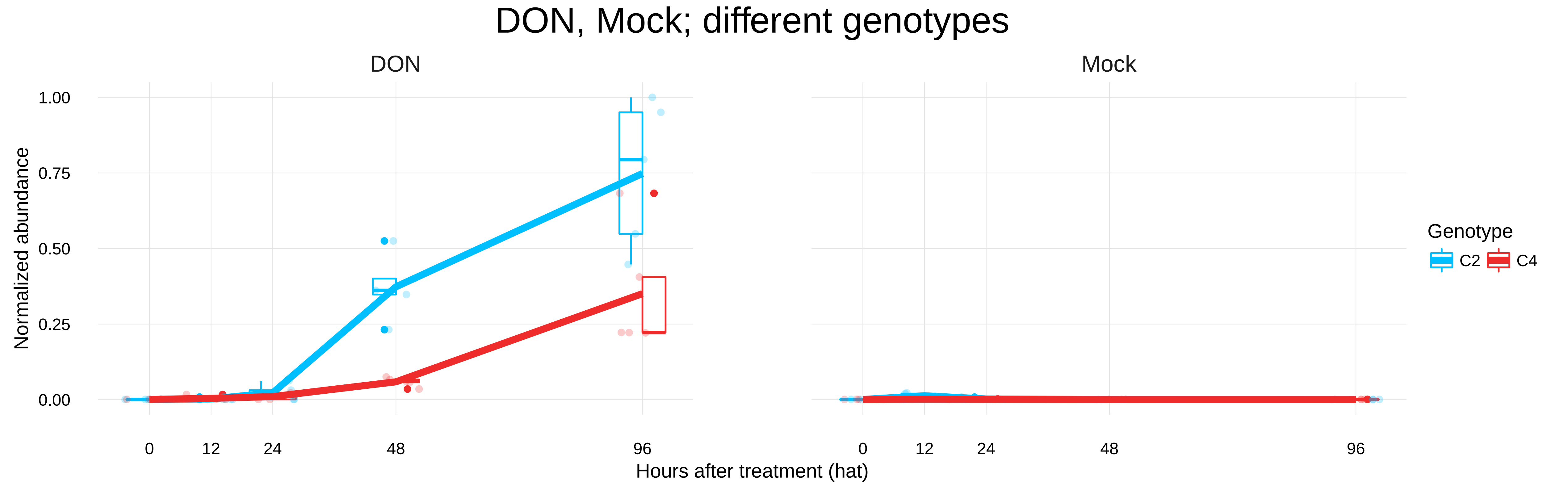

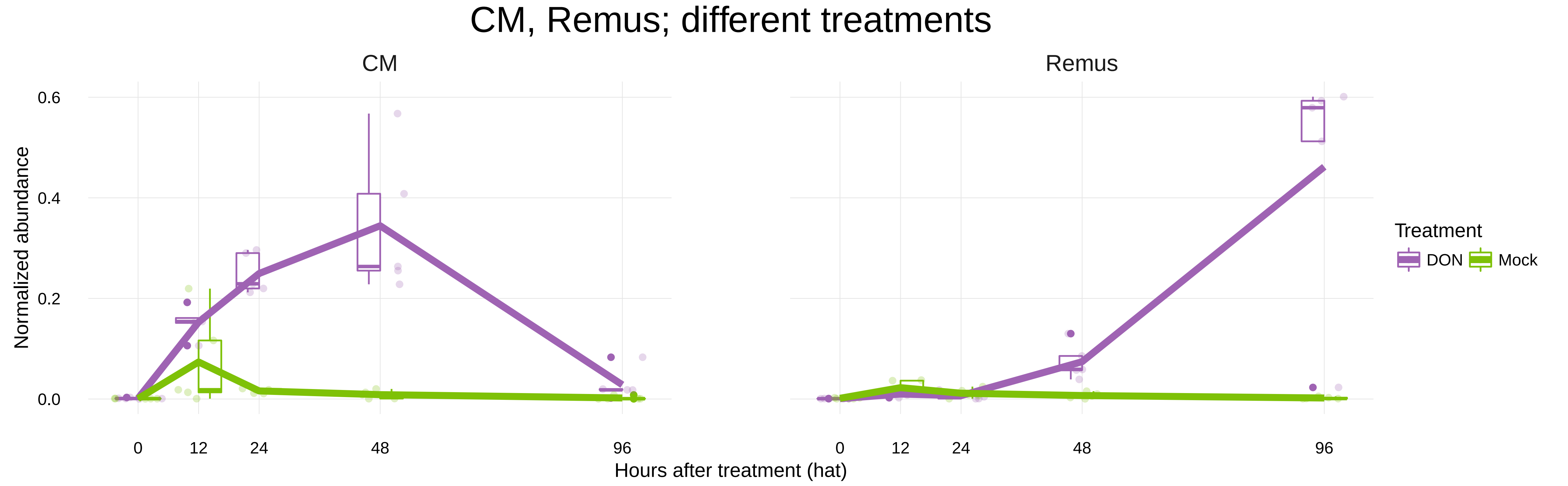

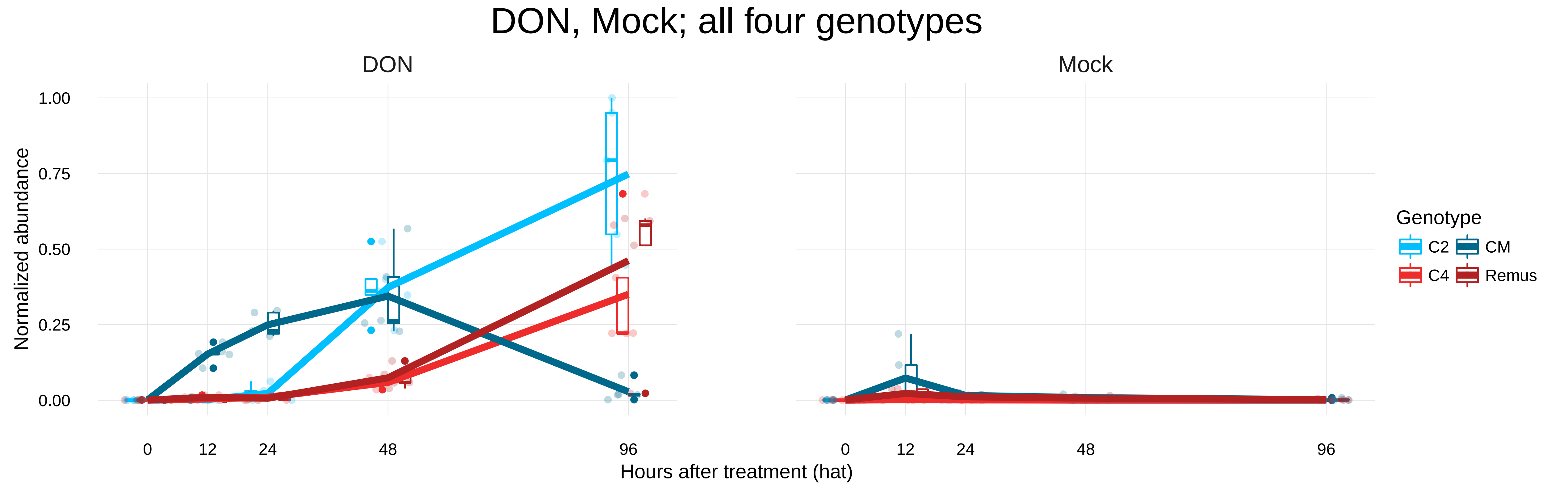

A.41

Annotated as HCA derivative
(1 database hit)

MZ	335.1106
RT	10.43 min
Normalization	Directly via KPX samples
Cluster	Cluster 1B
Cn total / Phe	15 /

C2, C4; different treatments

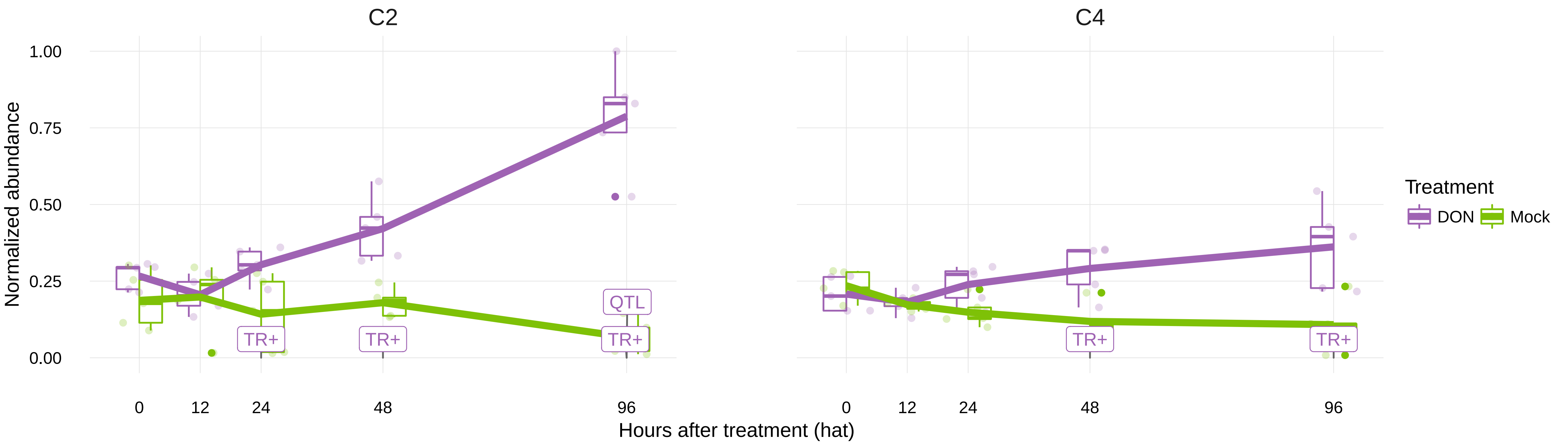

DON, Mock; different genotypes

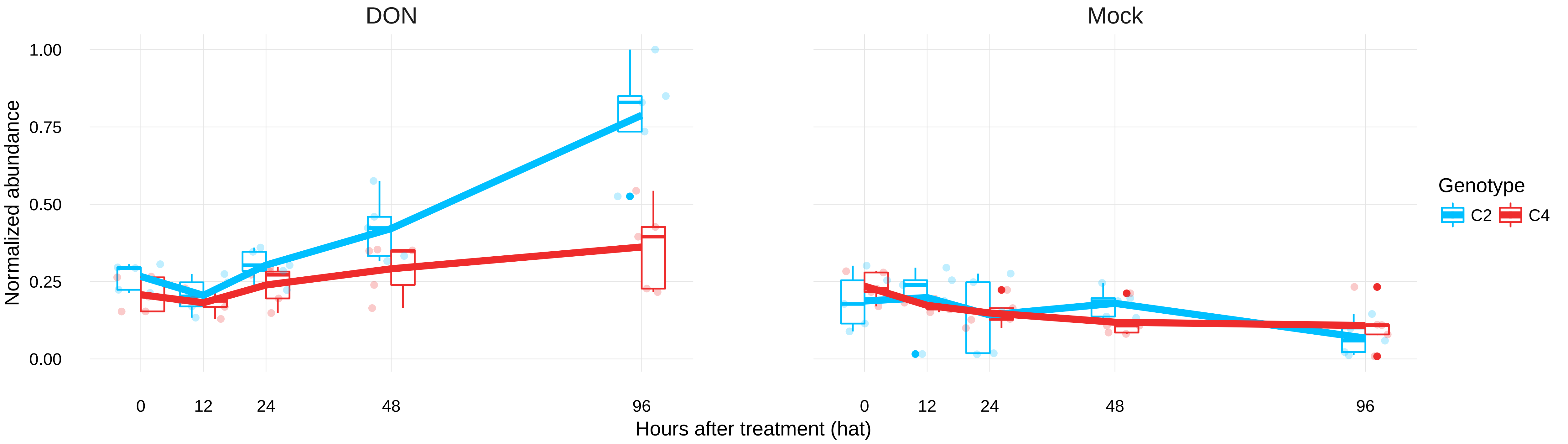

CM, Remus; different treatments

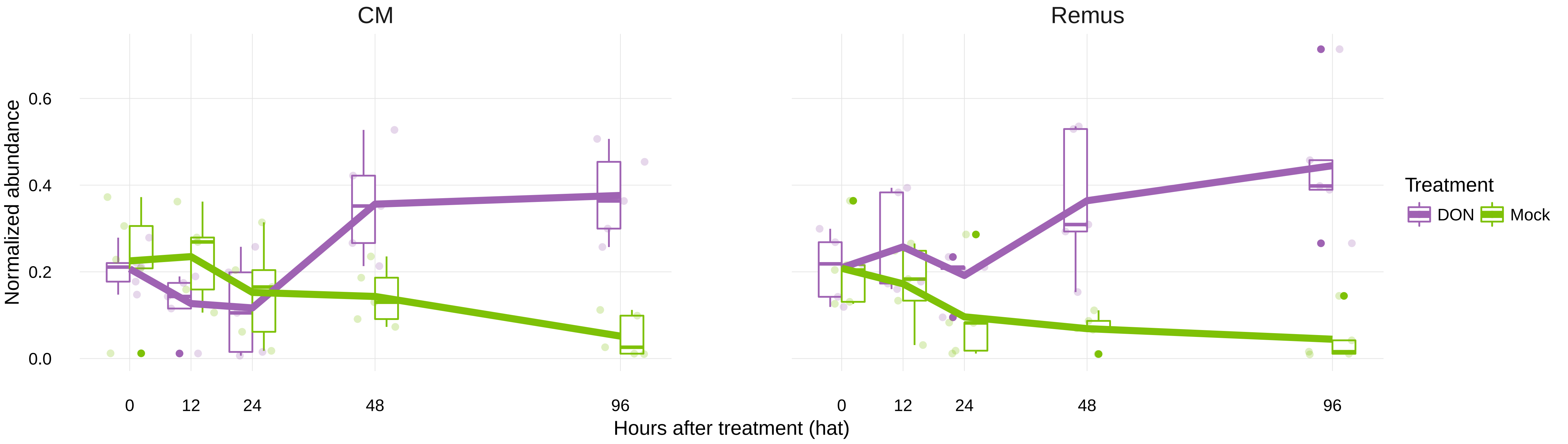

DON, Mock; all four genotypes

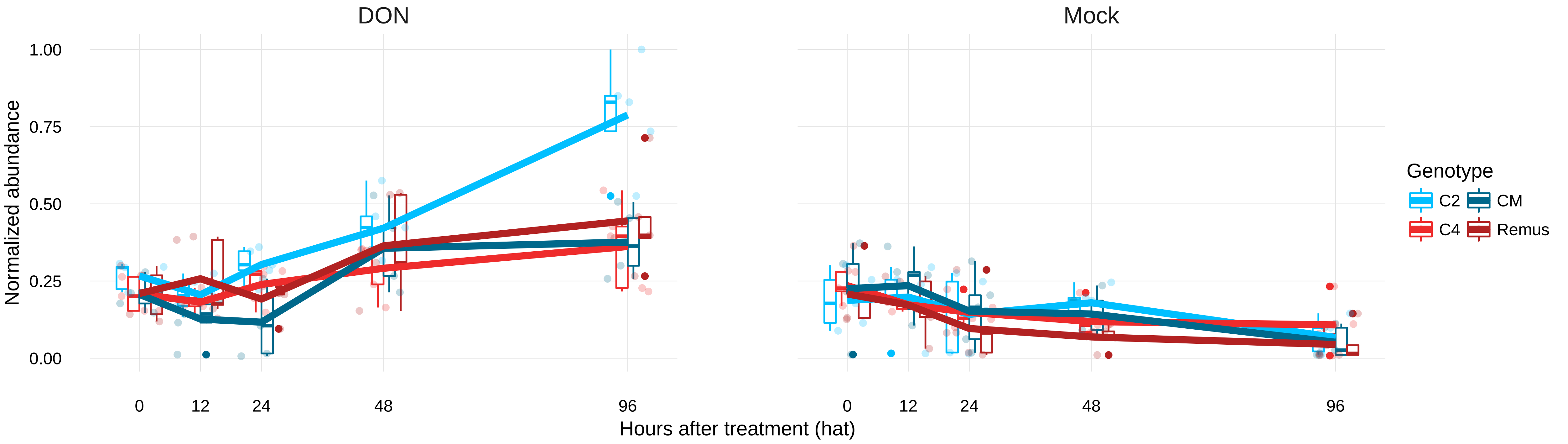

A.134

Annotated as HCA derivative
(1 database hit)

MZ	557.1654
RT	21.51 min
Normalization	Directly via KPX samples
Cluster	Cluster 1B
Cn total / Phe	28 / 18

C2, C4; different treatments

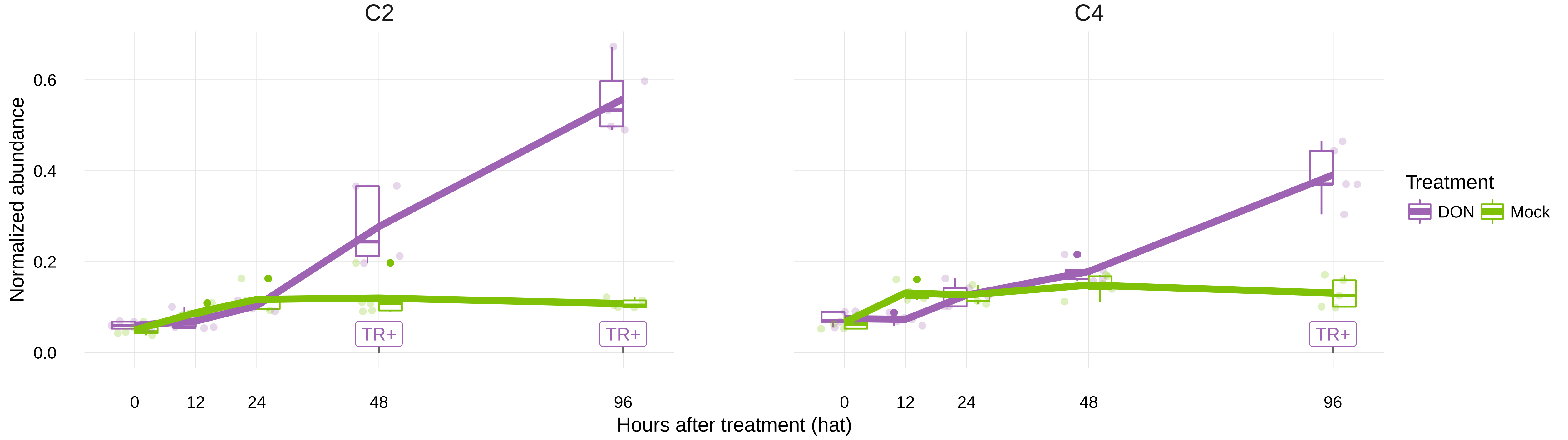

DON, Mock; different genotypes

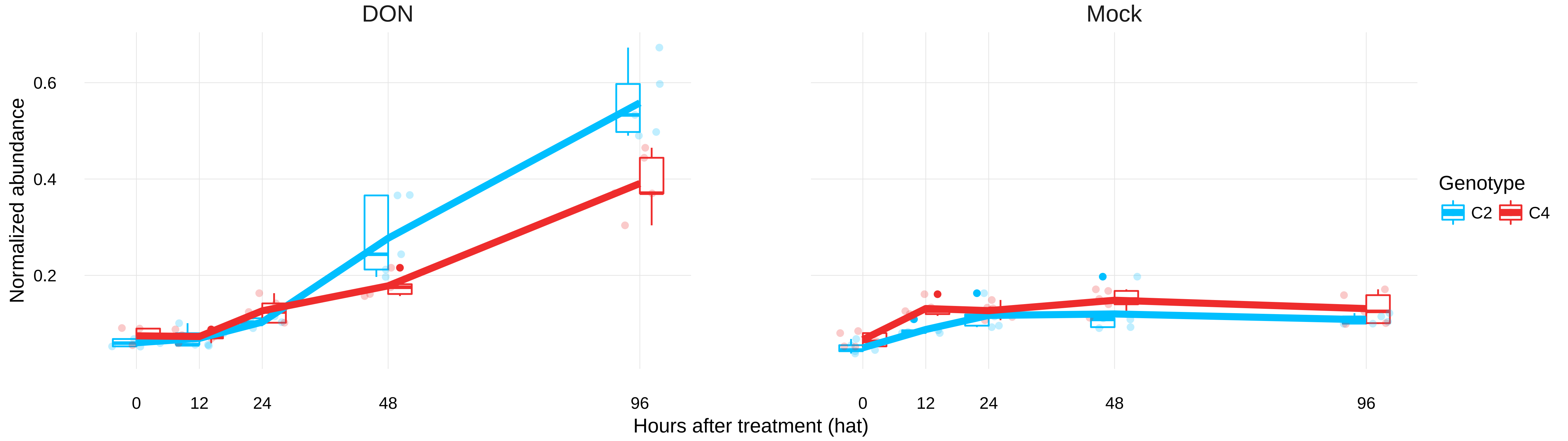

CM, Remus; different treatments

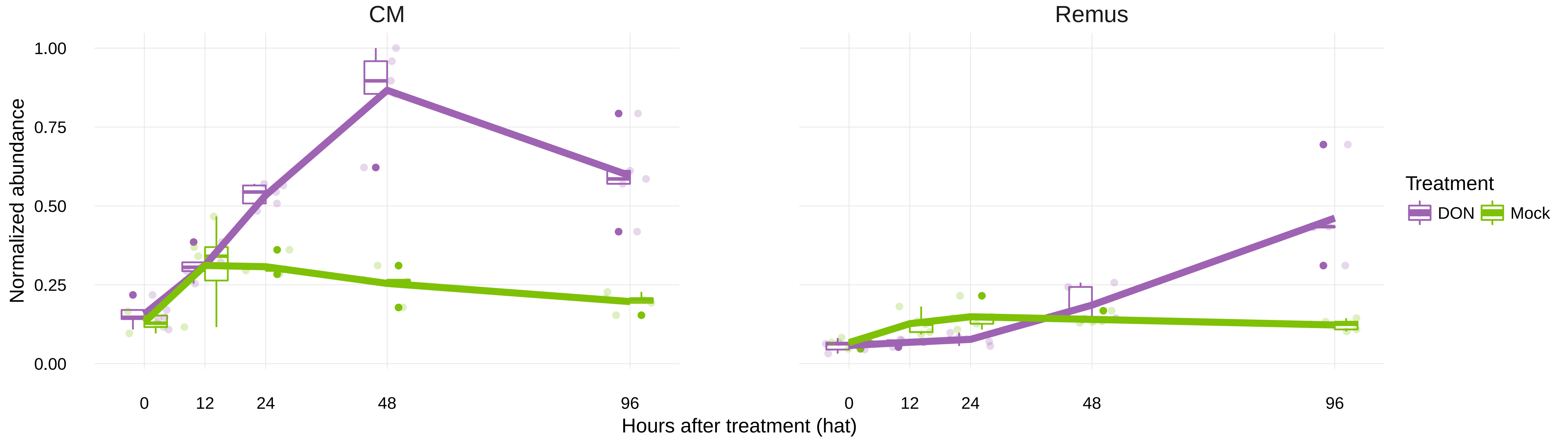

DON, Mock; all four genotypes

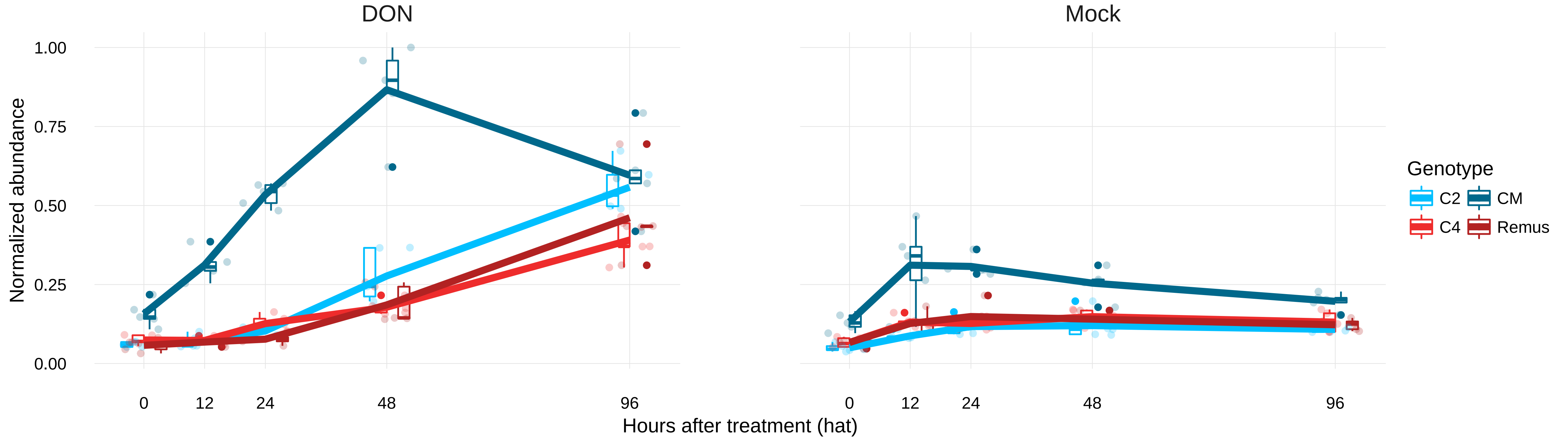

A.123

Annotated as Lignan or HCA derivative
(3 database hits)

MZ	515.1548
RT	19.78 min
Normalization	Directly via KPX samples
Cluster	Cluster 1B
Cn total / Phe	26 / Several: 18,9

C2, C4; different treatments

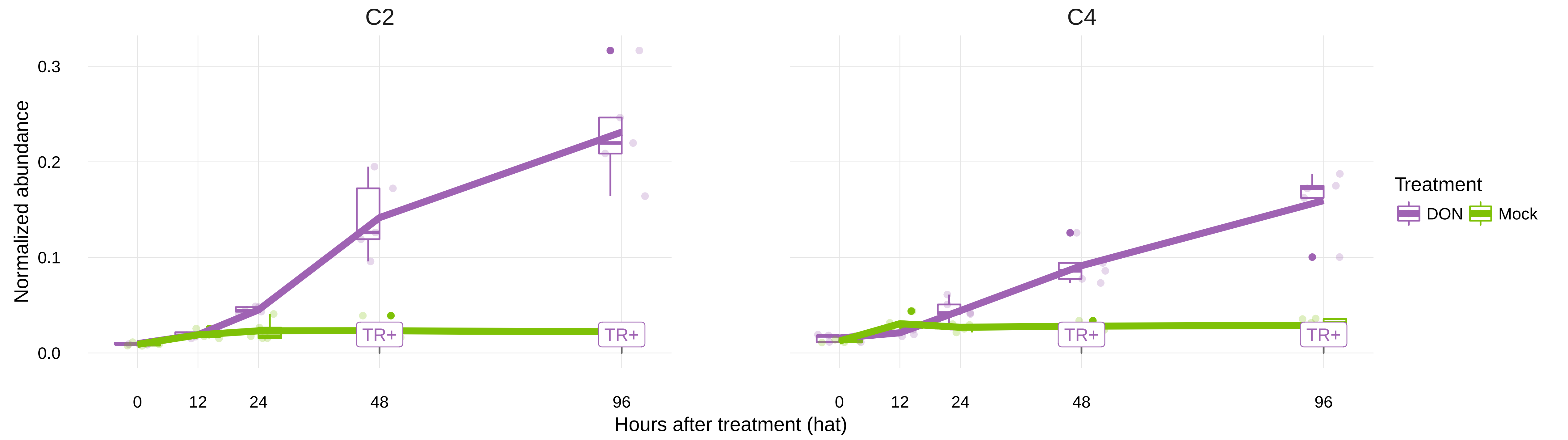

DON, Mock; different genotypes

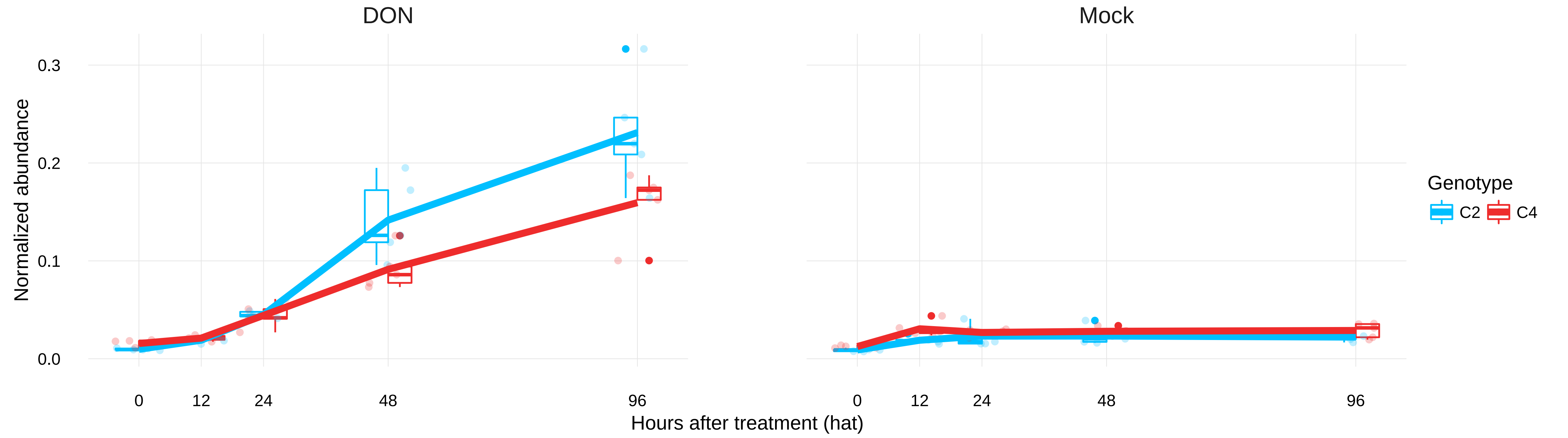

CM, Remus; different treatments

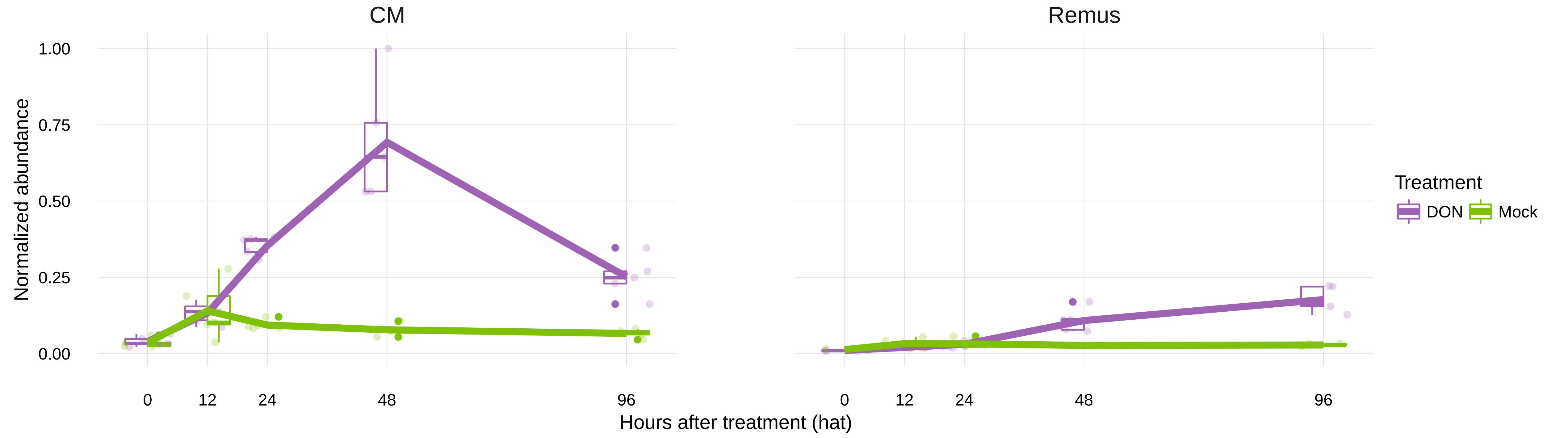

DON, Mock; all four genotypes

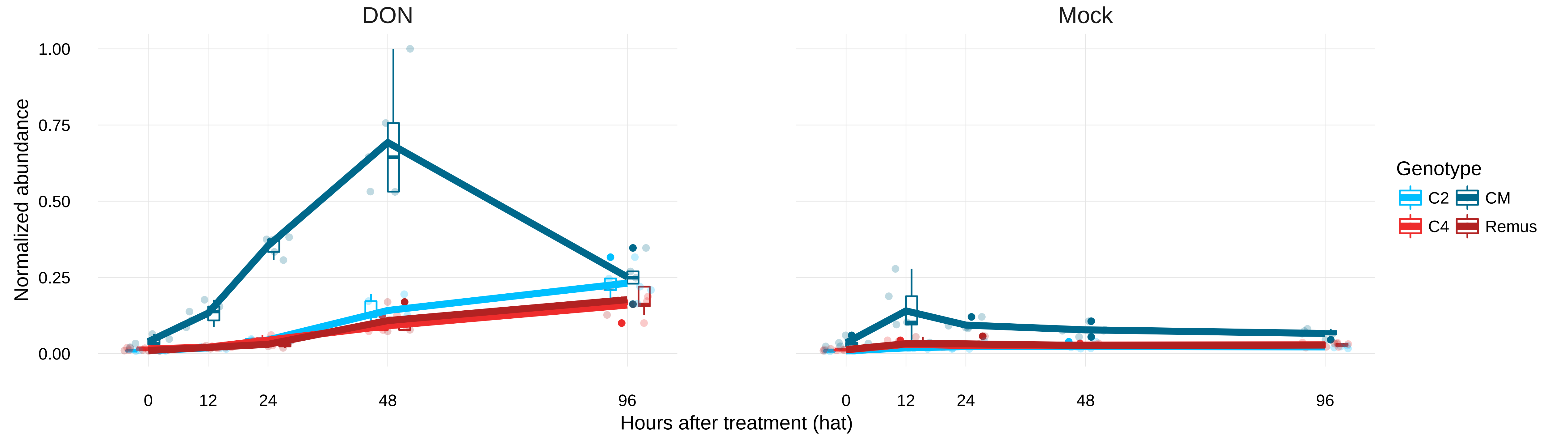

U.2

Unknown Phe−derived wheat−metabolite

MZ	163.0754
RT	8.71 min
Normalization	Directly via KPX samples
Cluster	Cluster 1B
Cn total / Phe	10 /

C2, C4; different treatments

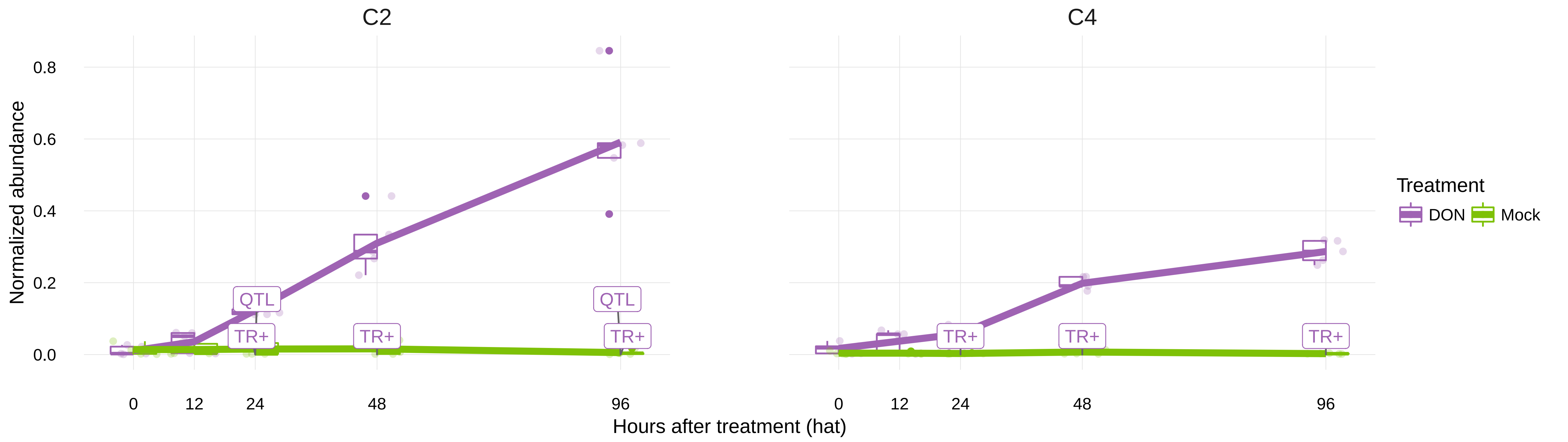

DON, Mock; different genotypes

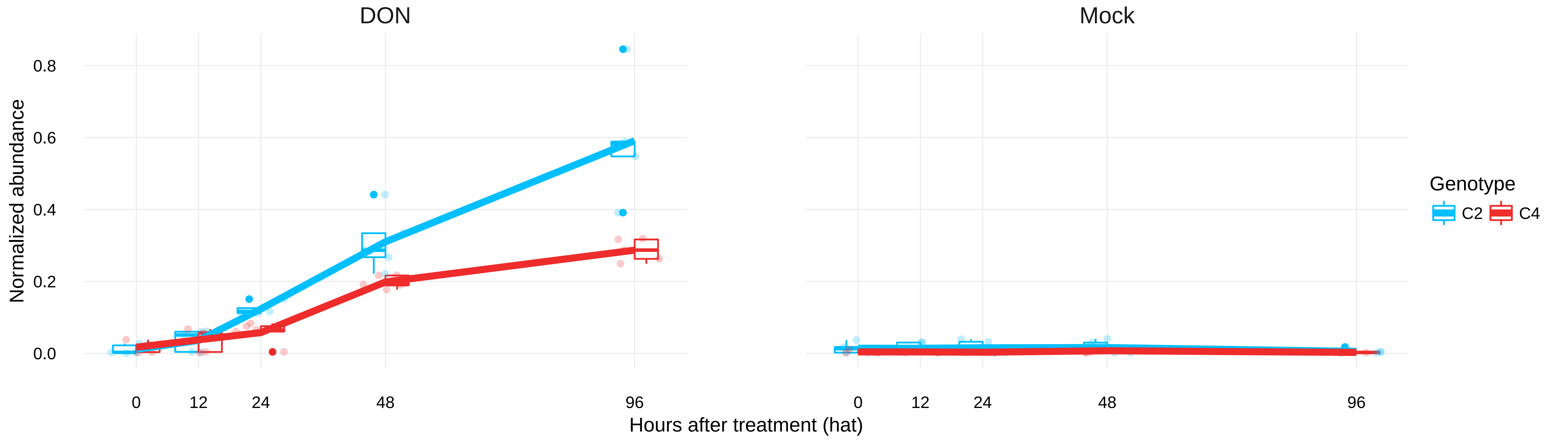

CM, Remus; different treatments

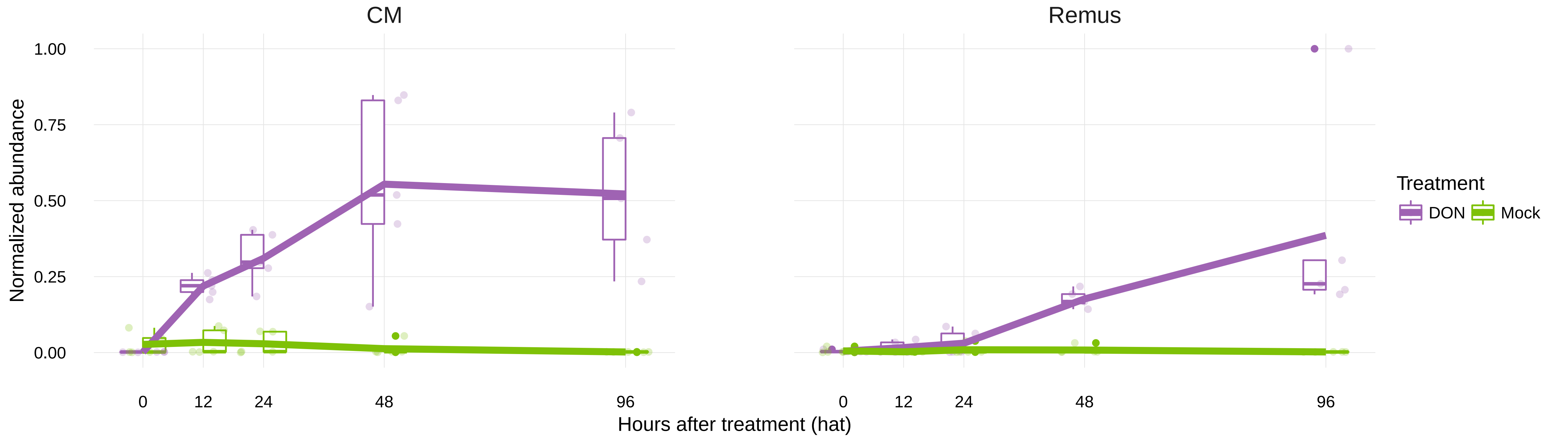

DON, Mock; all four genotypes

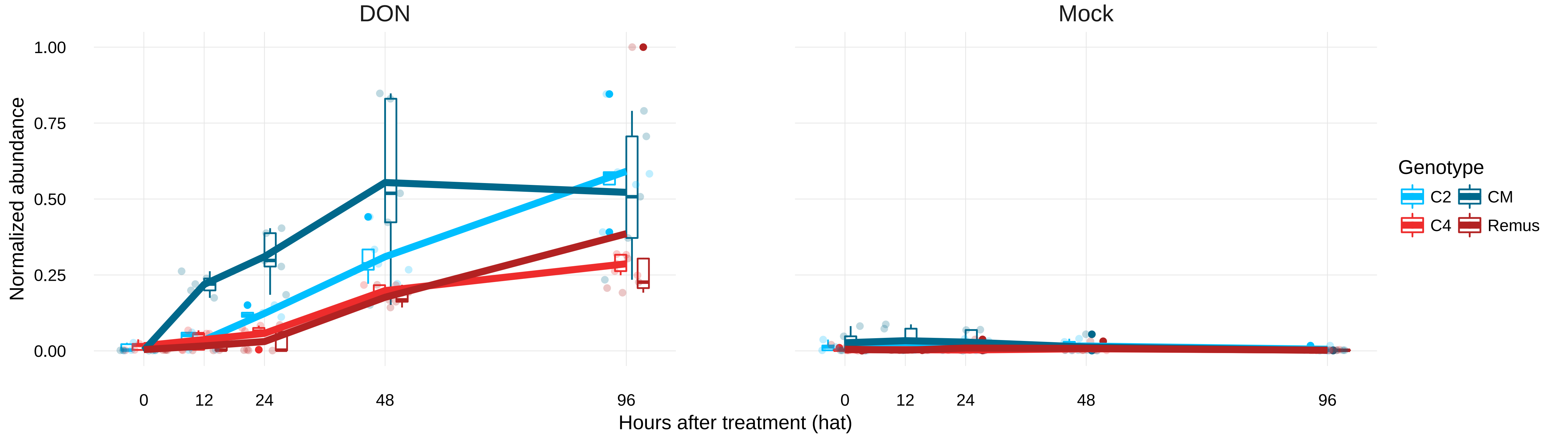

U.4

Unknown Phe−derived wheat−metabolite

MZ	163.0753
RT	12.31 min
Normalization	Directly via KPX samples
Cluster	Cluster 1B
Cn total / Phe	10 /

C2, C4; different treatments

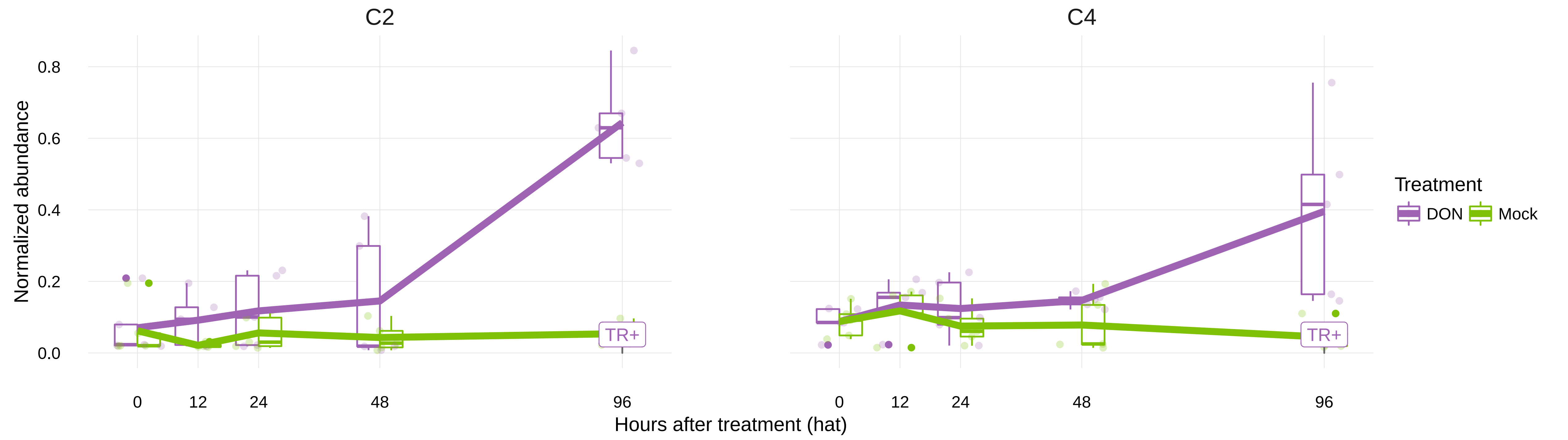

DON, Mock; different genotypes

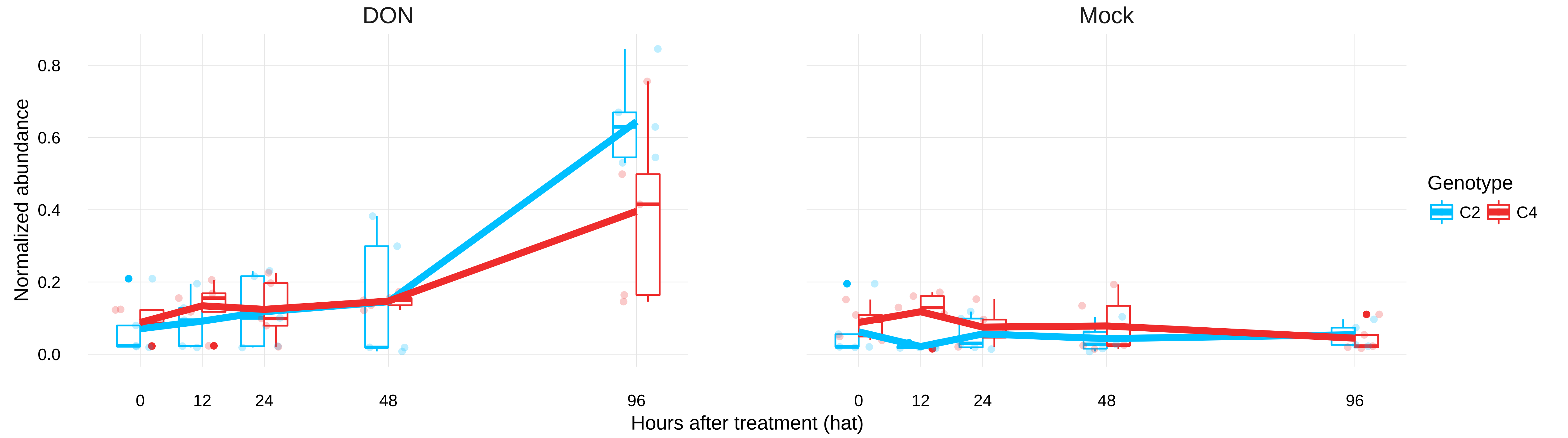

CM, Remus; different treatments

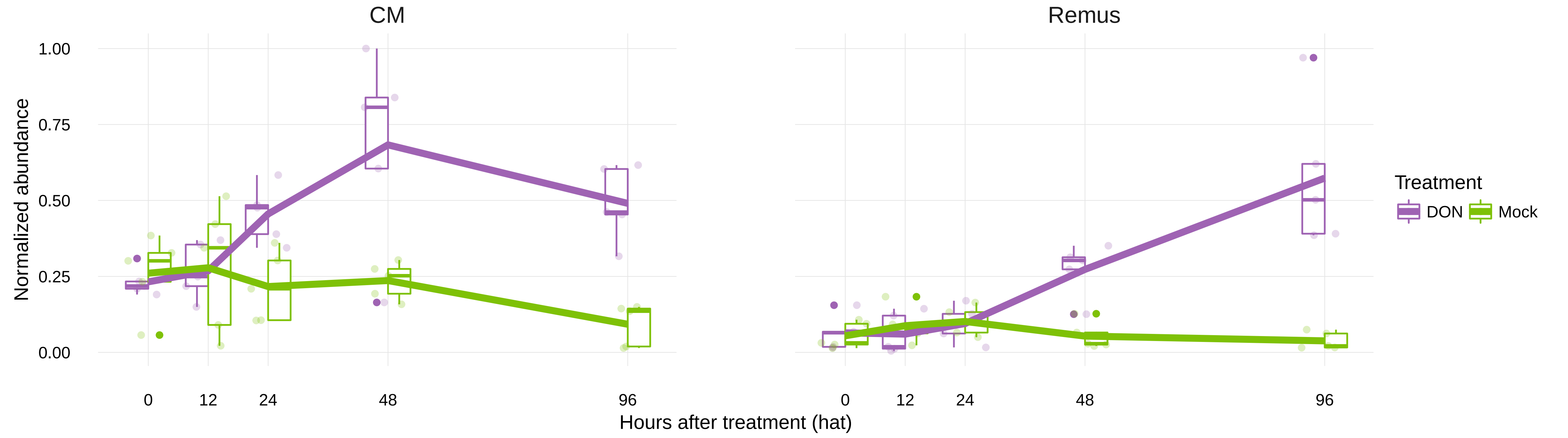

DON, Mock; all four genotypes

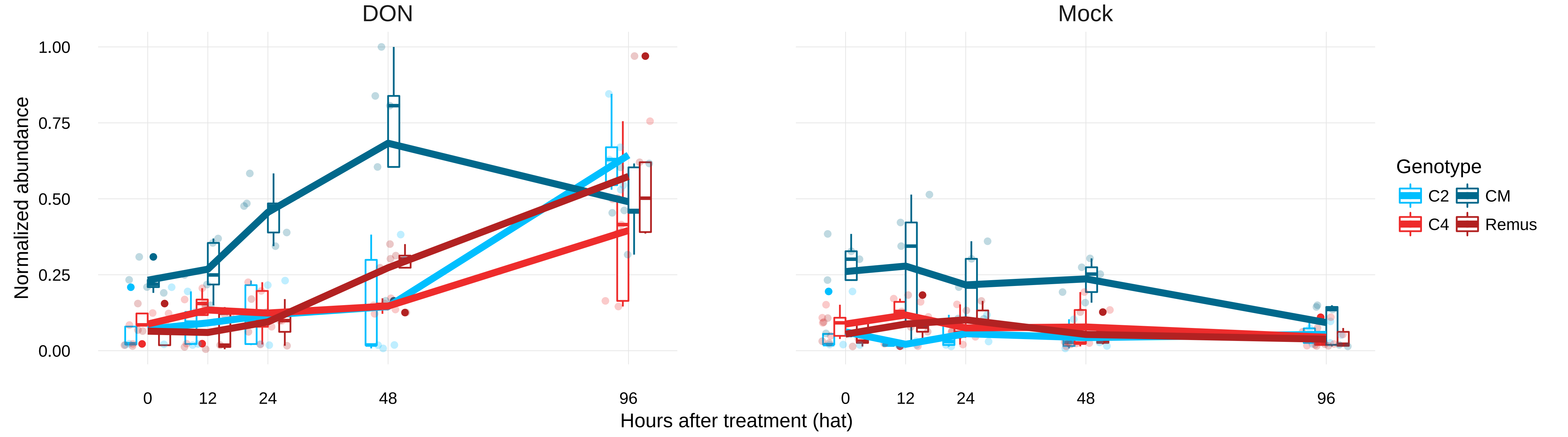

A.7

Annotated as HCAA
(2 database hits)

MZ	251.1392
RT	5.06 min
Normalization	Not normalized
Cluster	Cluster 1A
Cn total / Phe	13 /

C2, C4; different treatments

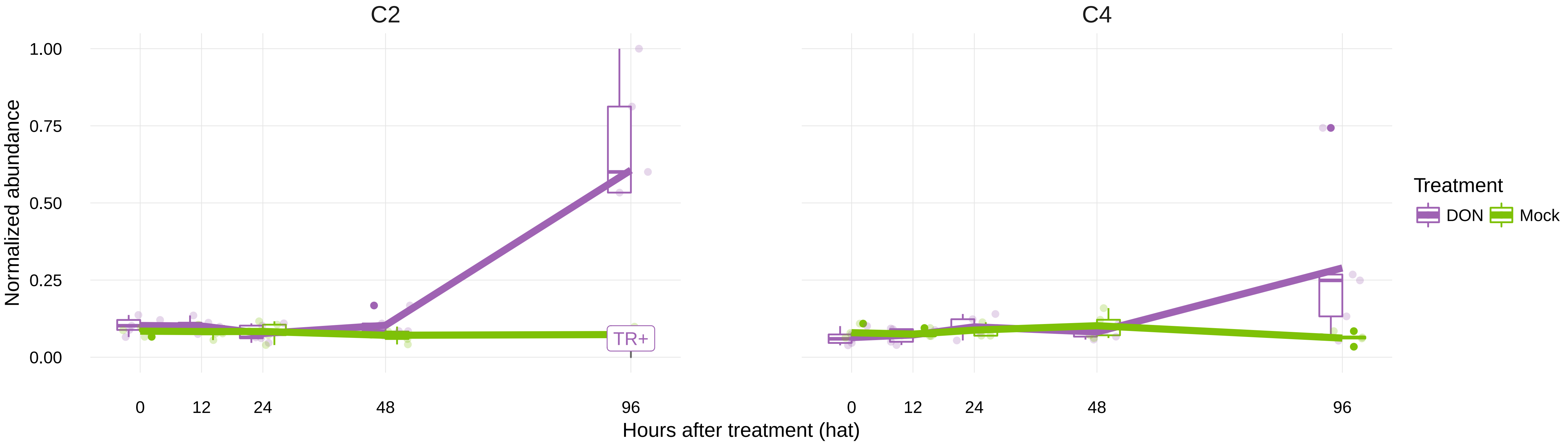

DON, Mock; different genotypes

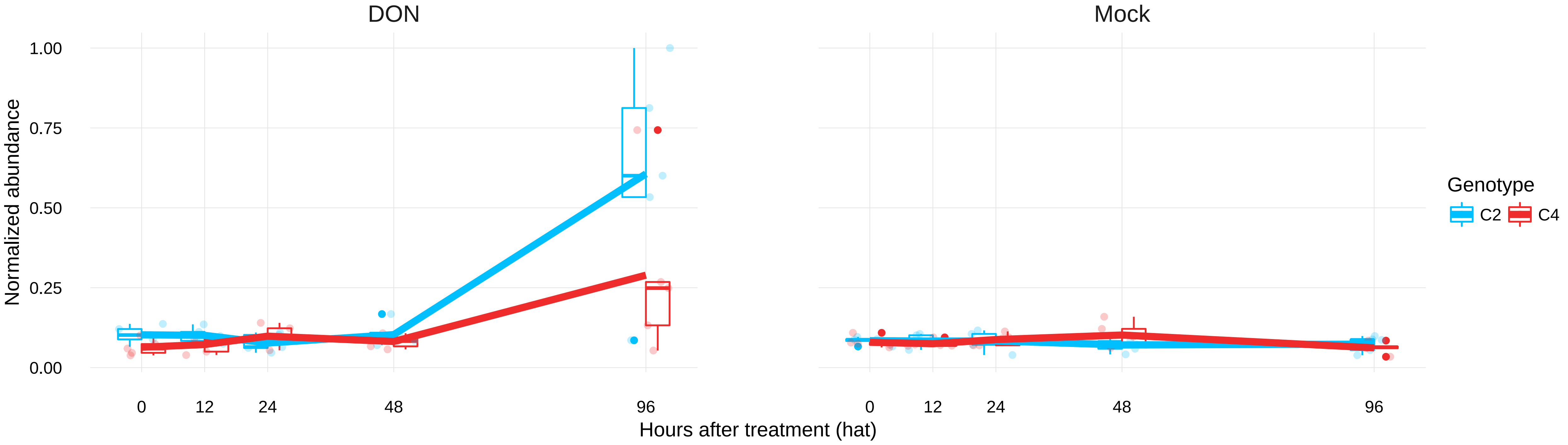

CM, Remus; different treatments

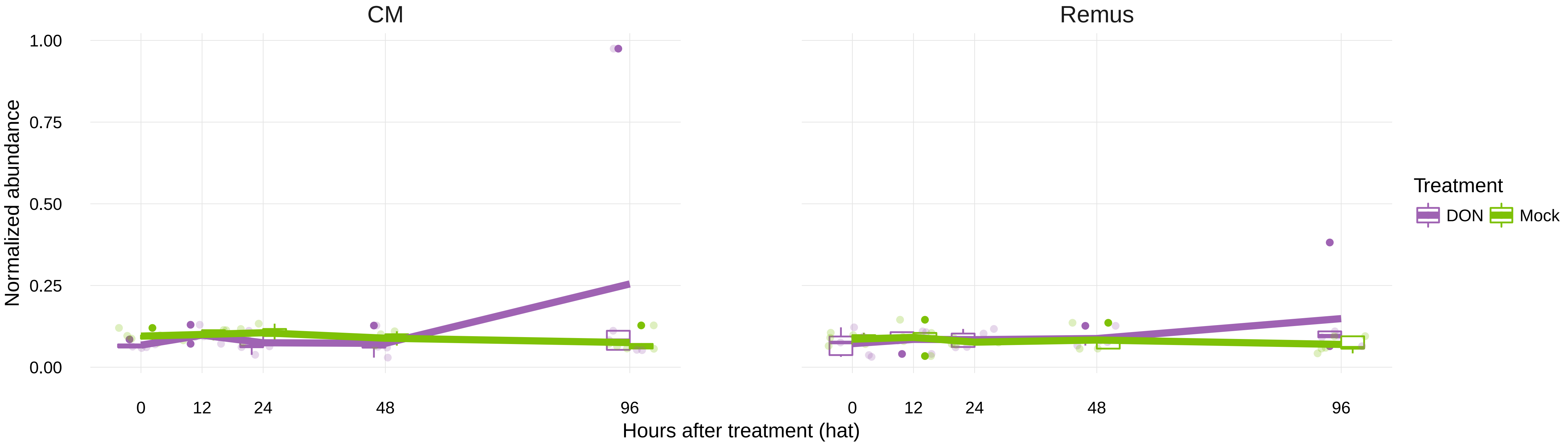

DON, Mock; all four genotypes

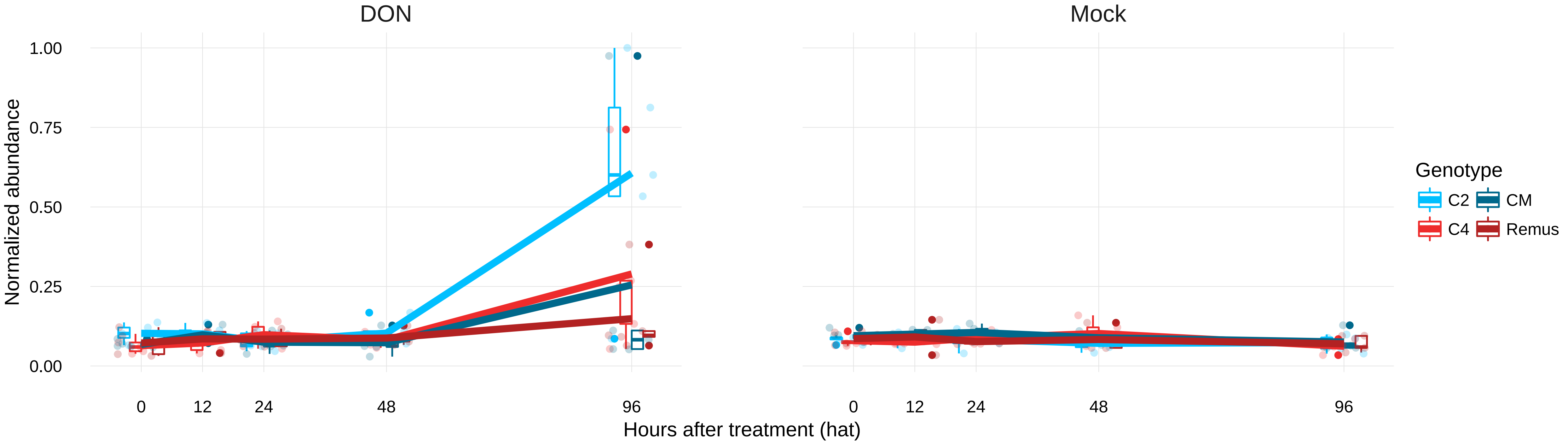

FerSer

Identified metabolite (level 1)

MZ	353.1498
RT	16.43 min
Normalization	Directly via KPX samples
Cluster	Cluster 1A
Cn total / Phe	20 / 9

C2, C4; different treatments

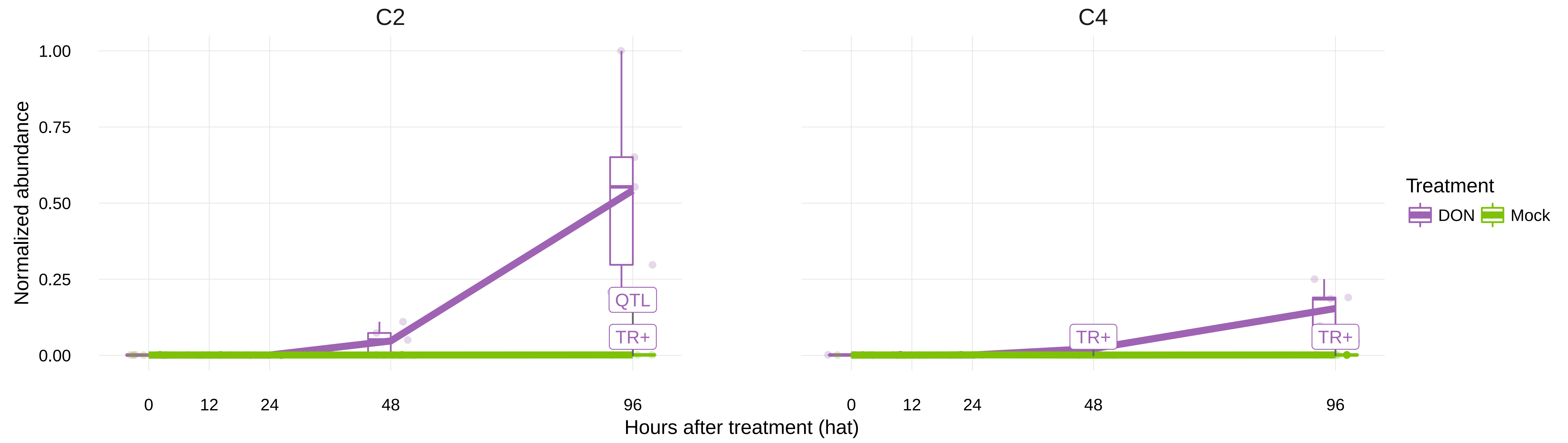

DON, Mock; different genotypes

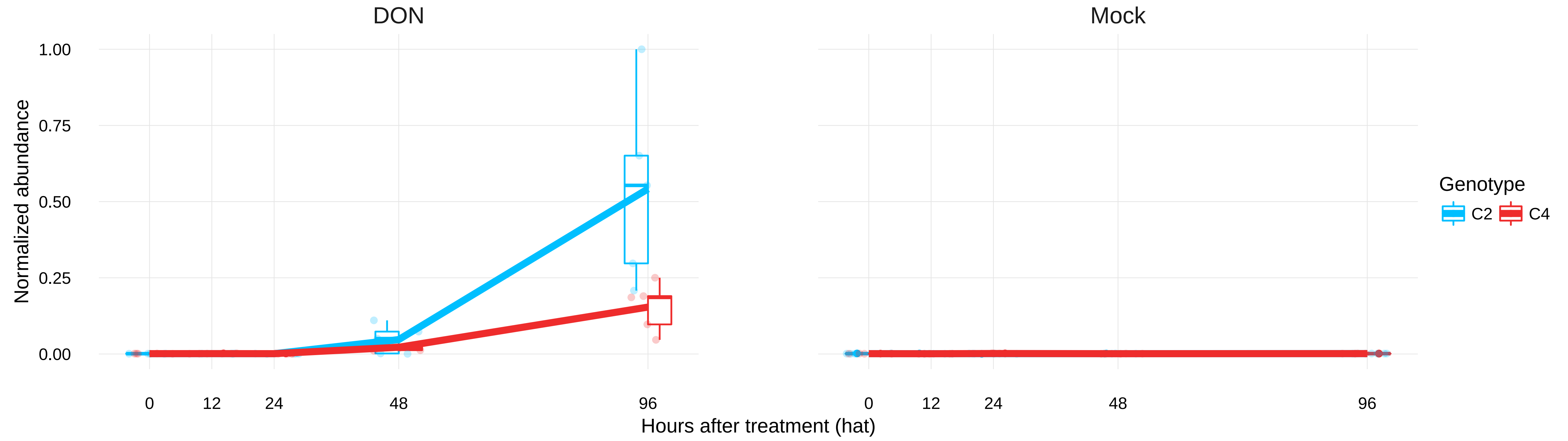

CM, Remus; different treatments

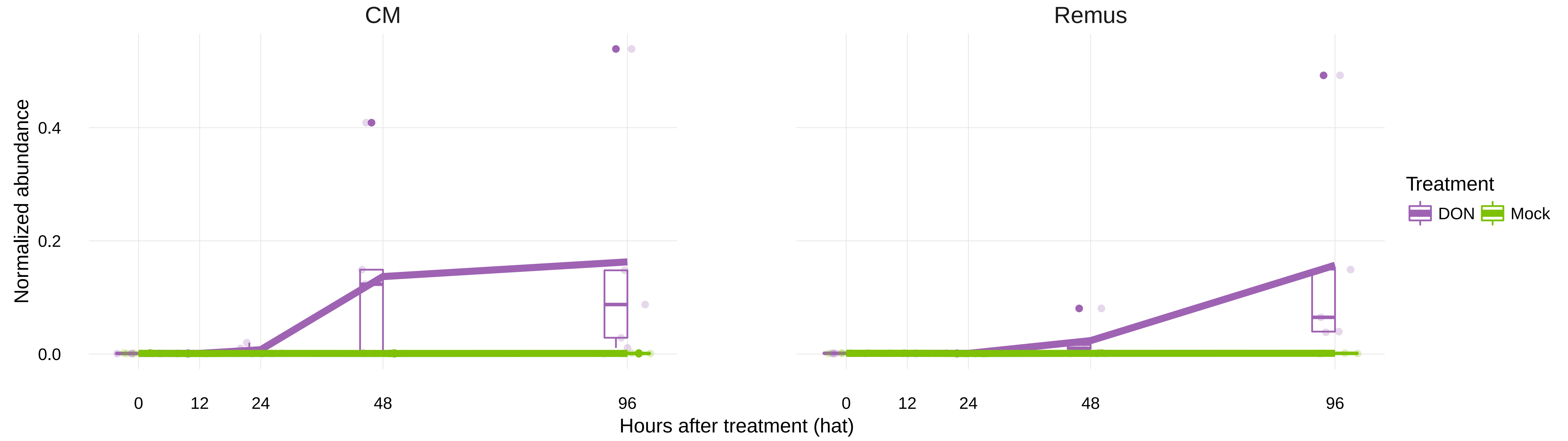

DON, Mock; all four genotypes

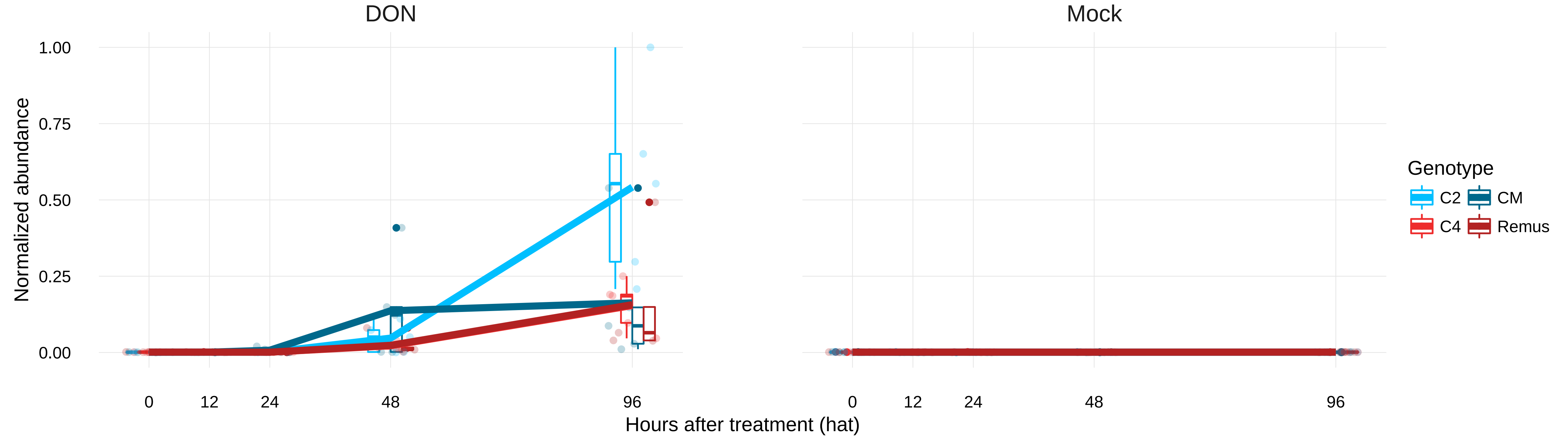

A.118

Annotated as HCAA (diHCAA)
(4 database hits)

MZ	441.202
RT	18.66 min
Normalization	Indirectly via surrogate in the KPX samples
Cluster	Cluster 1A
Cn total / Phe	24 / 18

C2, C4; different treatments

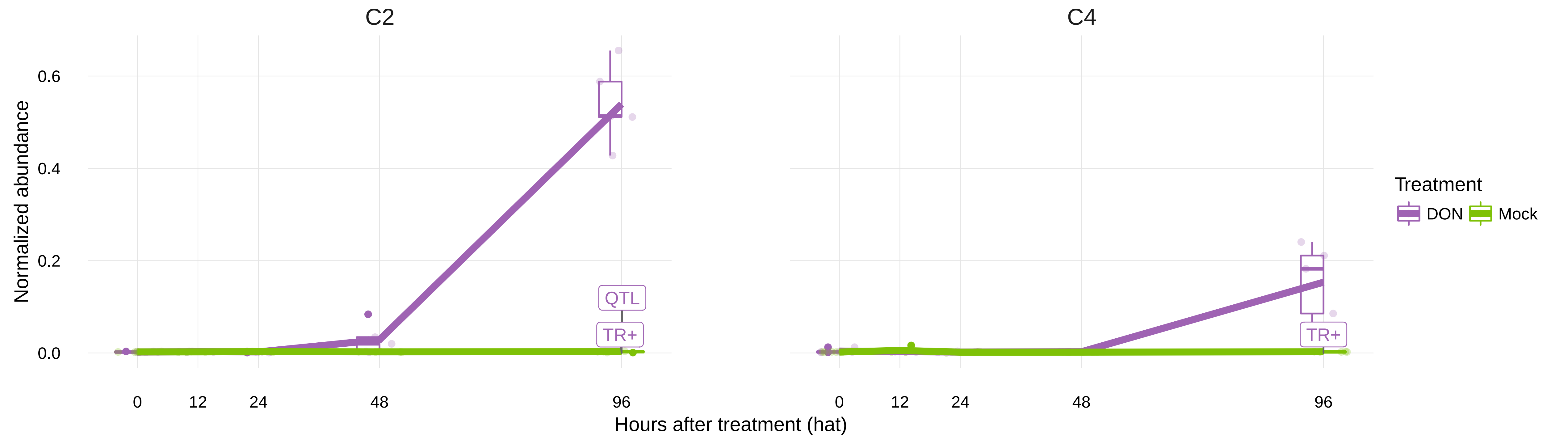

DON, Mock; different genotypes

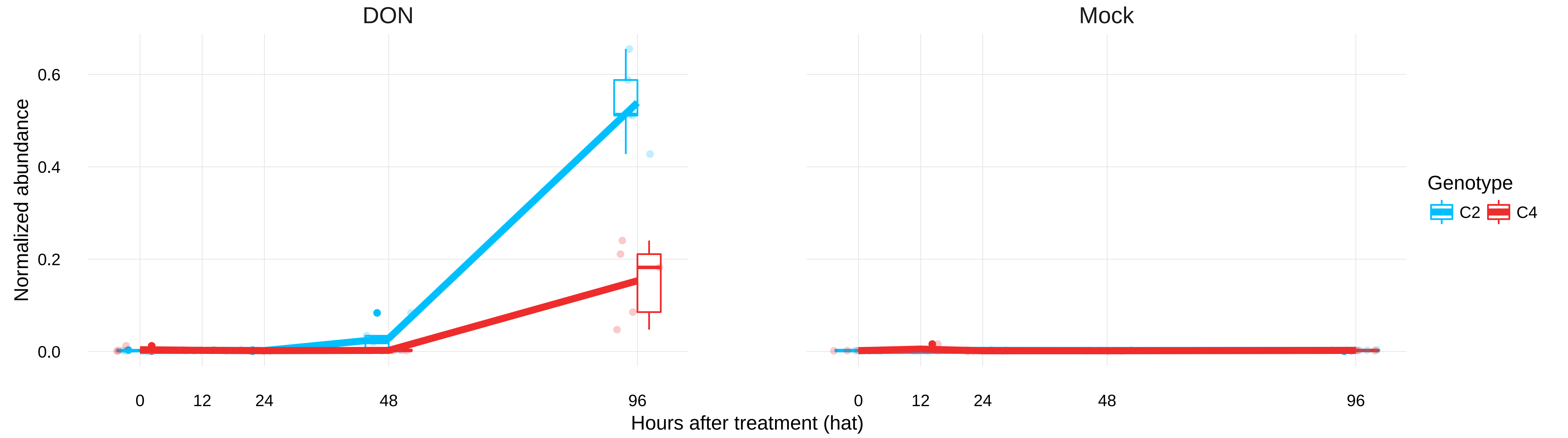

CM, Remus; different treatments

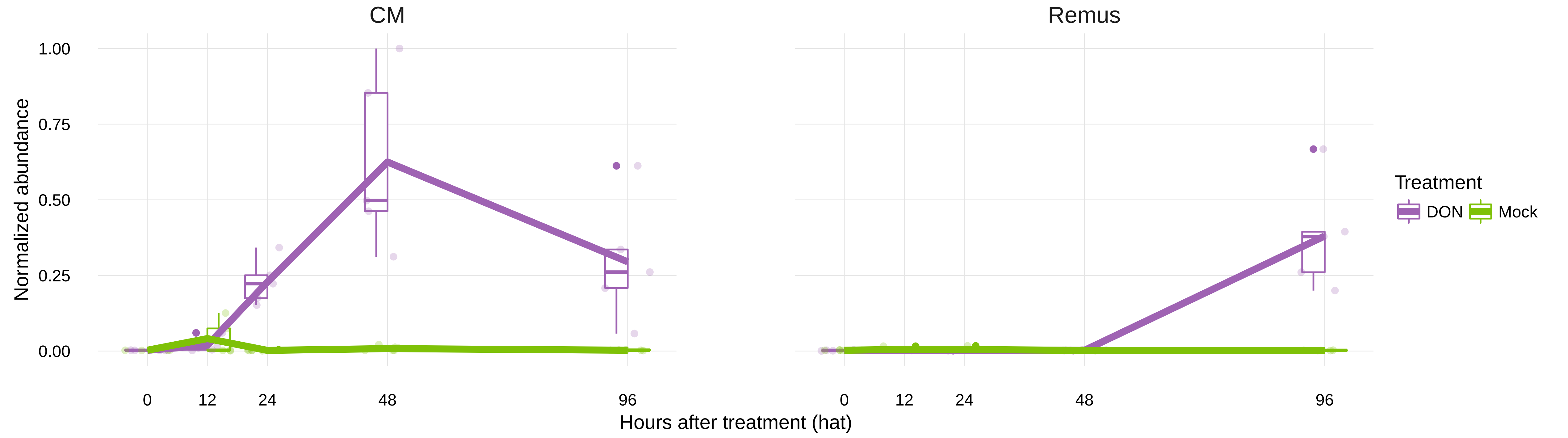

DON, Mock; all four genotypes

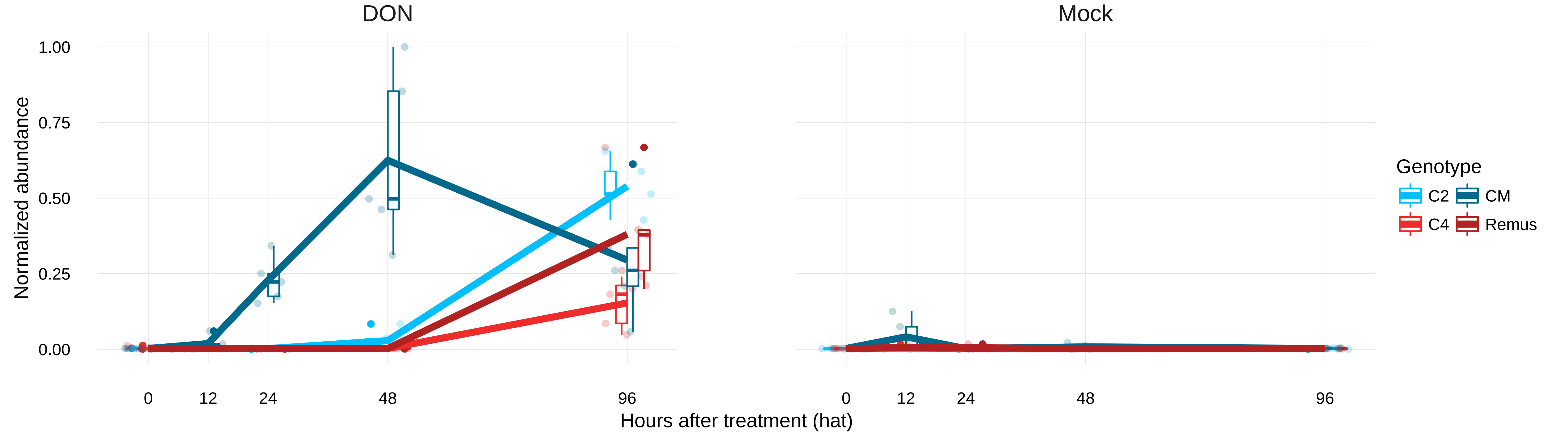

U.11

Unknown Phe−derived wheat−metabolite

MZ	427.1865
RT	16.92 min
Normalization	Indirectly via surrogate in the KPX samples
Cluster	Cluster 1A
Cn total / Phe	28 / 9

C2, C4; different treatments

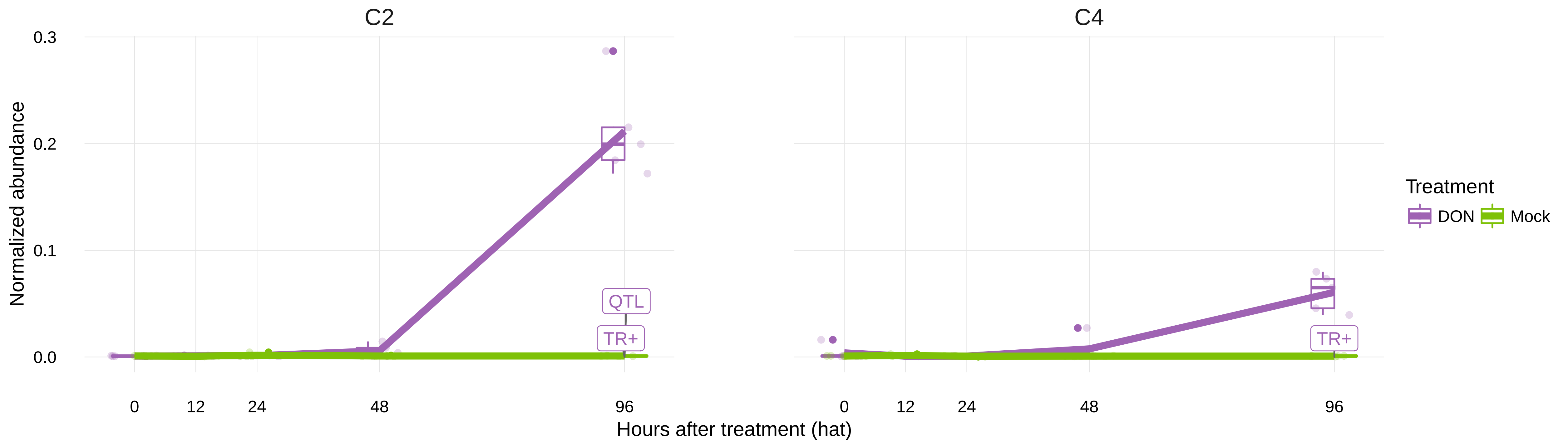

DON, Mock; different genotypes

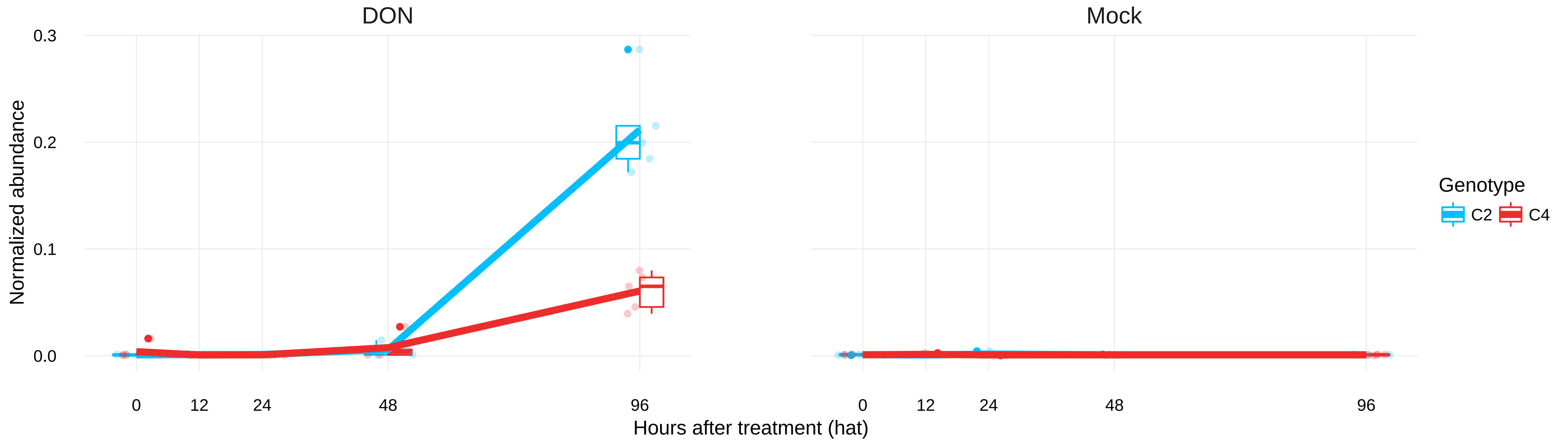

CM, Remus; different treatments

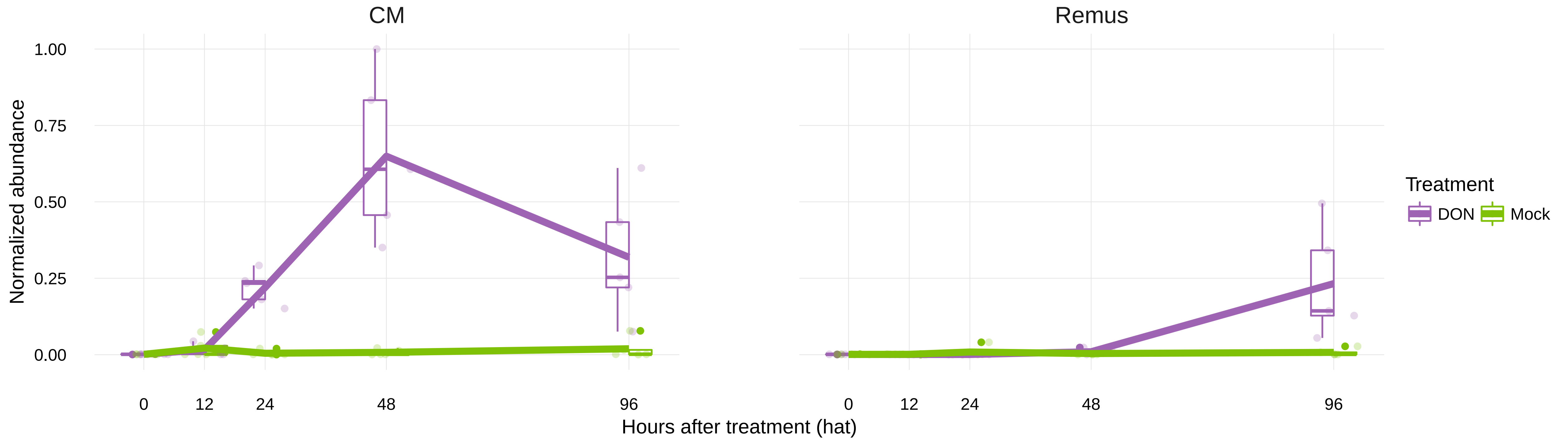

DON, Mock; all four genotypes

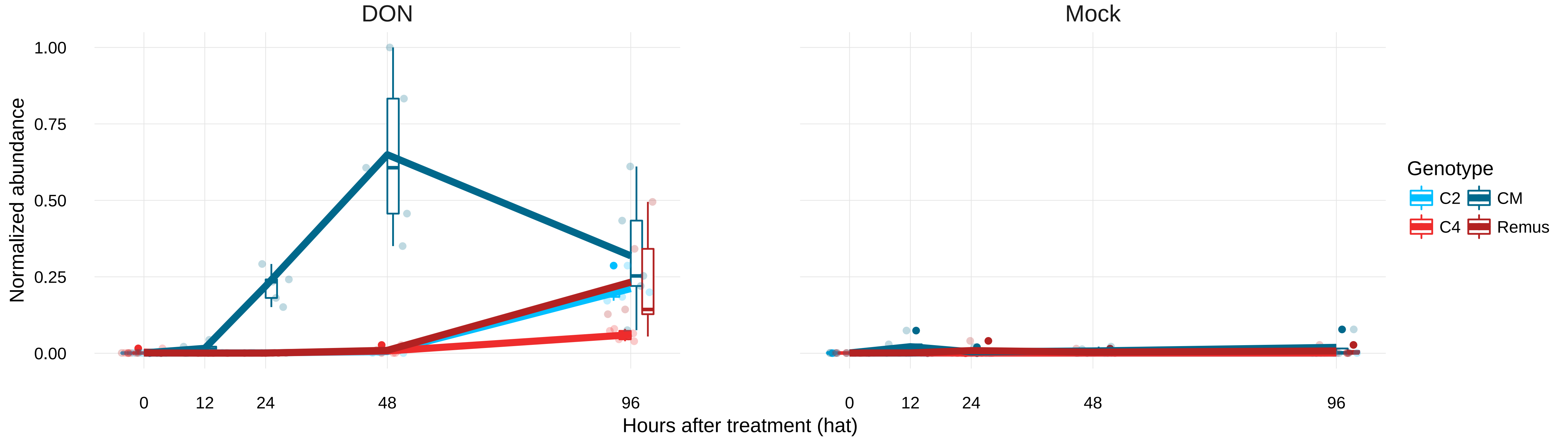

A.116

Annotated as HCAA (diHCAA)
(2 database hits)

MZ	411.1916
RT	18.49 min
Normalization	Directly via KPX samples
Cluster	Cluster 1A
Cn total / Phe	23 / 18

C2, C4; different treatments

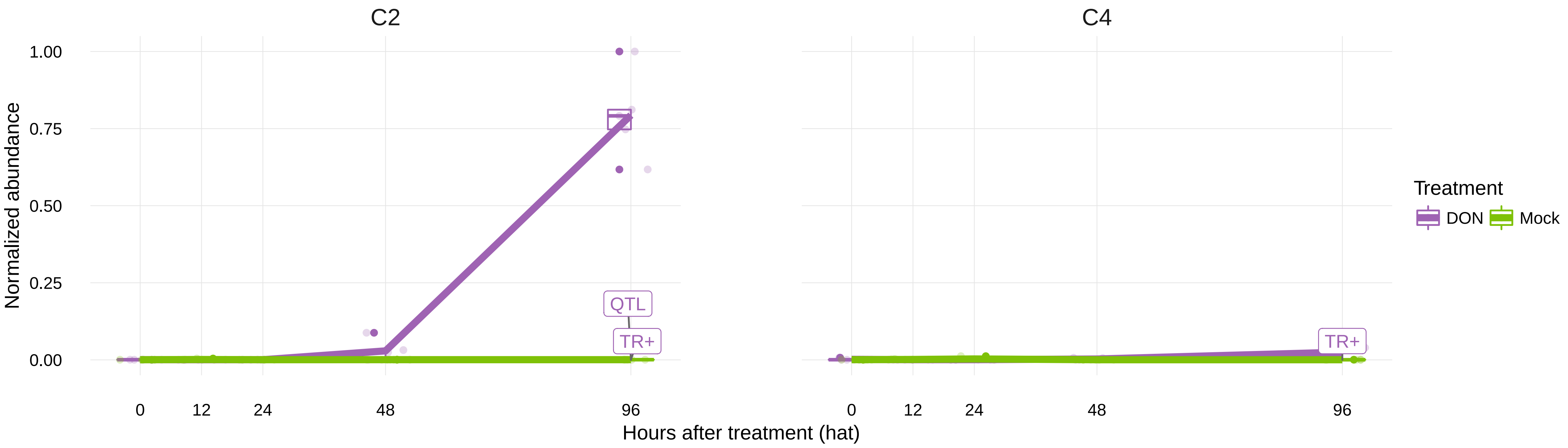

DON, Mock; different genotypes

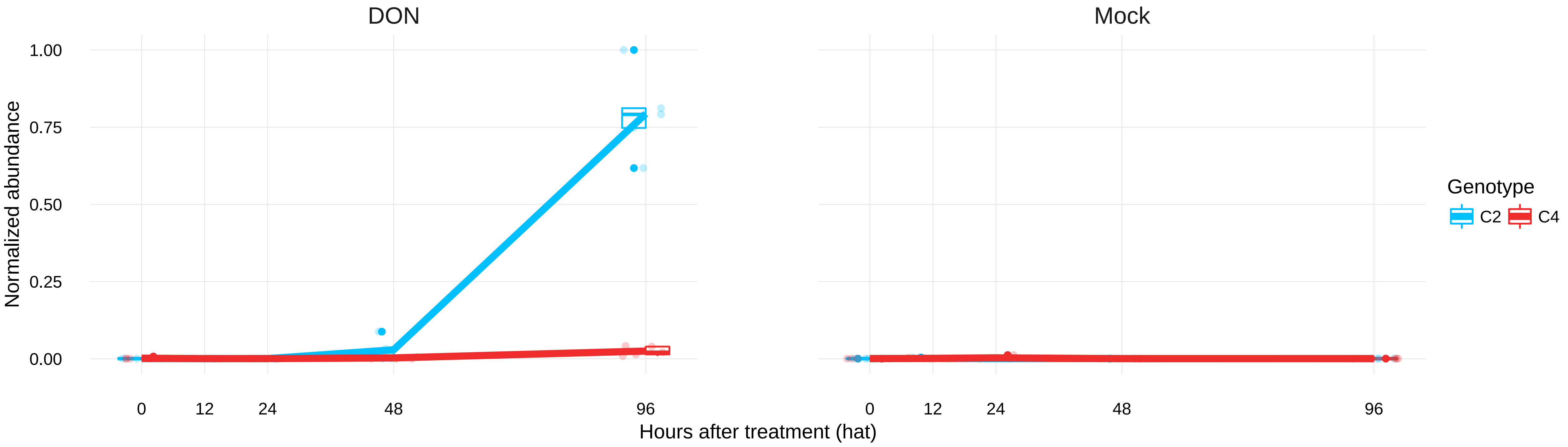

CM, Remus; different treatments

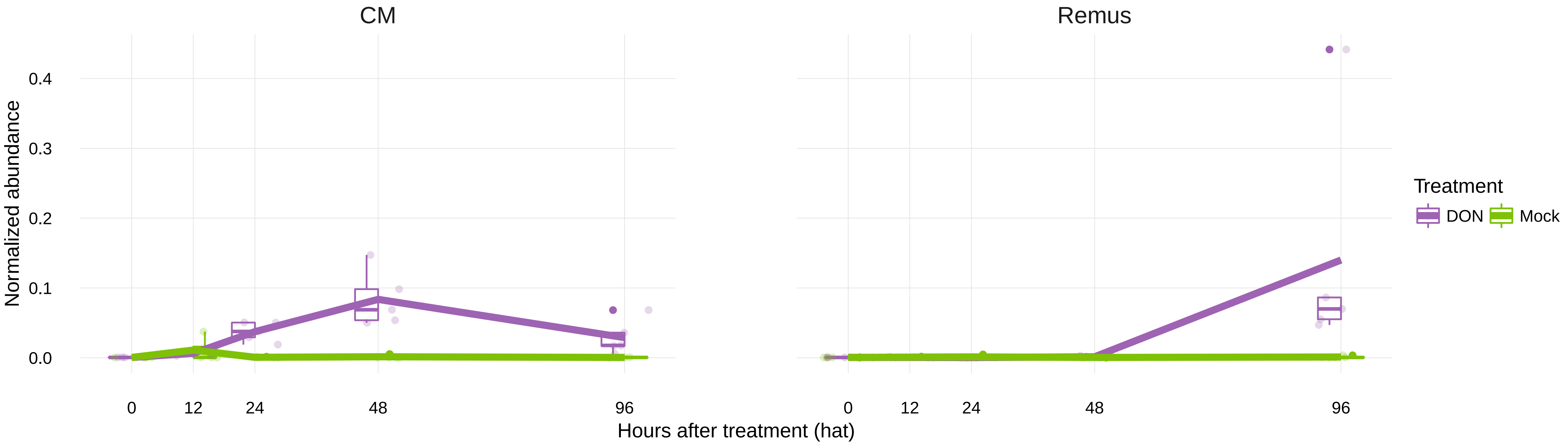

DON, Mock; all four genotypes

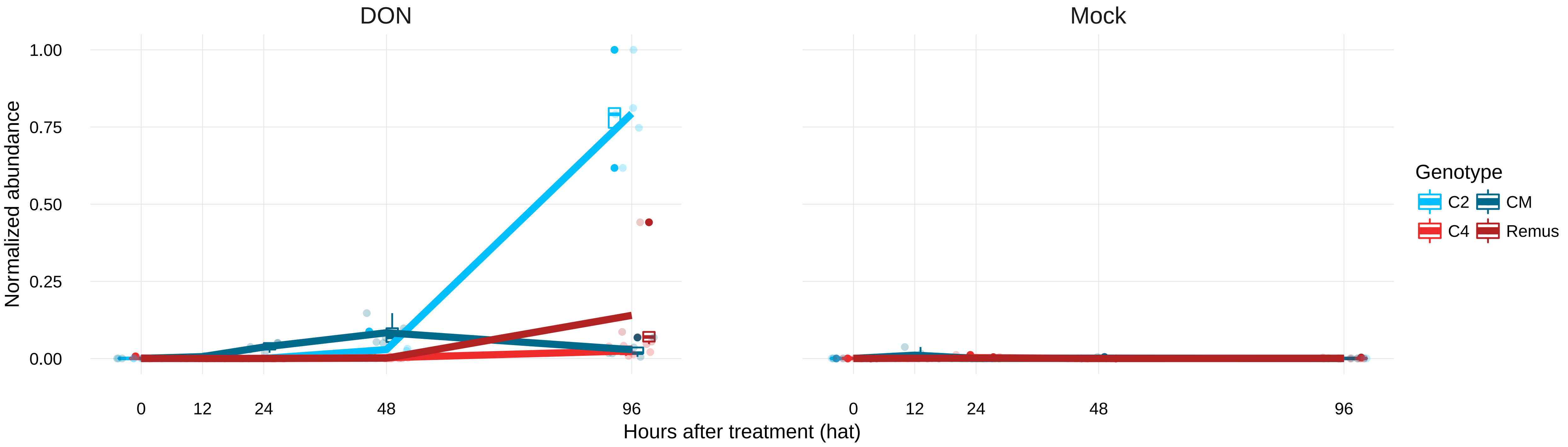

A.16

Annotated as HCAA
(2 database hits)

MZ	251.1393
RT	6.95 min
Normalization	Directly via KPX samples
Cluster	Cluster 1A
Cn total / Phe	13 / 9

C2, C4; different treatments

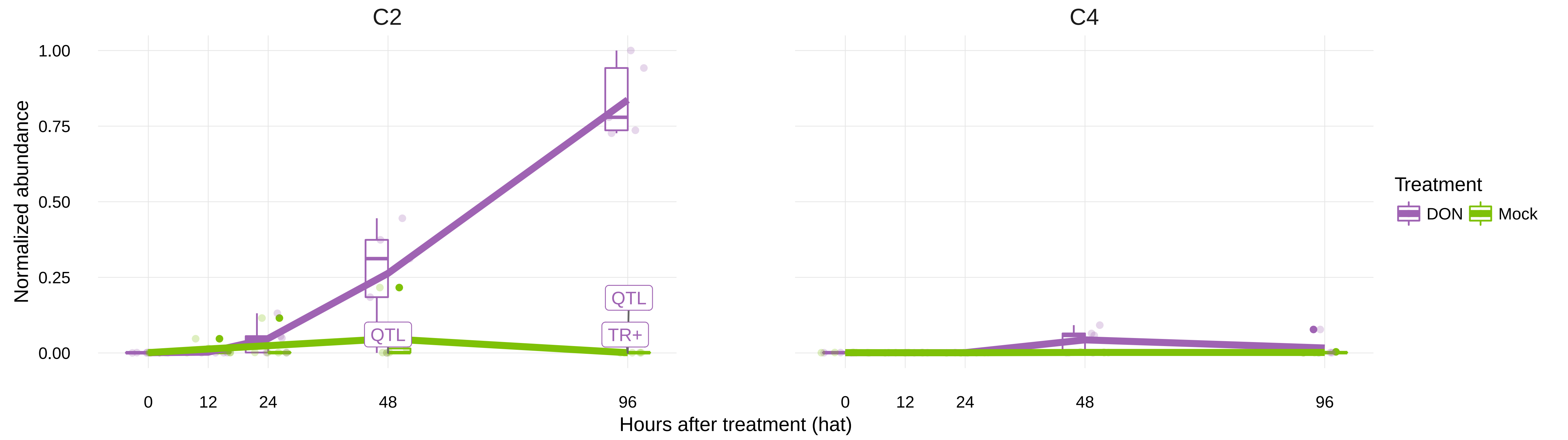

DON, Mock; different genotypes

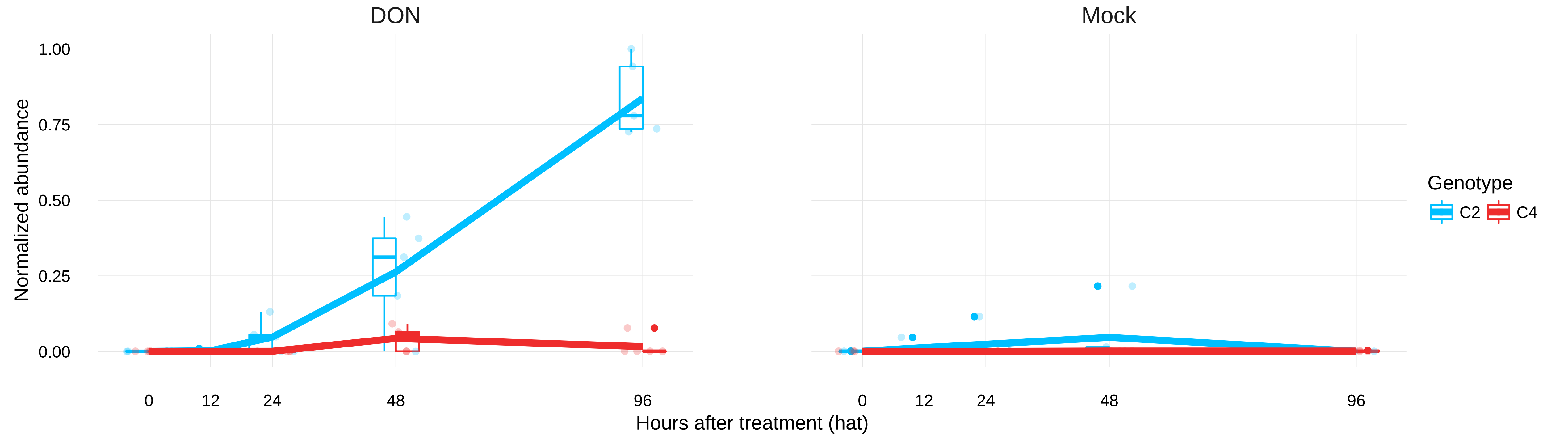

CM, Remus; different treatments

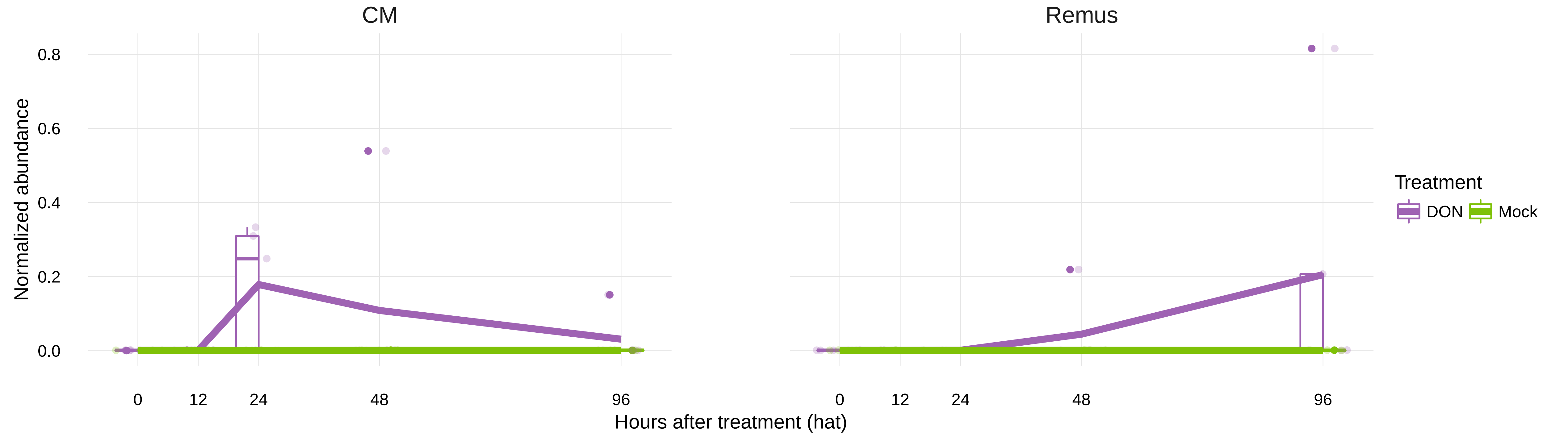

DON, Mock; all four genotypes

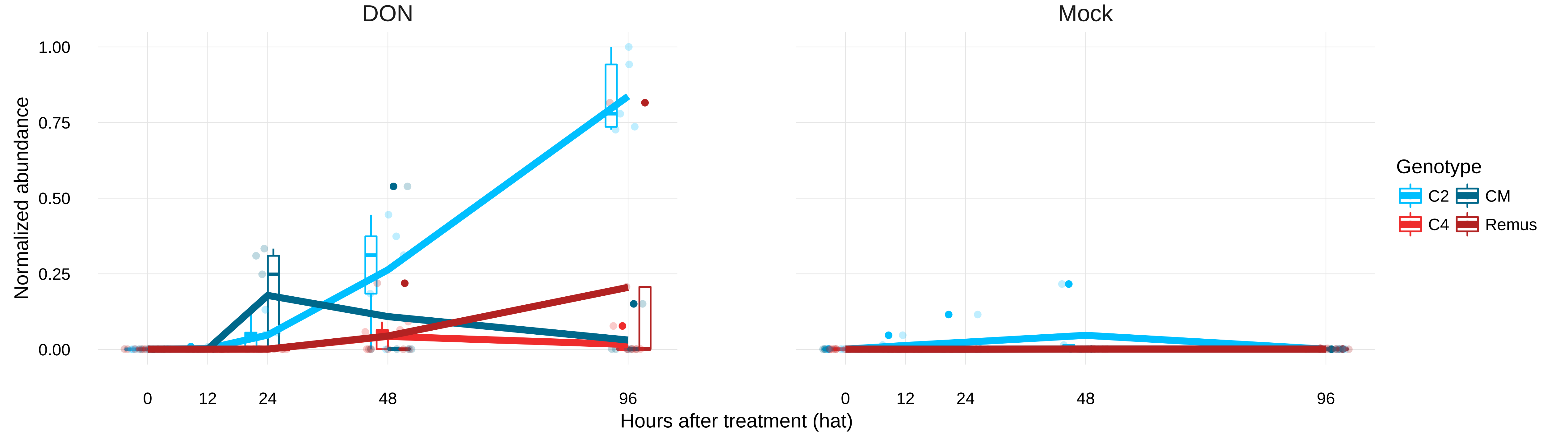

A.115

Annotated as HCAA (diHCAA)
(1 database hit)

MZ	381.181
RT	18.3 min
Normalization	Indirectly via surrogate in the KPX samples
Cluster	Cluster 1A
Cn total / Phe	22 /

C2, C4; different treatments

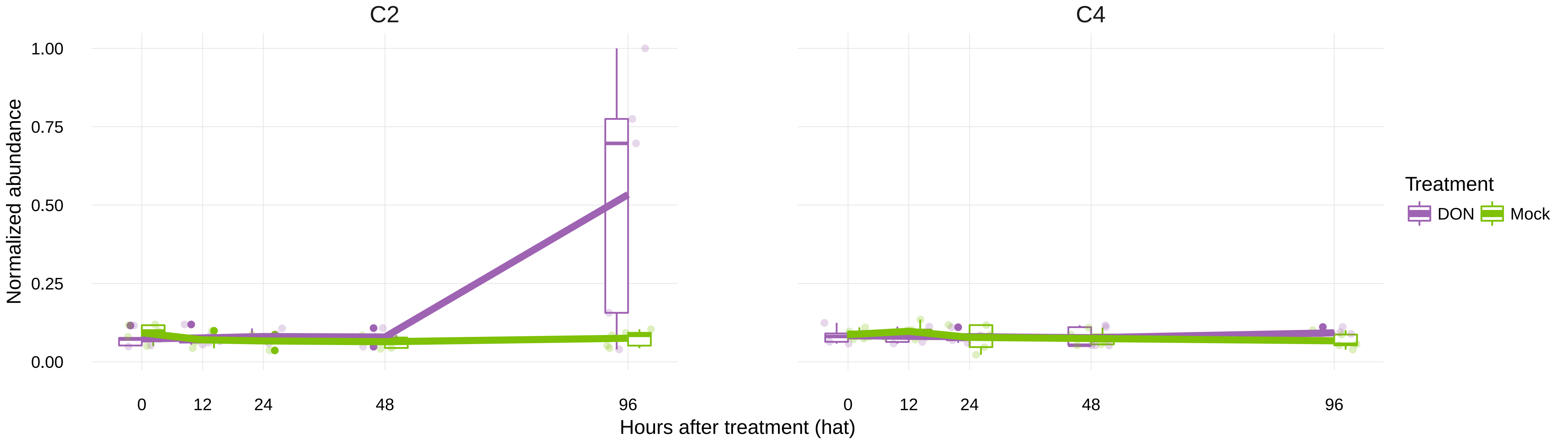

DON, Mock; different genotypes

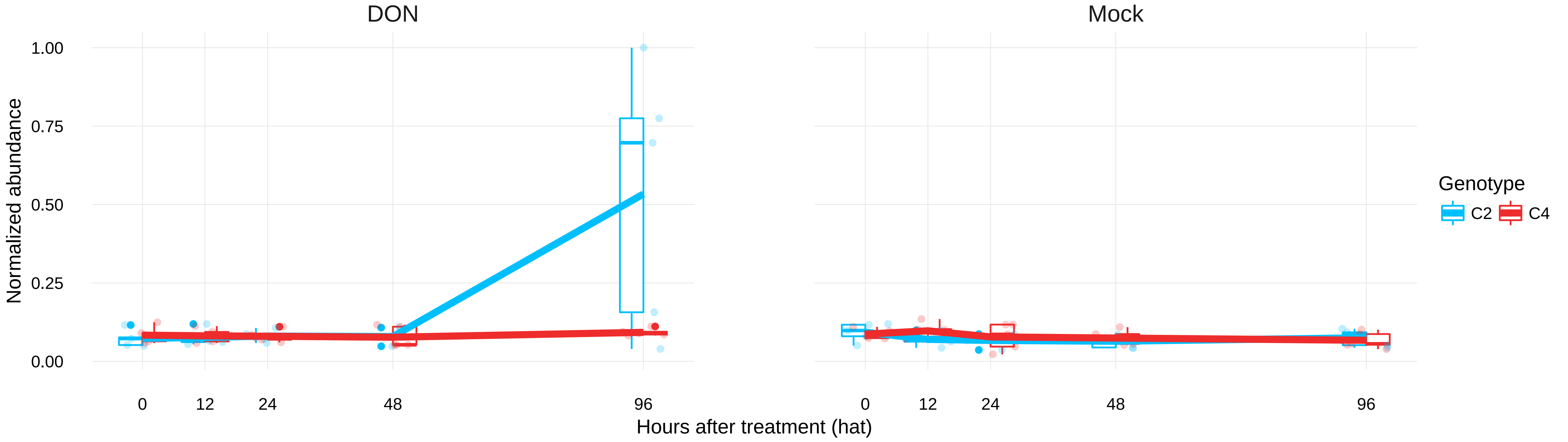

CM, Remus; different treatments

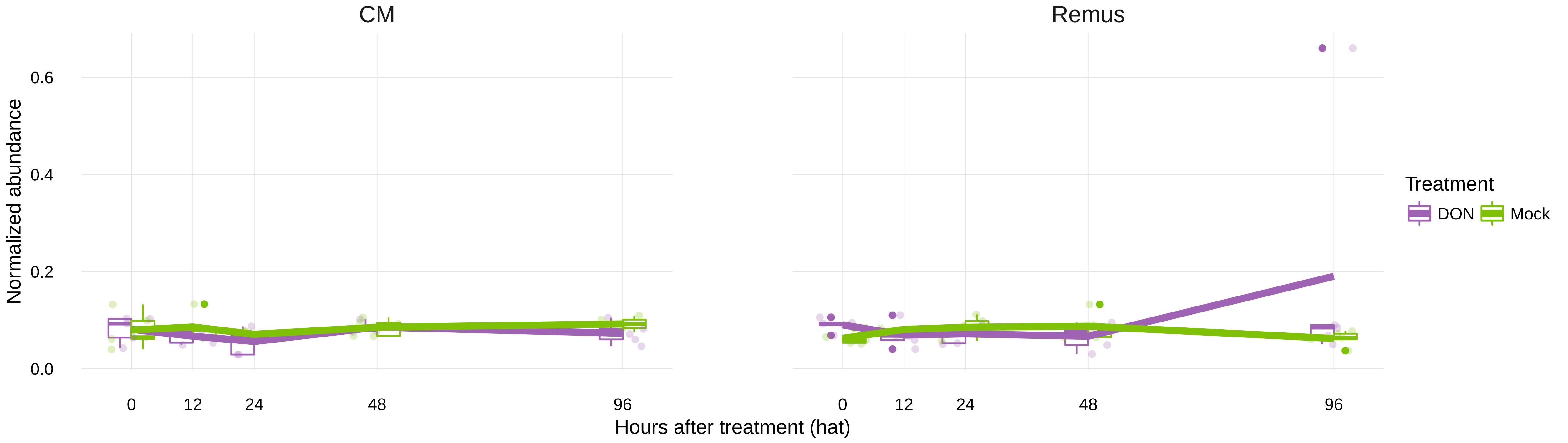

DON, Mock; all four genotypes

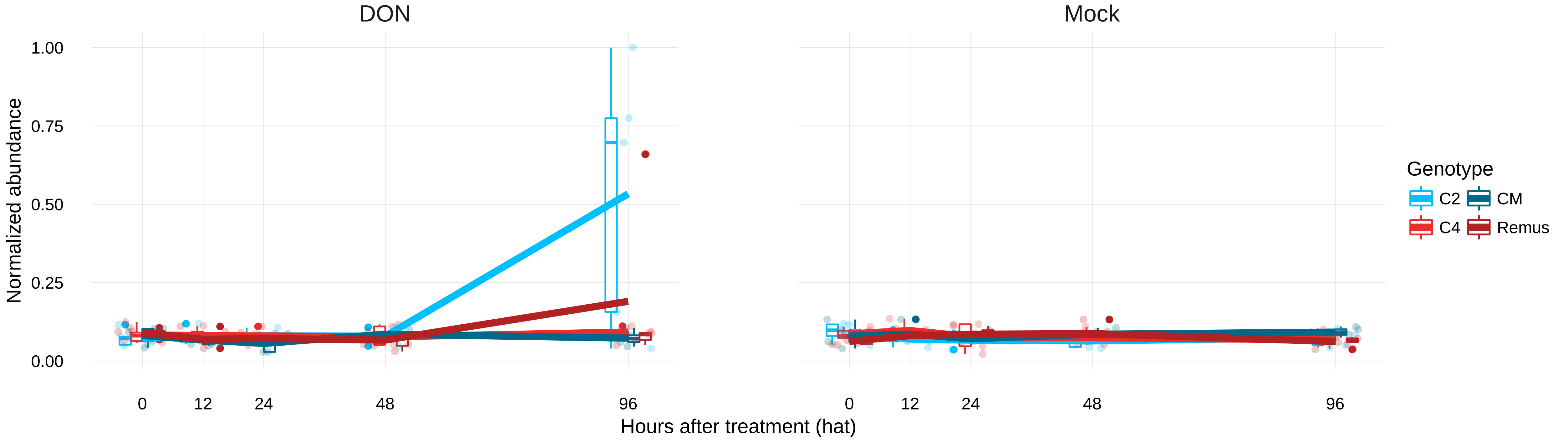

A.25

Annotated as others (dipeptide)
(1 database hit)

MZ	281.1139
RT	8.84 min
Normalization	Indirectly via surrogate in the KPX samples
Cluster	Cluster 1A
Cn total / Phe	9 / 9

C2, C4; different treatments

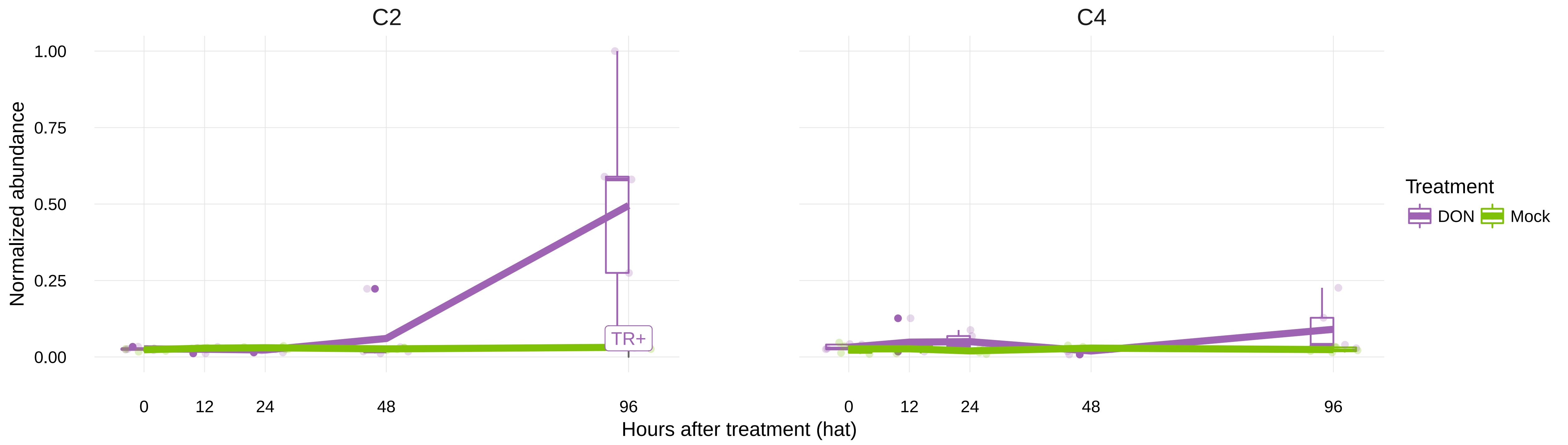

DON, Mock; different genotypes

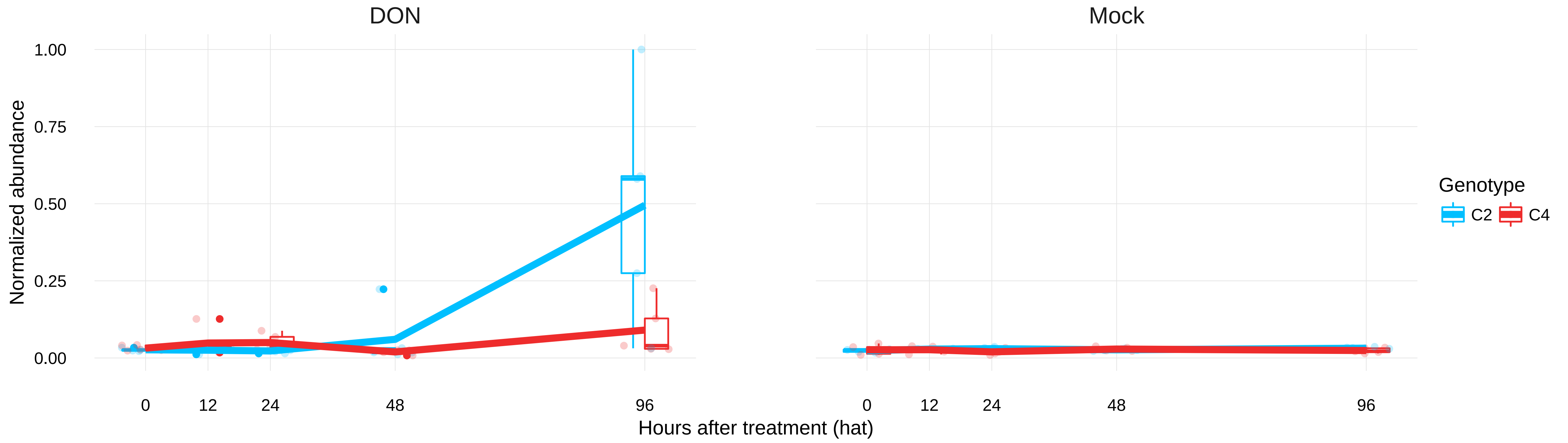

CM, Remus; different treatments

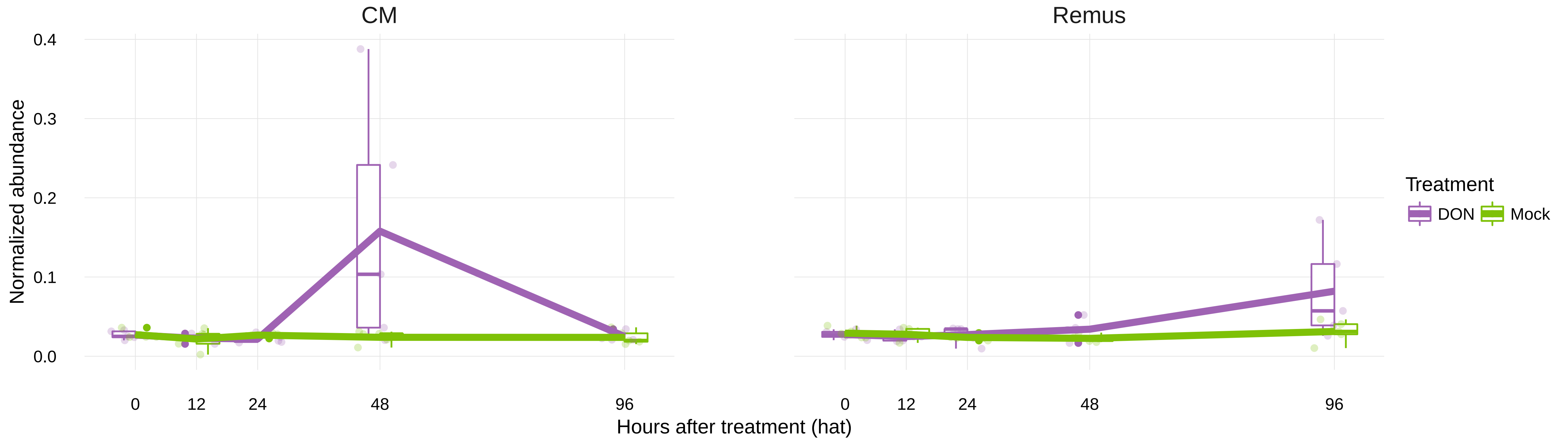

DON, Mock; all four genotypes

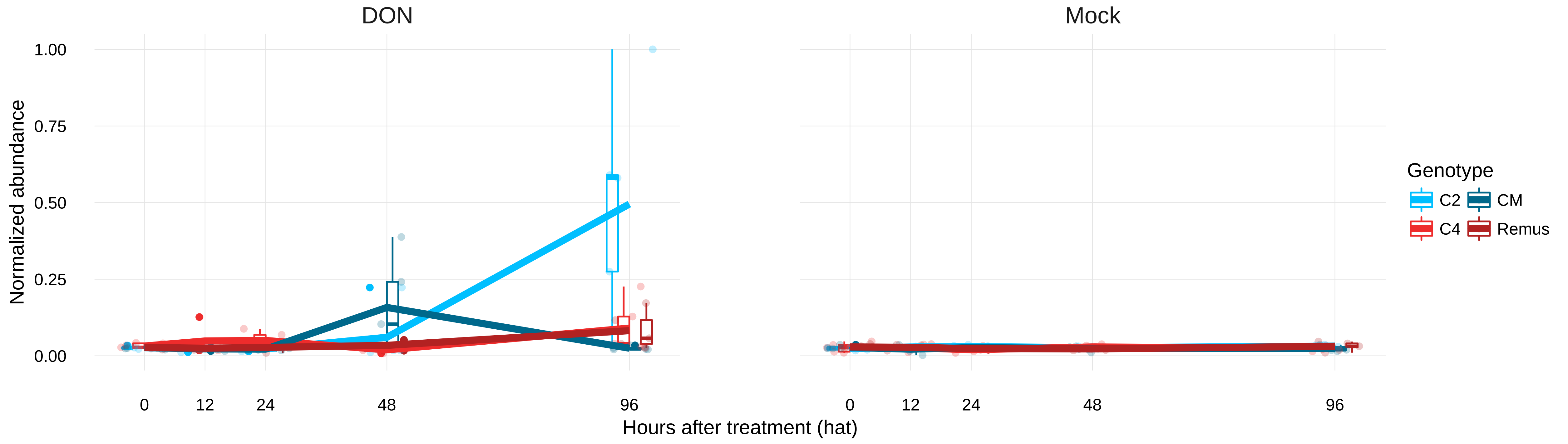

U.13

Unknown Phe−derived wheat−metabolite

MZ	147.0442
RT	21.22 min
Normalization	Directly via KPX samples
Cluster	−
Cn total / Phe	9 / 9

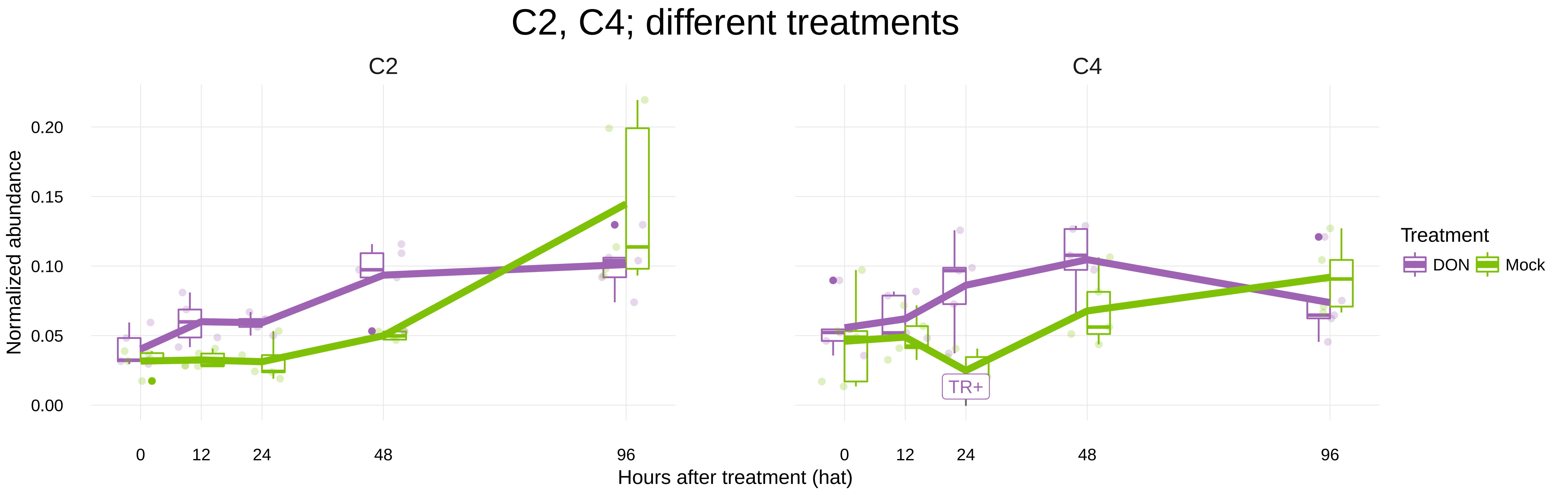

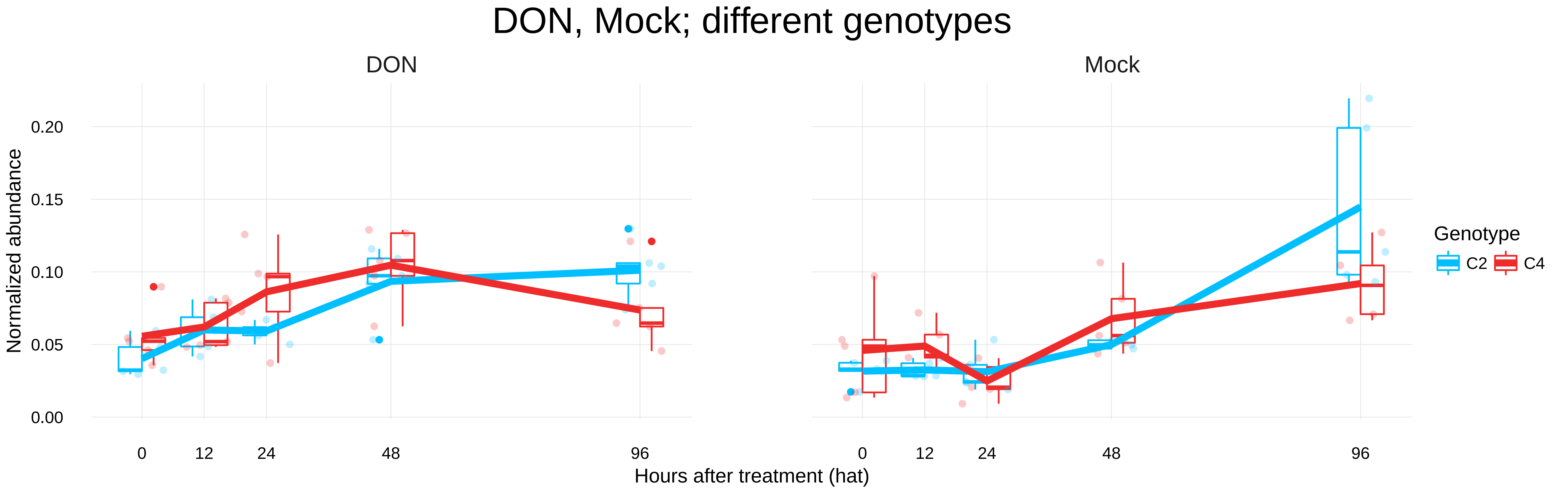

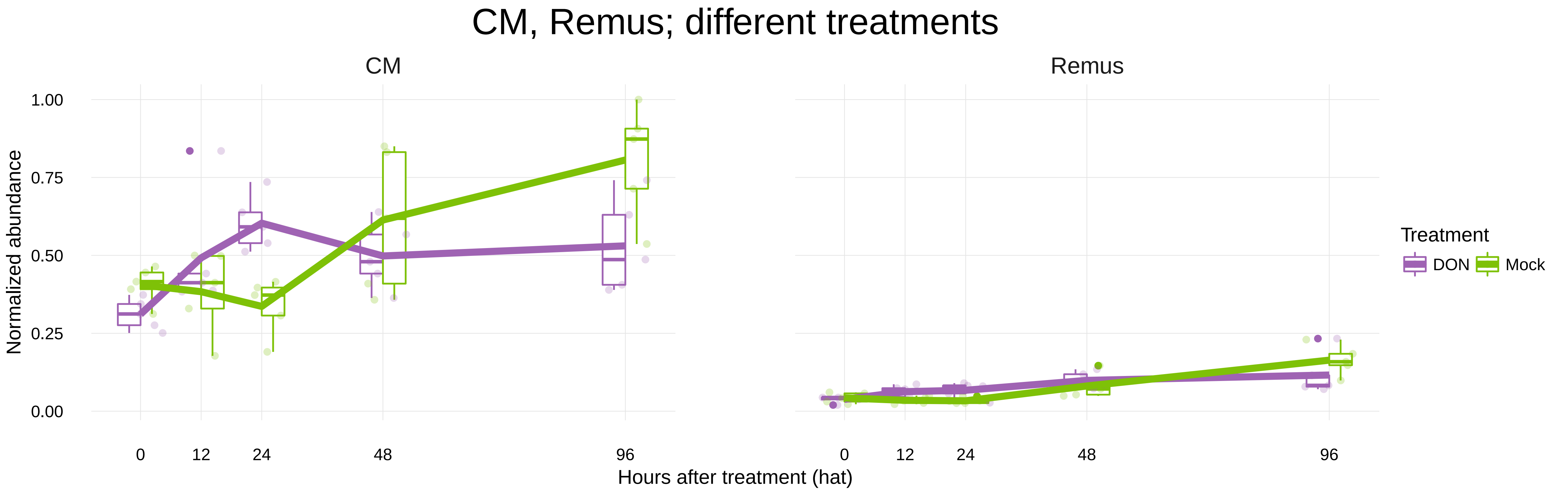

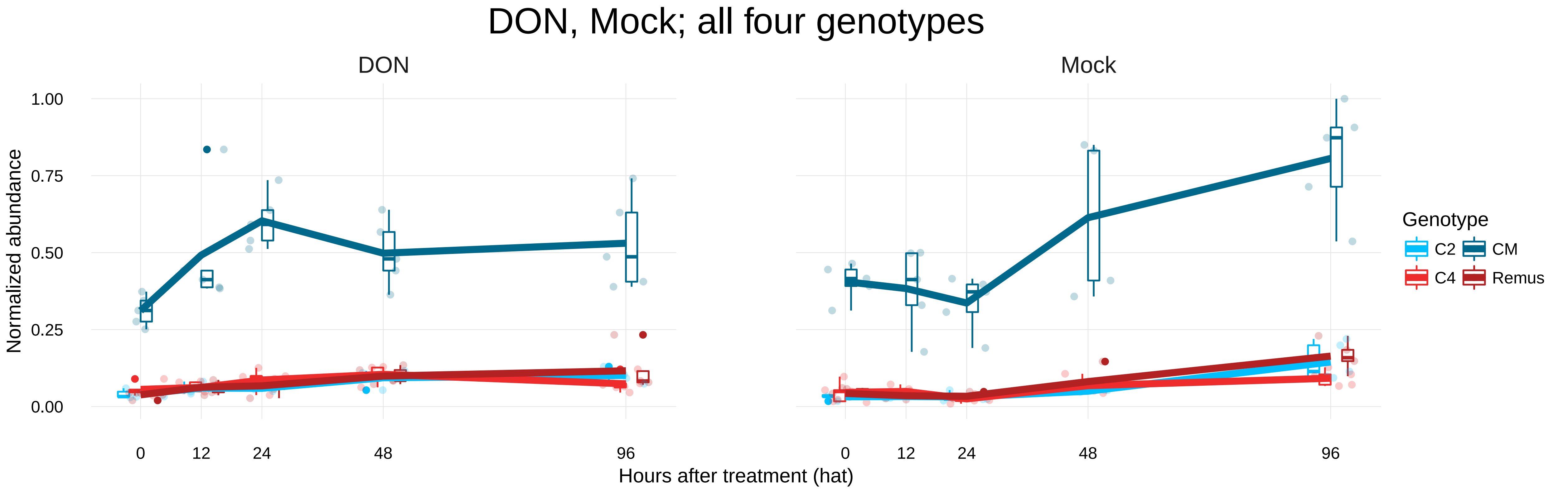

A.125

Annotated as Lignan or Flavonoid
(3 database hits)

MZ	417.1545
RT	20.35 min
Normalization	Directly via KPX samples
Cluster	–
Cn total / Phe	22 /

C2, C4; different treatments

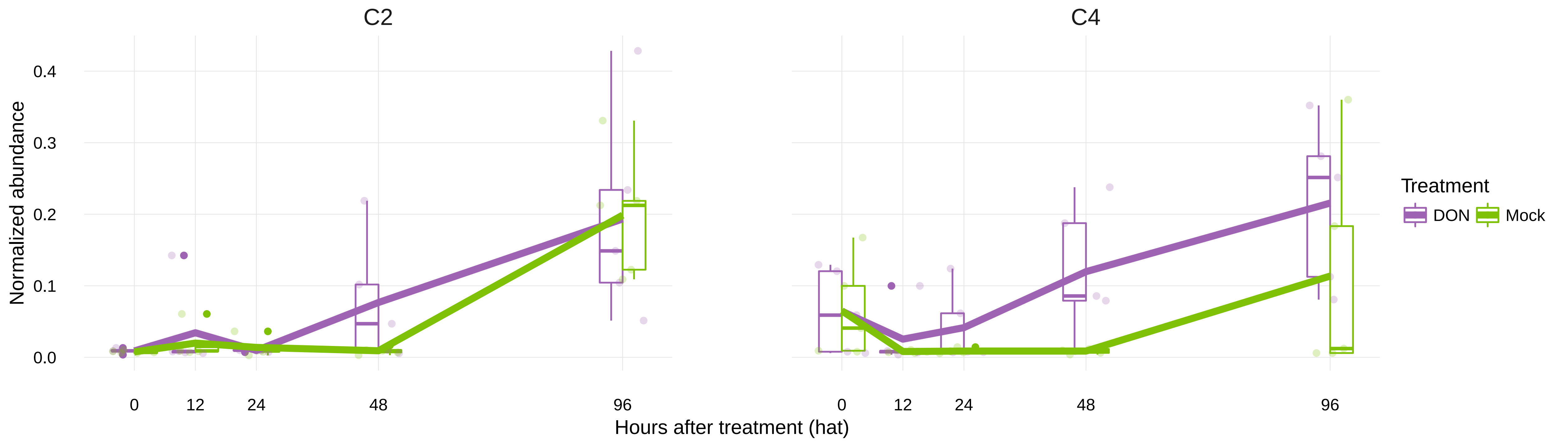

DON, Mock; different genotypes

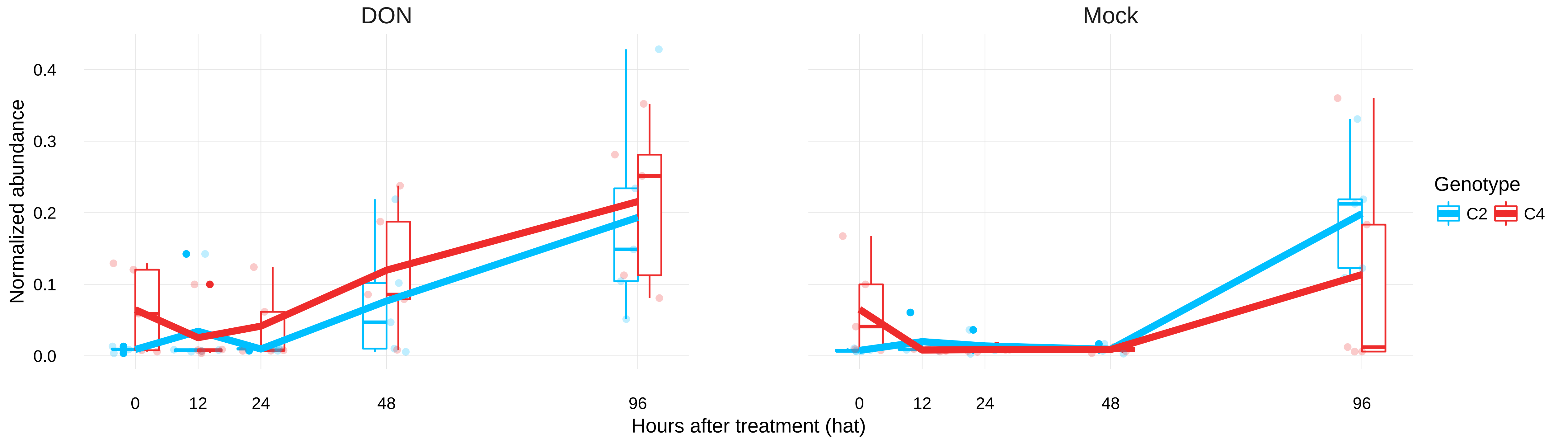

CM, Remus; different treatments

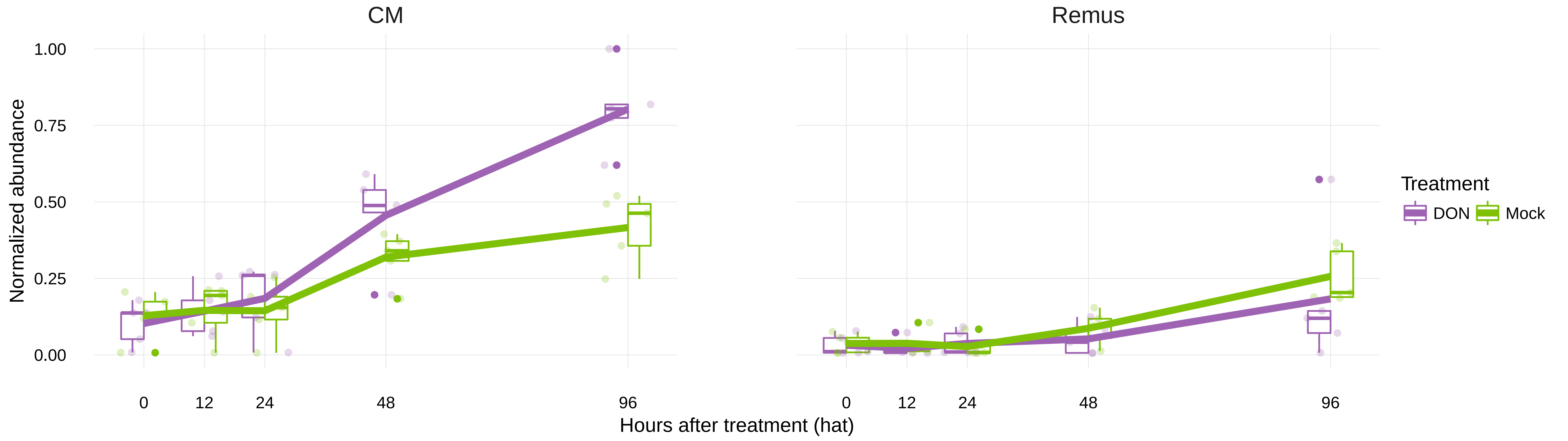

DON, Mock; all four genotypes

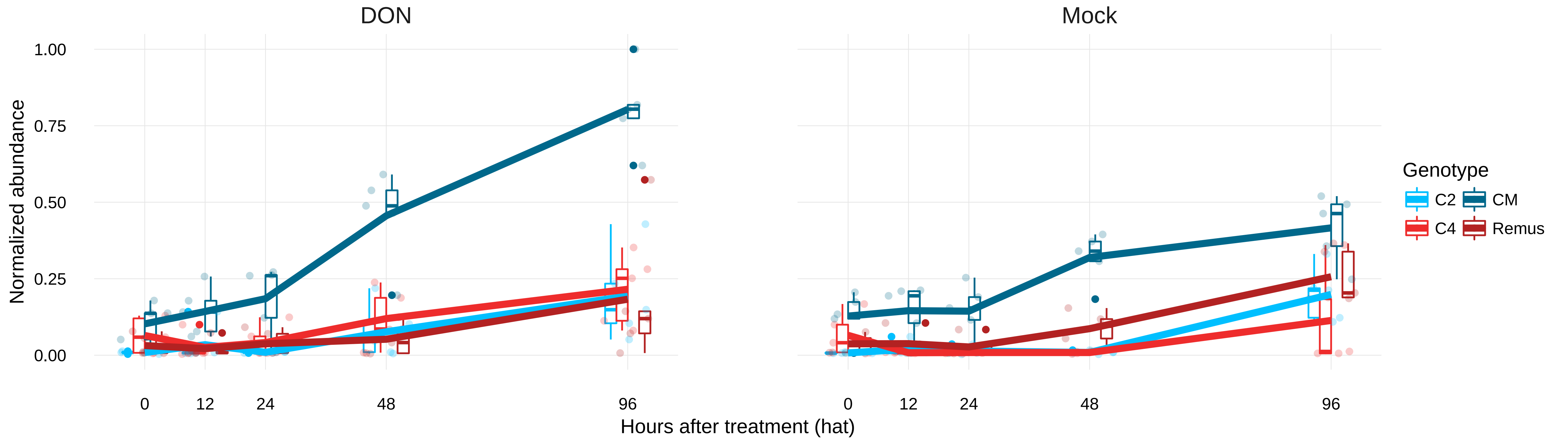

A.88

Annotated as HCA derivative
(1 database hit)

MZ	393.1157
RT	15.31 min
Normalization	Directly via KPX samples
Cluster	–
Cn total / Phe	17 /

C2, C4; different treatments

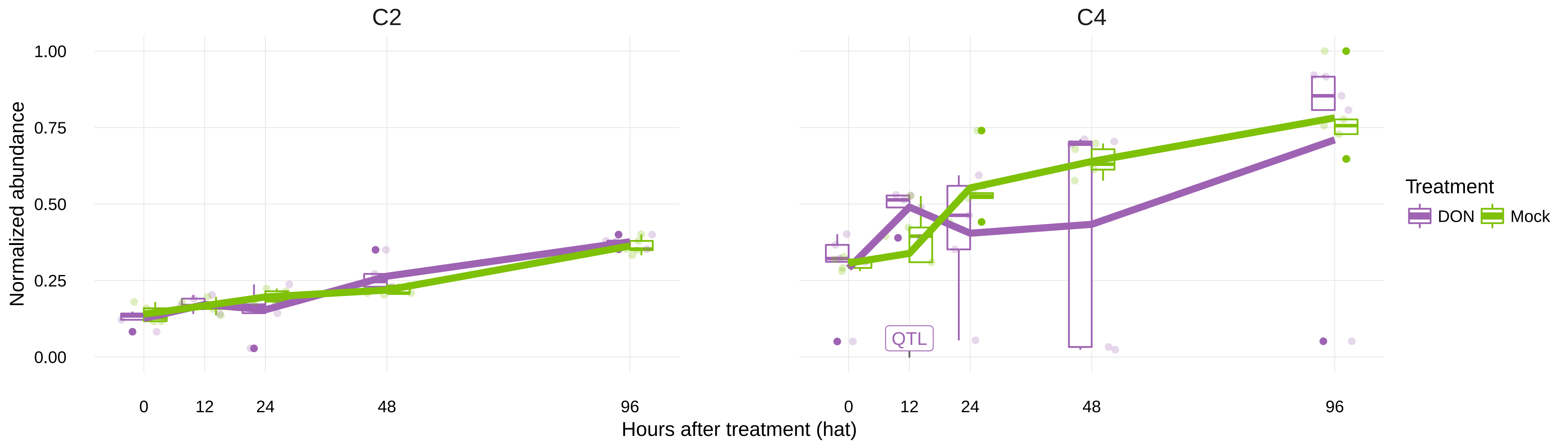

DON, Mock; different genotypes

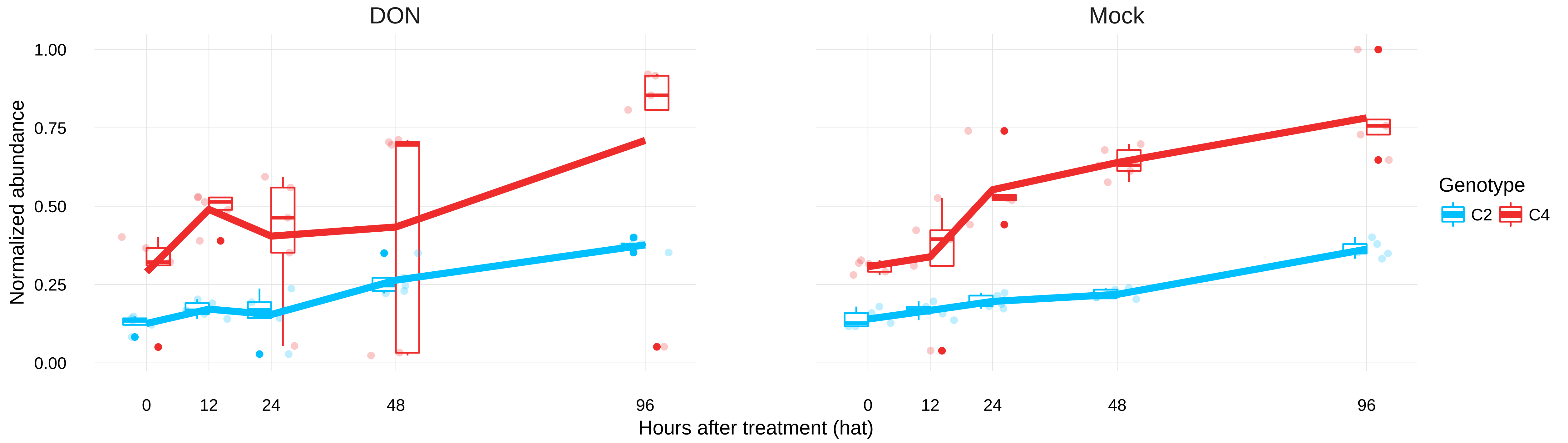

CM, Remus; different treatments

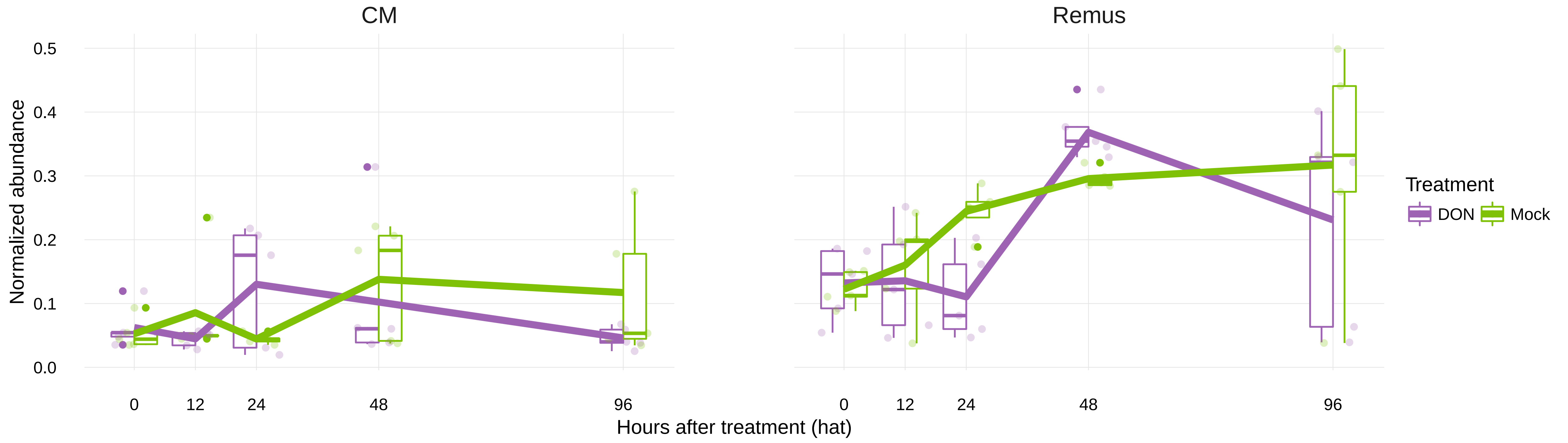

DON, Mock; all four genotypes

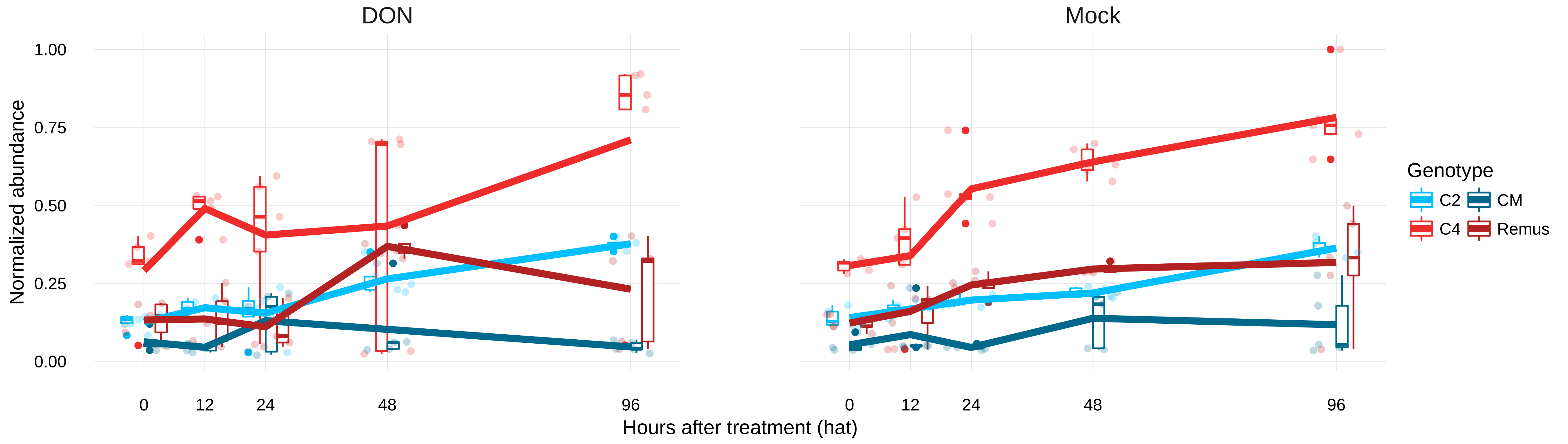

A.52

Annotated as HCA derivative
(1 database hit)

MZ	343.1024
RT	12.22 min
Normalization	Directly via KPX samples
Cluster	–
Cn total / Phe	15 /

C2, C4; different treatments

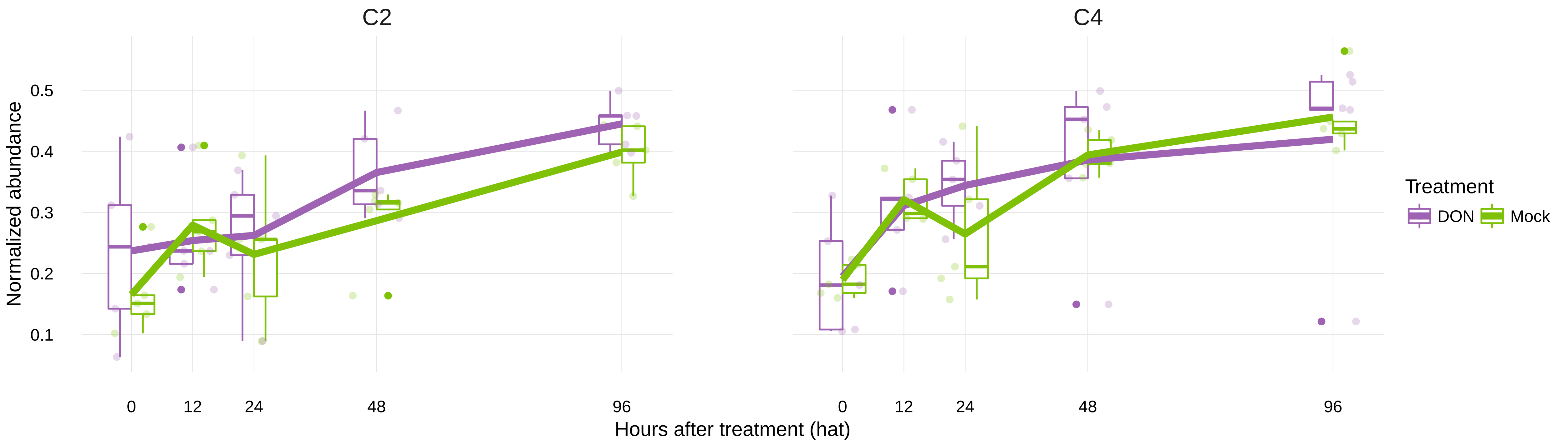

DON, Mock; different genotypes

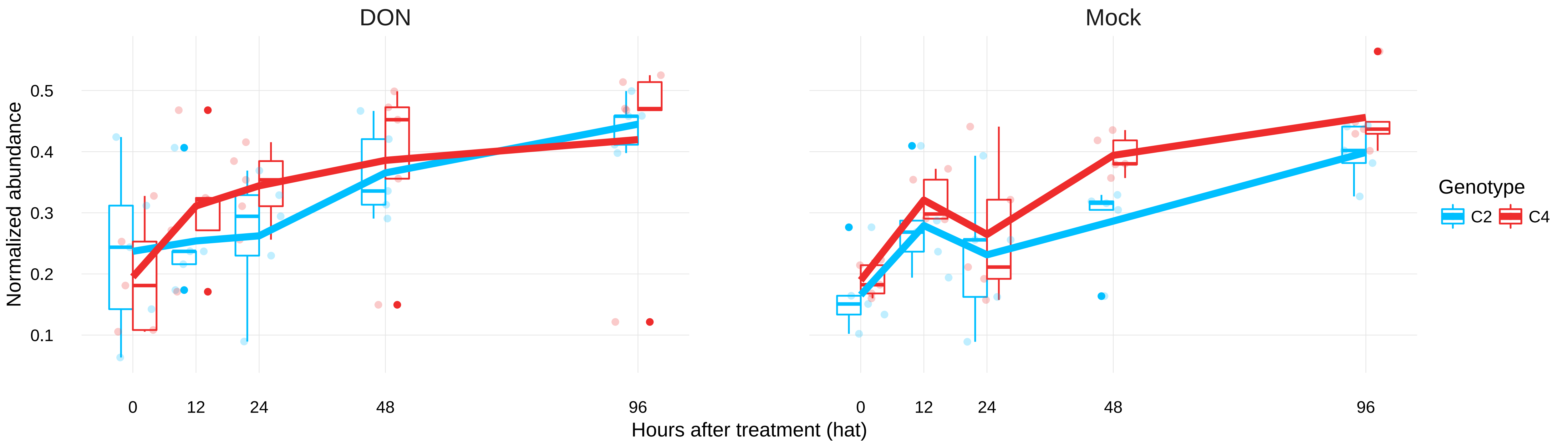

CM, Remus; different treatments

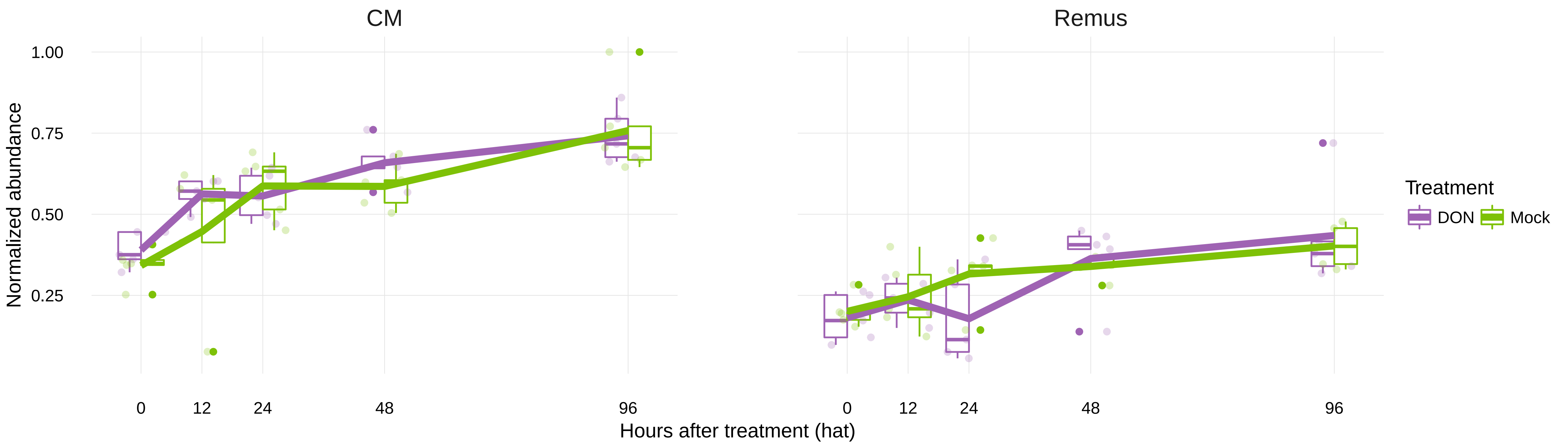

DON, Mock; all four genotypes

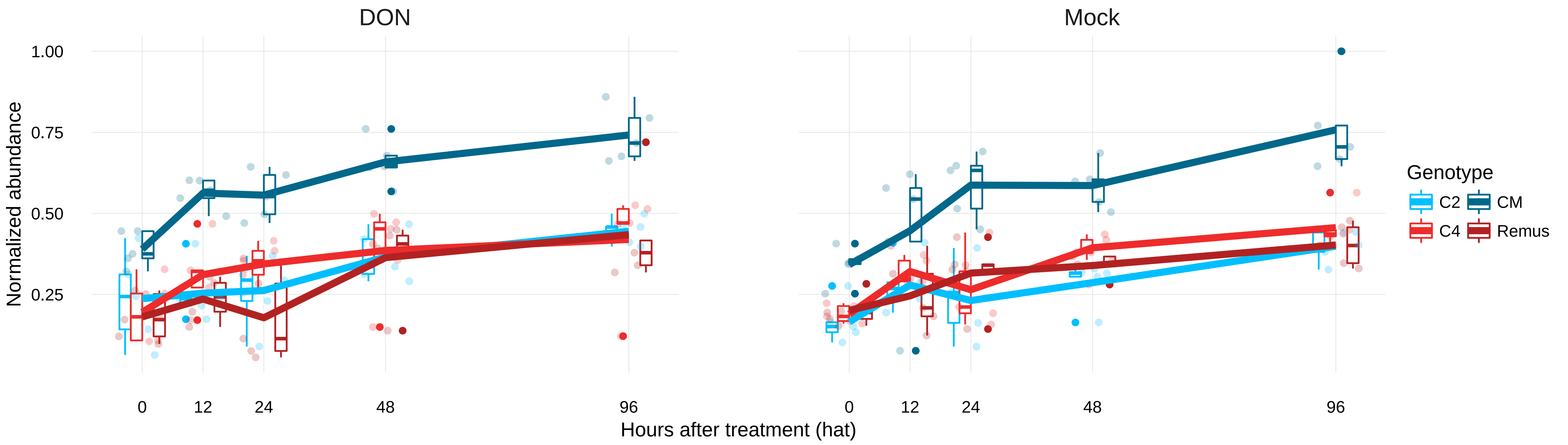

A.131

Annotated as others (Coumarins)

(1 database hit)

MZ	177.0546
RT	21.16 min
Normalization	Directly via KPX samples
Cluster	–
Cn total / Phe	10 / 9

C2, C4; different treatments

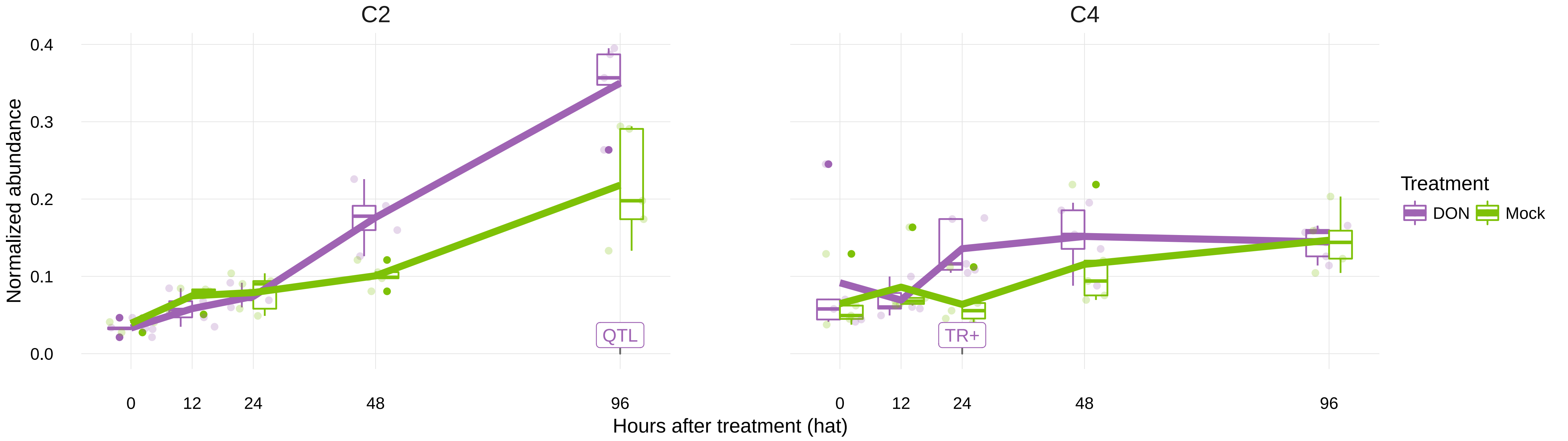

DON, Mock; different genotypes

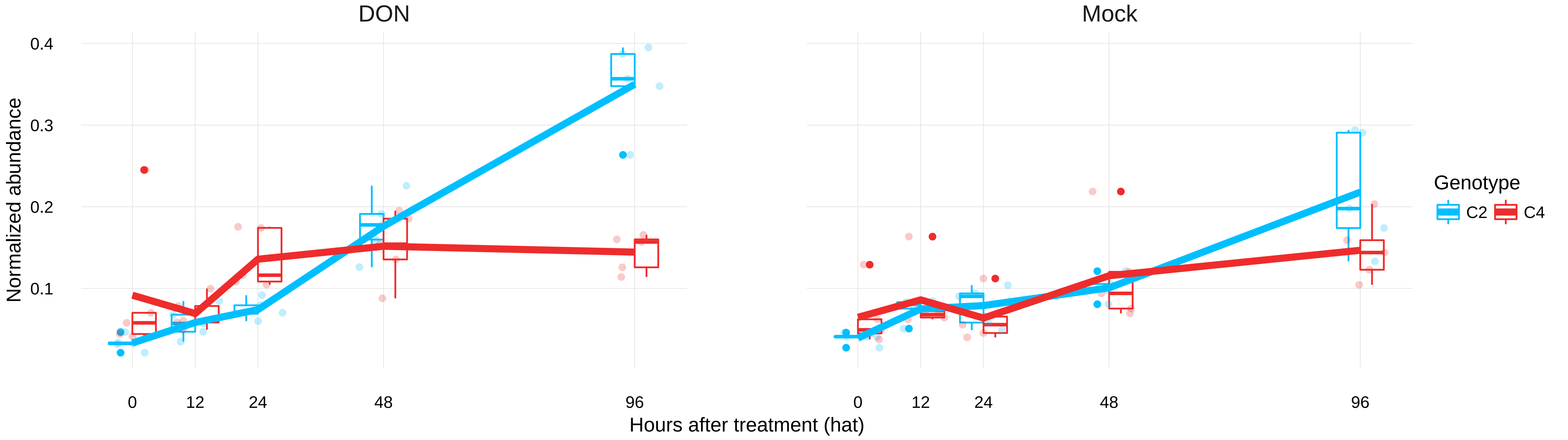

CM, Remus; different treatments

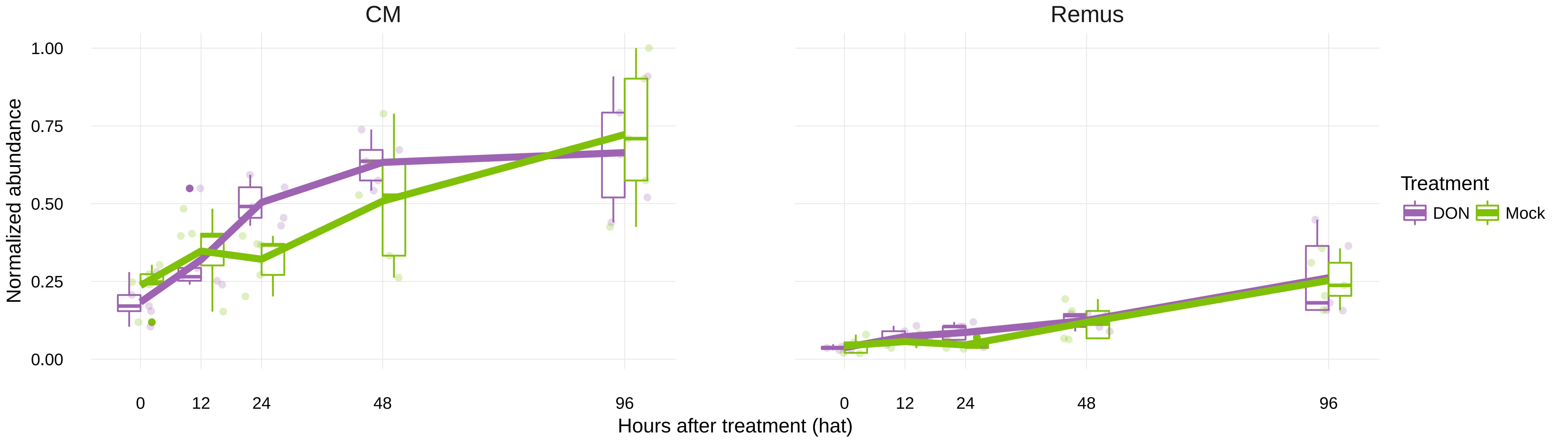

DON, Mock; all four genotypes

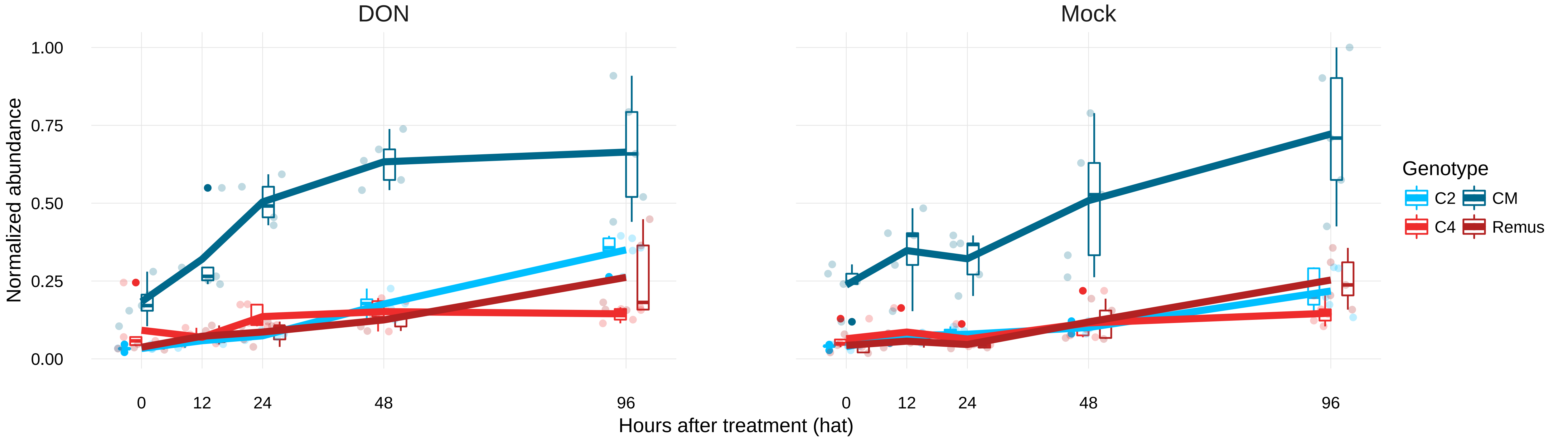

A.128

Annotated as Flavonoid

(25 database hits)

MZ	405.1181
RT	20.88 min
Normalization	Directly via KPX samples
Cluster	–
Cn total / Phe	20 /

C2, C4; different treatments

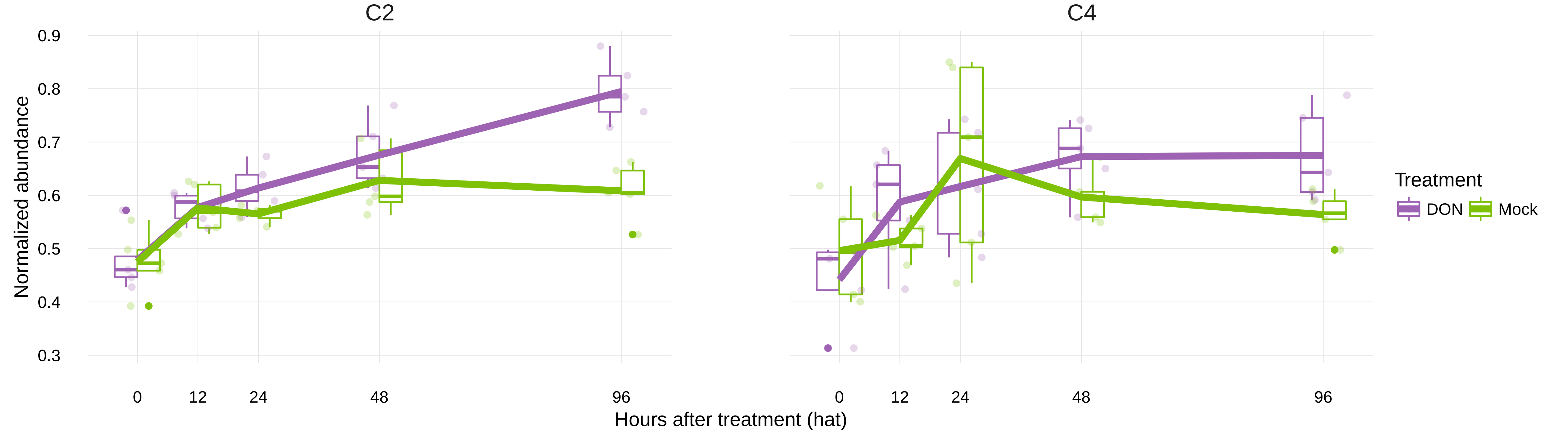

DON, Mock; different genotypes

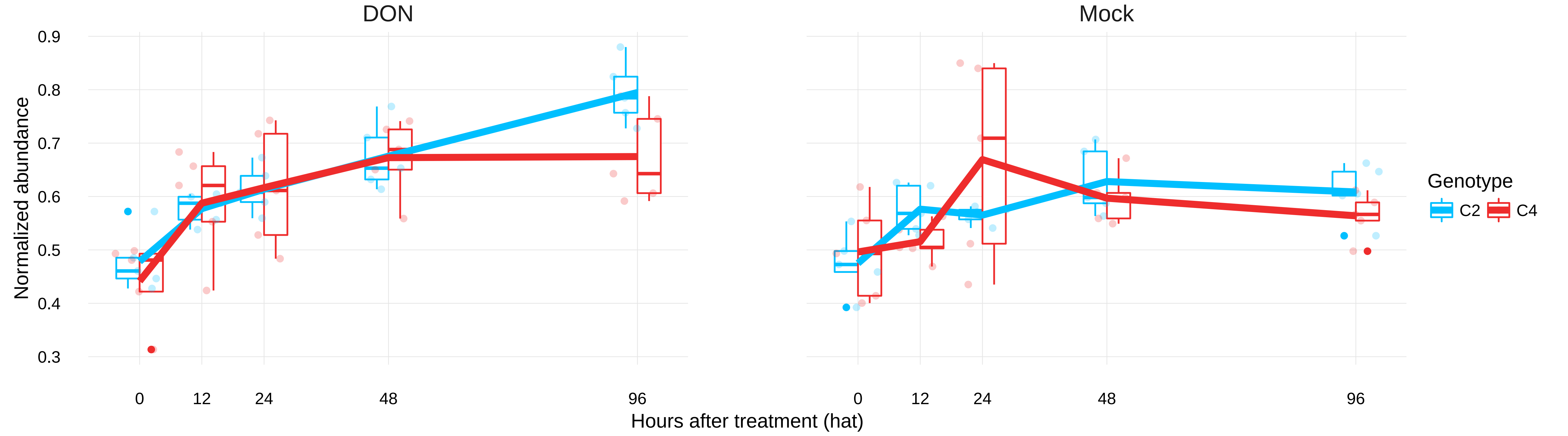

CM, Remus; different treatments

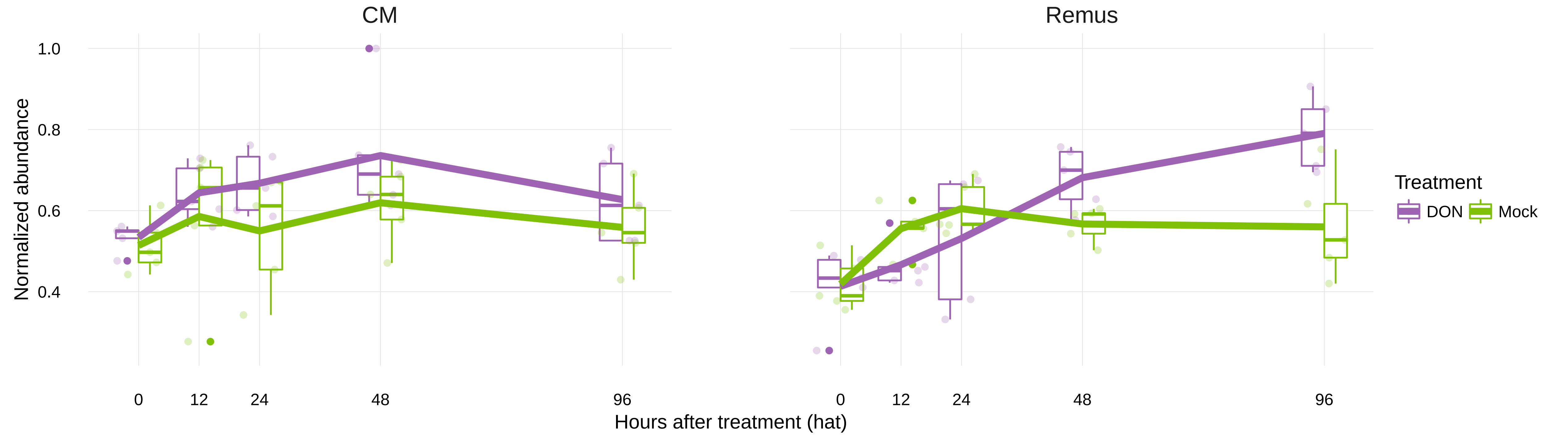

DON, Mock; all four genotypes

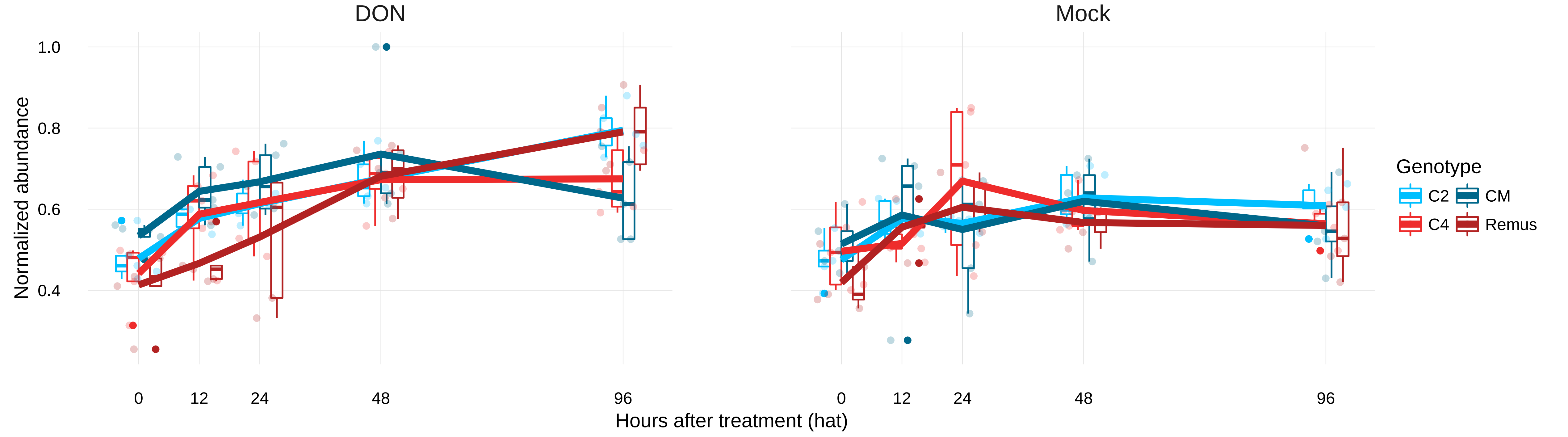

A.135

Annotated as HCA derivative
(1 database hit)

MZ	885.2422
RT	21.51 min
Normalization	Indirectly via surrogate in the KPX samples
Cluster	–
Cn total / Phe	40 / Several: 9,18

C2, C4; different treatments

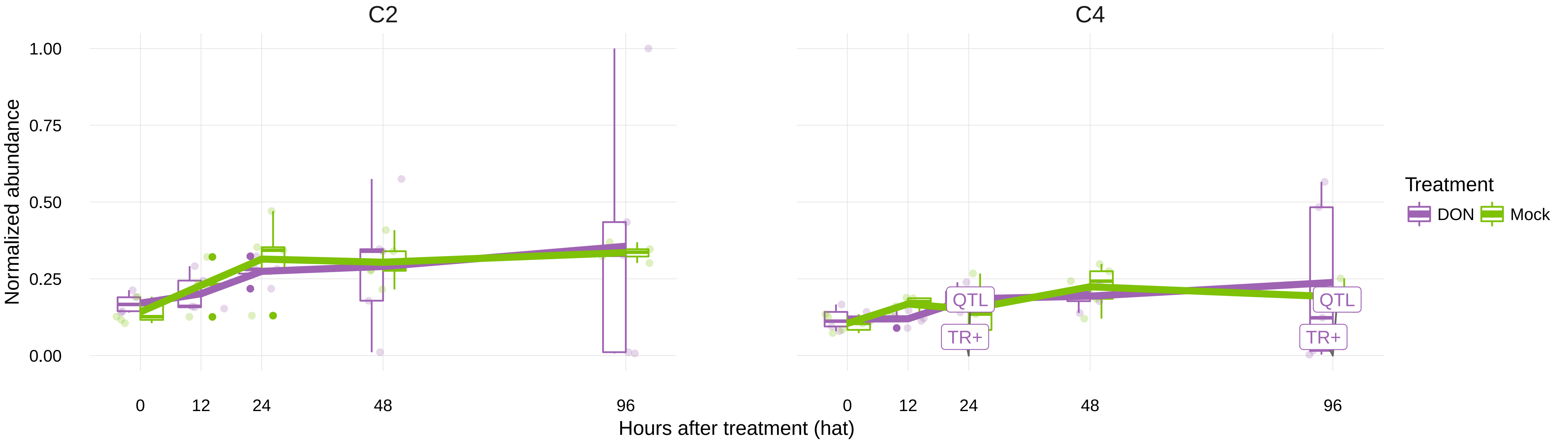

DON, Mock; different genotypes

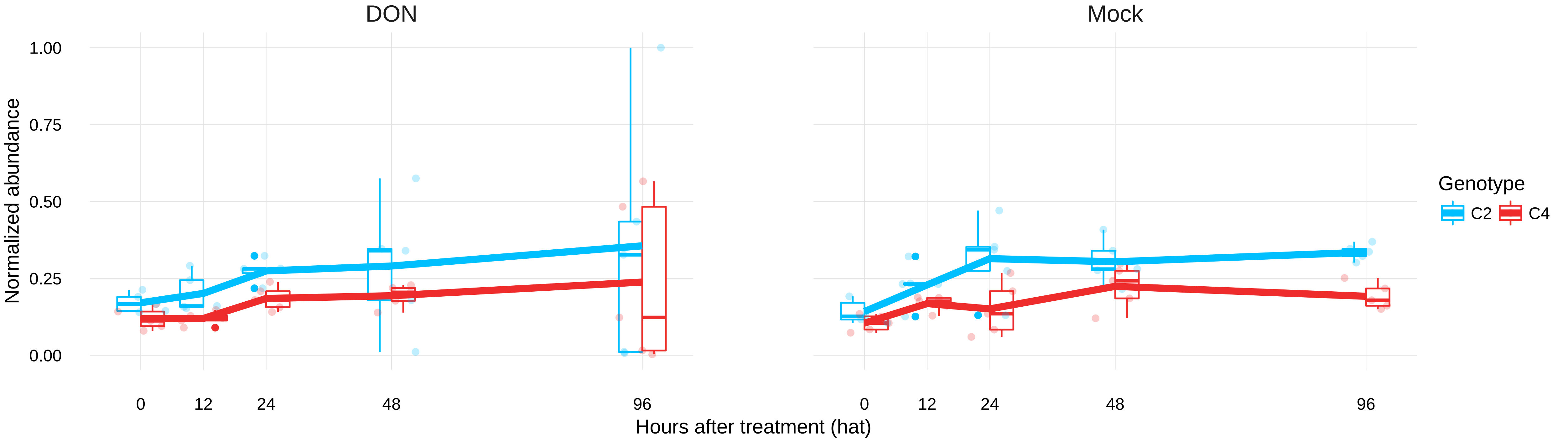

CM, Remus; different treatments

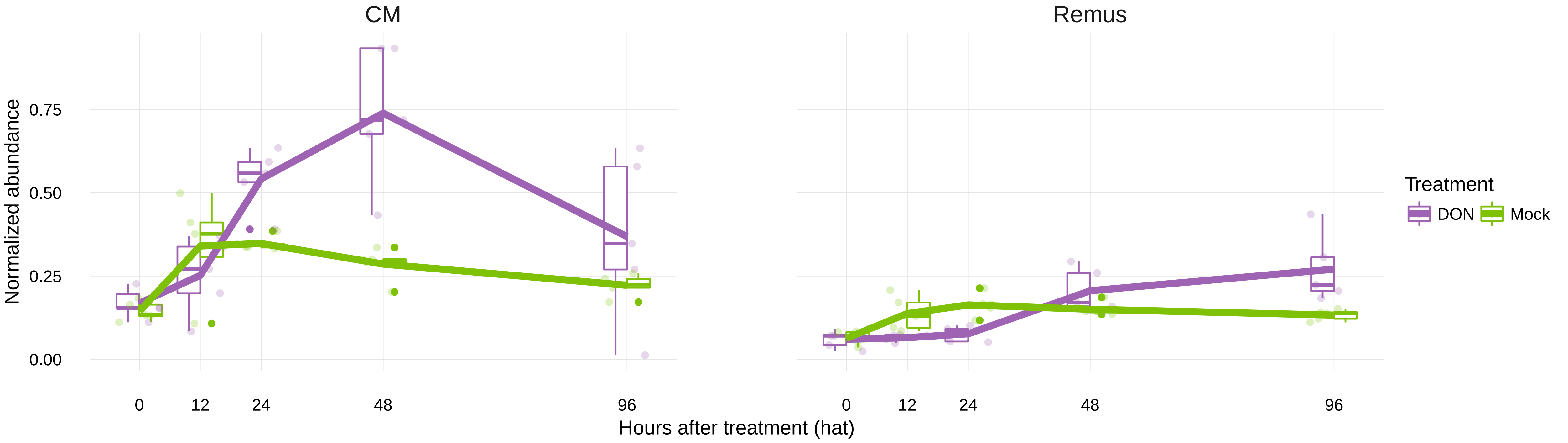

DON, Mock; all four genotypes

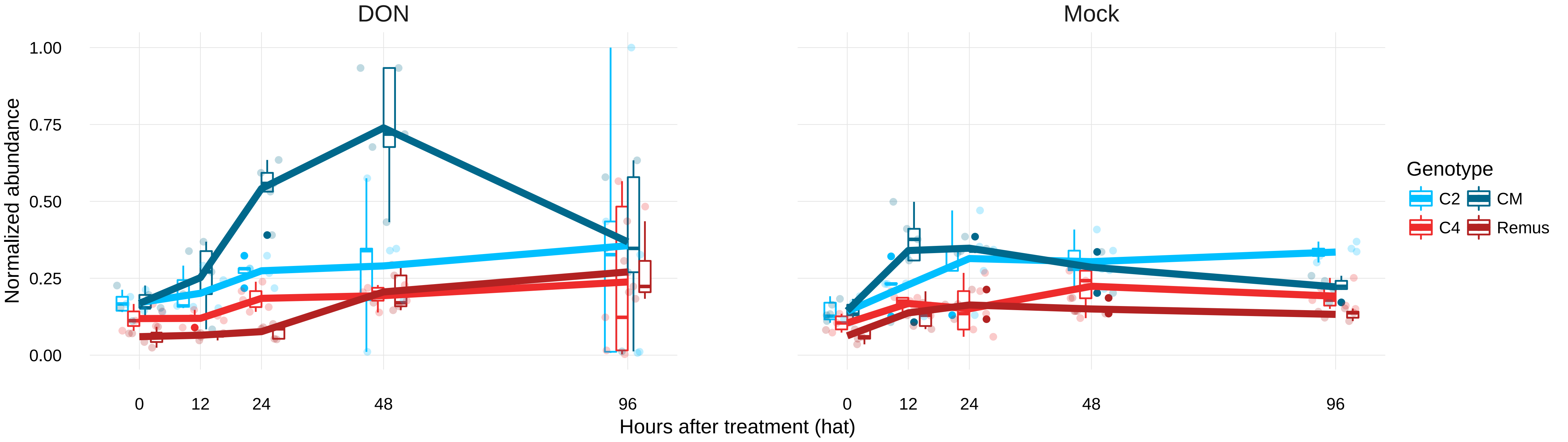

A.21

Annotated as HCAA
(2 database hits)

MZ	293.1611
RT	8.25 min
Normalization	Directly via KPX samples
Cluster	–
Cn total / Phe	14 /

C2, C4; different treatments

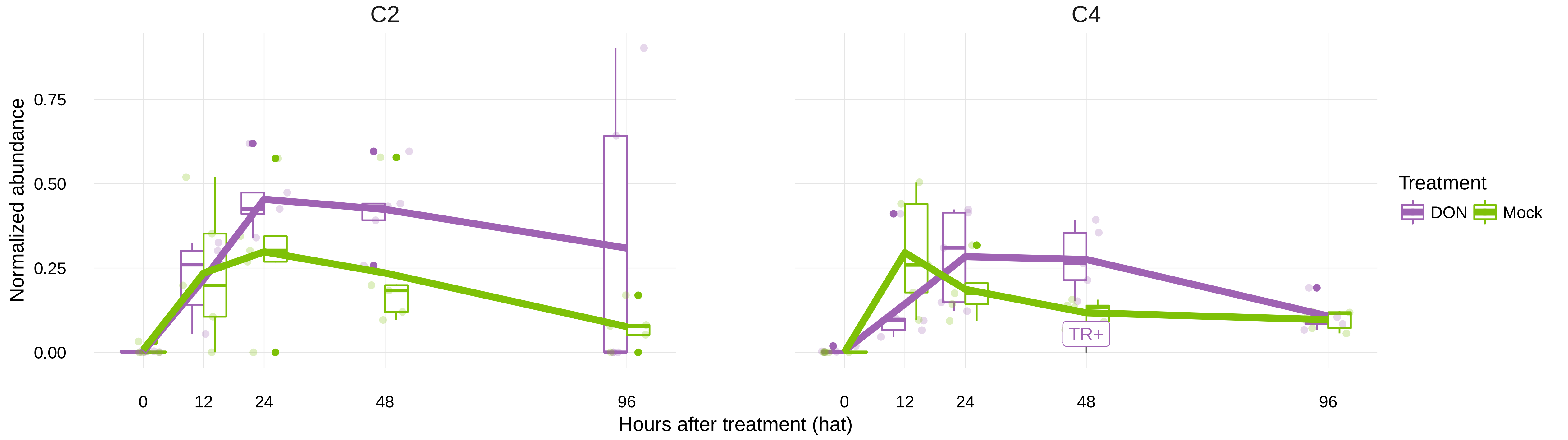

DON, Mock; different genotypes

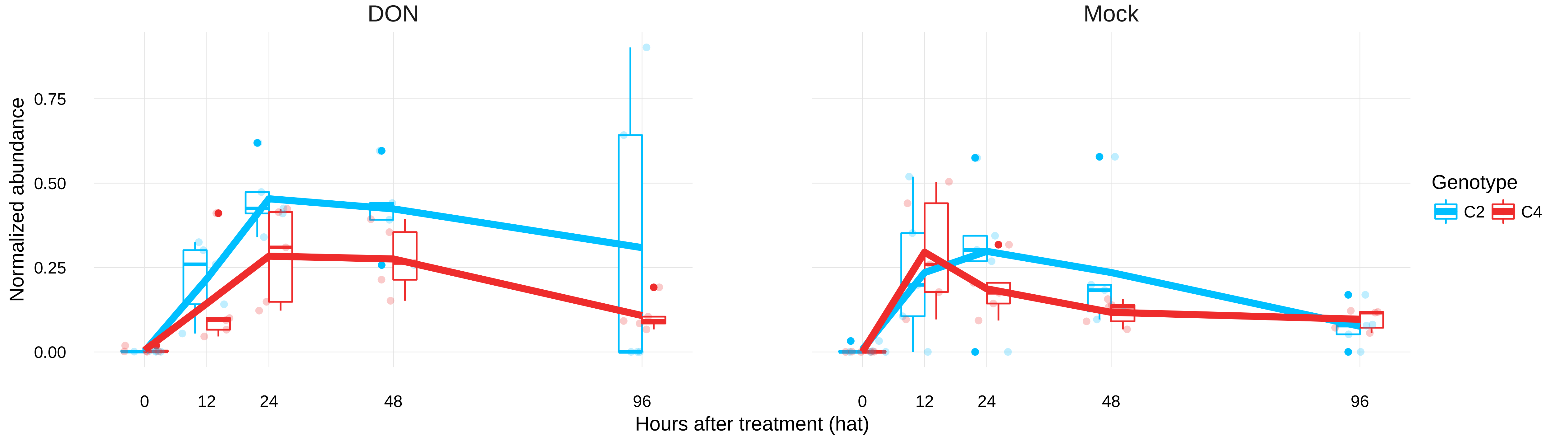

CM, Remus; different treatments

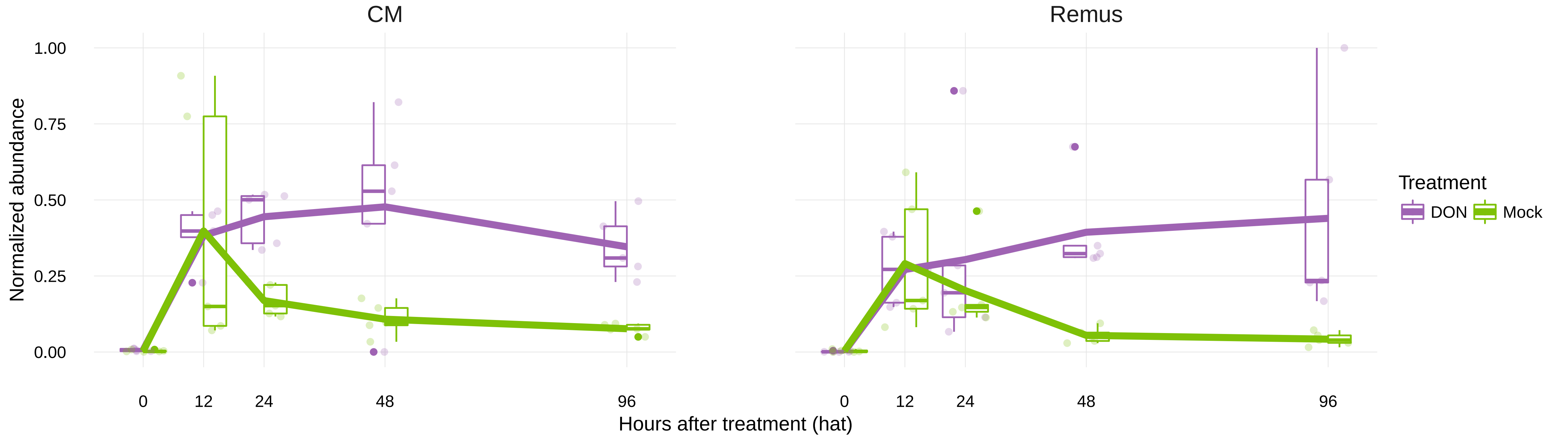

DON, Mock; all four genotypes

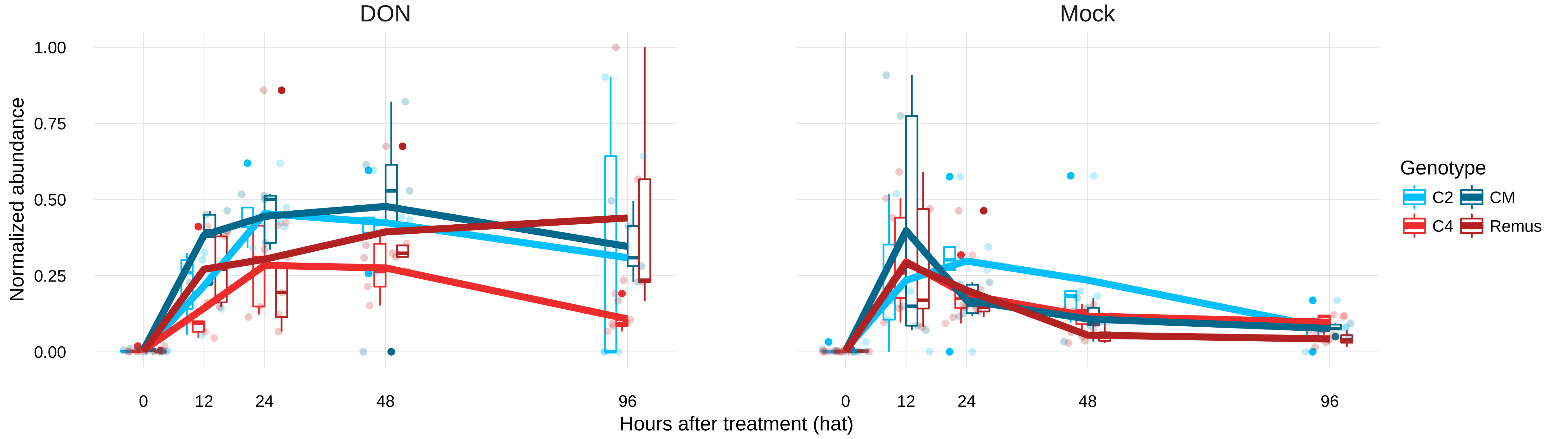

A.124

Annotated as Flavonoid
(30 database hits)

MZ	341.1385
RT	20.25 min
Normalization	Indirectly via surrogate in the KPX samples
Cluster	–
Cn total / Phe	20 /

C2, C4; different treatments

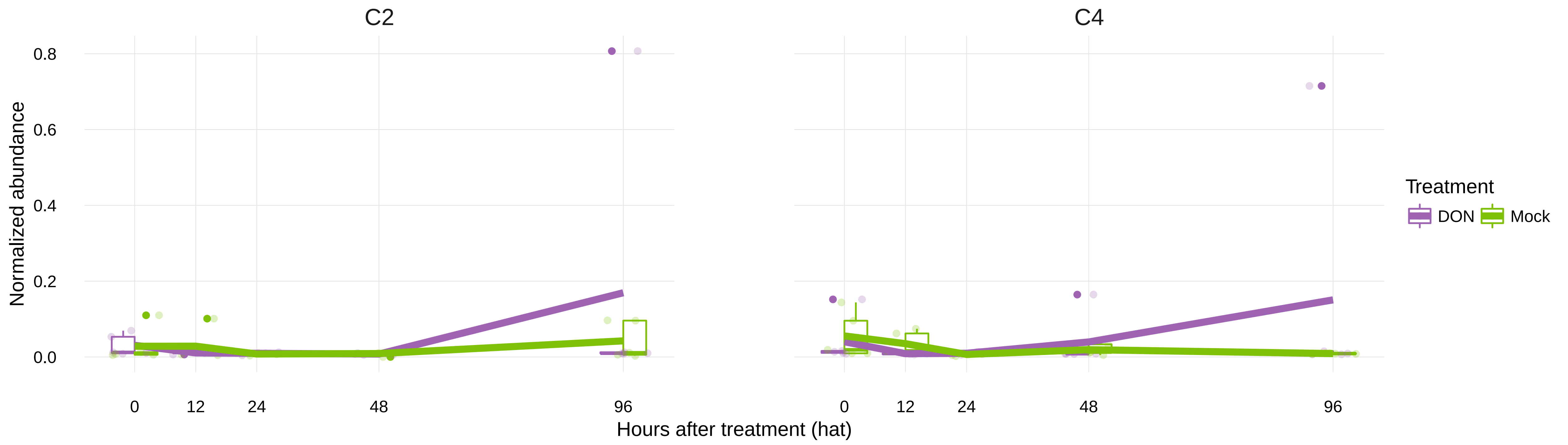

DON, Mock; different genotypes

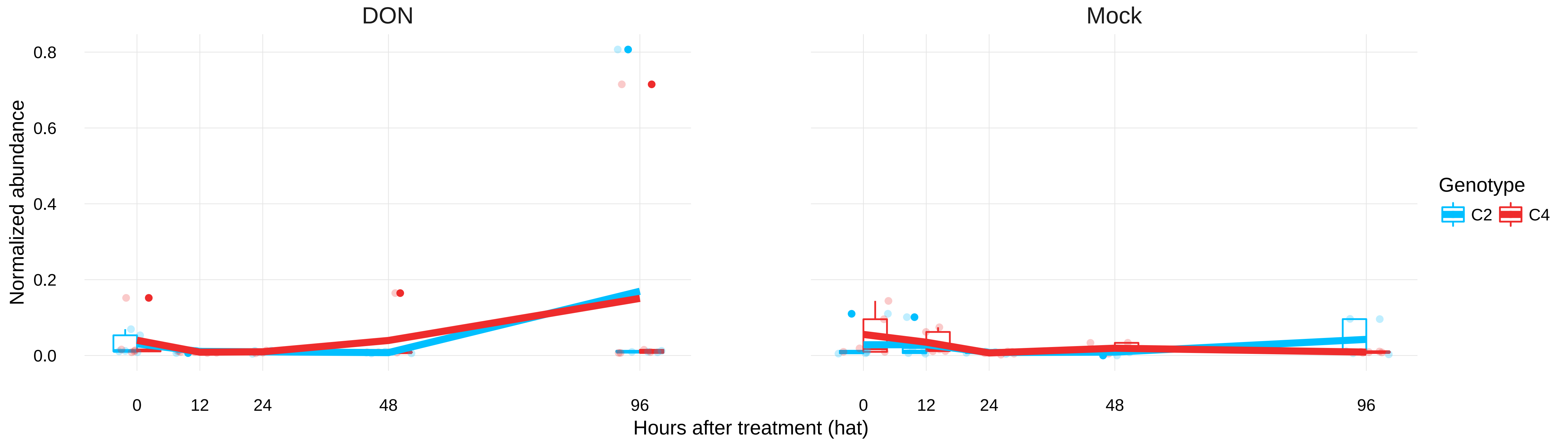

CM, Remus; different treatments

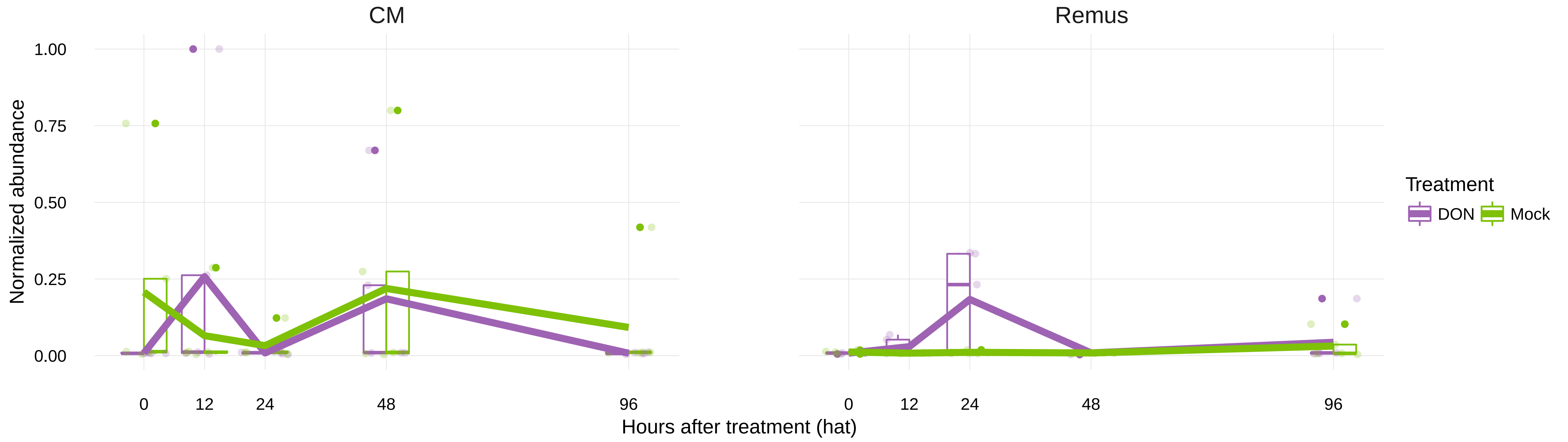

DON, Mock; all four genotypes

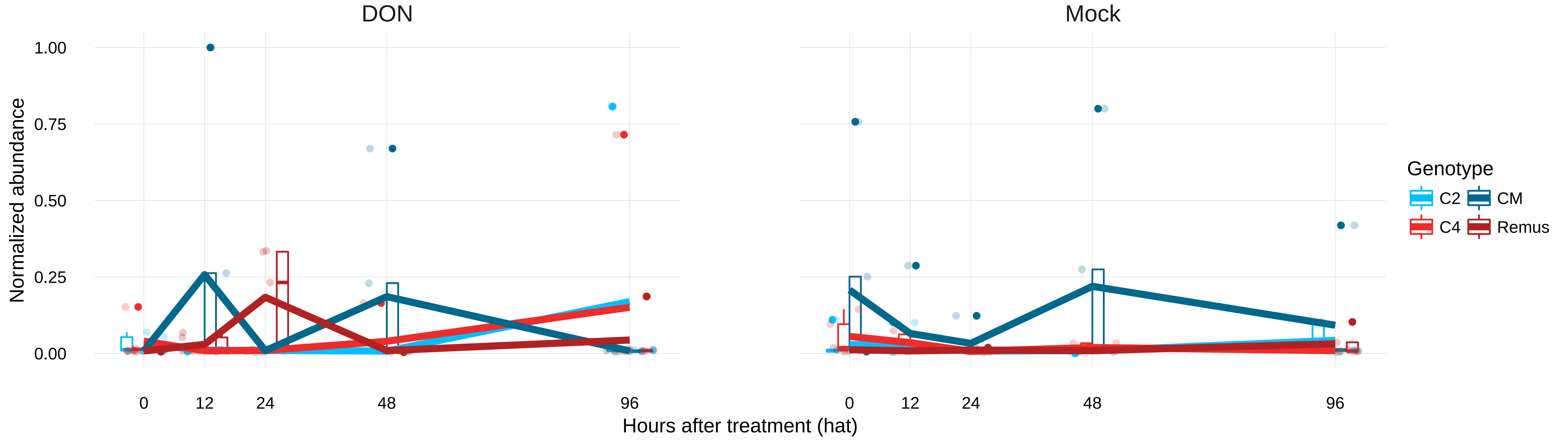

A.19

Annotated as HCAA
(1 database hit)

MZ	235.1447
RT	7.48 min
Normalization	Indirectly via surrogate in the KPX samples
Cluster	–
Cn total / Phe	9 / 9

C2, C4; different treatments

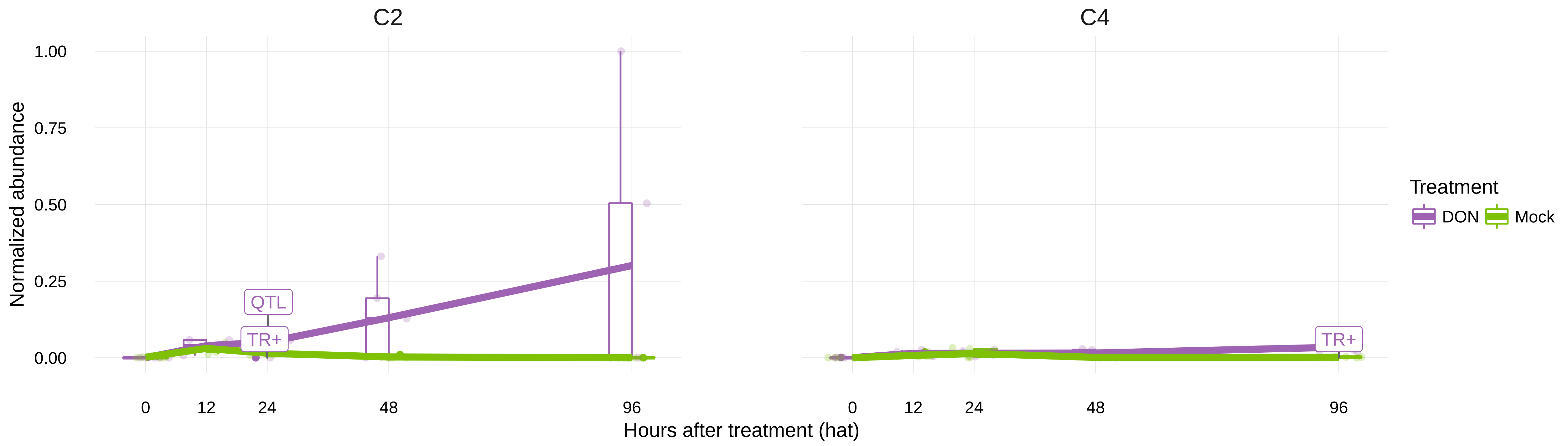

DON, Mock; different genotypes

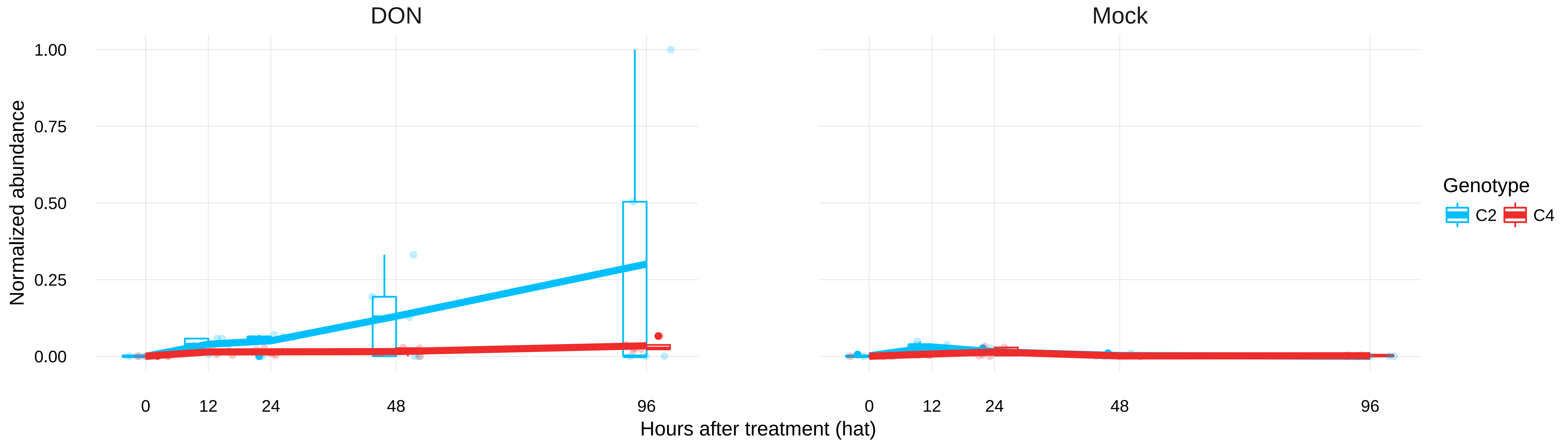

CM, Remus; different treatments

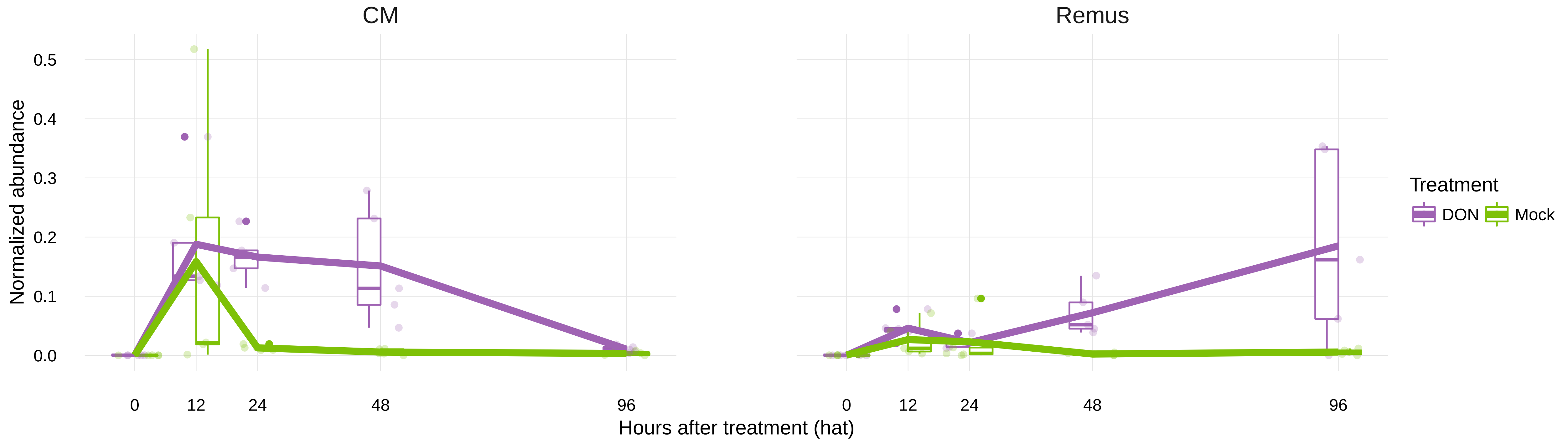

DON, Mock; all four genotypes

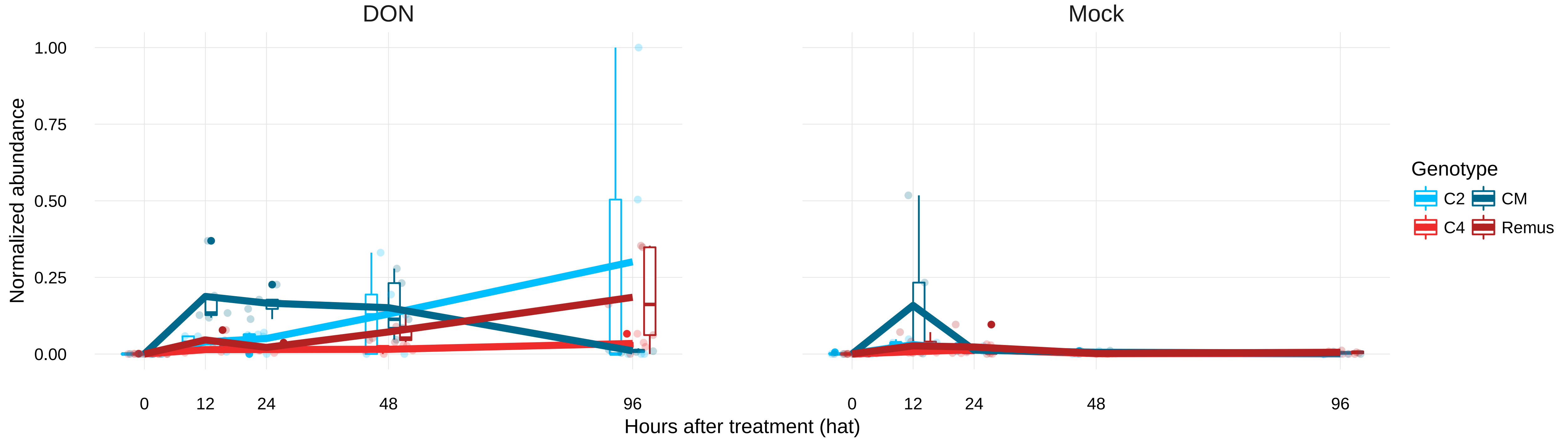

A.140

Annotated as Flavonoid

(43 database hits)

MZ	301.1073
RT	27.16 min
Normalization	Directly via KPX samples
Cluster	–
Cn total / Phe	17 /

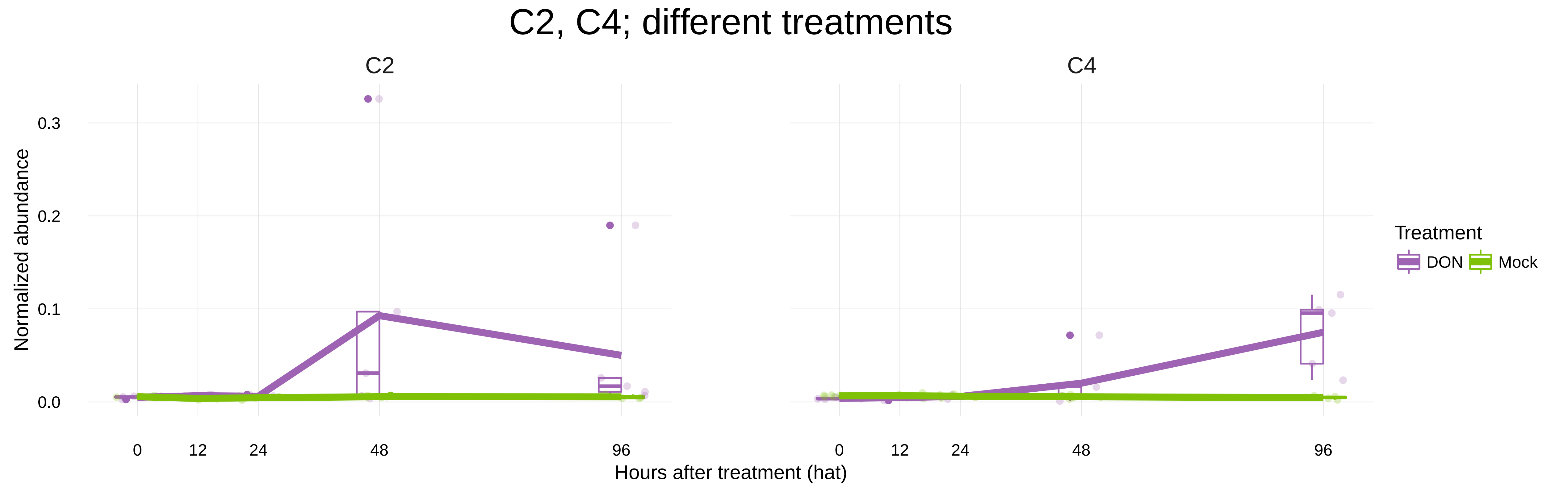

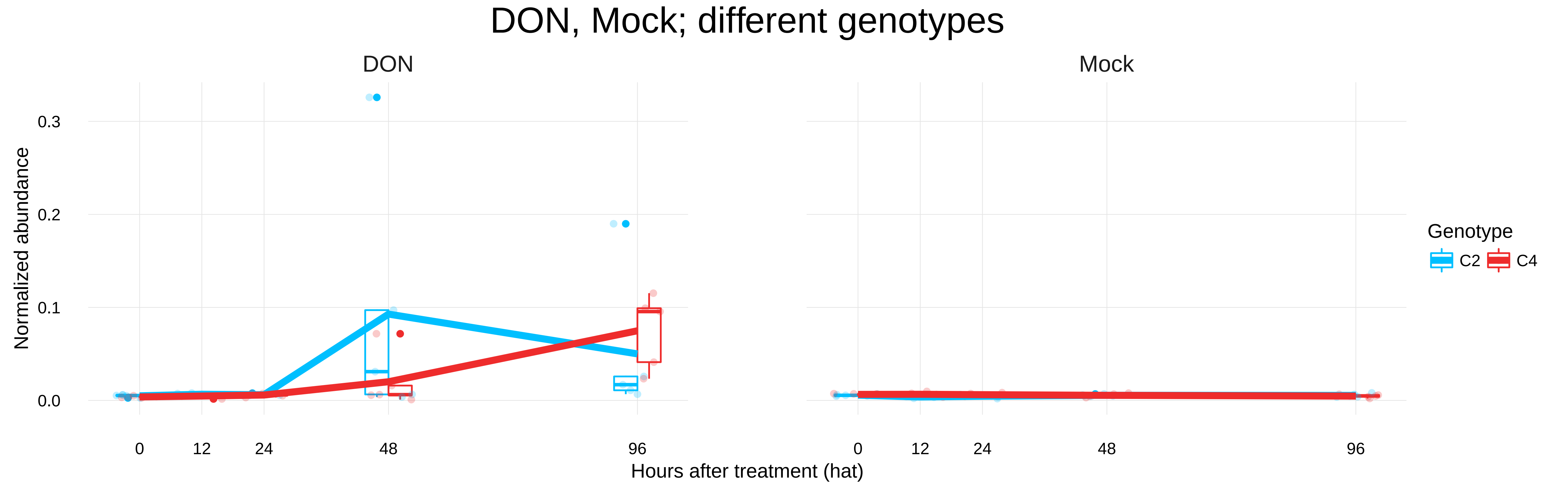

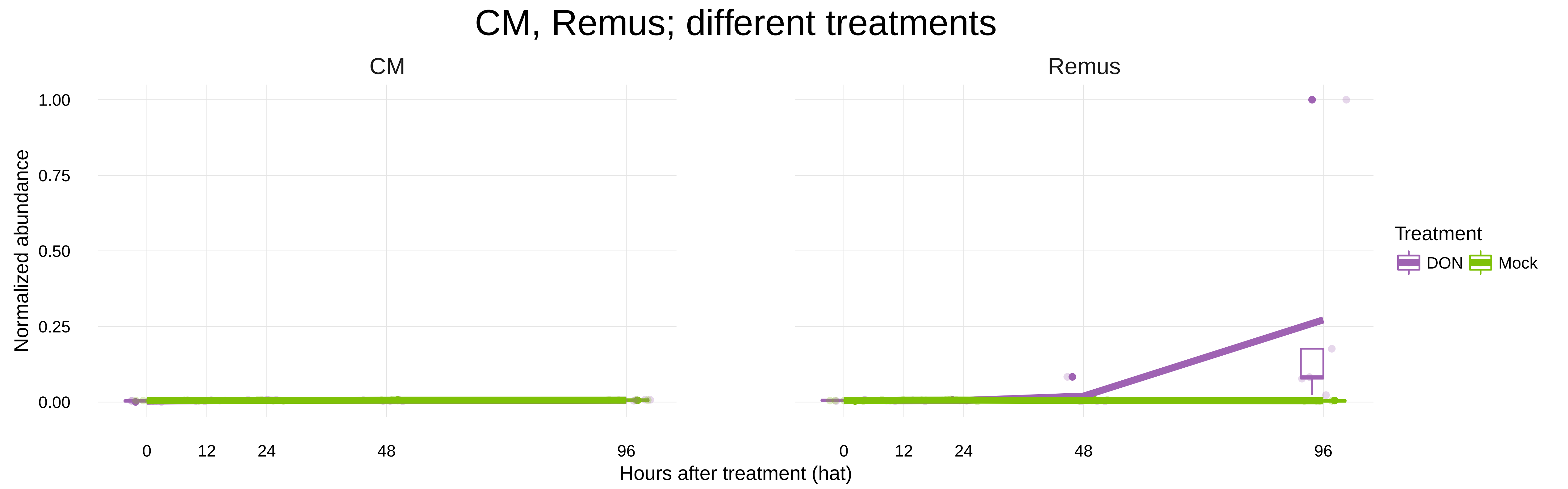

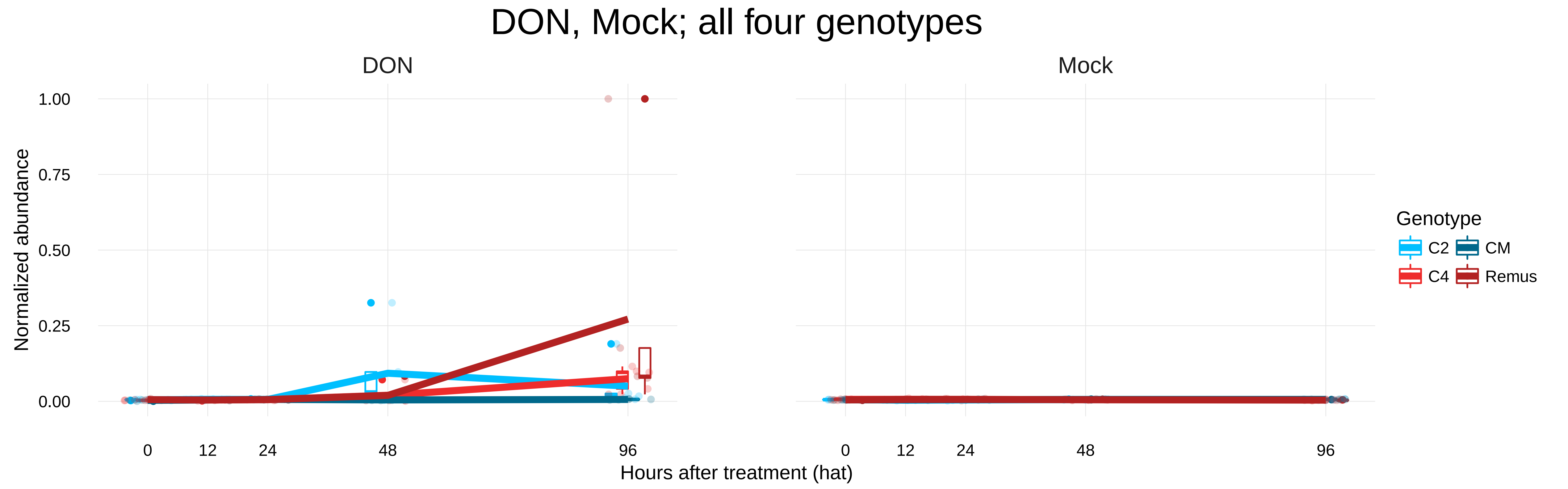

A.103

Annotated as Flavonoid, Lignan or HCA derivative
(15 database hits)

MZ	359.1491
RT	16.85 min
Normalization	Indirectly via surrogate in the KPX samples
Cluster	–
Cn total / Phe	20 / 9

C2, C4; different treatments

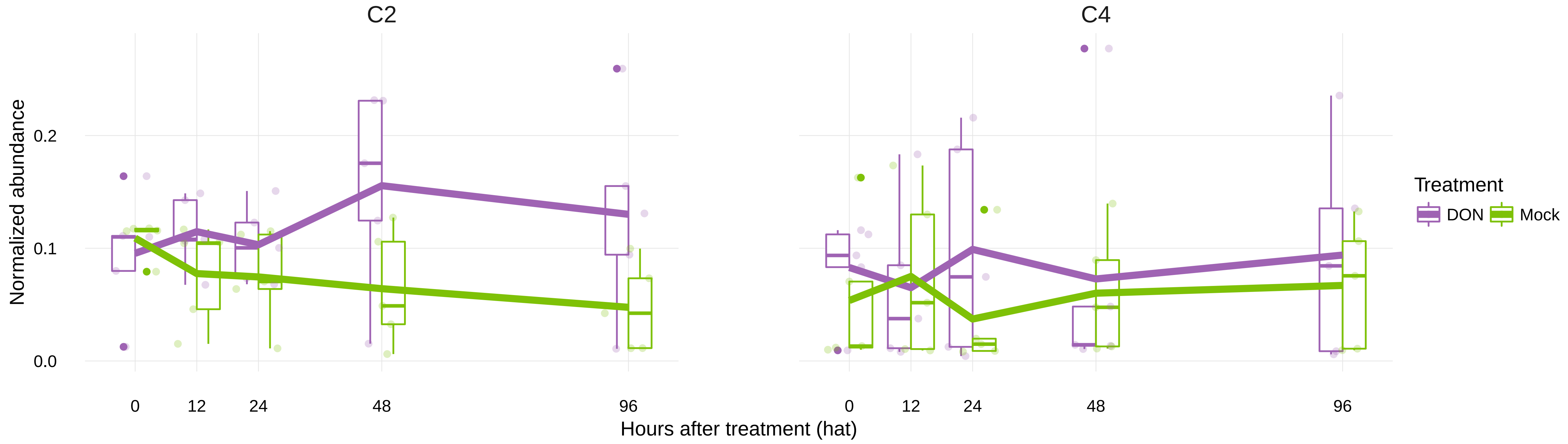

DON, Mock; different genotypes

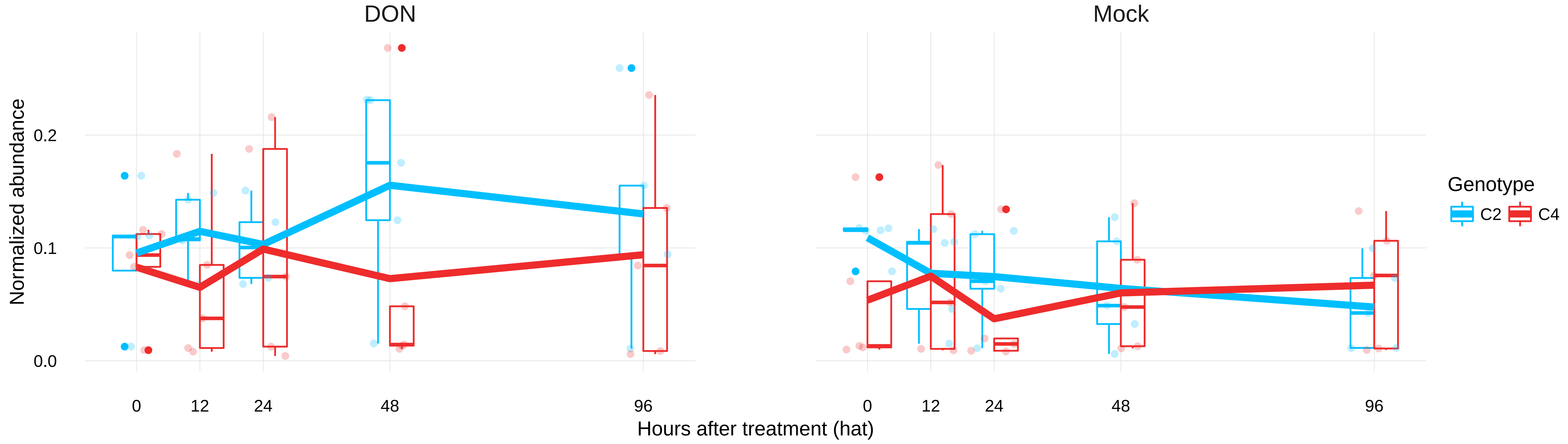

CM, Remus; different treatments

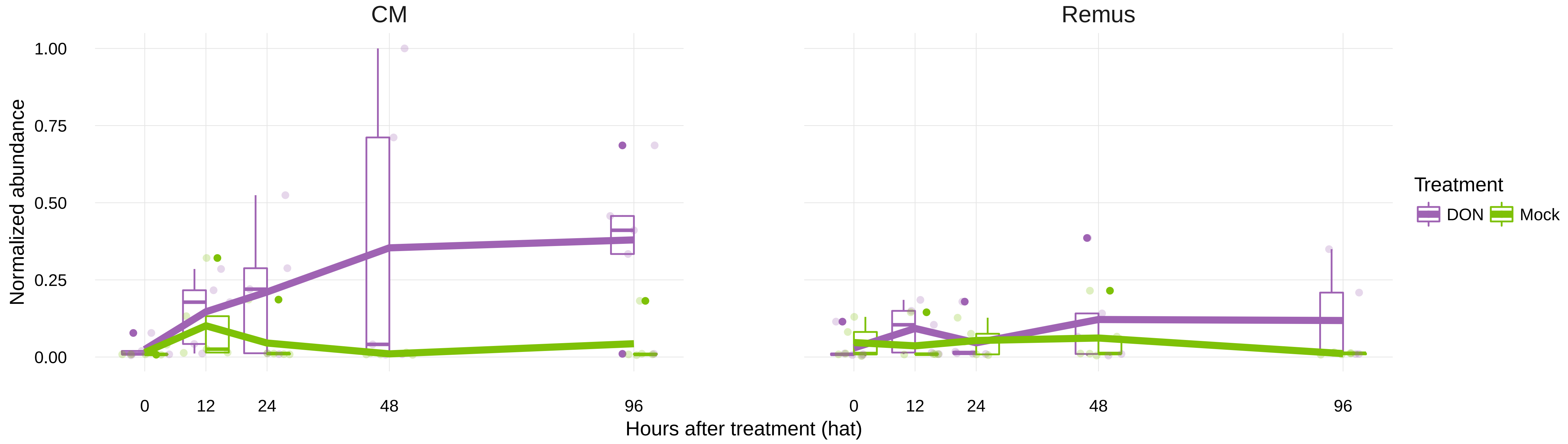

DON, Mock; all four genotypes

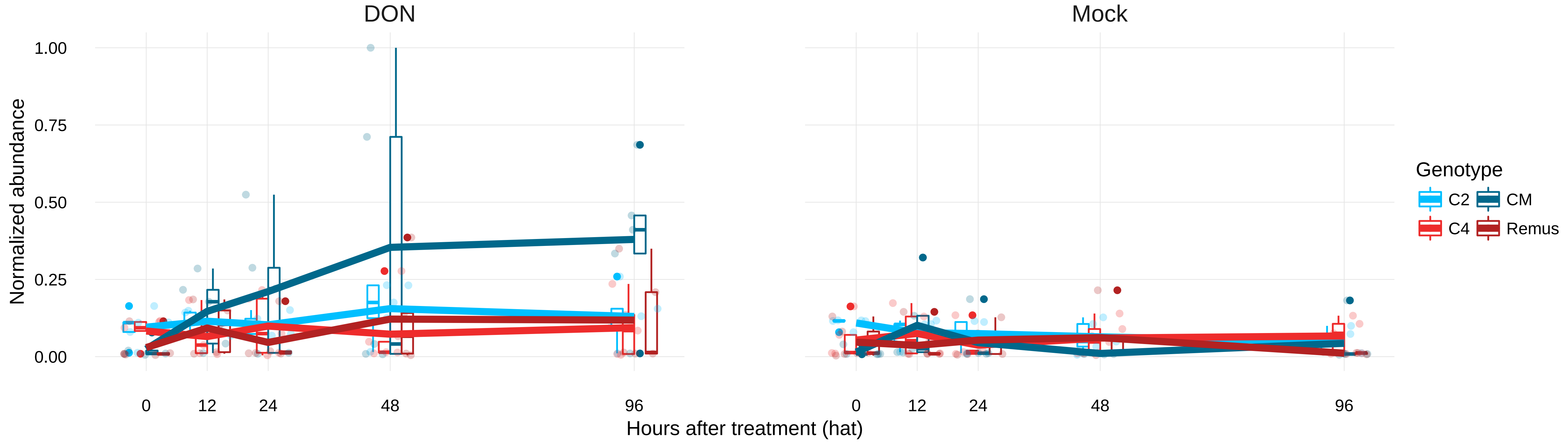

A.18

Annotated as others
(1 database hit)

MZ	153.0545
RT	7.3 min
Normalization	Indirectly via surrogate in the KPX samples
Cluster	–
Cn total / Phe	8 /

C2, C4; different treatments

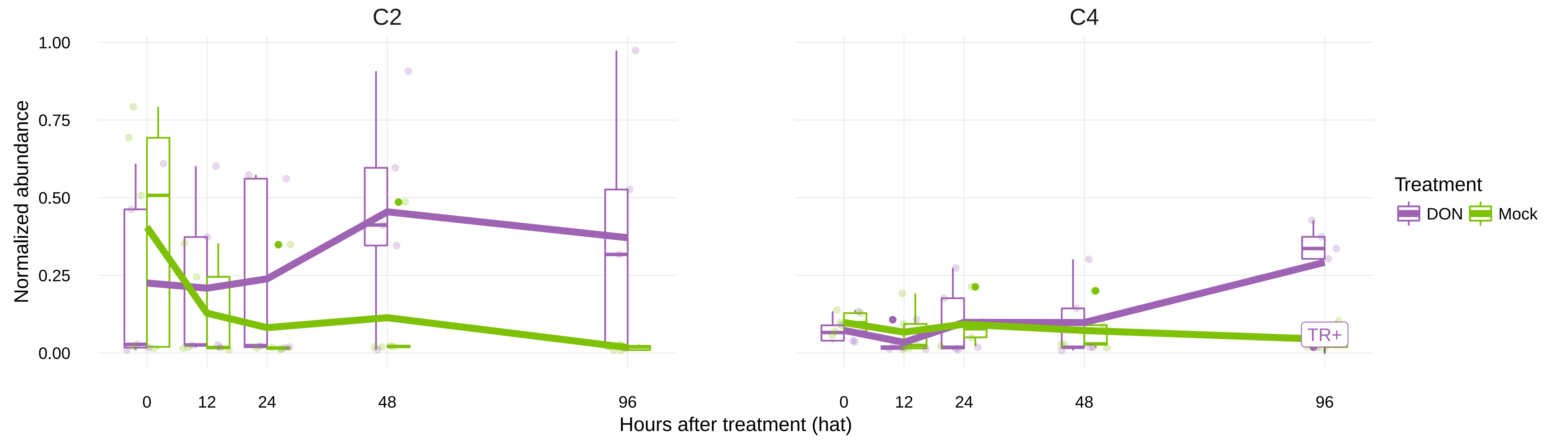

DON, Mock; different genotypes

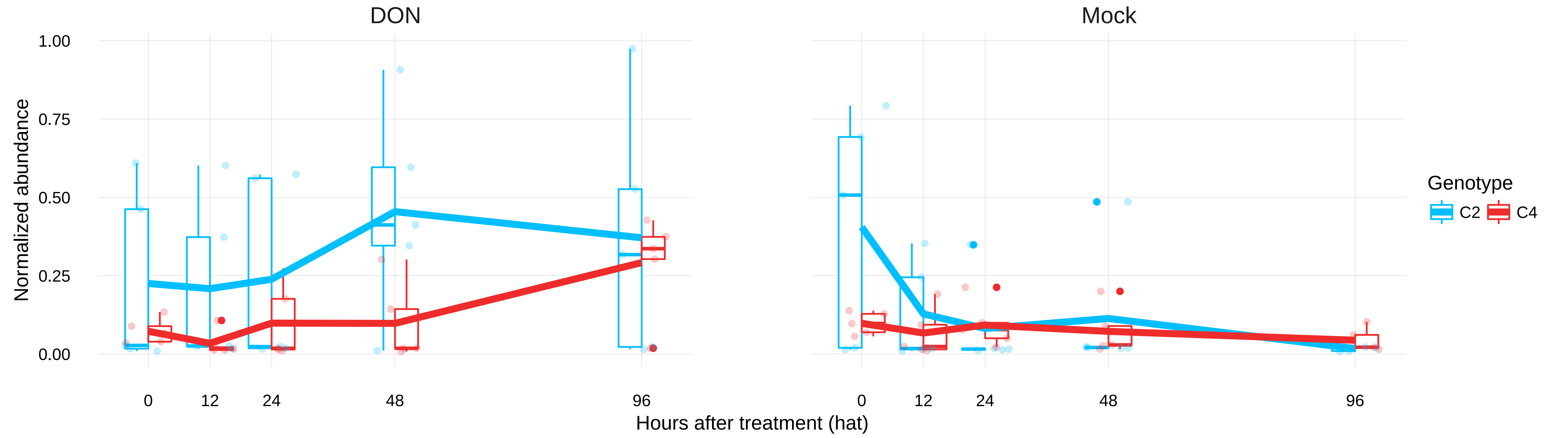

CM, Remus; different treatments

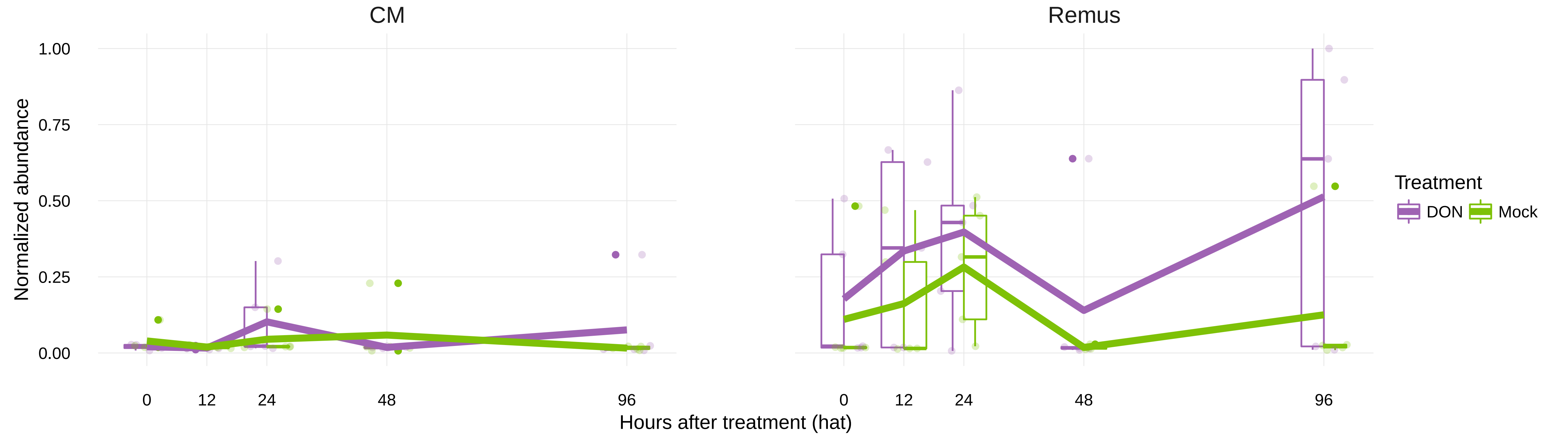

DON, Mock; all four genotypes

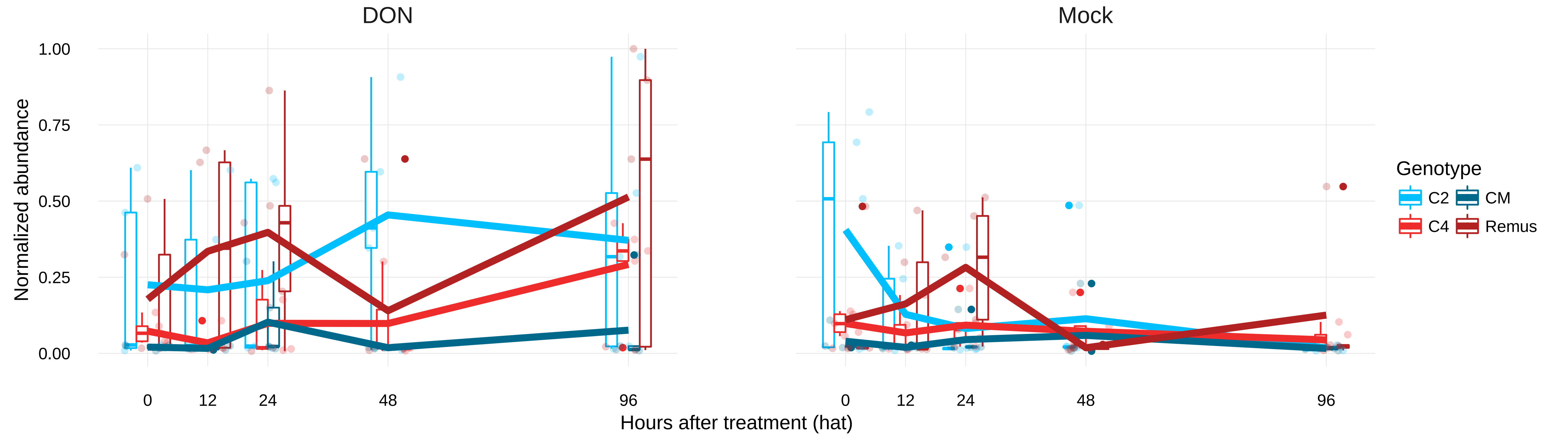

A.72

Annotated as Lignan
(1 database hit)

MZ	551.2125
RT	13.8 min
Normalization	Indirectly via surrogate in the KPX samples
Cluster	–
Cn total / Phe	27 /

C2, C4; different treatments

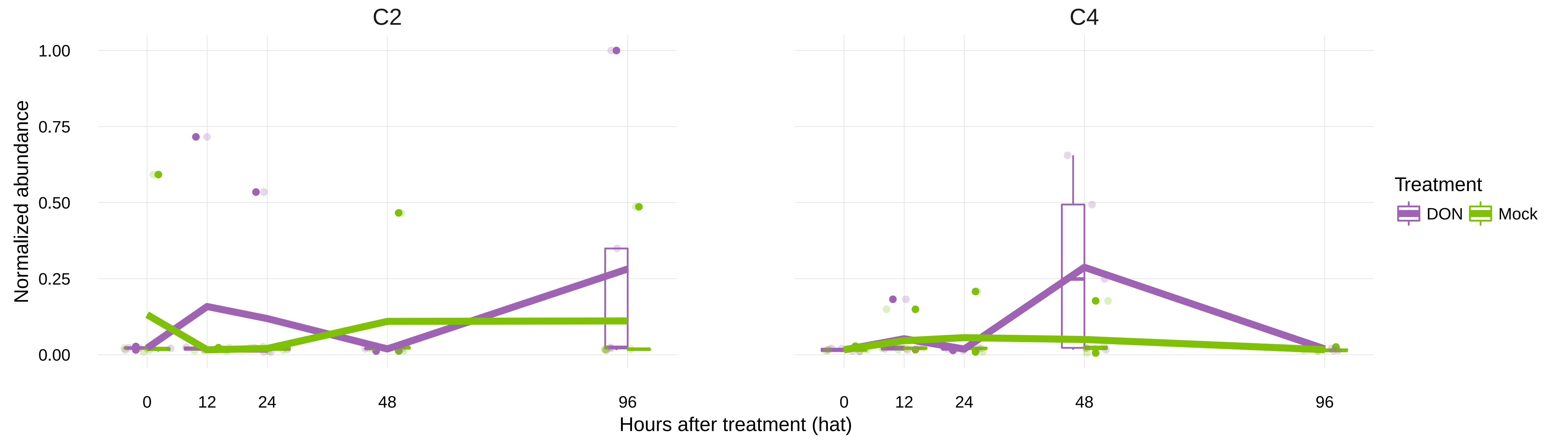

DON, Mock; different genotypes

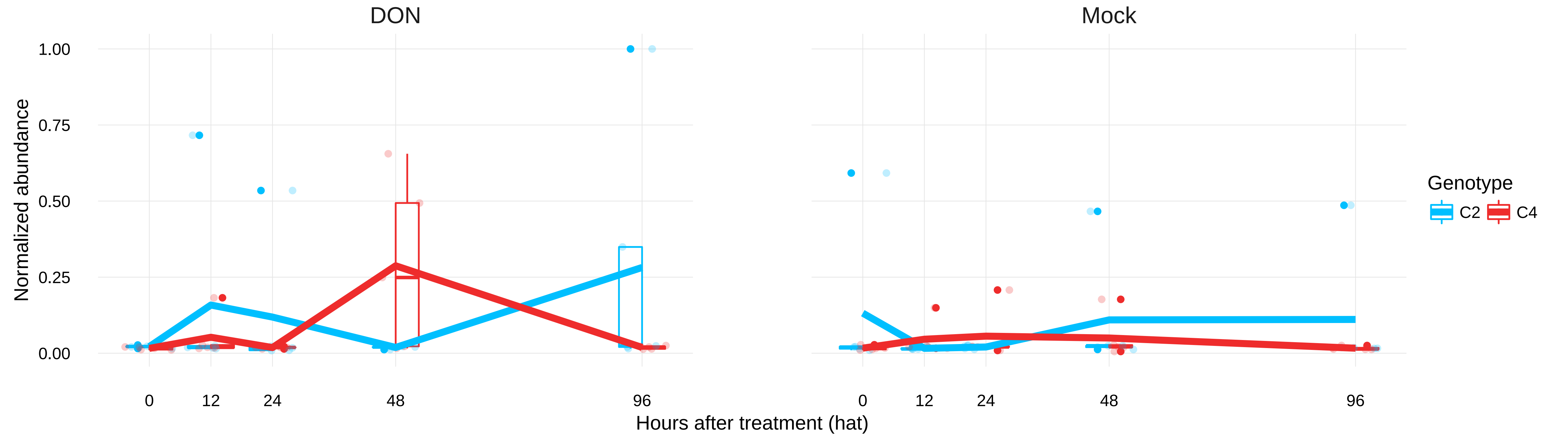

CM, Remus; different treatments

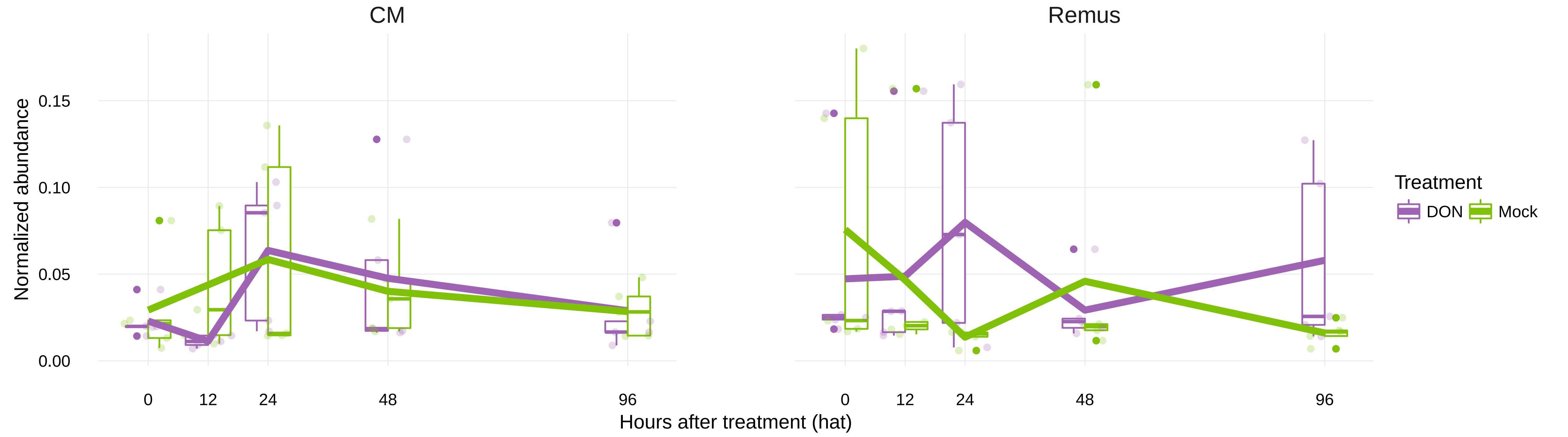

DON, Mock; all four genotypes

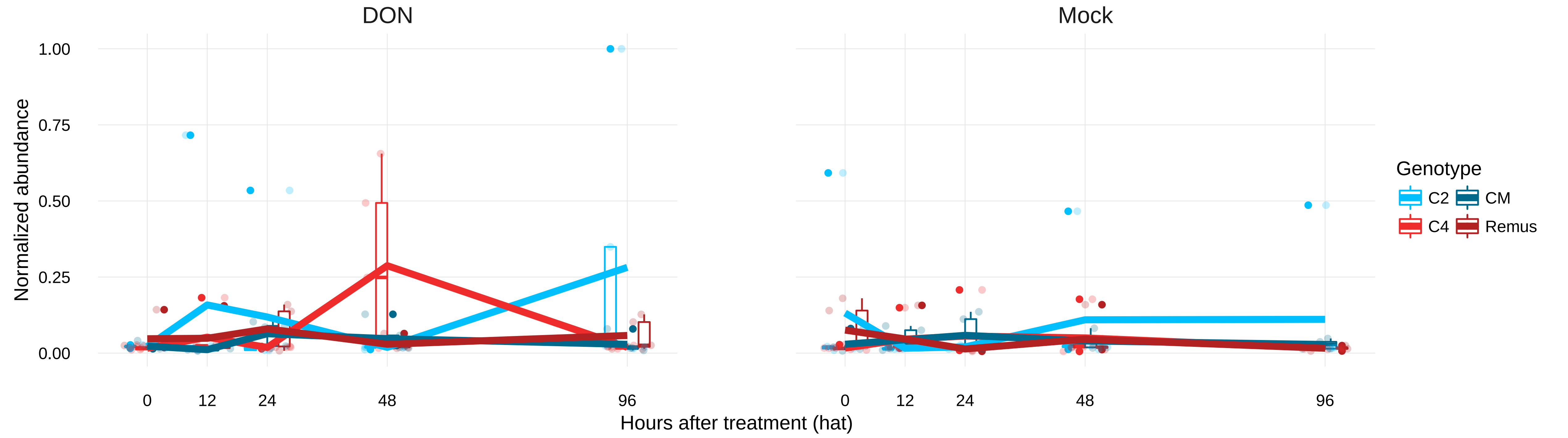

A.113

Annotated as others
(1 database hit)

MZ	125.0596
RT	18 min
Normalization	Directly via KPX samples
Cluster	–
Cn total / Phe	7 /

C2, C4; different treatments

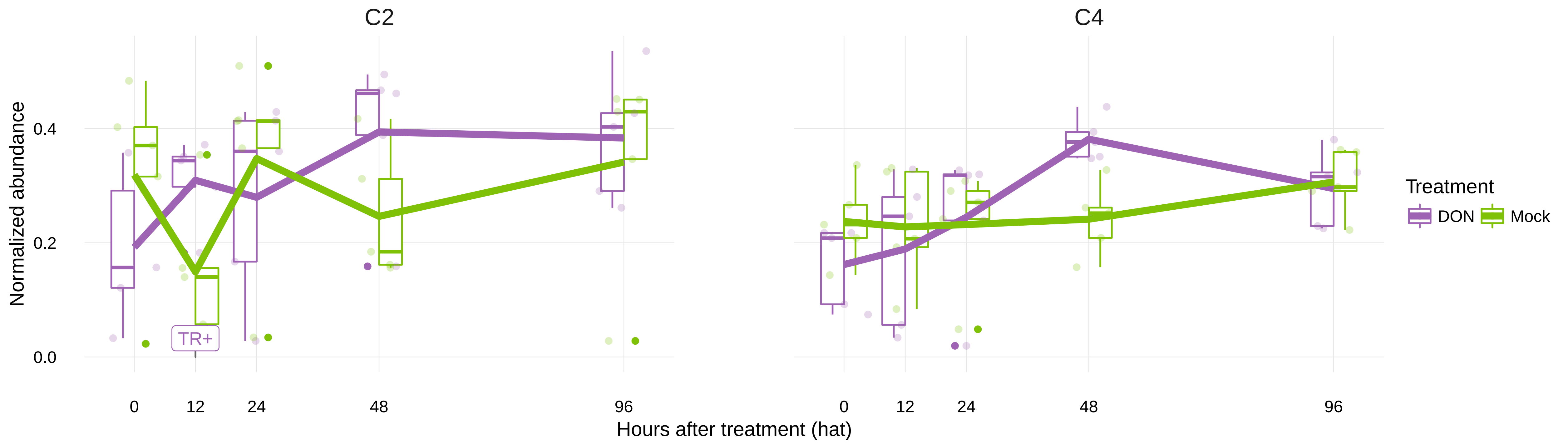

DON, Mock; different genotypes

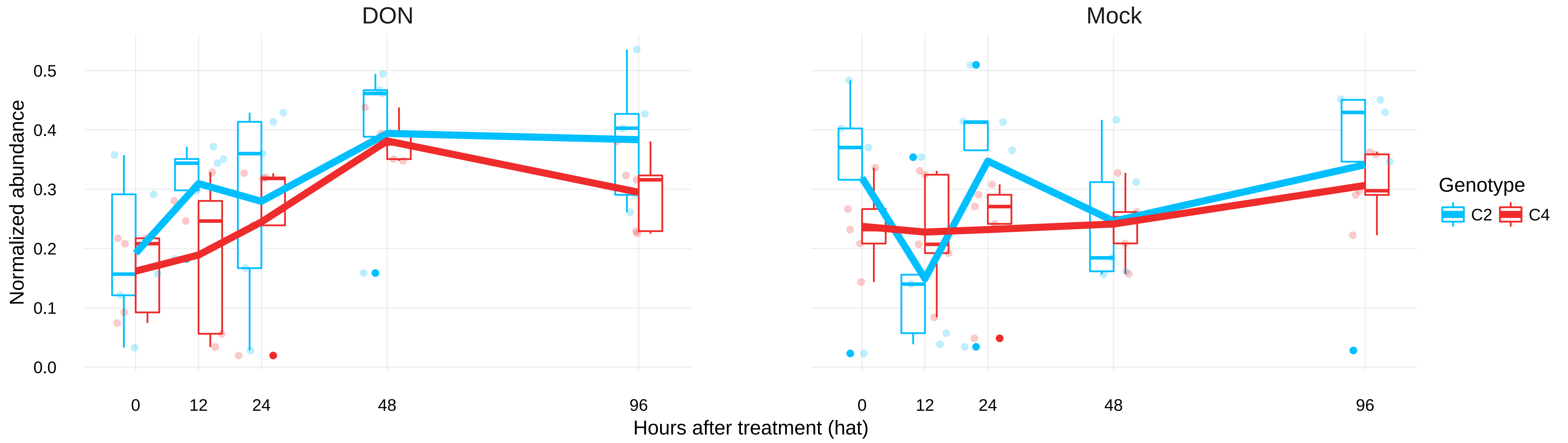

CM, Remus; different treatments

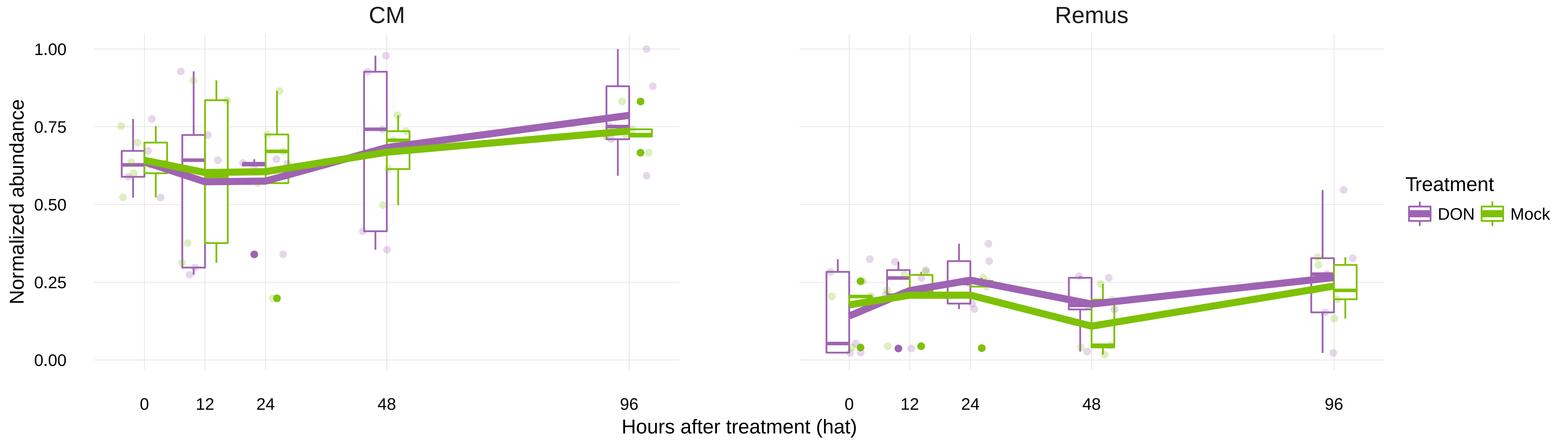

DON, Mock; all four genotypes

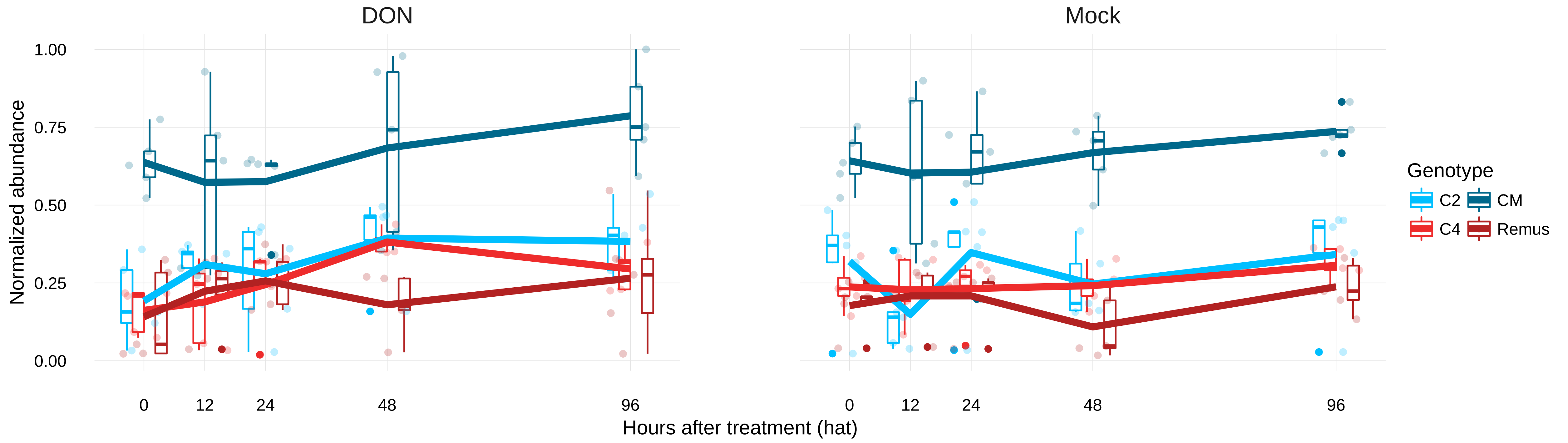

A.34

Annotated as HCAA
(1 database hit)

MZ	277.1667
RT	9.95 min
Normalization	Indirectly via surrogate in the KPX samples
Cluster	–
Cn total / Phe	9 / 9

C2, C4; different treatments

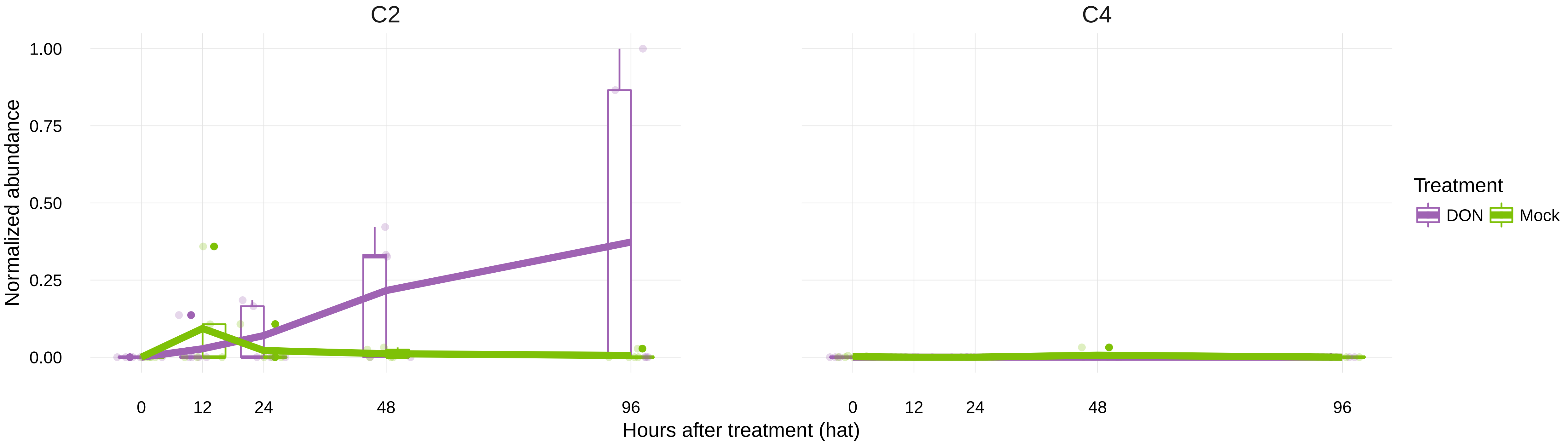

DON, Mock; different genotypes

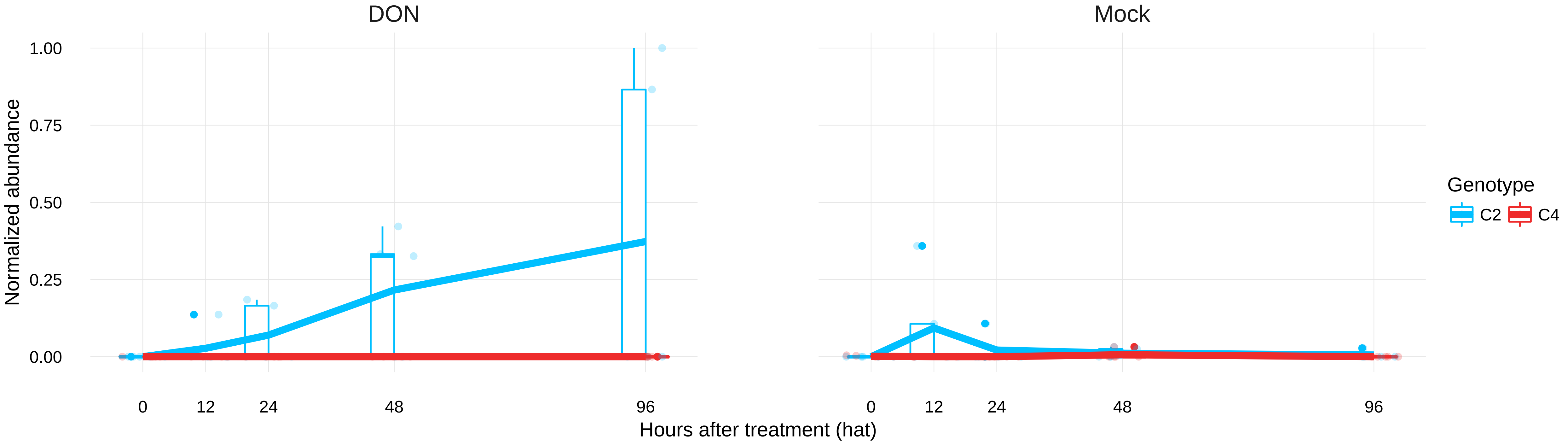

CM, Remus; different treatments

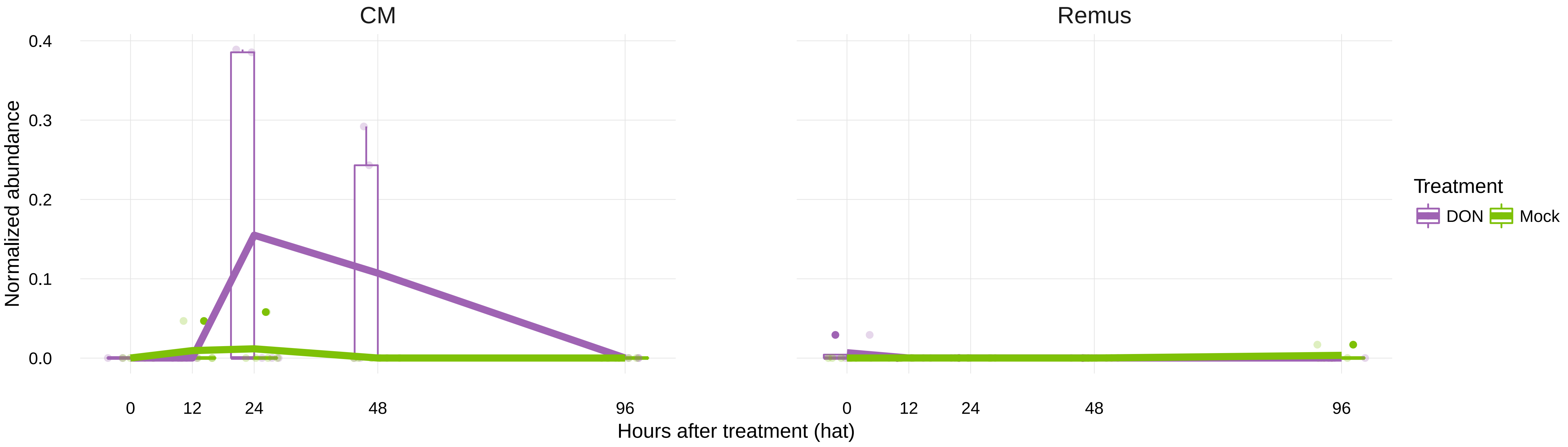

DON, Mock; all four genotypes

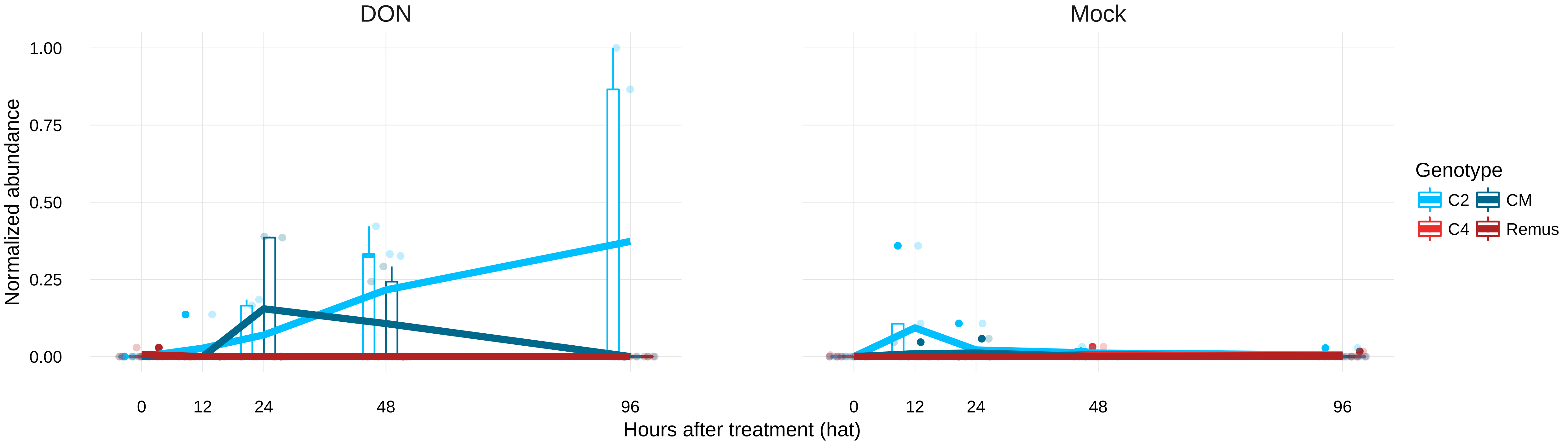

A.126

Annotated as Flavonoid (GlcF)

(1 database hit)

MZ	557.1654
RT	20.5 min
Normalization	Directly via KPX samples
Cluster	–
Cn total / Phe	28 /

C2, C4; different treatments

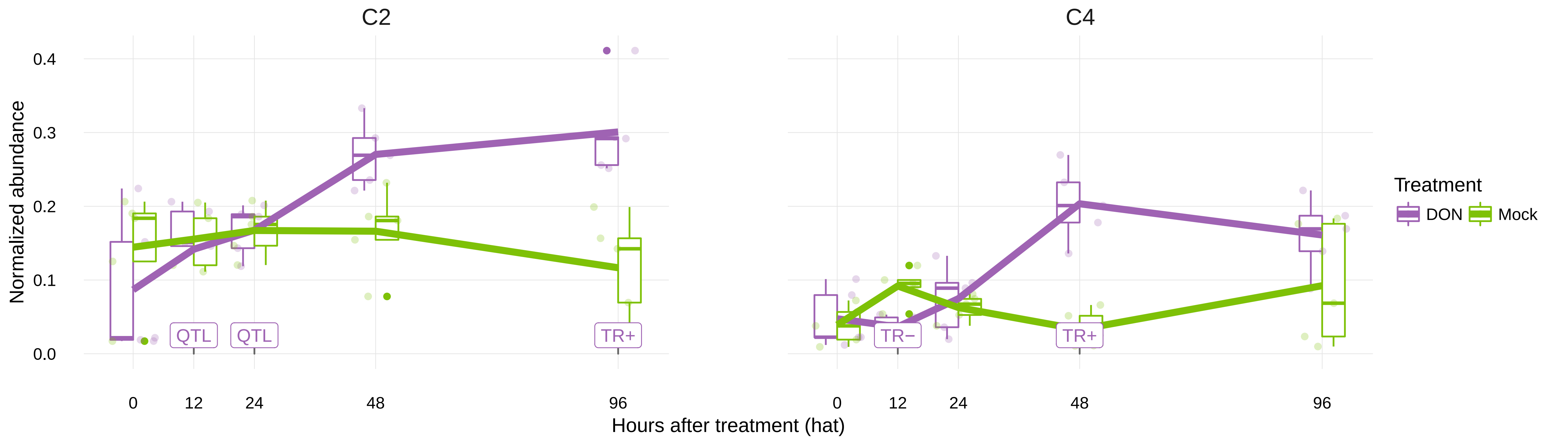

DON, Mock; different genotypes

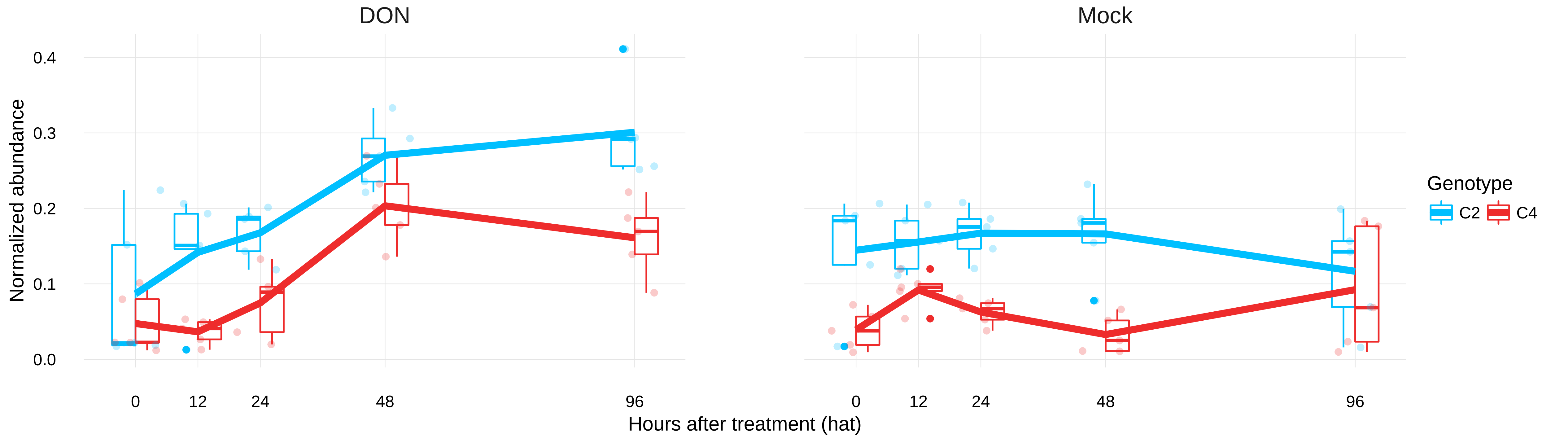

CM, Remus; different treatments

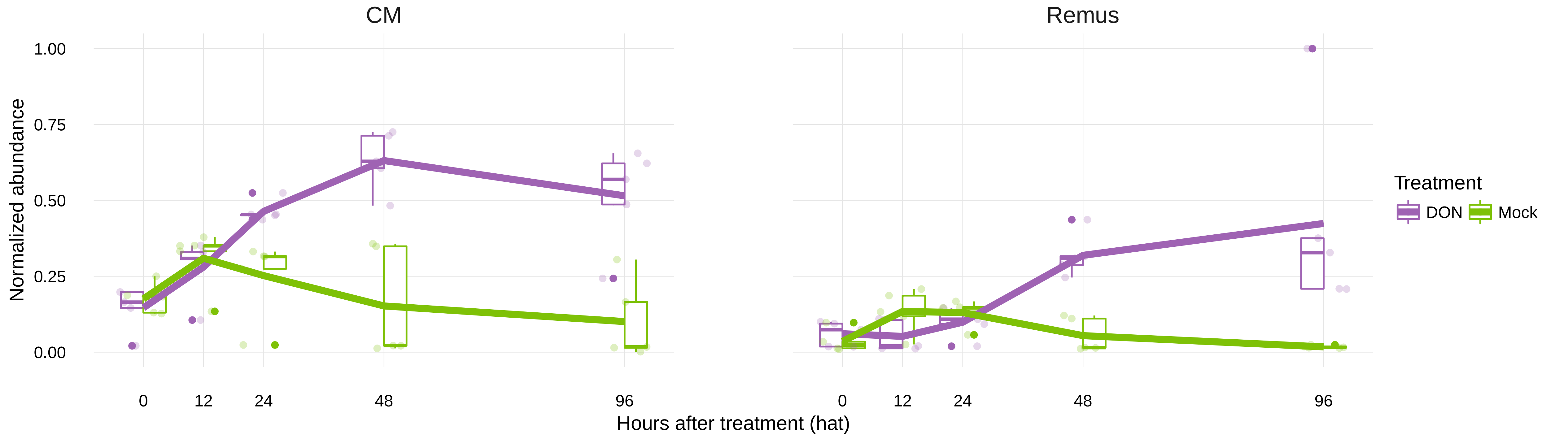

DON, Mock; all four genotypes

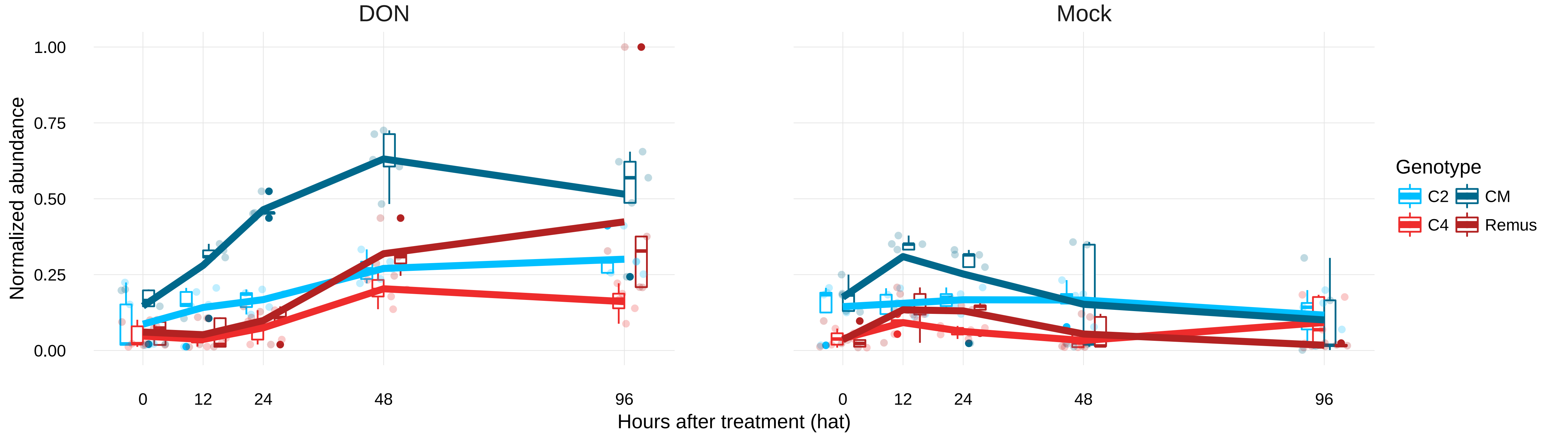

A.8

Annotated as Putative phenylacetic acid derivative
(1 database hit)

MZ	122.0965
RT	5.11 min
Normalization	Not normalized
Cluster	–
Cn total / Phe	8 / 8

C2, C4; different treatments

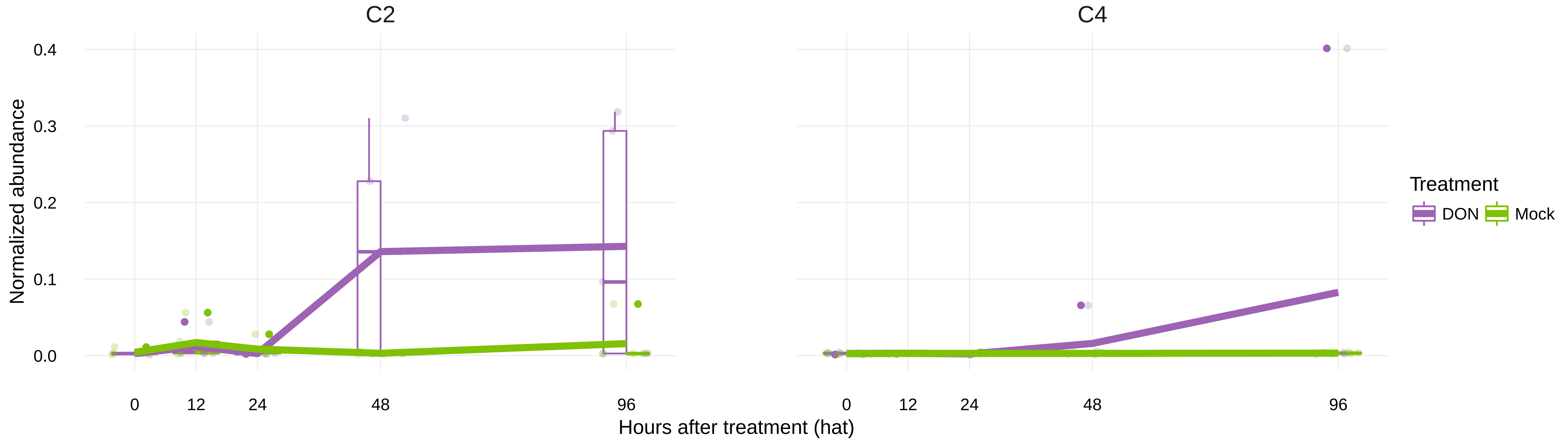

DON, Mock; different genotypes

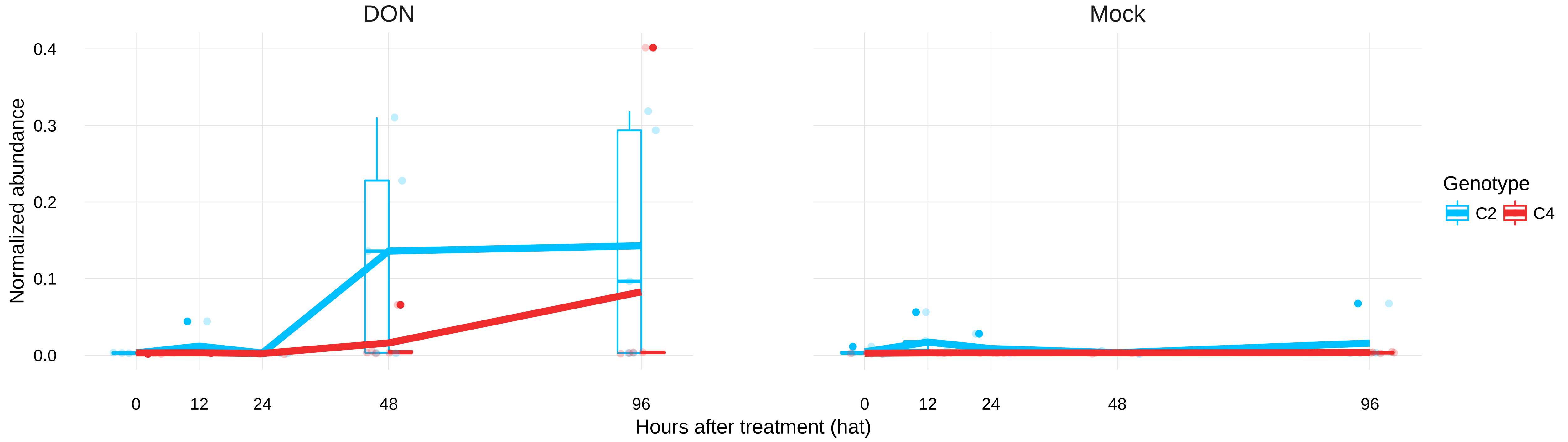

CM, Remus; different treatments

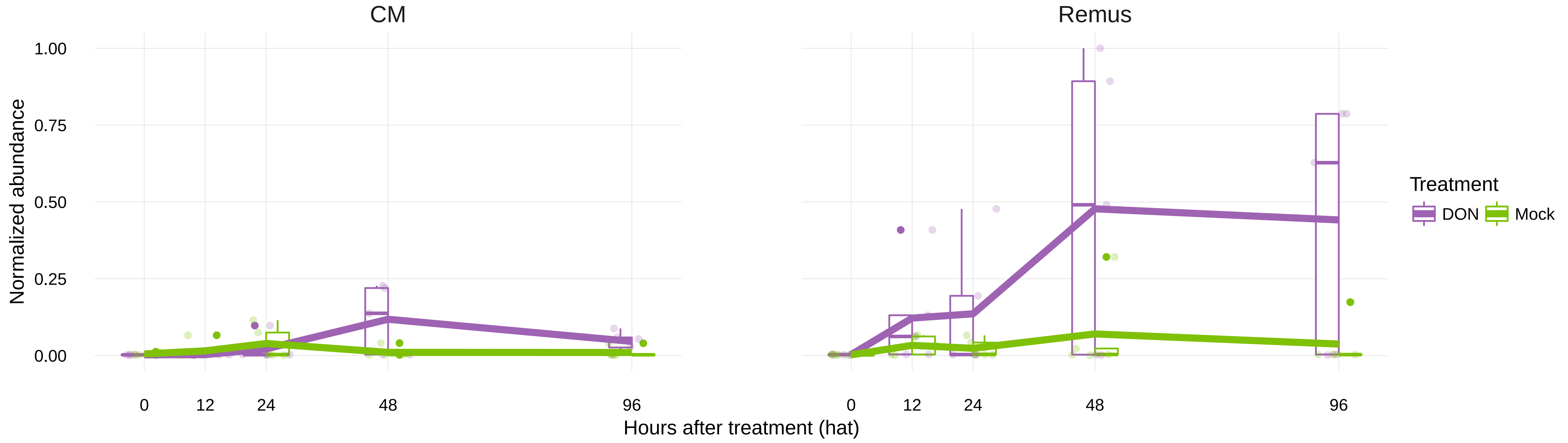

DON, Mock; all four genotypes

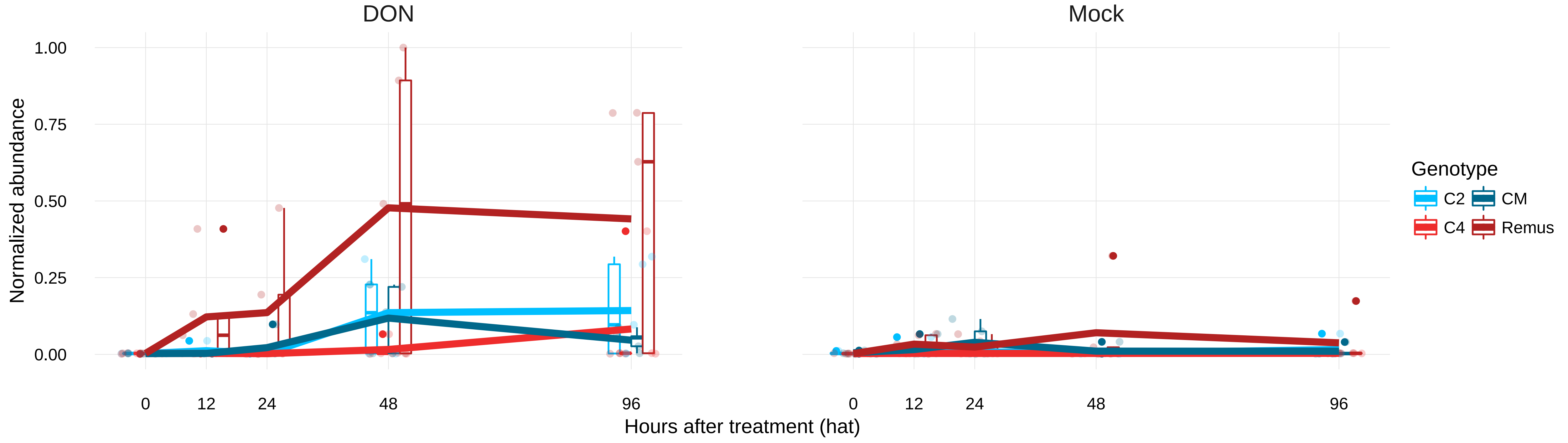

A.100

Annotated as Flavonoid
(21 database hits)

MZ	371.149
RT	16.65 min
Normalization	Indirectly via surrogate in the KPX samples
Cluster	–
Cn total / Phe	21 /

C2, C4; different treatments

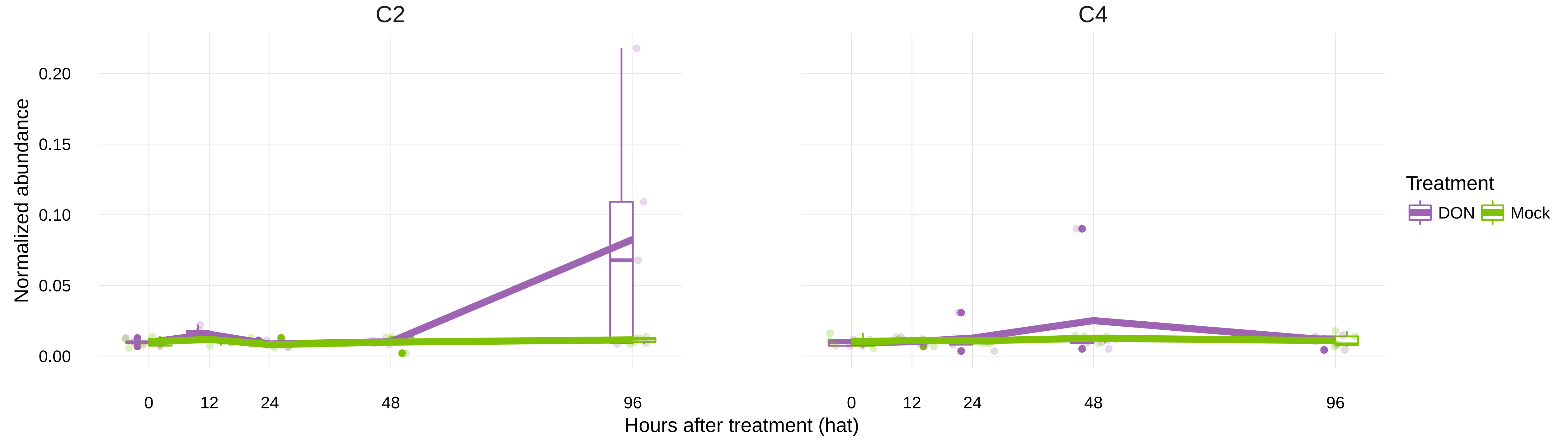

DON, Mock; different genotypes

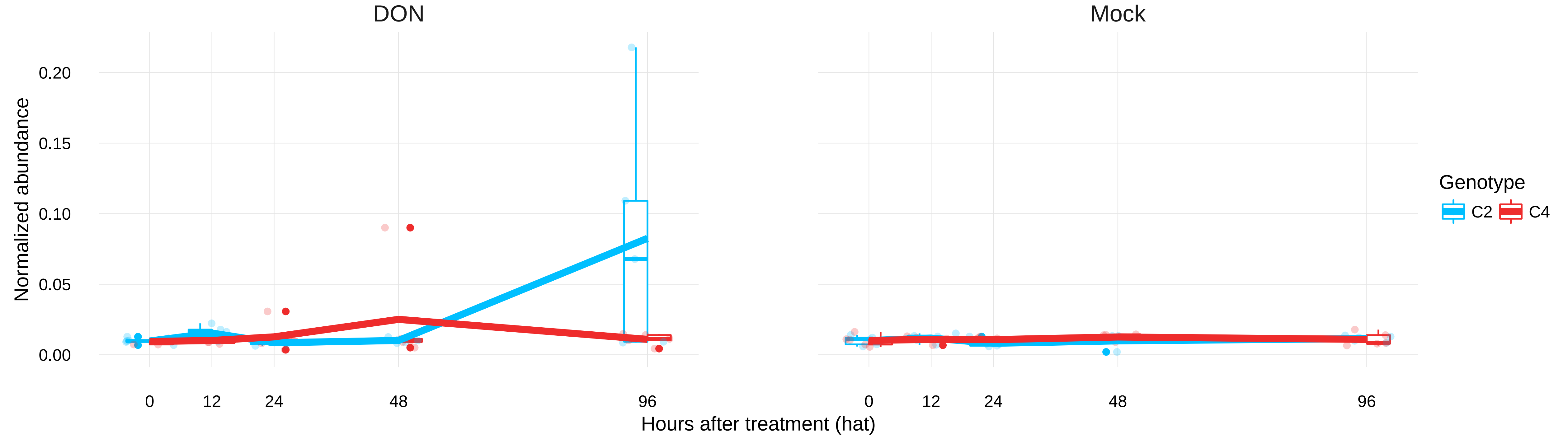

CM, Remus; different treatments

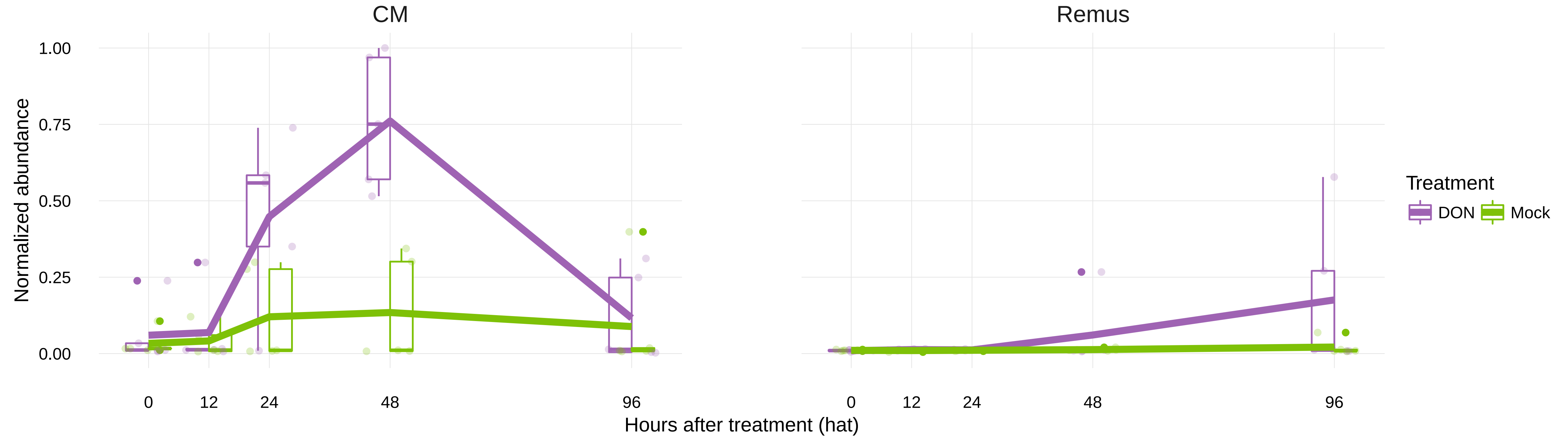

DON, Mock; all four genotypes

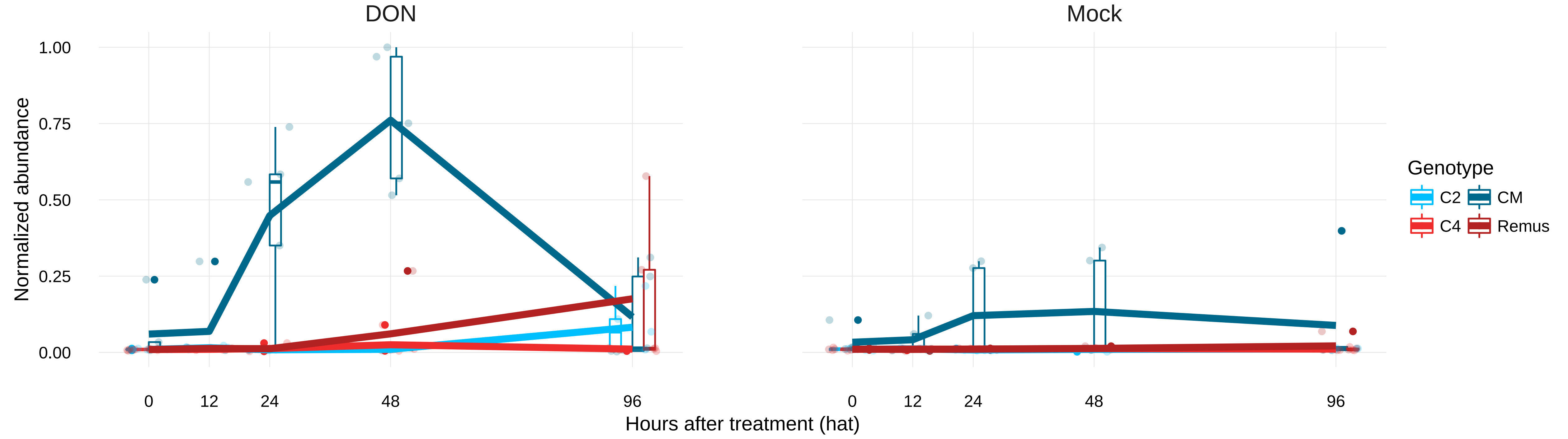

A.71

Annotated as Flavonoid, Lignan or HCA derivative
(15 database hits)

MZ	359.1492
RT	13.64 min
Normalization	Indirectly via surrogate in the KPX samples
Cluster	–
Cn total / Phe	20 / 9

C2, C4; different treatments

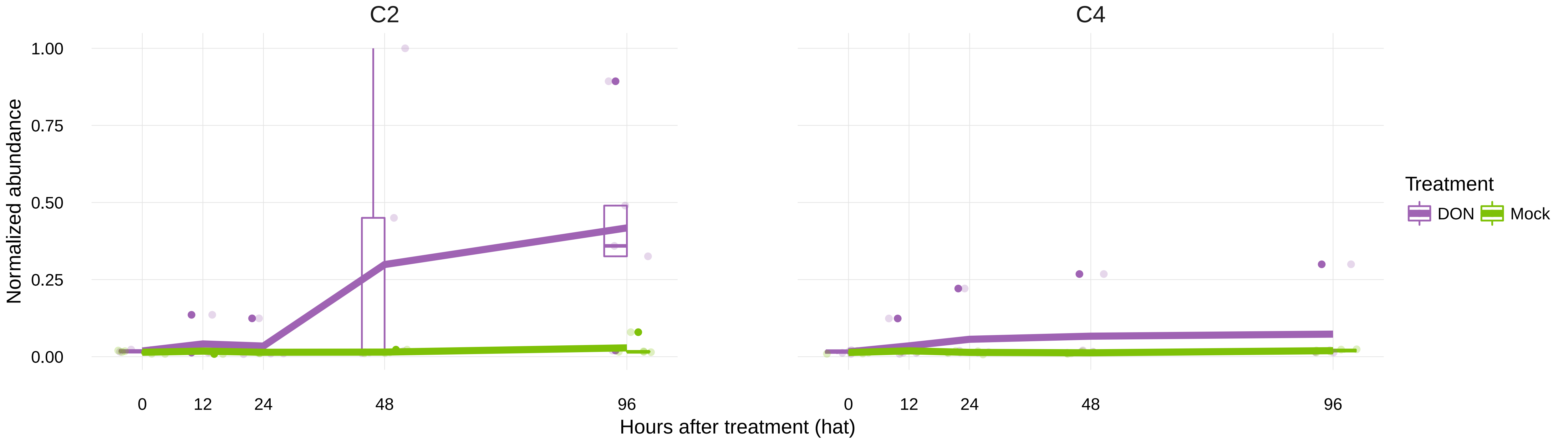

DON, Mock; different genotypes

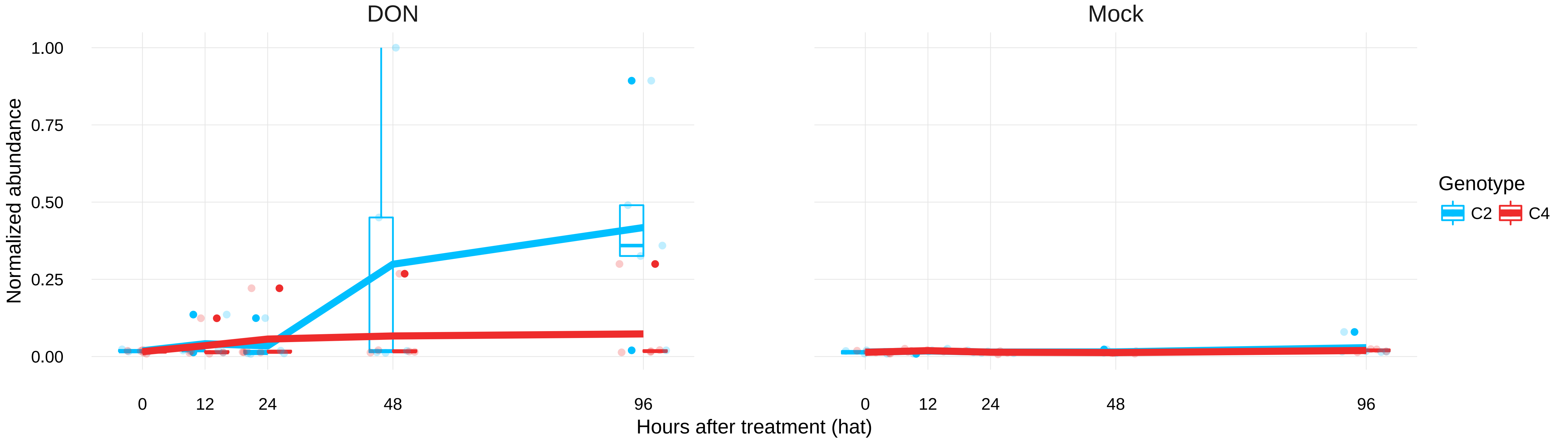

CM, Remus; different treatments

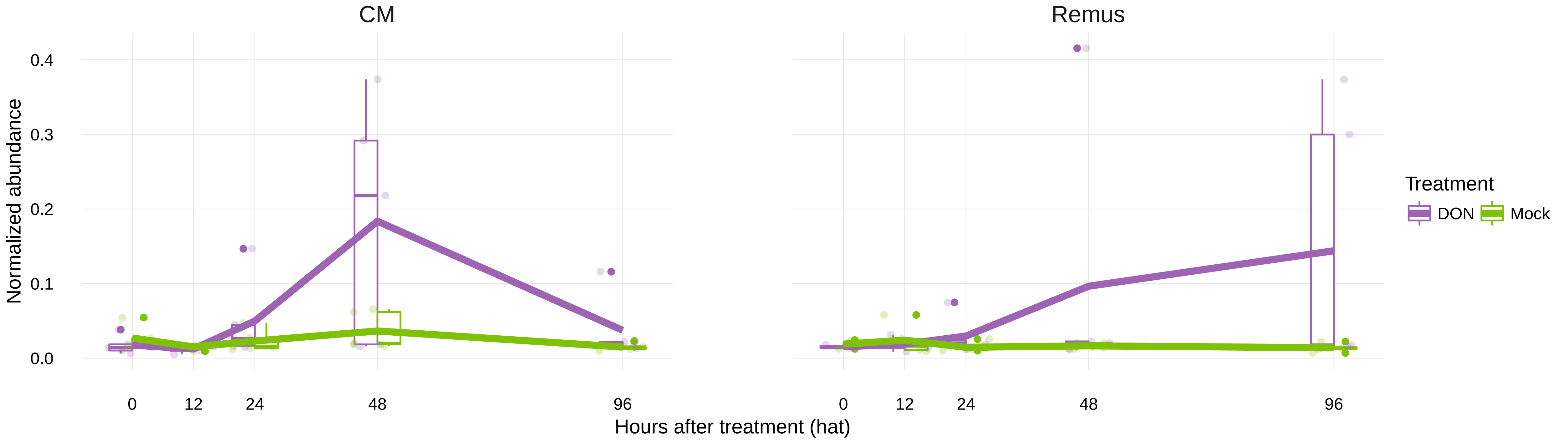

DON, Mock; all four genotypes

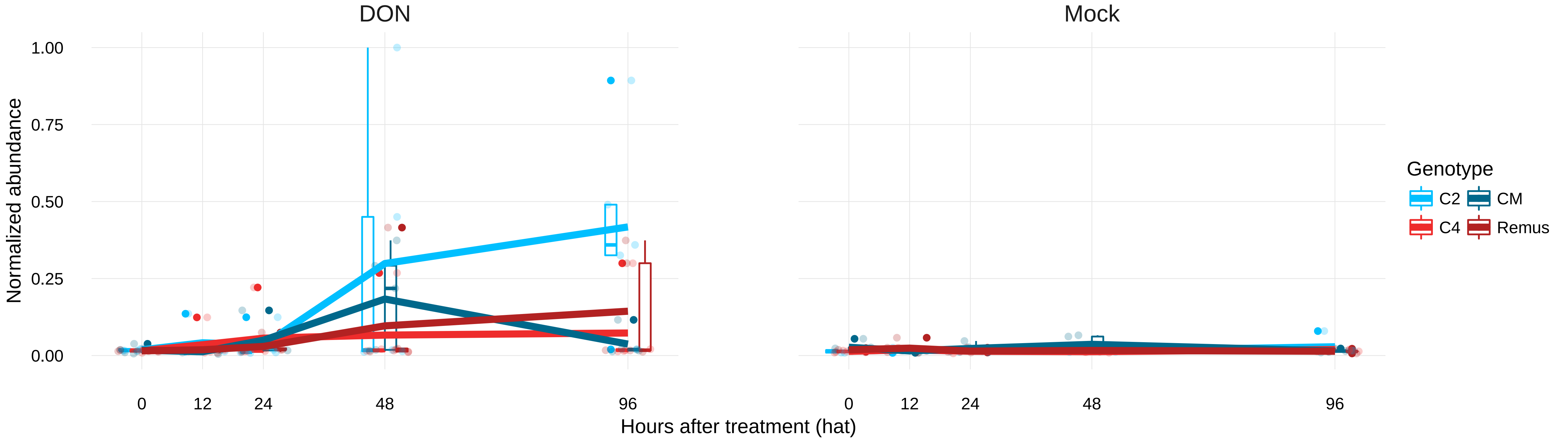

A.121

Annotated as Flavonoid (GlcF)
(17 database hits)

MZ	773.1922
RT	19.63 min
Normalization	Directly via KPX samples
Cluster	–
Cn total / Phe	36 /

C2, C4; different treatments

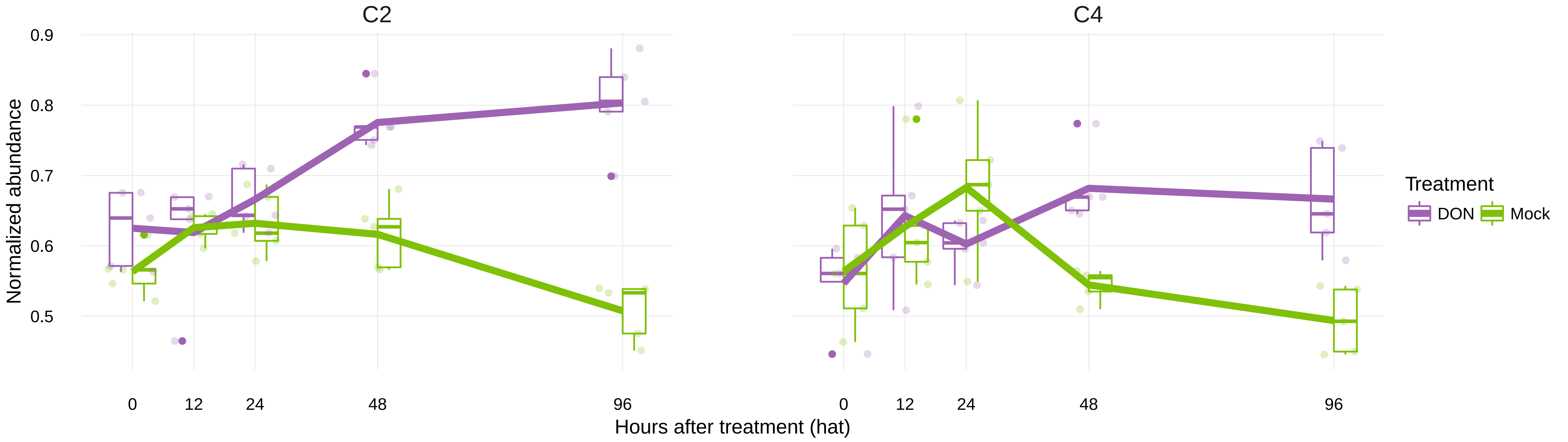

DON, Mock; different genotypes

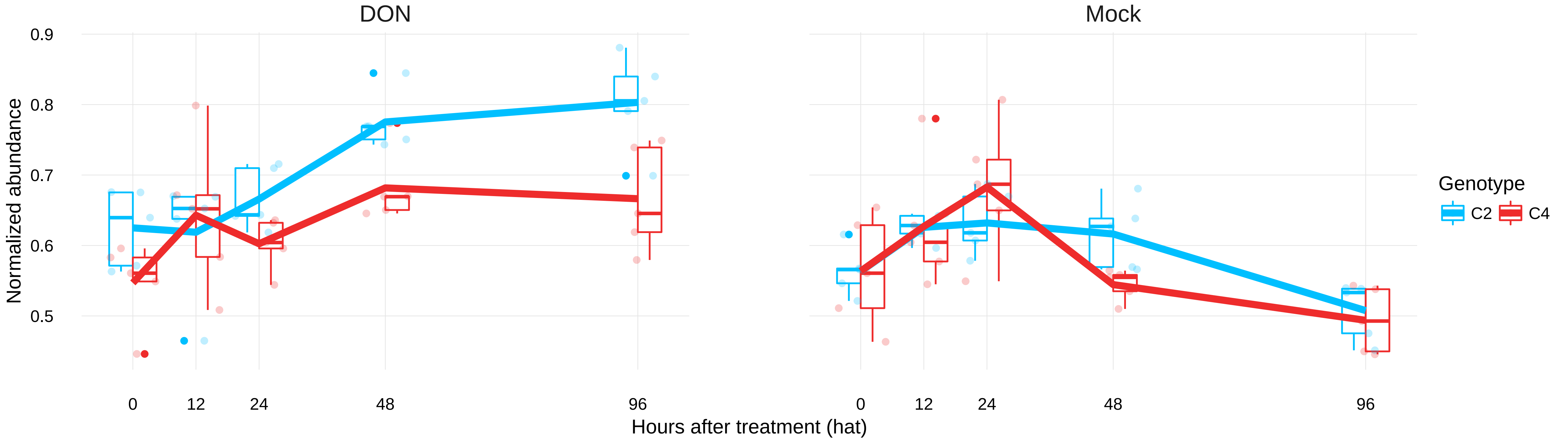

CM, Remus; different treatments

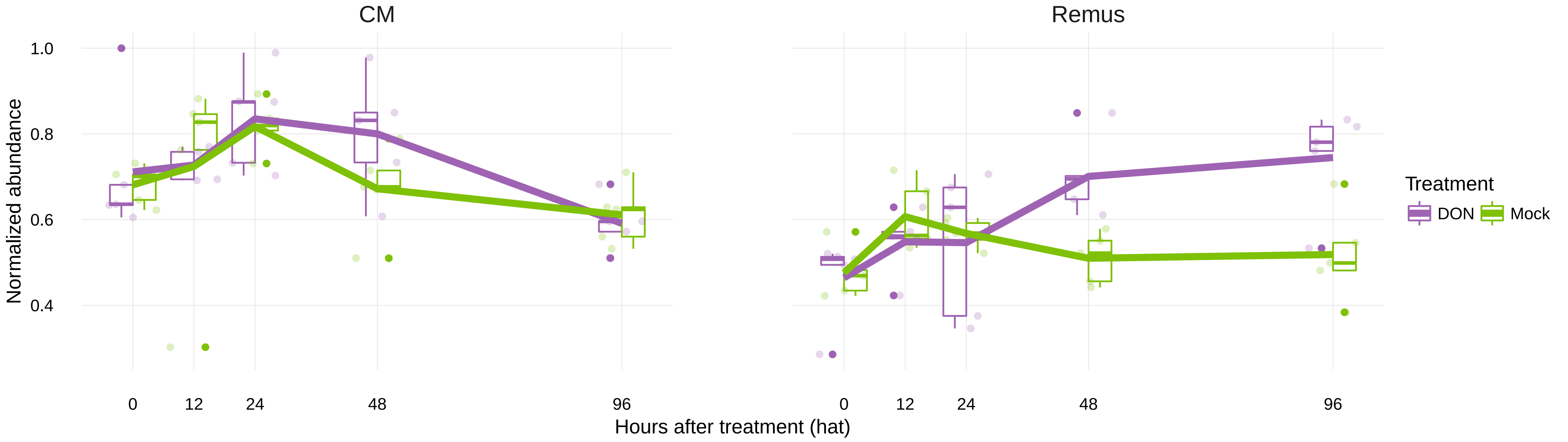

DON, Mock; all four genotypes

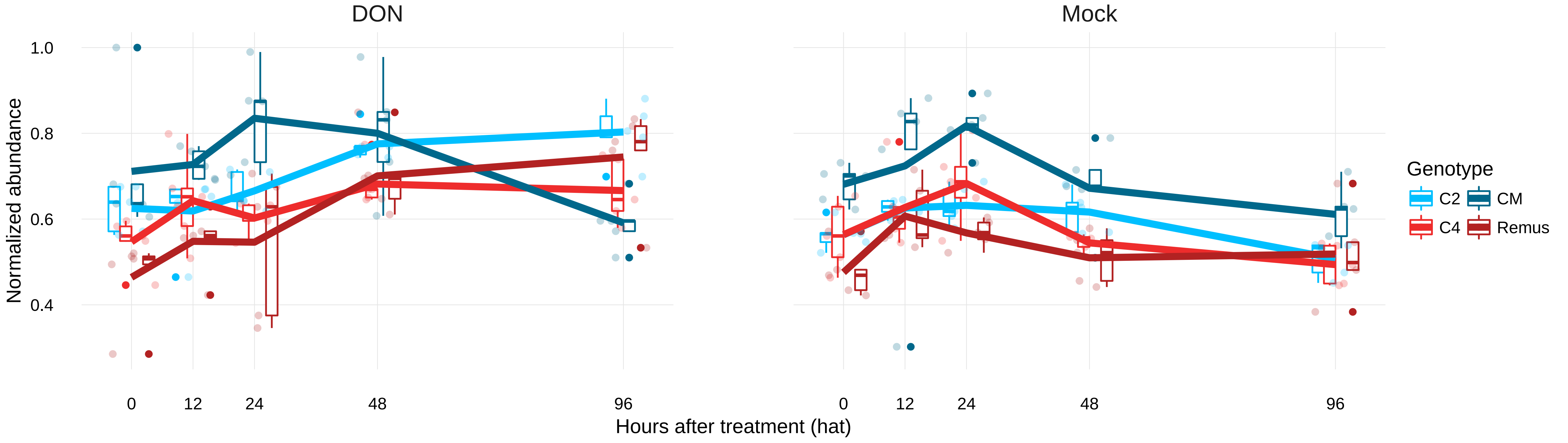

U.12

Unknown Phe−derived wheat−metabolite

MZ	579.1345
RT	18.66 min
Normalization	Directly via KPX samples
Cluster	−
Cn total / Phe	26 / 18

C2, C4; different treatments

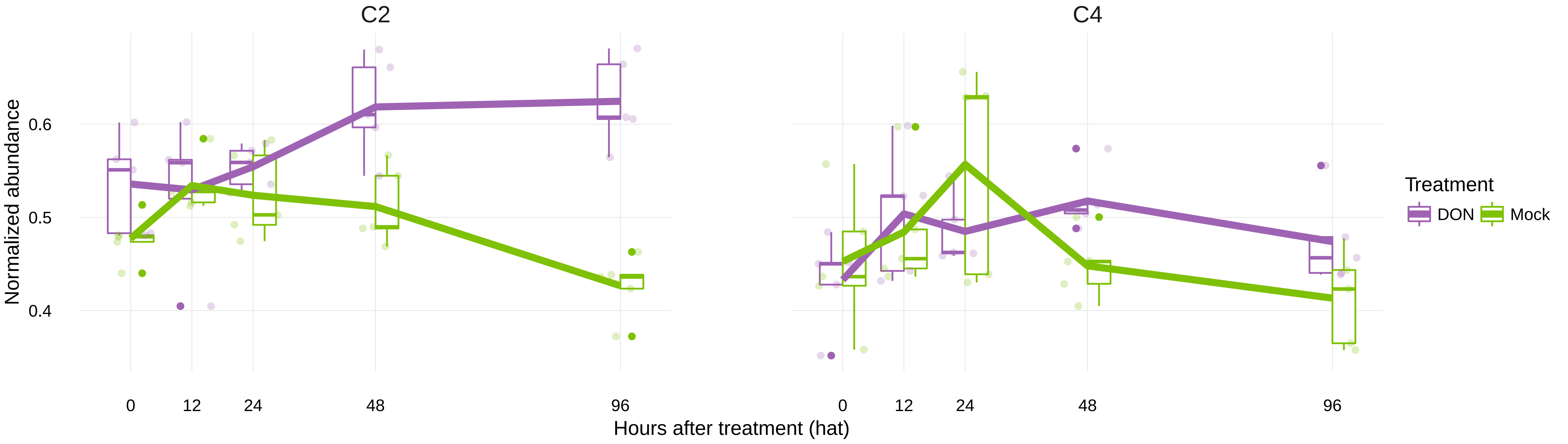

DON, Mock; different genotypes

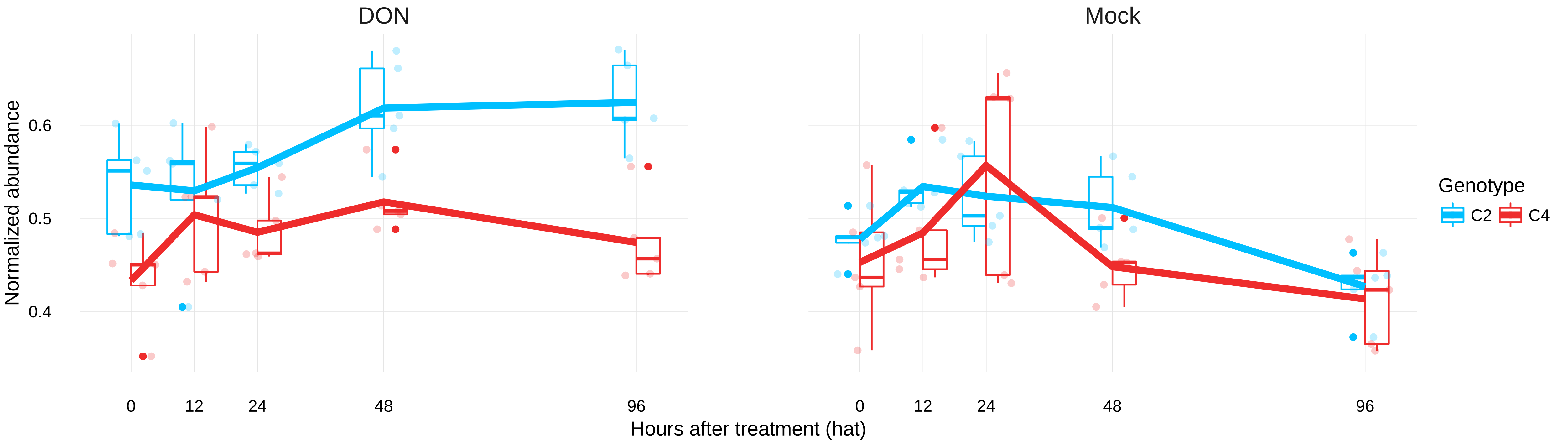

CM, Remus; different treatments

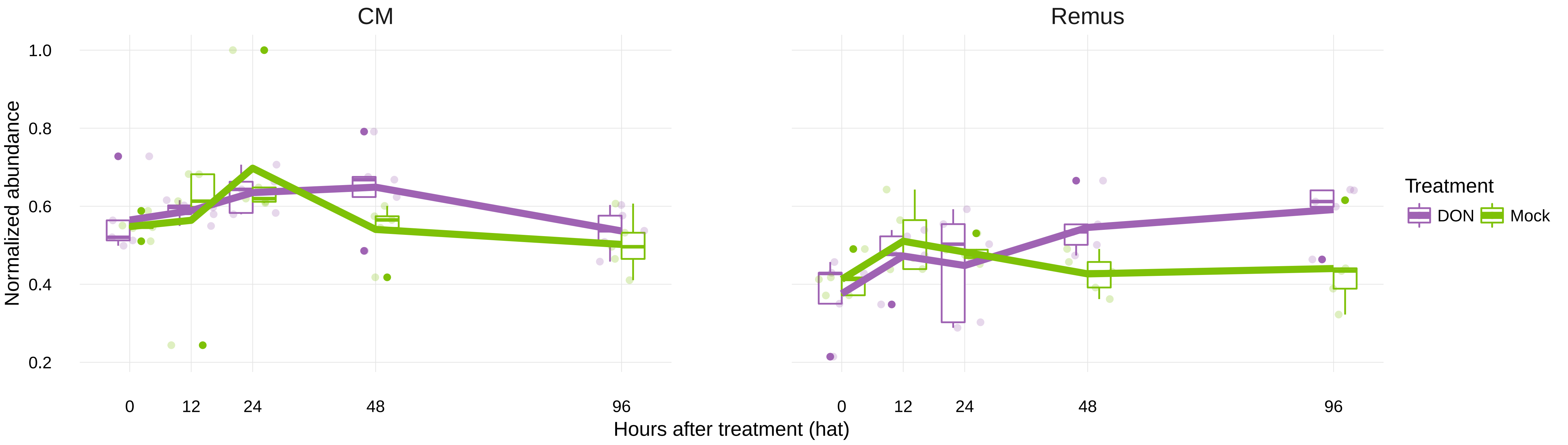

DON, Mock; all four genotypes

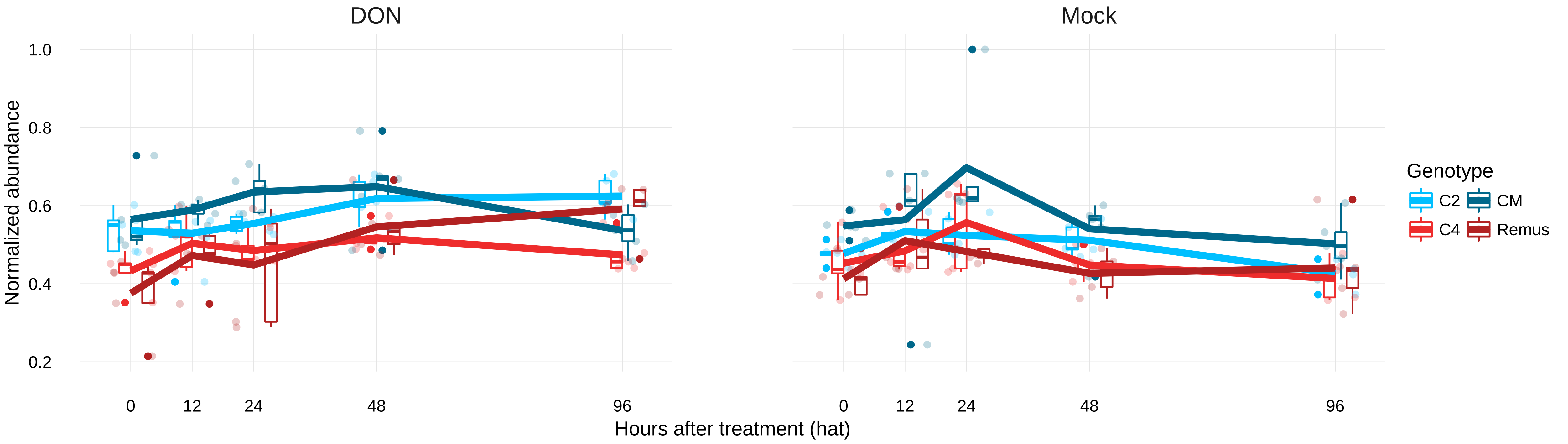

A.120

Annotated as Flavonoid (GlcF)
(17 database hits)

MZ	507.1498
RT	19.45 min
Normalization	Directly via KPX samples
Cluster	Cluster 4
Cn total / Phe	24 /

C2, C4; different treatments

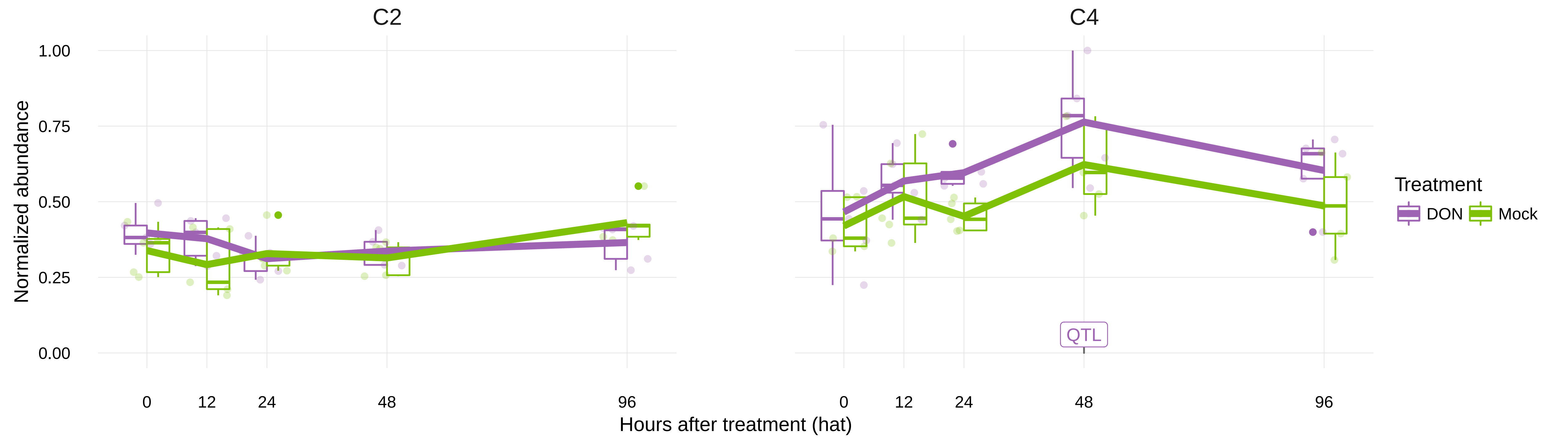

DON, Mock; different genotypes

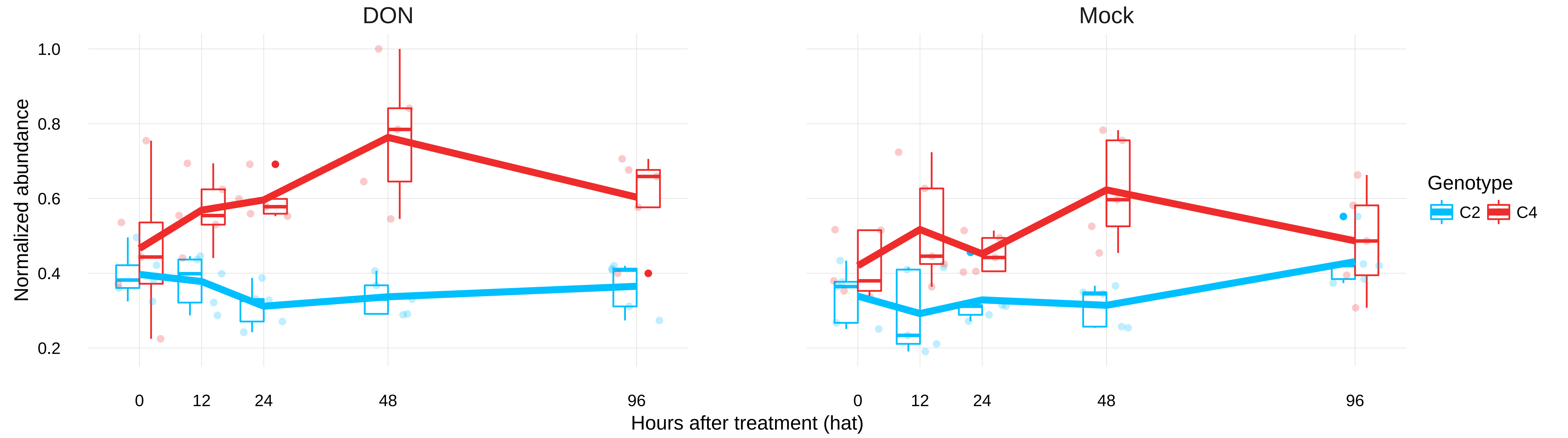

CM, Remus; different treatments

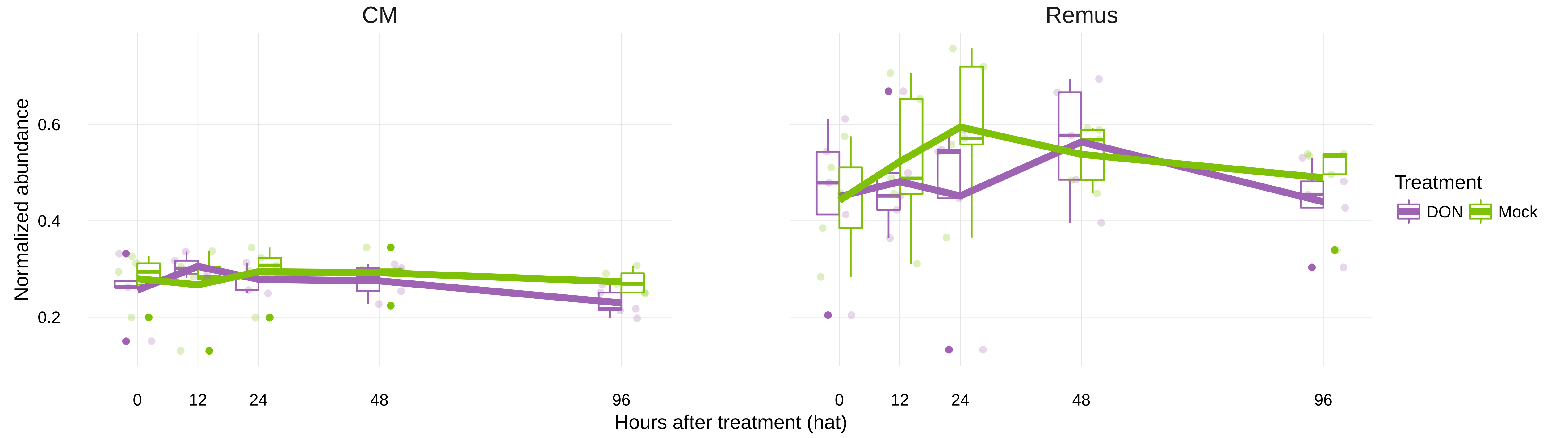

DON, Mock; all four genotypes

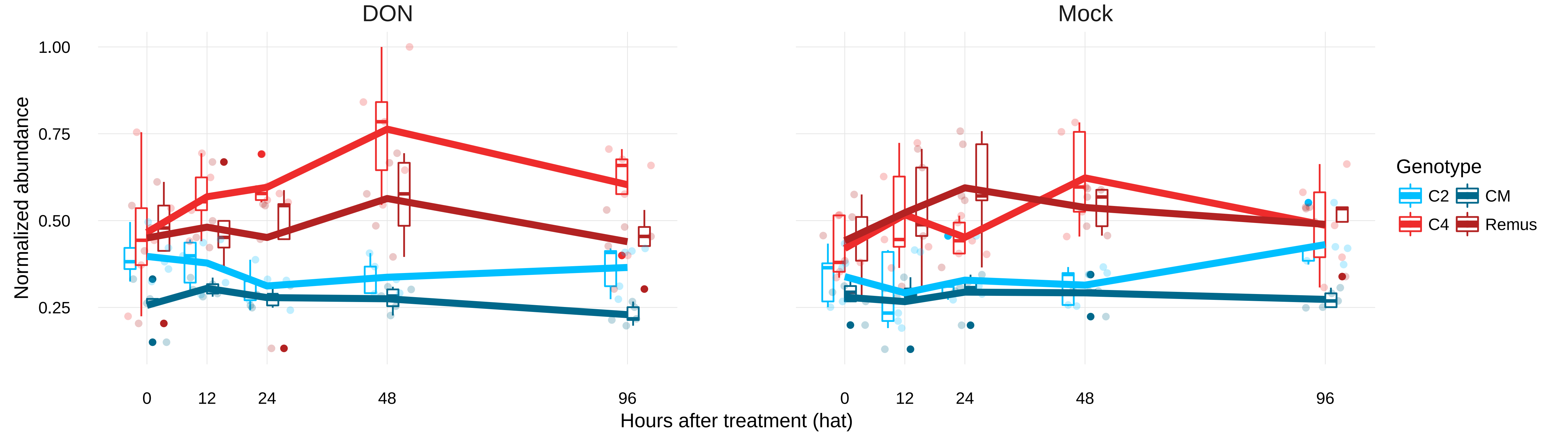

Vitexin

Identified metabolite (level 1)

MZ	433.1131
RT	14.63 min
Normalization	Directly via KPX samples
Cluster	Cluster 4
Cn total / Phe	21 /

C2, C4; different treatments

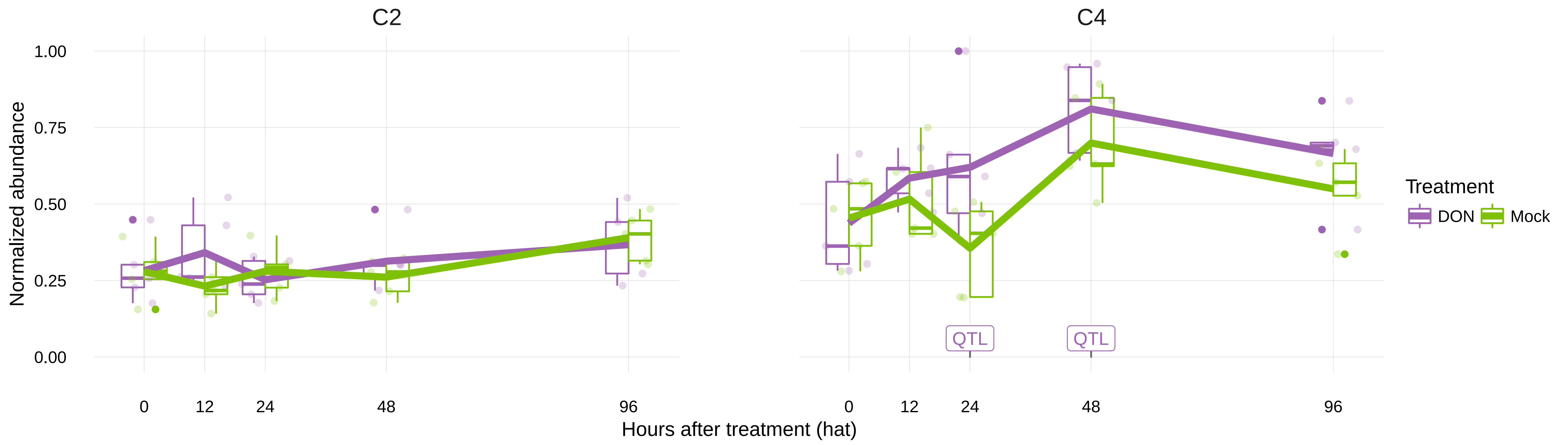

DON, Mock; different genotypes

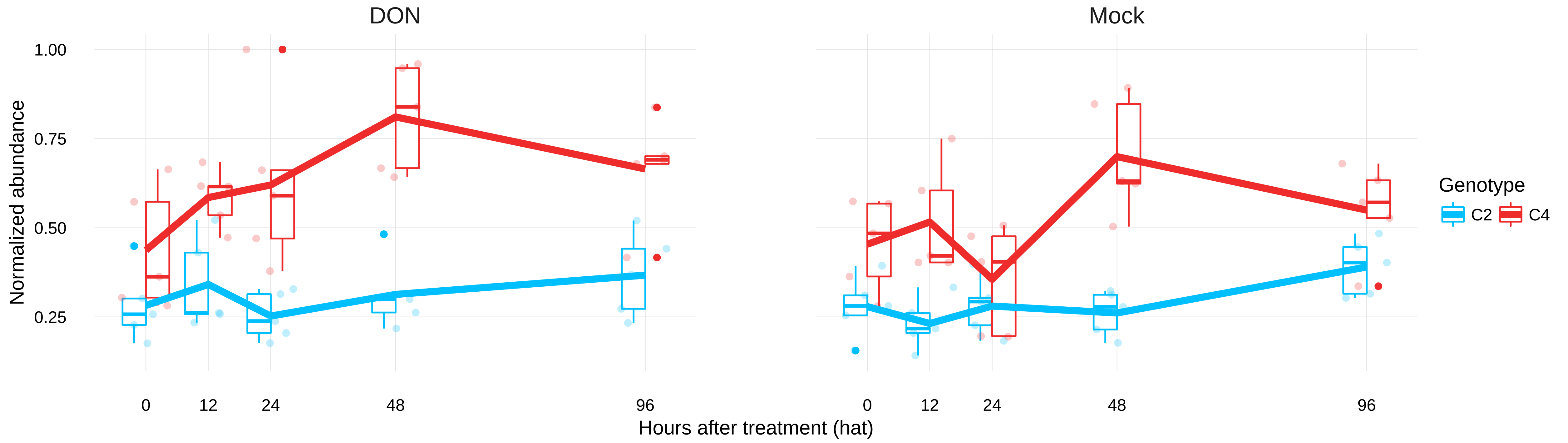

CM, Remus; different treatments

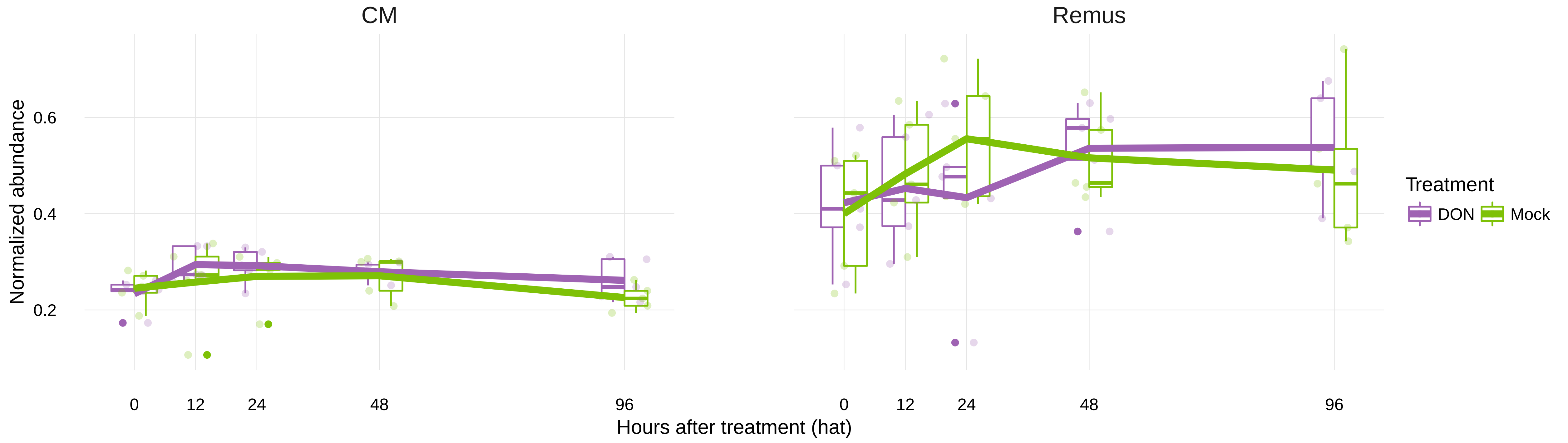

DON, Mock; all four genotypes

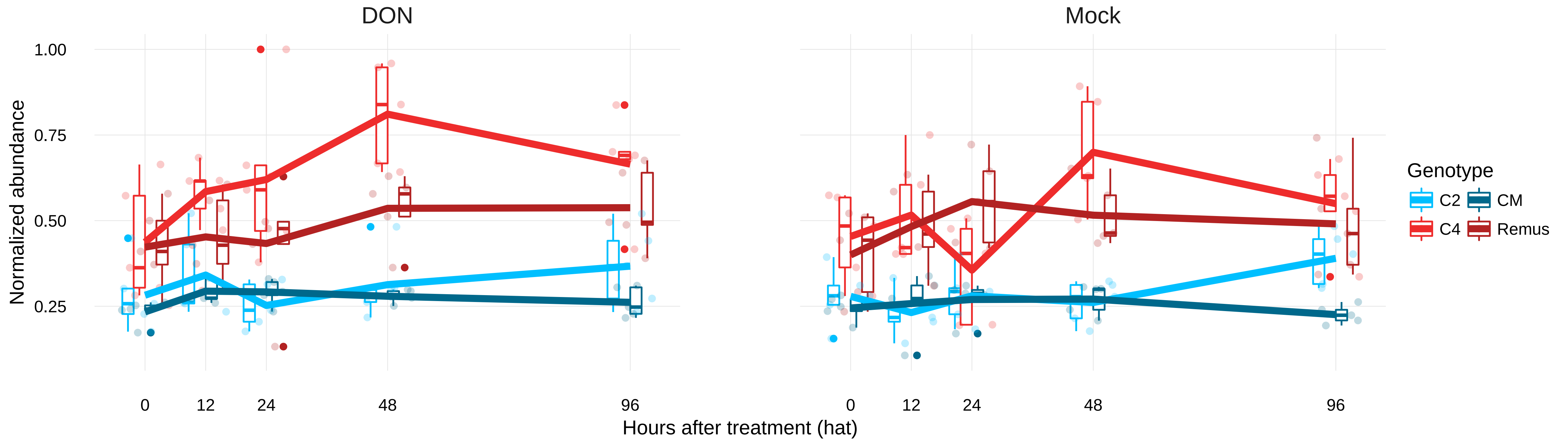

A.92

Annotated as Flavonoid (diGlcF)

(52 database hits)

MZ	609.1815
RT	15.69 min
Normalization	Directly via KPX samples
Cluster	Cluster 4
Cn total / Phe	28 /

C2, C4; different treatments

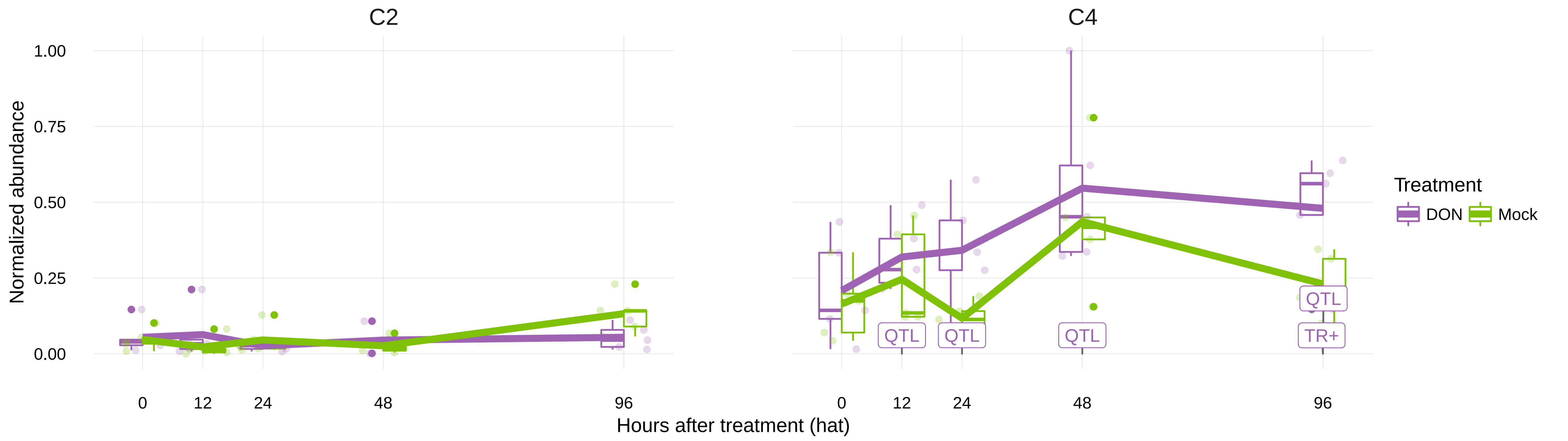

DON, Mock; different genotypes

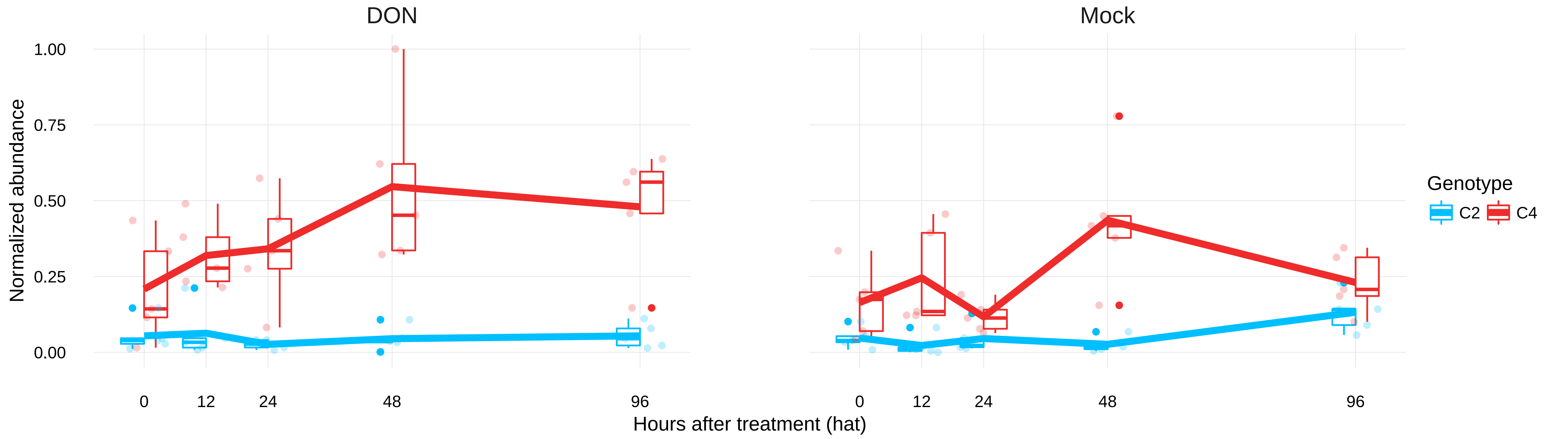

CM, Remus; different treatments

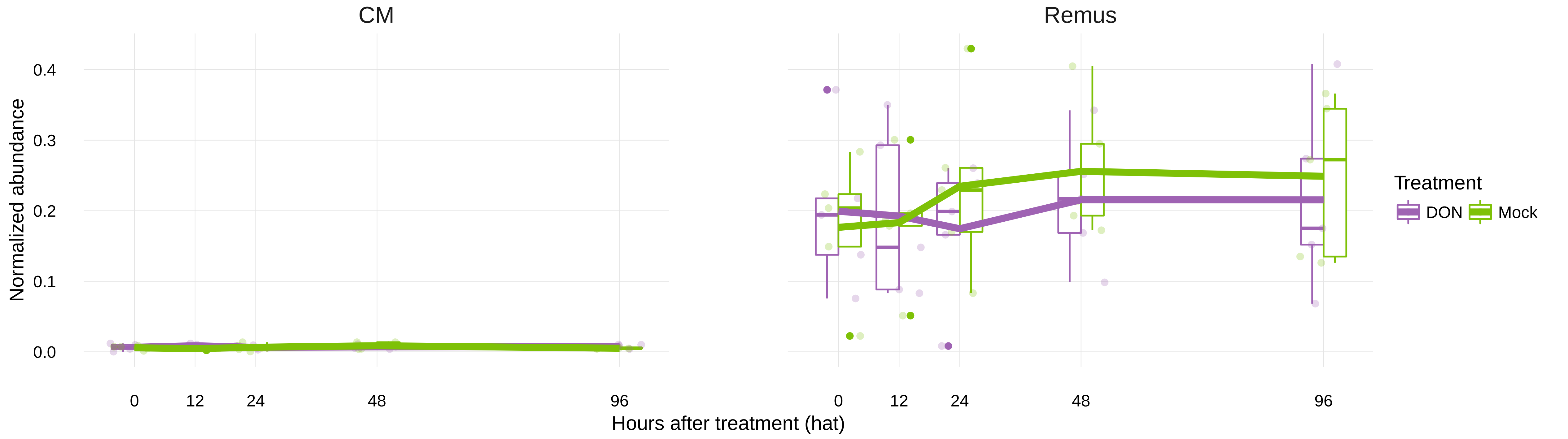

DON, Mock; all four genotypes

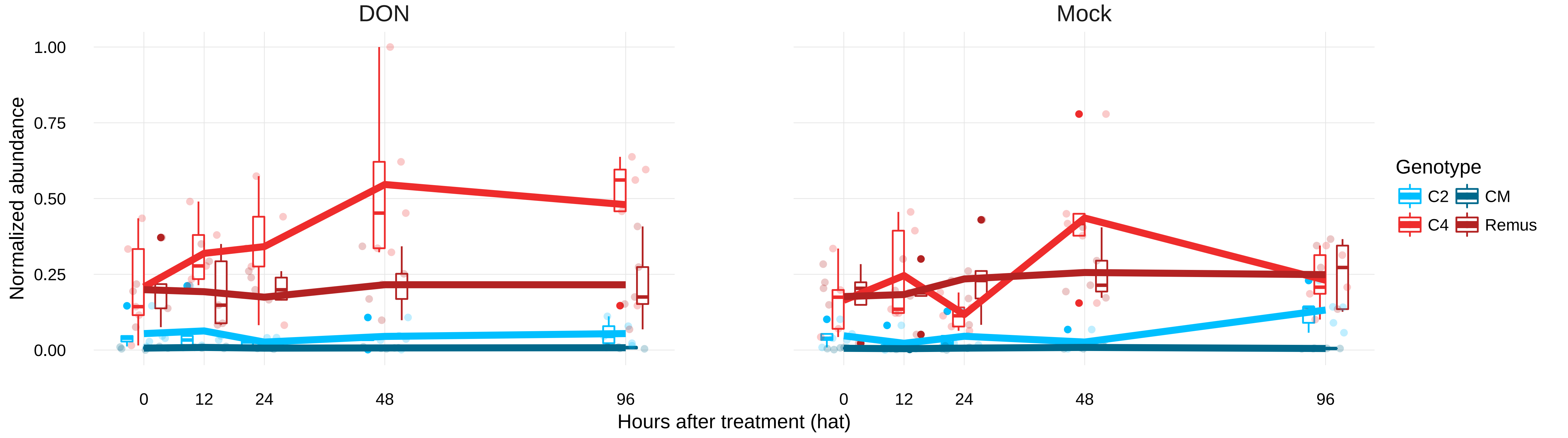

Homoorientin

Identified metabolite (level 1)

MZ	449.1081
RT	13.88 min
Normalization	Directly via KPX samples
Cluster	Cluster 4
Cn total / Phe	21 /

C2, C4; different treatments

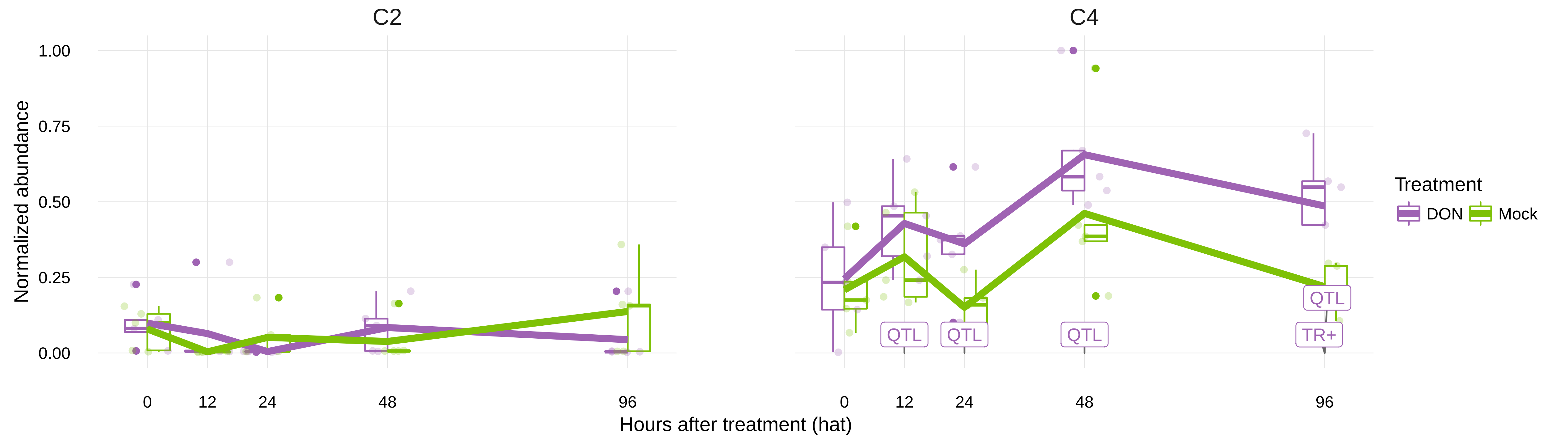

DON, Mock; different genotypes

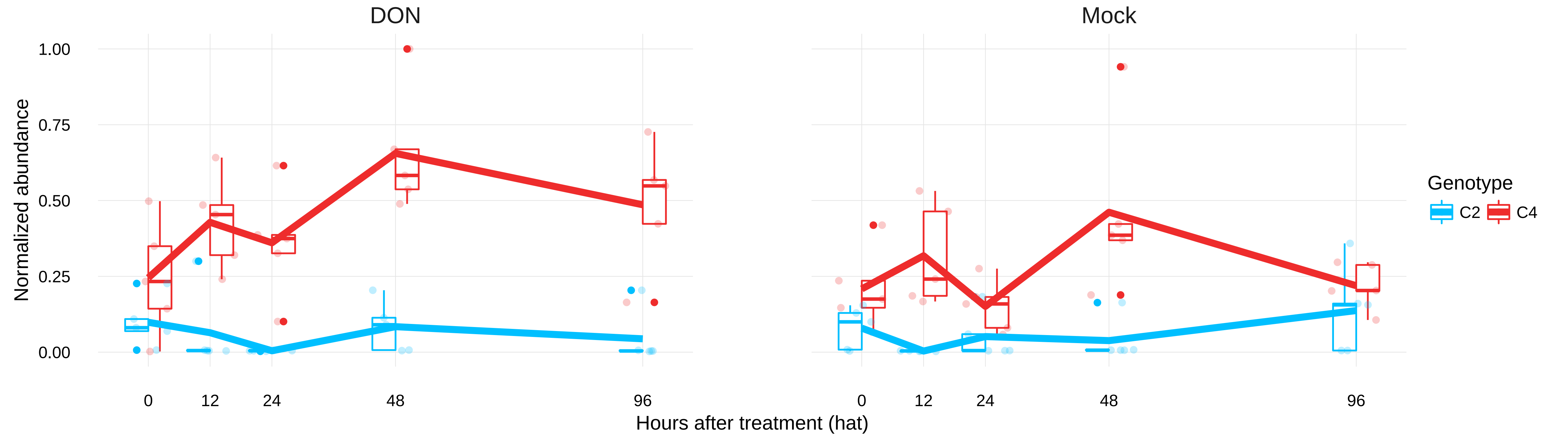

CM, Remus; different treatments

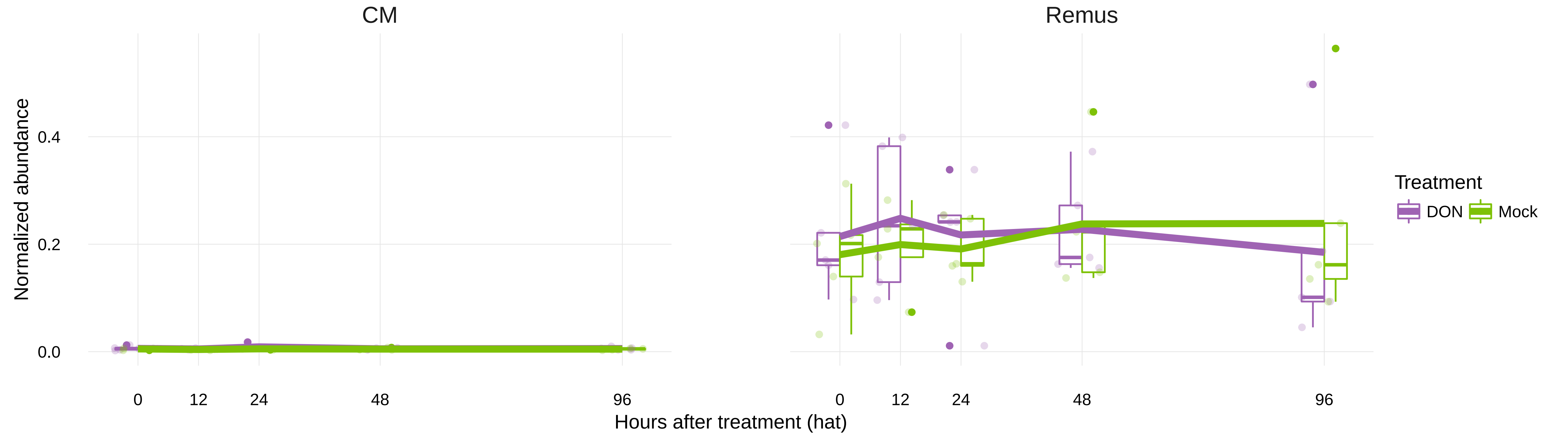

DON, Mock; all four genotypes

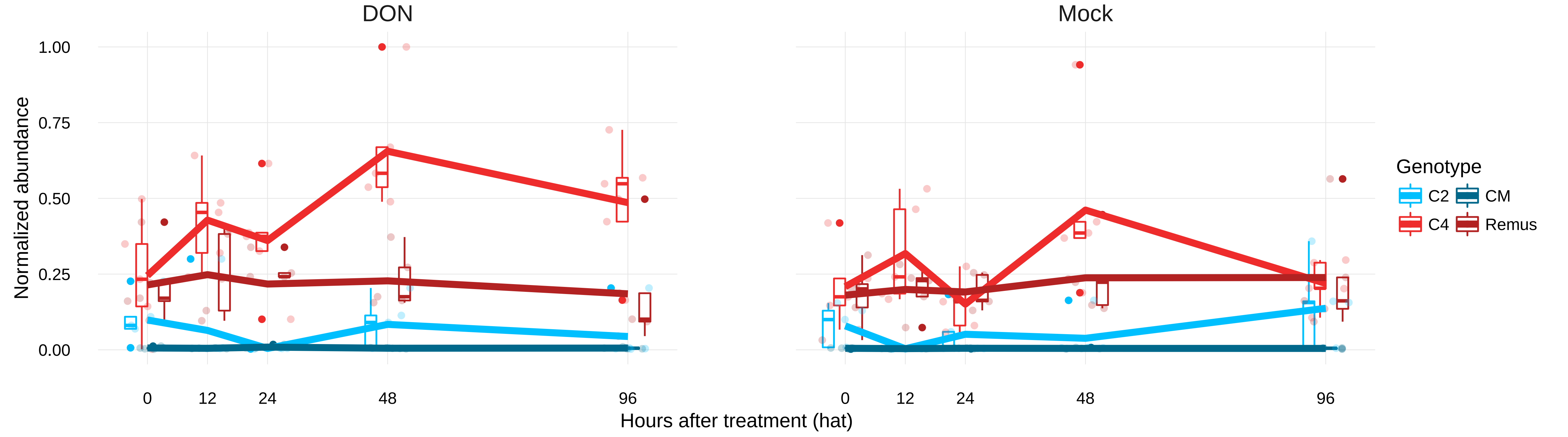

A.70

Annotated as Flavonoid (diGlcF)
(60 database hits)

MZ	625.1765
RT	13.46 min
Normalization	Directly via KPX samples
Cluster	Cluster 4
Cn total / Phe	28 /

C2, C4; different treatments

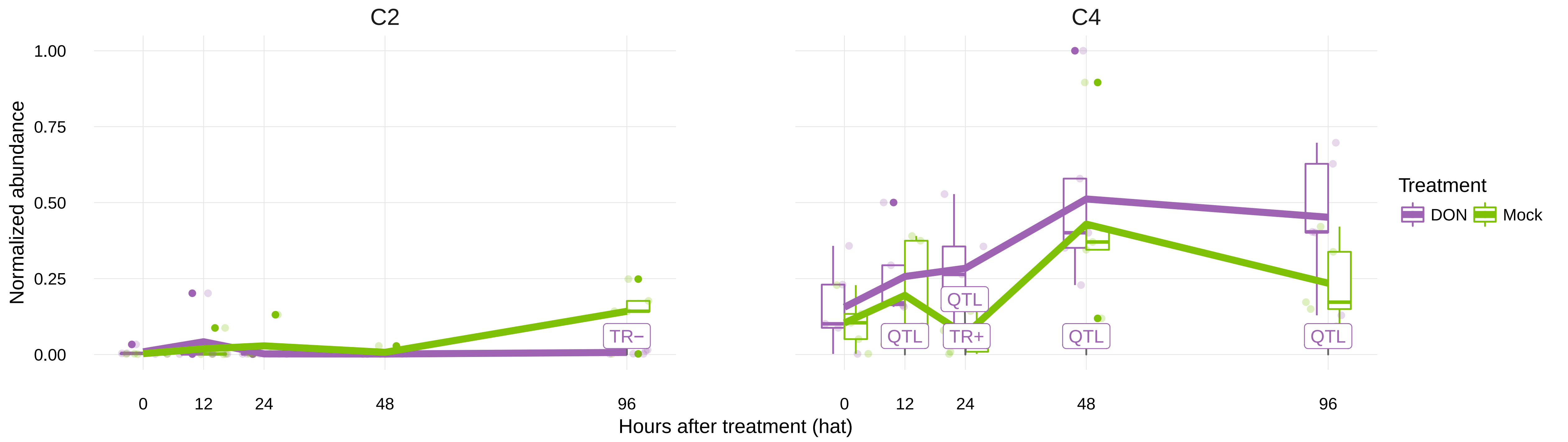

DON, Mock; different genotypes

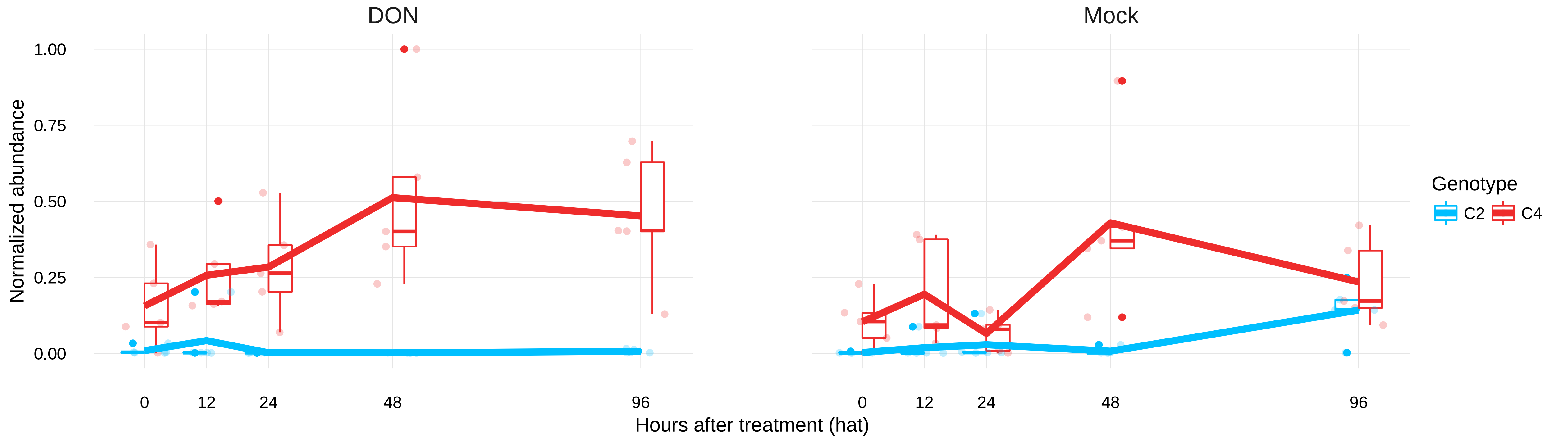

CM, Remus; different treatments

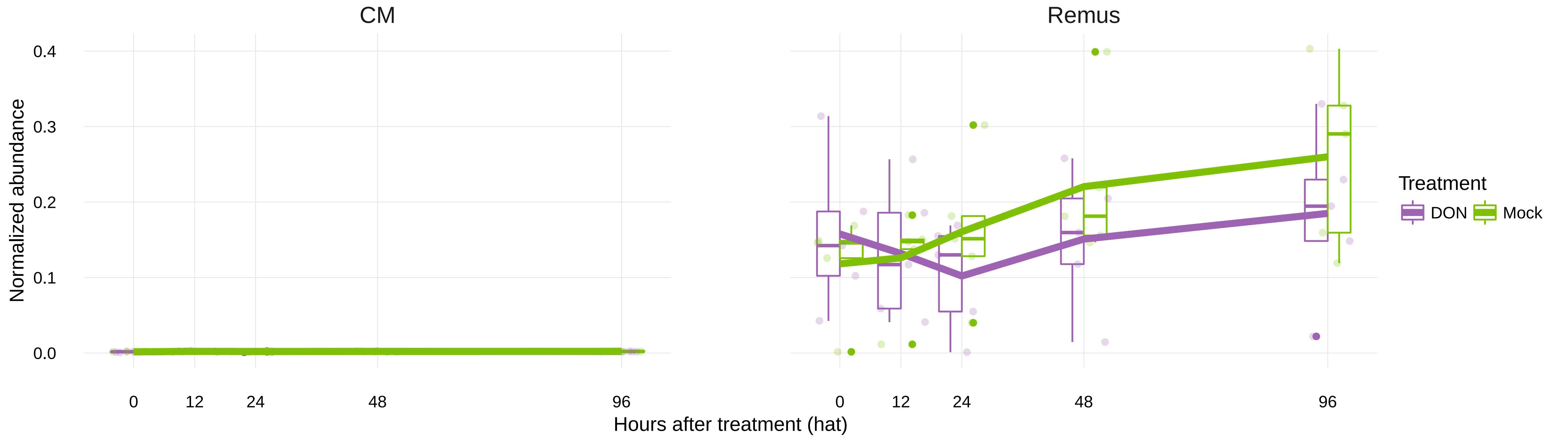

DON, Mock; all four genotypes

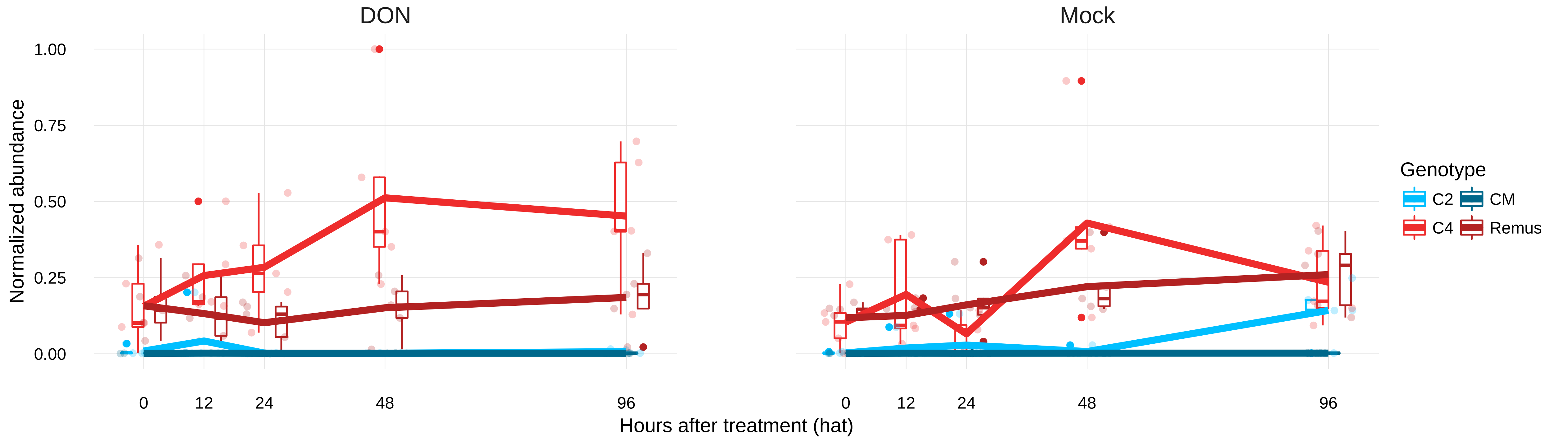

A.55

Annotated as Flavonoid (diGlcF)

(48 database hits)

MZ	581.1504
RT	12.58 min
Normalization	Directly via KPX samples
Cluster	Cluster 4
Cn total / Phe	26 / 9

C2, C4; different treatments

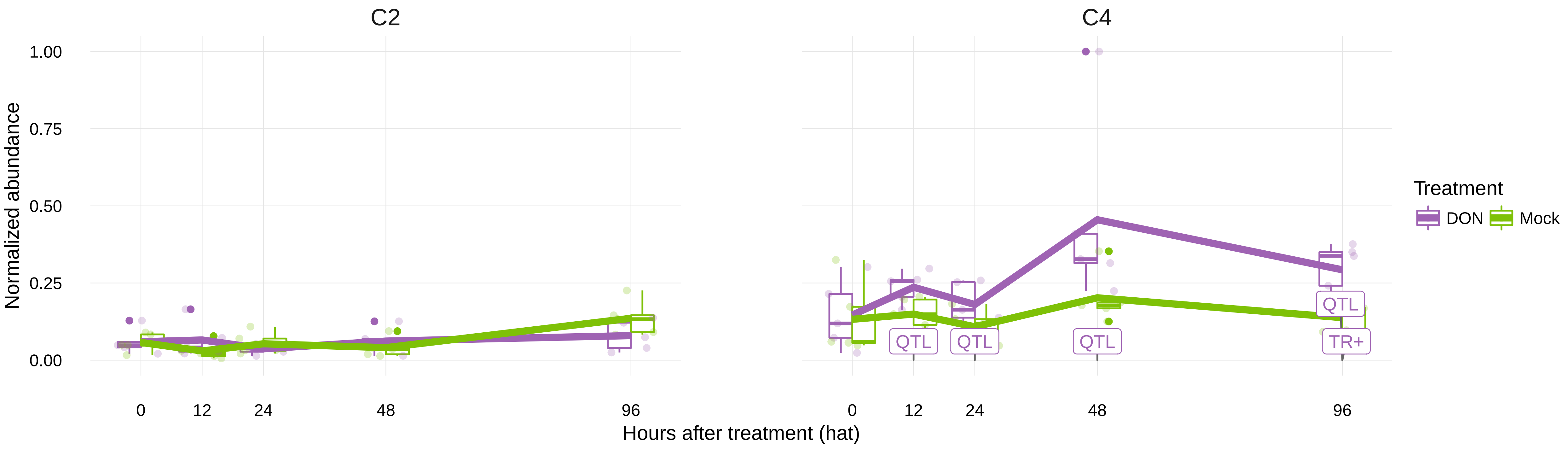

DON, Mock; different genotypes

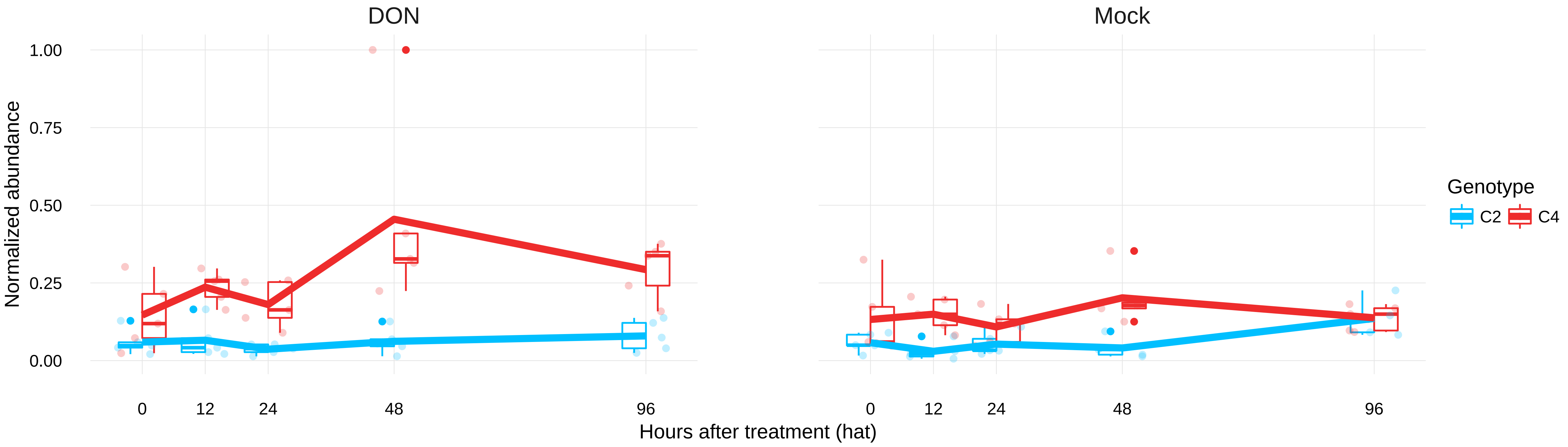

CM, Remus; different treatments

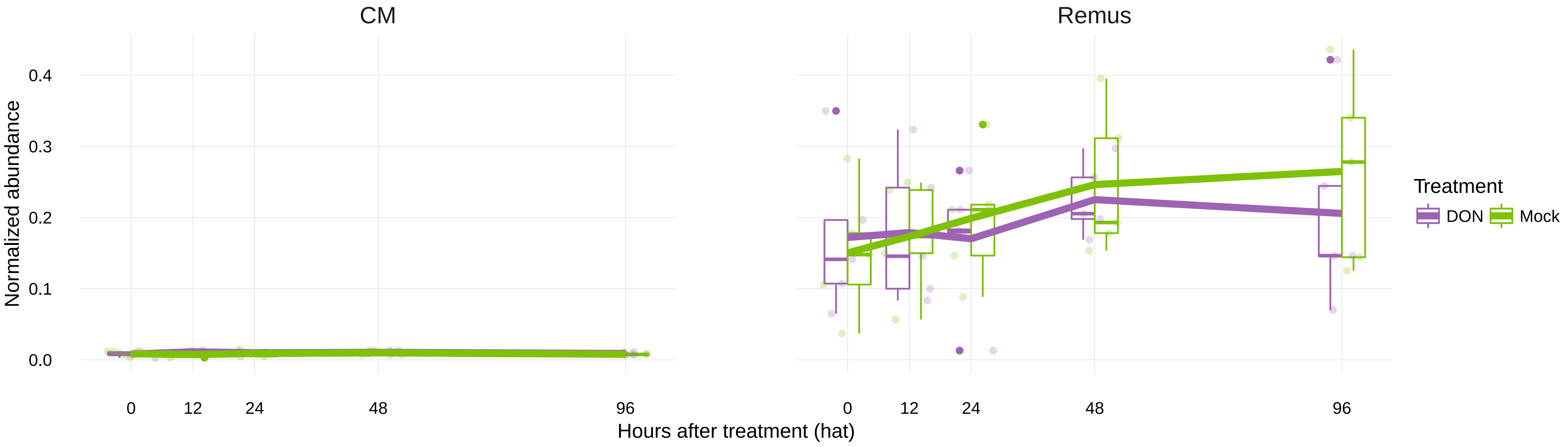

DON, Mock; all four genotypes

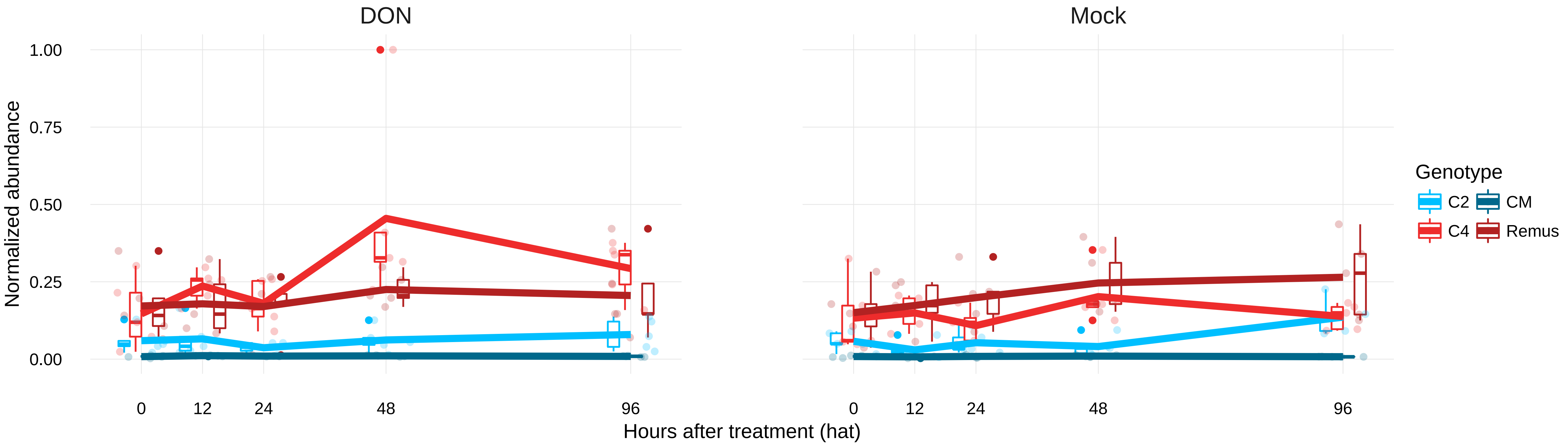

A.81

Annotated as Flavonoid (GlcF)

(52 database hits)

MZ	479.1188
RT	14.6 min
Normalization	Directly via KPX samples
Cluster	Cluster 4
Cn total / Phe	22 /

C2, C4; different treatments

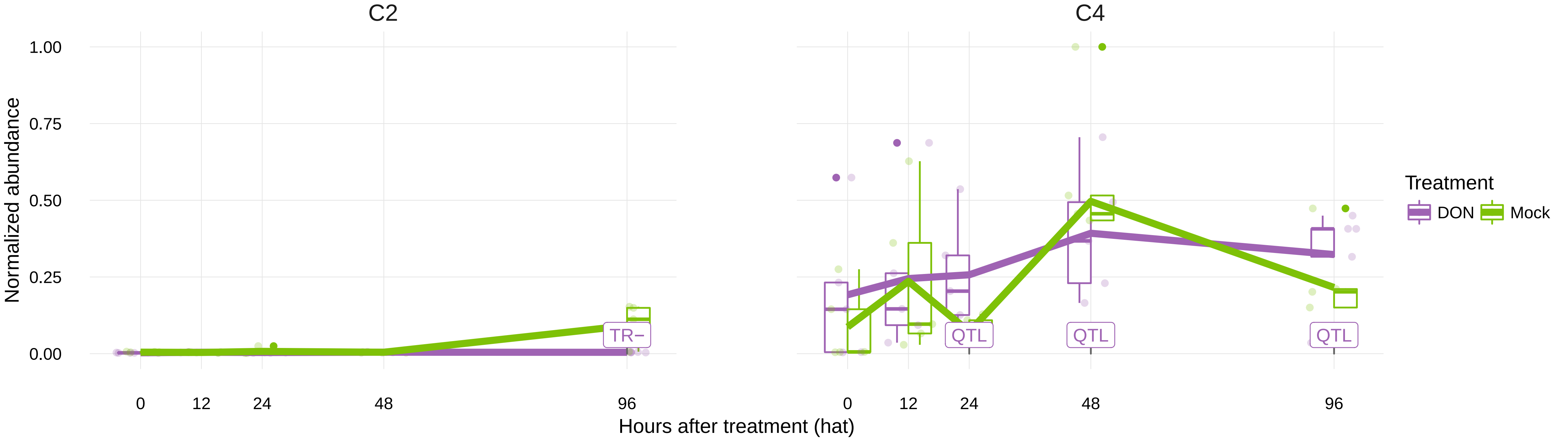

DON, Mock; different genotypes

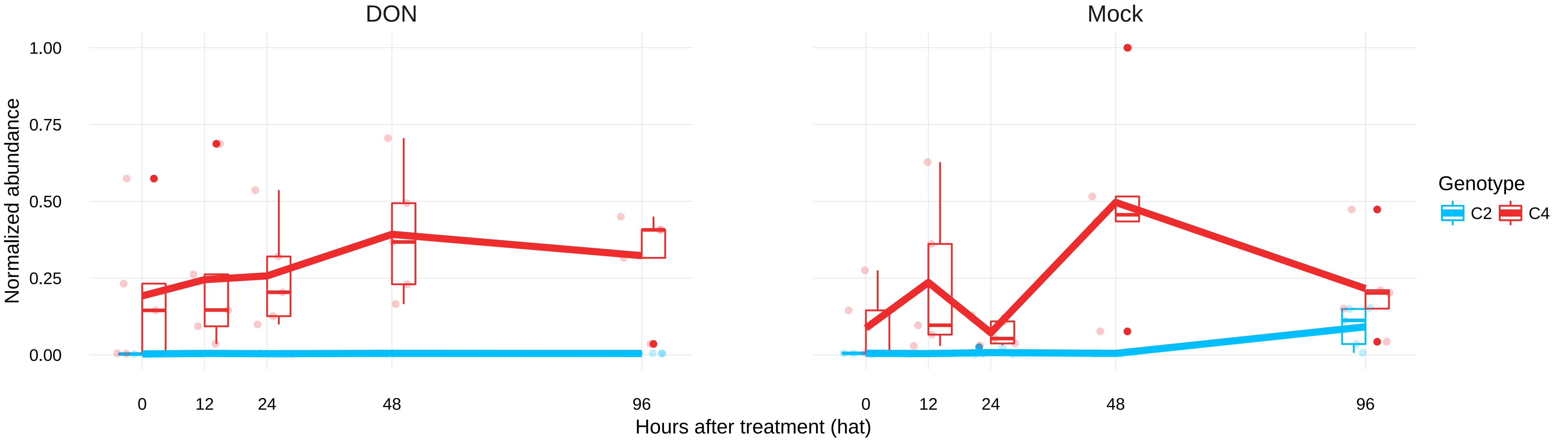

CM, Remus; different treatments

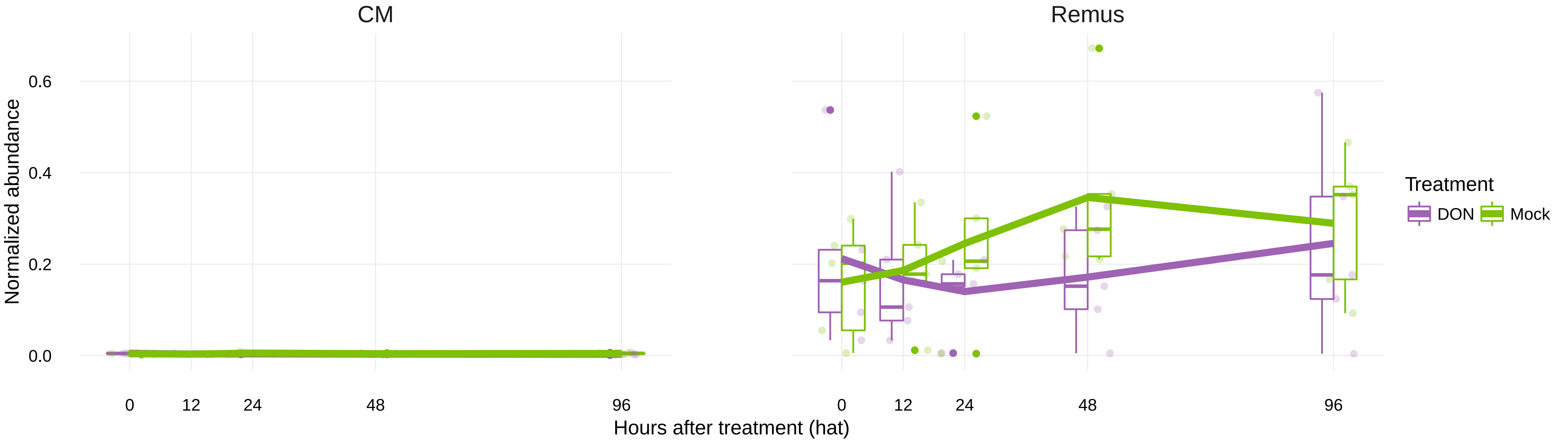

DON, Mock; all four genotypes

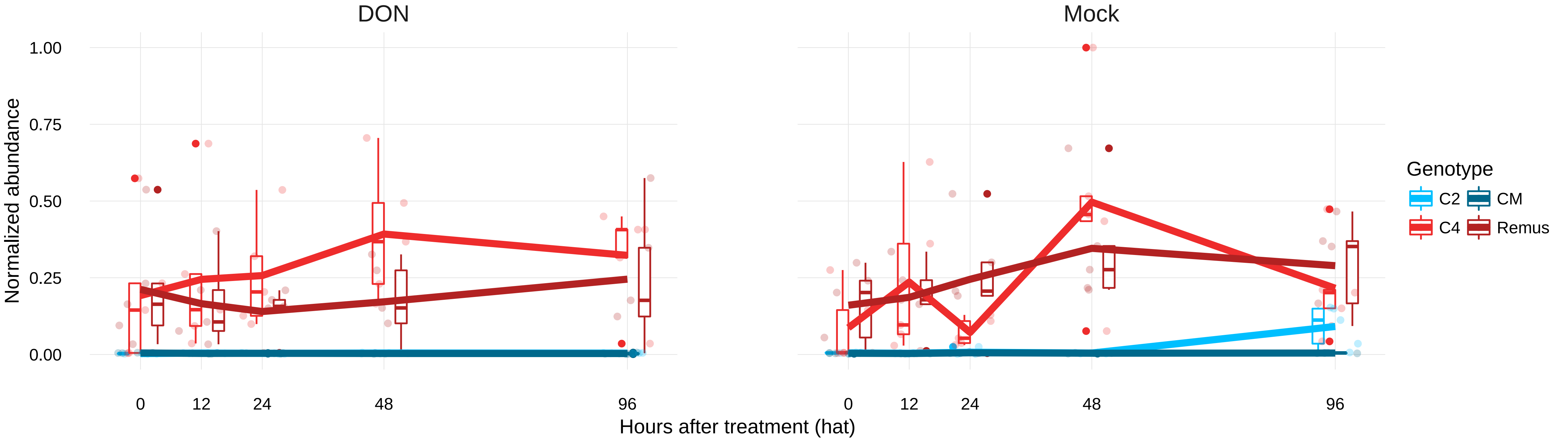

U.9

Unknown Phe−derived wheat−metabolite

MZ	584.1288
RT	13.44 min
Normalization	Directly via KPX samples
Cluster	Cluster 4
Cn total / Phe	8 / 8

C2, C4; different treatments

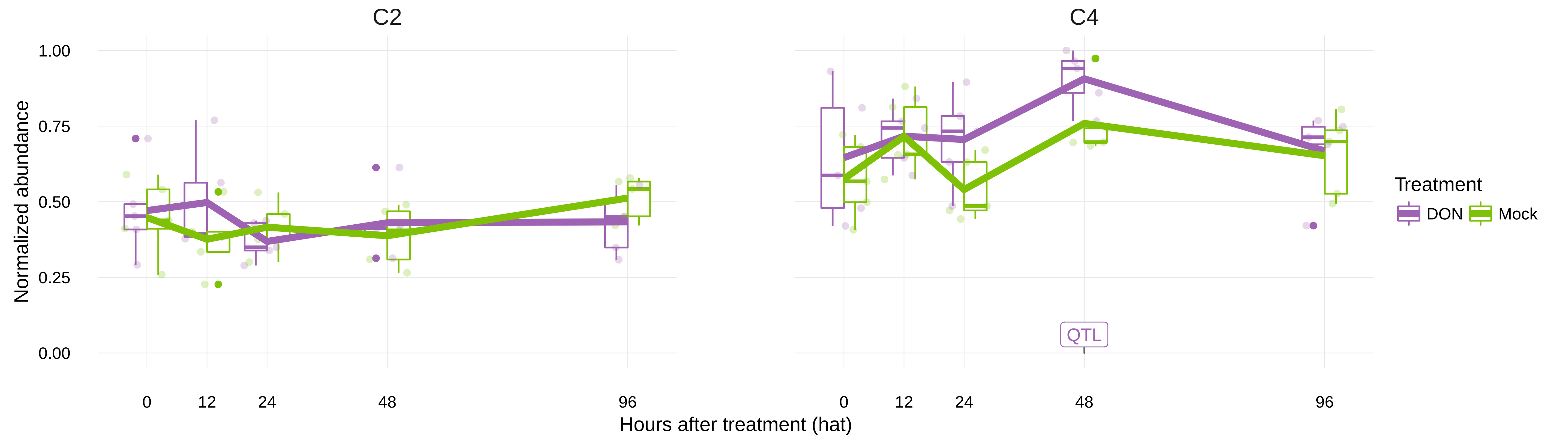

DON, Mock; different genotypes

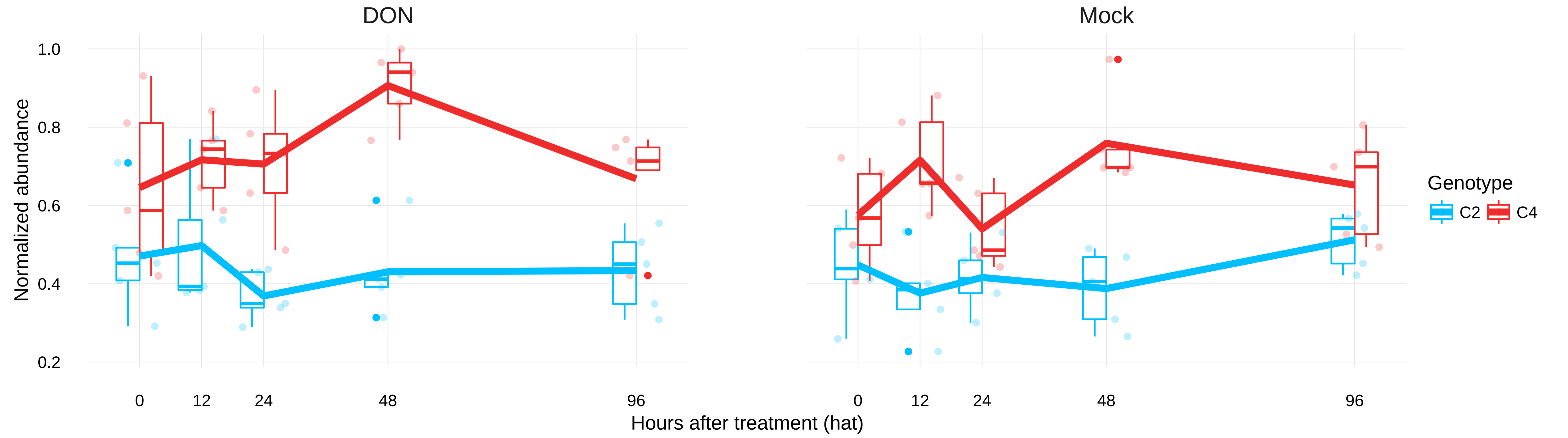

CM, Remus; different treatments

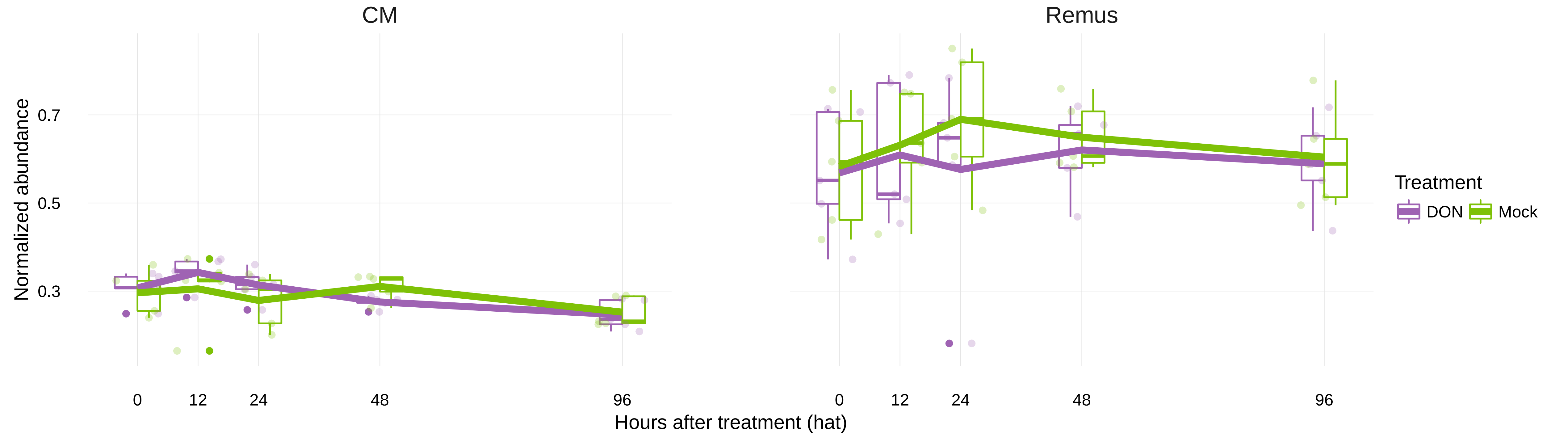

DON, Mock; all four genotypes

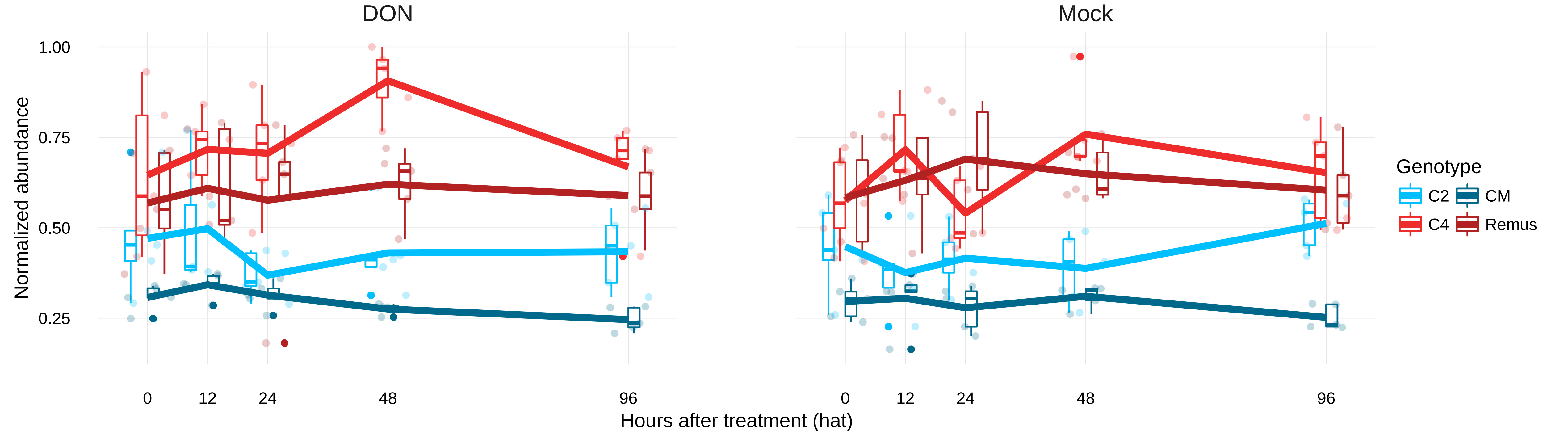

A.95

Annotated as Putative phenylacetic acid derivative
(2 database hits)

MZ	388.1605
RT	16.26 min
Normalization	Indirectly via surrogate in the KPX samples
Cluster	Cluster 4
Cn total / Phe	17 / 8

C2, C4; different treatments

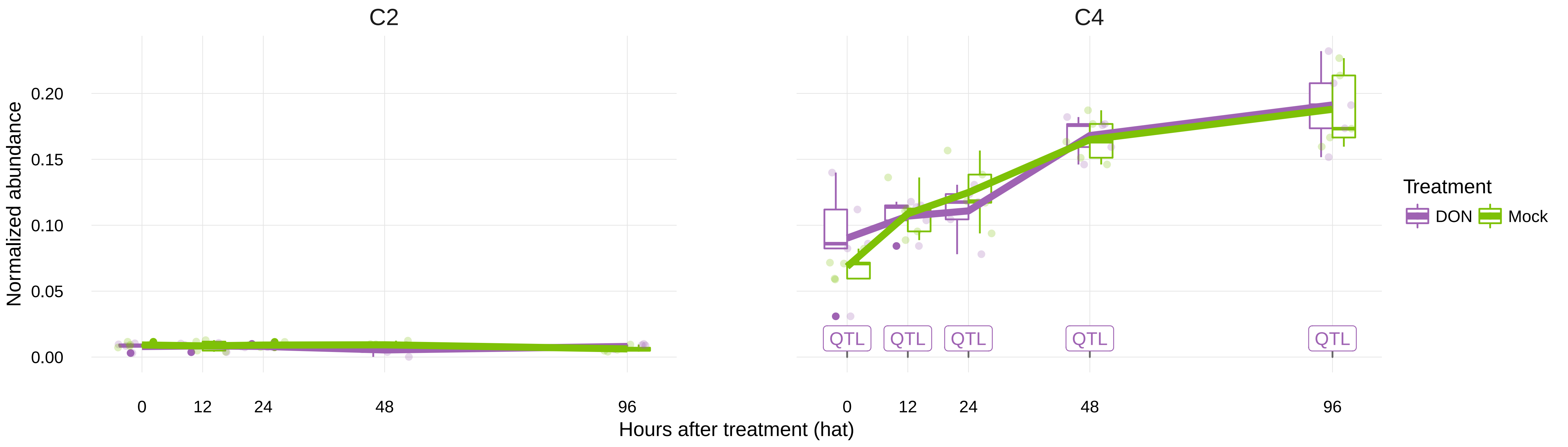

DON, Mock; different genotypes

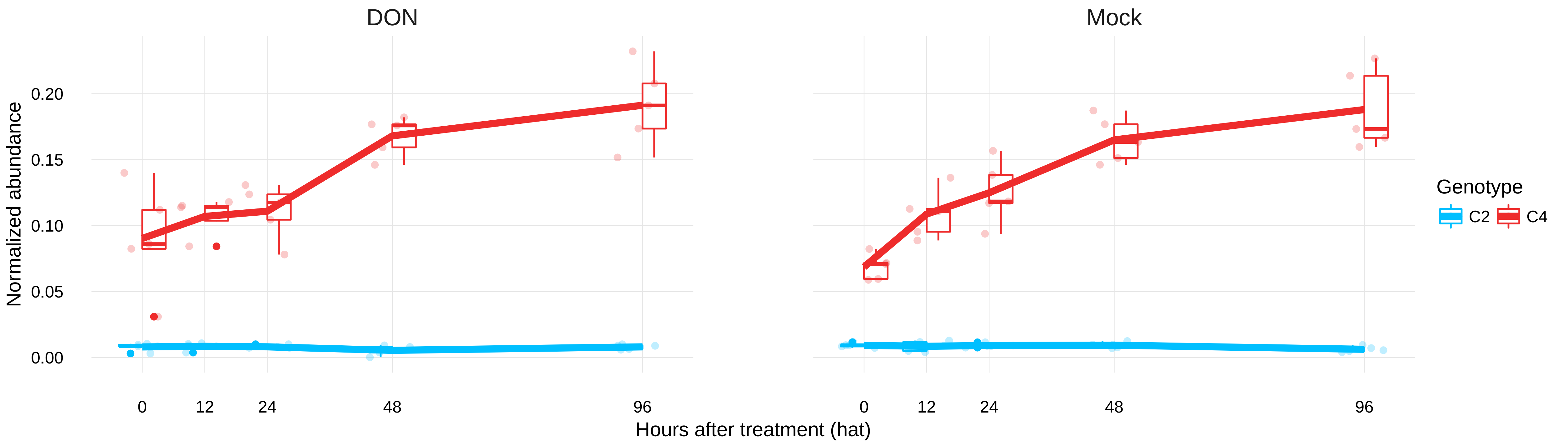

CM, Remus; different treatments

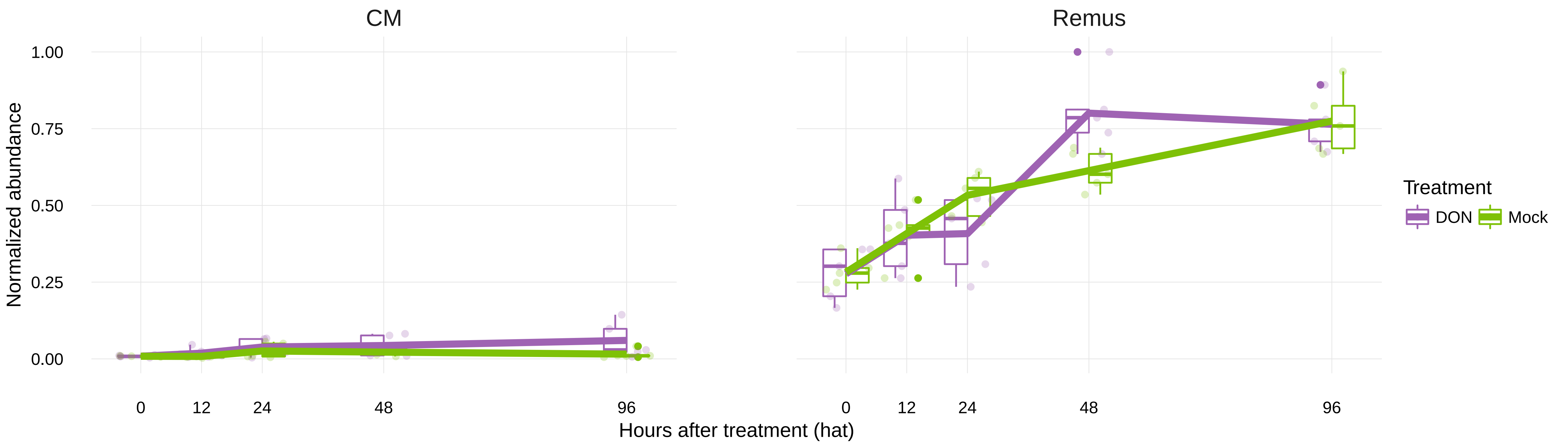

DON, Mock; all four genotypes

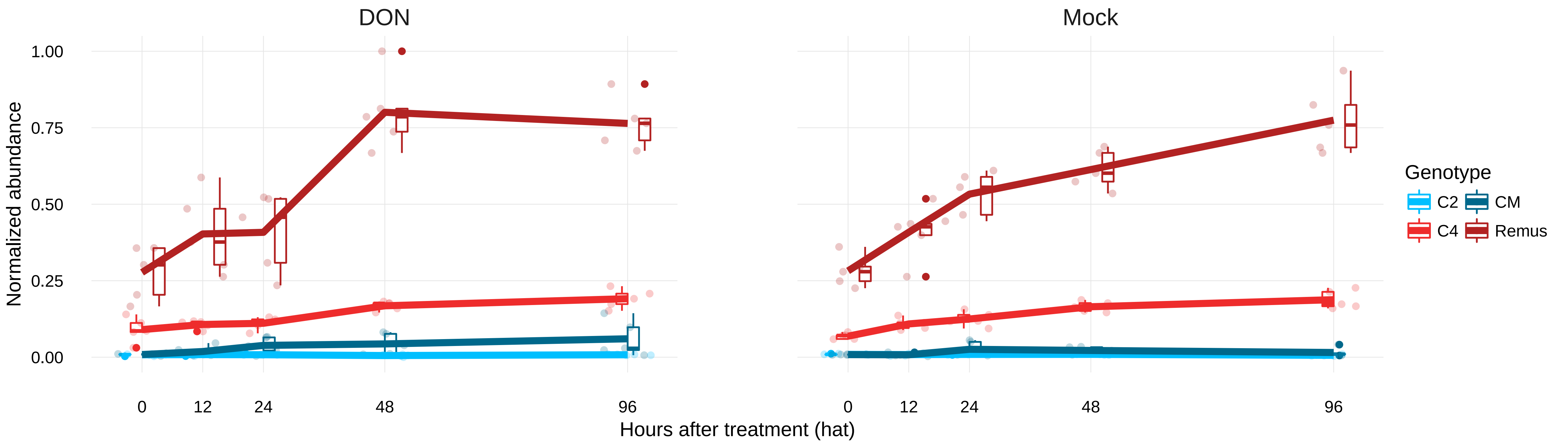

A.56

Annotated as Putative benzoic acid derivative
(14 database hits)

MZ	355.1025
RT	12.75 min
Normalization	Indirectly via surrogate in the KPX samples
Cluster	Cluster 4
Cn total / Phe	16 / 7

C2, C4; different treatments

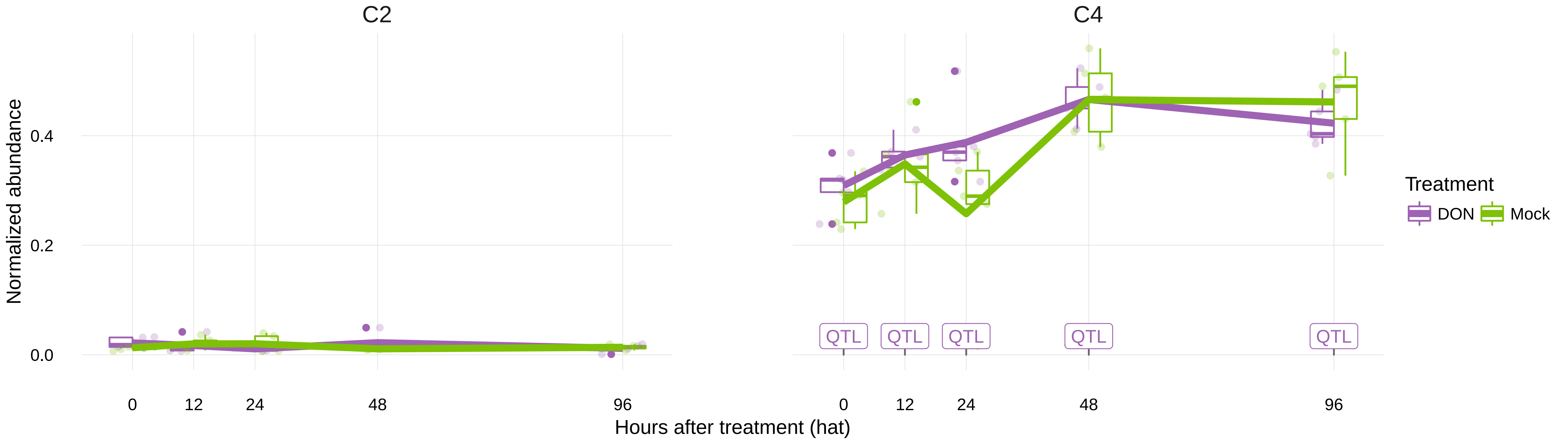

DON, Mock; different genotypes

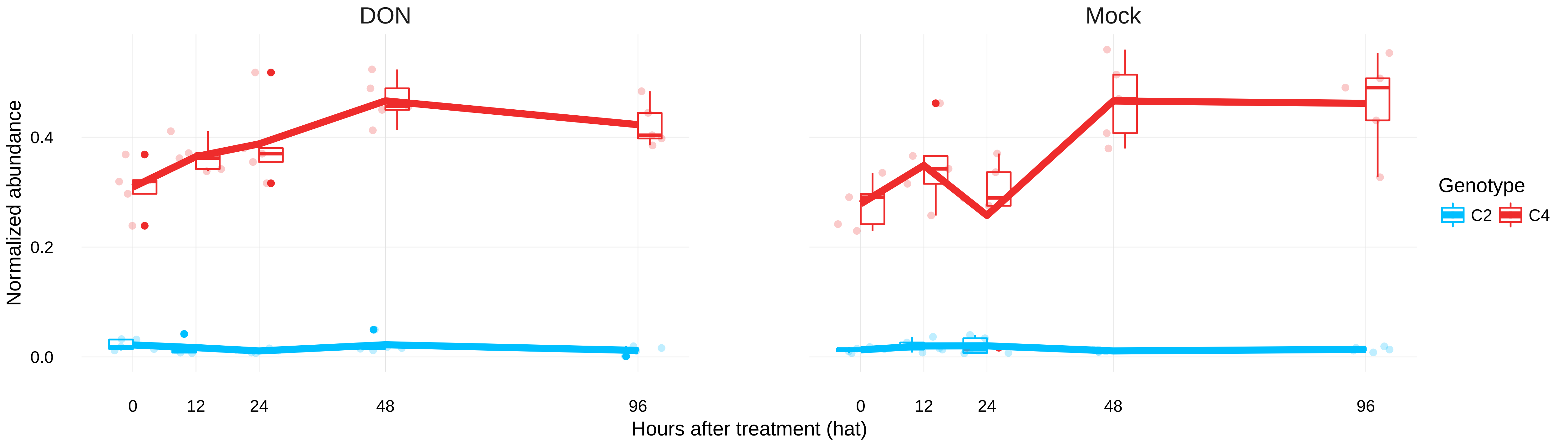

CM, Remus; different treatments

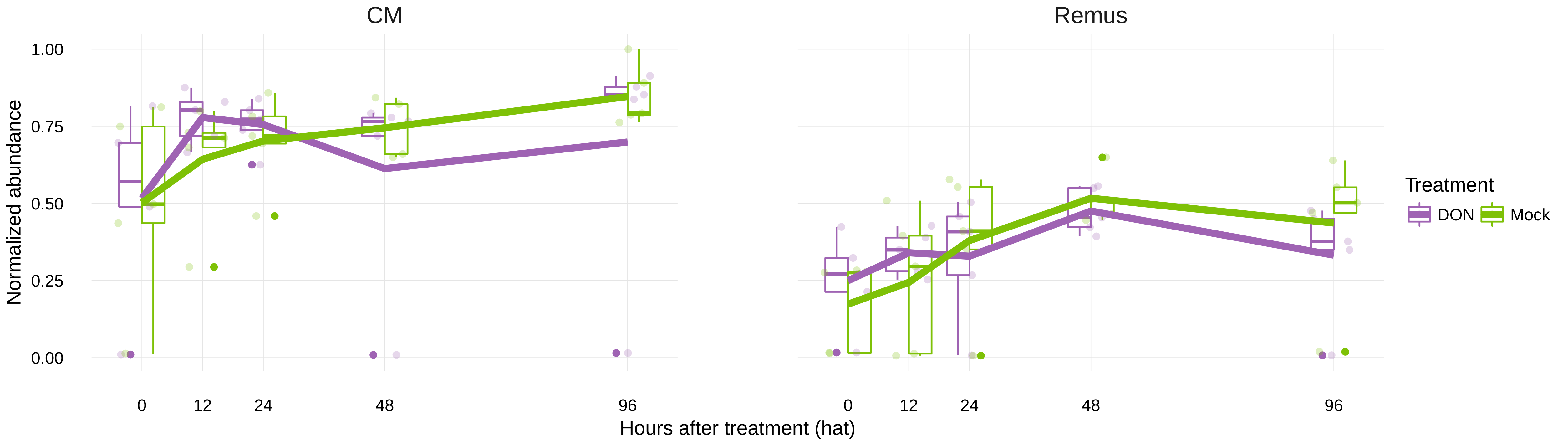

DON, Mock; all four genotypes

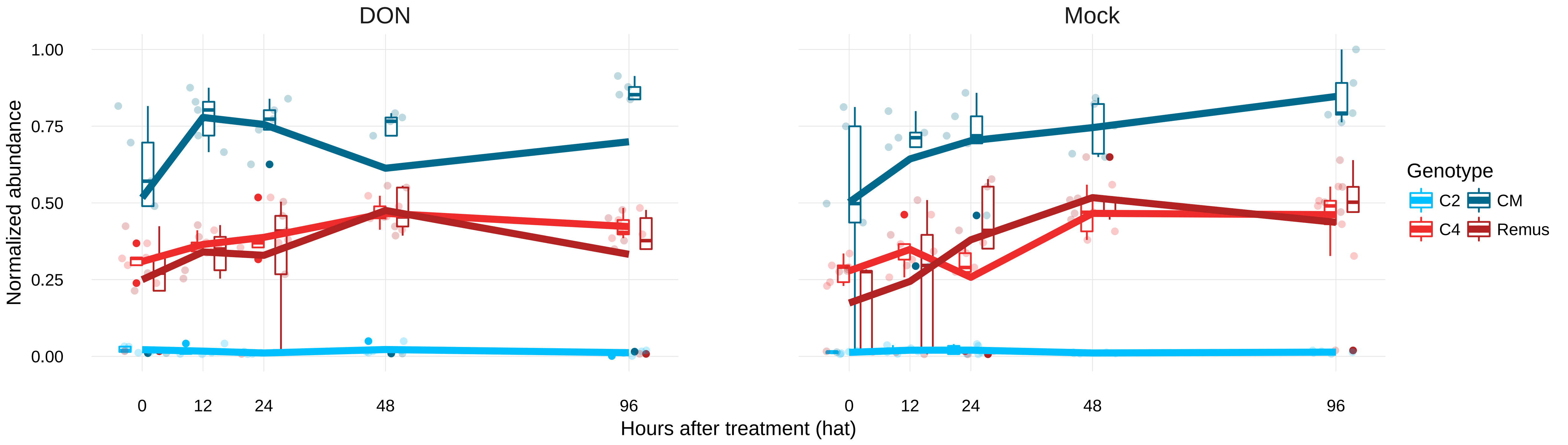

A.82

Annotated as others (Iridoid)
(1 database hit)

MZ	584.234
RT	14.65 min
Normalization	Indirectly via surrogate in the KPX samples
Cluster	Cluster 4
Cn total / Phe	27 / 9

C2, C4; different treatments

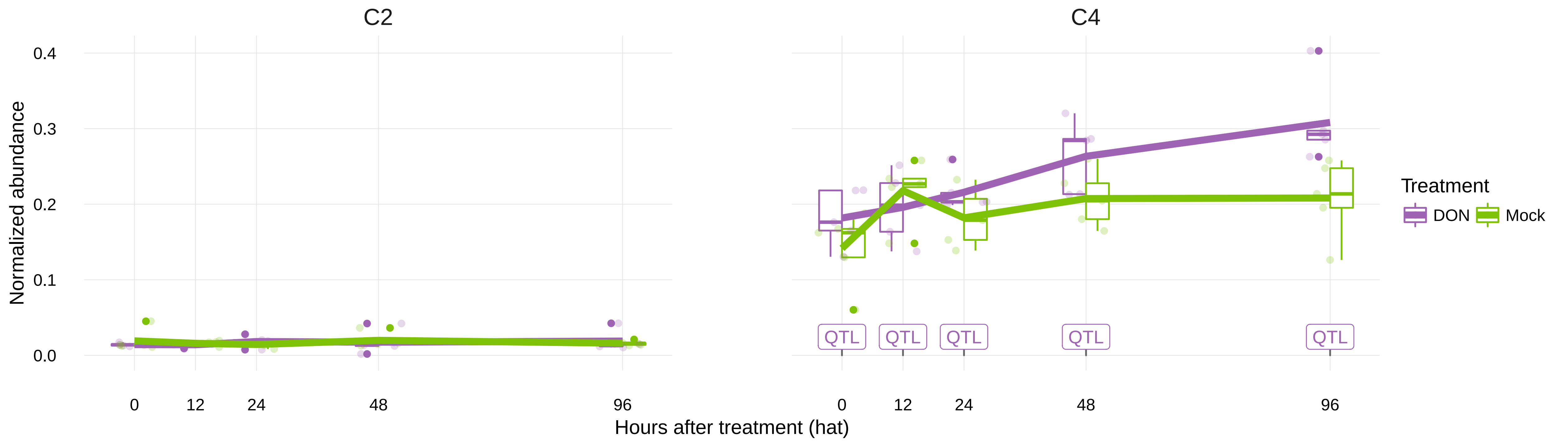

DON, Mock; different genotypes

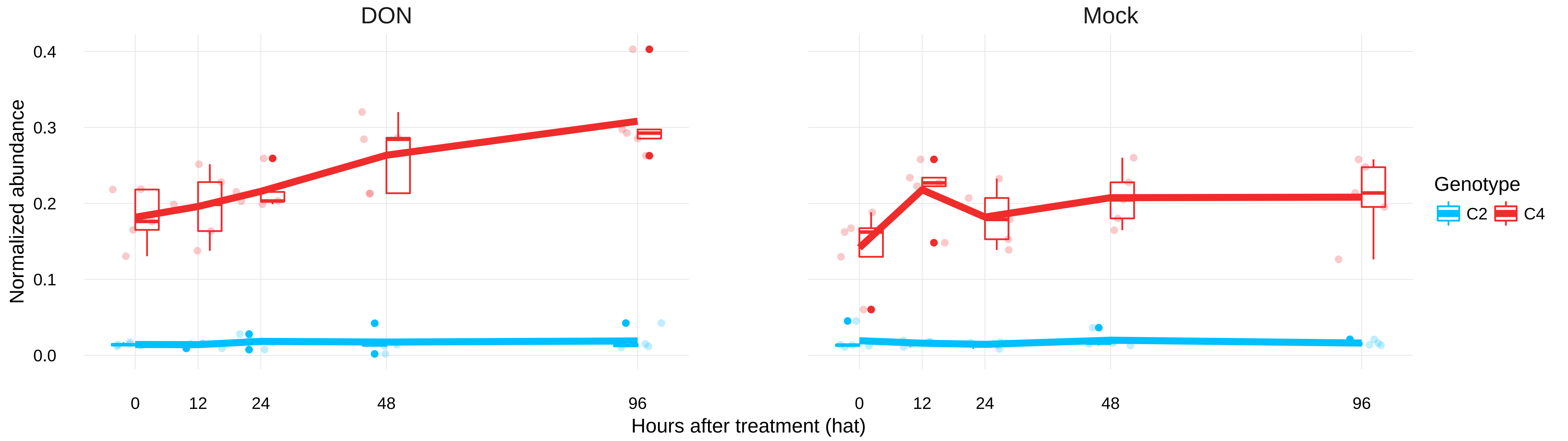

CM, Remus; different treatments

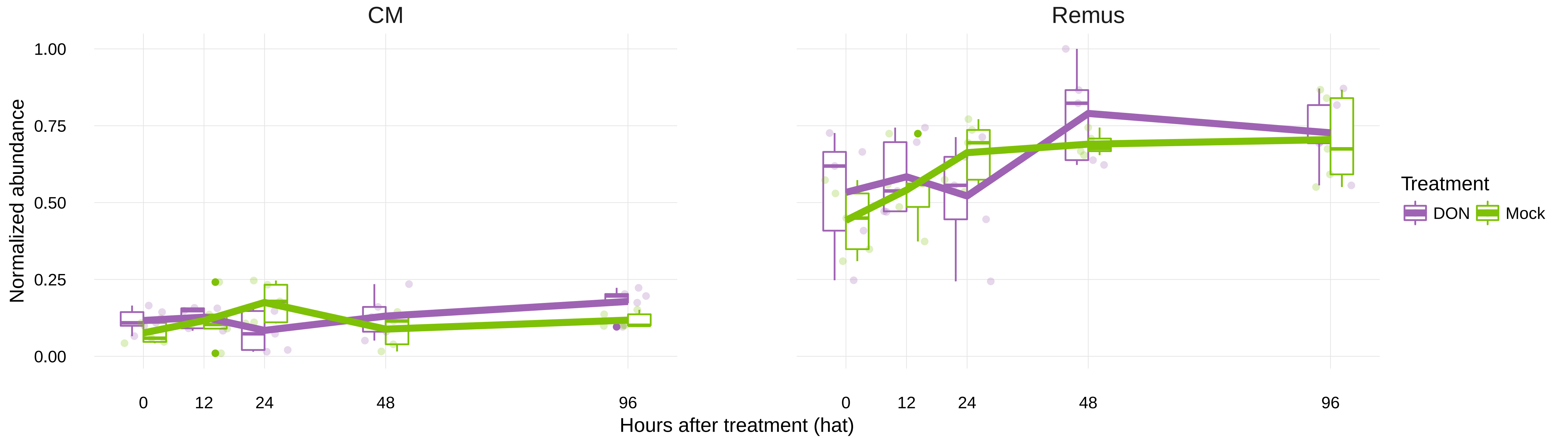

DON, Mock; all four genotypes

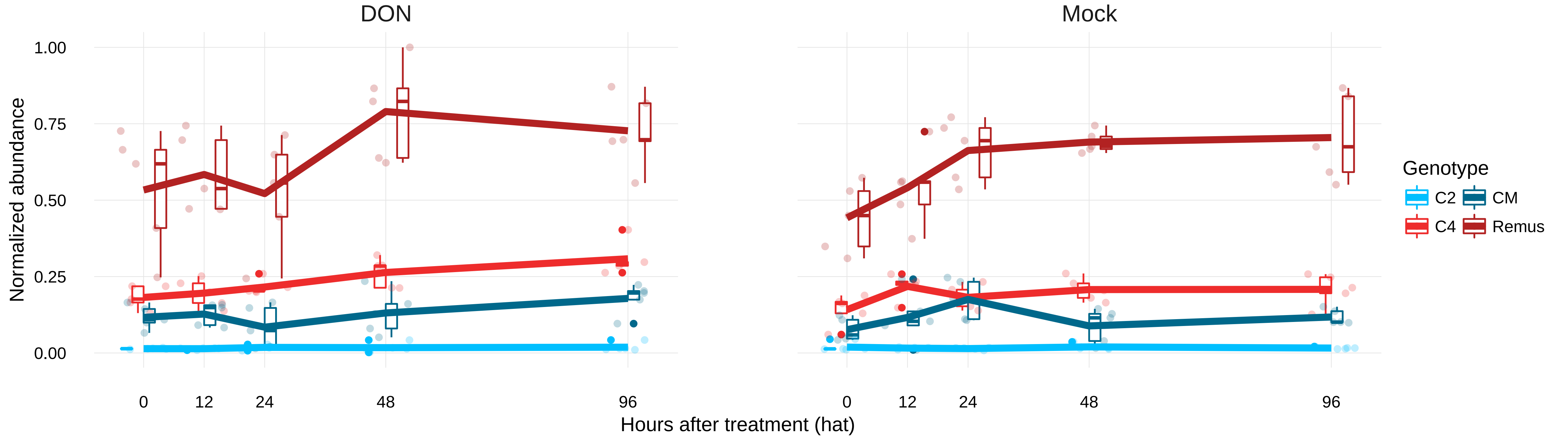

A.138

Annotated as HCA derivative
(1 database hit)

MZ	495.1285
RT	24.08 min
Normalization	Directly via KPX samples
Cluster	Cluster 4
Cn total / Phe	26 /

C2, C4; different treatments

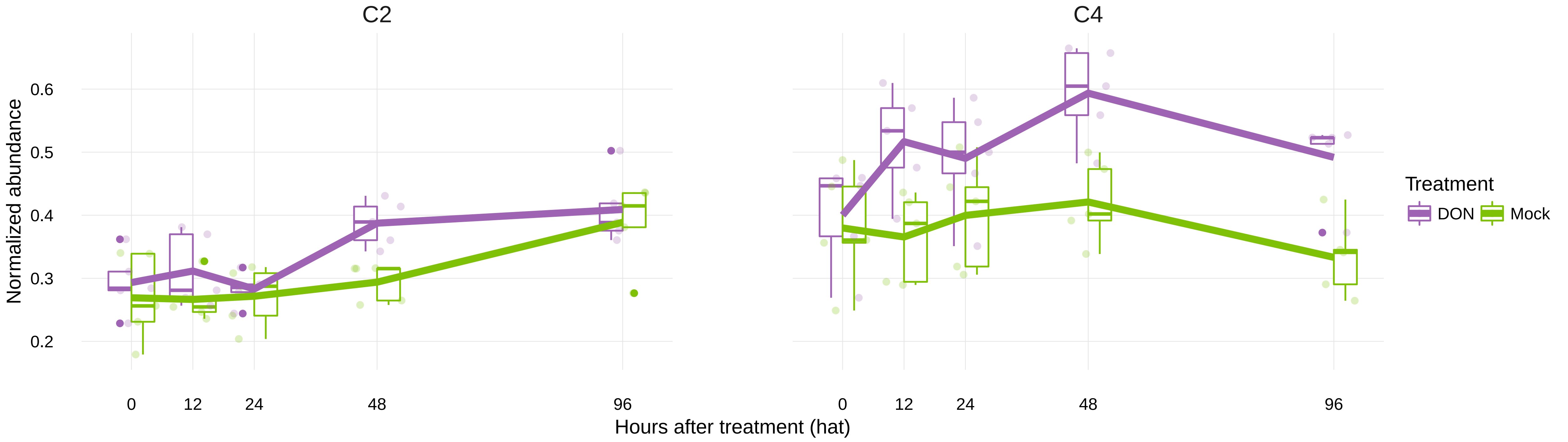

DON, Mock; different genotypes

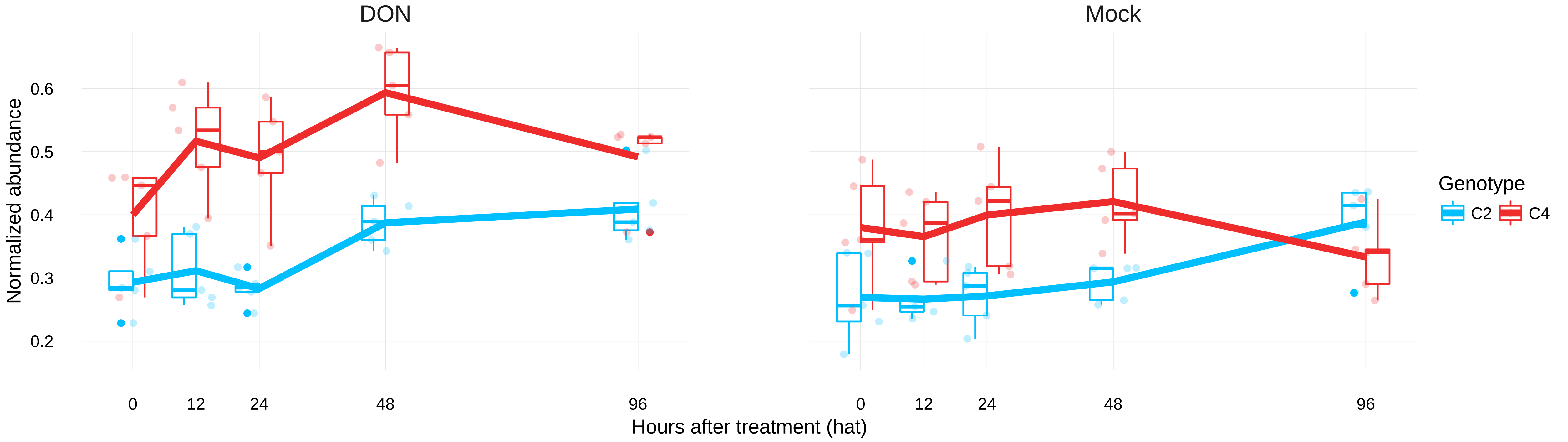

CM, Remus; different treatments

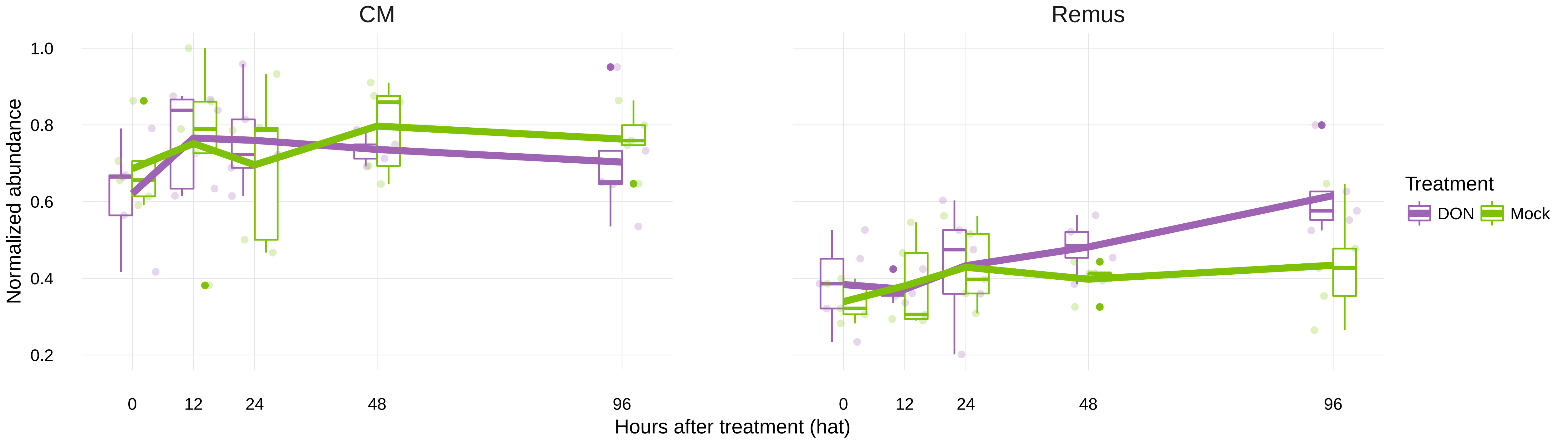

DON, Mock; all four genotypes

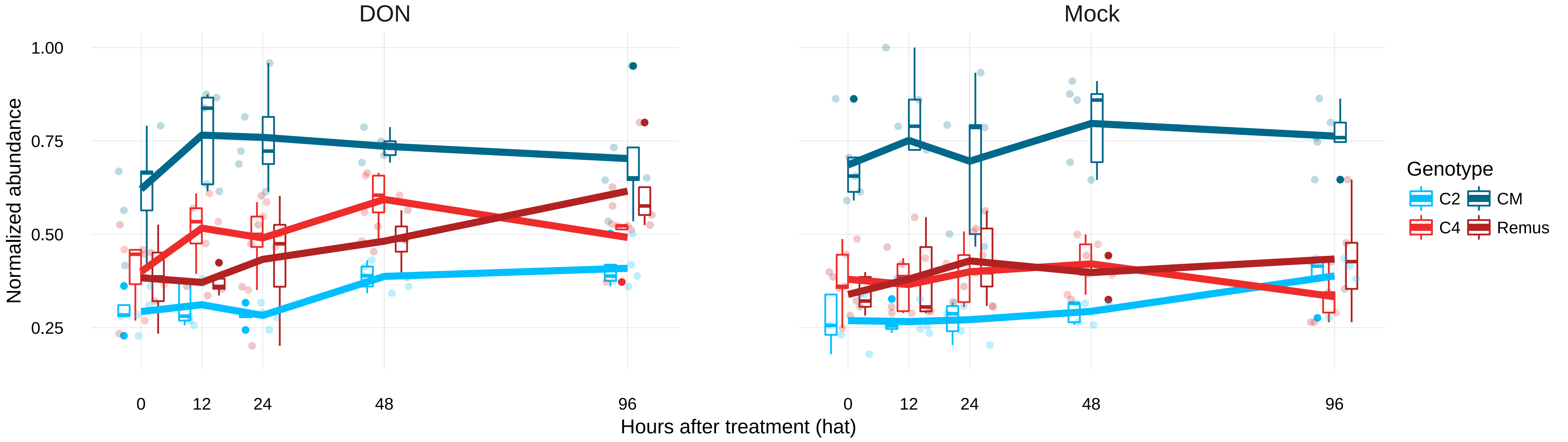

A.42

Annotated as HCA derivative
(1 database hit)

MZ	415.1239
RT	10.87 min
Normalization	Directly via KPX samples
Cluster	Cluster 4
Cn total / Phe	18 / 9

C2, C4; different treatments

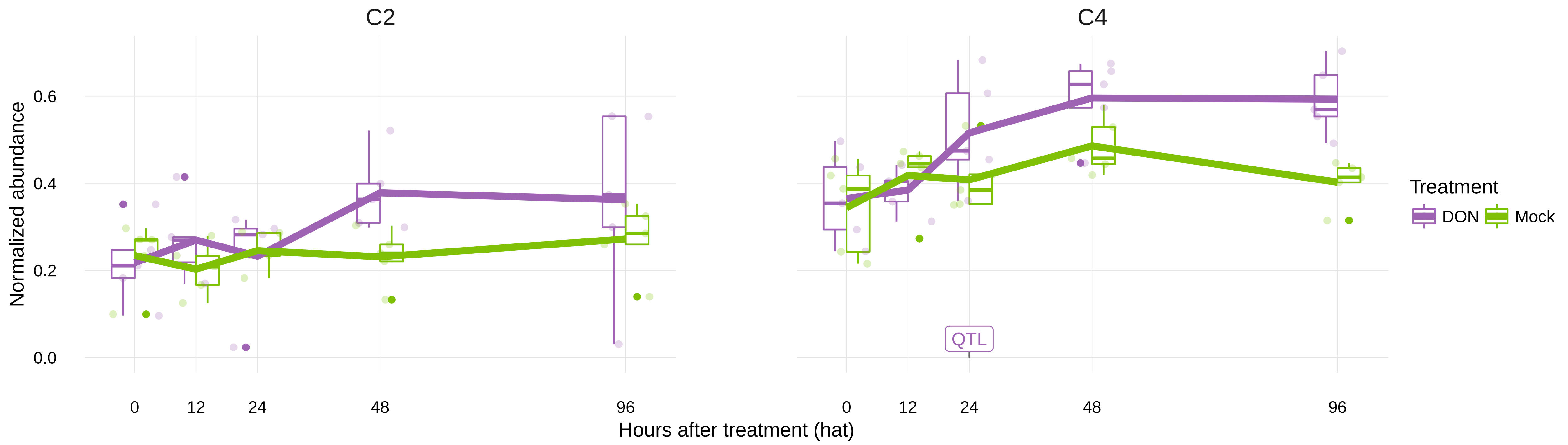

DON, Mock; different genotypes

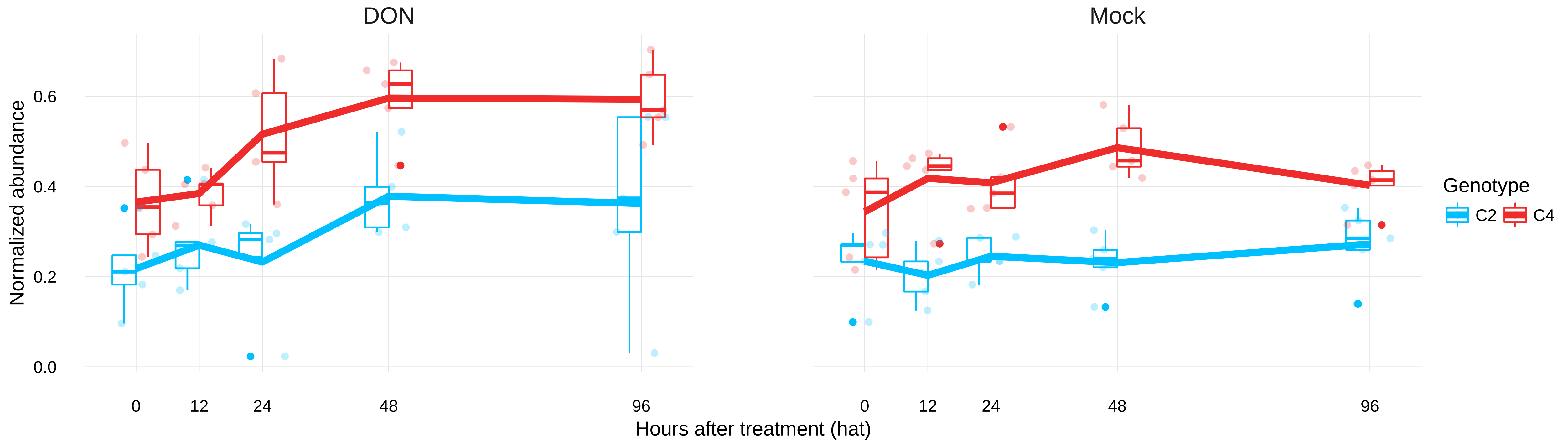

CM, Remus; different treatments

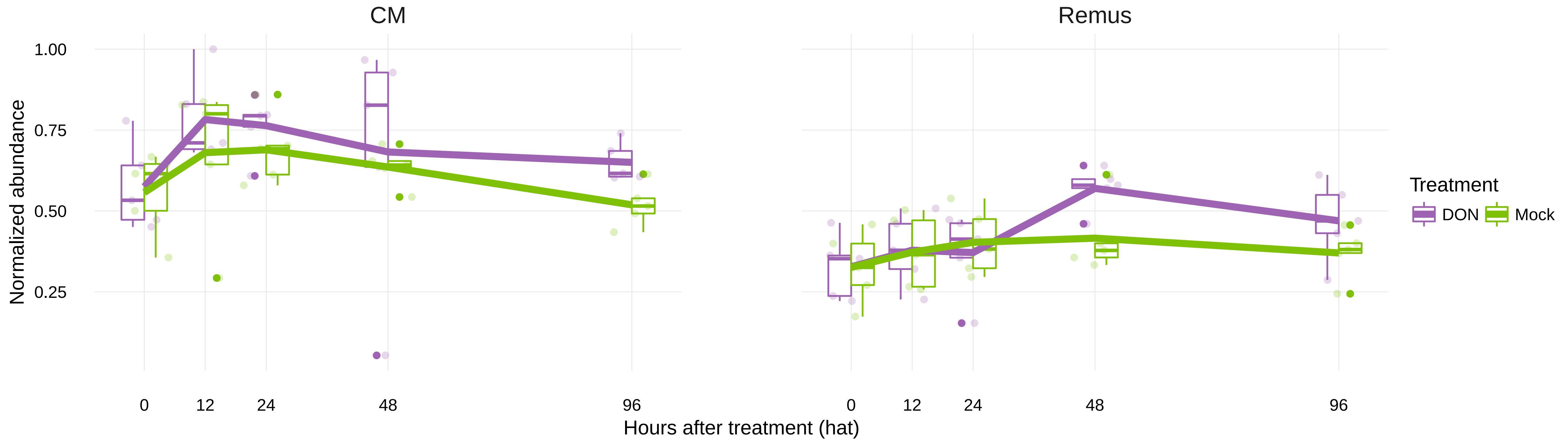

DON, Mock; all four genotypes

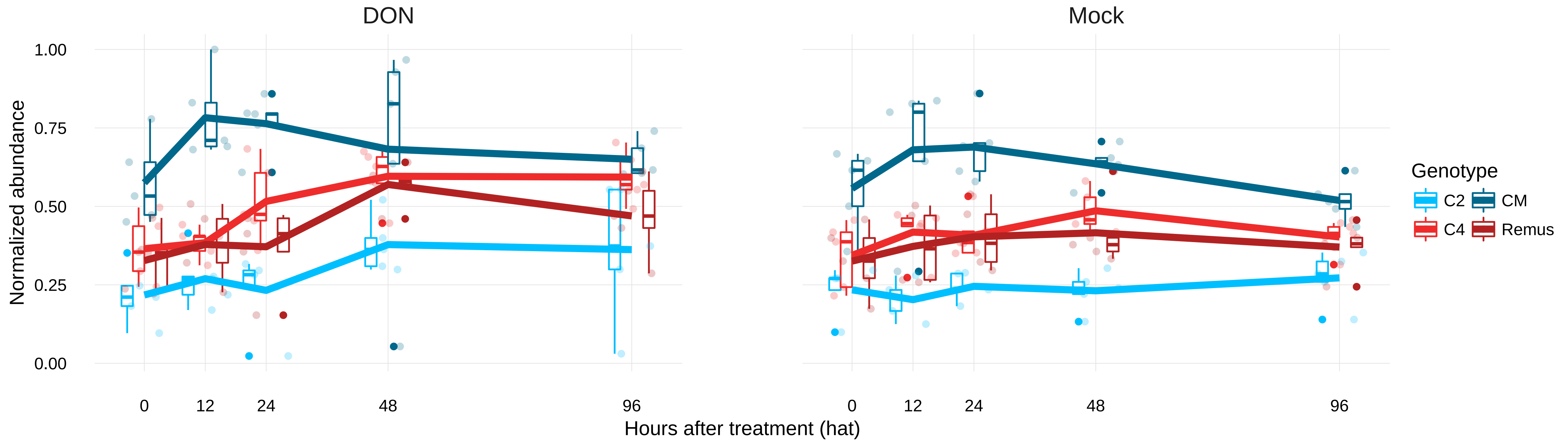

A.117

Annotated as Flavonoid (GlcF)

(52 database hits)

MZ	493.1341
RT	18.65 min
Normalization	Directly via KPX samples
Cluster	Cluster 4
Cn total / Phe	23 /

C2, C4; different treatments

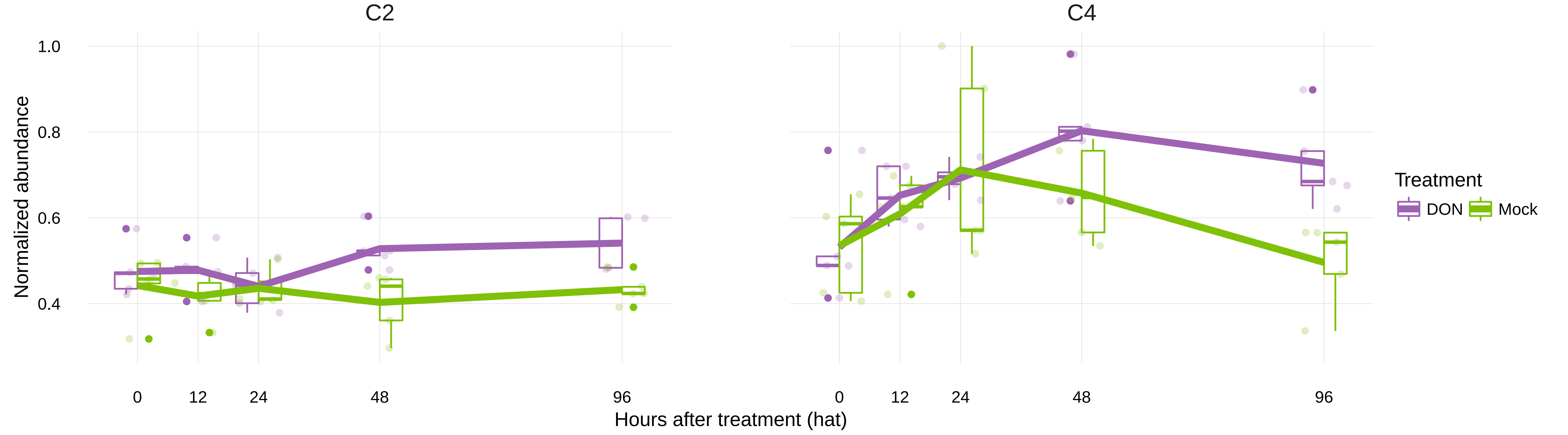

DON, Mock; different genotypes

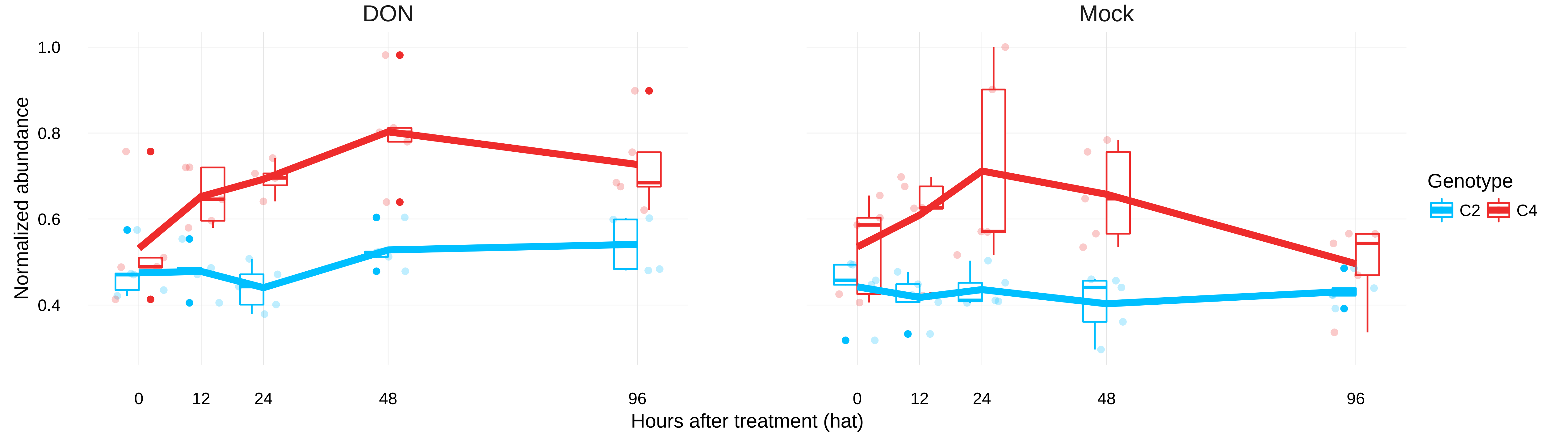

CM, Remus; different treatments

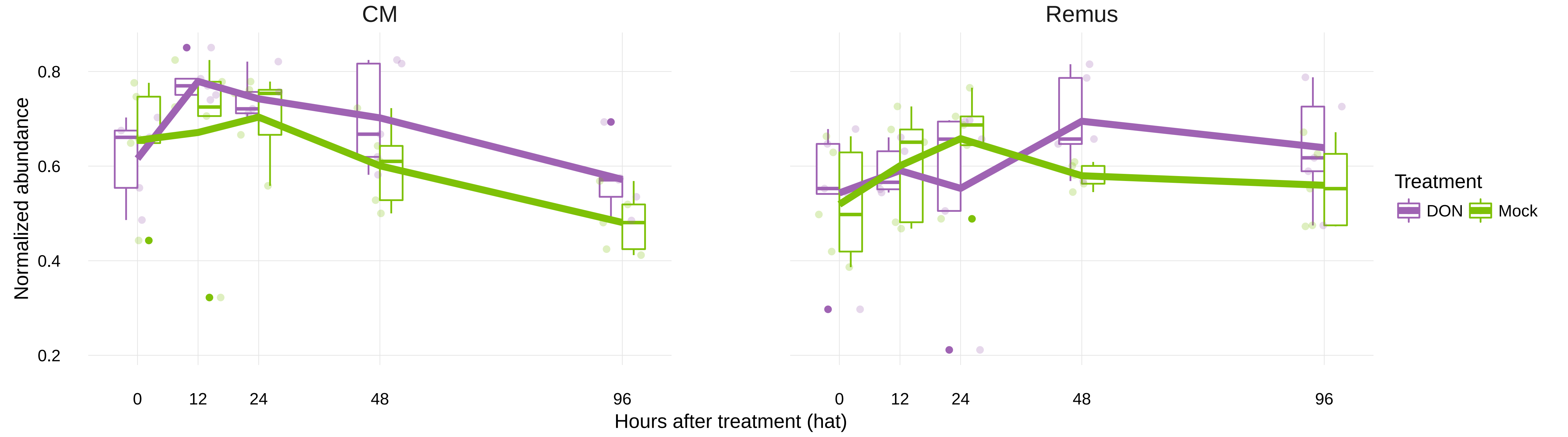

DON, Mock; all four genotypes

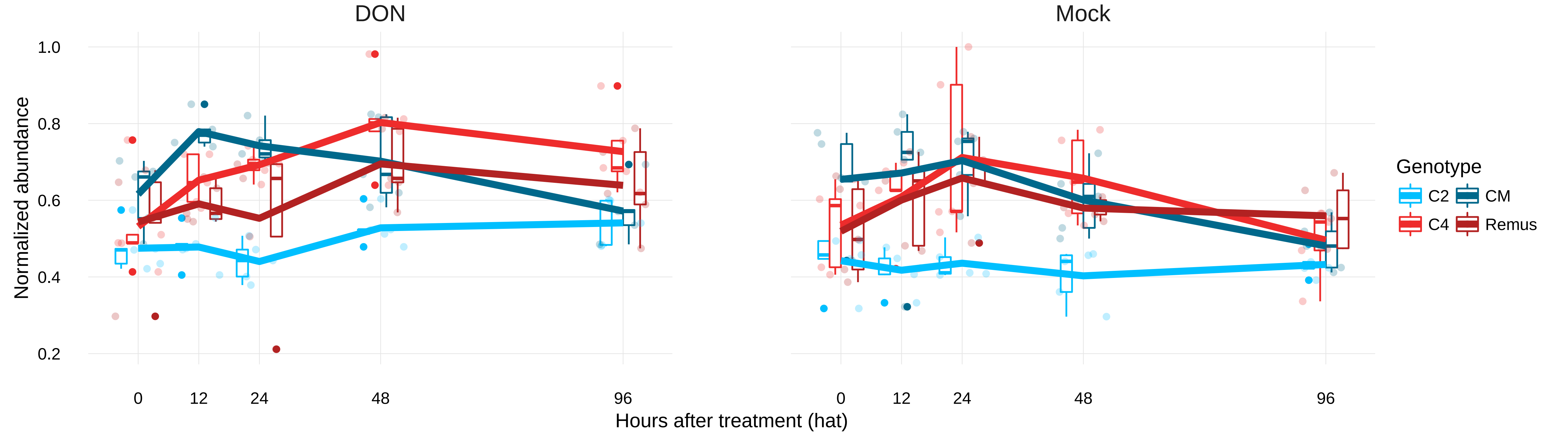

A.110

Annotated as Flavonoid (diGlcF)
(60 database hits)

MZ	647.158
RT	17.81 min
Normalization	Directly via KPX samples
Cluster	Cluster 4
Cn total / Phe	28 /

C2, C4; different treatments

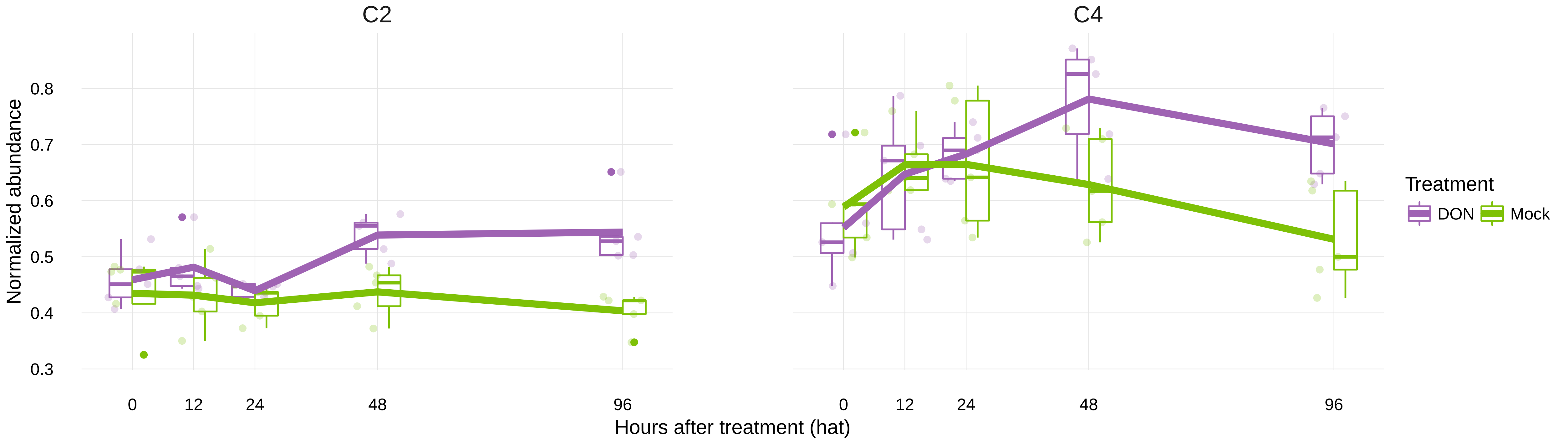

DON, Mock; different genotypes

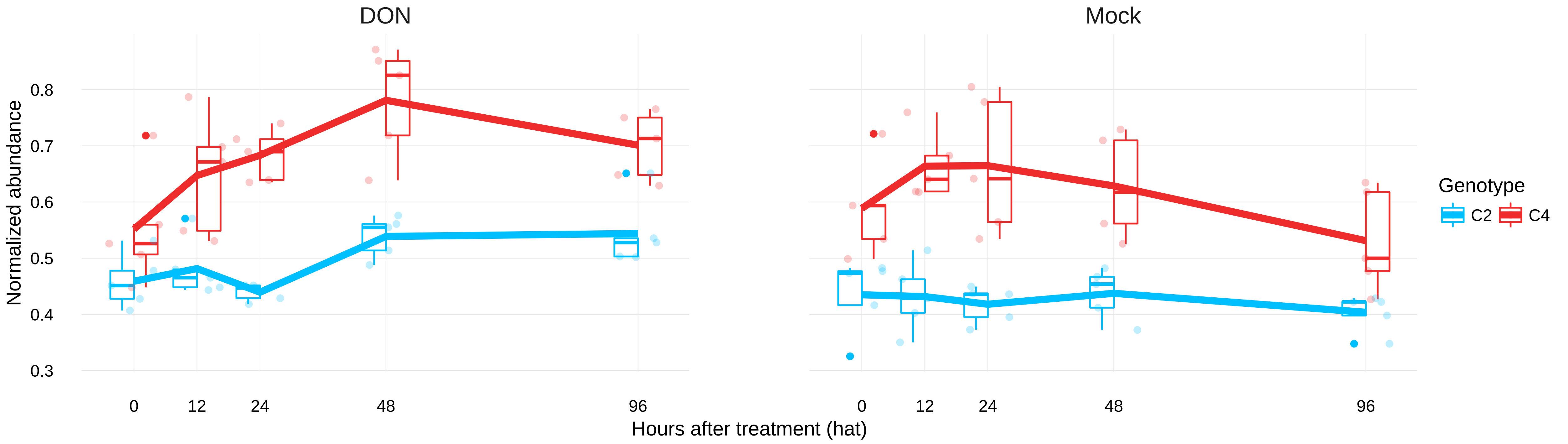

CM, Remus; different treatments

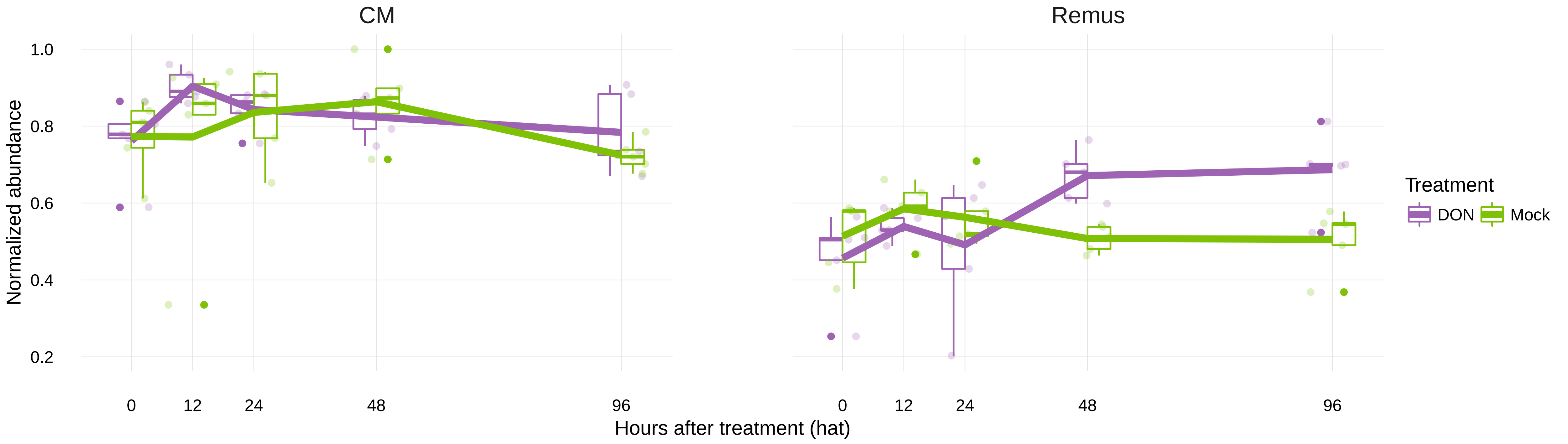

DON, Mock; all four genotypes

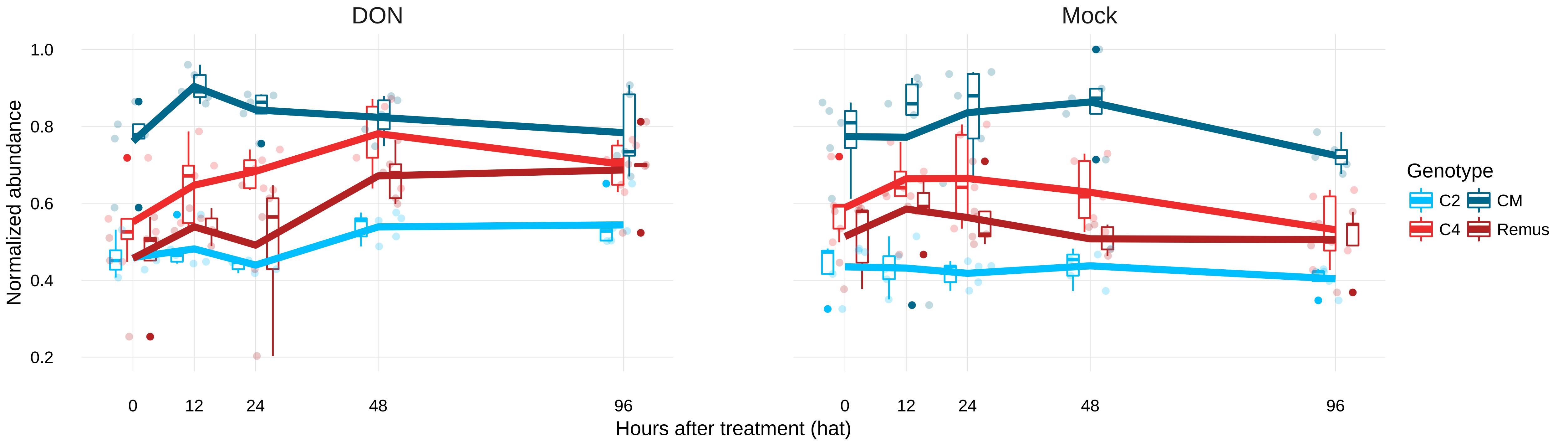

A.85

Annotated as Flavonoid (diGlcF)

(58 database hits)

MZ	579.171
RT	14.99 min
Normalization	Directly via KPX samples
Cluster	Cluster 4
Cn total / Phe	27 /

C2, C4; different treatments

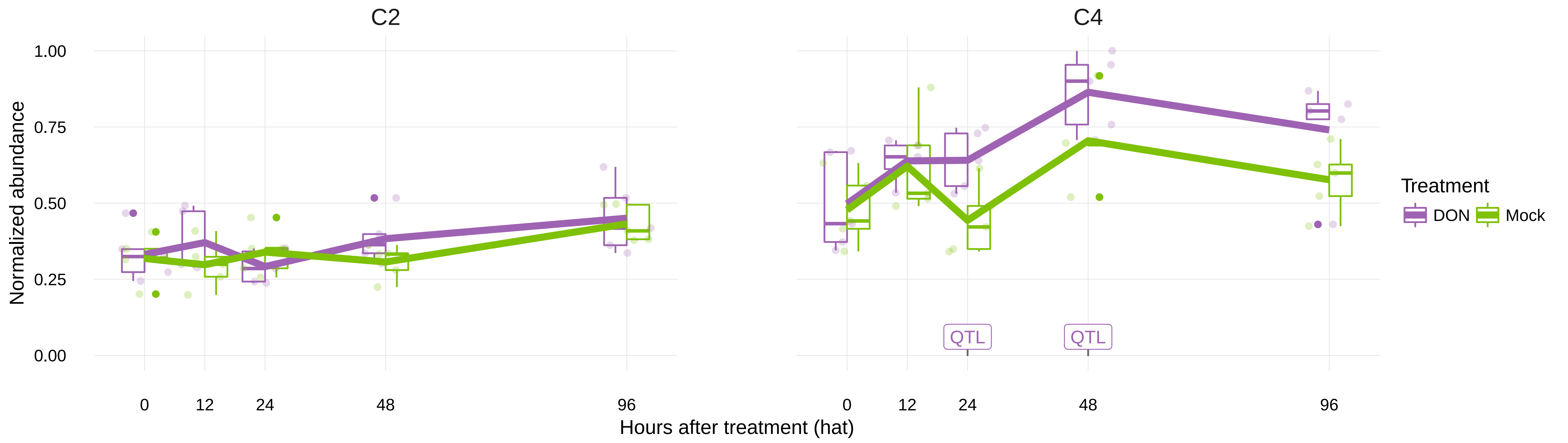

DON, Mock; different genotypes

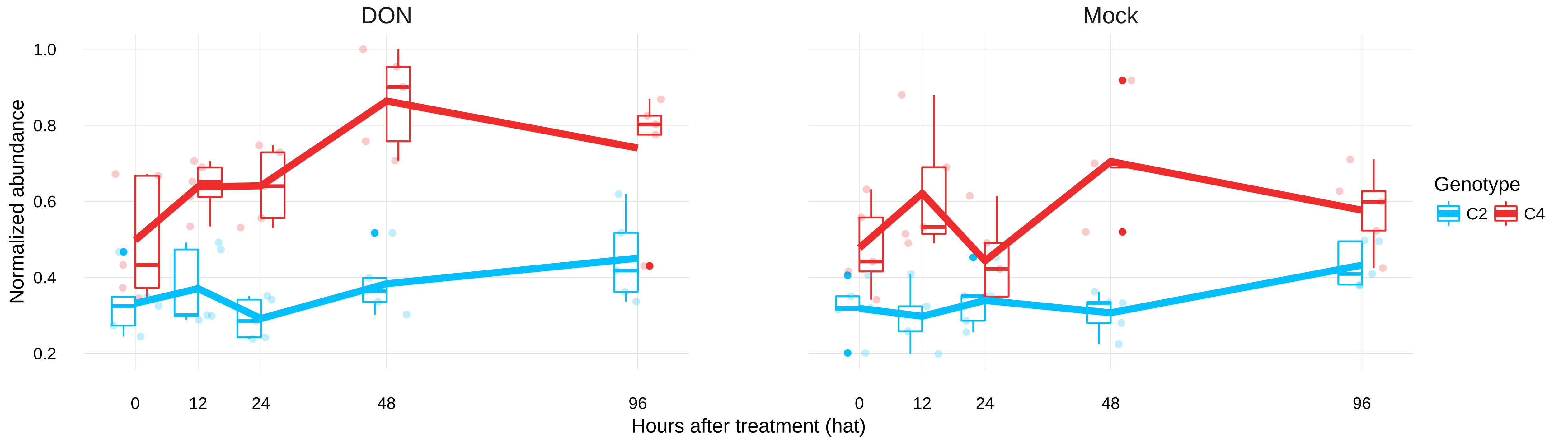

CM, Remus; different treatments

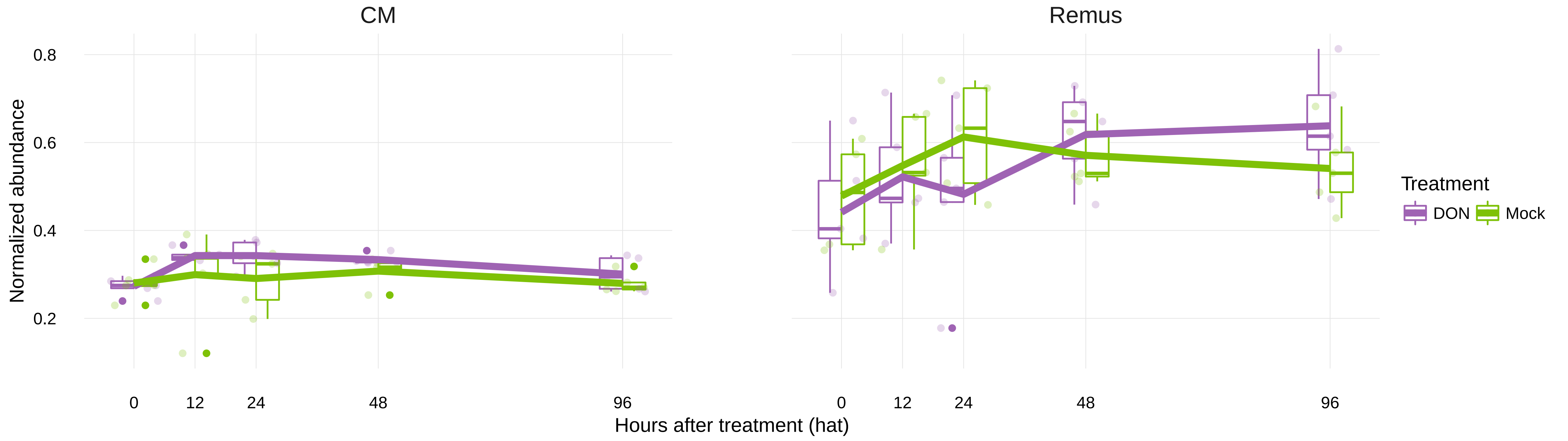

DON, Mock; all four genotypes

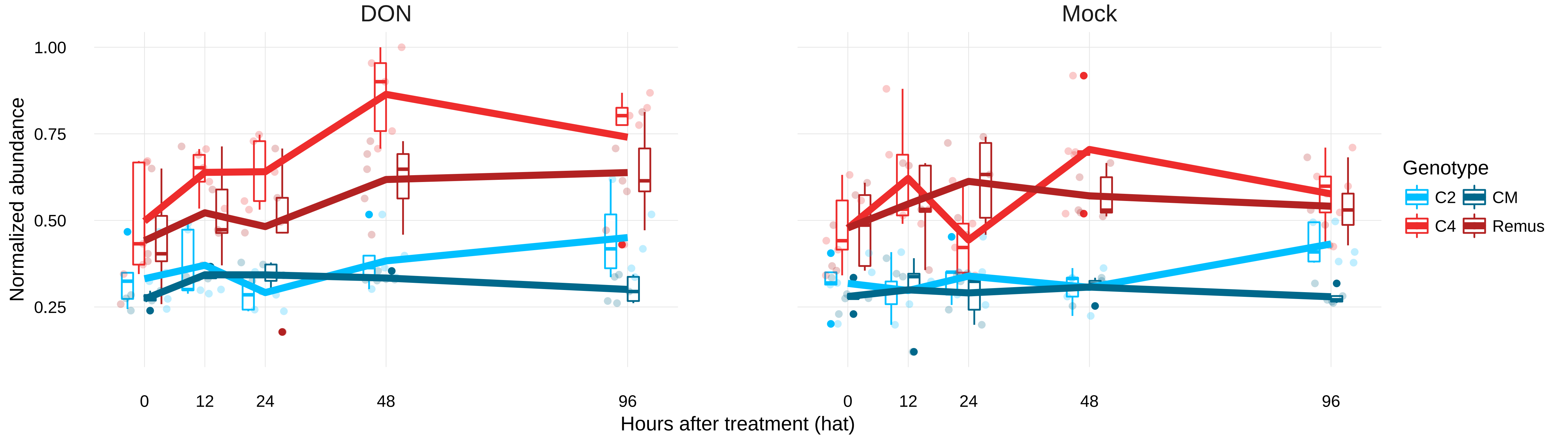

A.69

Annotated as Flavonoid (diGlcF)
(57 database hits)

MZ	565.1558
RT	13.44 min
Normalization	Directly via KPX samples
Cluster	Cluster 4
Cn total / Phe	26 / 9

C2, C4; different treatments

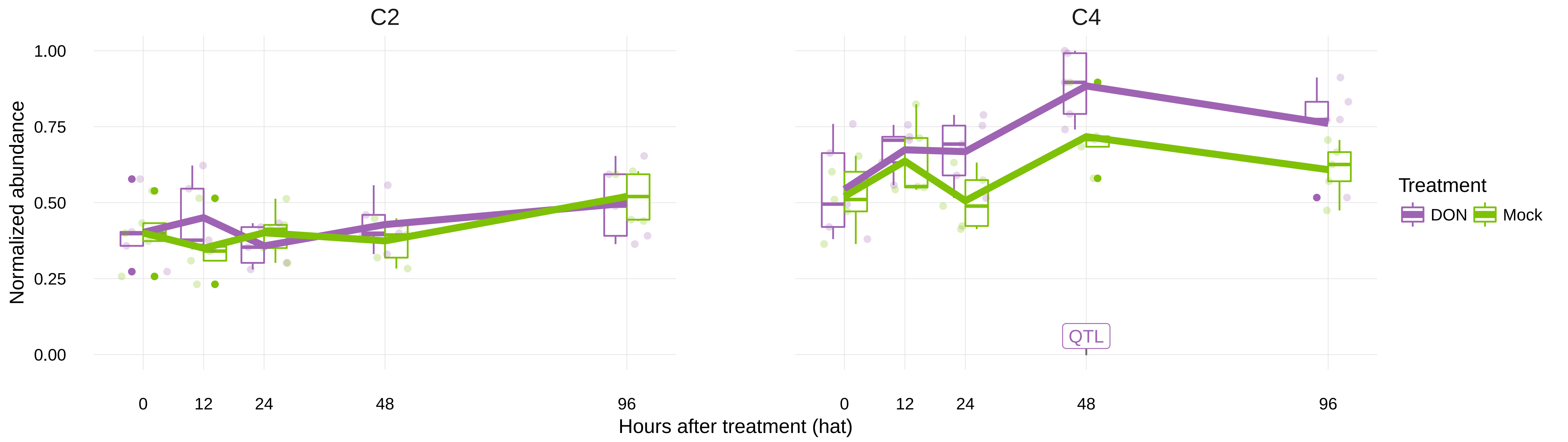

DON, Mock; different genotypes

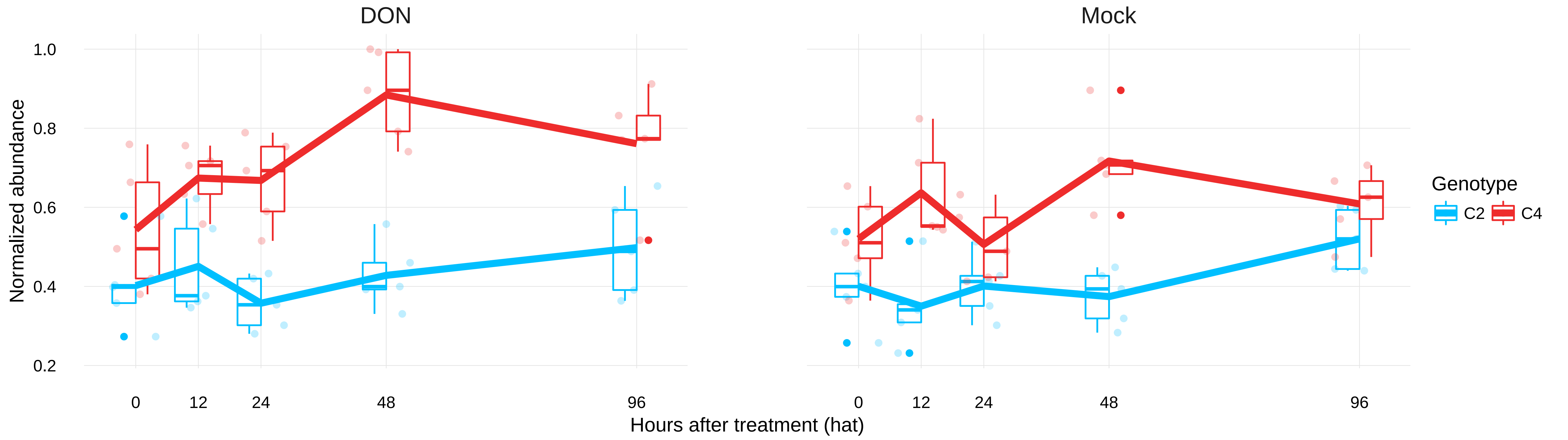

CM, Remus; different treatments

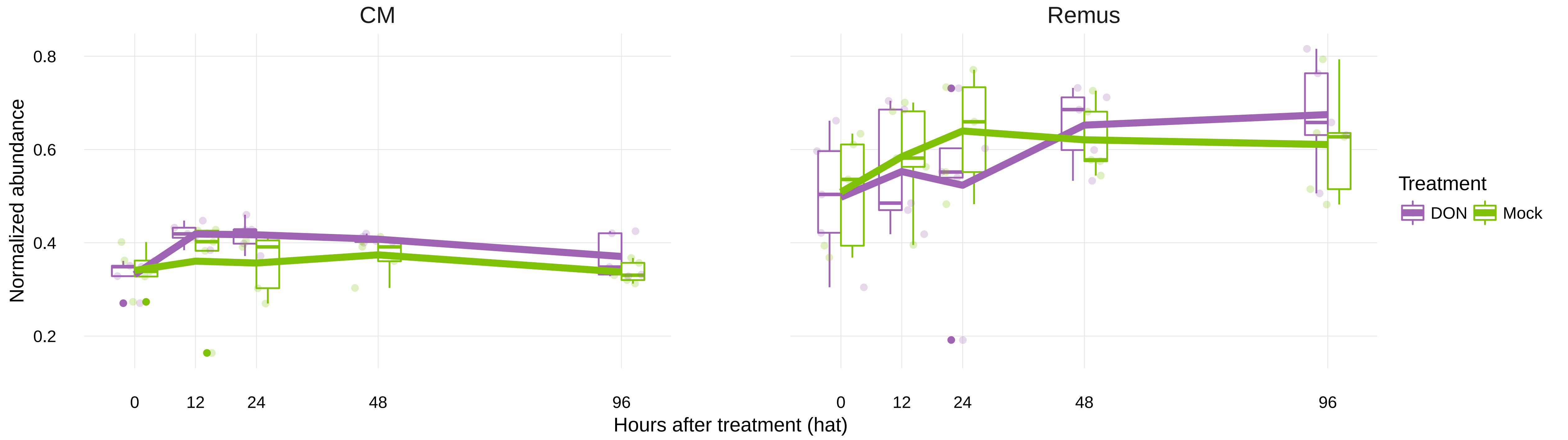

DON, Mock; all four genotypes

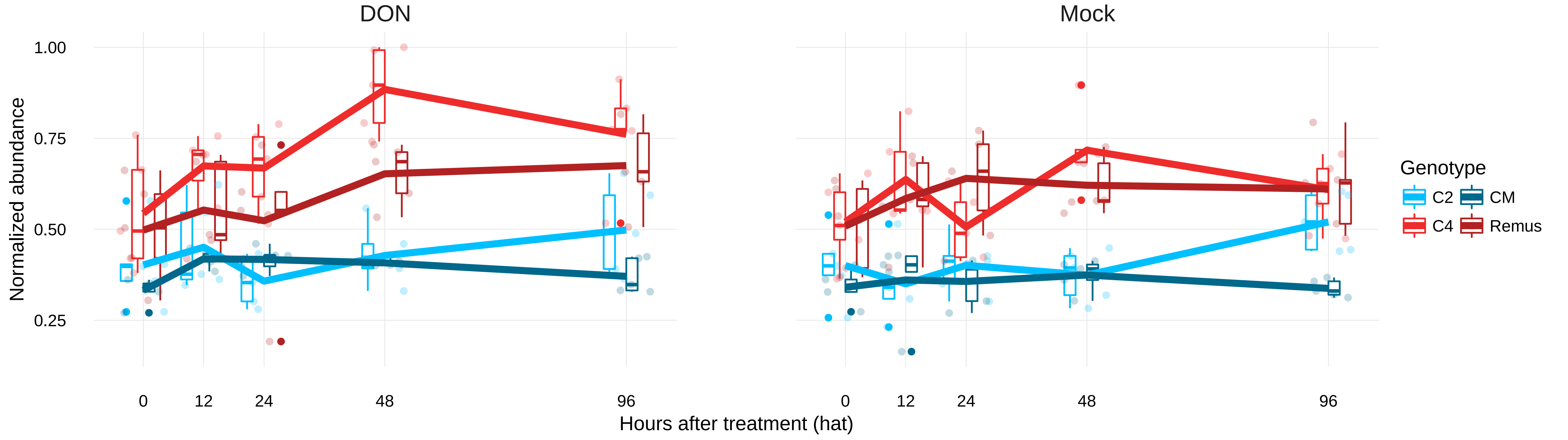

A.84

Annotated as Flavonoid (diGlcF)

(57 database hits)

MZ	565.1558
RT	14.9 min
Normalization	Directly via KPX samples
Cluster	Cluster 4
Cn total / Phe	26 /

C2, C4; different treatments

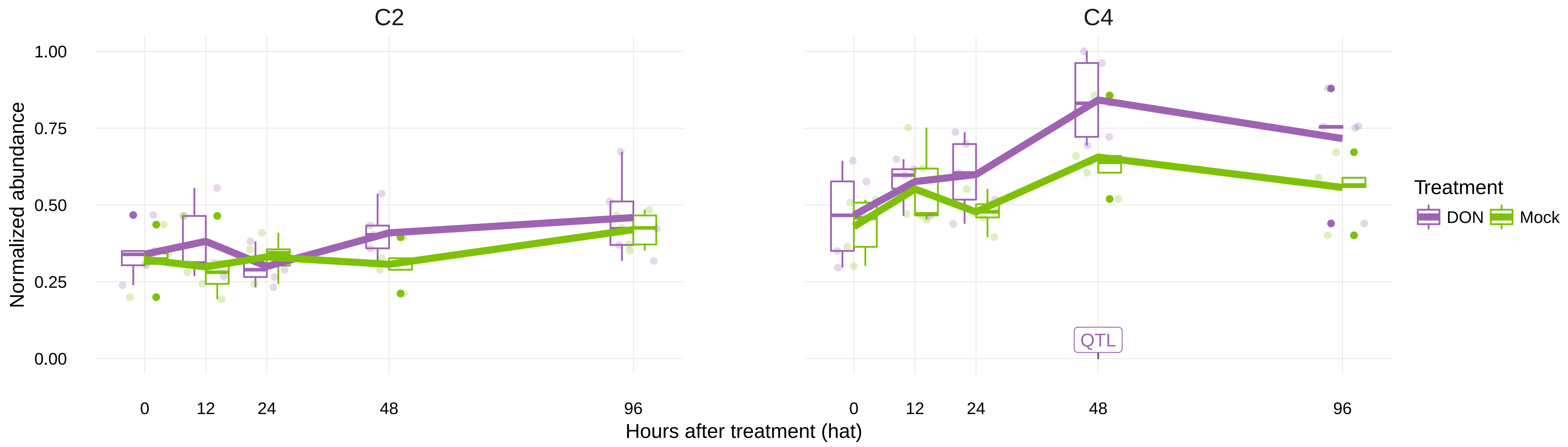

DON, Mock; different genotypes

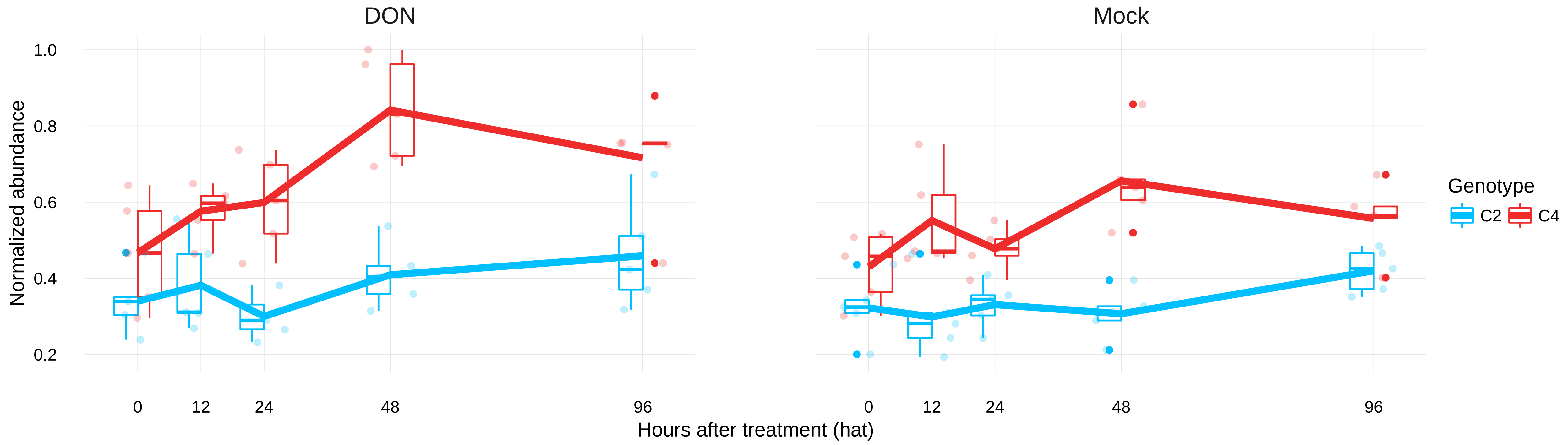

CM, Remus; different treatments

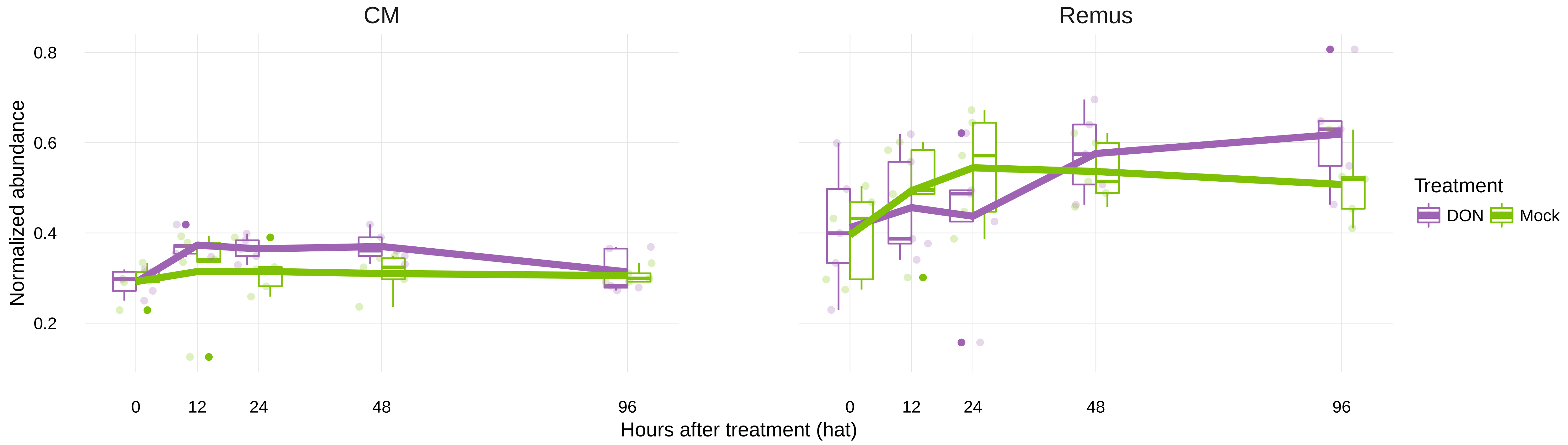

DON, Mock; all four genotypes

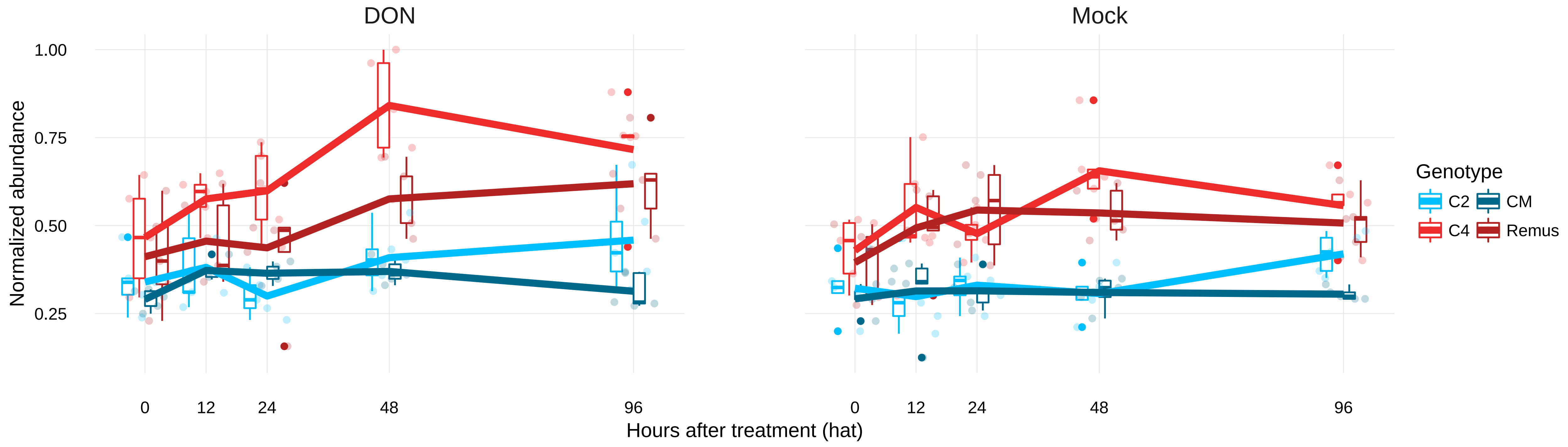

A.137

Annotated as Flavonoid or HCA derivative (GlcF)
(2 database hits)

MZ	527.1548
RT	22.01 min
Normalization	Directly via KPX samples
Cluster	–
Cn total / Phe	27 / 9

C2, C4; different treatments

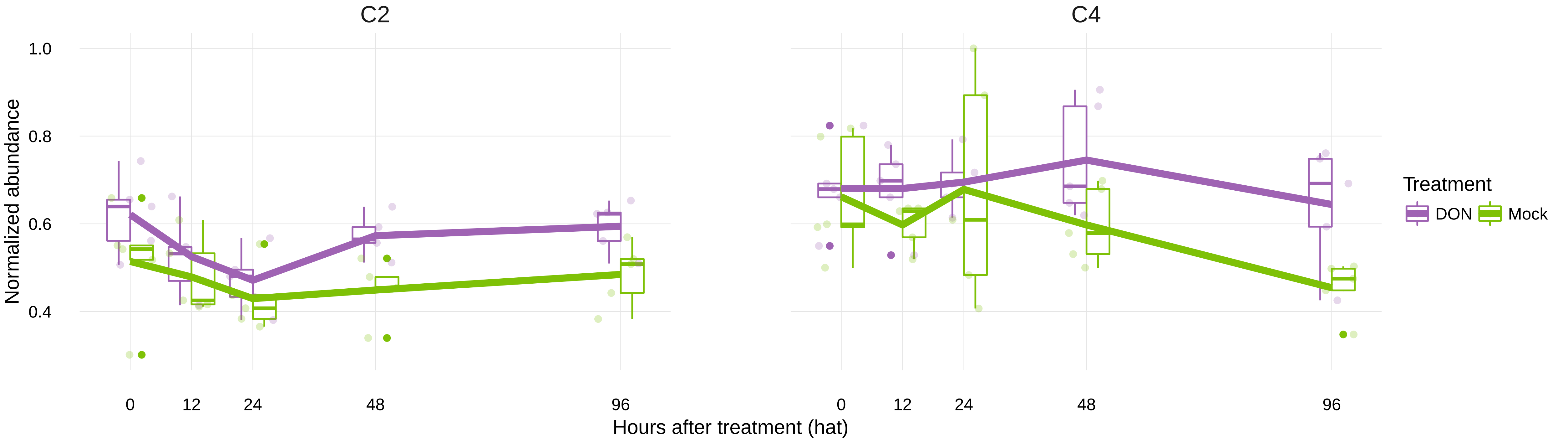

DON, Mock; different genotypes

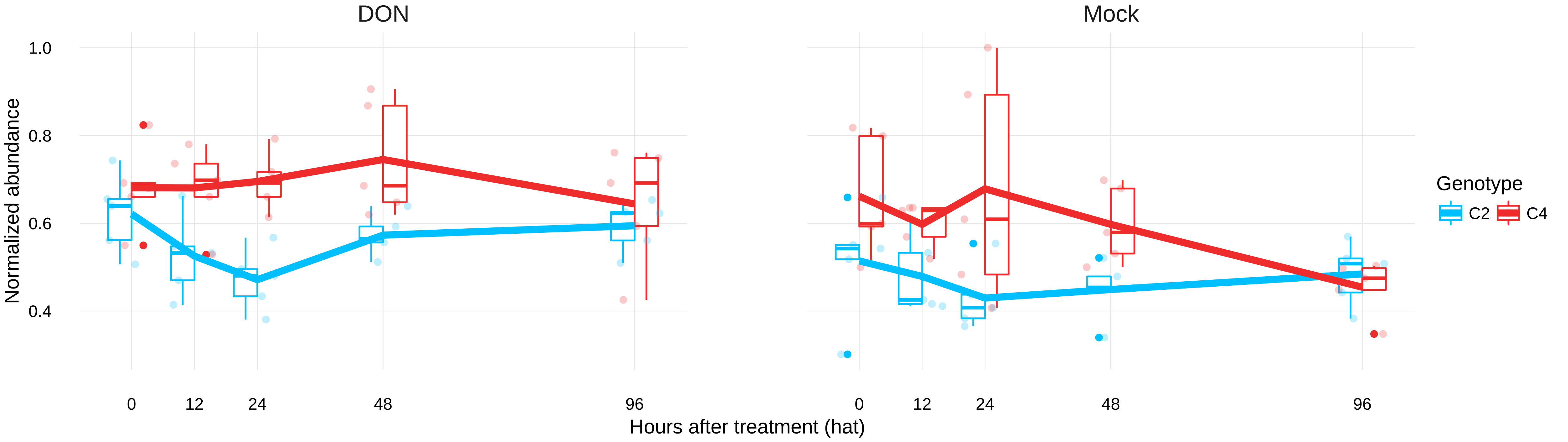

CM, Remus; different treatments

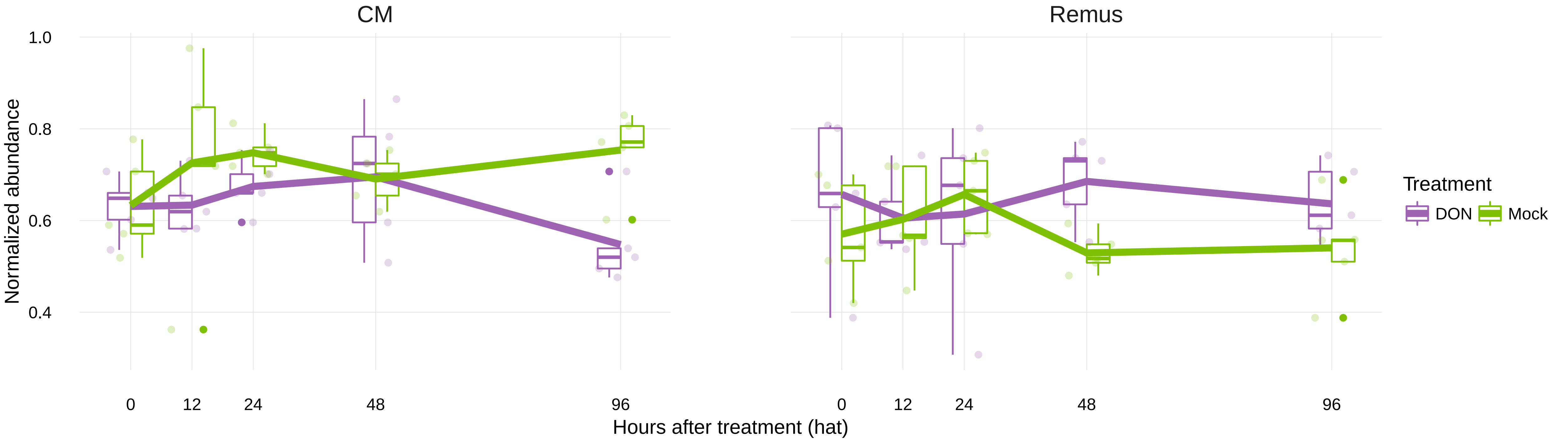

DON, Mock; all four genotypes

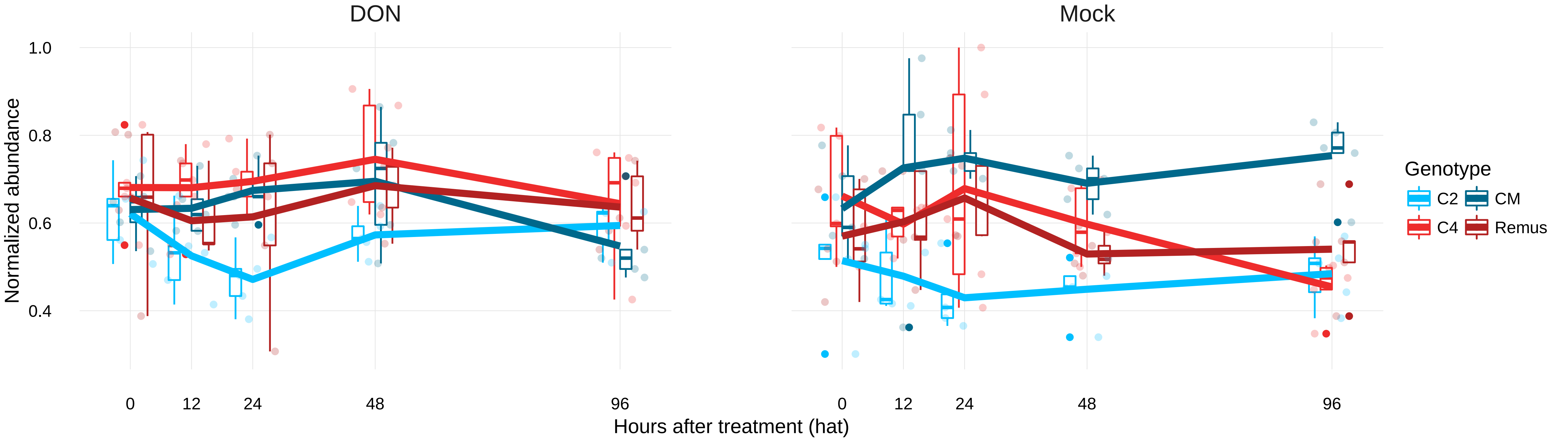

A.133

Annotated as Flavonoid or HCA derivative (GlcF)
(2 database hits)

MZ	527.1548
RT	21.45 min
Normalization	Directly via KPX samples
Cluster	–
Cn total / Phe	27 / 9

C2, C4; different treatments

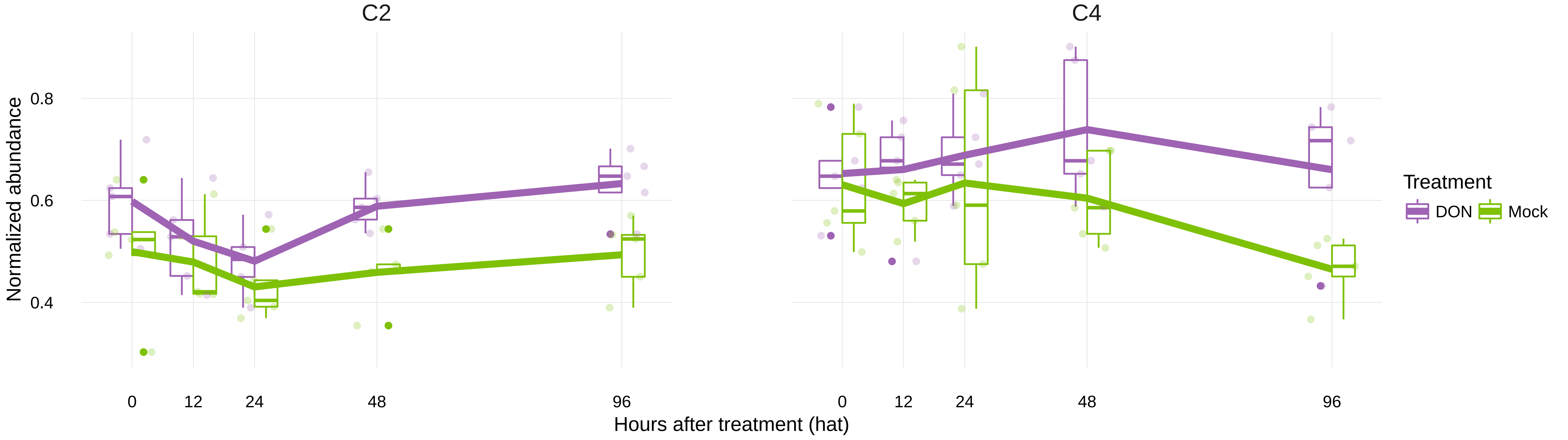

DON, Mock; different genotypes

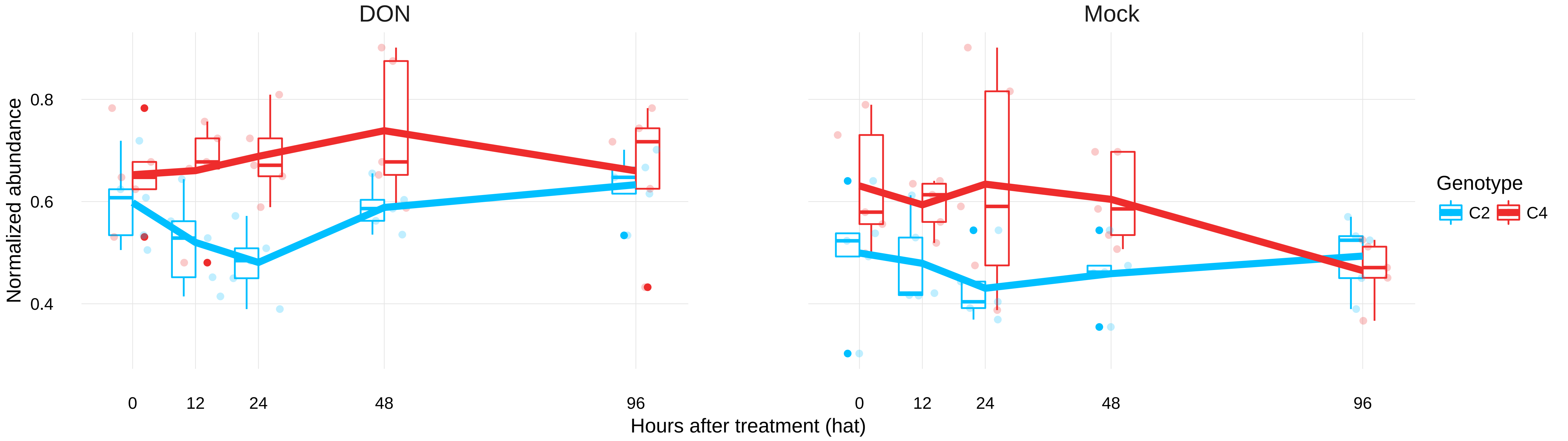

CM, Remus; different treatments

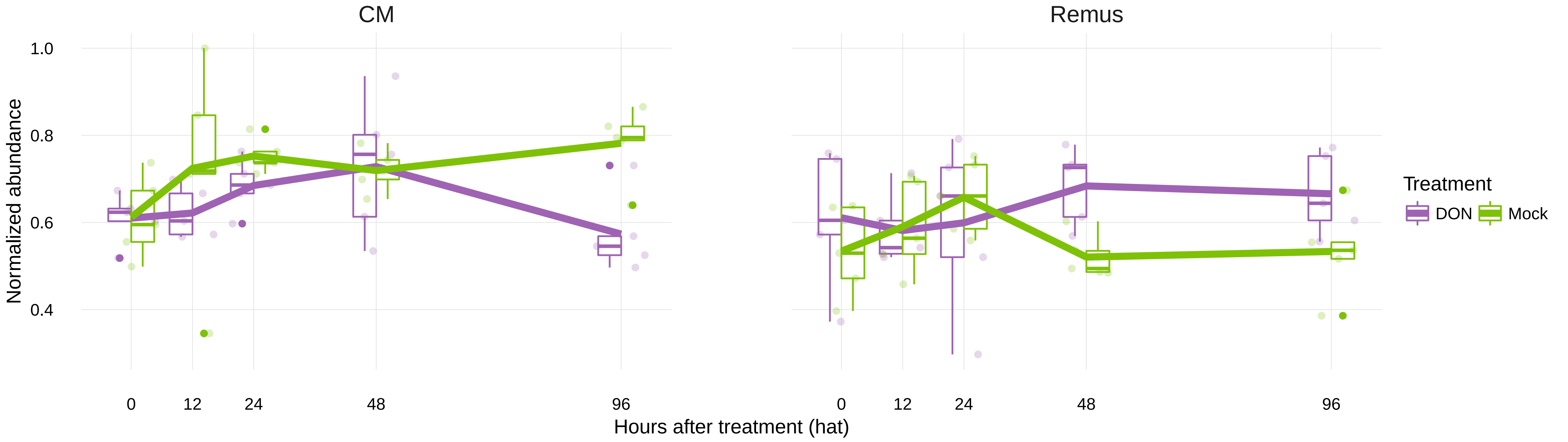

DON, Mock; all four genotypes

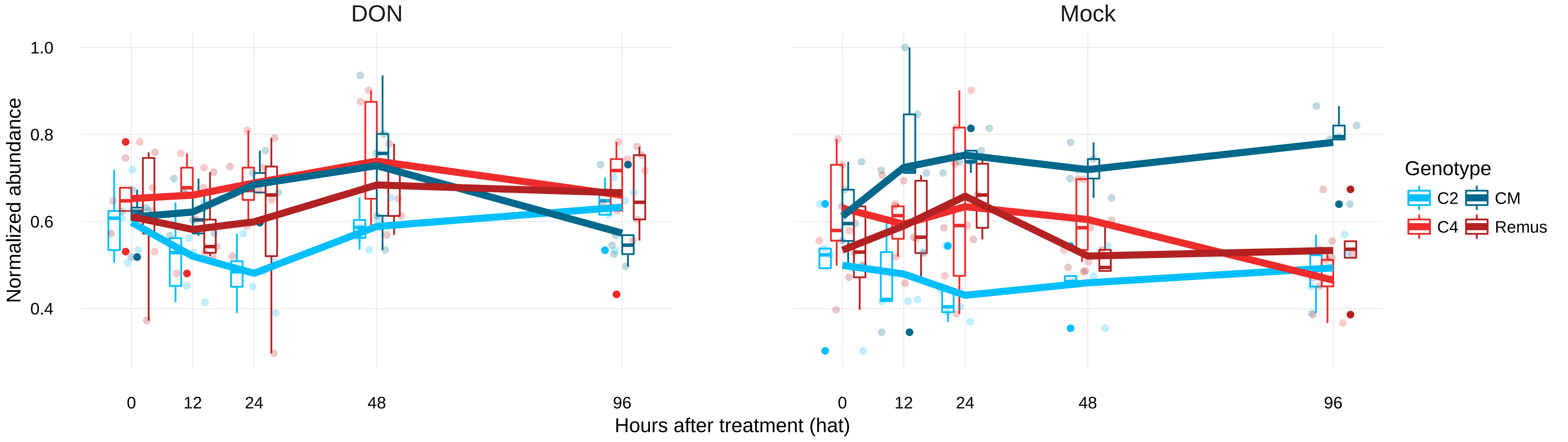

A.130

Annotated as Flavonoid (diF)

(1 database hit)

MZ	675.2072
RT	20.99 min
Normalization	Directly via KPX samples
Cluster	–
Cn total / Phe	36 /

C2, C4; different treatments

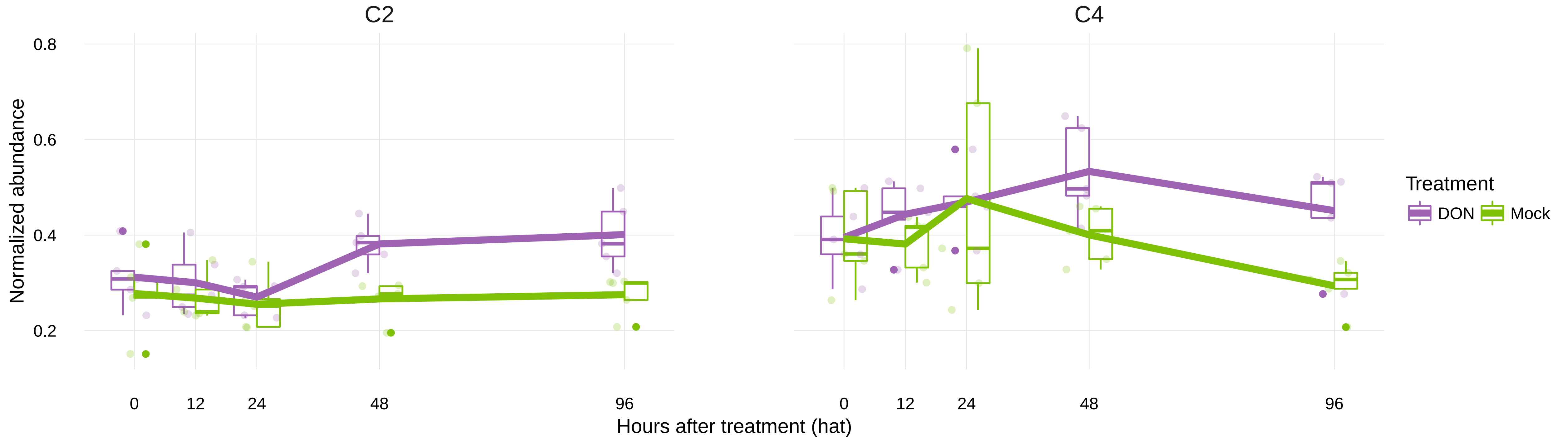

DON, Mock; different genotypes

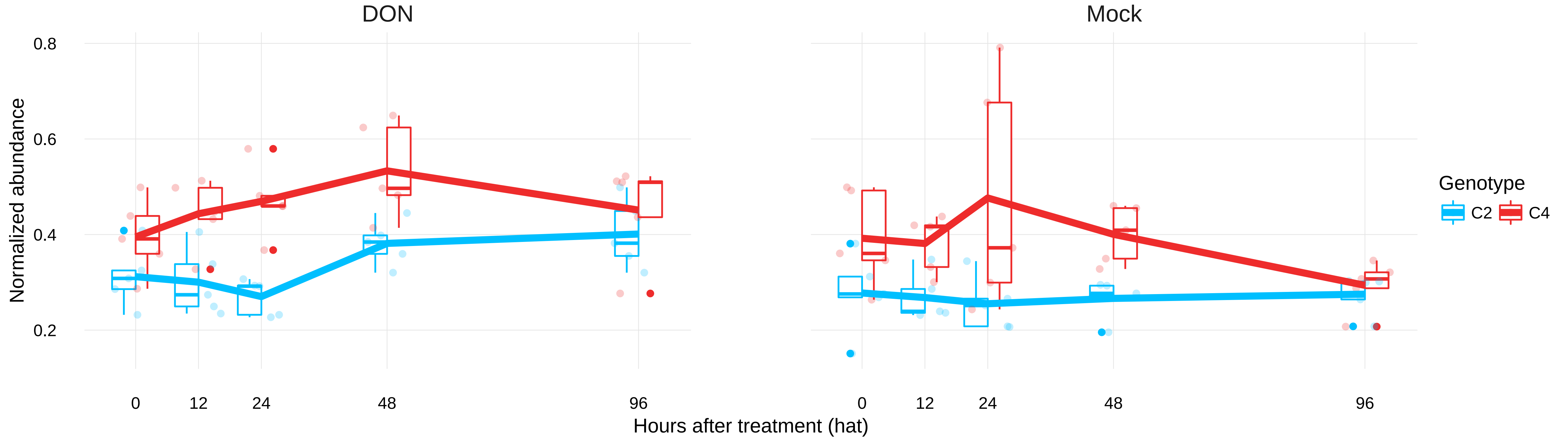

CM, Remus; different treatments

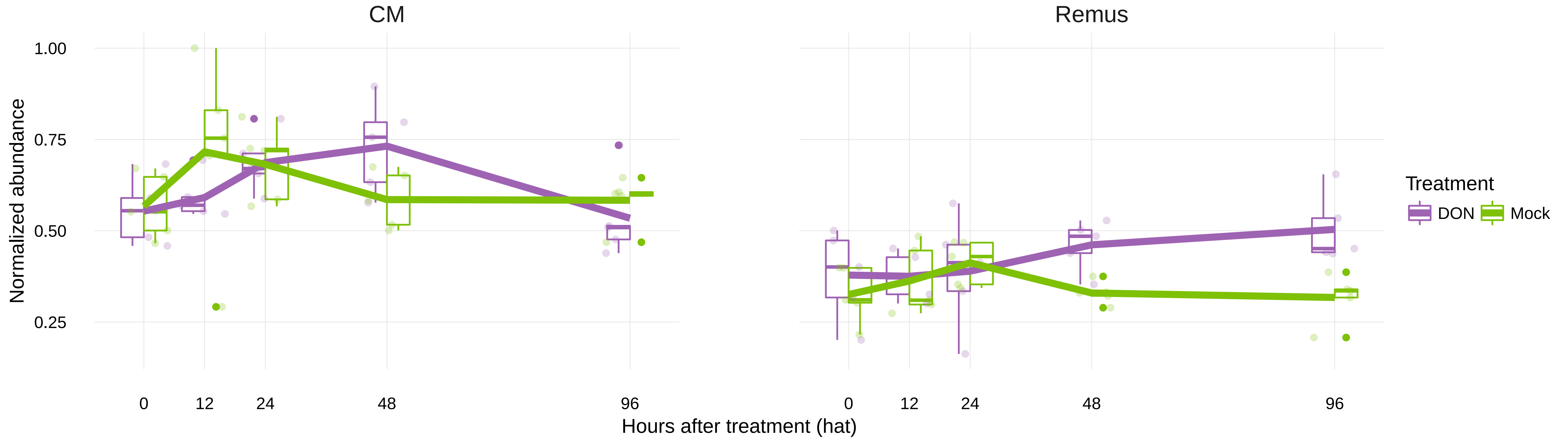

DON, Mock; all four genotypes

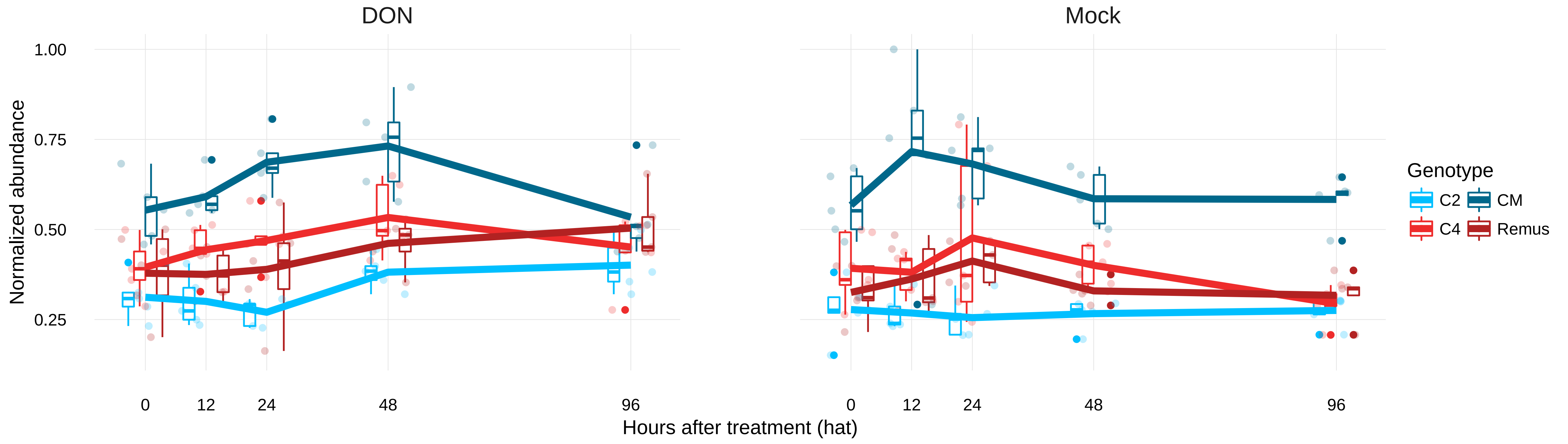

U.15

Unknown Phe−derived wheat−metabolite

MZ	331.082
RT	21.72 min
Normalization	Directly via KPX samples
Cluster	−
Cn total / Phe	18 / 18

C2, C4; different treatments

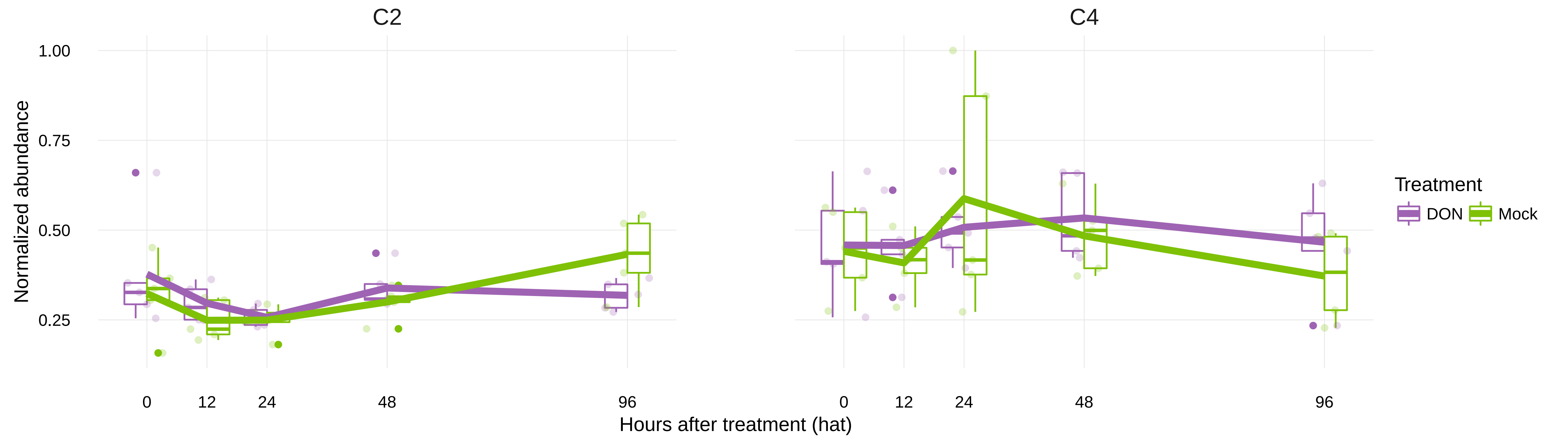

DON, Mock; different genotypes

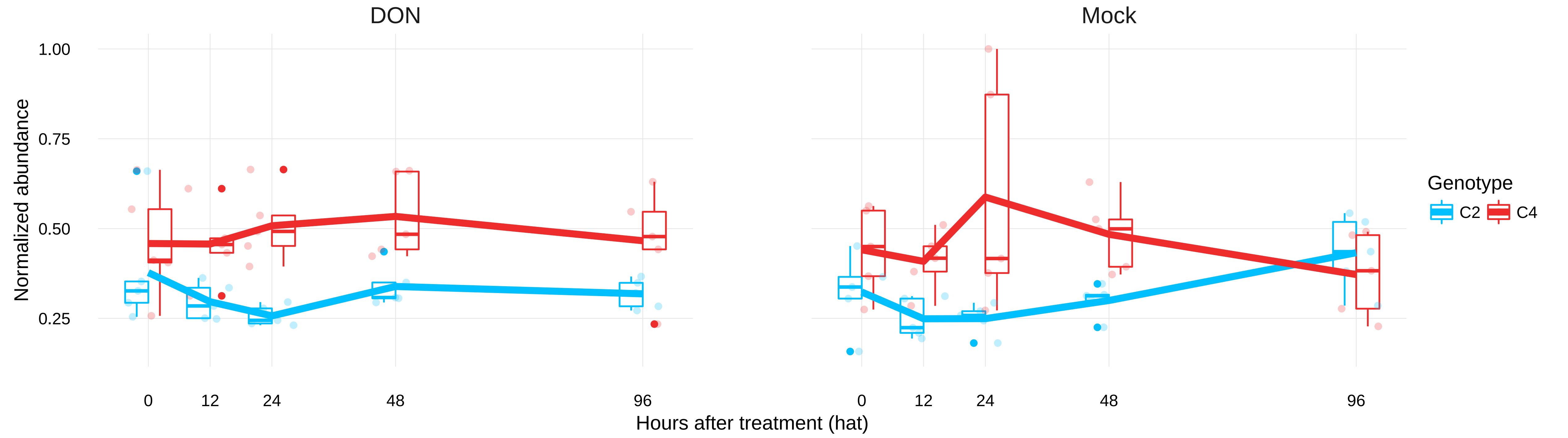

CM, Remus; different treatments

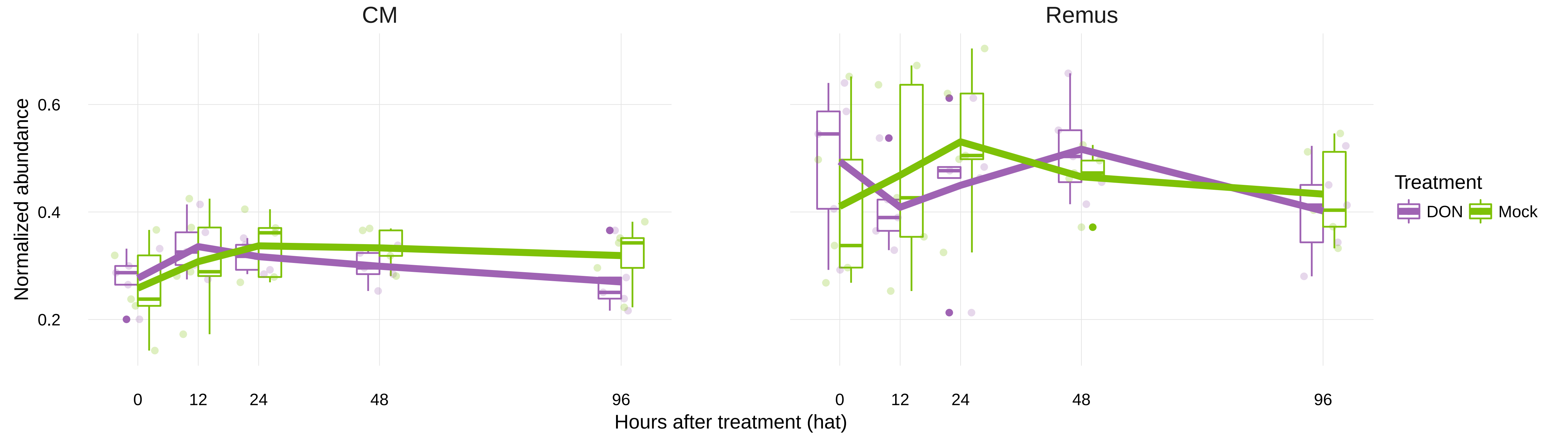

DON, Mock; all four genotypes

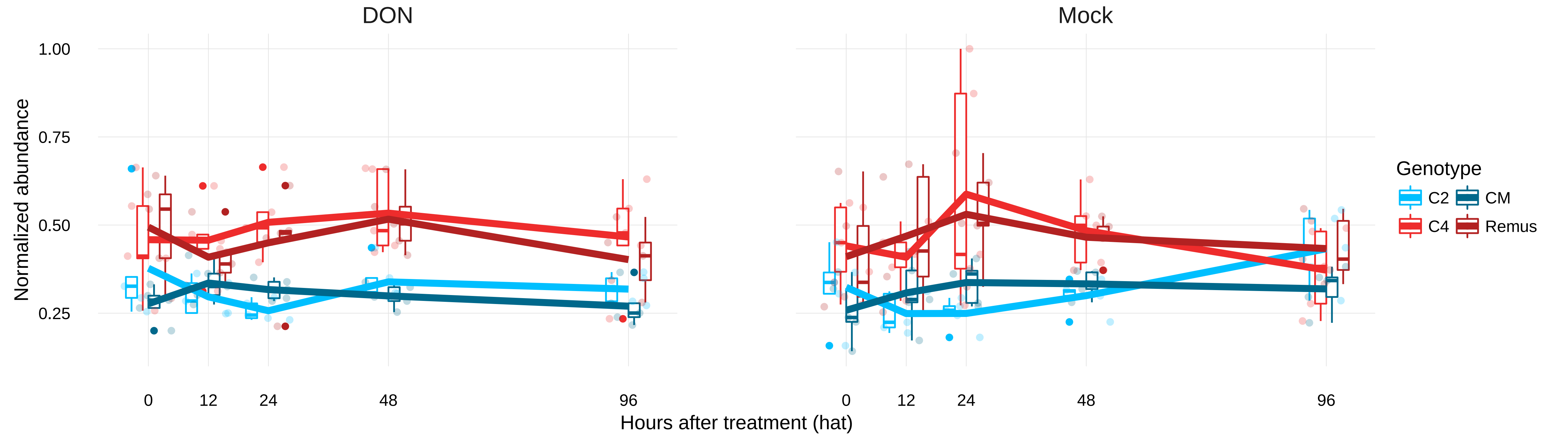

A.136

Annotated as Flavonoid
(74 database hits)

MZ	331.0814
RT	21.68 min
Normalization	Directly via KPX samples
Cluster	–
Cn total / Phe	17 / 9

C2, C4; different treatments

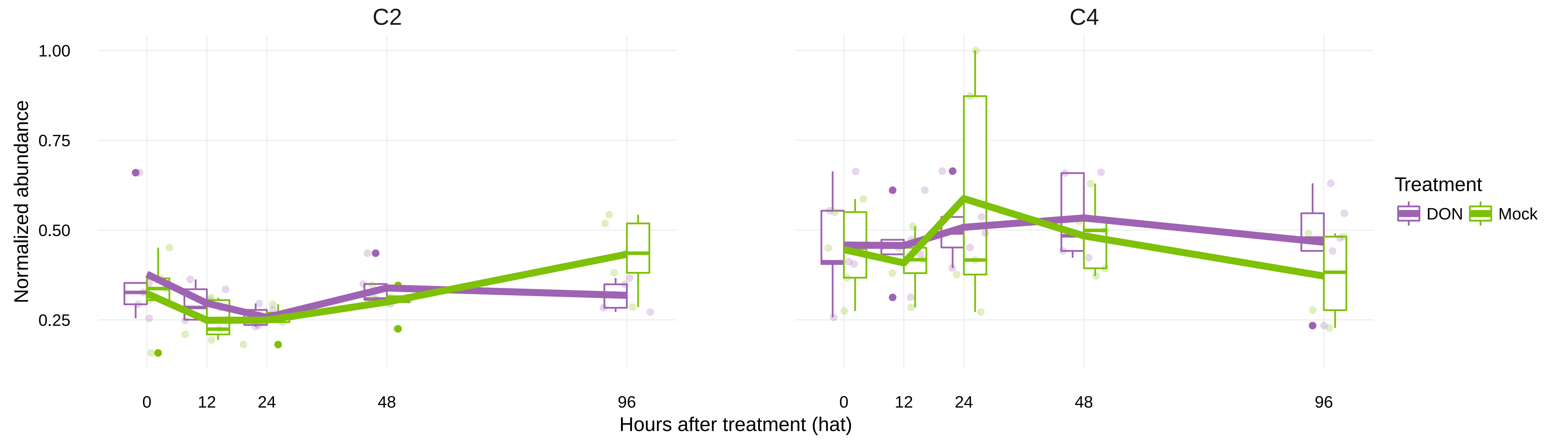

DON, Mock; different genotypes

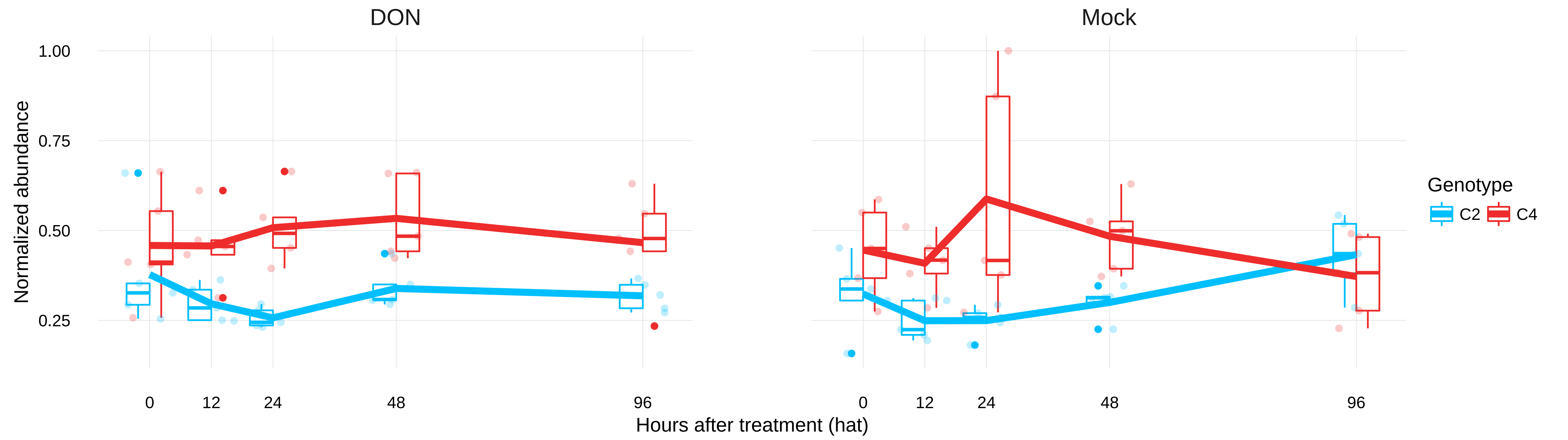

CM, Remus; different treatments

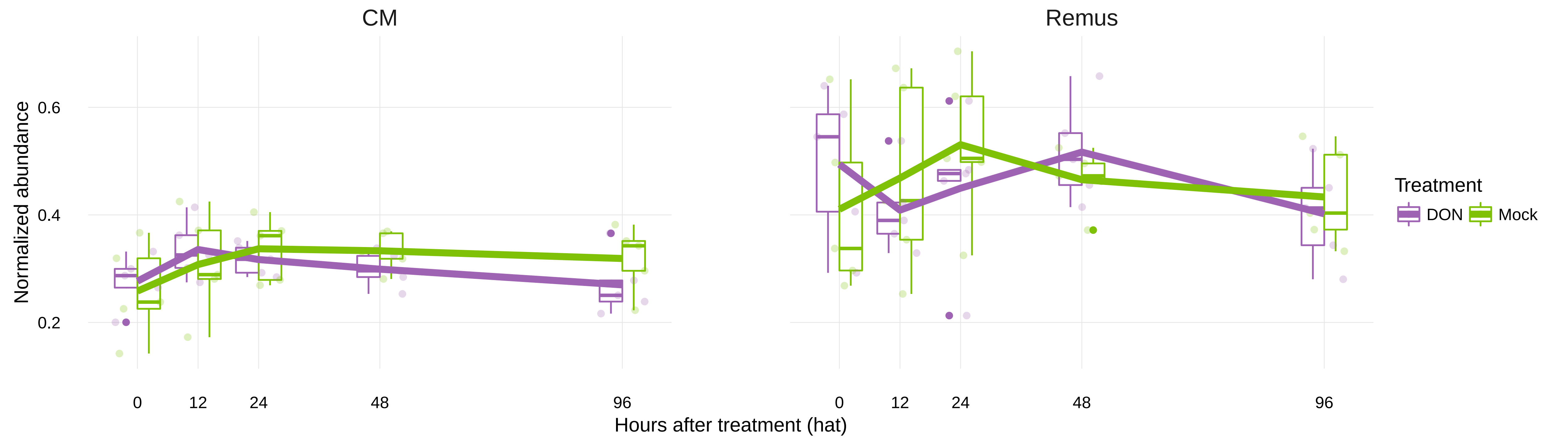

DON, Mock; all four genotypes

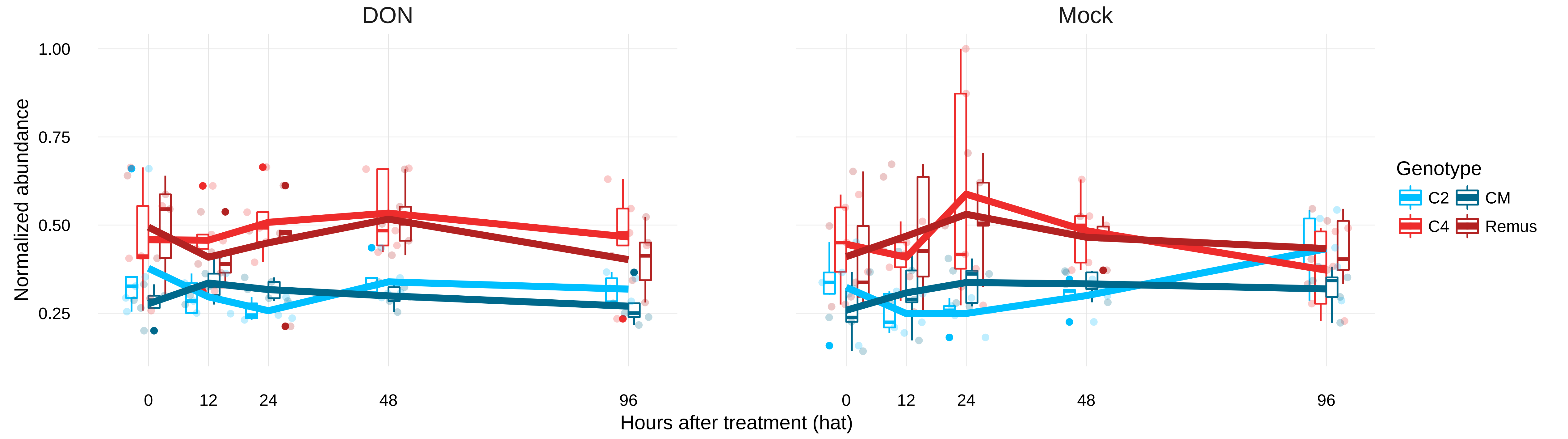

A.83

Annotated as Flavonoid (GlcF)

(6 database hits)

MZ	557.1631
RT	14.88 min
Normalization	Directly via KPX samples
Cluster	–
Cn total / Phe	26 /

C2, C4; different treatments

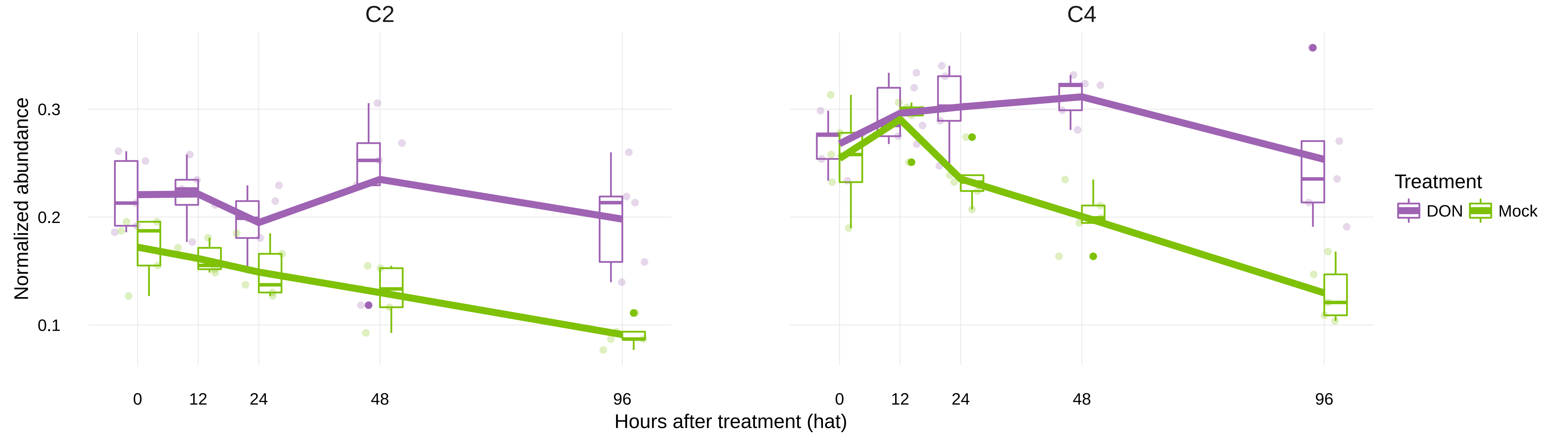

DON, Mock; different genotypes

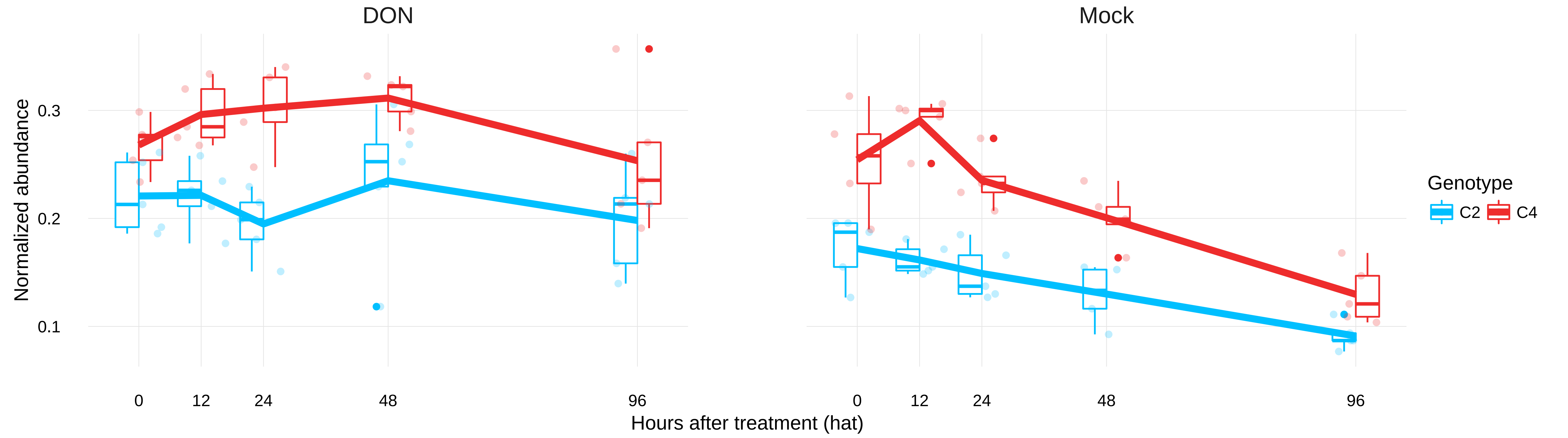

CM, Remus; different treatments

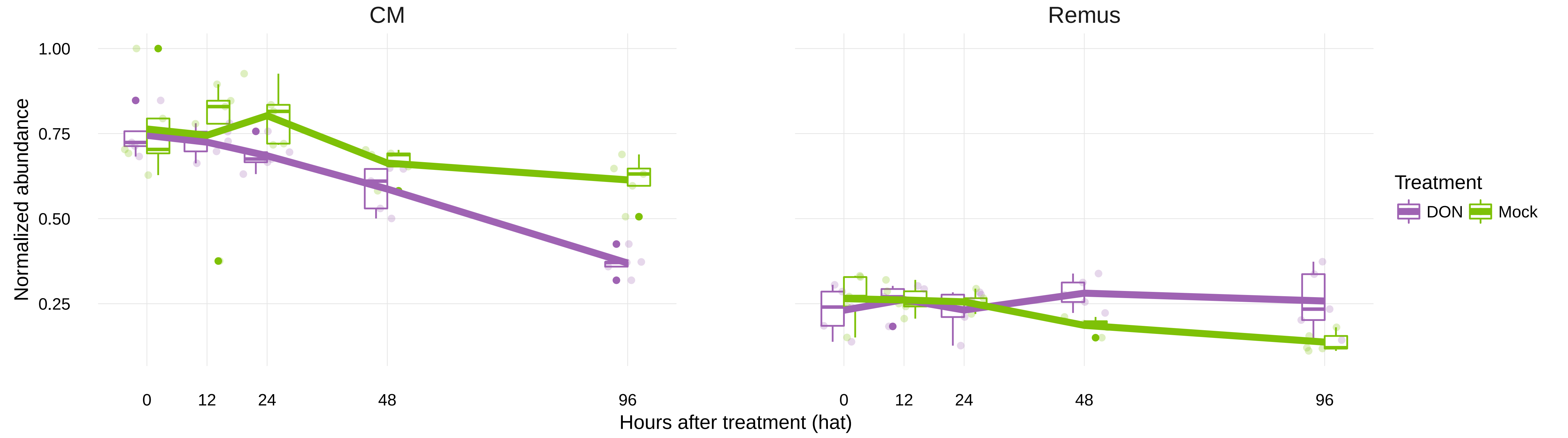

DON, Mock; all four genotypes

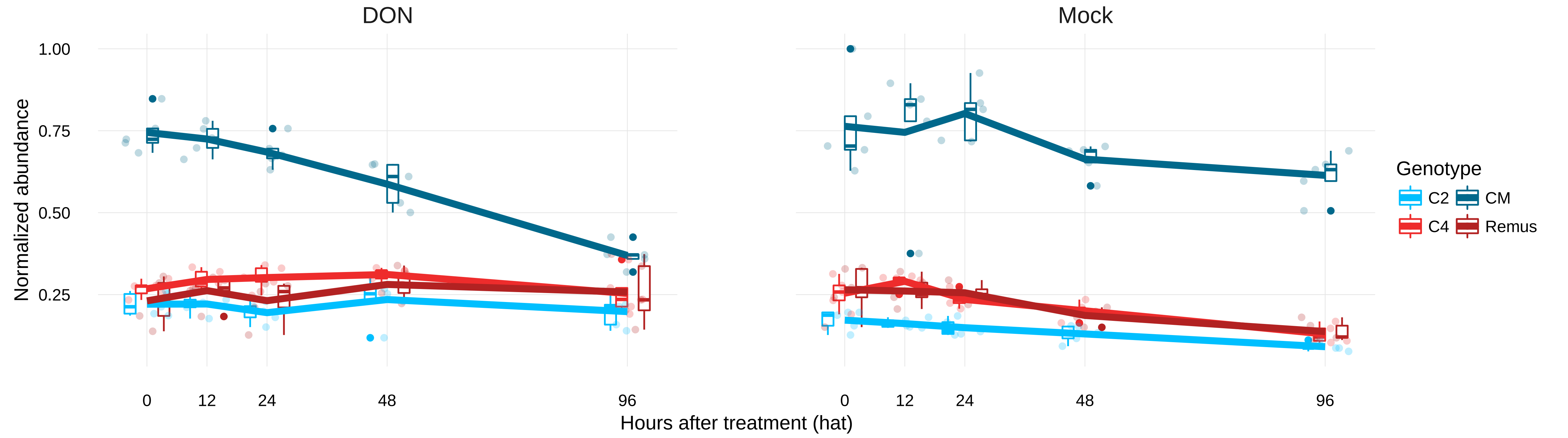

A.45

Annotated as Flavonoid

(30 database hits)

MZ	373.1284
RT	11.58 min
Normalization	Directly via KPX samples
Cluster	–
Cn total / Phe	20 /

C2, C4; different treatments

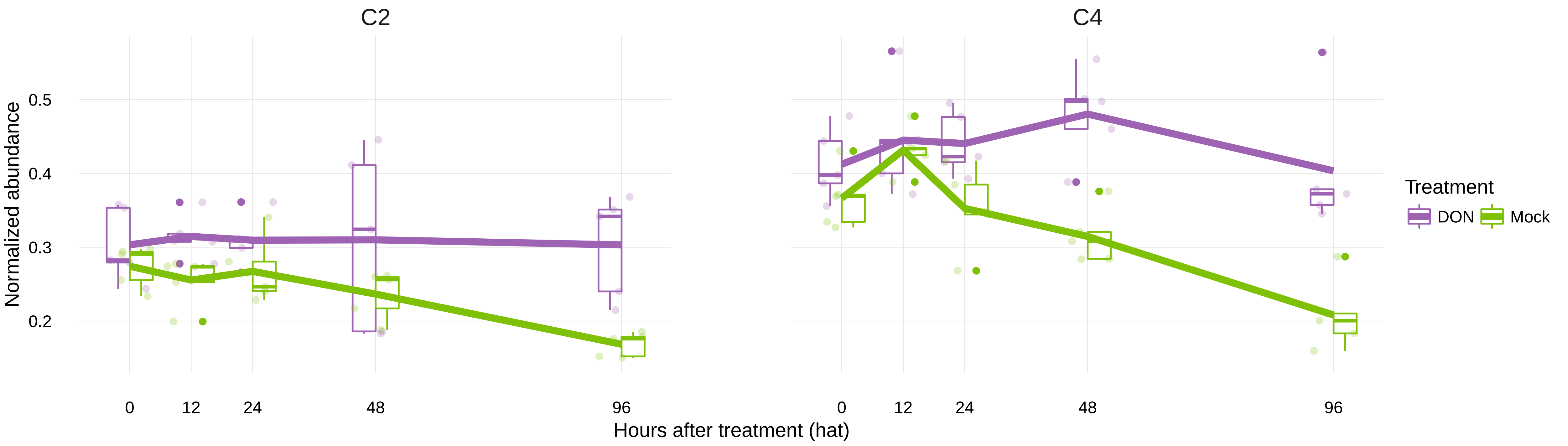

DON, Mock; different genotypes

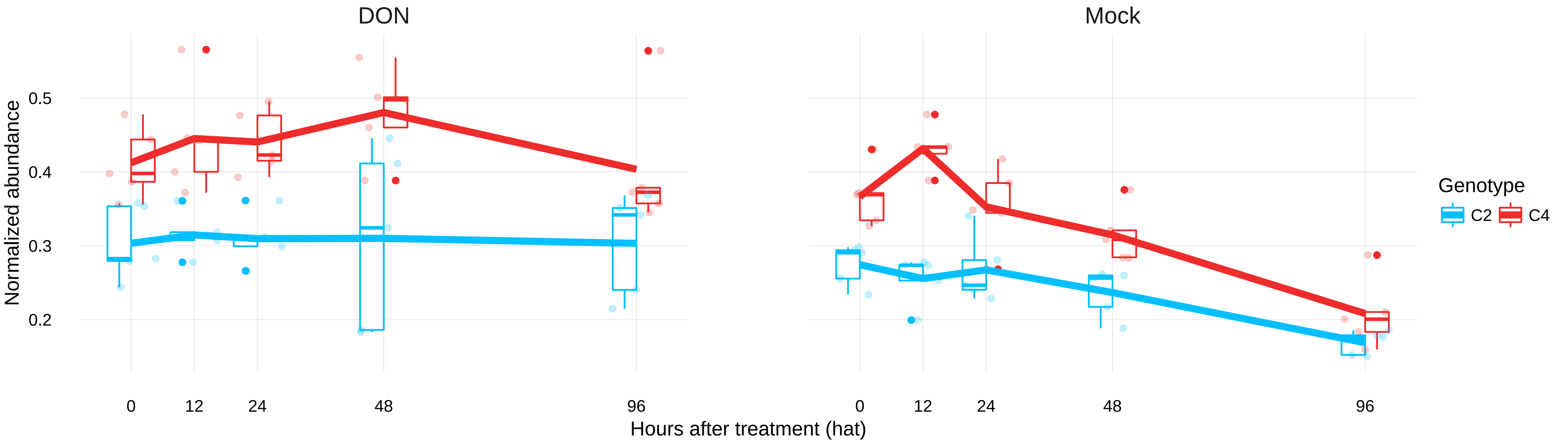

CM, Remus; different treatments

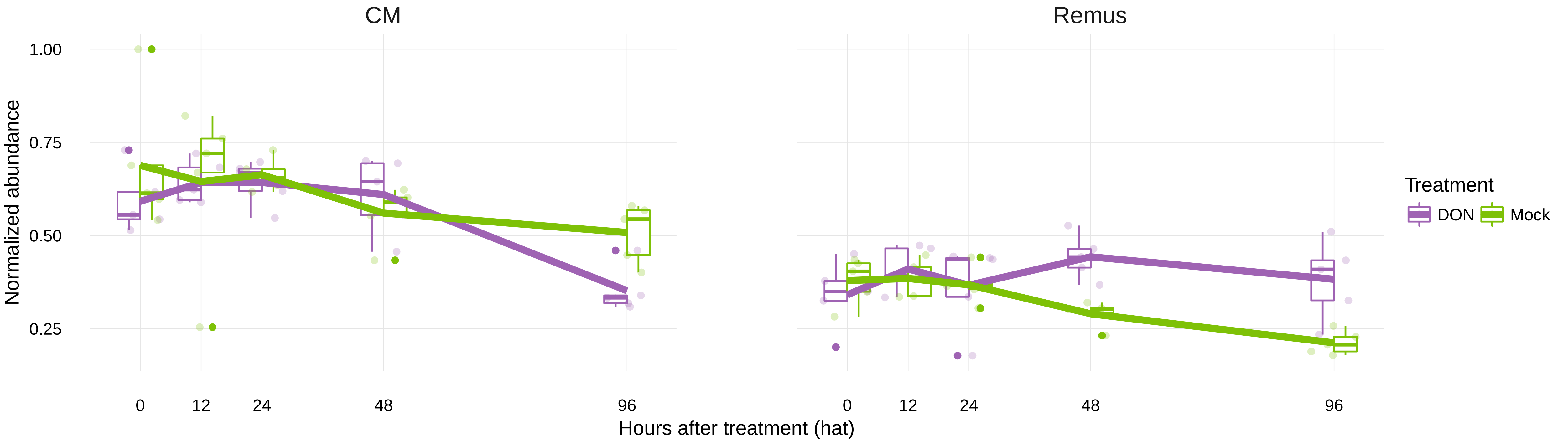

DON, Mock; all four genotypes

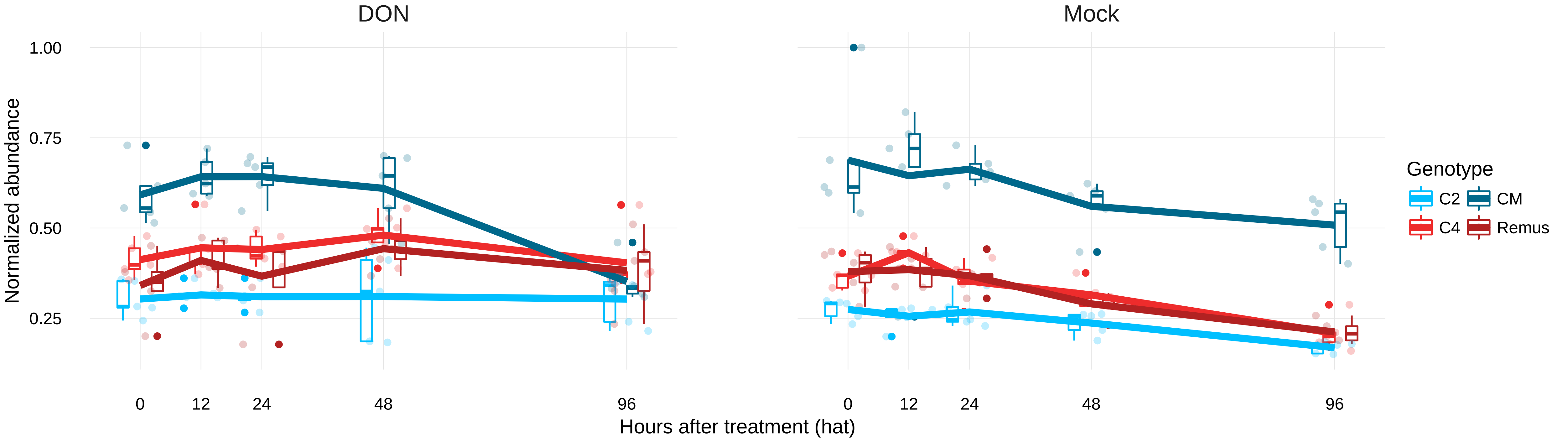

A.39

Annotated as others (Coumarins)
(1 database hit)

MZ	177.0546
RT	10.3 min
Normalization	Indirectly via surrogate in the KPX samples
Cluster	–
Cn total / Phe	10 / 9

C2, C4; different treatments

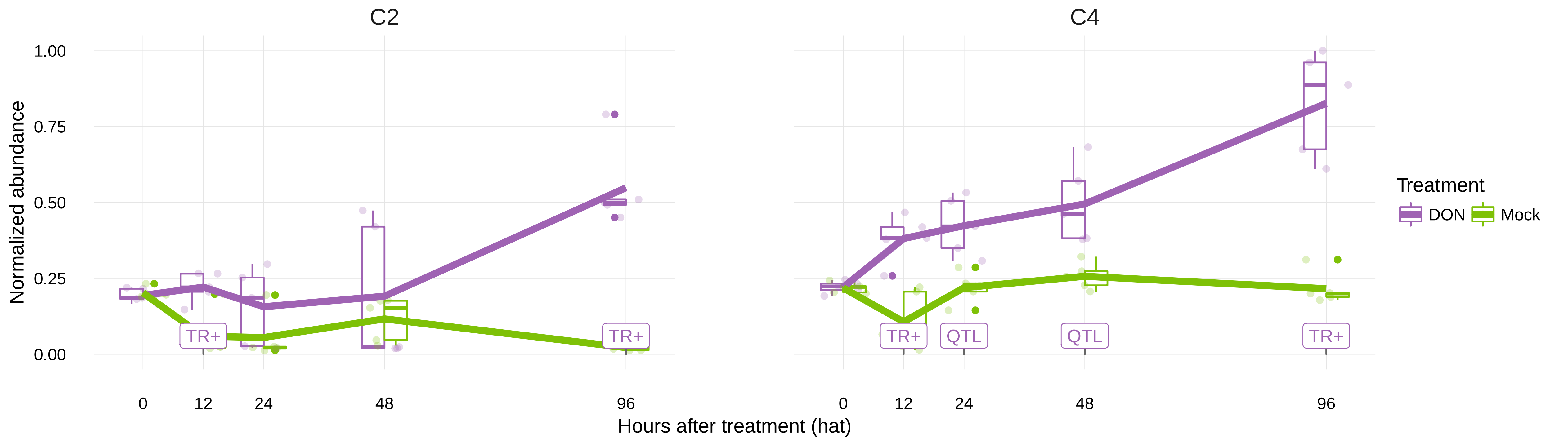

DON, Mock; different genotypes

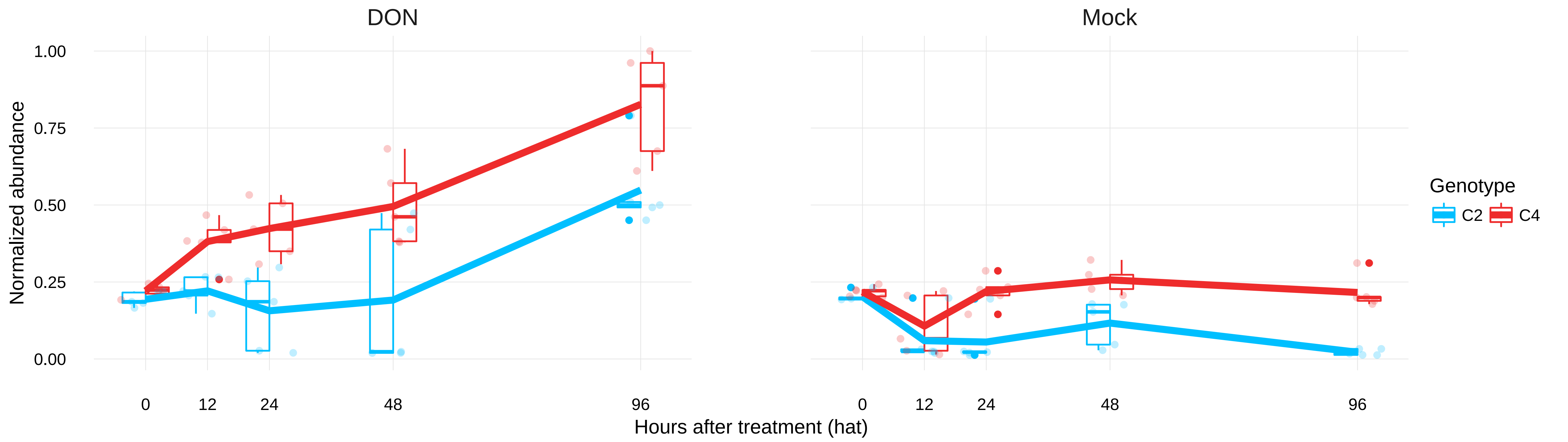

CM, Remus; different treatments

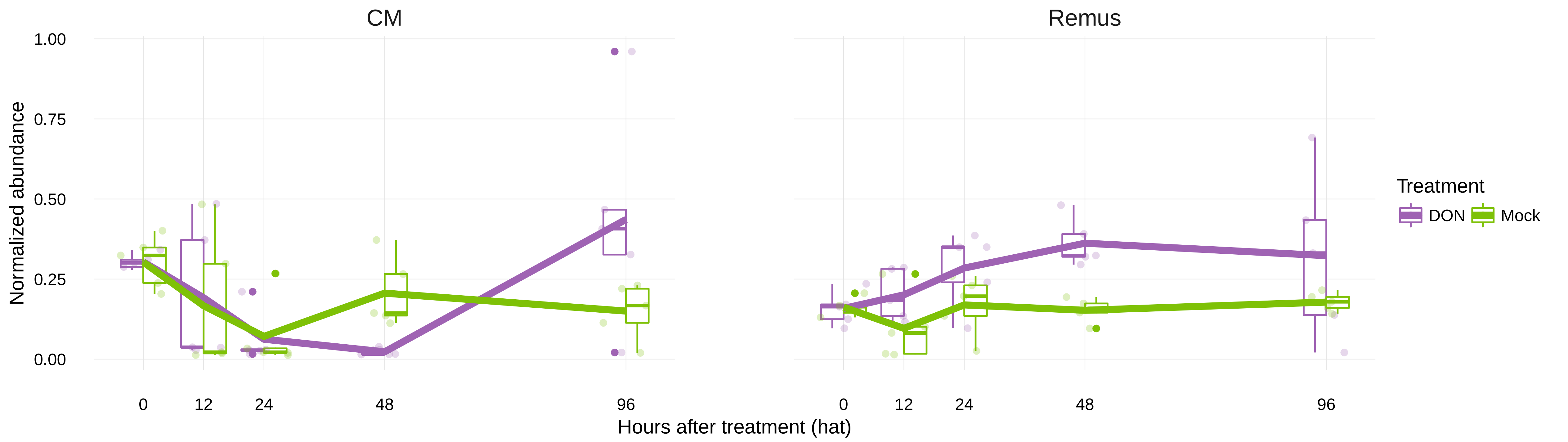

DON, Mock; all four genotypes

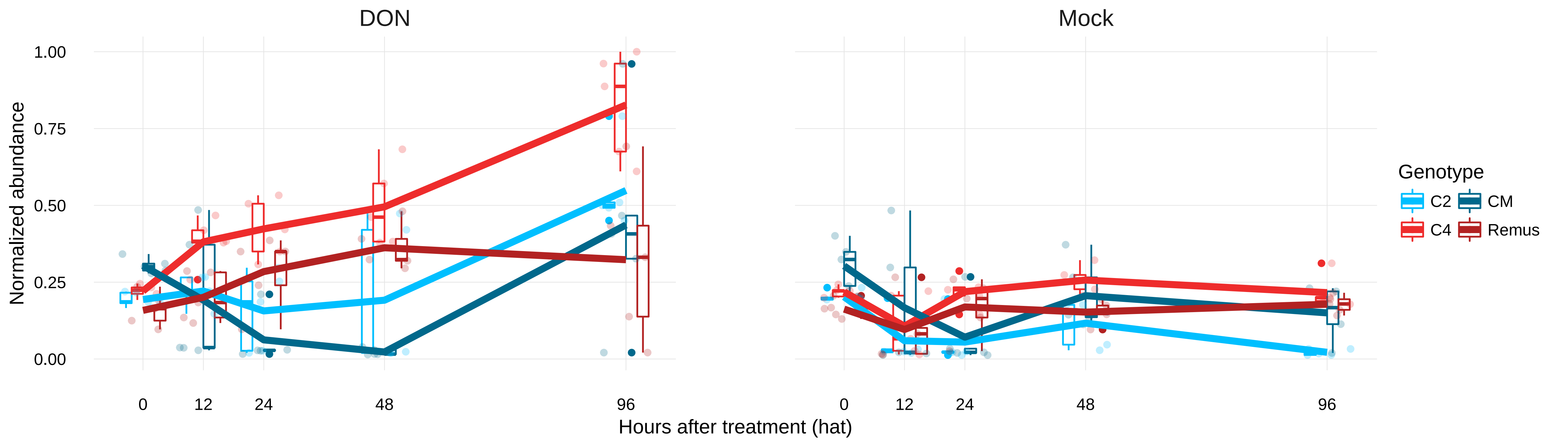

A.47

Annotated as HCA derivative
(1 database hit)

MZ	369.1182
RT	11.61 min
Normalization	Directly via KPX samples
Cluster	–
Cn total / Phe	17 /

C2, C4; different treatments

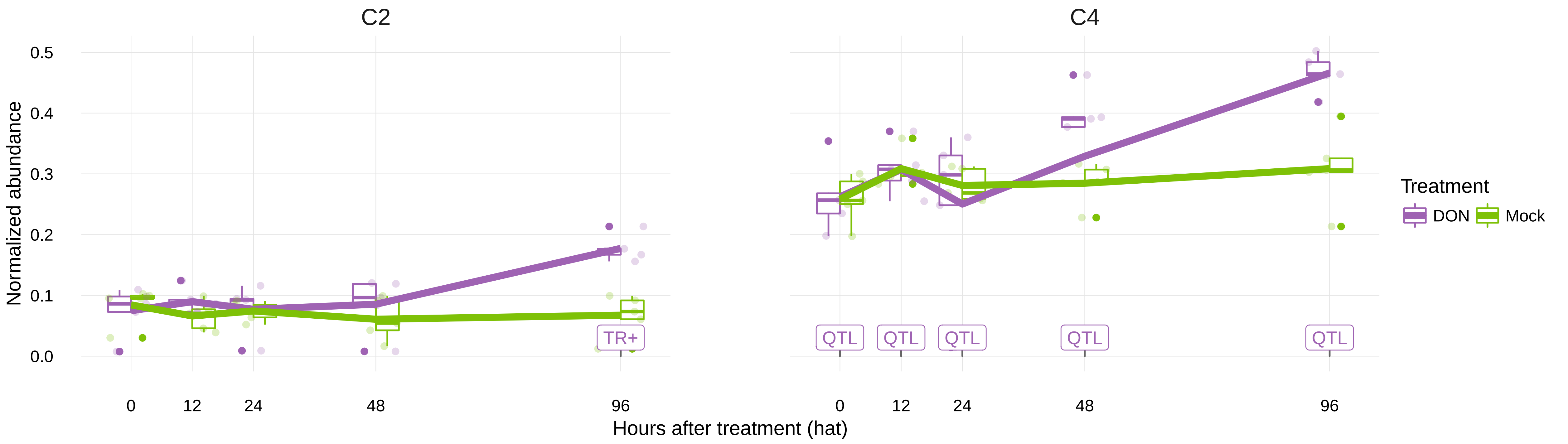

DON, Mock; different genotypes

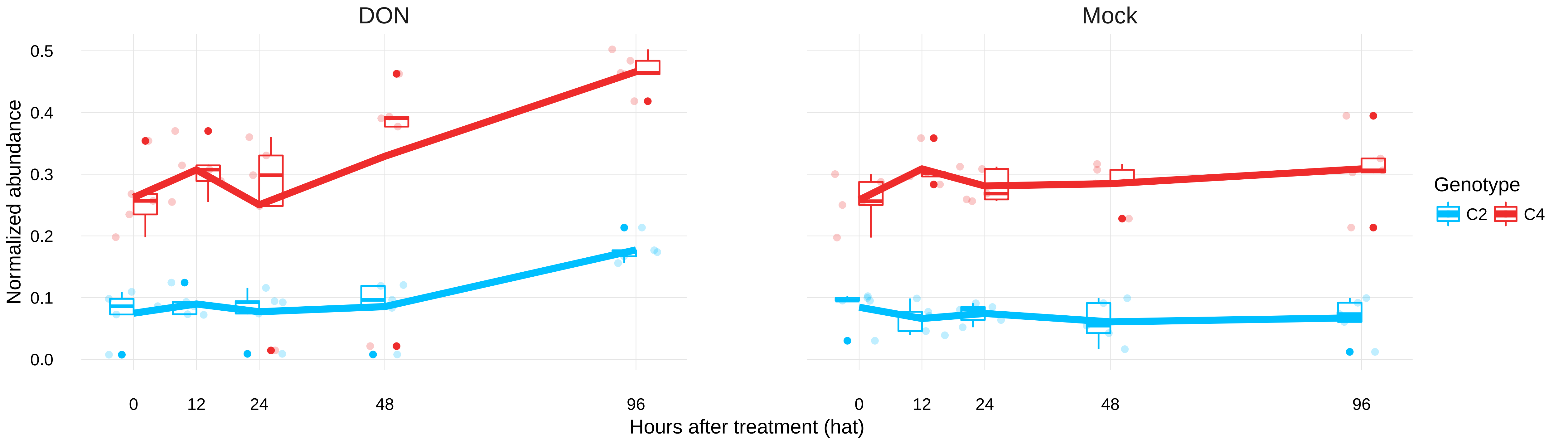

CM, Remus; different treatments

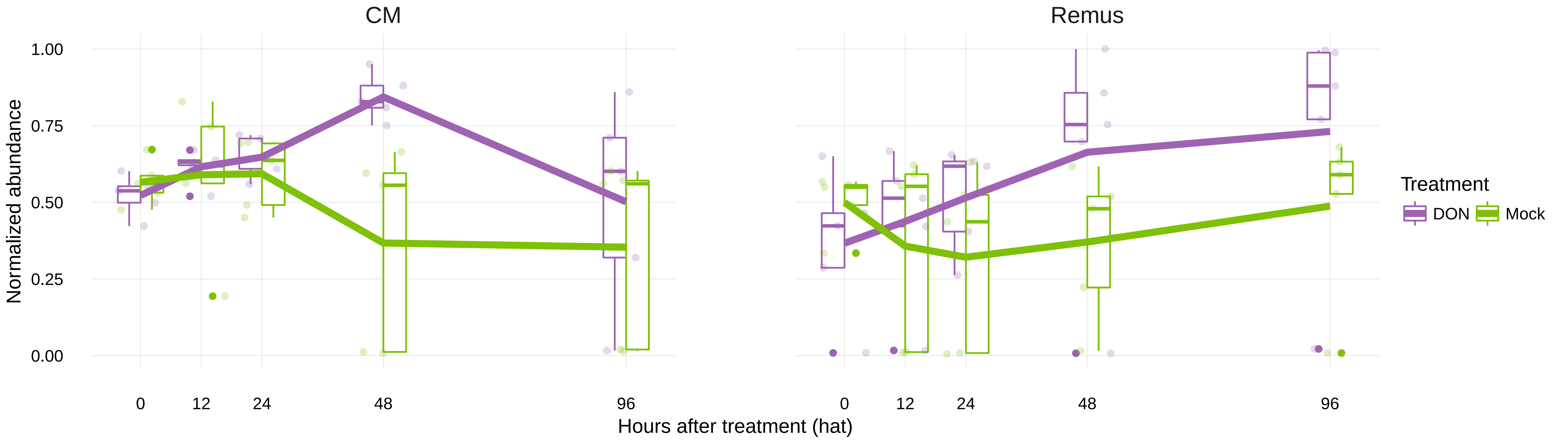

DON, Mock; all four genotypes

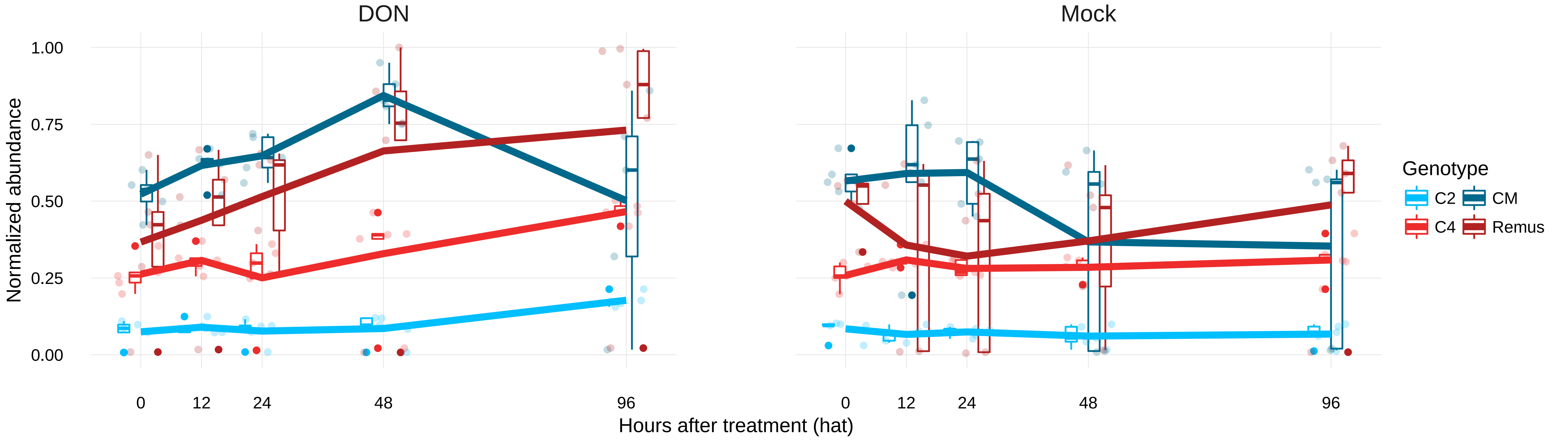

A.68

Annotated as Flavonoid or others (GlcF)
(3 database hits)

MZ	559.1784
RT	13.27 min
Normalization	Indirectly via surrogate in the KPX samples
Cluster	–
Cn total / Phe	26 /

C2, C4; different treatments

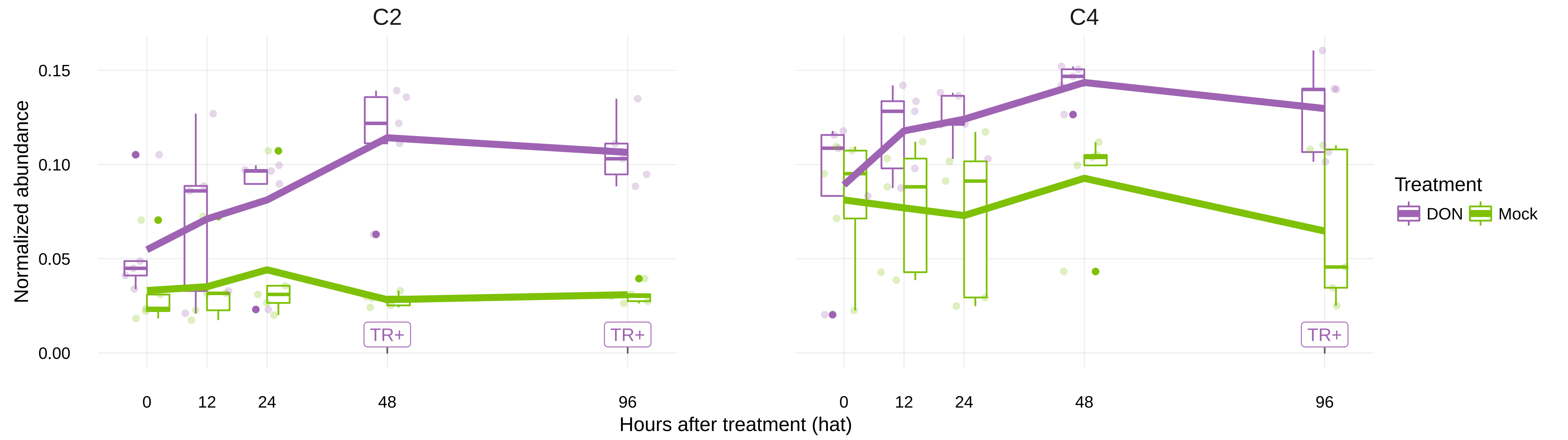

DON, Mock; different genotypes

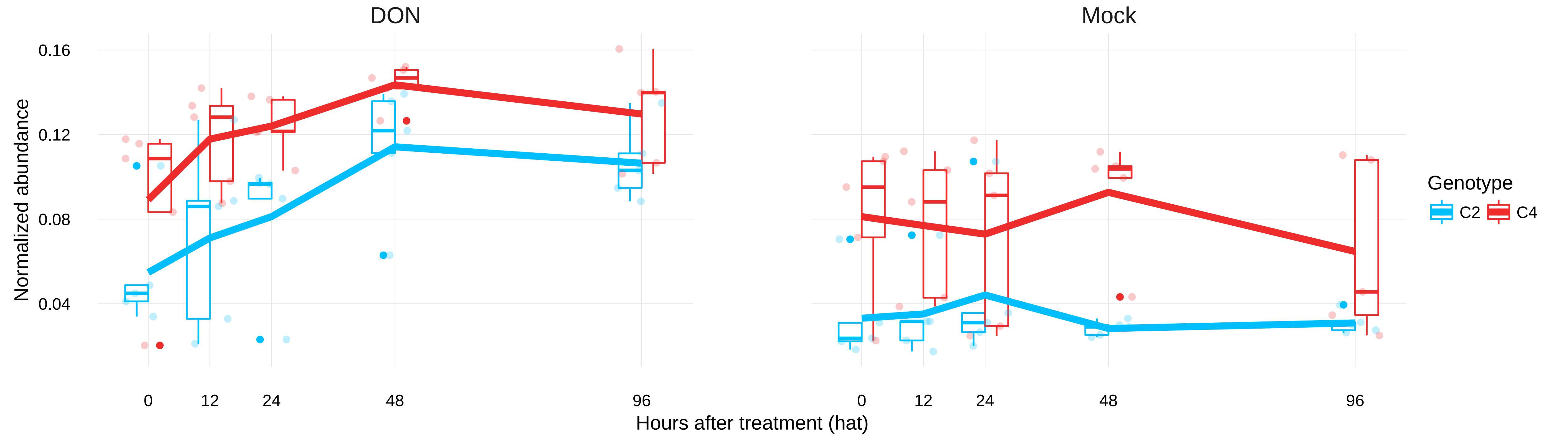

CM, Remus; different treatments

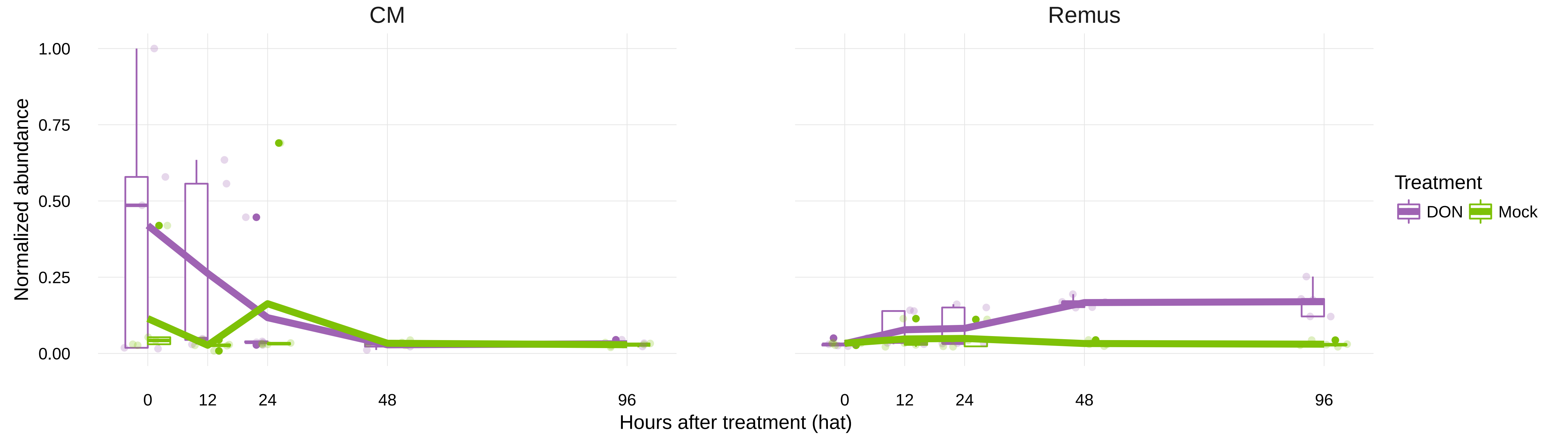

DON, Mock; all four genotypes

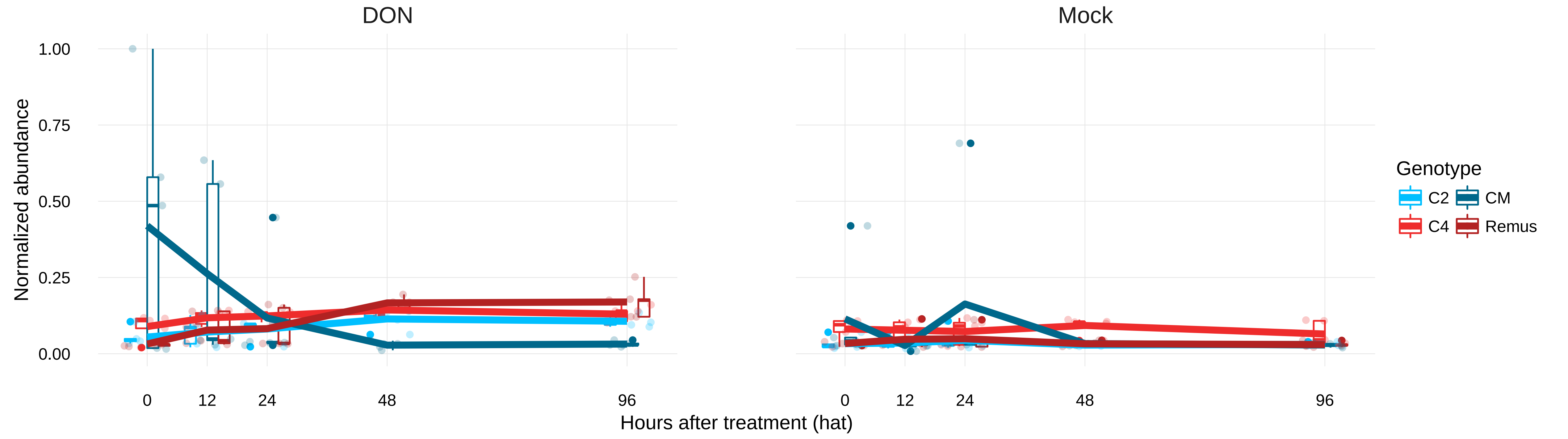

Trimethyltricetin

Identified metabolite (level 1)

MZ	345.097
RT	24.01 min
Normalization	Directly via KPX samples
Cluster	–
Cn total / Phe	18 /

C2, C4; different treatments

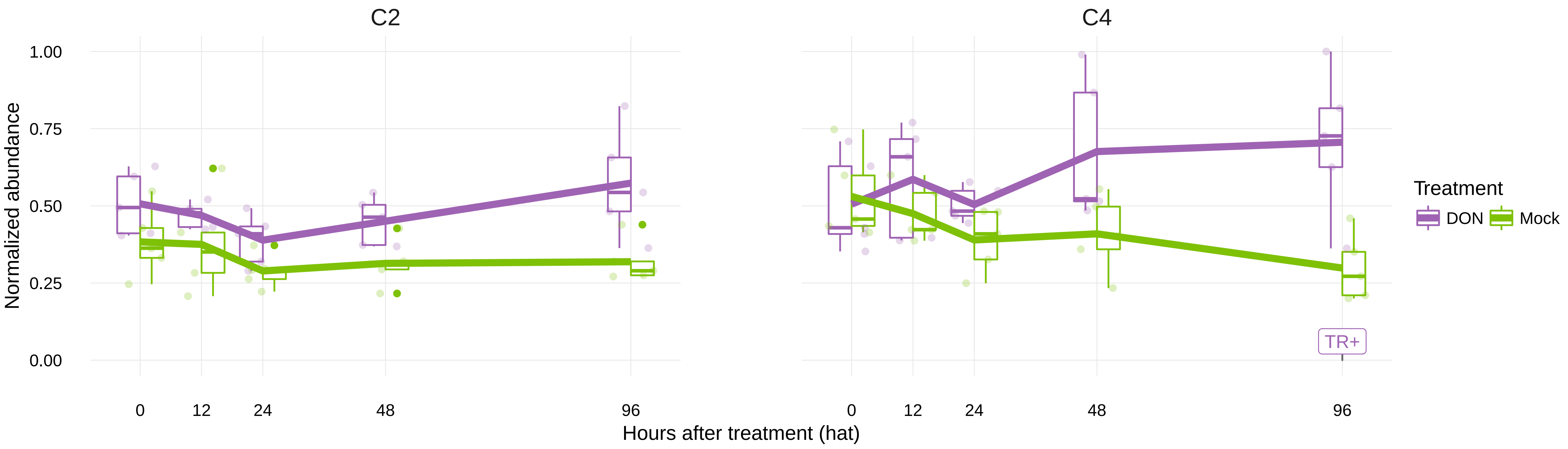

DON, Mock; different genotypes

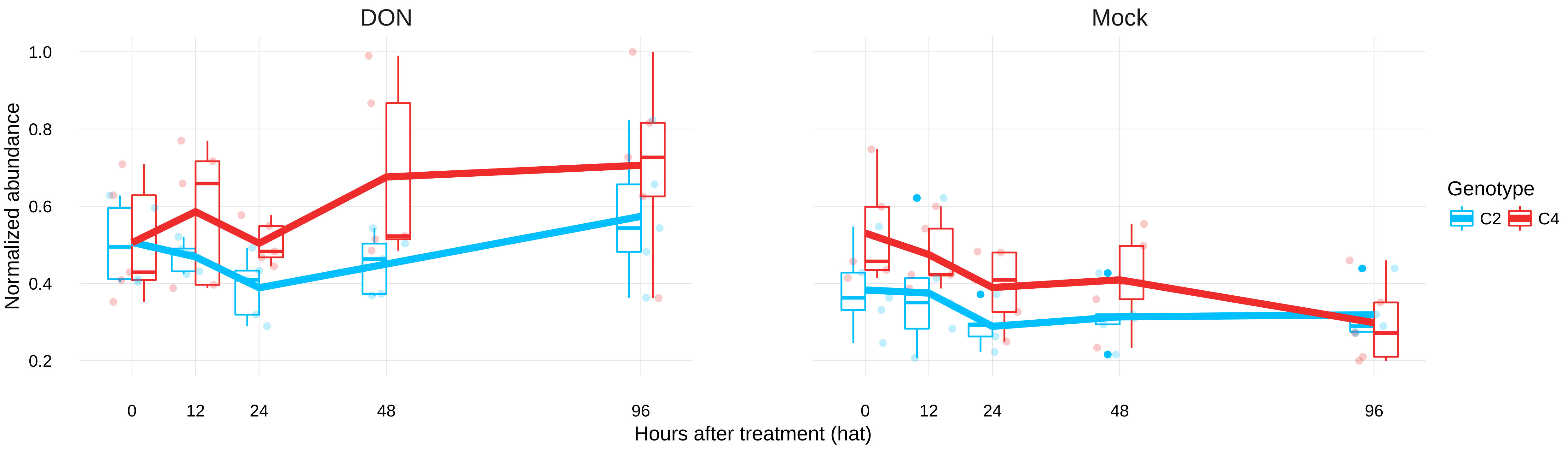

CM, Remus; different treatments

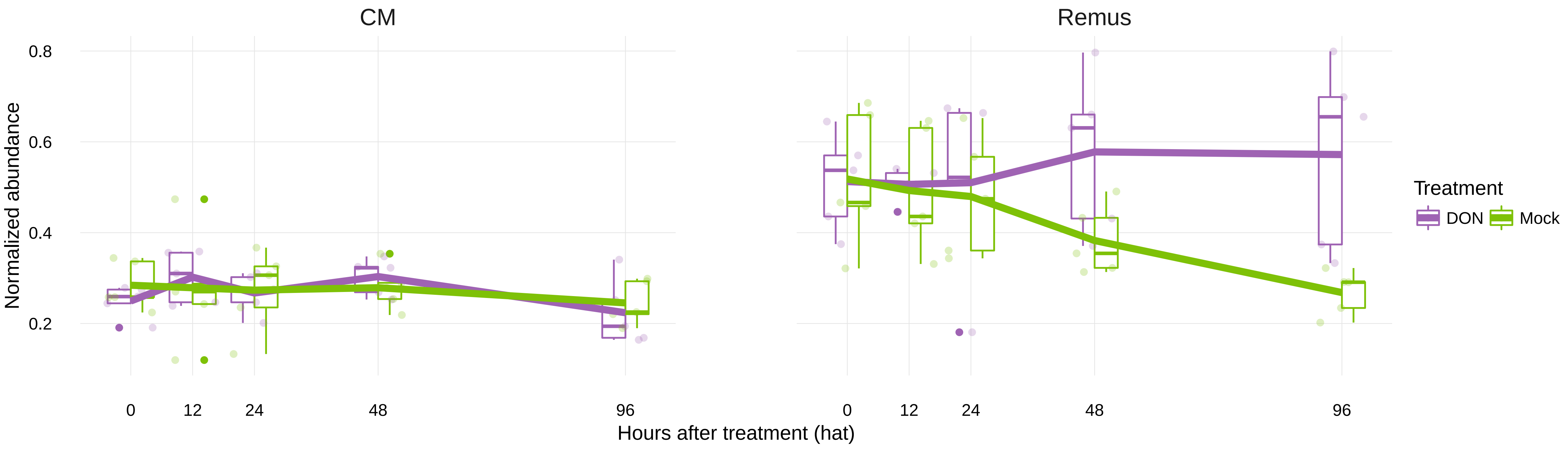

DON, Mock; all four genotypes

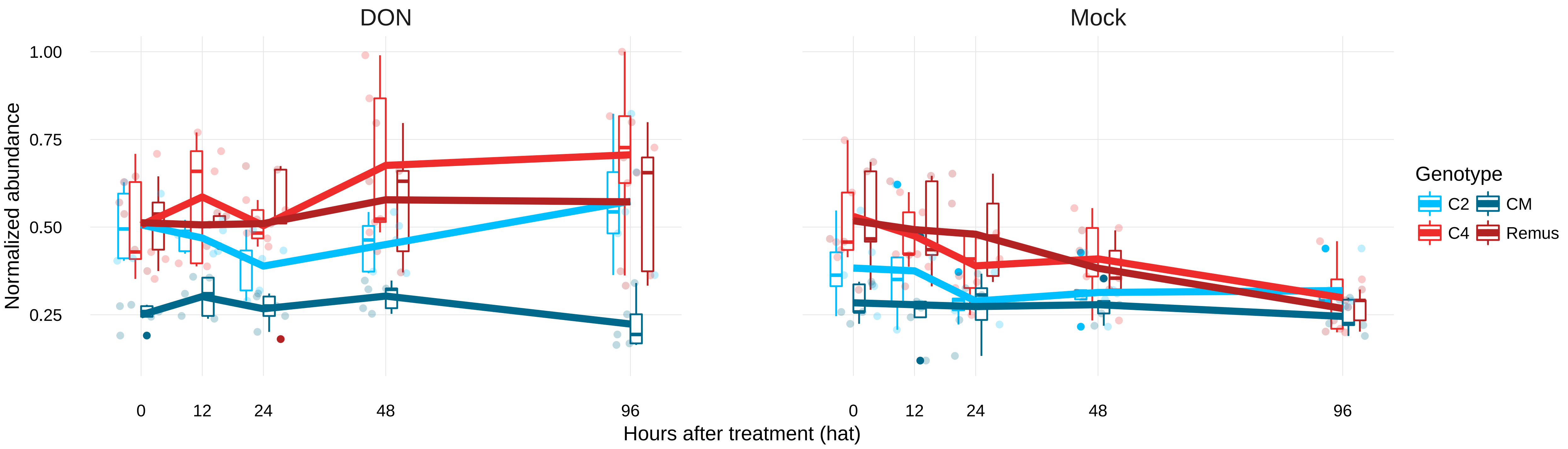

A.93

Annotated as Flavonoid (diGlcF)

(10 database hits)

MZ	640.2237
RT	15.8 min
Normalization	Directly via KPX samples
Cluster	–
Cn total / Phe	29 / 9

C2, C4; different treatments

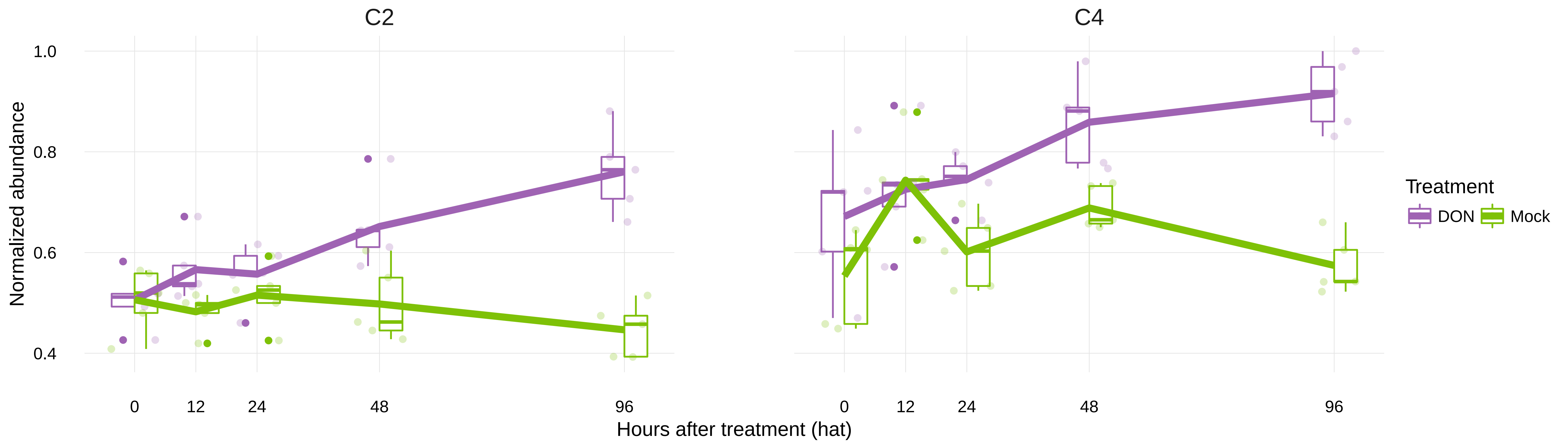

DON, Mock; different genotypes

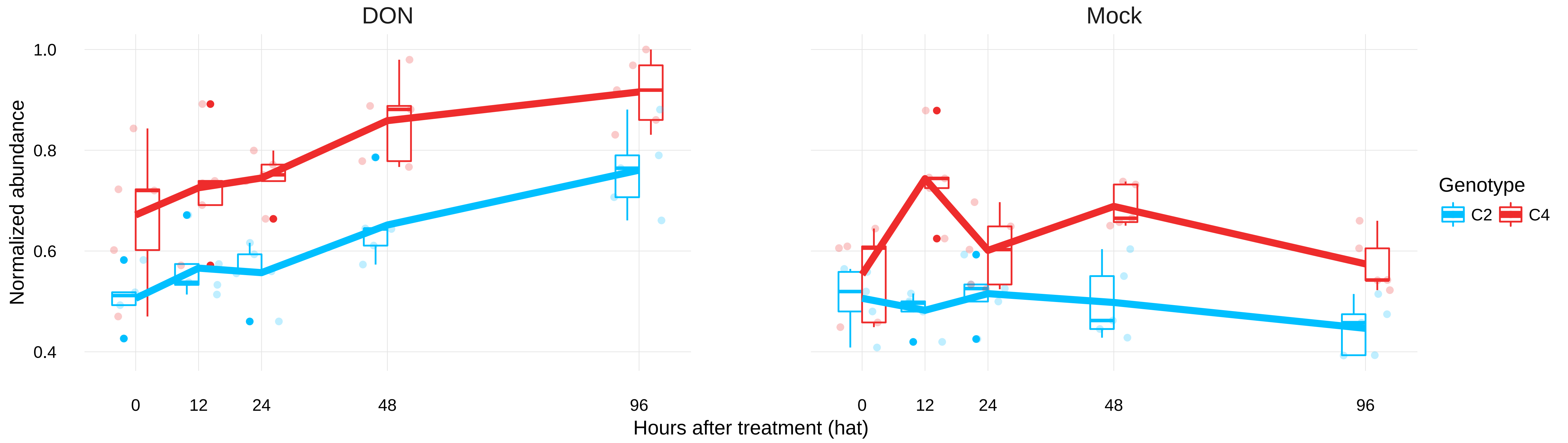

CM, Remus; different treatments

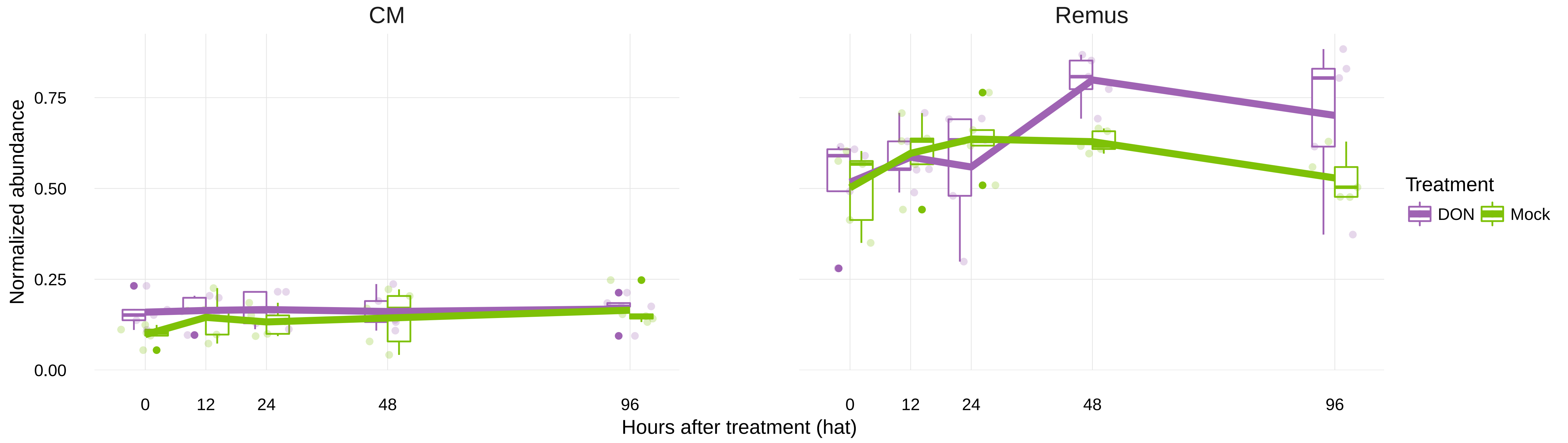

DON, Mock; all four genotypes

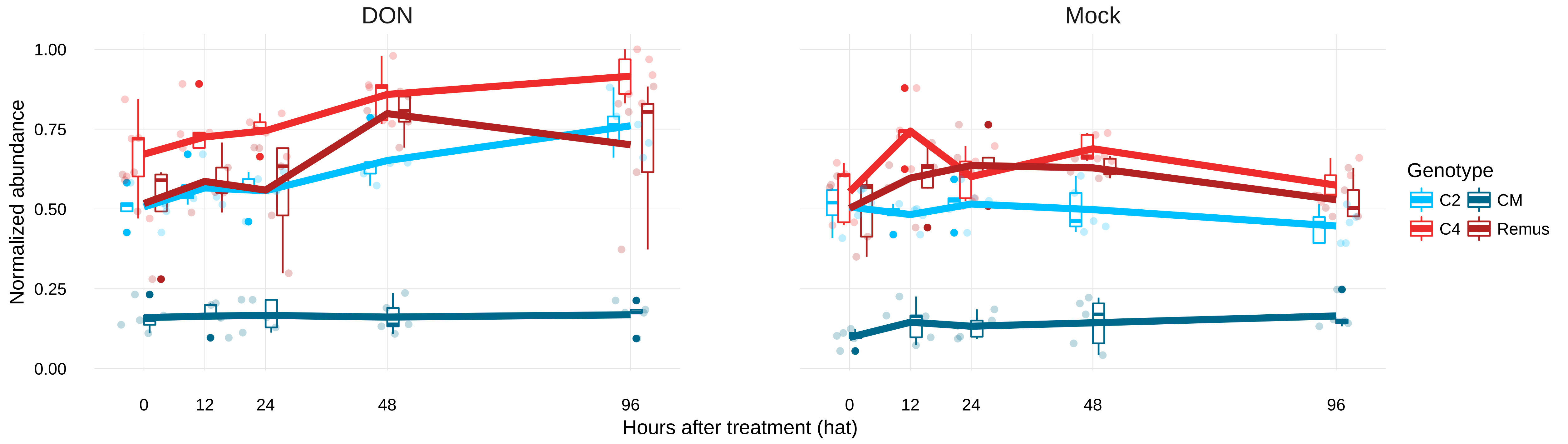

A.53

Annotated as HCA derivative

(1 database hit)

MZ	415.1238
RT	12.26 min
Normalization	Directly via KPX samples
Cluster	–
Cn total / Phe	18 / 9

C2, C4; different treatments

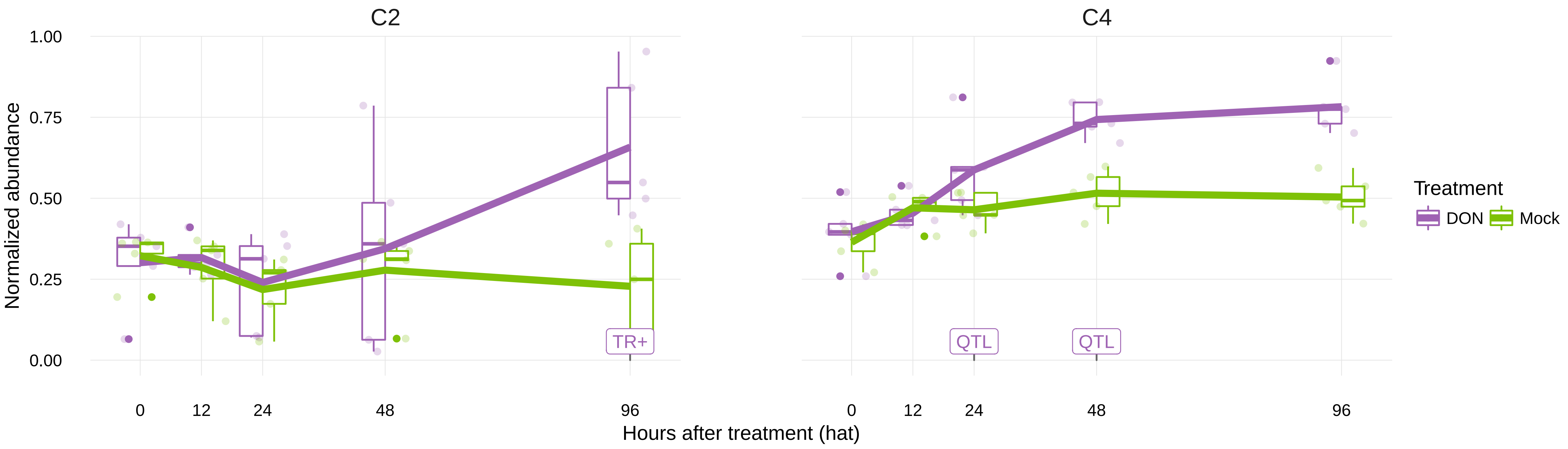

DON, Mock; different genotypes

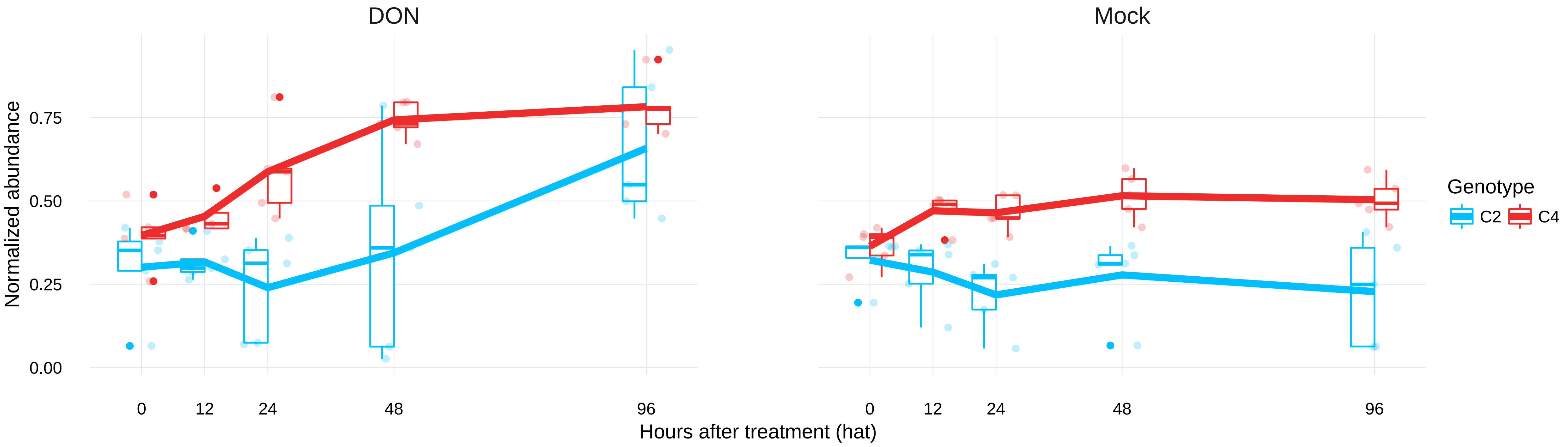

CM, Remus; different treatments

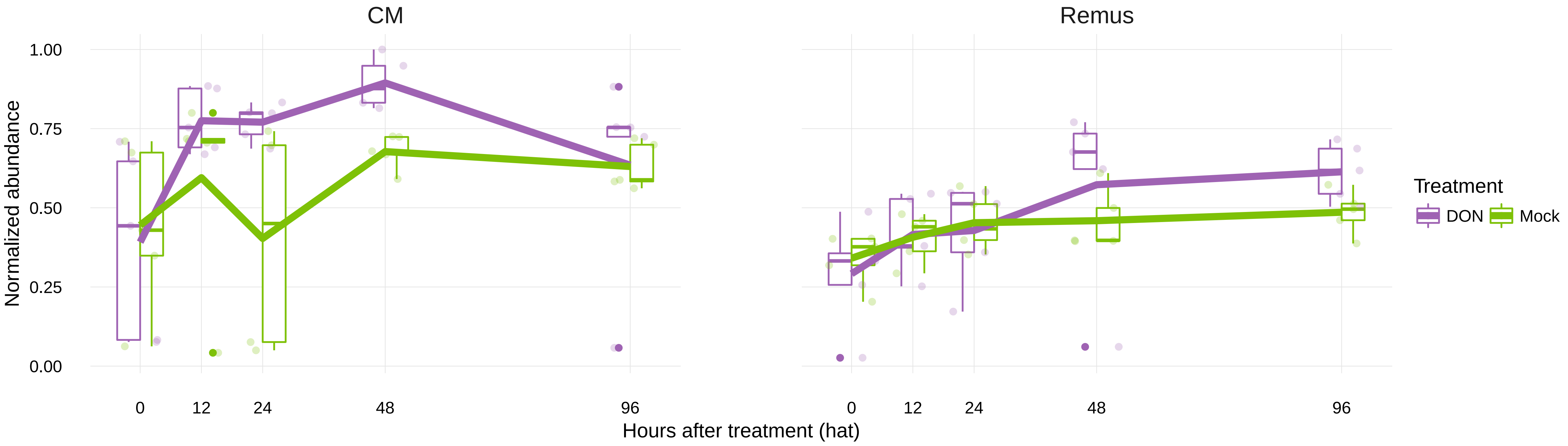

DON, Mock; all four genotypes

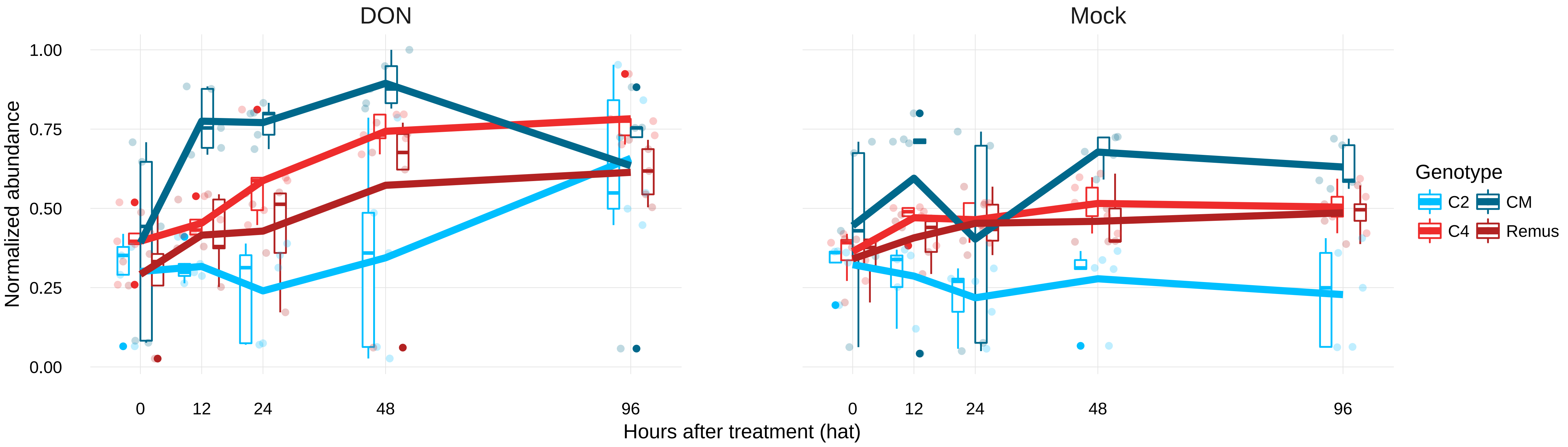

A.77

Annotated as HCA derivative
(1 database hit)

MZ	251.0915
RT	14.17 min
Normalization	Directly via KPX samples
Cluster	–
Cn total / Phe	13 / 9

C2, C4; different treatments

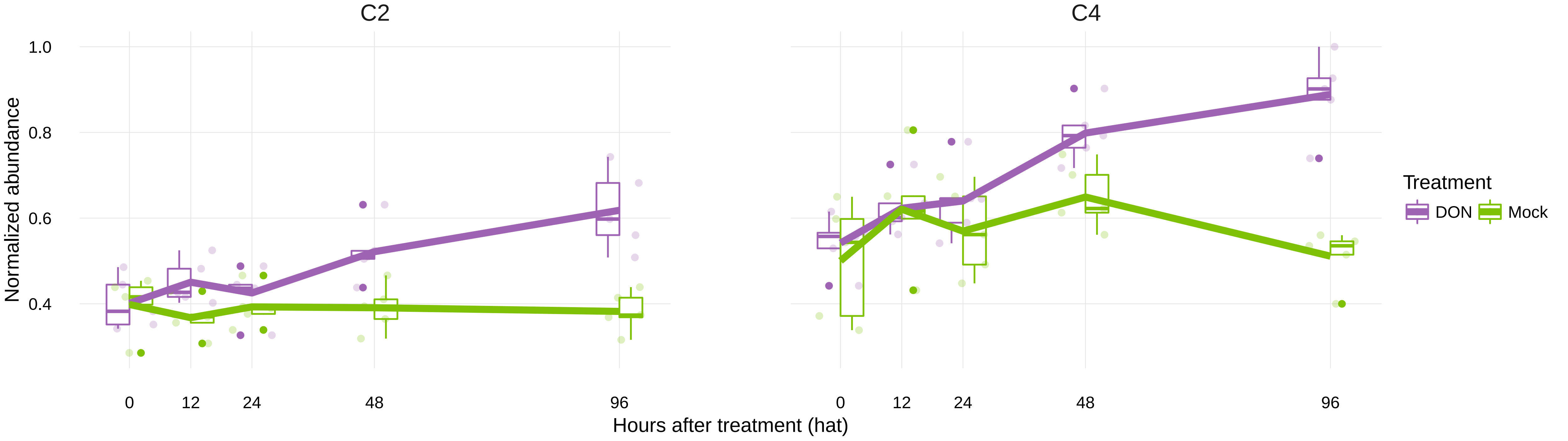

DON, Mock; different genotypes

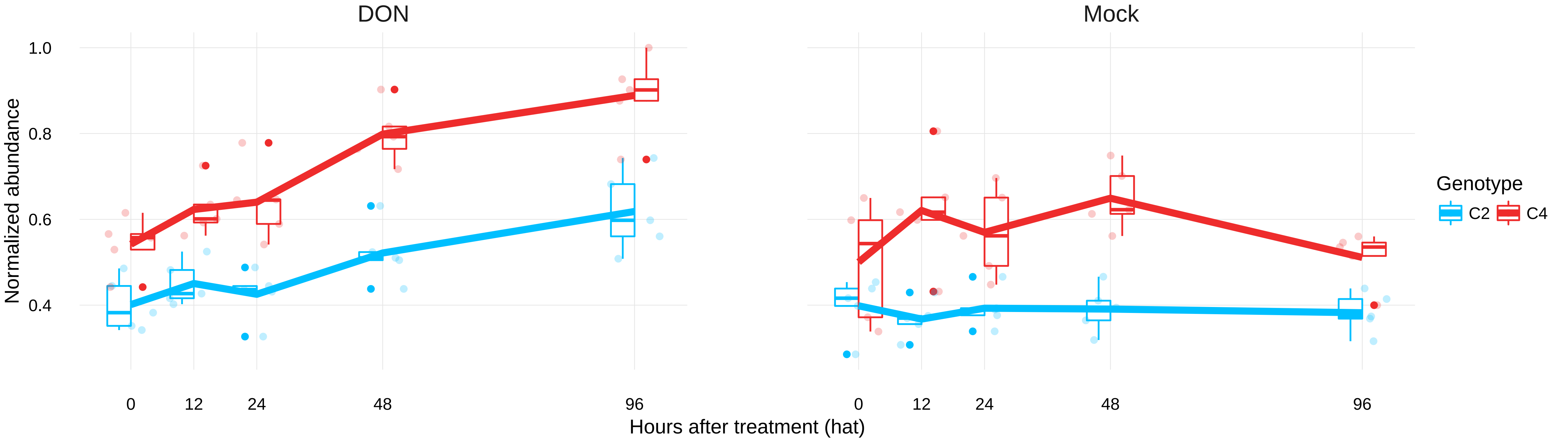

CM, Remus; different treatments

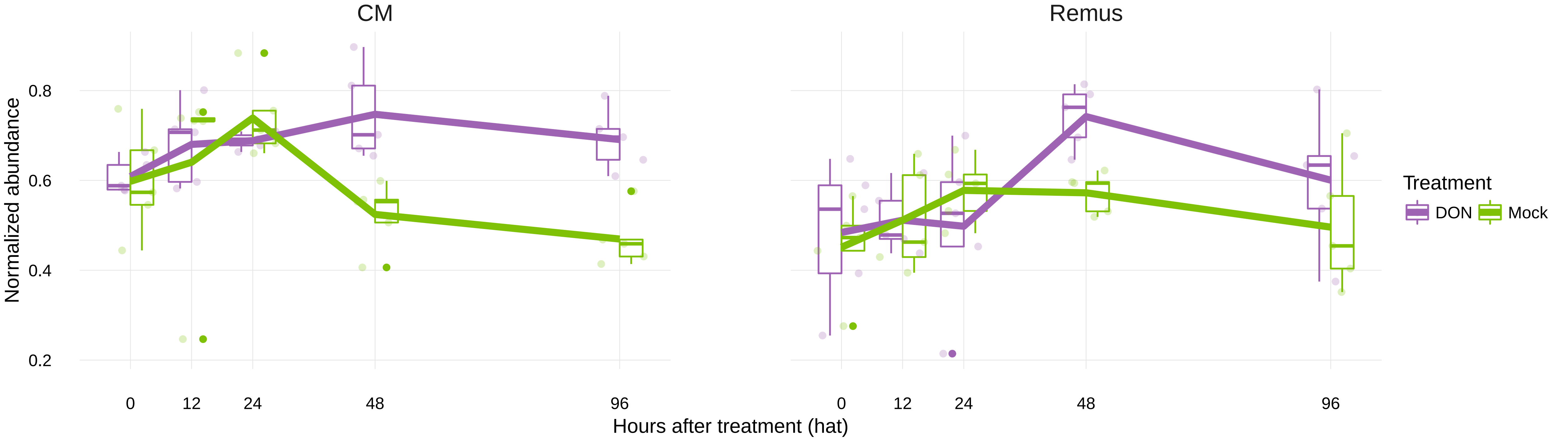

DON, Mock; all four genotypes

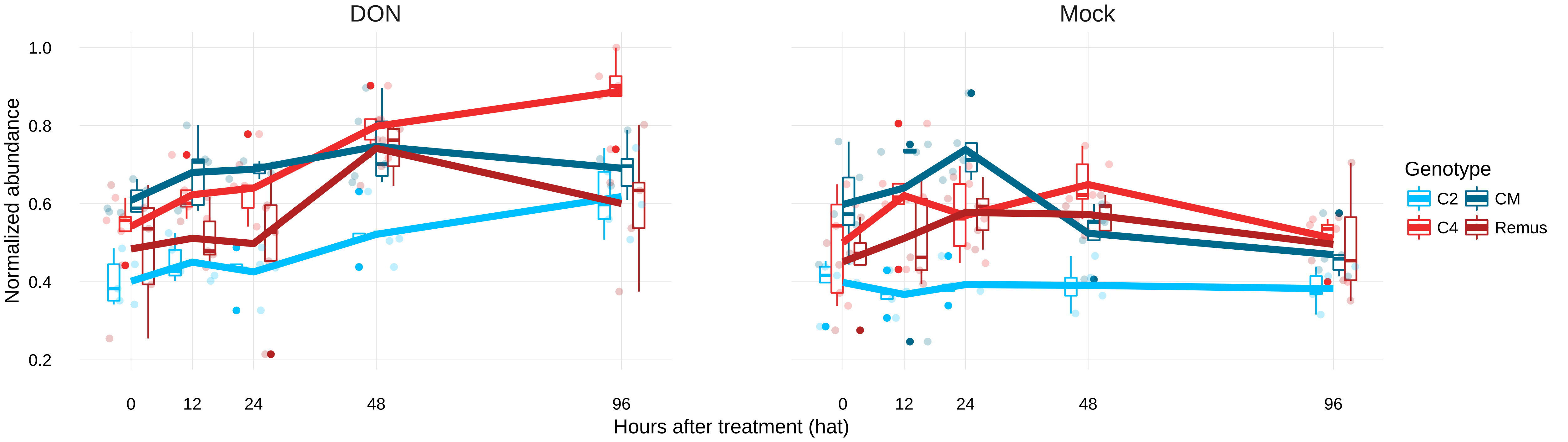

A.101

Annotated as Lignan
(1 database hit)

MZ	601.1893
RT	16.79 min
Normalization	Directly via KPX samples
Cluster	–
Cn total / Phe	28 /

C2, C4; different treatments

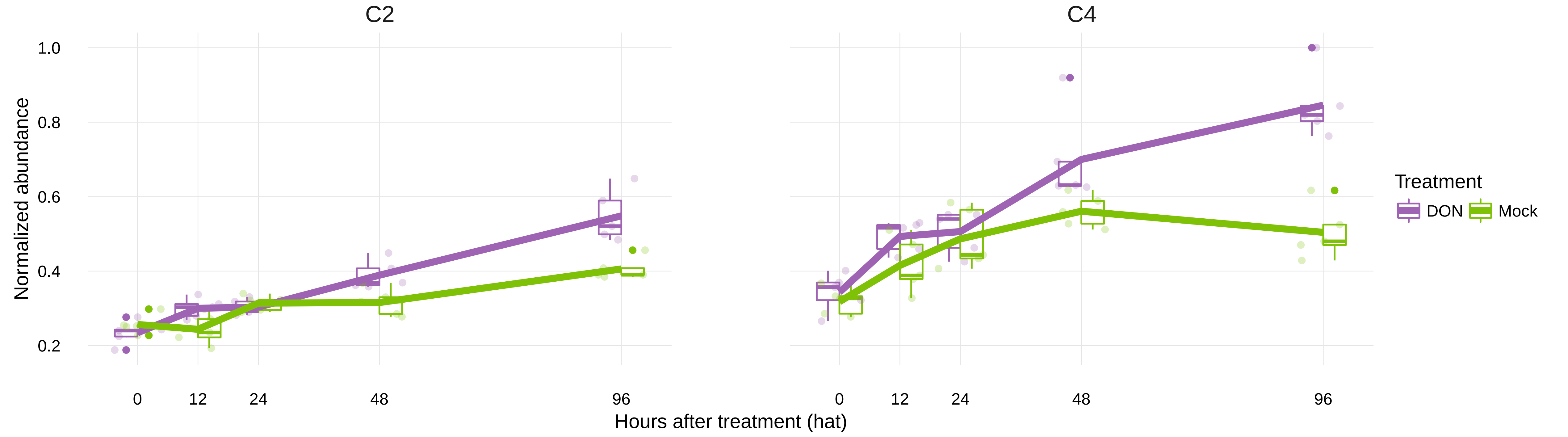

DON, Mock; different genotypes

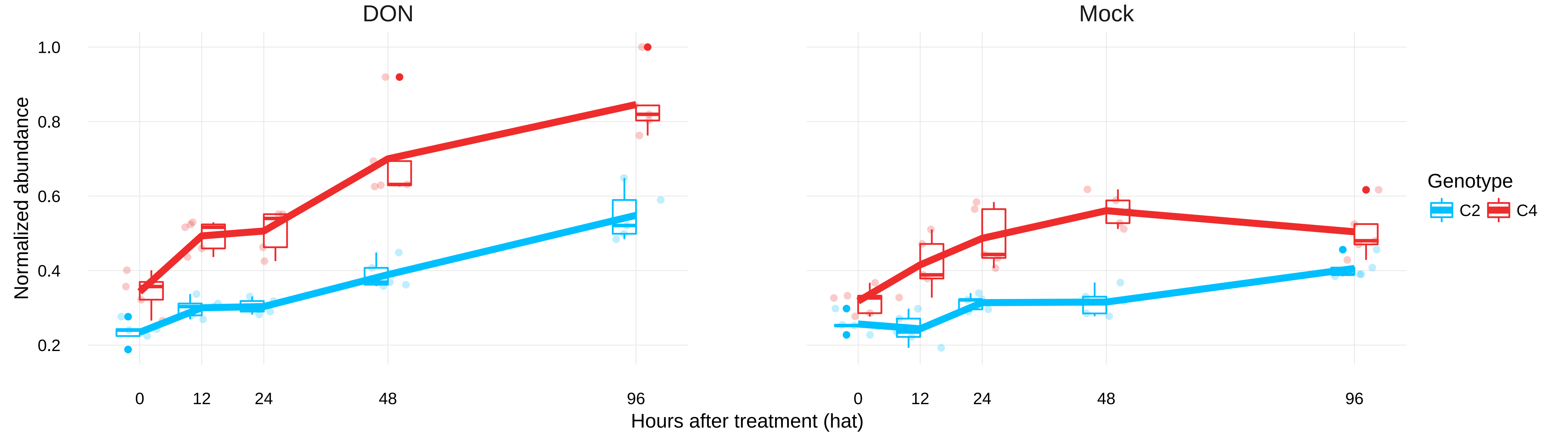

CM, Remus; different treatments

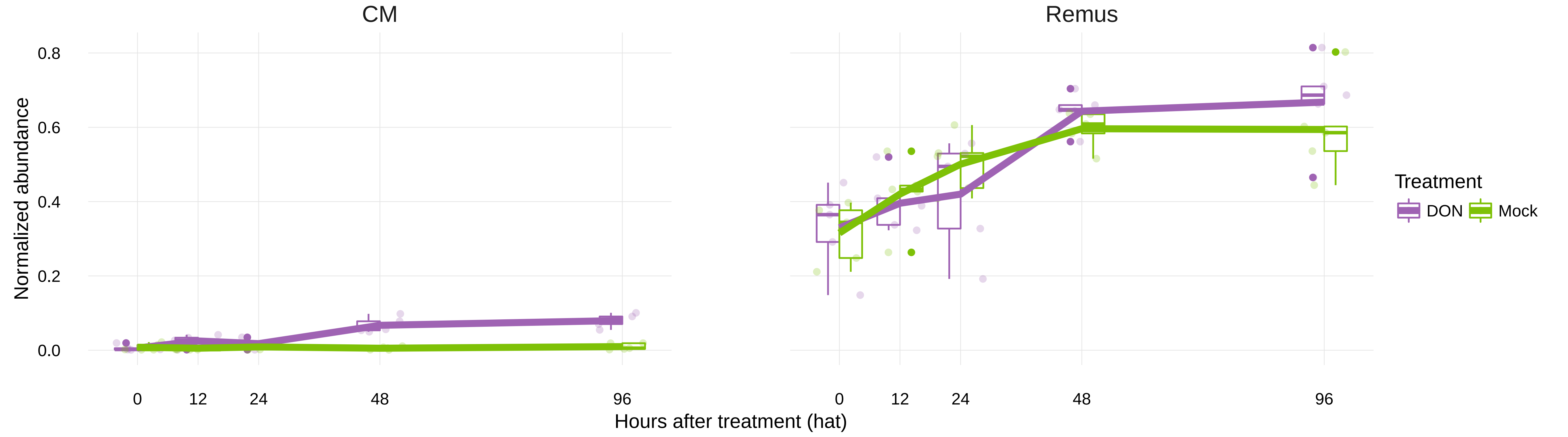

DON, Mock; all four genotypes

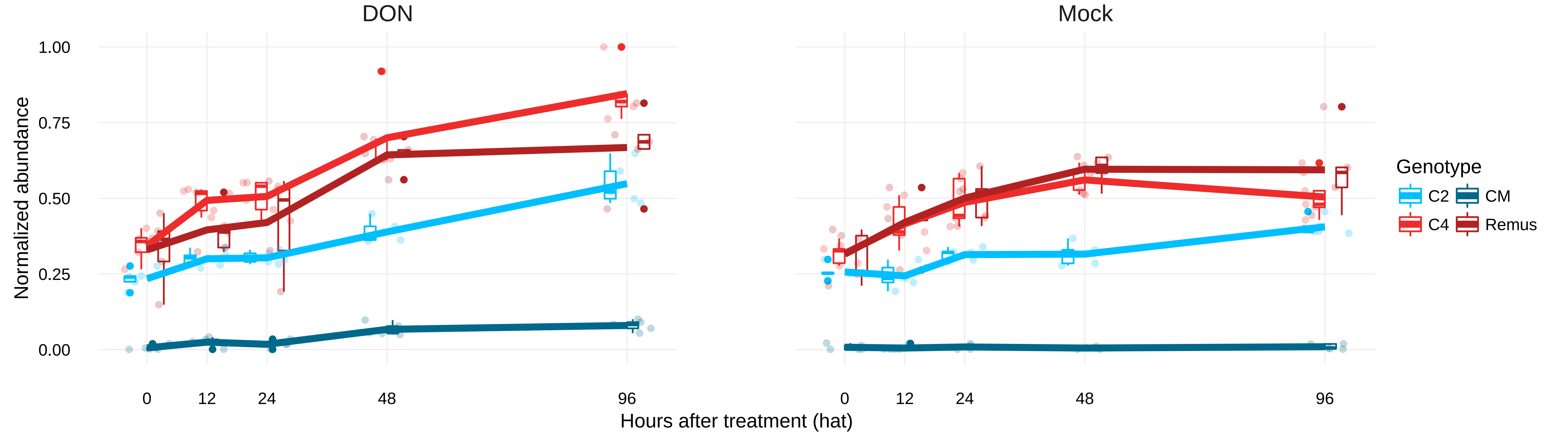

A.91

Annotated as others (Iridoid)

(1 database hit)

MZ	531.1474
RT	15.67 min
Normalization	Directly via KPX samples
Cluster	–
Cn total / Phe	24 /

C2, C4; different treatments

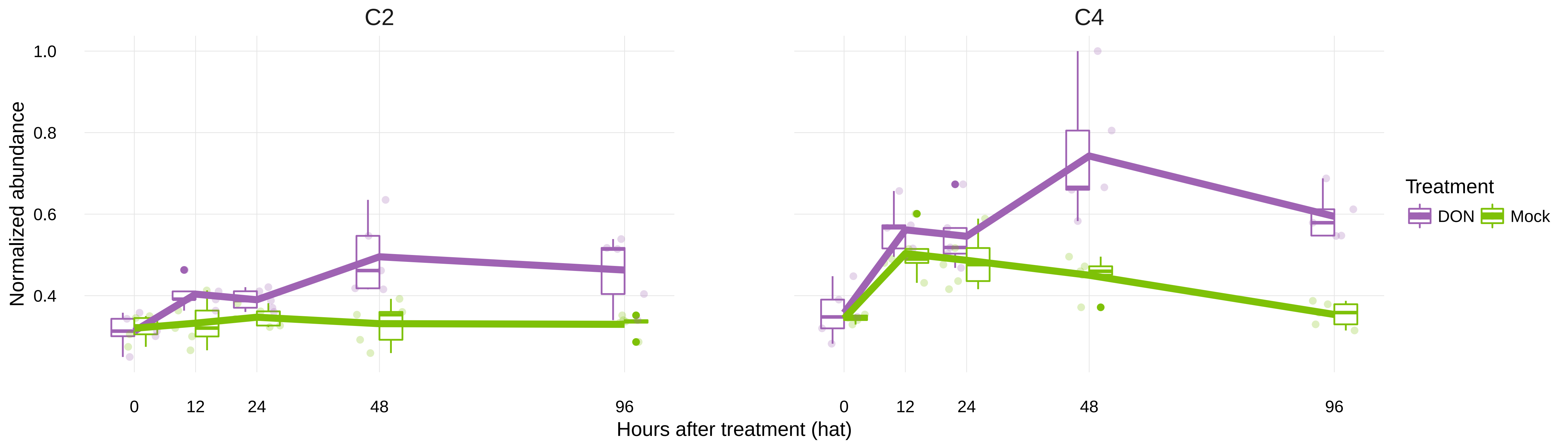

DON, Mock; different genotypes

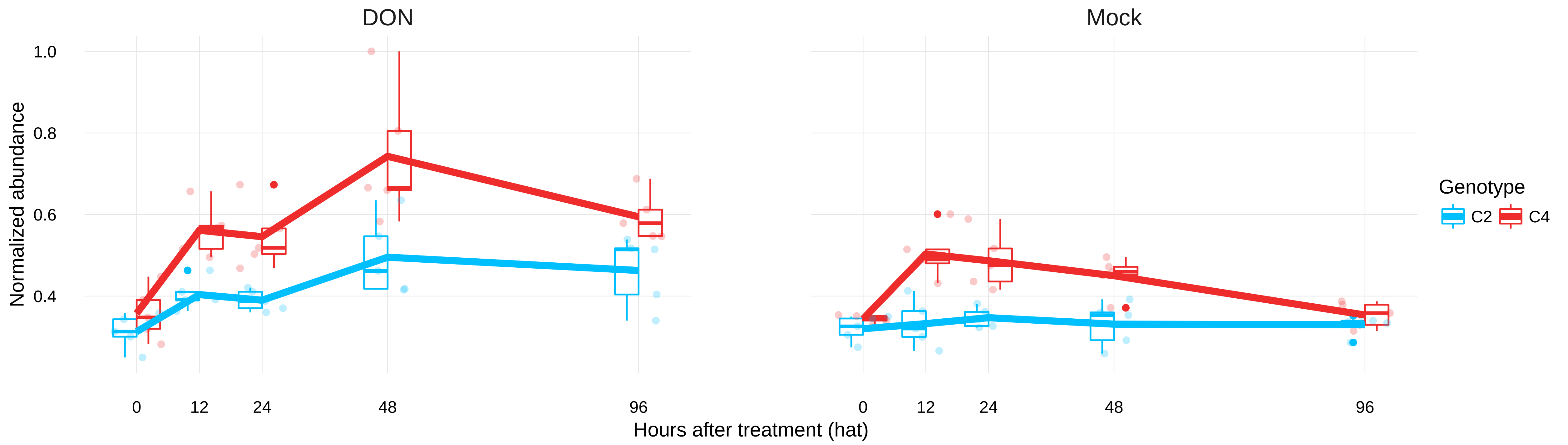

CM, Remus; different treatments

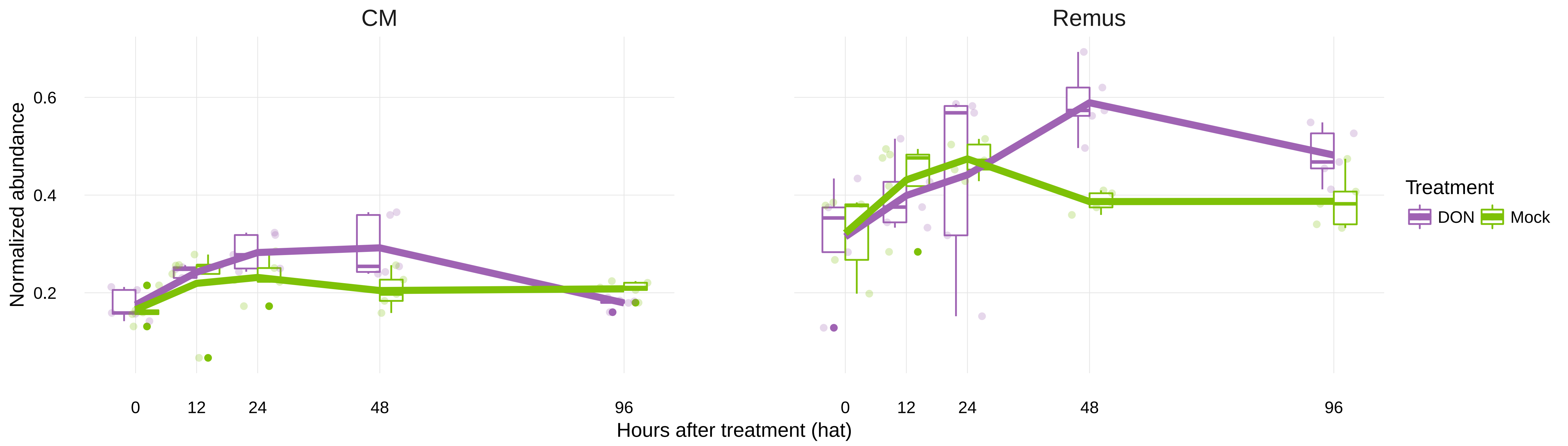

DON, Mock; all four genotypes

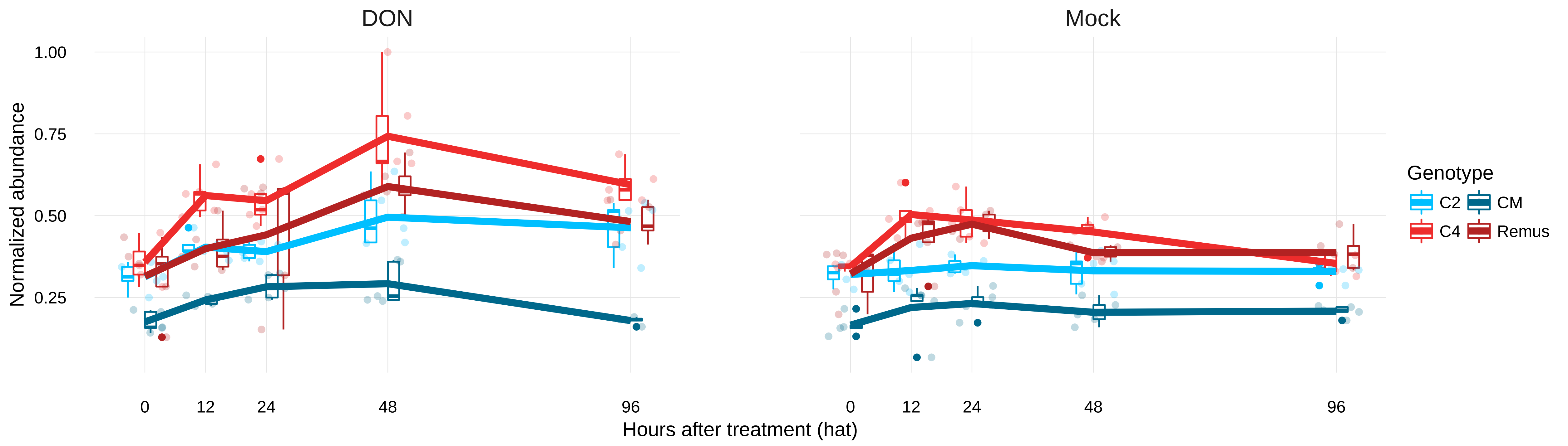

A.129

Annotated as Flavonoid (GlcF)

(17 database hits)

MZ	419.0974
RT	20.88 min
Normalization	Directly via KPX samples
Cluster	–
Cn total / Phe	20 /

C2, C4; different treatments

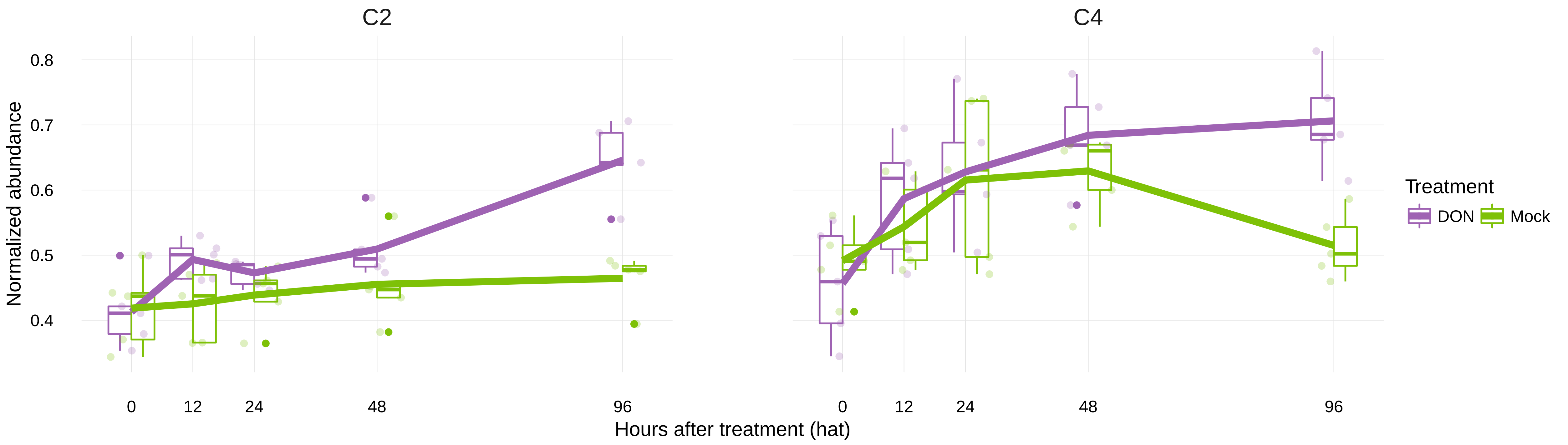

DON, Mock; different genotypes

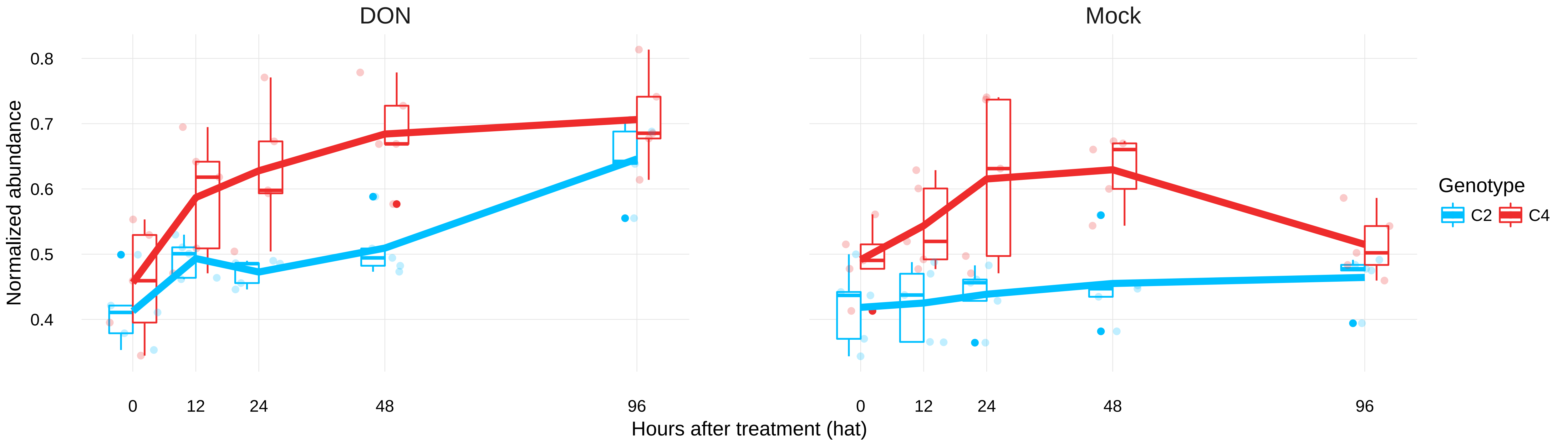

CM, Remus; different treatments

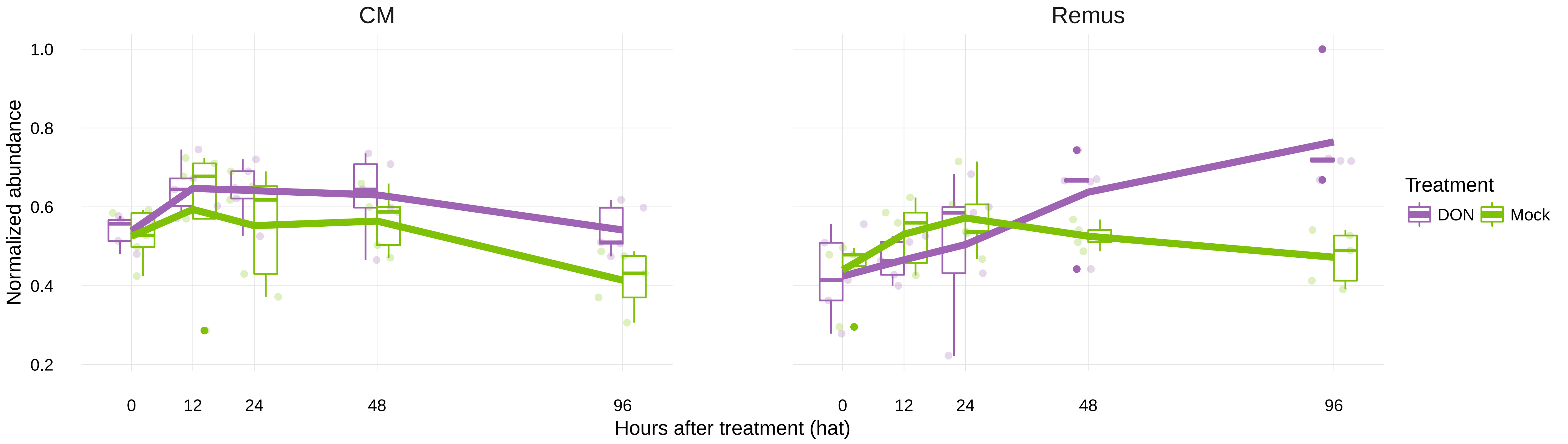

DON, Mock; all four genotypes

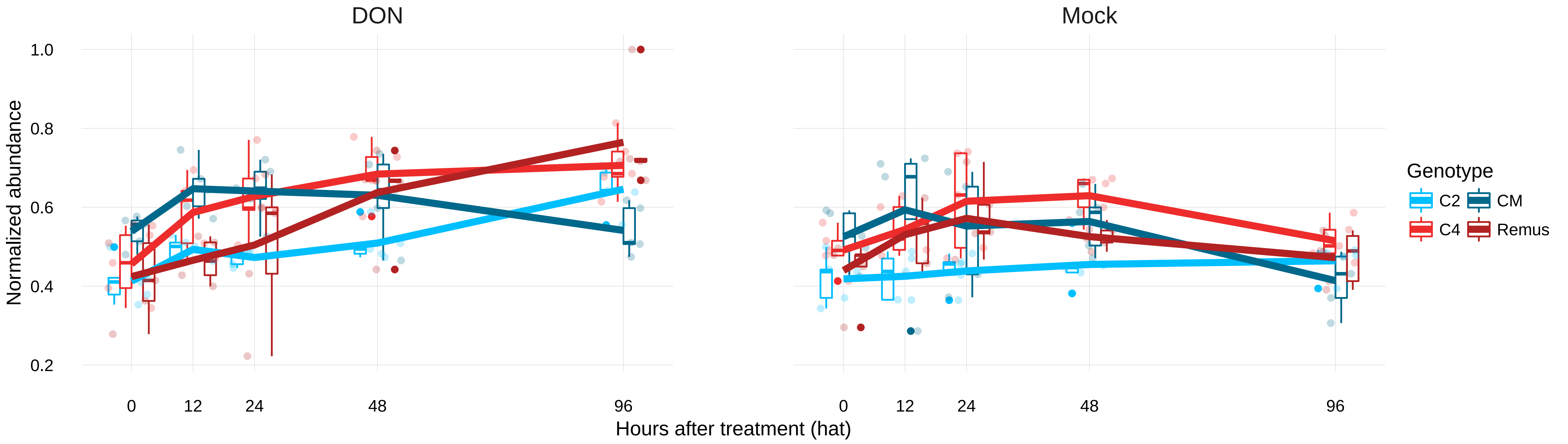

A.127

Annotated as Flavonoid
(74 database hits)

MZ	331.0813
RT	20.69 min
Normalization	Directly via KPX samples
Cluster	–
Cn total / Phe	17 /

C2, C4; different treatments

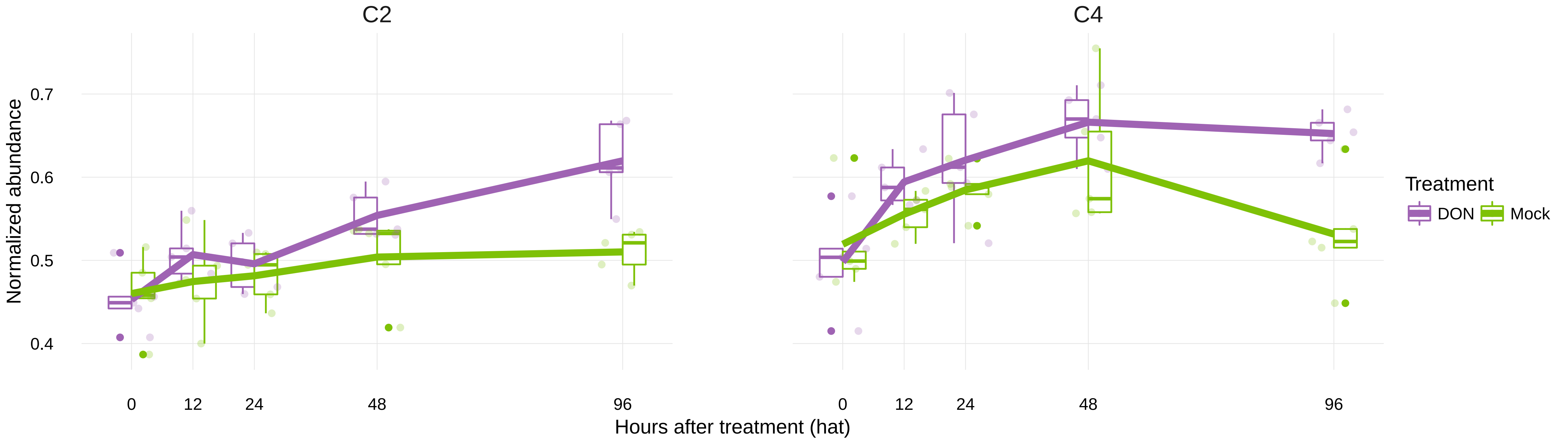

DON, Mock; different genotypes

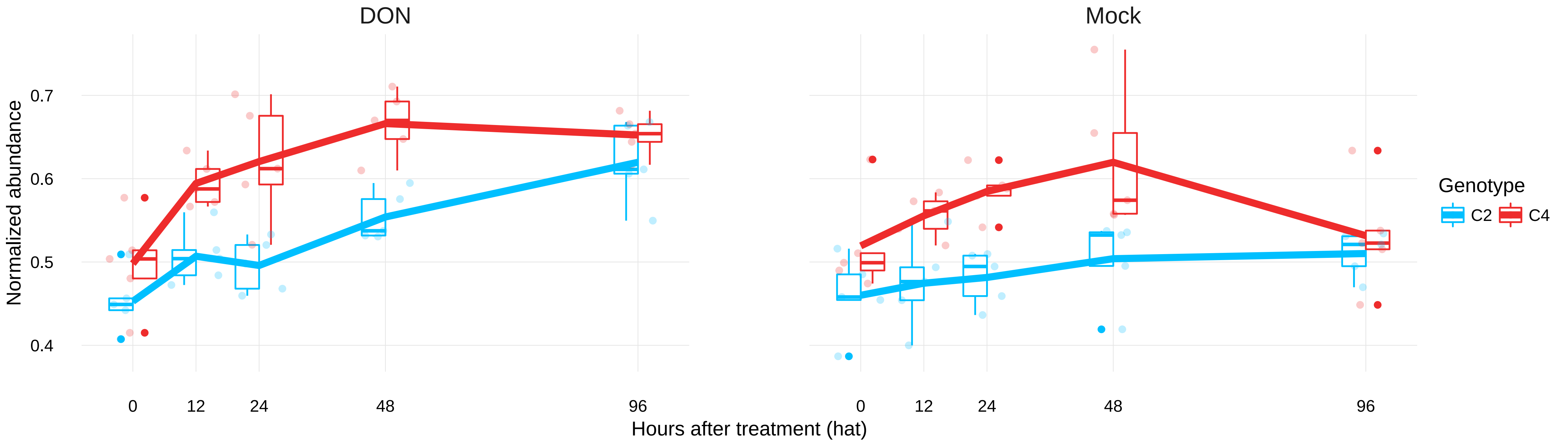

CM, Remus; different treatments

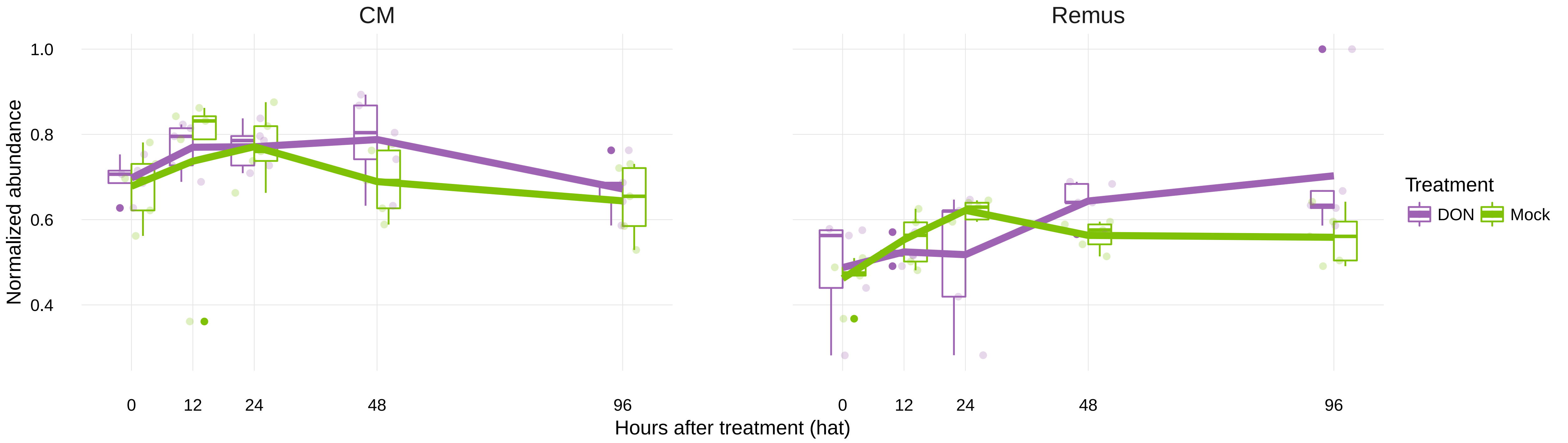

DON, Mock; all four genotypes

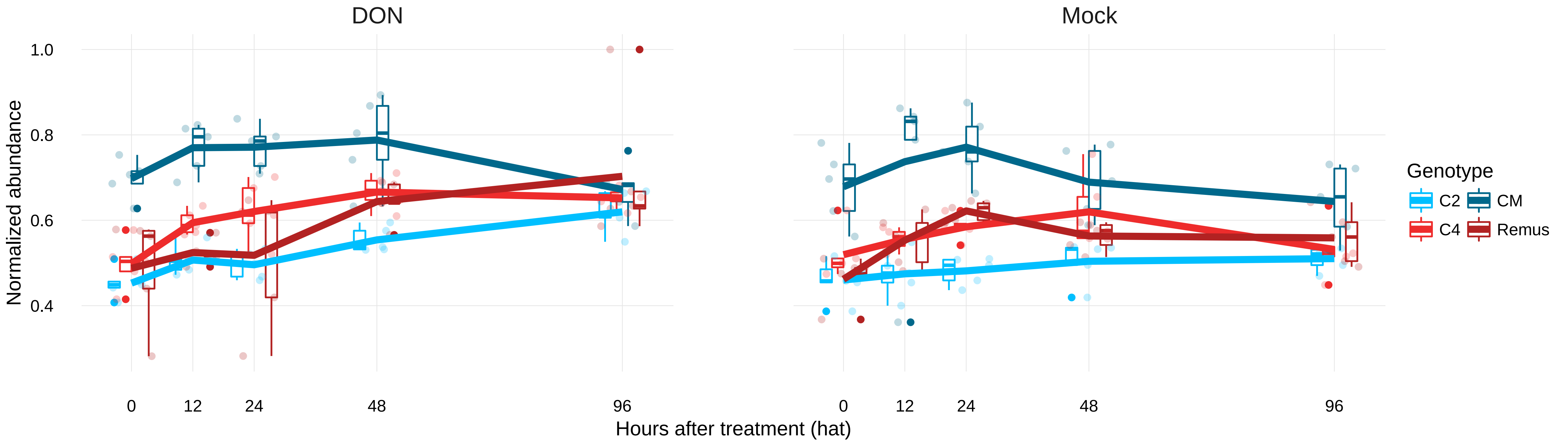

A.98

Annotated as Flavonoid (diGlcF)

(17 database hits)

MZ	639.1923
RT	16.48 min
Normalization	Directly via KPX samples
Cluster	–
Cn total / Phe	29 /

C2, C4; different treatments

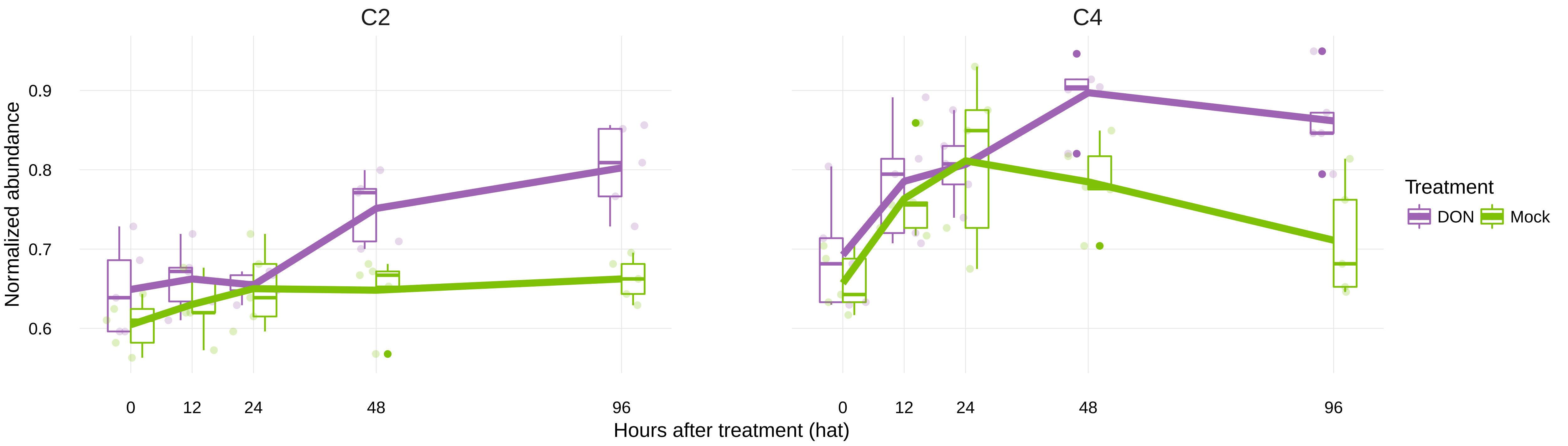

DON, Mock; different genotypes

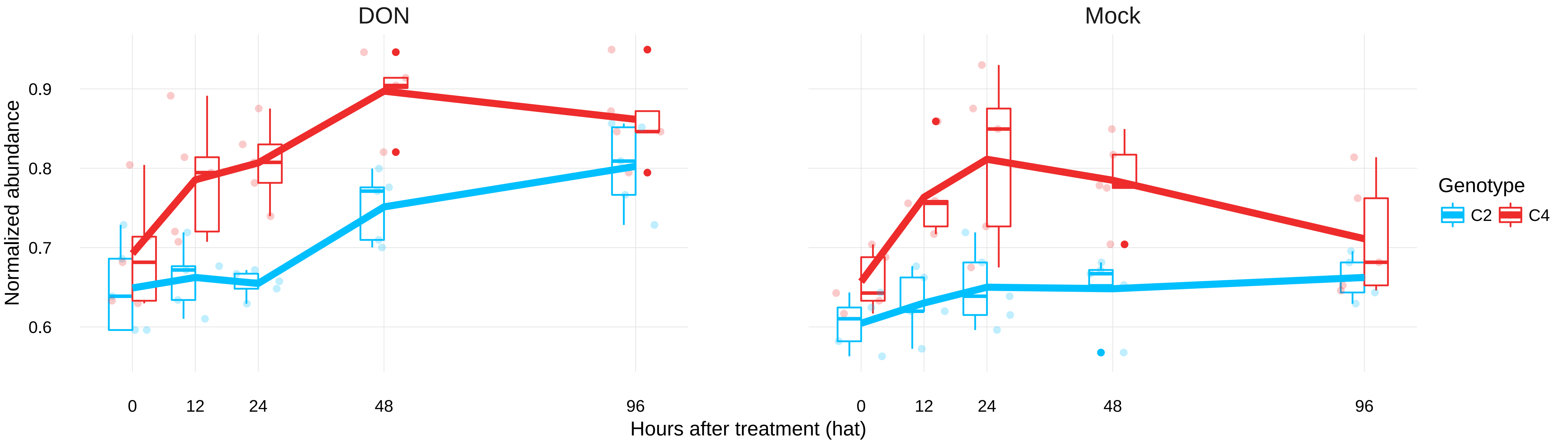

CM, Remus; different treatments

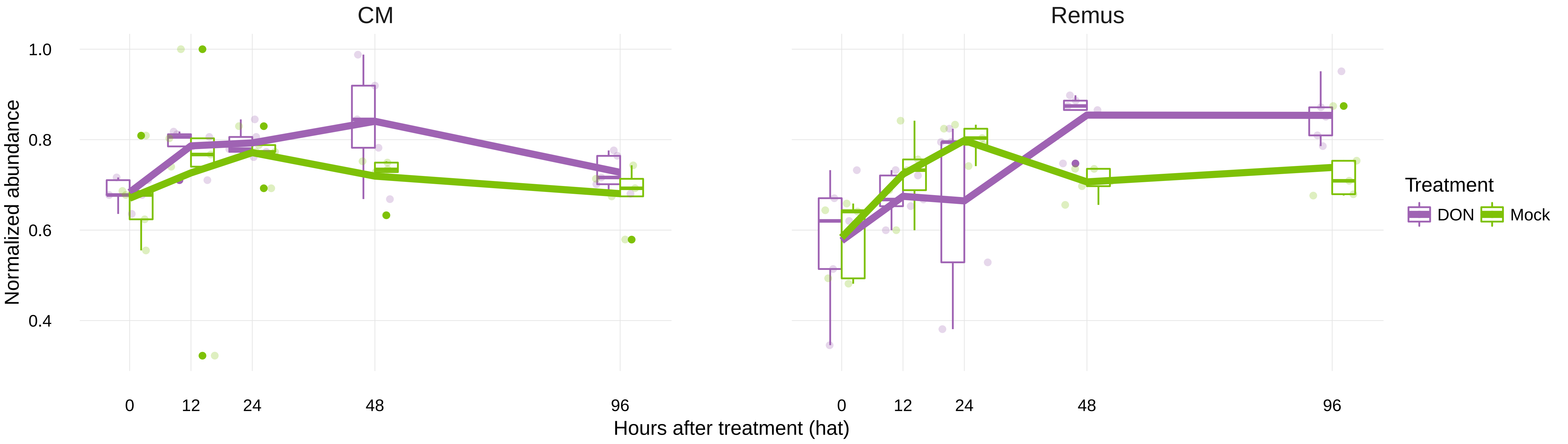

DON, Mock; all four genotypes

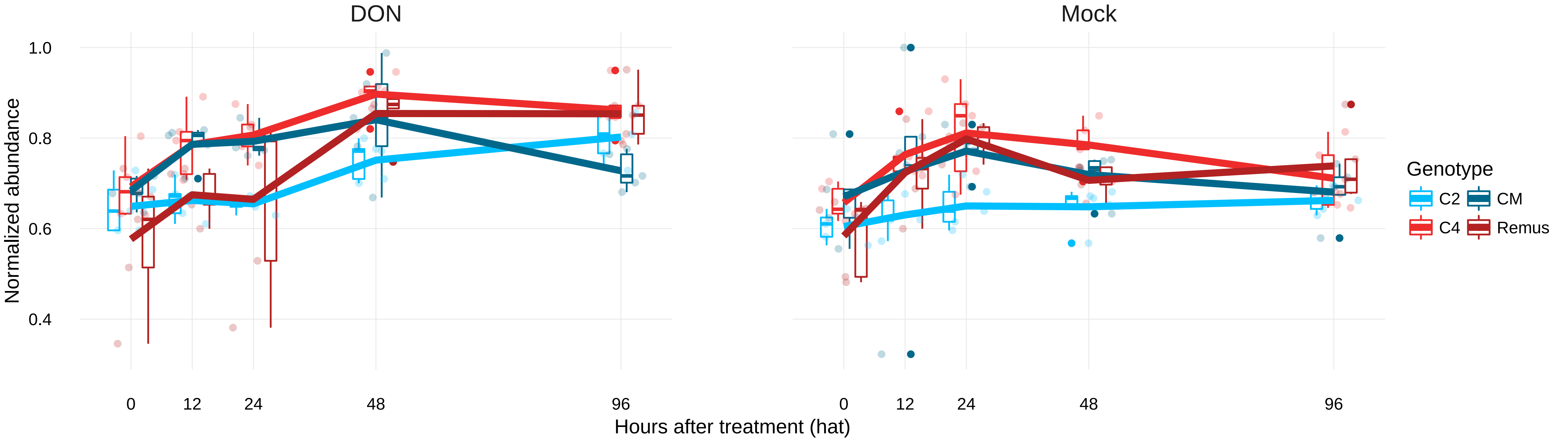

A.97

Annotated as Flavonoid (GlcF)

(51 database hits)

MZ	493.1342
RT	16.47 min
Normalization	Directly via KPX samples
Cluster	–
Cn total / Phe	23 /

C2, C4; different treatments

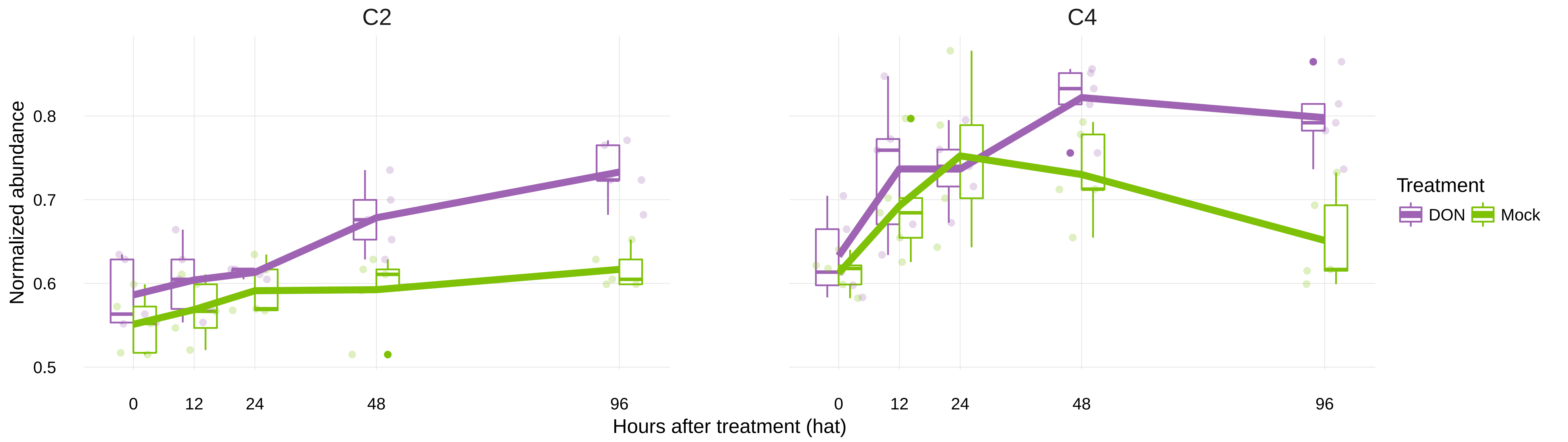

DON, Mock; different genotypes

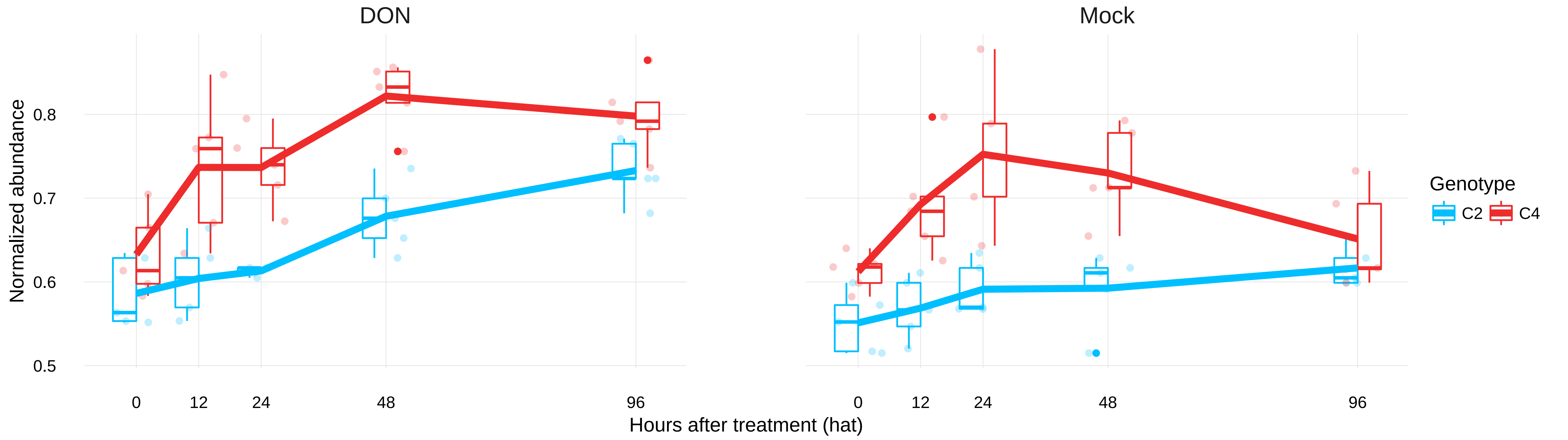

CM, Remus; different treatments

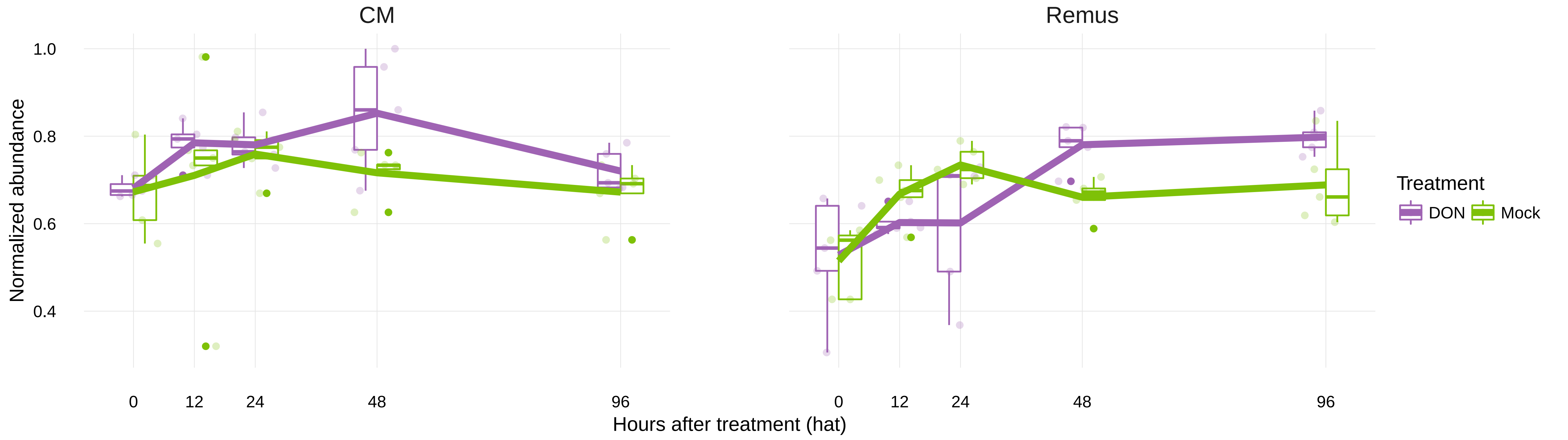

DON, Mock; all four genotypes

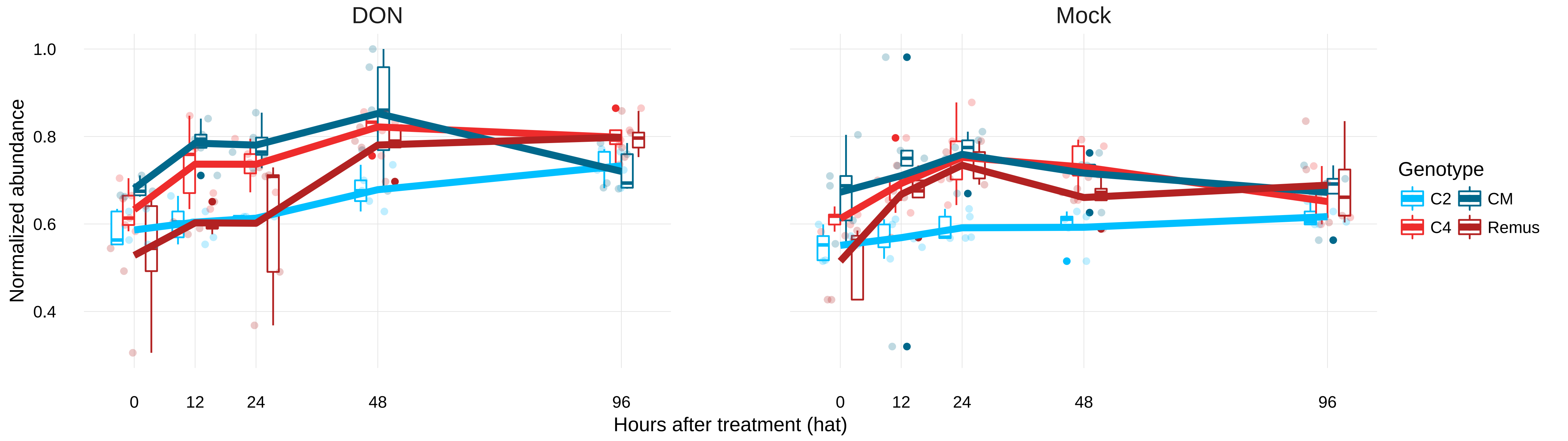

A.67

Annotated as HCA derivative
(1 database hit)

MZ	411.1287
RT	13.18 min
Normalization	Indirectly via surrogate in the KPX samples
Cluster	–
Cn total / Phe	19 / 9

C2, C4; different treatments

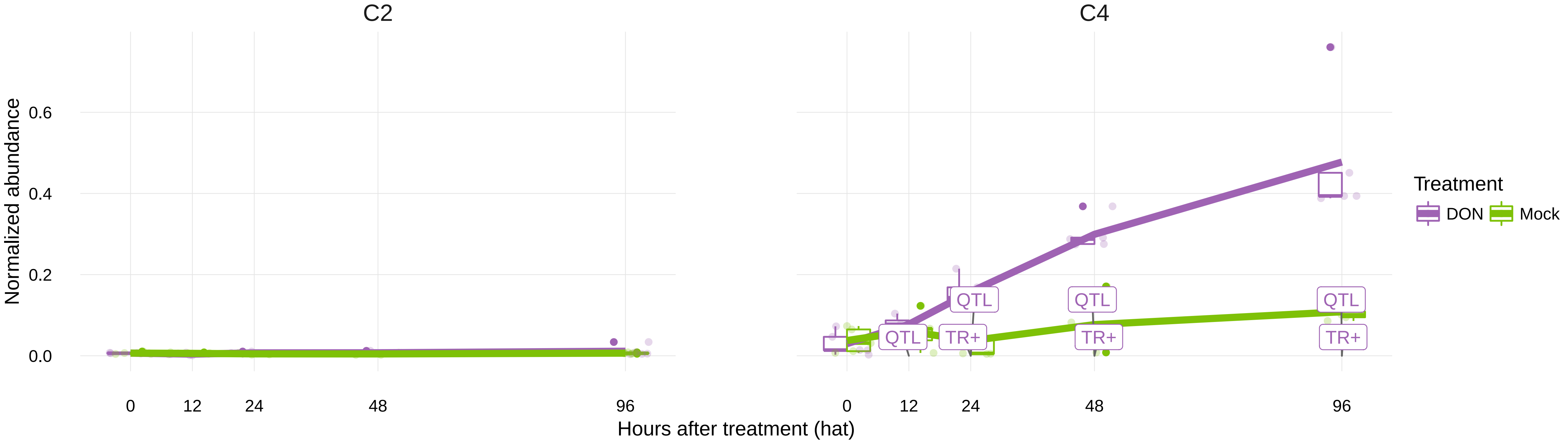

DON, Mock; different genotypes

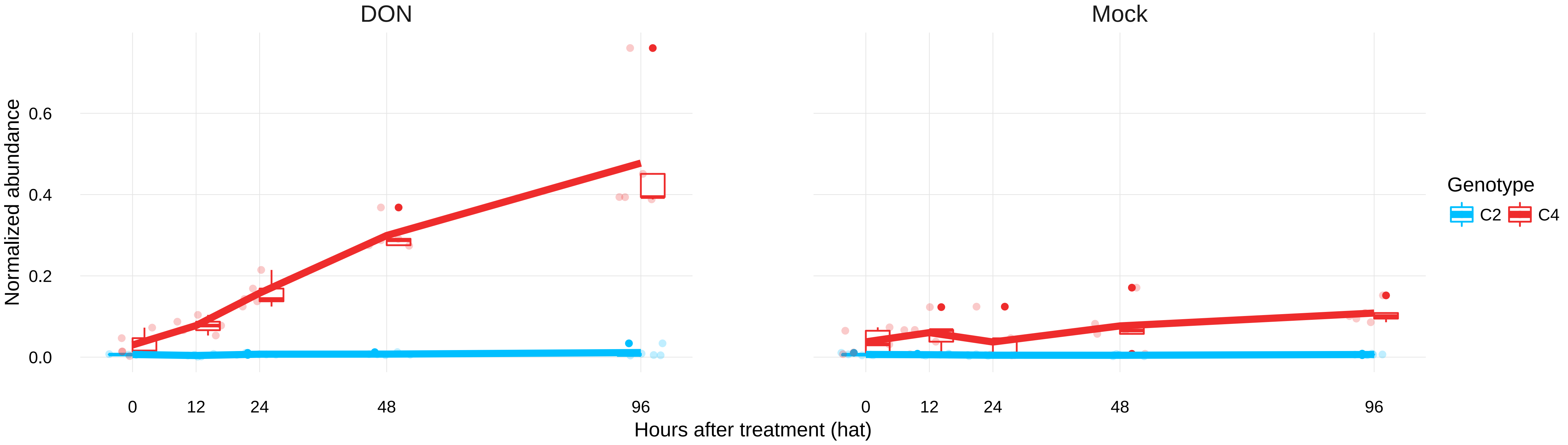

CM, Remus; different treatments

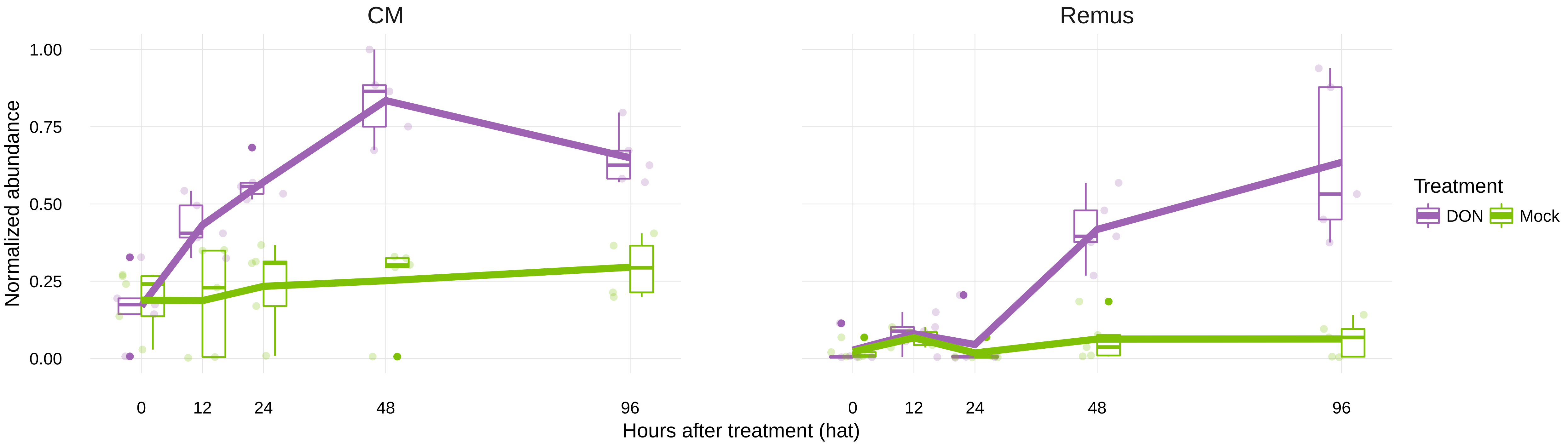

DON, Mock; all four genotypes

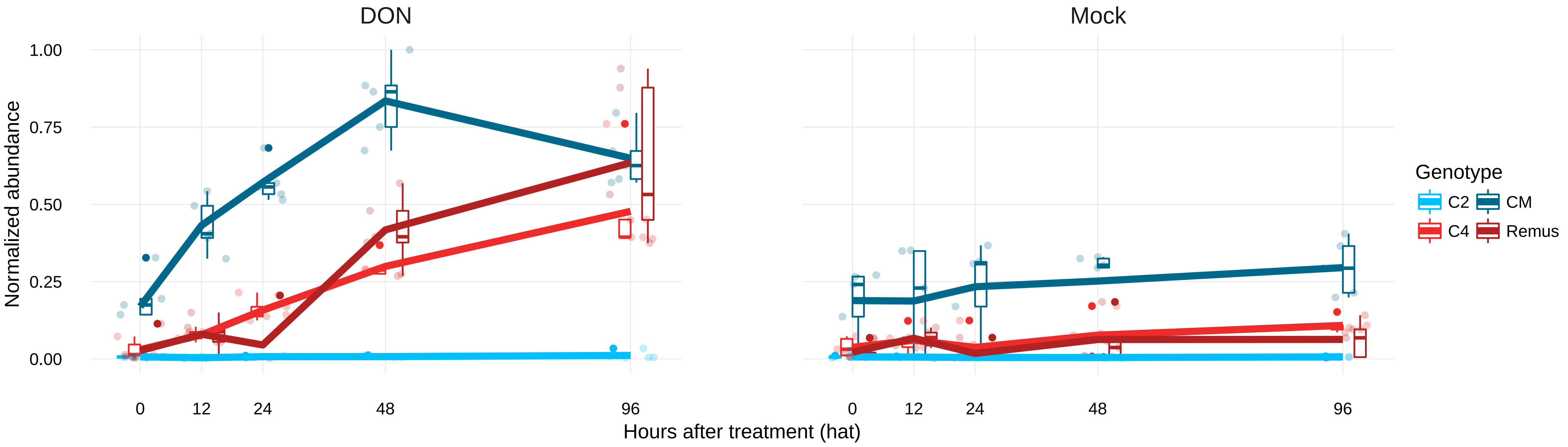

A.59

Annotated as HCA derivative
(1 database hit)

MZ	445.1343
RT	12.78 min
Normalization	Indirectly via surrogate in the KPX samples
Cluster	–
Cn total / Phe	19 / 9

C2, C4; different treatments

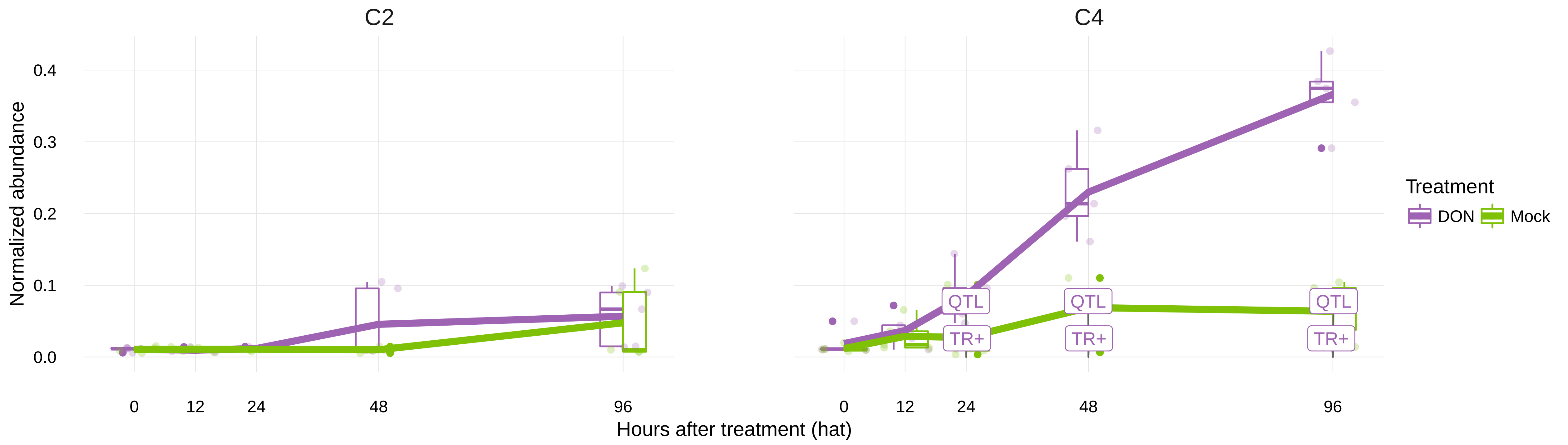

DON, Mock; different genotypes

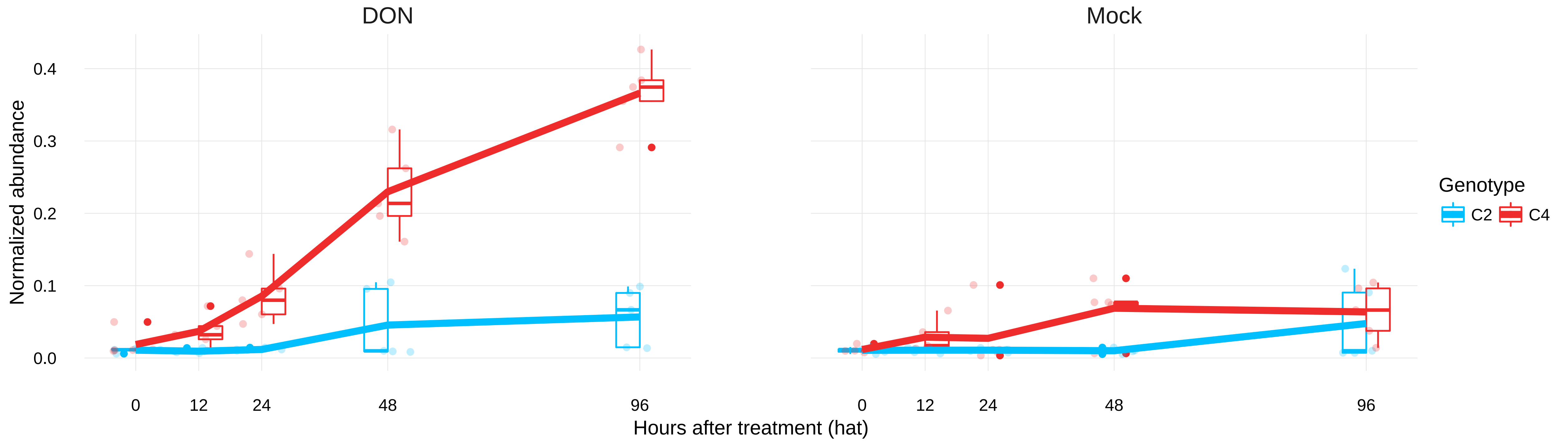

CM, Remus; different treatments

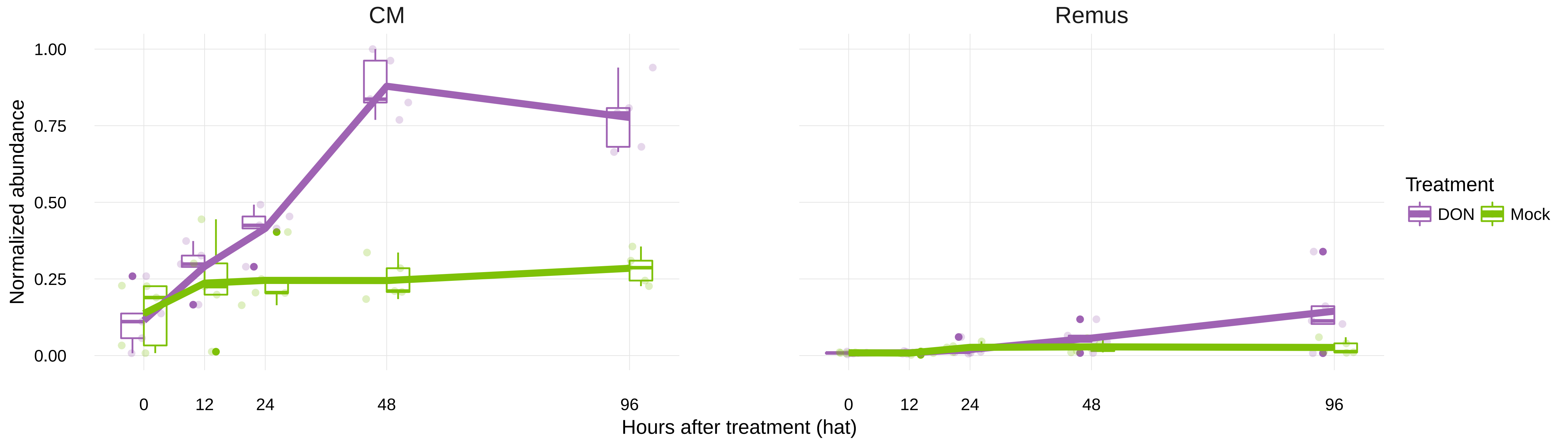

DON, Mock; all four genotypes

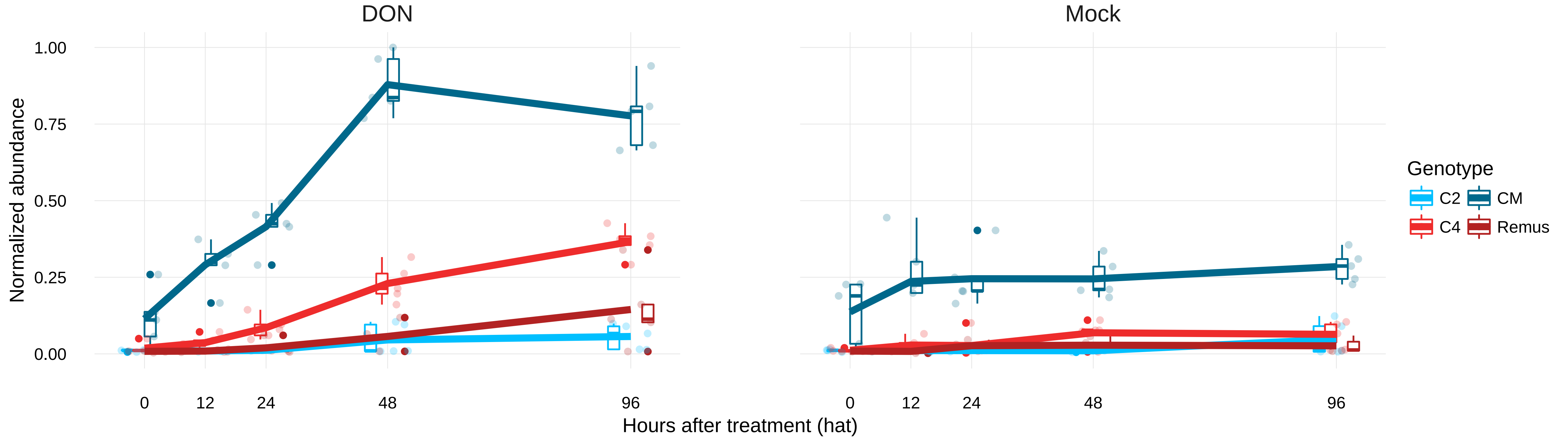

A.74

Annotated as Lignan
(2 database hits)

MZ	538.2285
RT	13.97 min
Normalization	Indirectly via surrogate in the KPX samples
Cluster	–
Cn total / Phe	26 / 18

C2, C4; different treatments

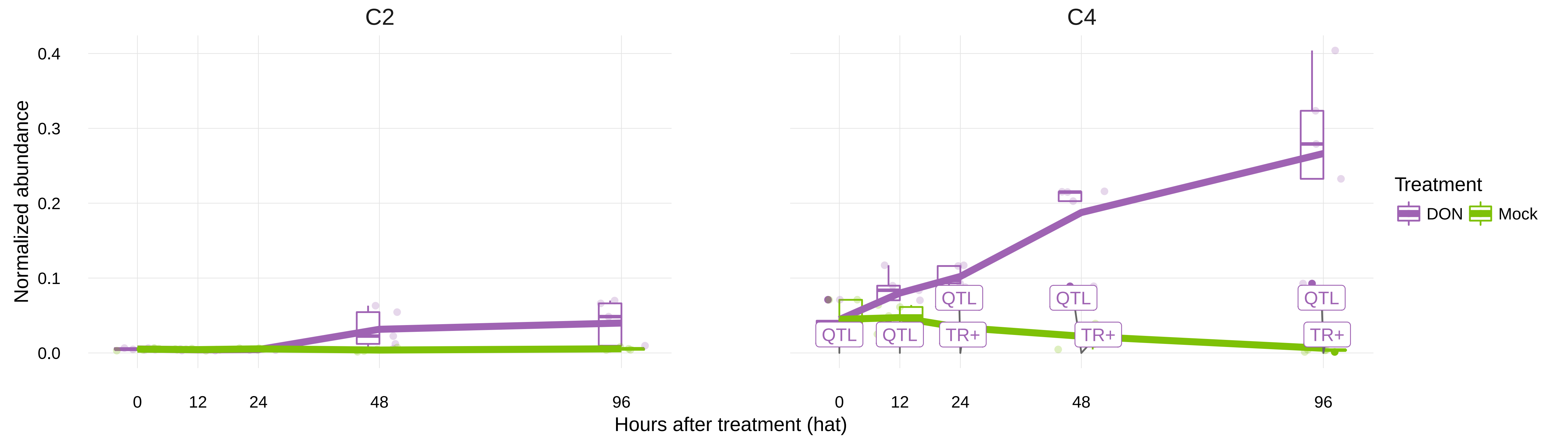

DON, Mock; different genotypes

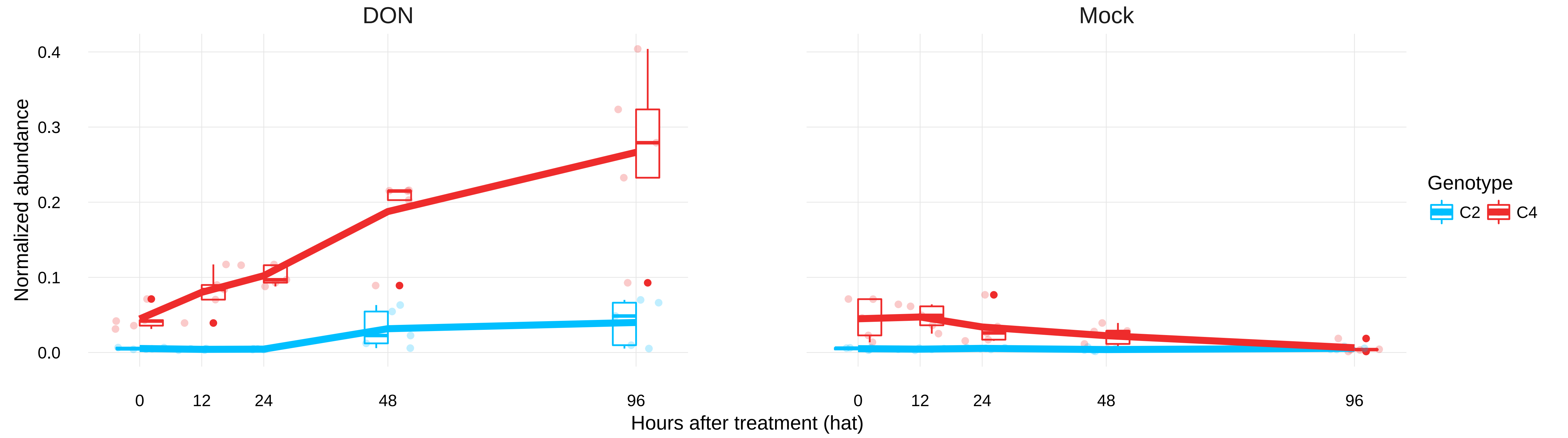

CM, Remus; different treatments

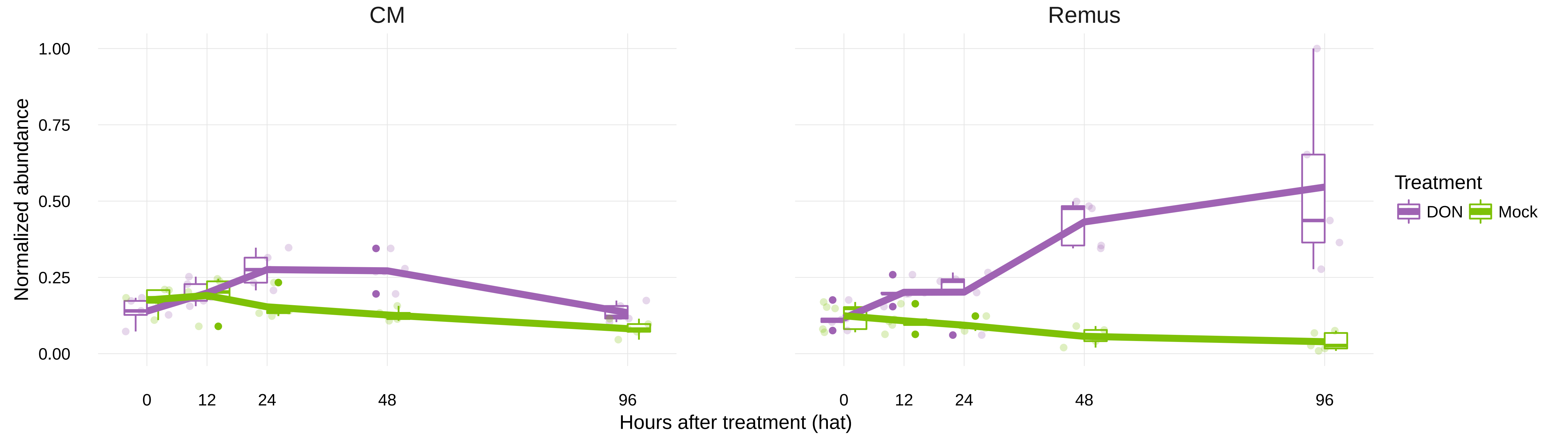

DON, Mock; all four genotypes

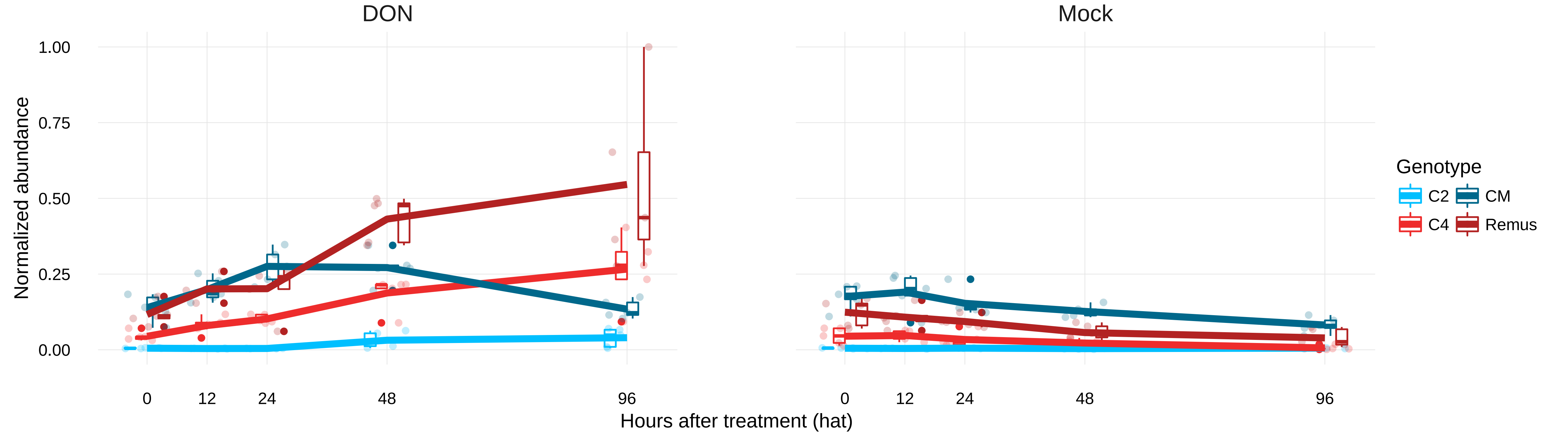

A.79

Annotated as Lignan
(1 database hit)

MZ	540.2441
RT	14.25 min
Normalization	Indirectly via surrogate in the KPX samples
Cluster	–
Cn total / Phe	26 / 18

C2, C4; different treatments

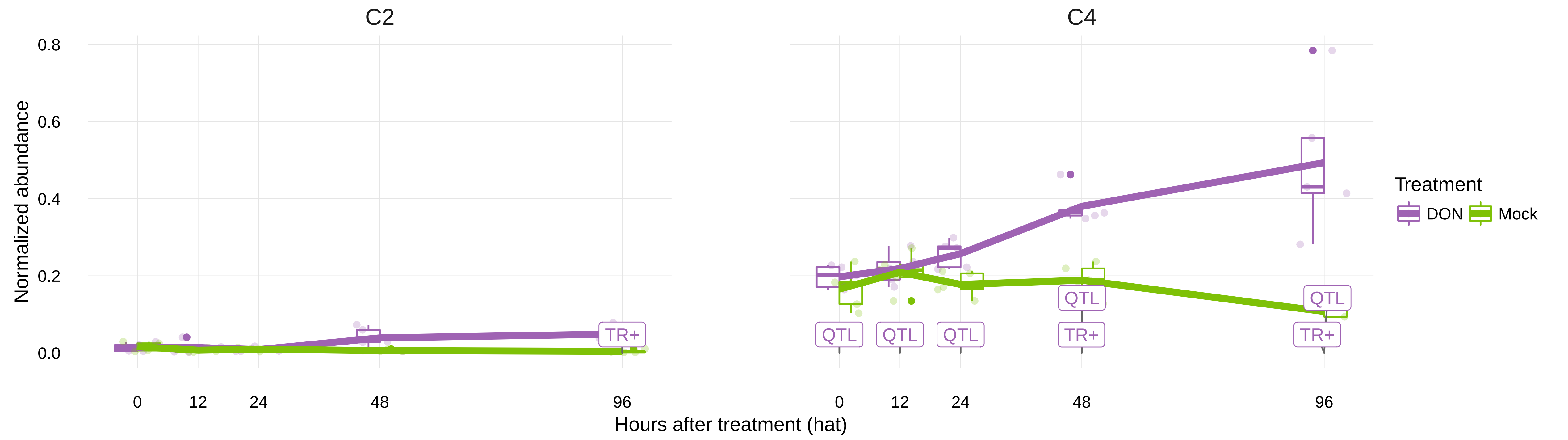

DON, Mock; different genotypes

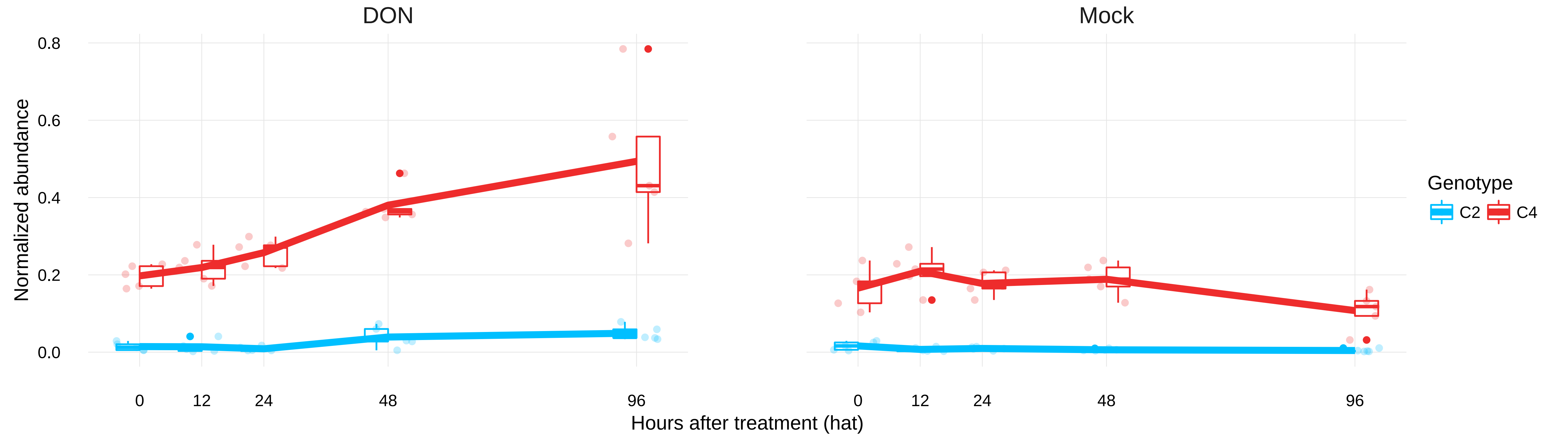

CM, Remus; different treatments

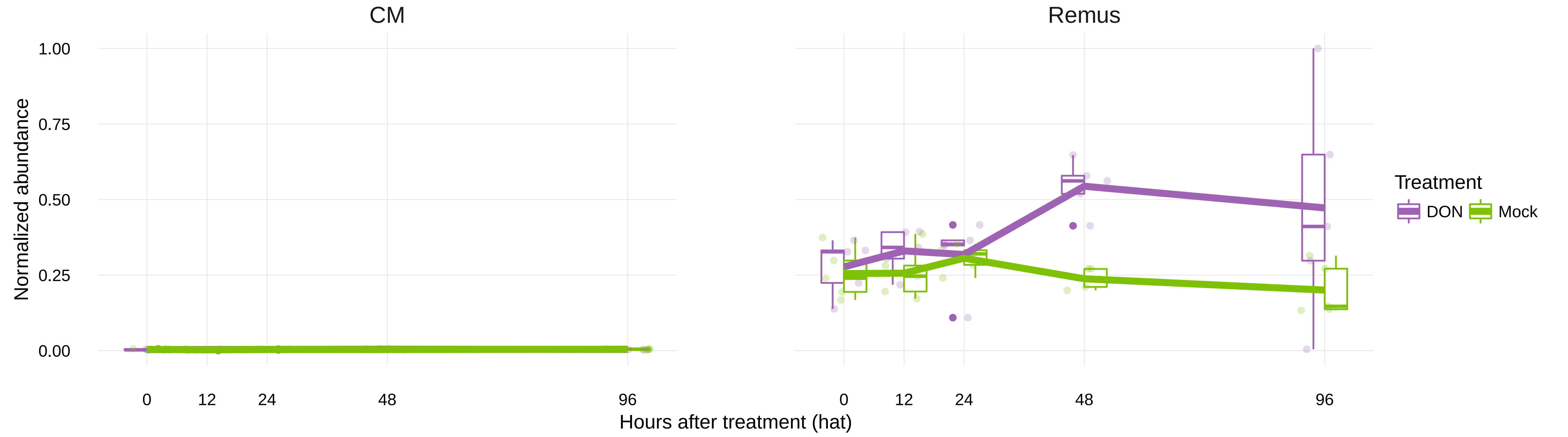

DON, Mock; all four genotypes

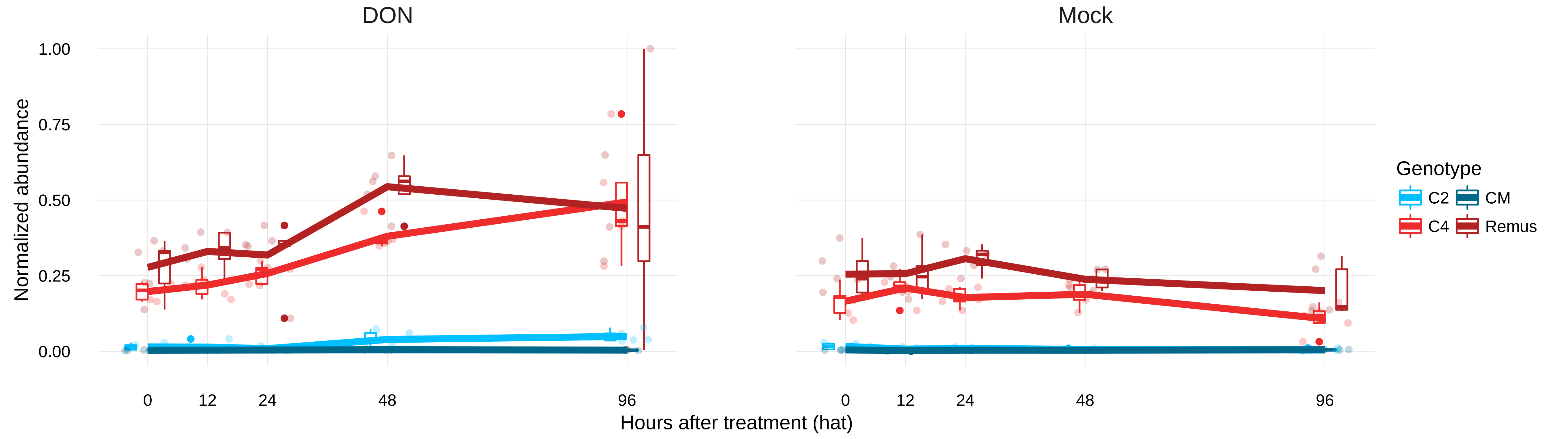

A.43

Annotated as Lignan
(1 database hit)

MZ	556.2392
RT	11.19 min
Normalization	Indirectly via surrogate in the KPX samples
Cluster	–
Cn total / Phe	26 / 18

C2, C4; different treatments

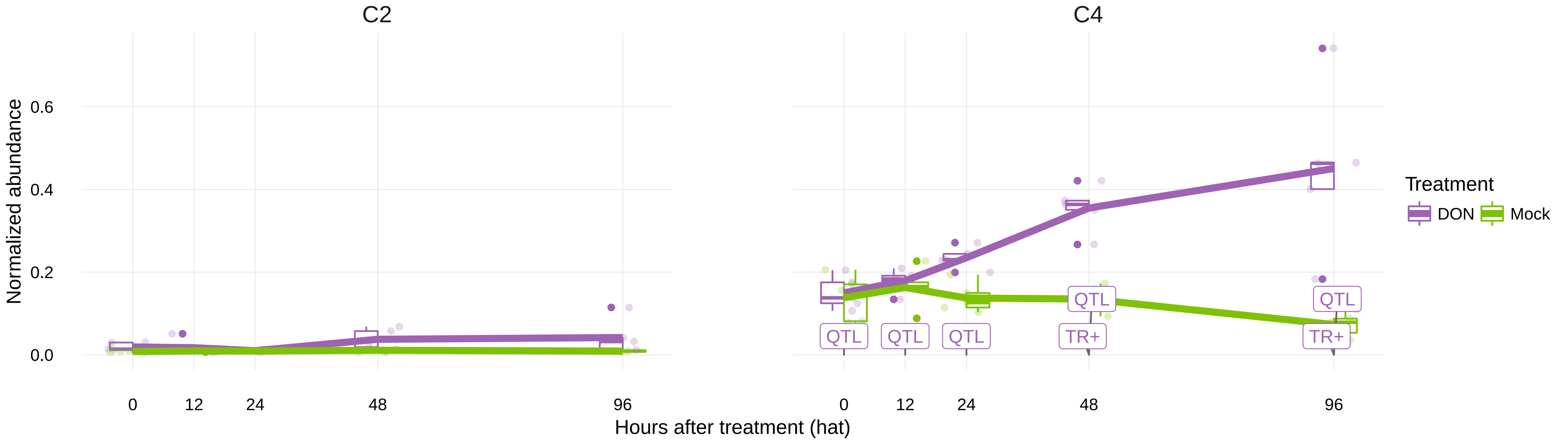

DON, Mock; different genotypes

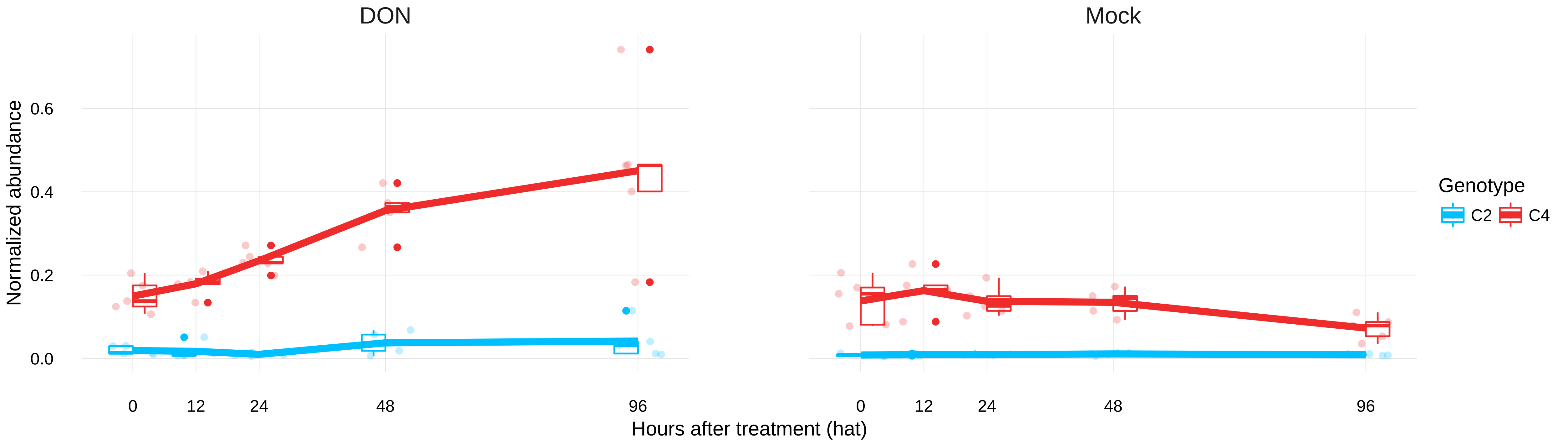

CM, Remus; different treatments

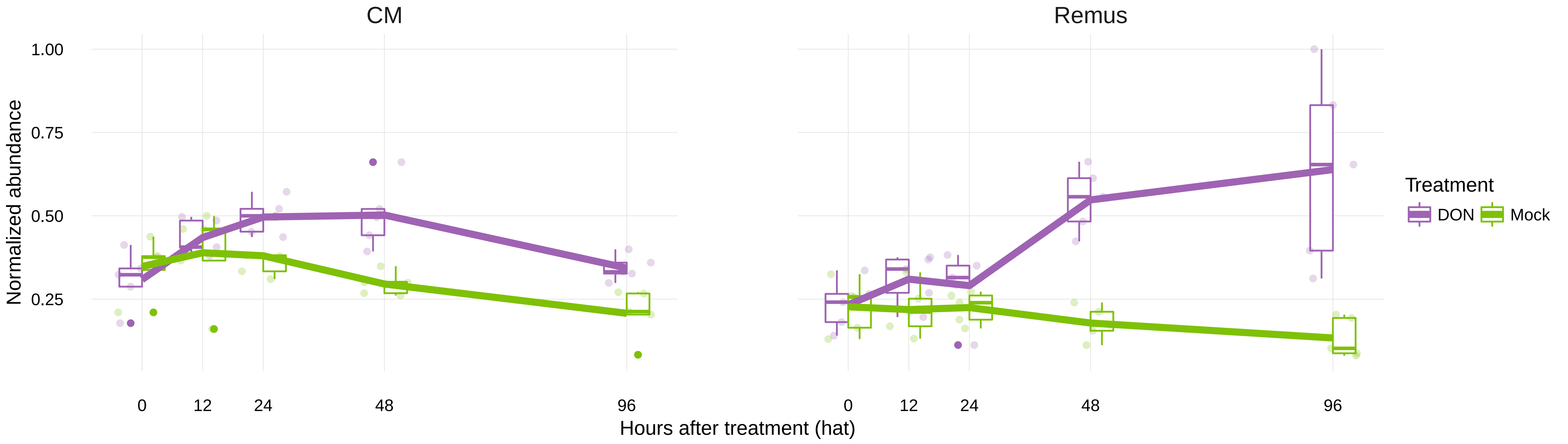

DON, Mock; all four genotypes

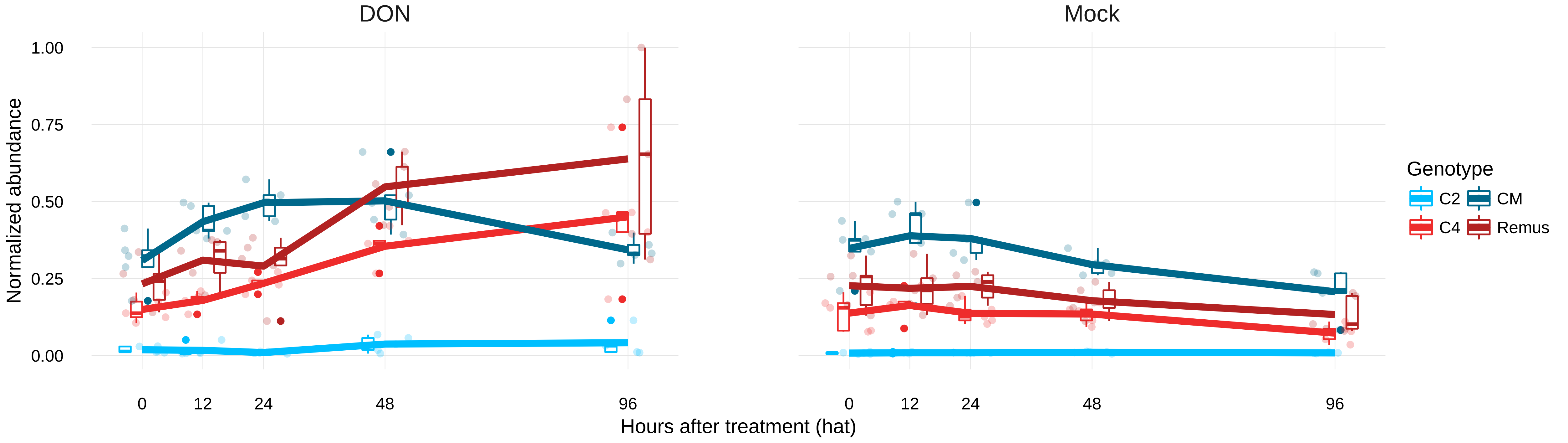

U.6

Unknown Phe−derived wheat−metabolite

MZ	395.0955
RT	12.79 min
Normalization	Directly via KPX samples
Cluster	−
Cn total / Phe	7 / 7

C2, C4; different treatments

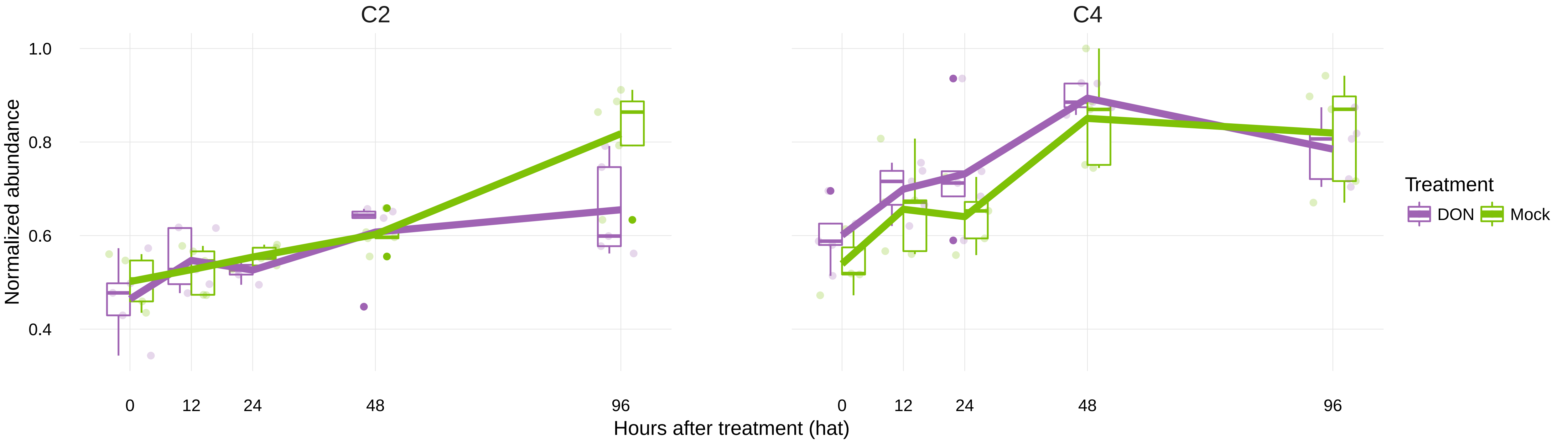

DON, Mock; different genotypes

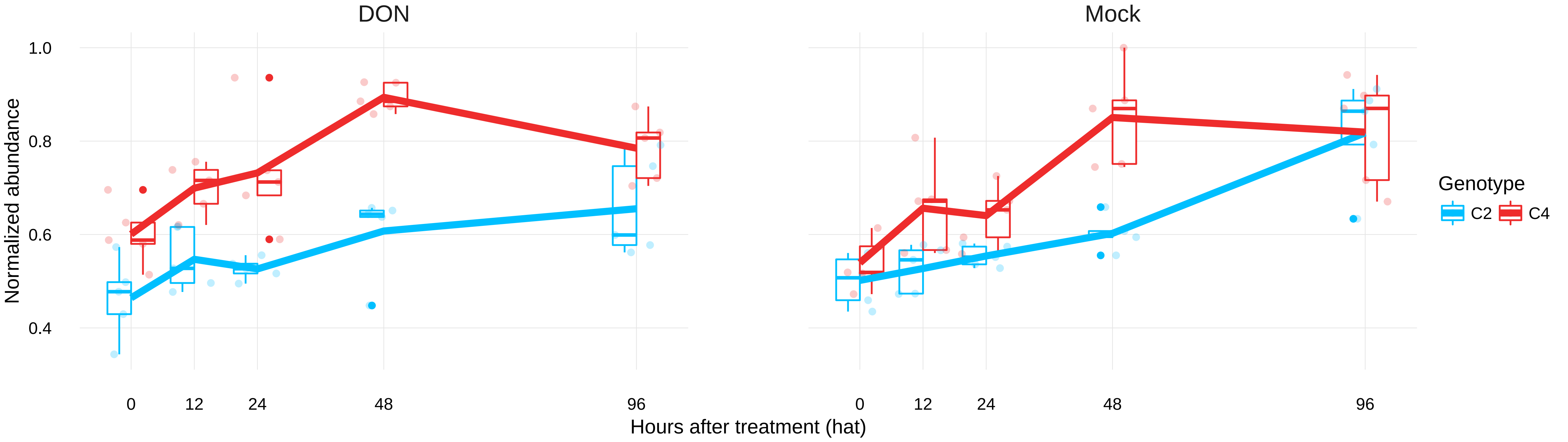

CM, Remus; different treatments

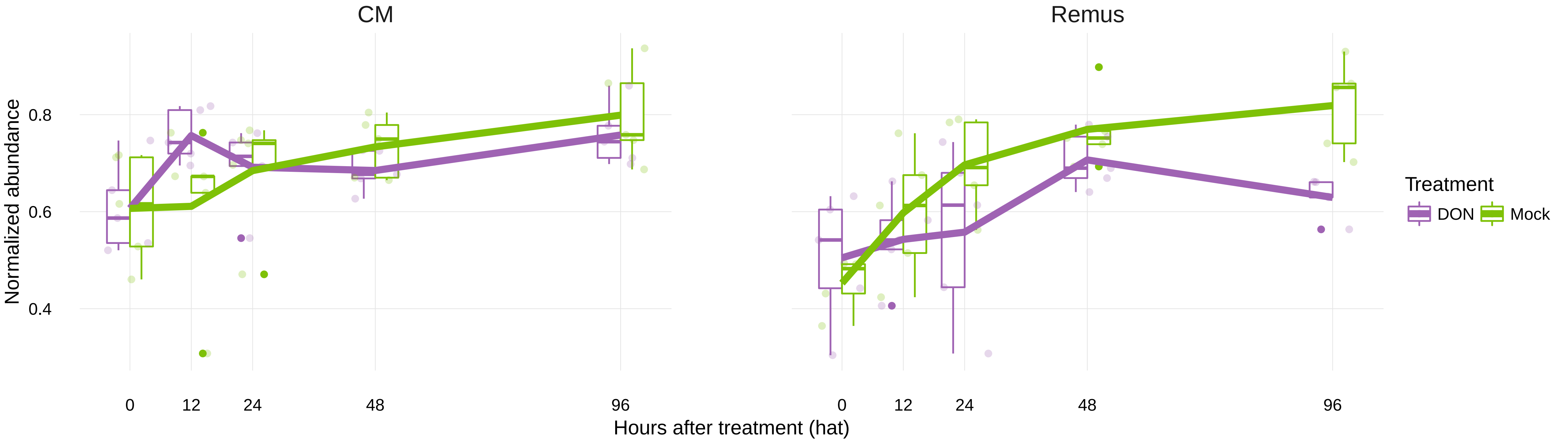

DON, Mock; all four genotypes

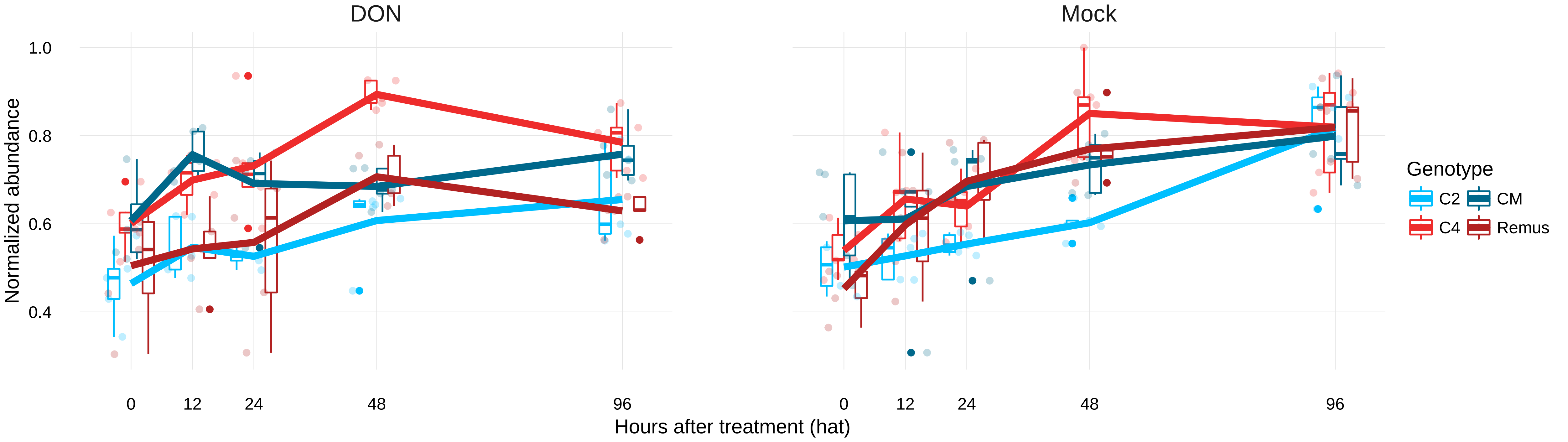

A.58

Annotated as Putative benzoic acid derivative
(1 database hit)

MZ	197.0809
RT	12.78 min
Normalization	Directly via KPX samples
Cluster	–
Cn total / Phe	10 / 7

C2, C4; different treatments

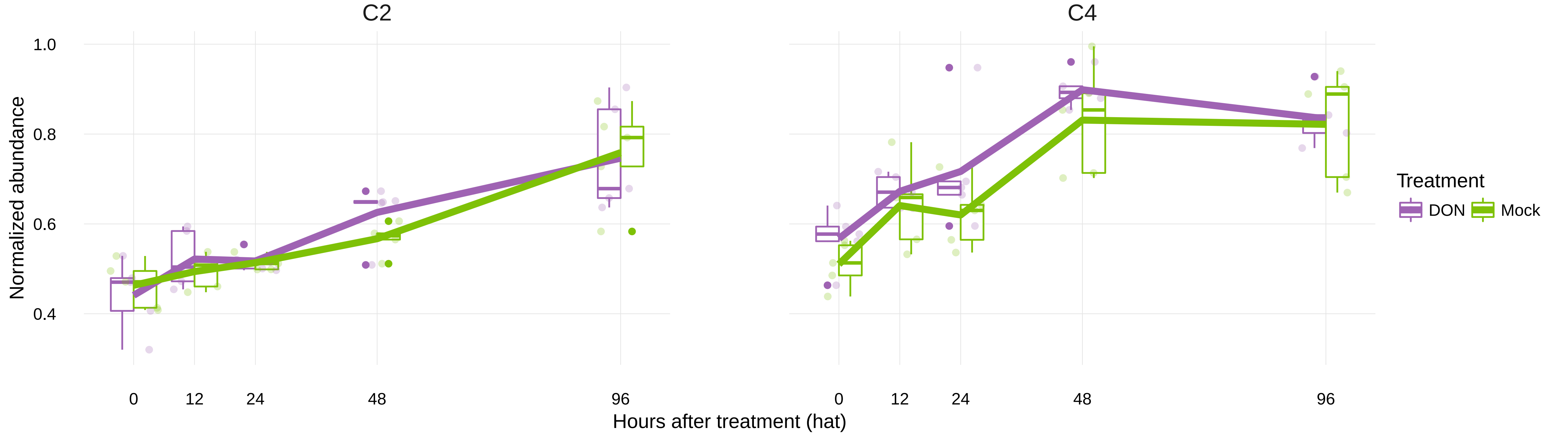

DON, Mock; different genotypes

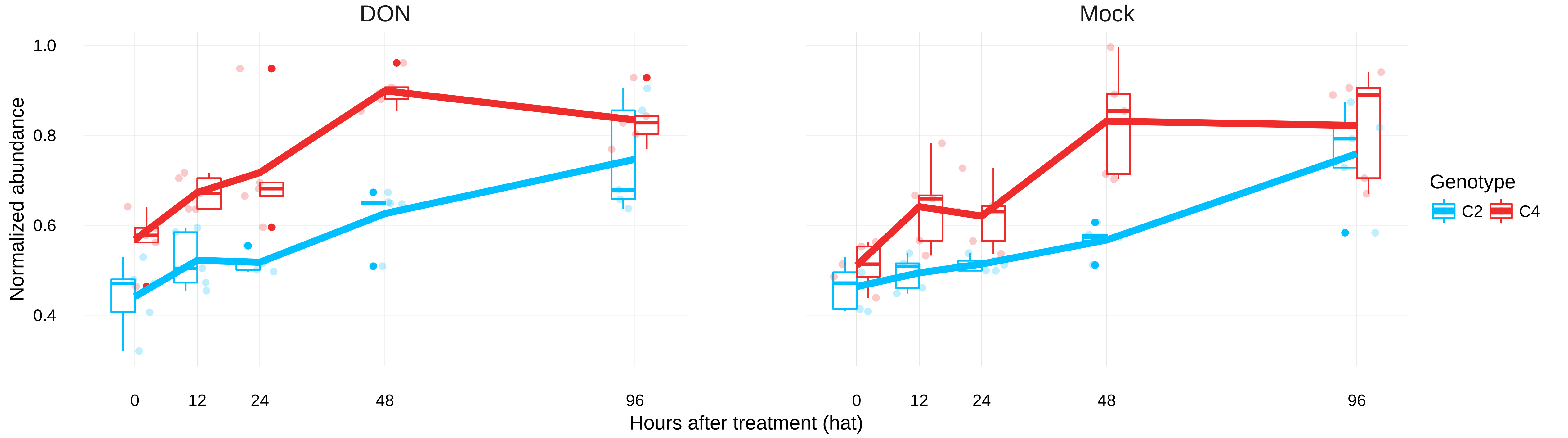

CM, Remus; different treatments

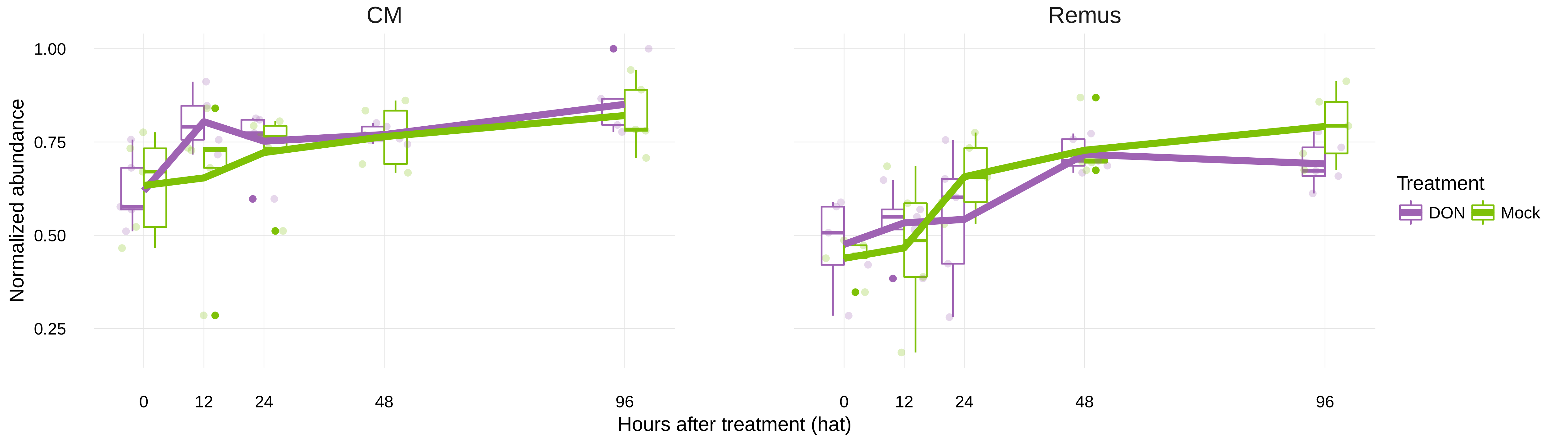

DON, Mock; all four genotypes

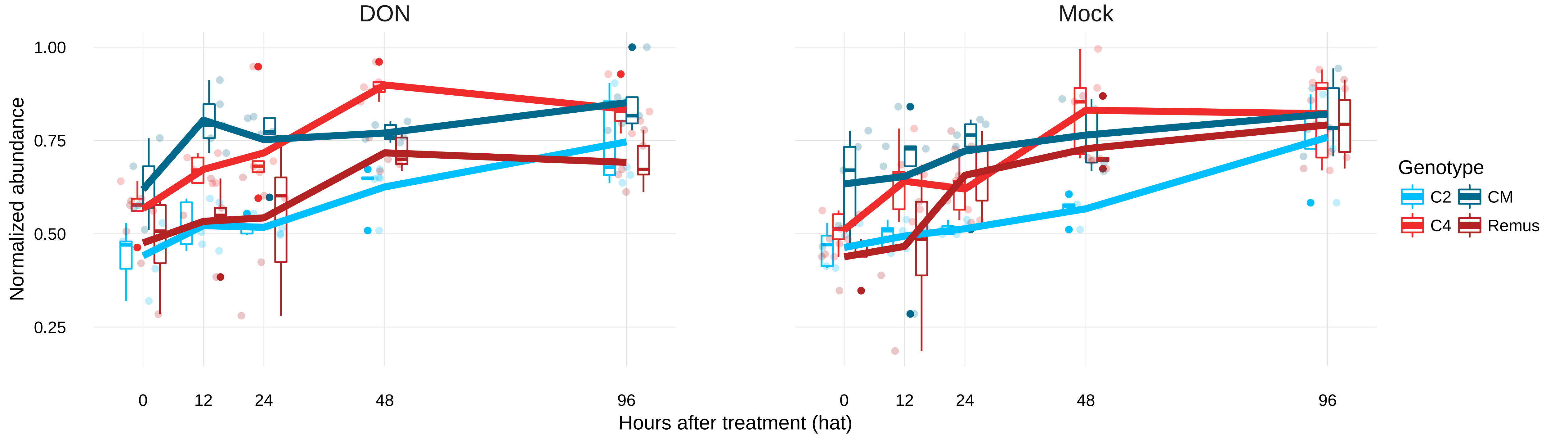

A.94

Annotated as Putative phenylacetic acid derivative
(2 database hits)

MZ	393.1158
RT	16.24 min
Normalization	Directly via KPX samples
Cluster	–
Cn total / Phe	17 / 8

C2, C4; different treatments

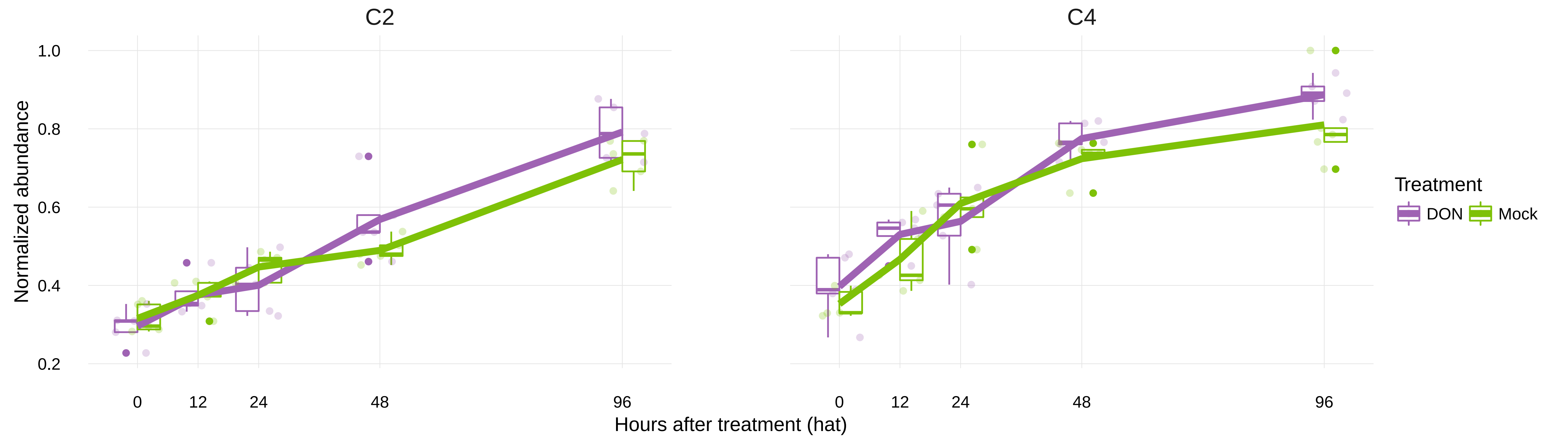

DON, Mock; different genotypes

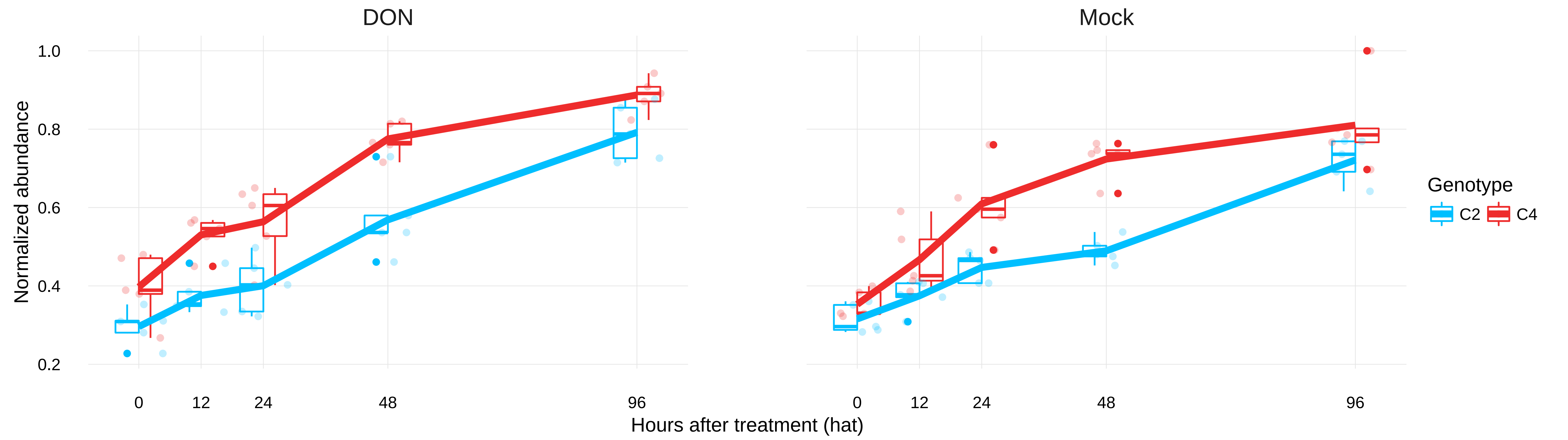

CM, Remus; different treatments

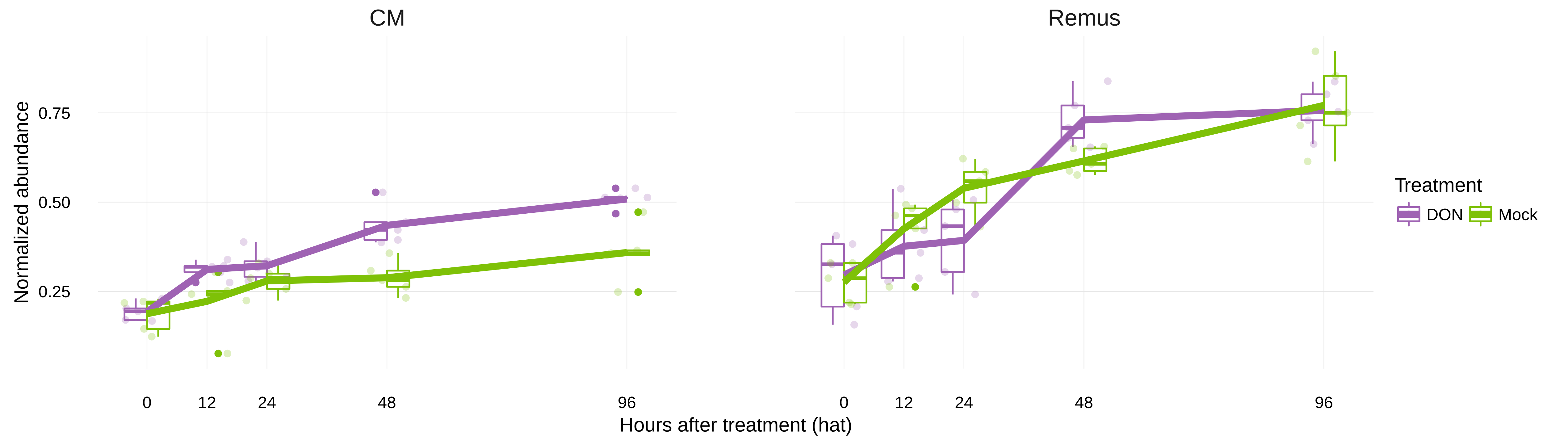

DON, Mock; all four genotypes

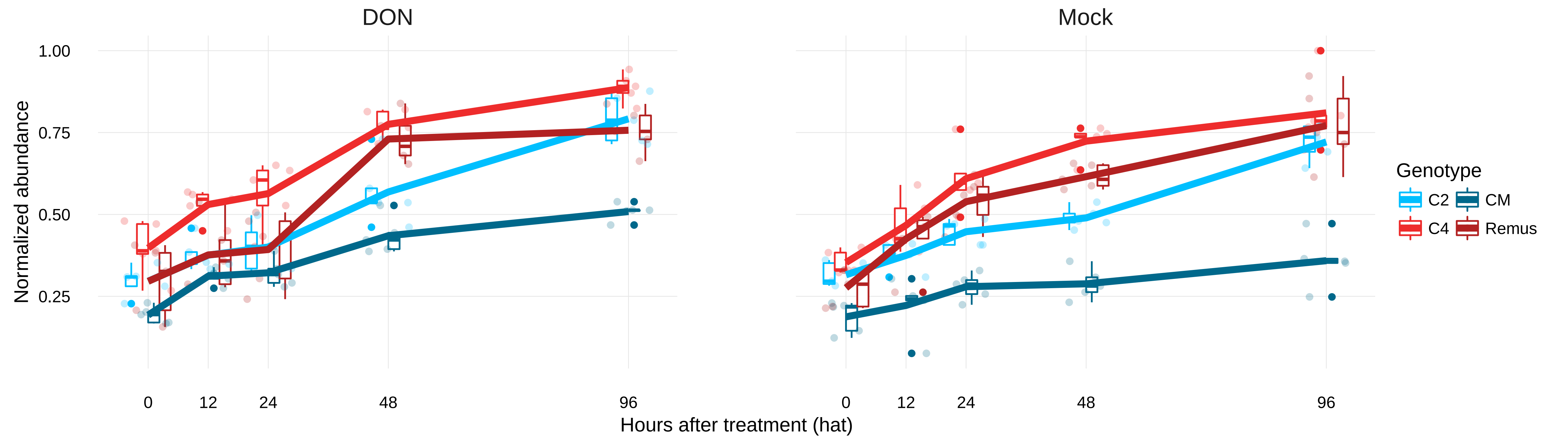

A.96

Annotated as Putative phenylacetic acid derivative
(1 database hit)

MZ	335.1128
RT	16.27 min
Normalization	Indirectly via surrogate in the KPX samples
Cluster	Cluster 2
Cn total / Phe	17 / 8

C2, C4; different treatments

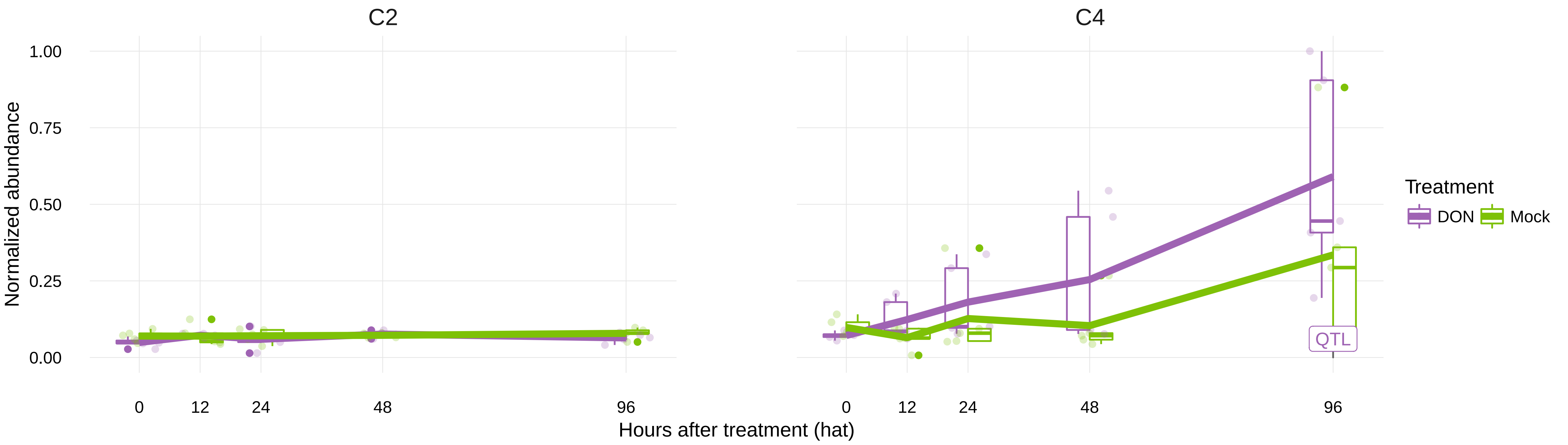

DON, Mock; different genotypes

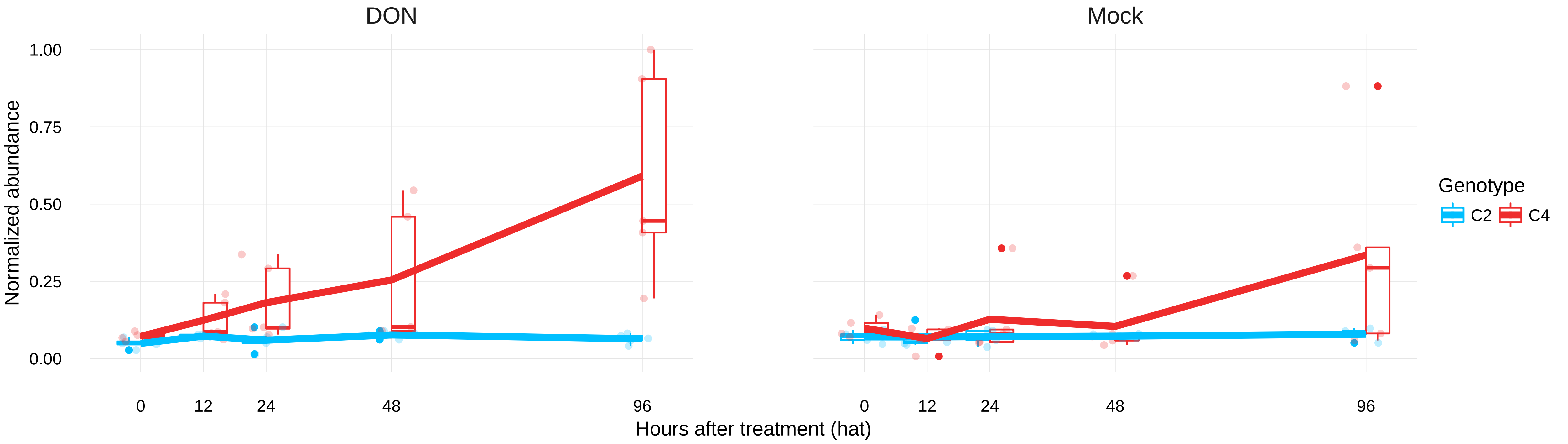

CM, Remus; different treatments

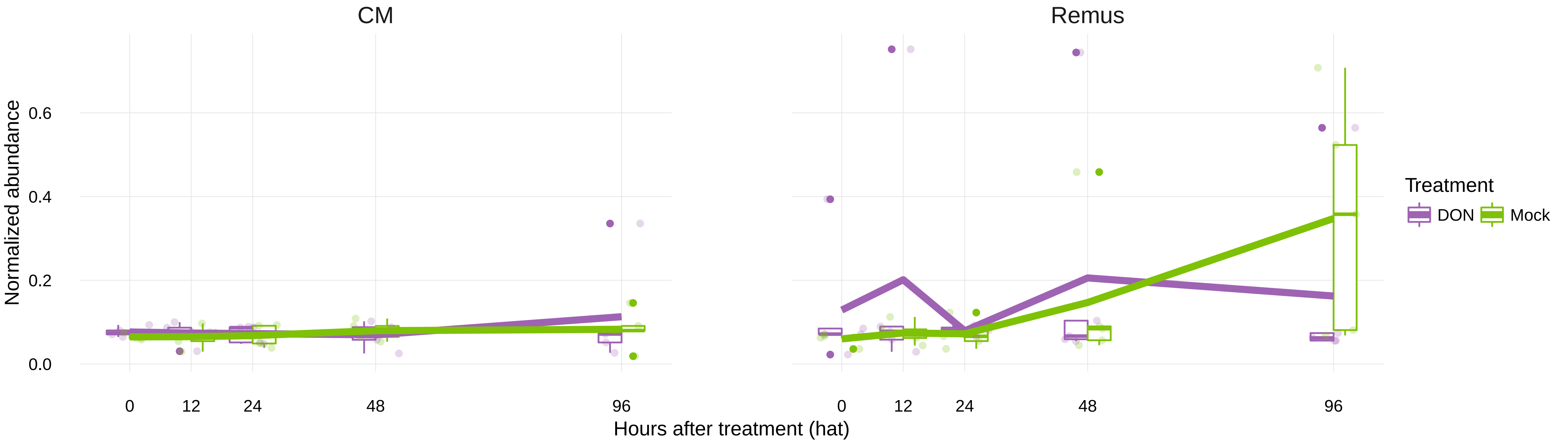

DON, Mock; all four genotypes

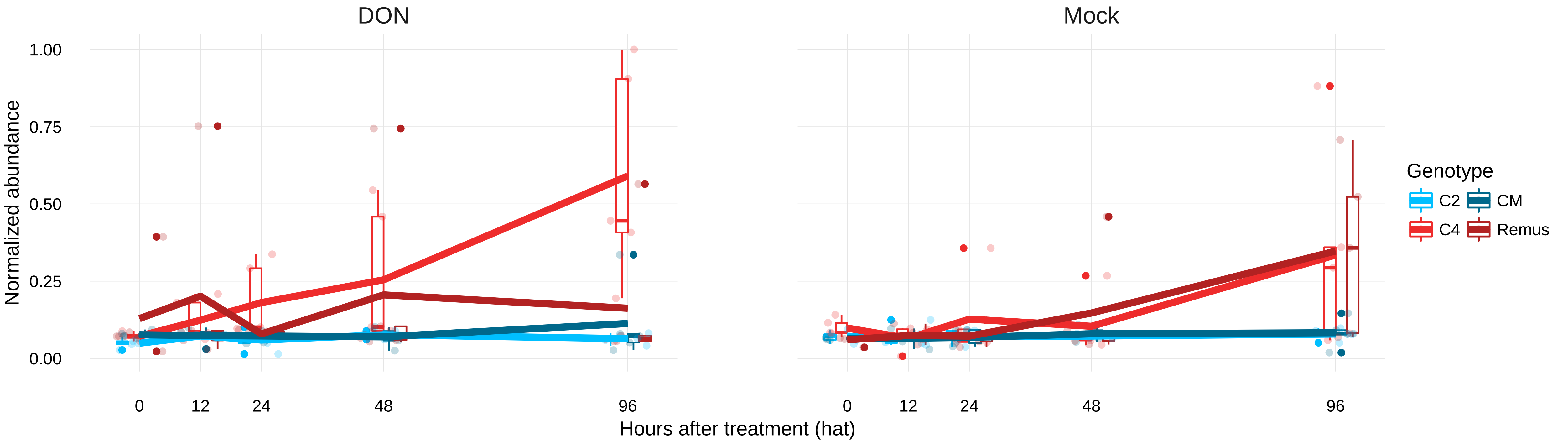

A.80

Annotated as Lignan
(1 database hit)

MZ	600.2654
RT	14.55 min
Normalization	Indirectly via surrogate in the KPX samples
Cluster	Cluster 2
Cn total / Phe	28 / 9

C2, C4; different treatments

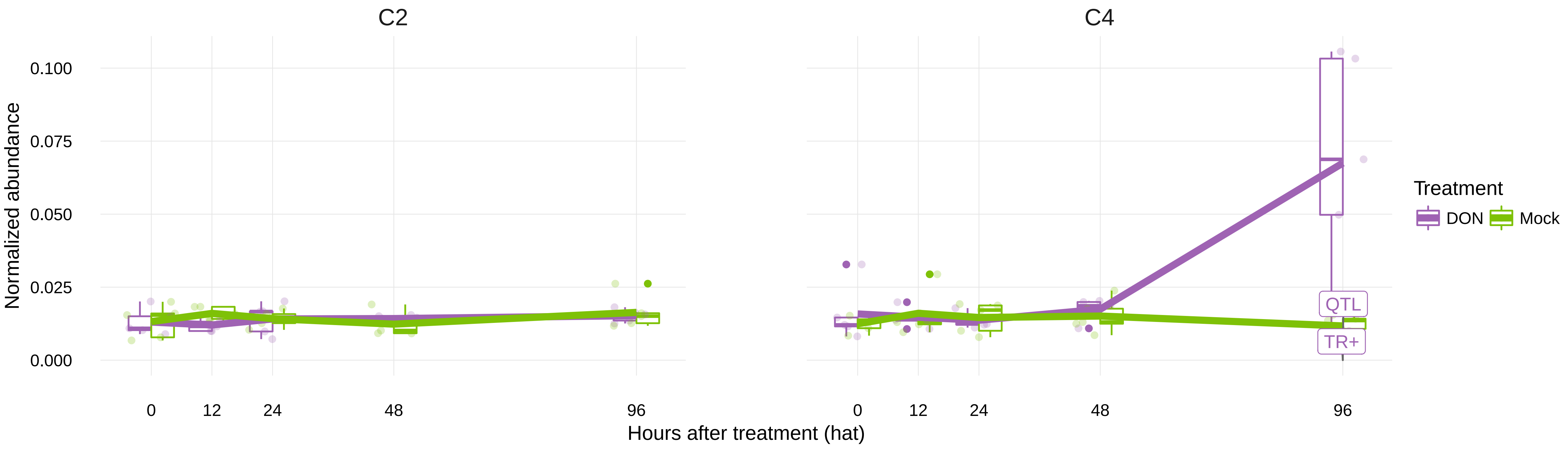

DON, Mock; different genotypes

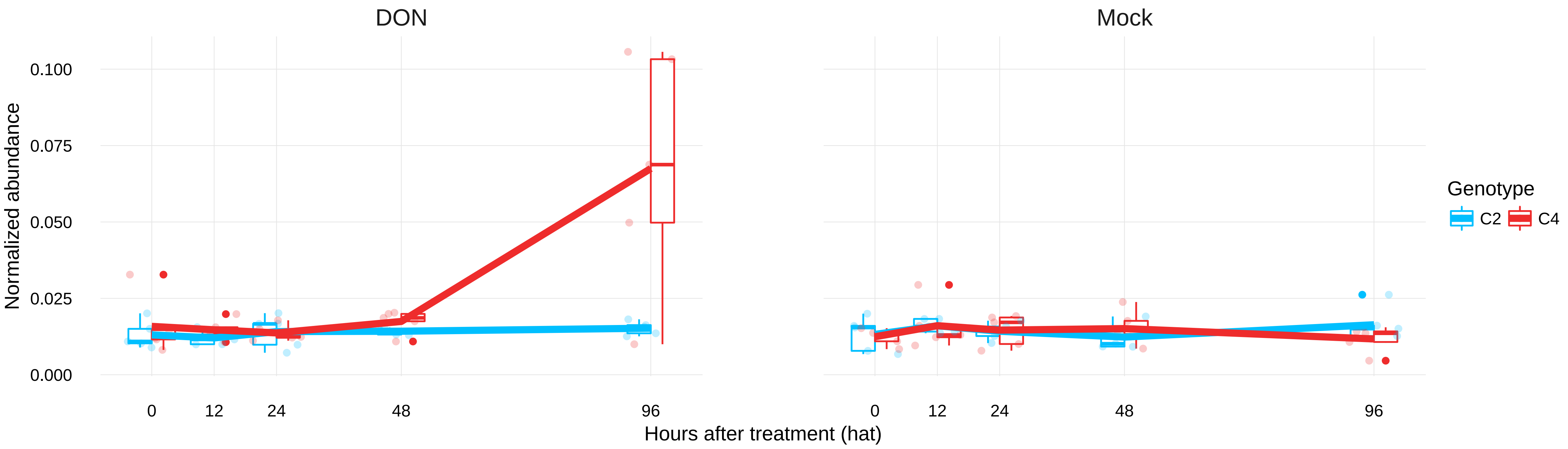

CM, Remus; different treatments

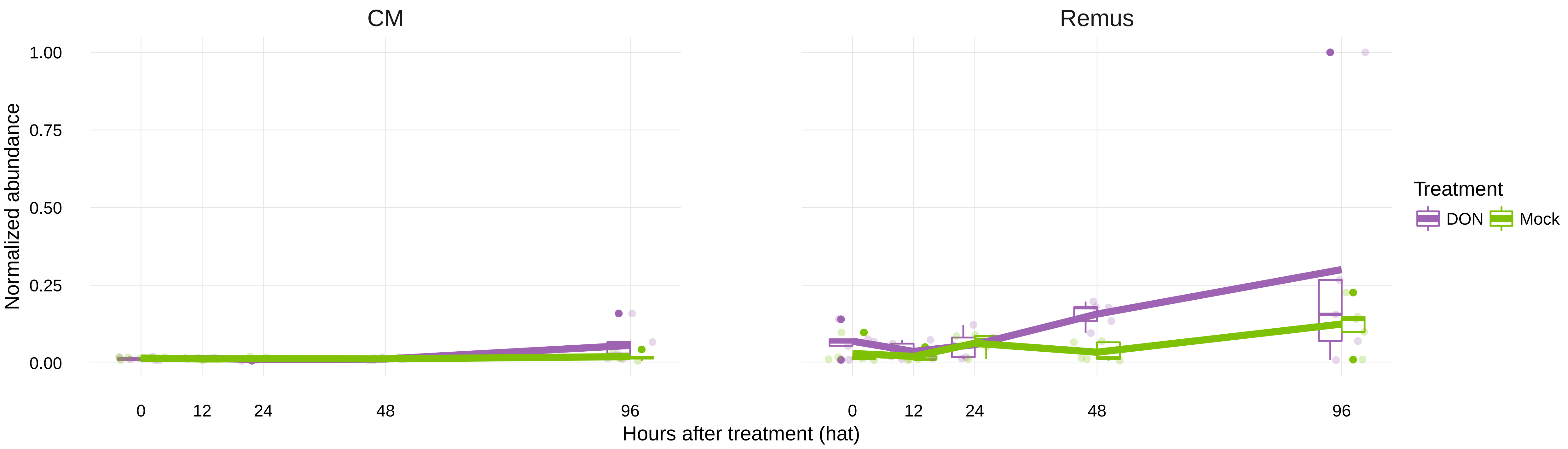

DON, Mock; all four genotypes

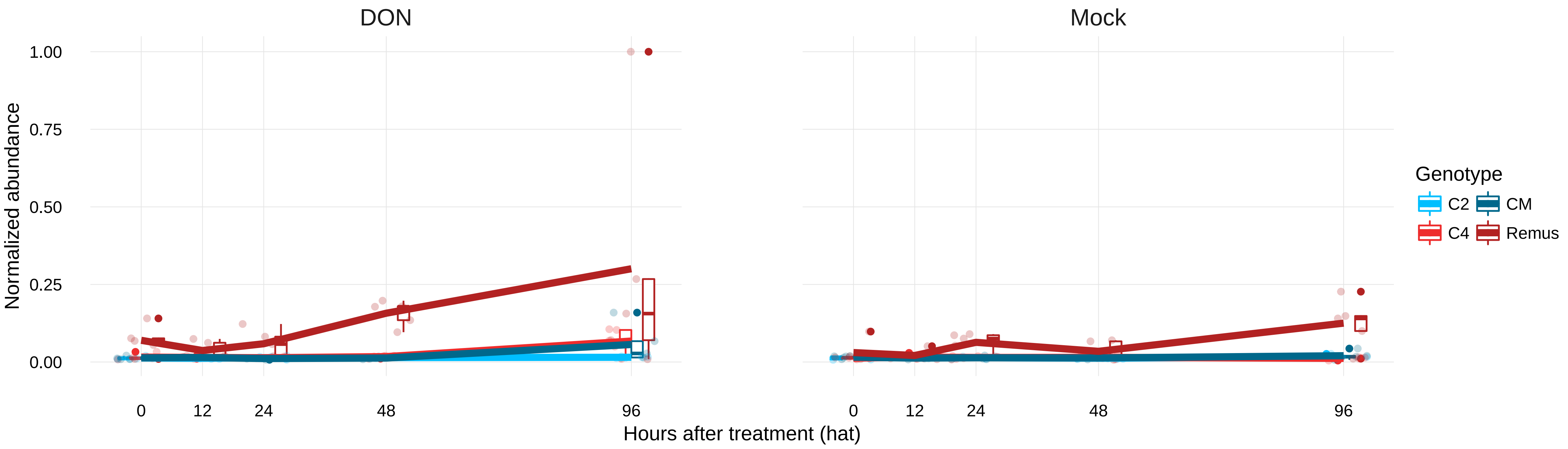

A.61

Annotated as HCA derivative
(1 database hit)

MZ	586.2496
RT	12.88 min
Normalization	Indirectly via surrogate in the KPX samples
Cluster	Cluster 2
Cn total / Phe	27 / 9

C2, C4; different treatments

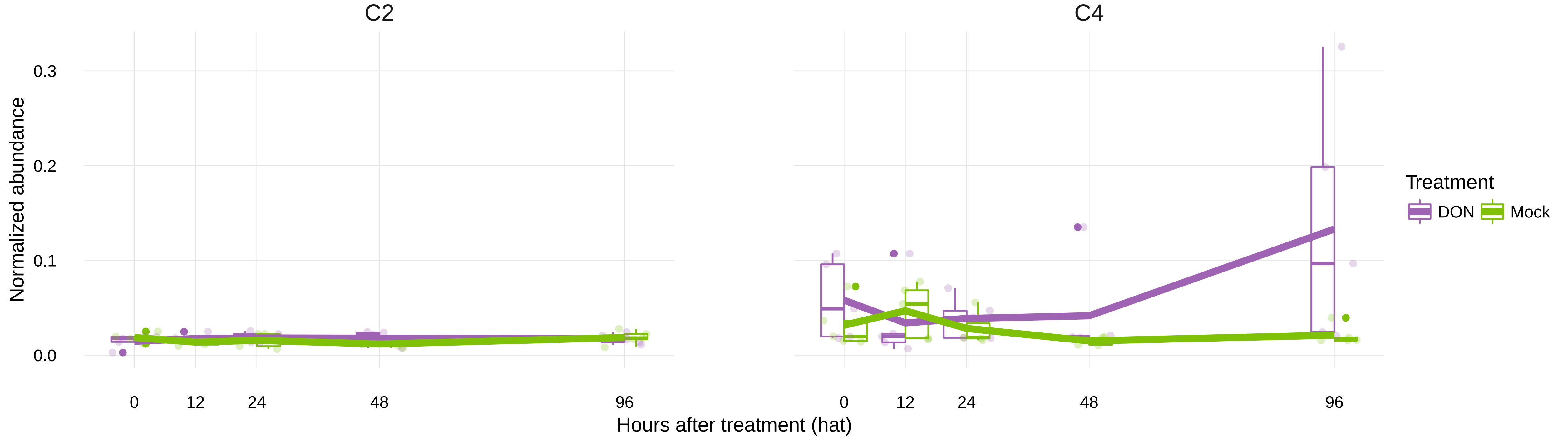

DON, Mock; different genotypes

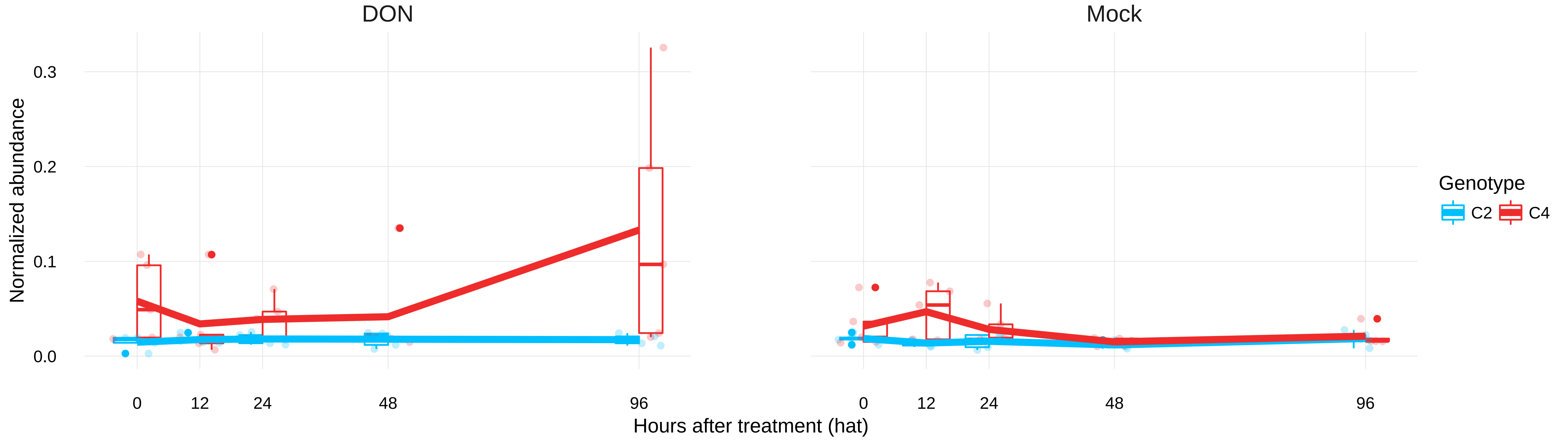

CM, Remus; different treatments

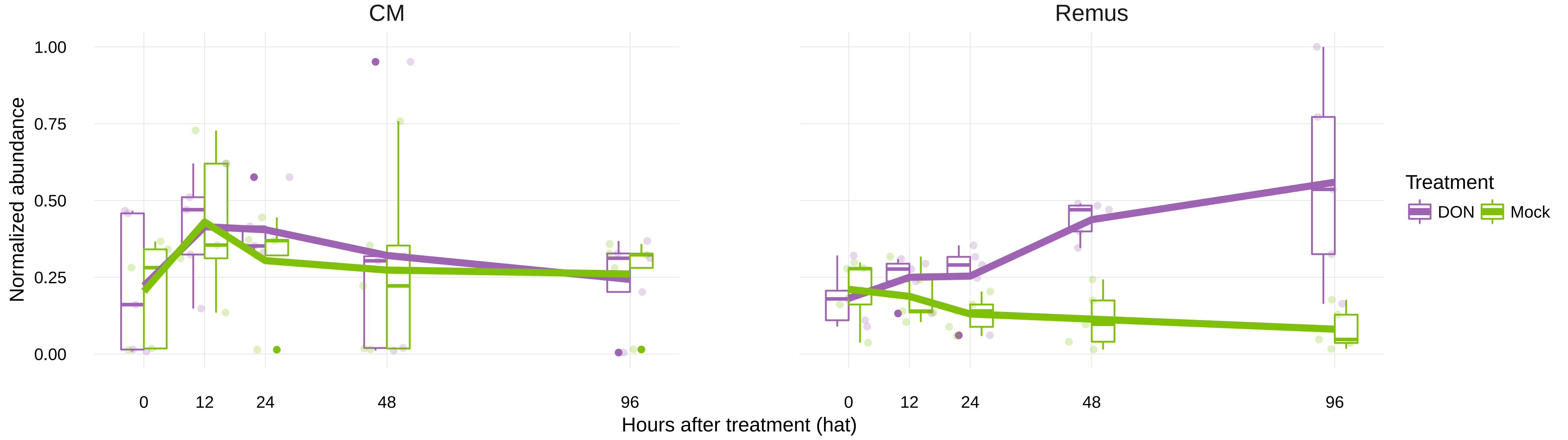

DON, Mock; all four genotypes

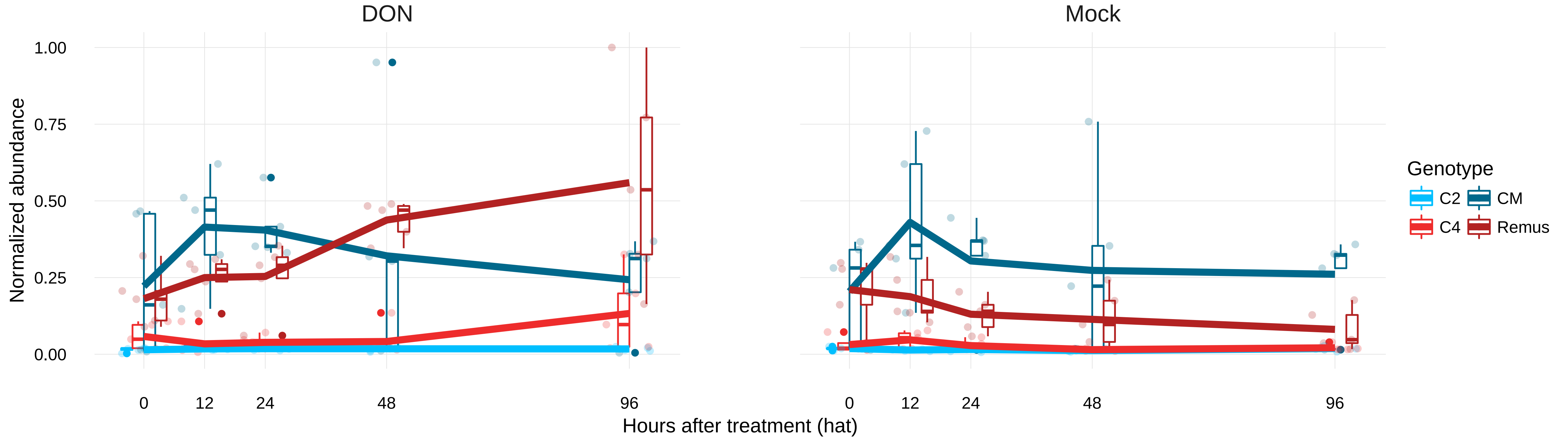

A.119

Annotated as Flavonoid
(21 database hits)

MZ	371.149
RT	19 min
Normalization	Indirectly via surrogate in the KPX samples
Cluster	Cluster 2
Cn total / Phe	21 /

C2, C4; different treatments

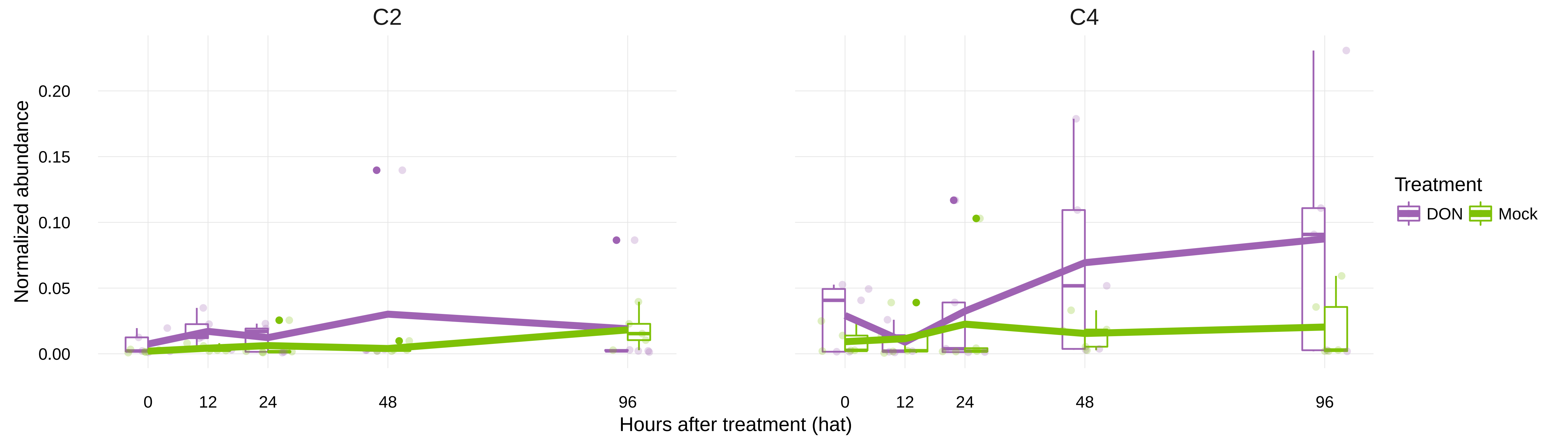

DON, Mock; different genotypes

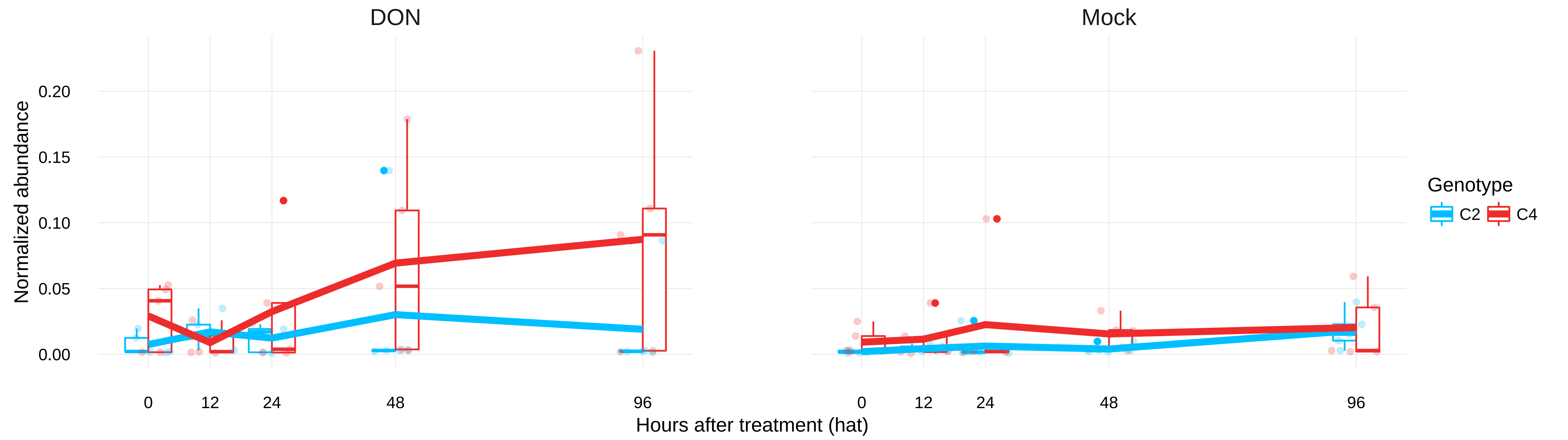

CM, Remus; different treatments

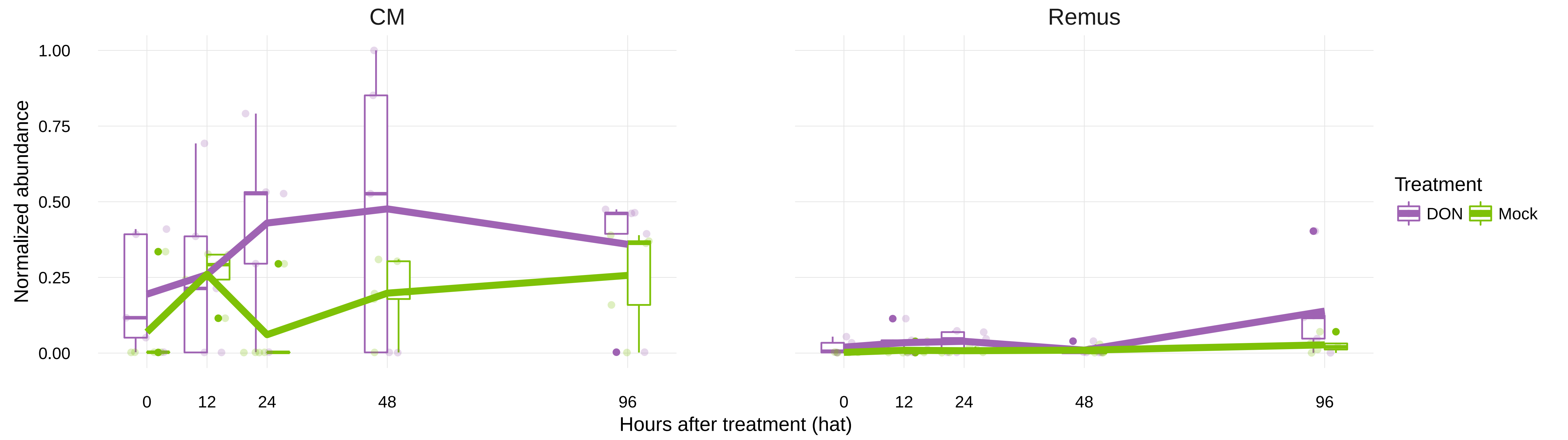

DON, Mock; all four genotypes

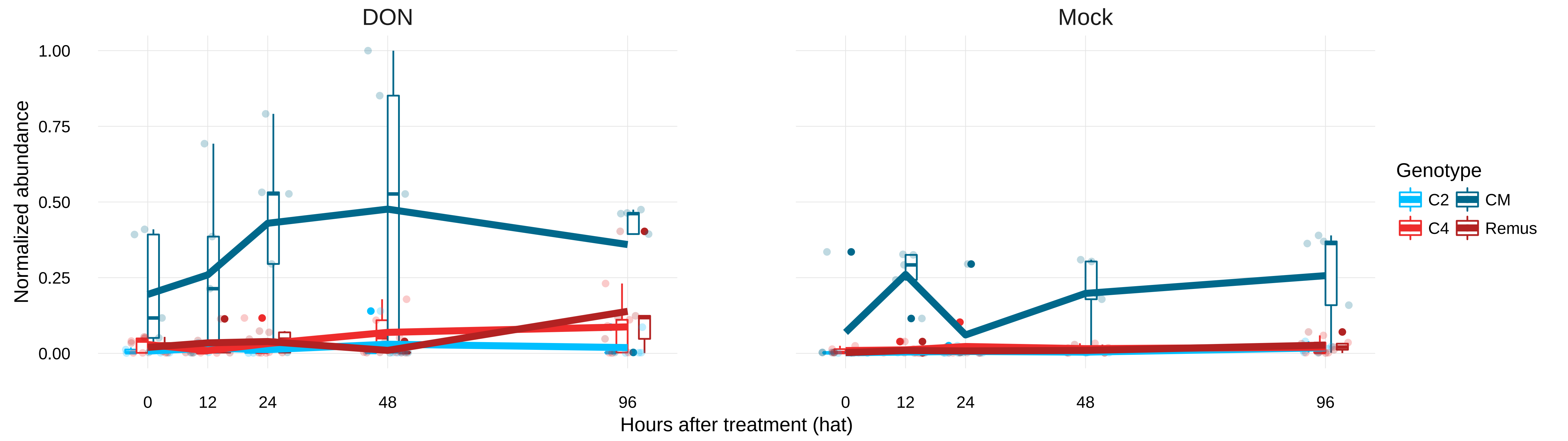

A.65

Annotated as Lignan
(1 database hit)

MZ	540.2439
RT	13.15 min
Normalization	Indirectly via surrogate in the KPX samples
Cluster	Cluster 2
Cn total / Phe	26 / 18

C2, C4; different treatments

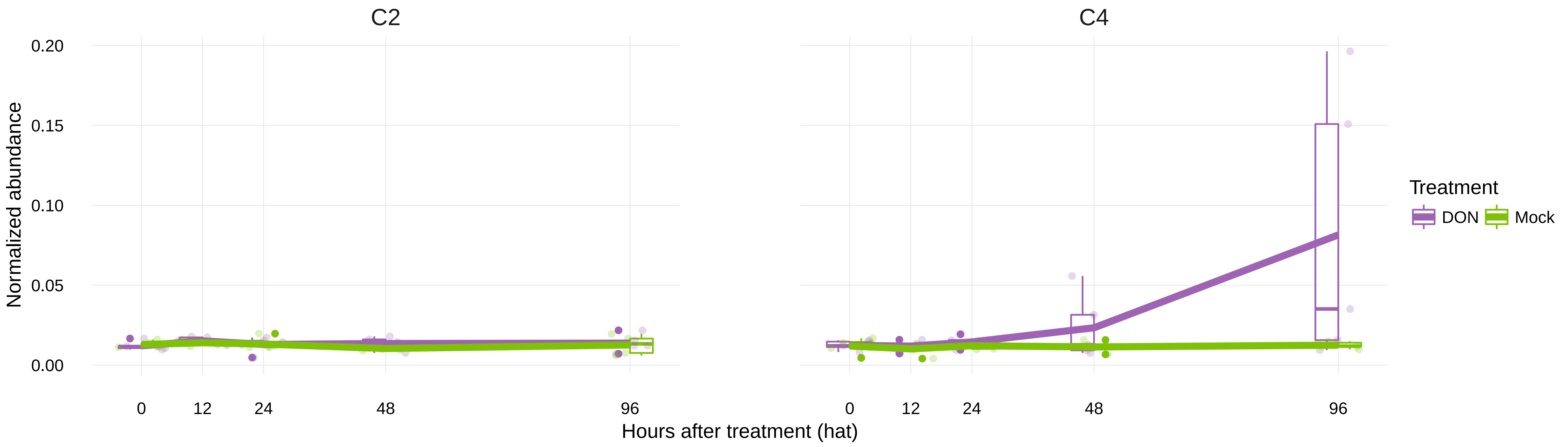

DON, Mock; different genotypes

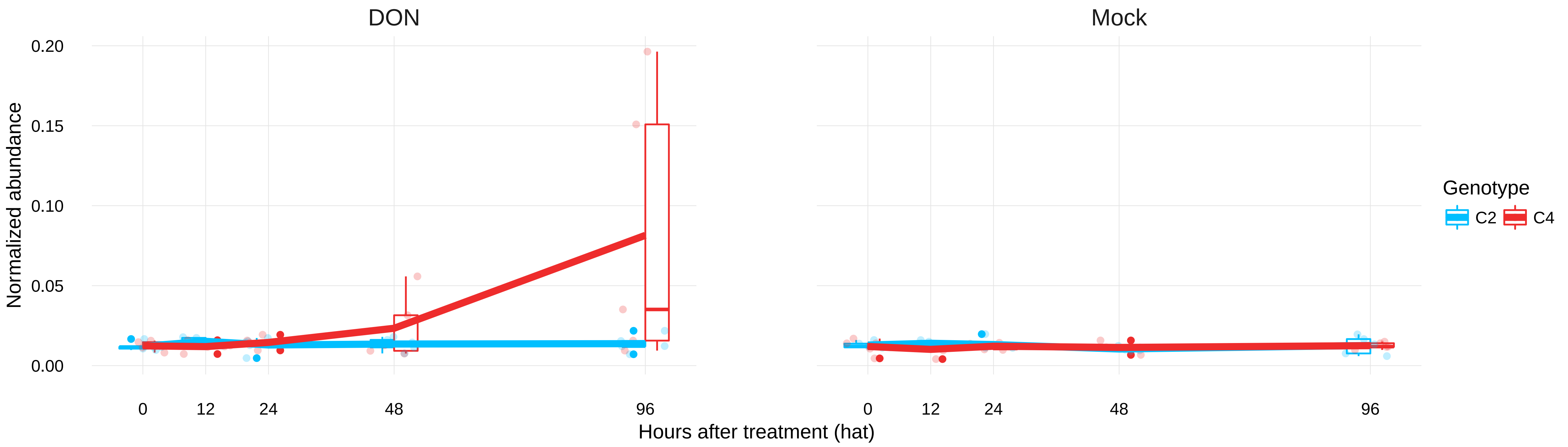

CM, Remus; different treatments

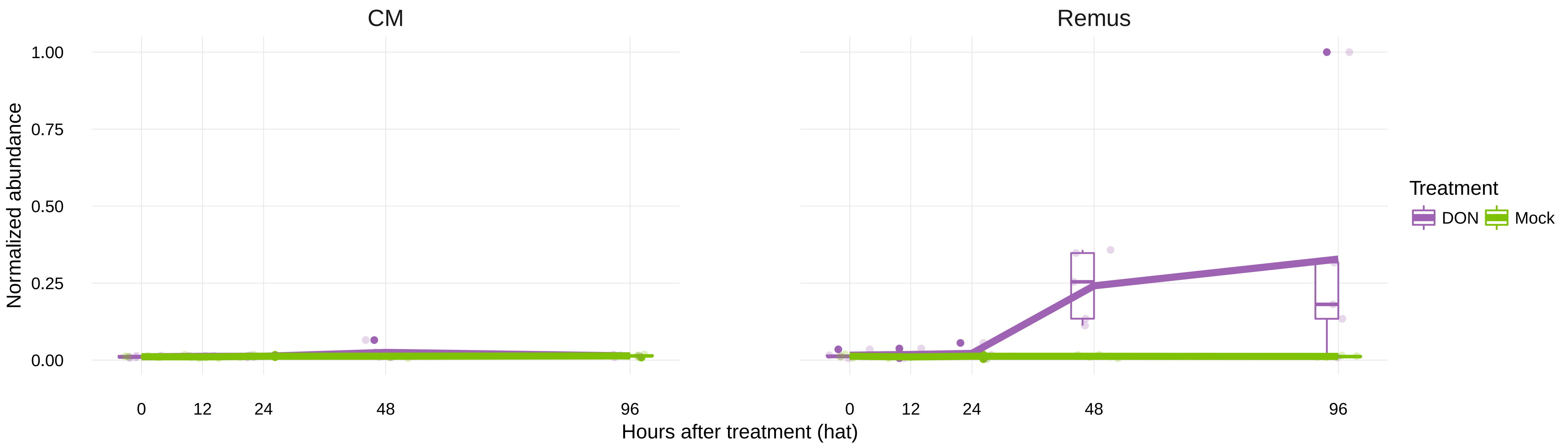

DON, Mock; all four genotypes

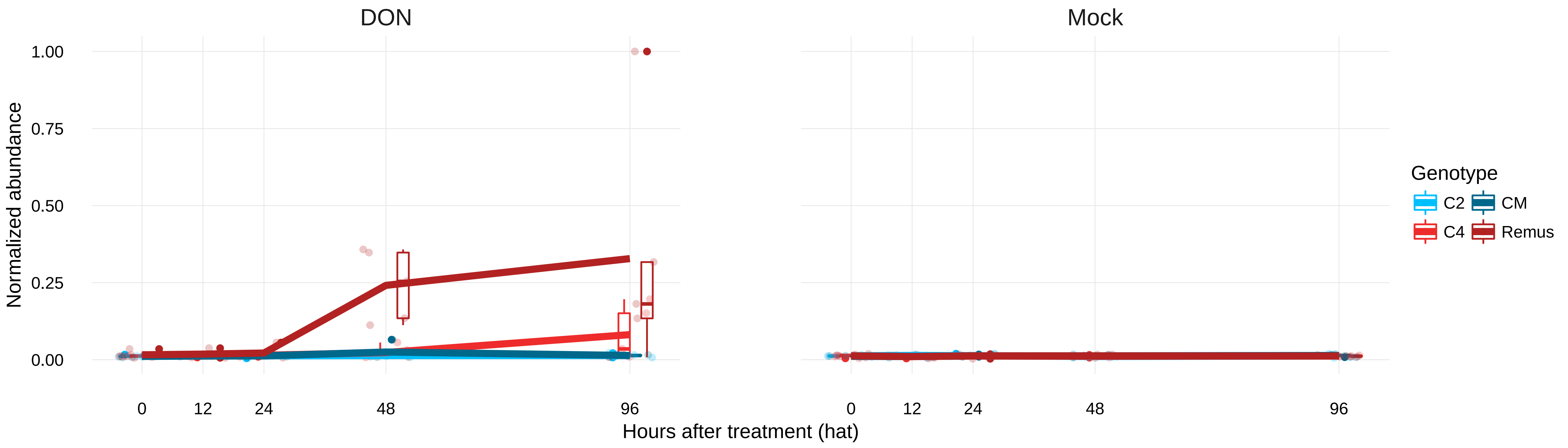

A.62

Annotated as Putative phenylacetic acid derivative
(8 database hits)

MZ	369.1182
RT	12.97 min
Normalization	Indirectly via surrogate in the KPX samples
Cluster	Cluster 2
Cn total / Phe	17 / 8

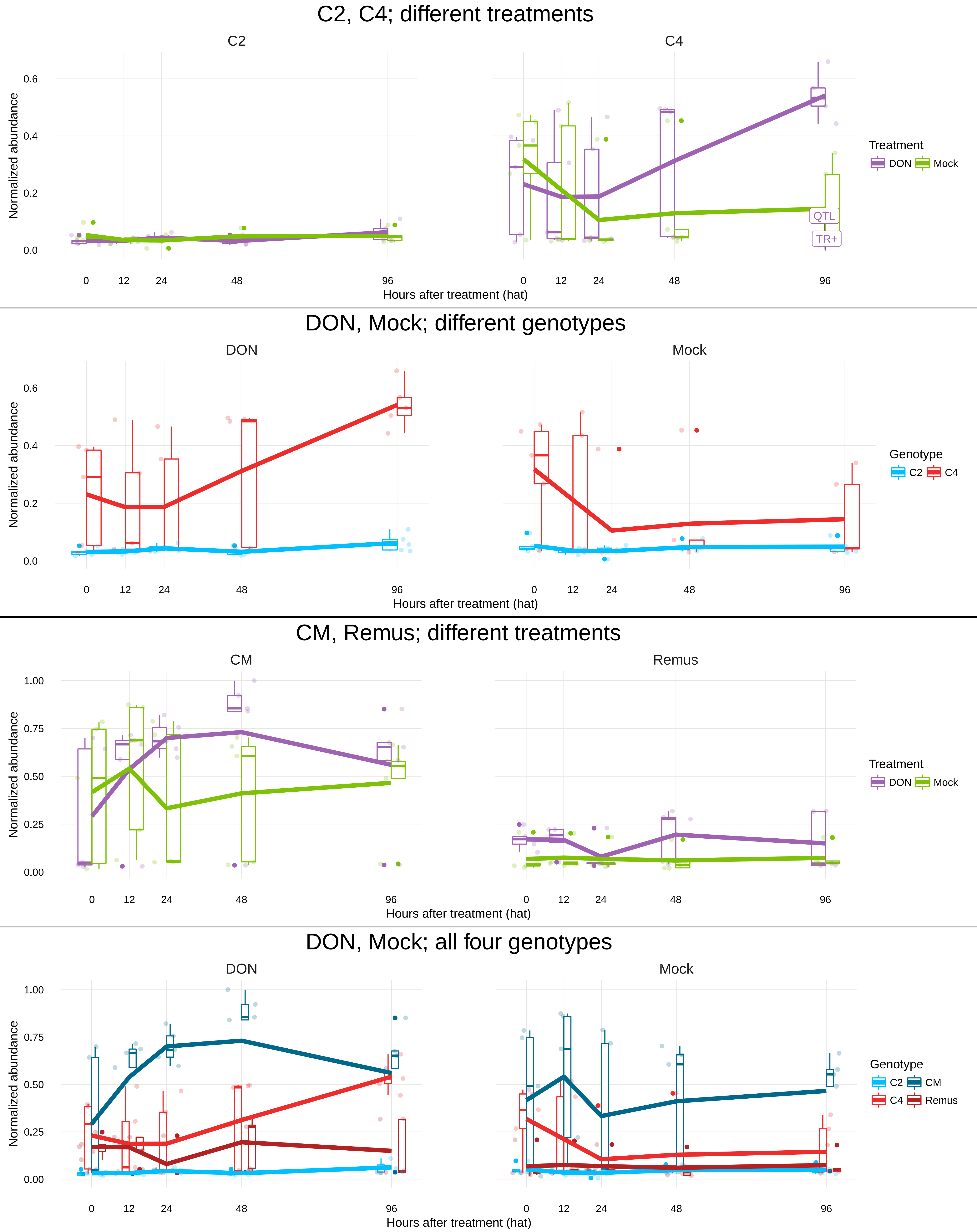

A.104

Annotated as Flavonoid (diGlcF)
(3 database hits)

MZ	565.1915
RT	17.01 min
Normalization	Indirectly via surrogate in the KPX samples
Cluster	Cluster 2
Cn total / Phe	27 / 9

C2, C4; different treatments

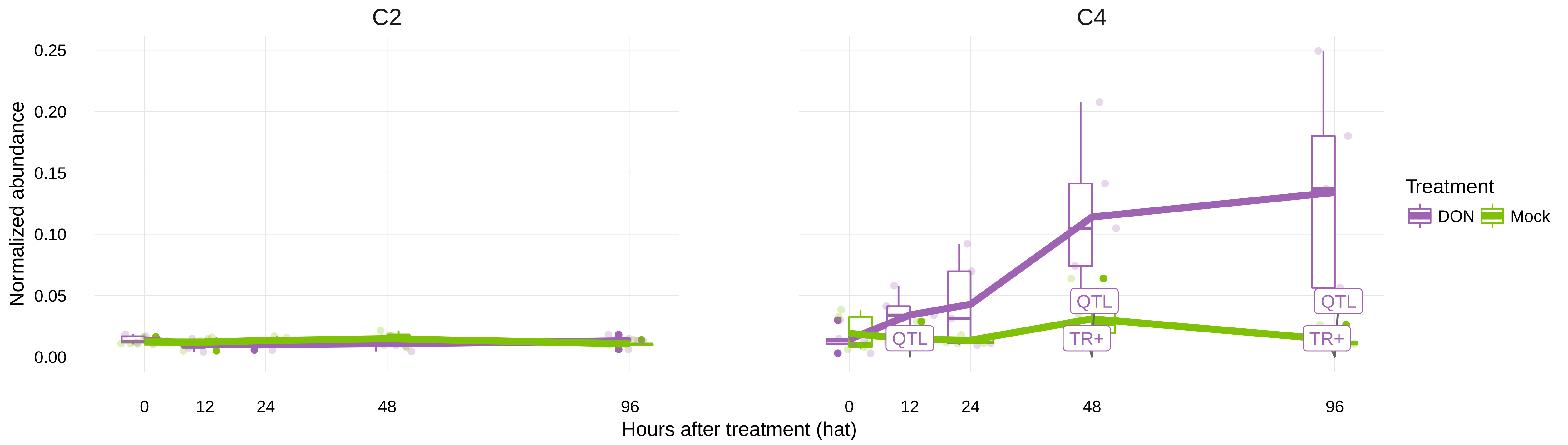

DON, Mock; different genotypes

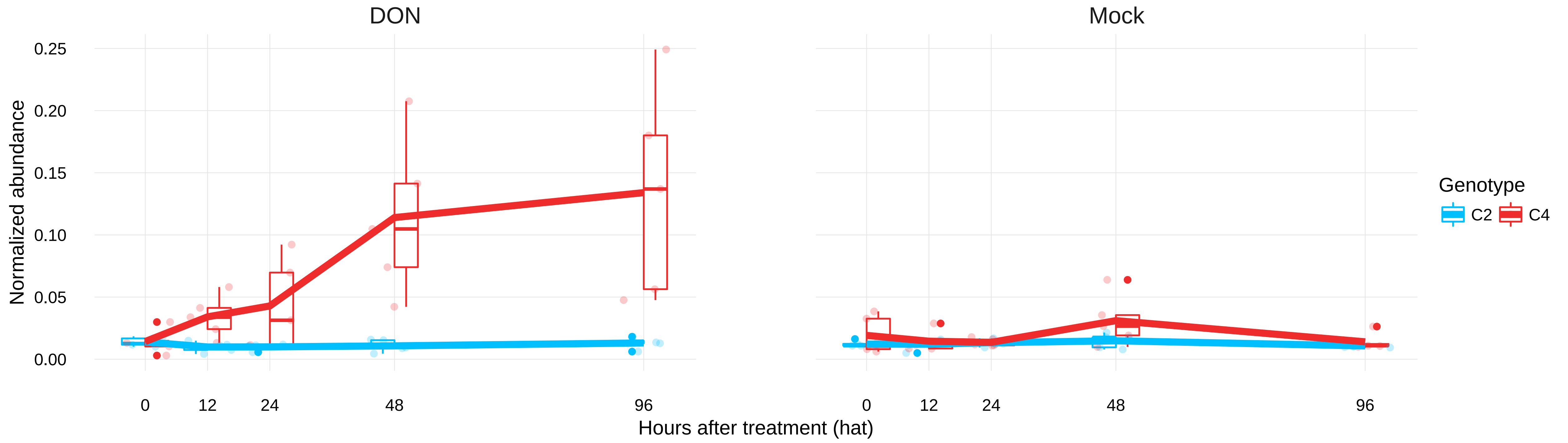

CM, Remus; different treatments

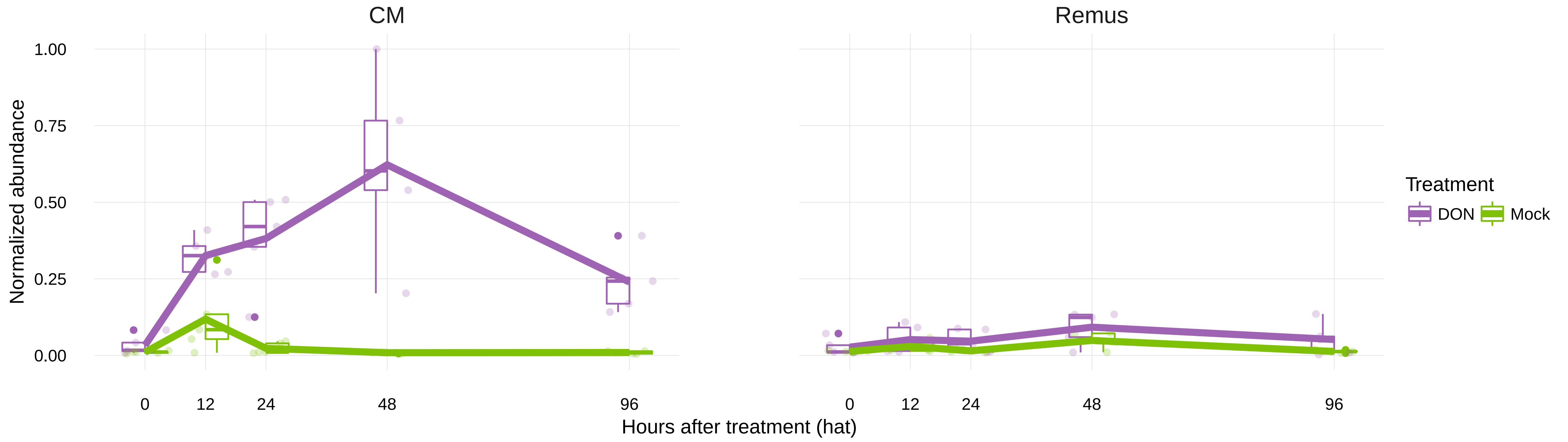

DON, Mock; all four genotypes

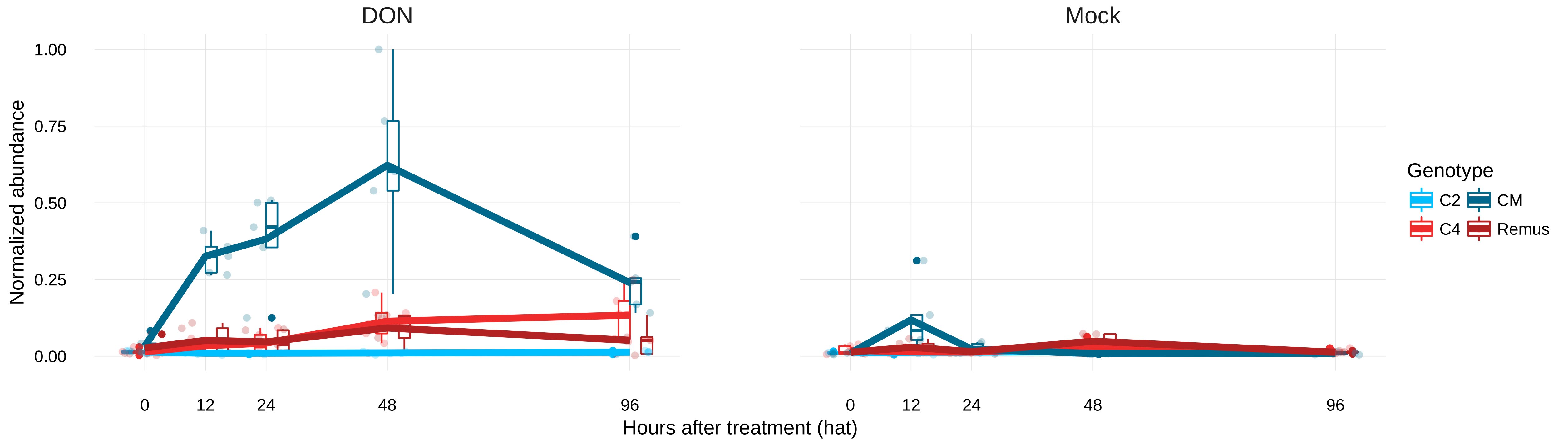

A.36

Annotated as HCA derivative
(1 database hit)

MZ	357.1183
RT	10.27 min
Normalization	Indirectly via surrogate in the KPX samples
Cluster	Cluster 2
Cn total / Phe	16 / 9

C2, C4; different treatments

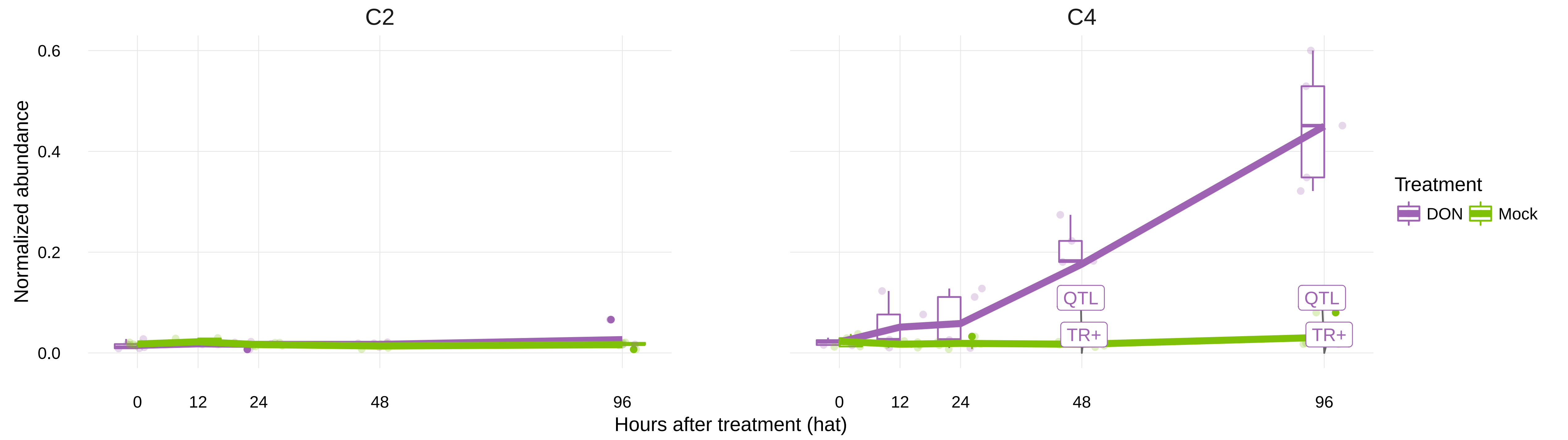

DON, Mock; different genotypes

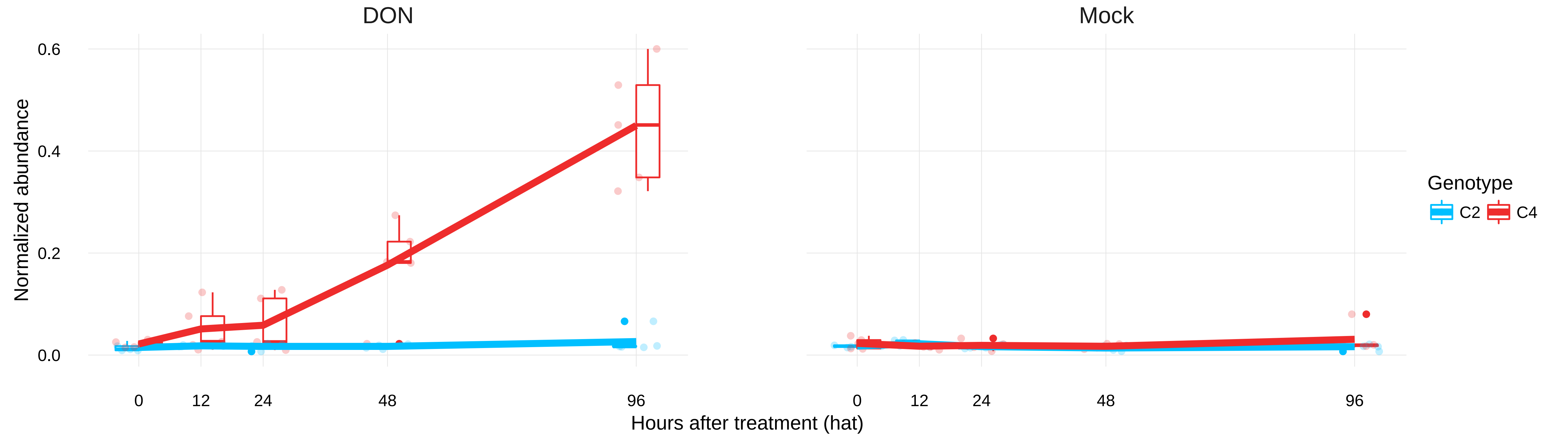

CM, Remus; different treatments

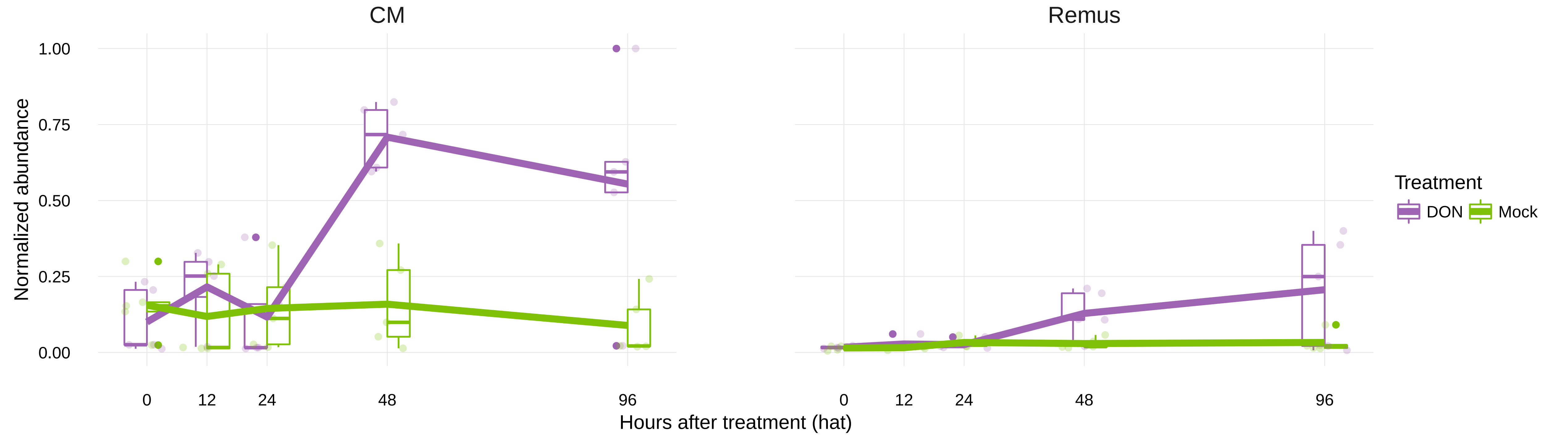

DON, Mock; all four genotypes

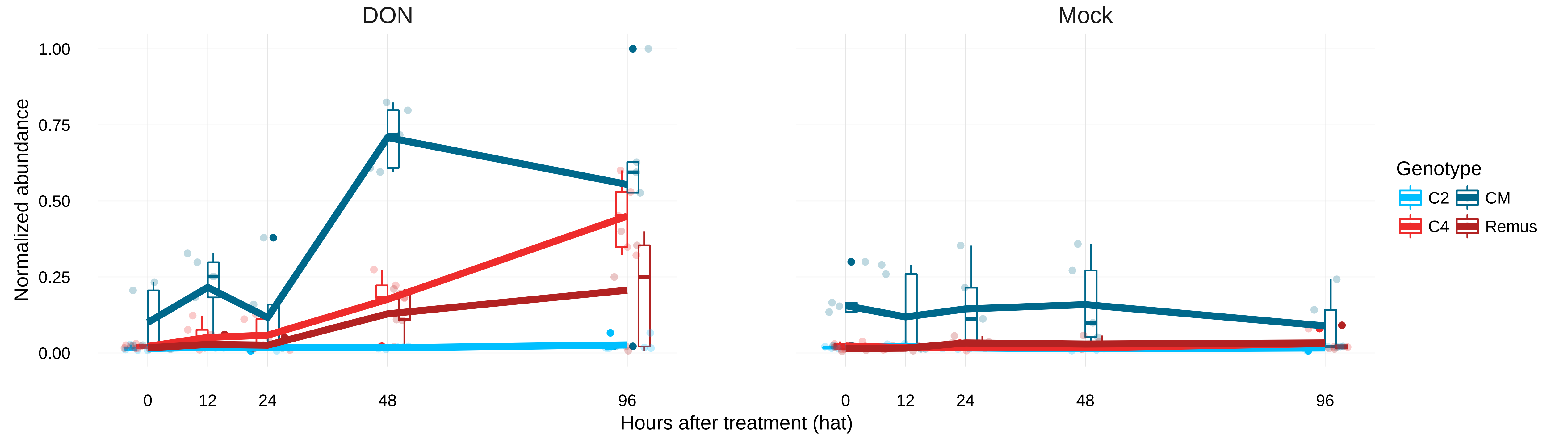

A.31

Annotated as HCA derivative
(3 database hits)

MZ	165.0549
RT	9.6 min
Normalization	Indirectly via surrogate in the KPX samples
Cluster	Cluster 2
Cn total / Phe	9 / 9

C2, C4; different treatments

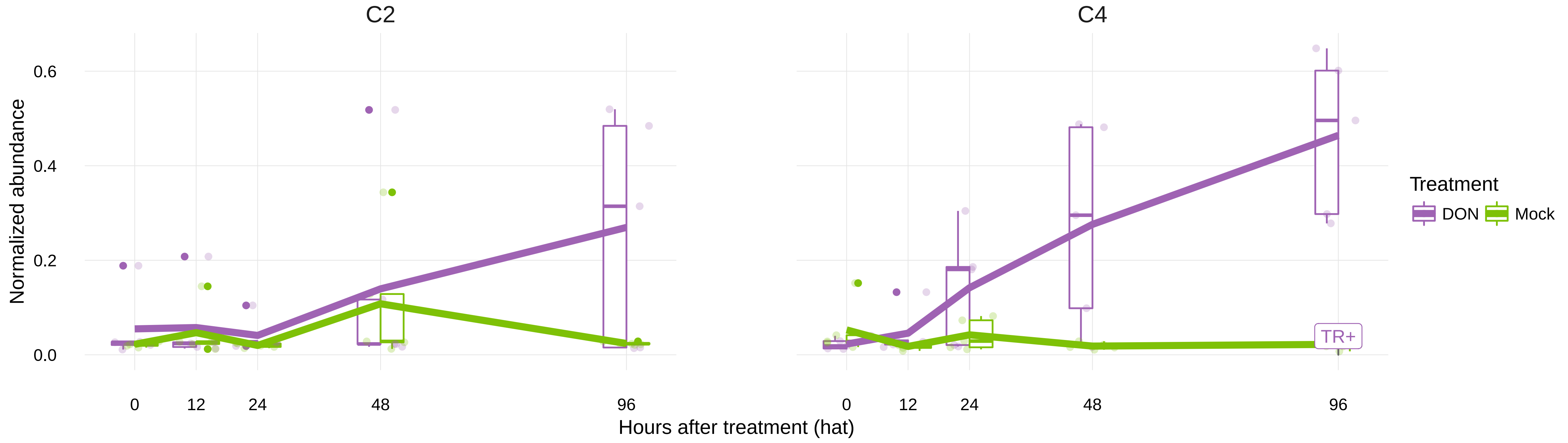

DON, Mock; different genotypes

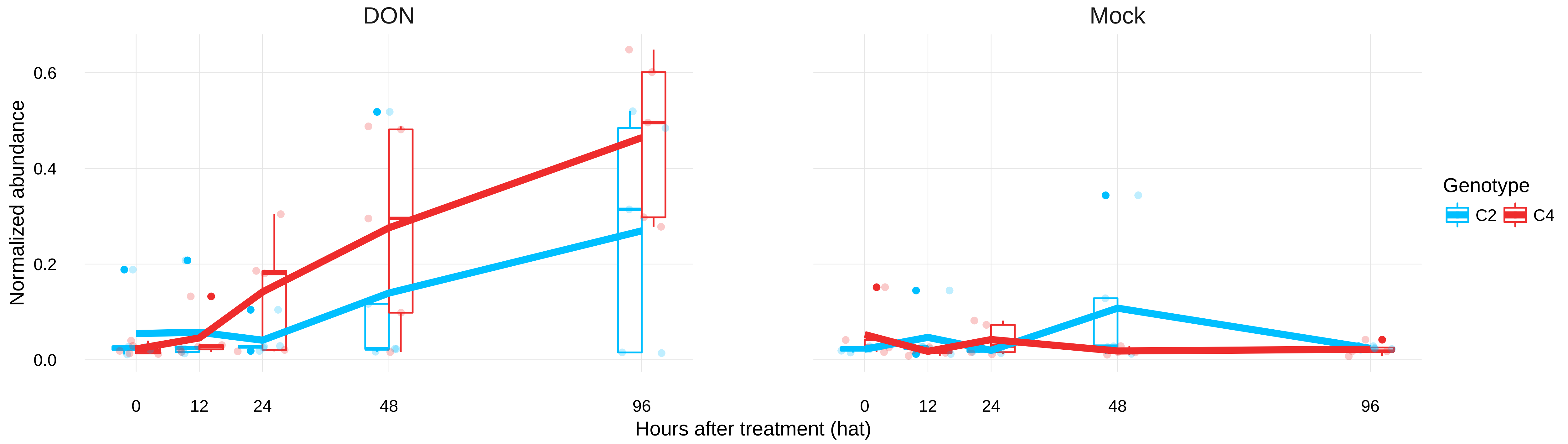

CM, Remus; different treatments

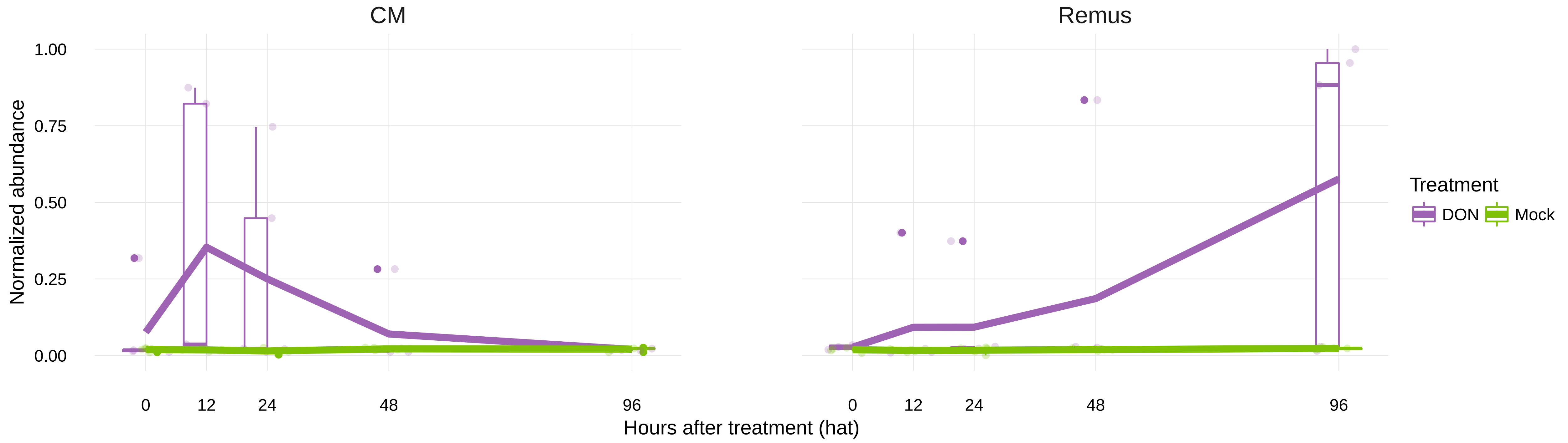

DON, Mock; all four genotypes

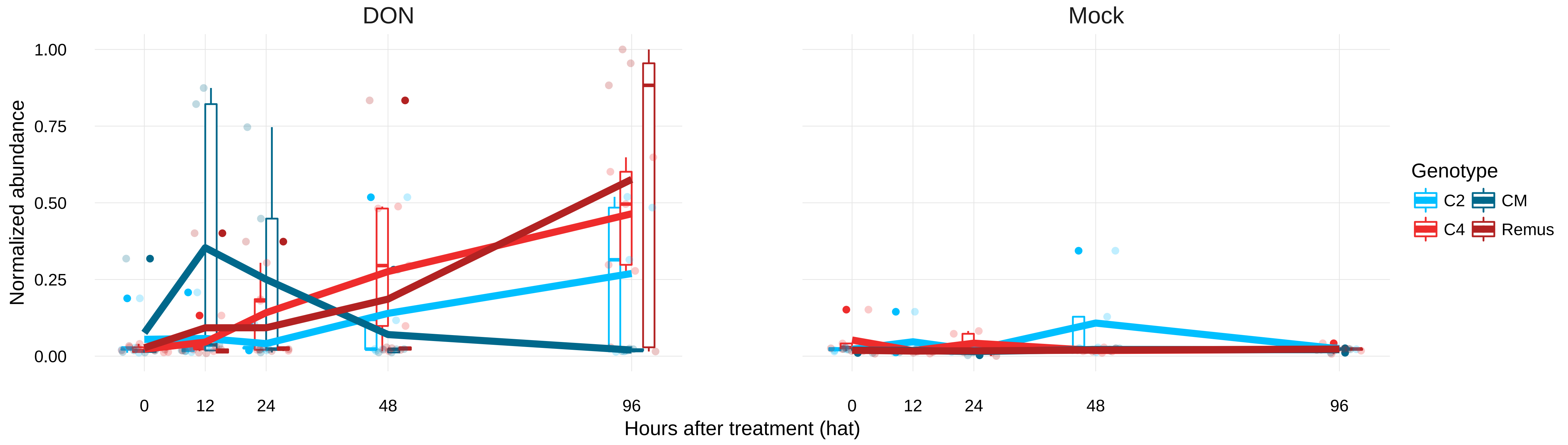

A.13

Annotated as HCA derivative
(1 database hit)

MZ	531.171
RT	6.42 min
Normalization	Indirectly via surrogate in the KPX samples
Cluster	Cluster 2
Cn total / Phe	23 / 9

C2, C4; different treatments

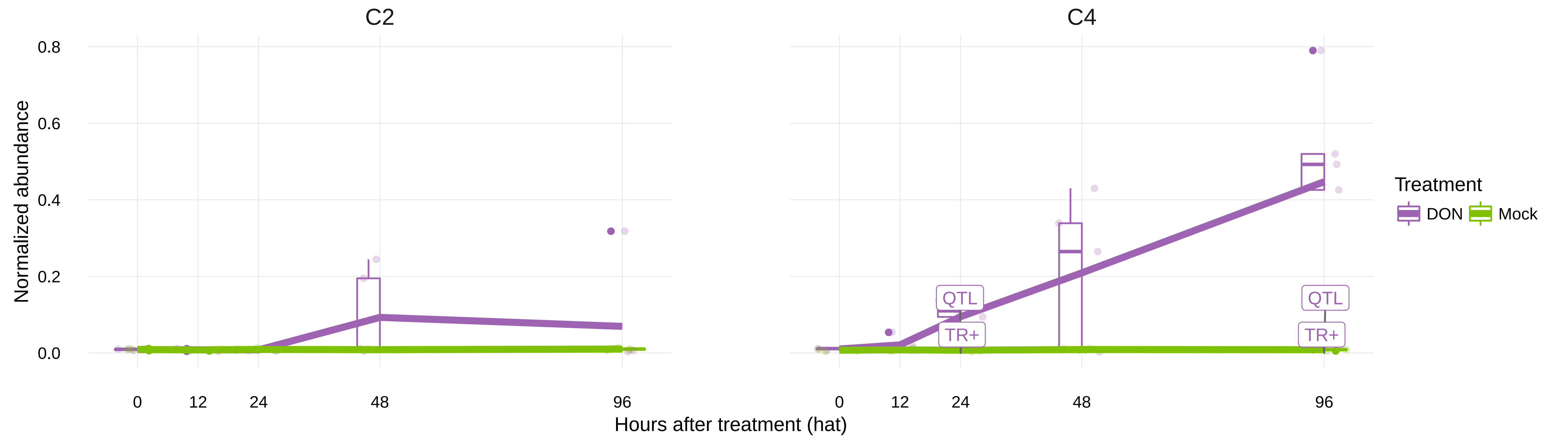

DON, Mock; different genotypes

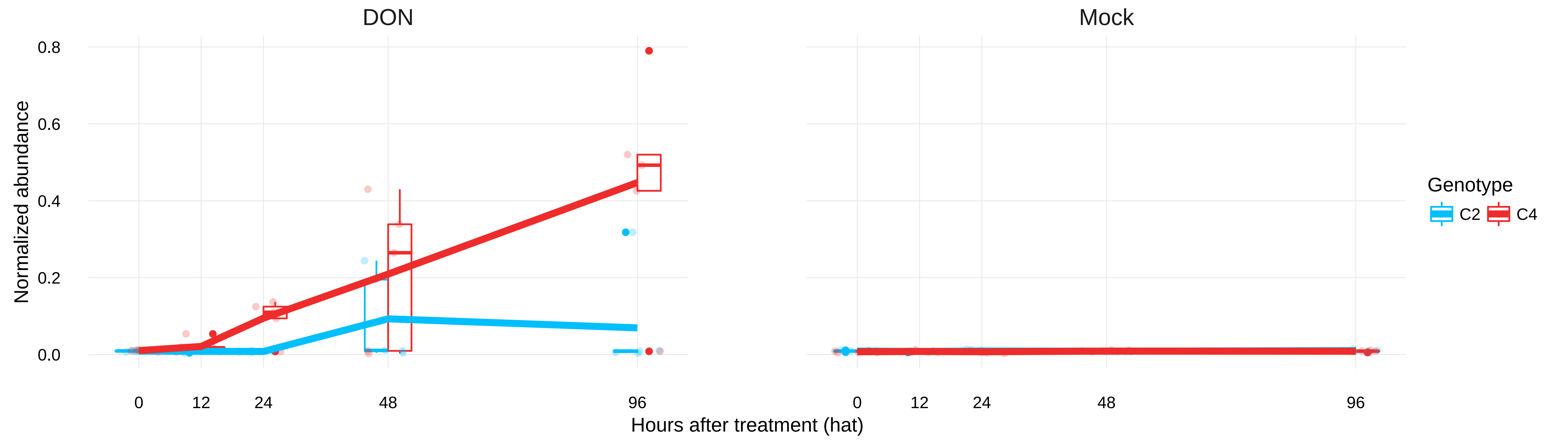

CM, Remus; different treatments

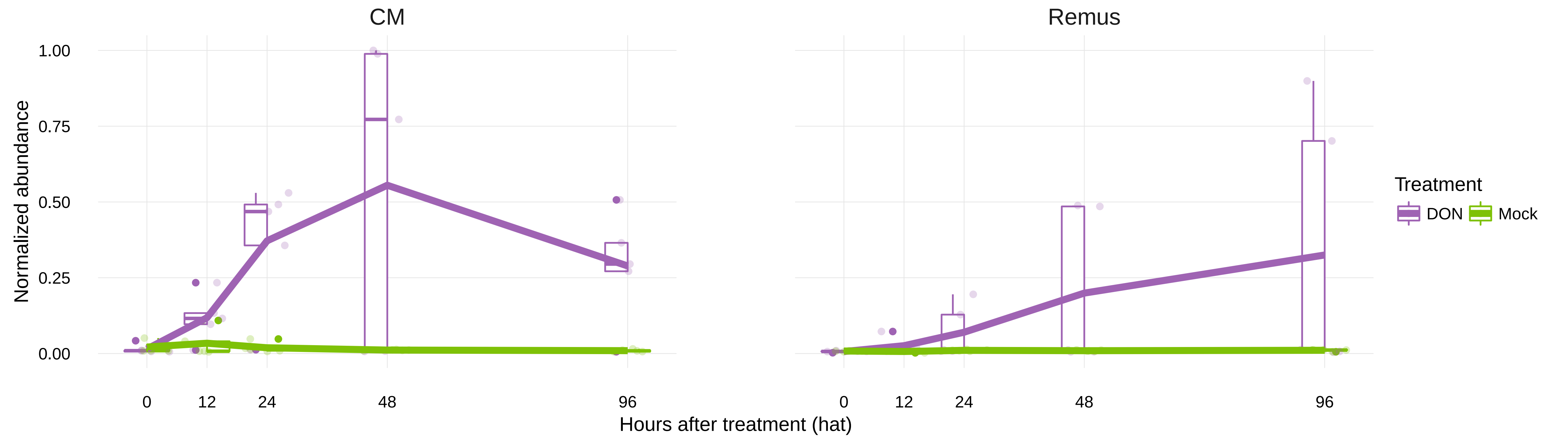

DON, Mock; all four genotypes

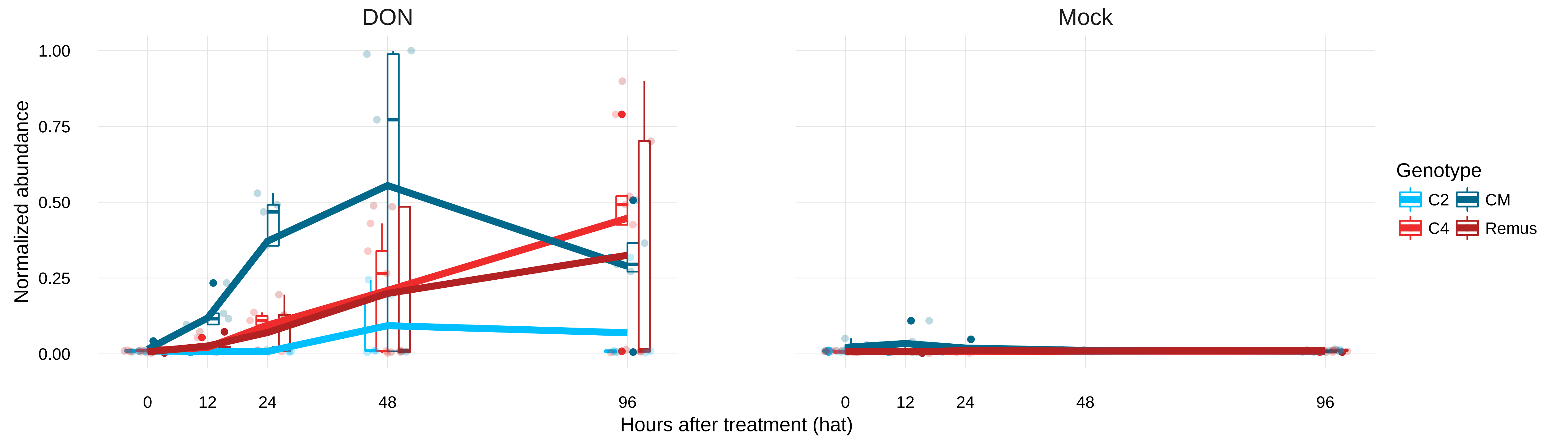

A.14

Annotated as HCAA
(2 database hits)

MZ	251.1393
RT	6.45 min
Normalization	Indirectly via surrogate in the KPX samples
Cluster	–
Cn total / Phe	13 / 9

C2, C4; different treatments

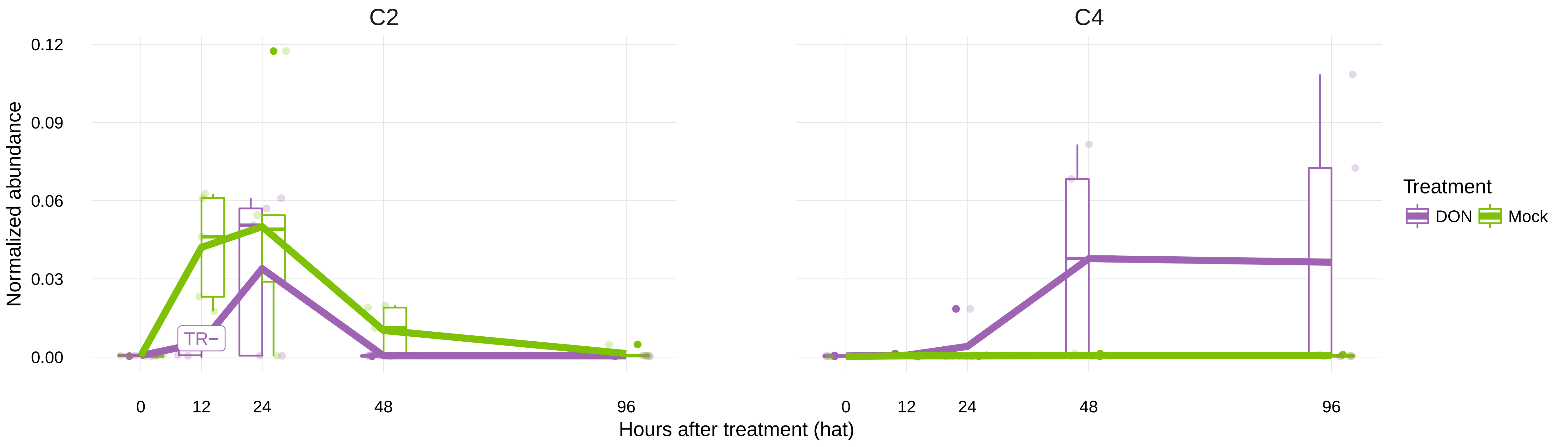

DON, Mock; different genotypes

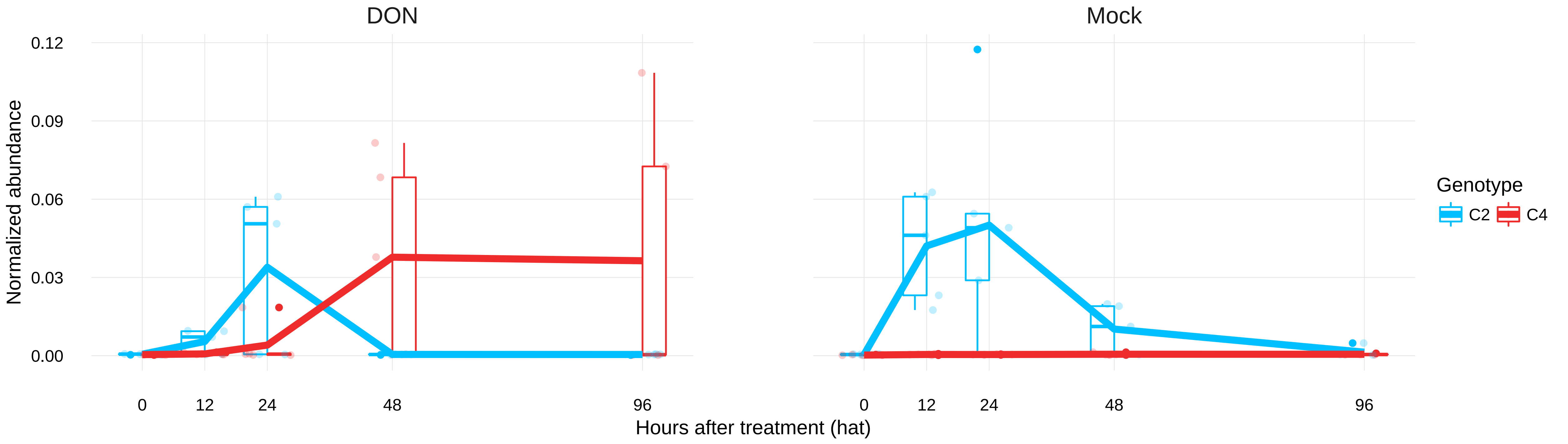

CM, Remus; different treatments

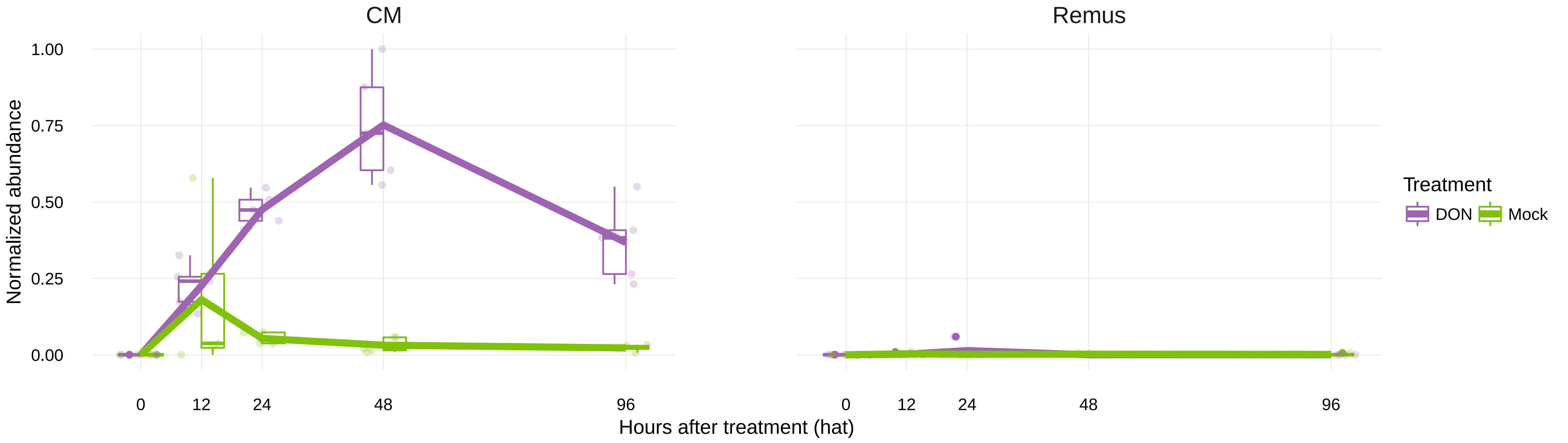

DON, Mock; all four genotypes

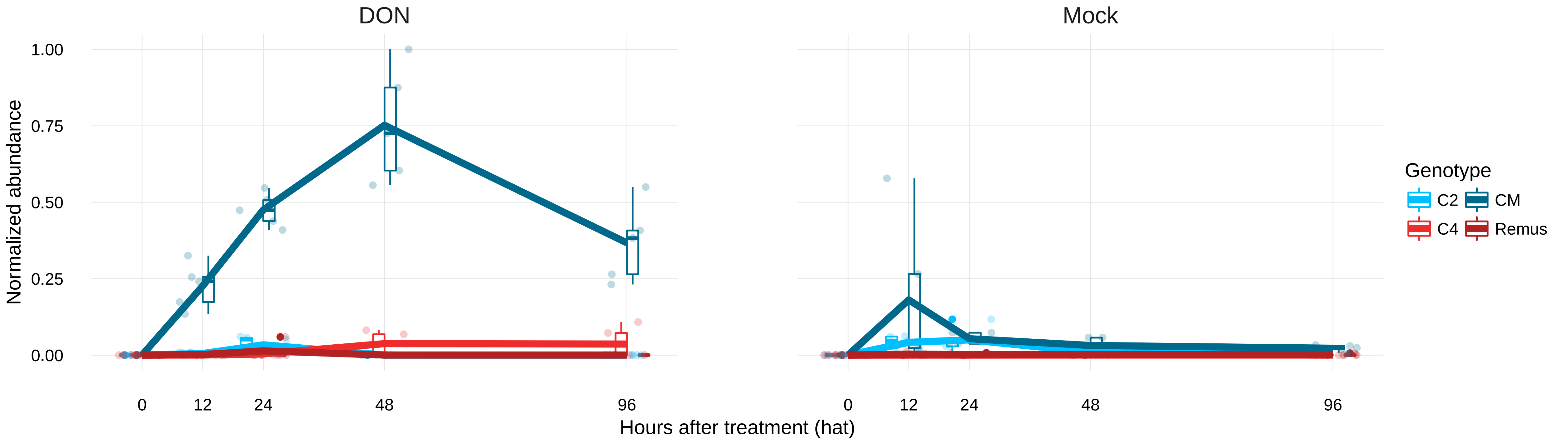

Sinapaldehyde

Identified metabolite (level 1)

MZ	209.0809
RT	14.46 min
Normalization	Directly via KPX samples
Cluster	–
Cn total / Phe	11 /

C2, C4; different treatments

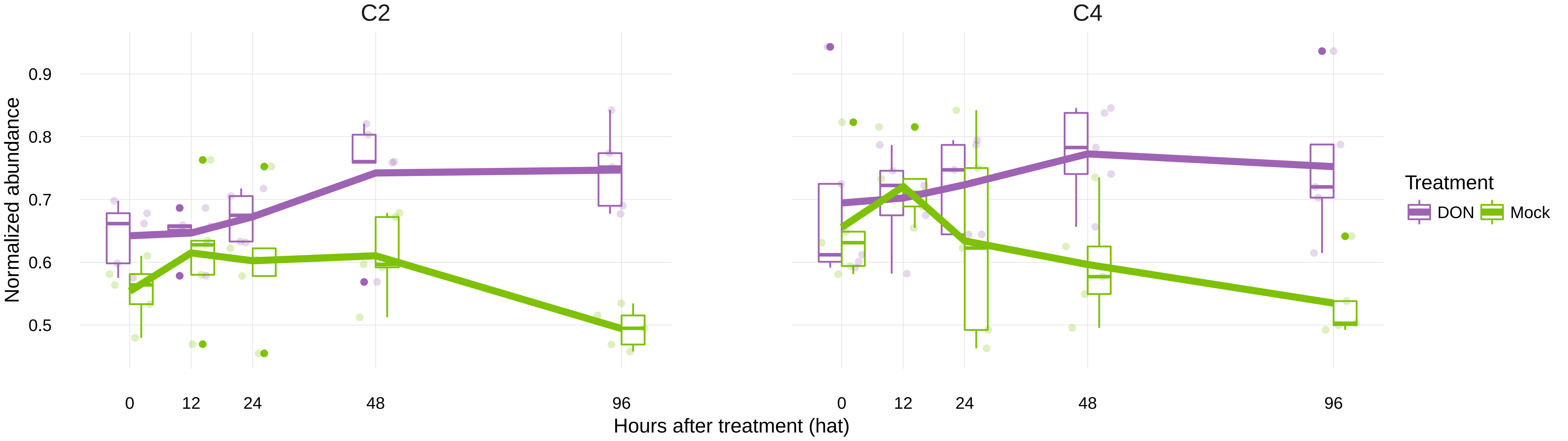

DON, Mock; different genotypes

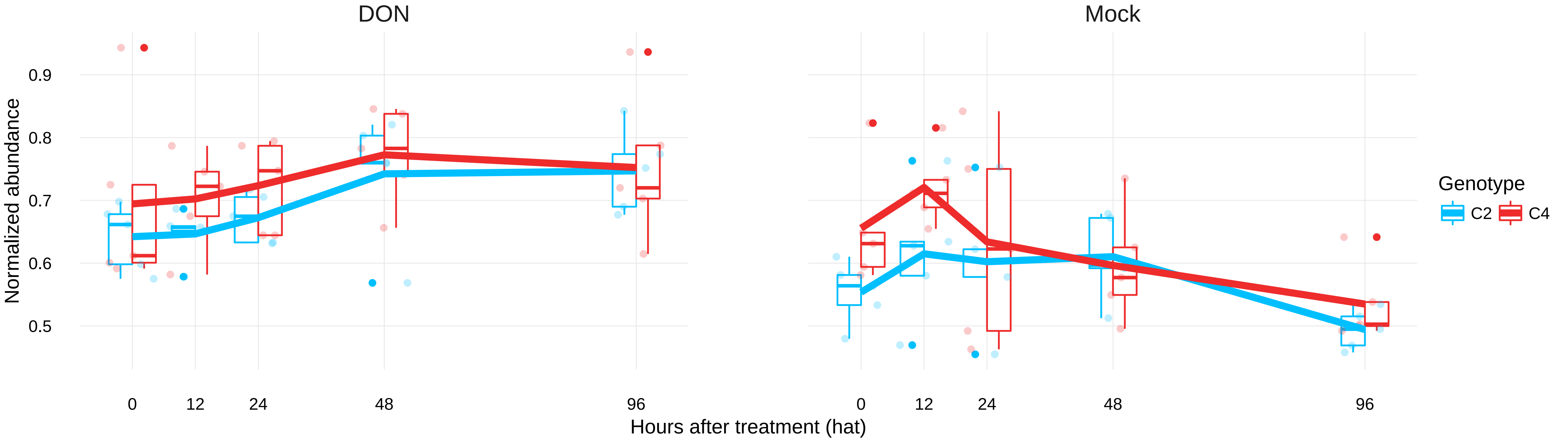

CM, Remus; different treatments

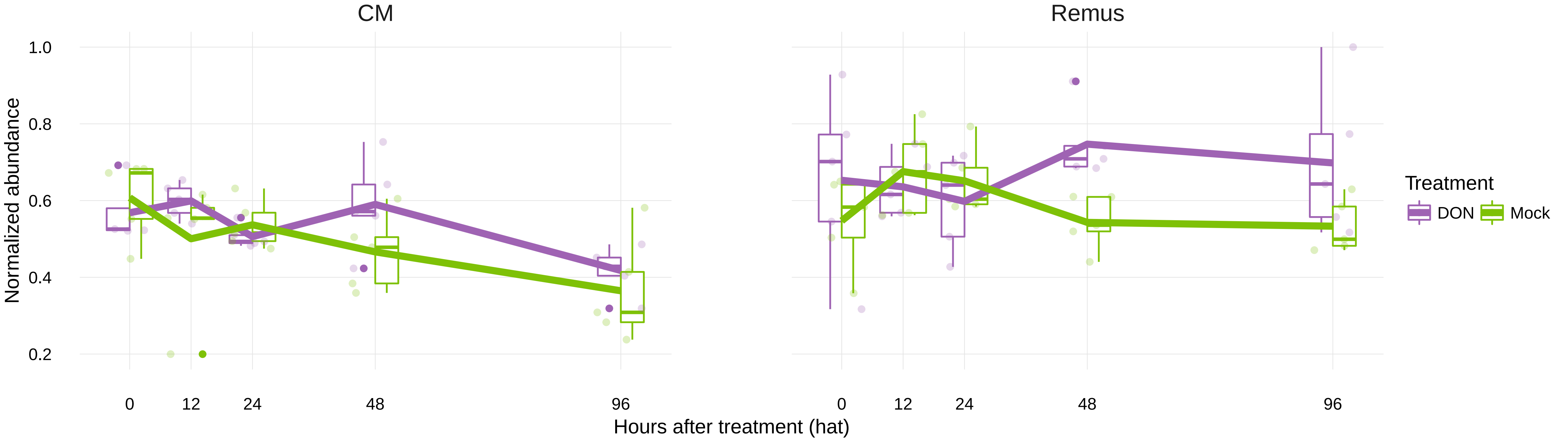

DON, Mock; all four genotypes

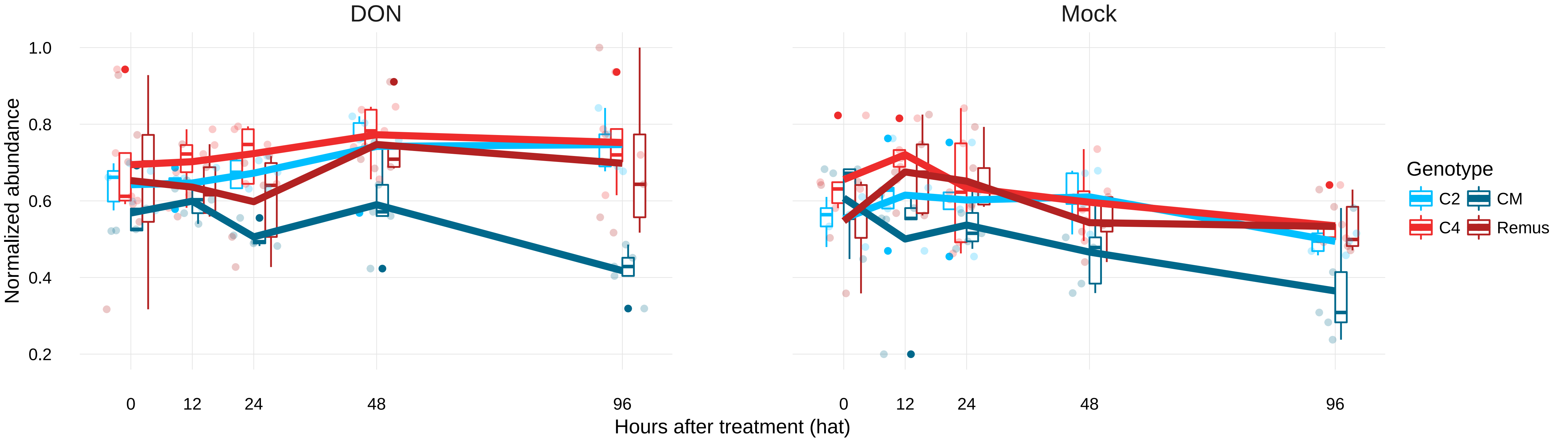

A.64

Annotated as Putative phenylacetic acid derivative
(1 database hit)

MZ	409.1107
RT	12.98 min
Normalization	Indirectly via surrogate in the KPX samples
Cluster	–
Cn total / Phe	17 / 8

C2, C4; different treatments

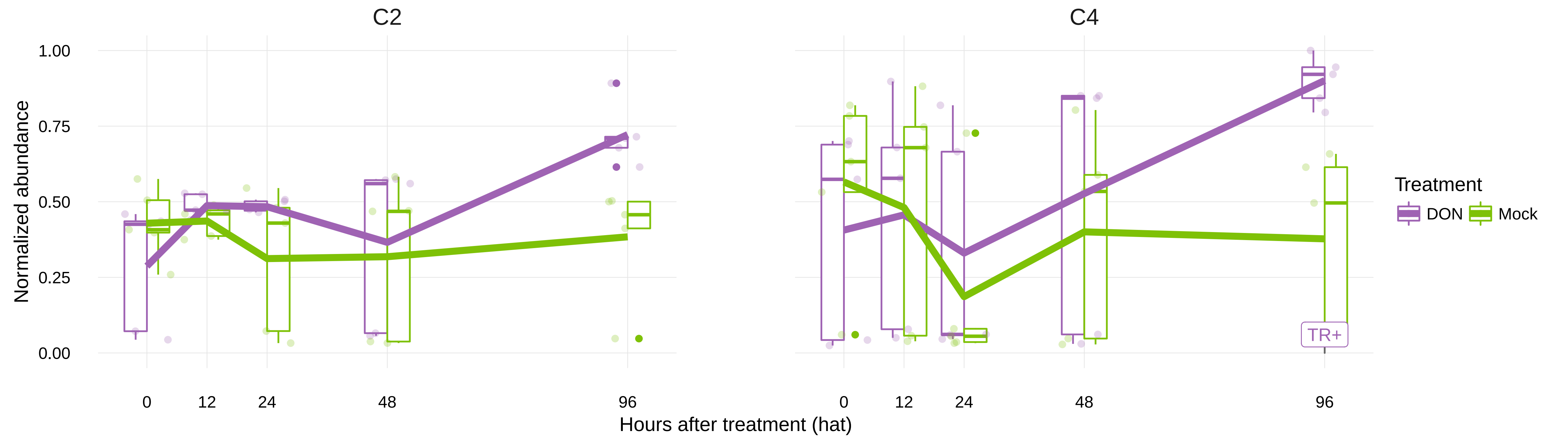

DON, Mock; different genotypes

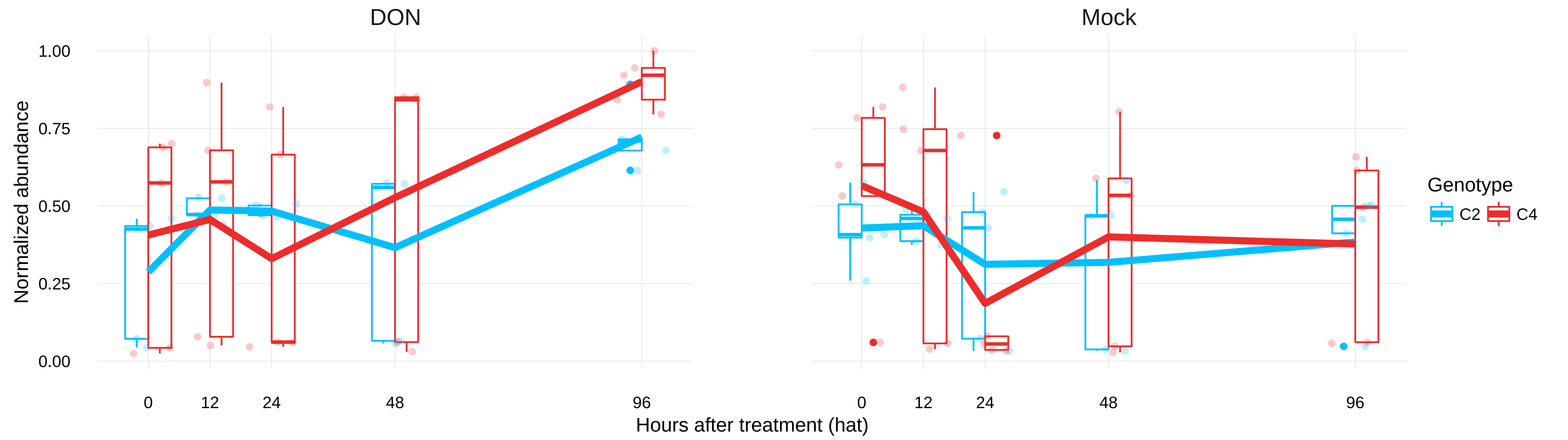

CM, Remus; different treatments

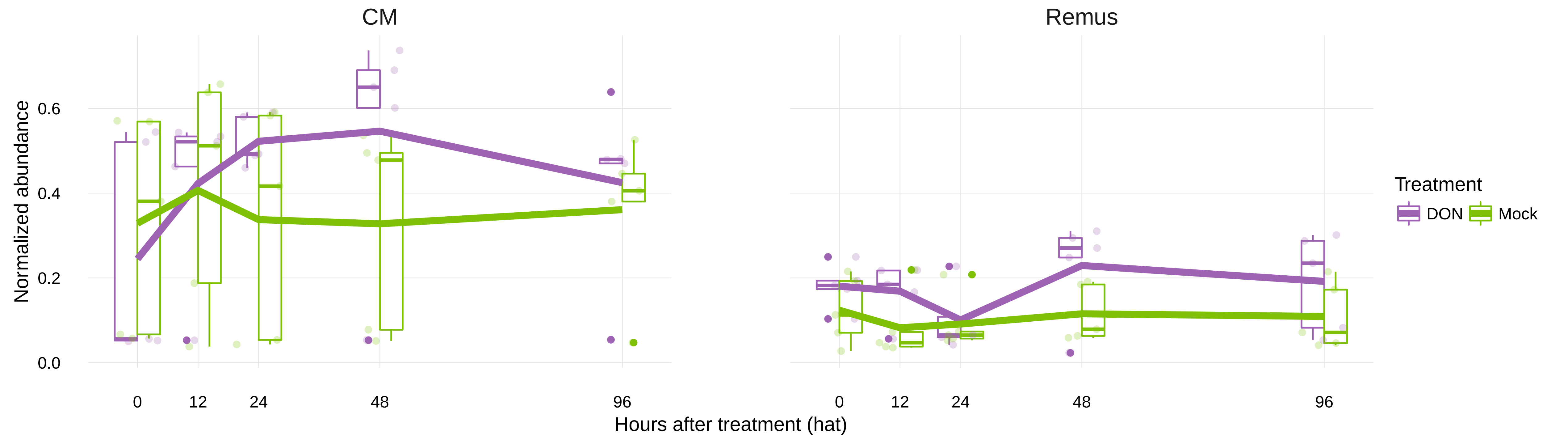

DON, Mock; all four genotypes

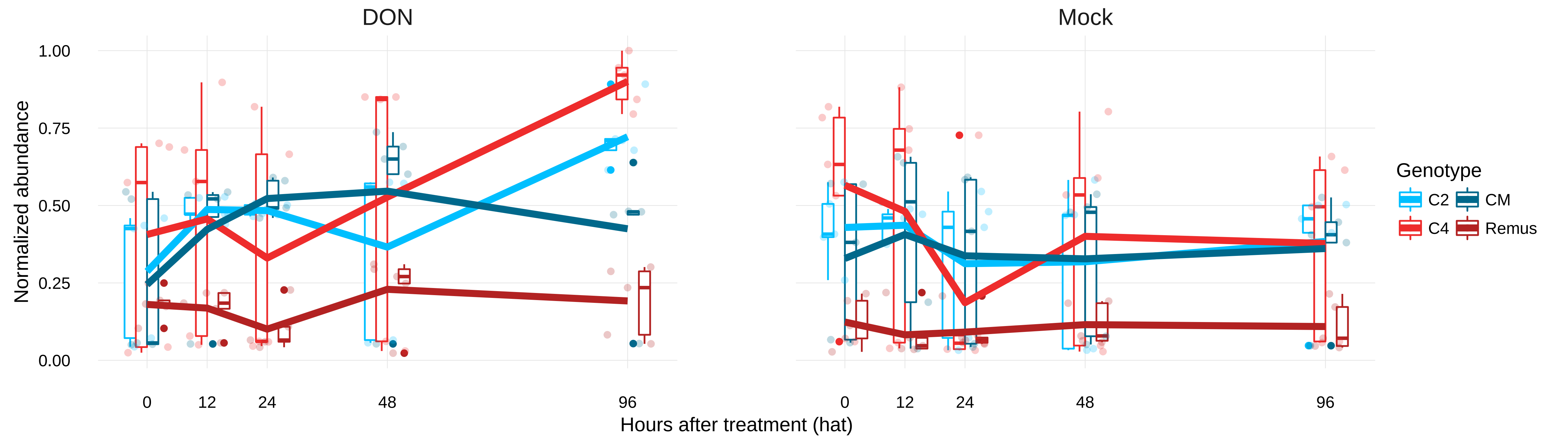

Sinapyl alcohol

Identified metabolite (level 1)

MZ	211.0966
RT	12.97 min
Normalization	Directly via KPX samples
Cluster	–
Cn total / Phe	11 /

C2, C4; different treatments

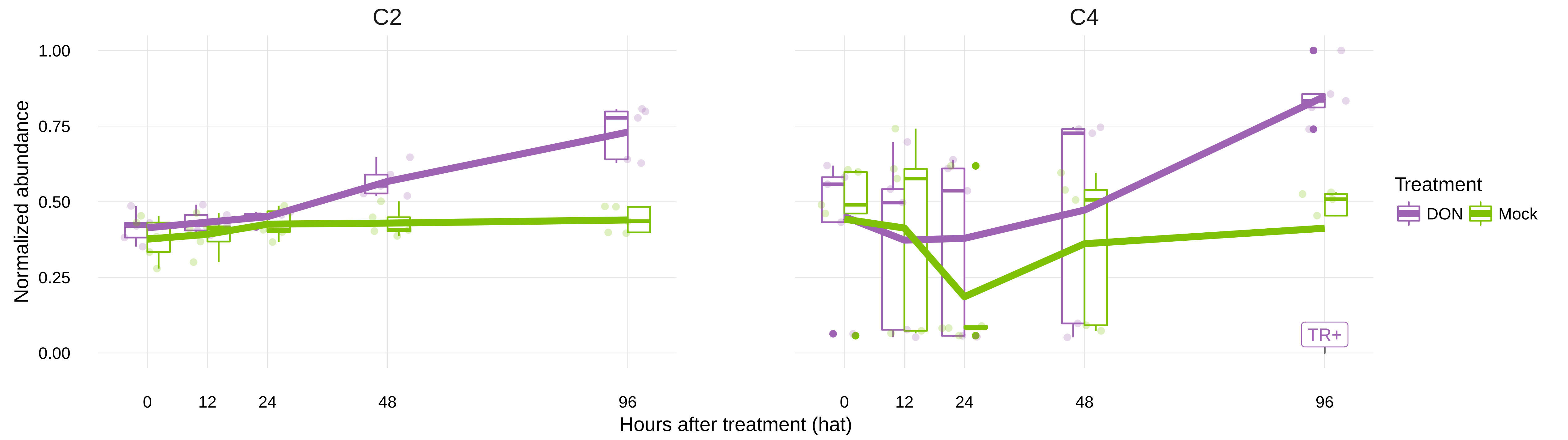

DON, Mock; different genotypes

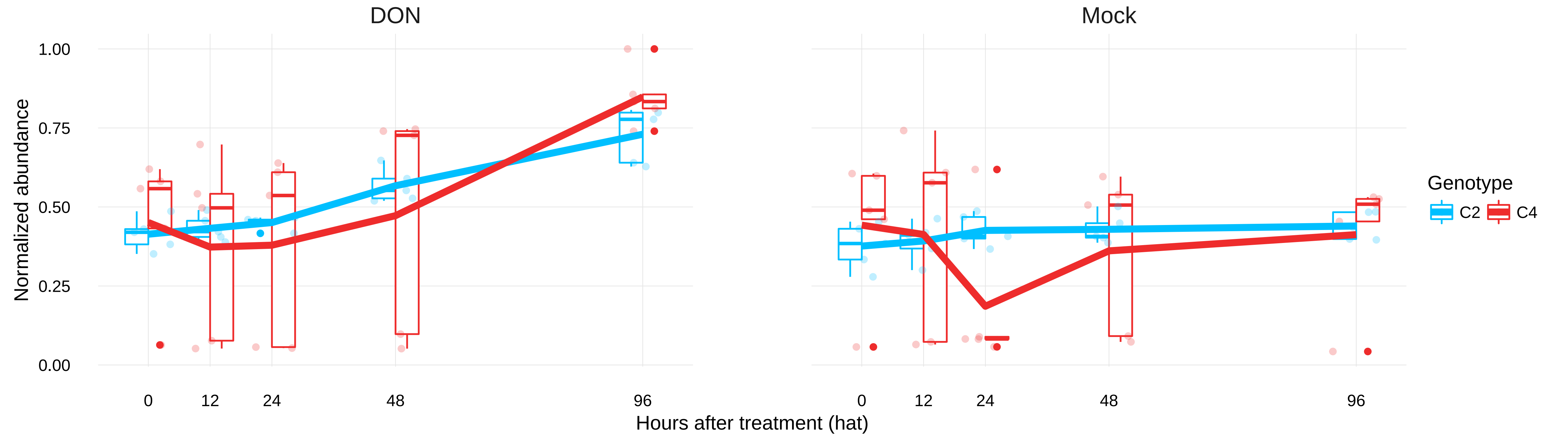

CM, Remus; different treatments

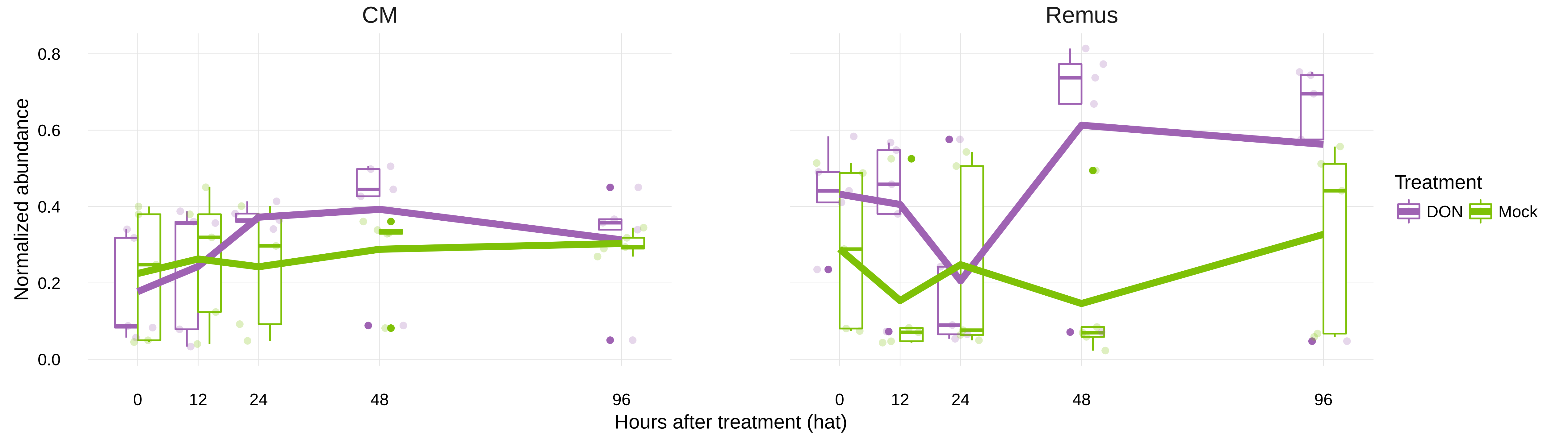

DON, Mock; all four genotypes

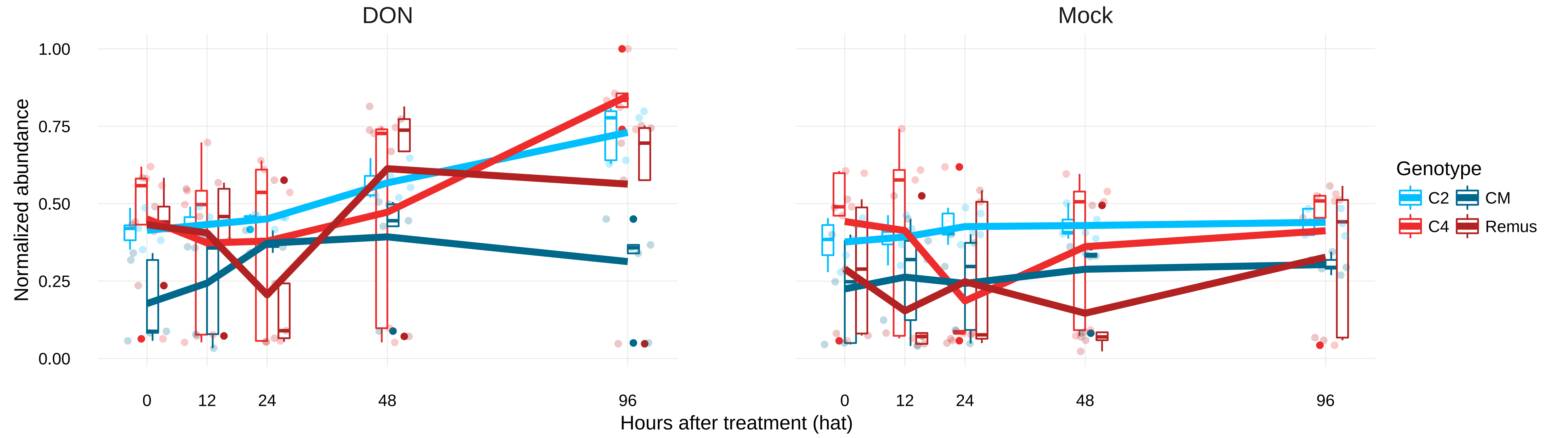

Phenylalanine

Identified metabolite (level 1)

MZ	166.0864
RT	4.46 min
Normalization	Not normalized
Cluster	–
Cn total / Phe	9 / 9

C2, C4; different treatments

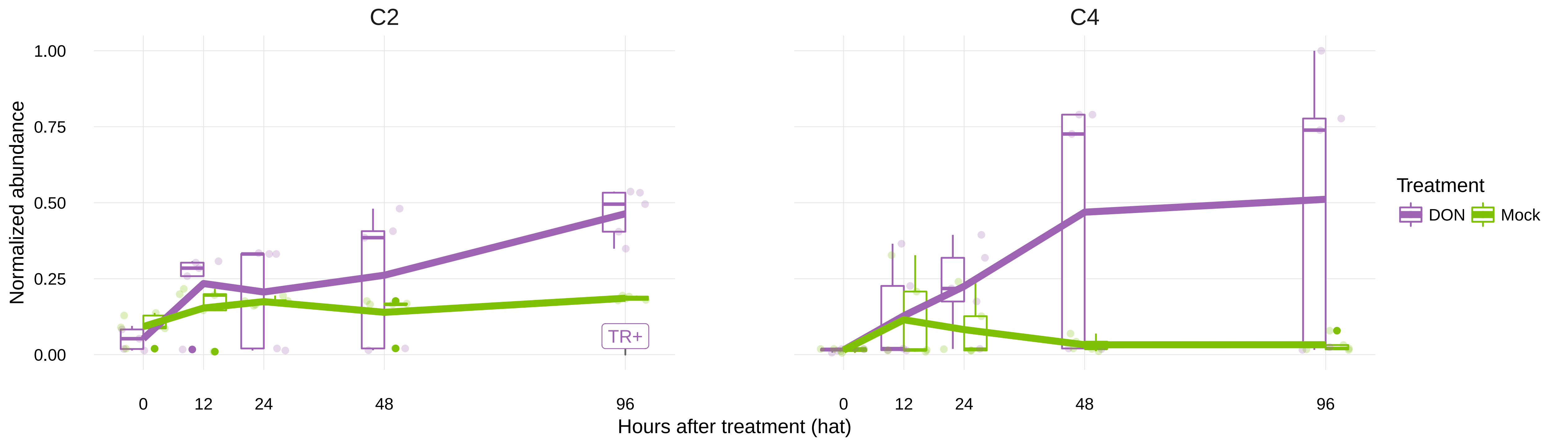

DON, Mock; different genotypes

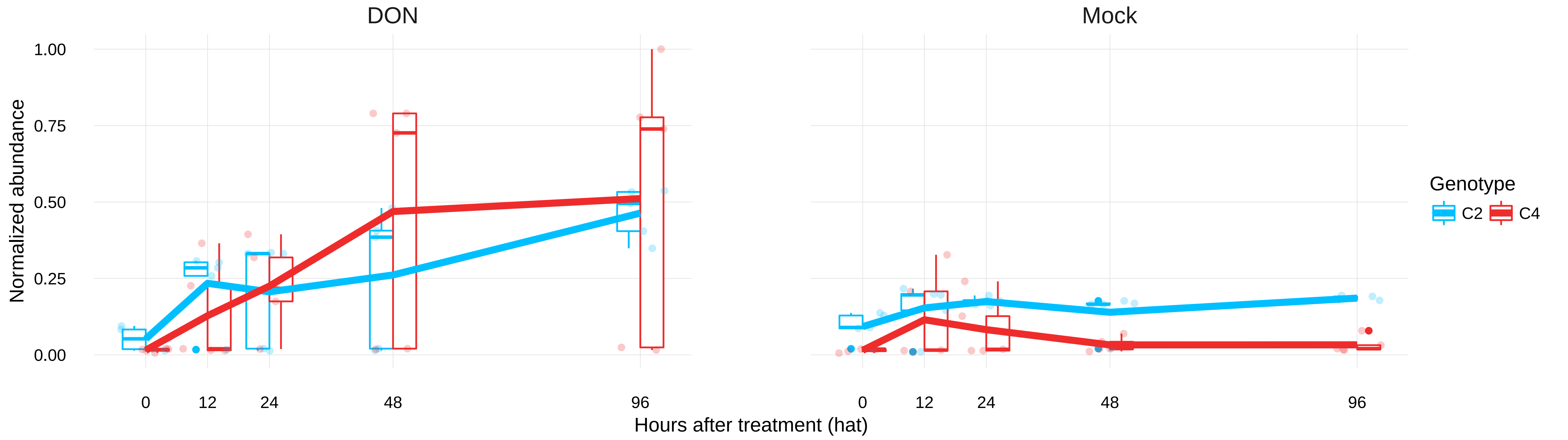

CM, Remus; different treatments

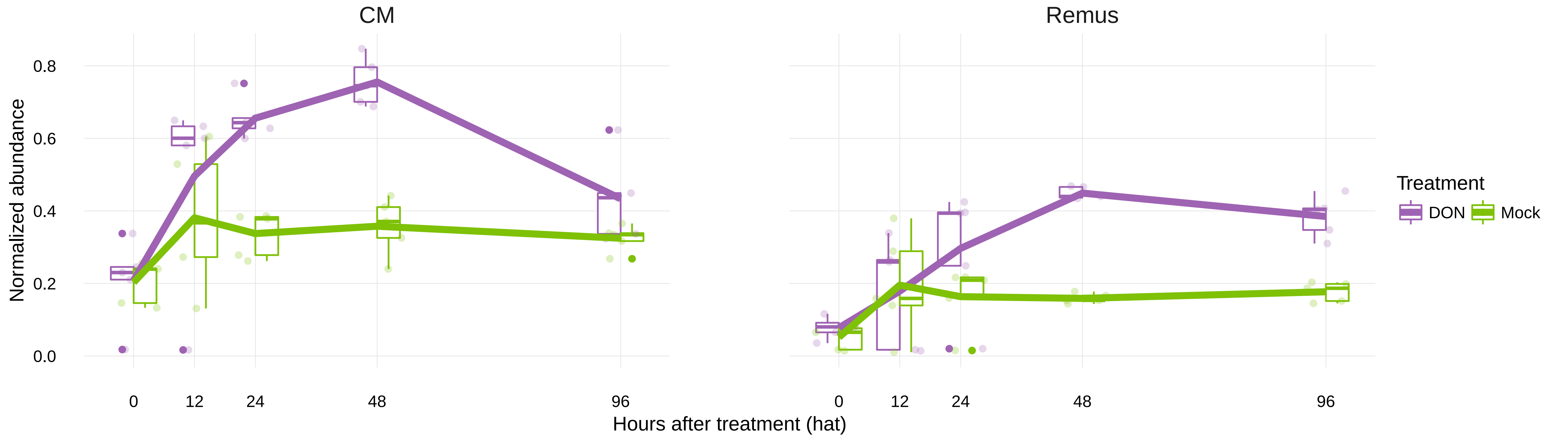

DON, Mock; all four genotypes

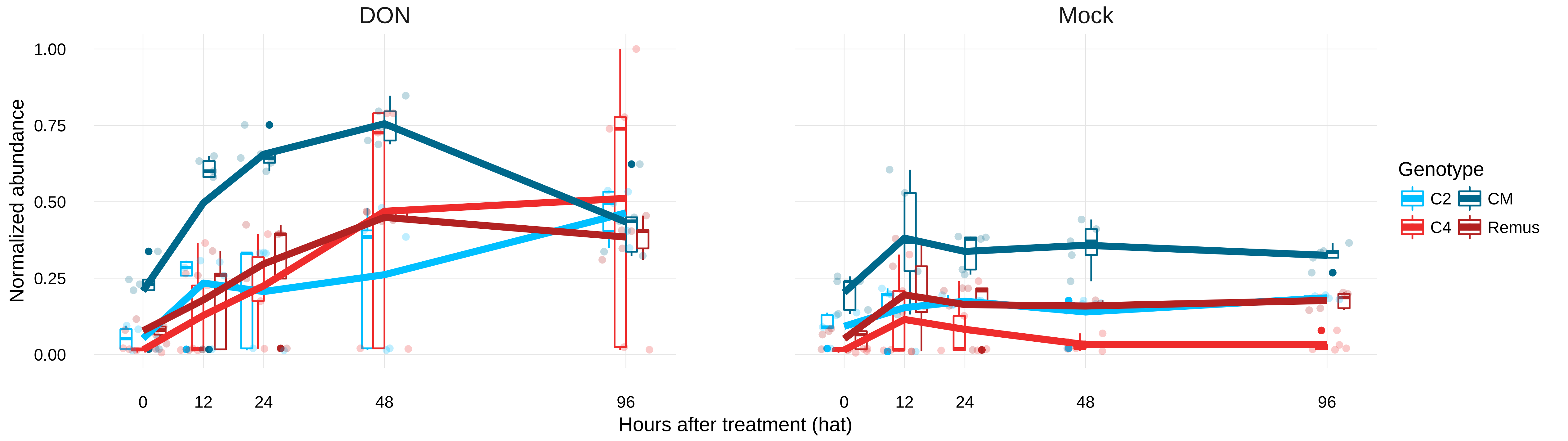

A.76

Annotated as Putative benzoic acid derivative
(10 database hits)

MZ	374.1448
RT	14 min
Normalization	Indirectly via surrogate in the KPX samples
Cluster	–
Cn total / Phe	16 / 7

C2, C4; different treatments

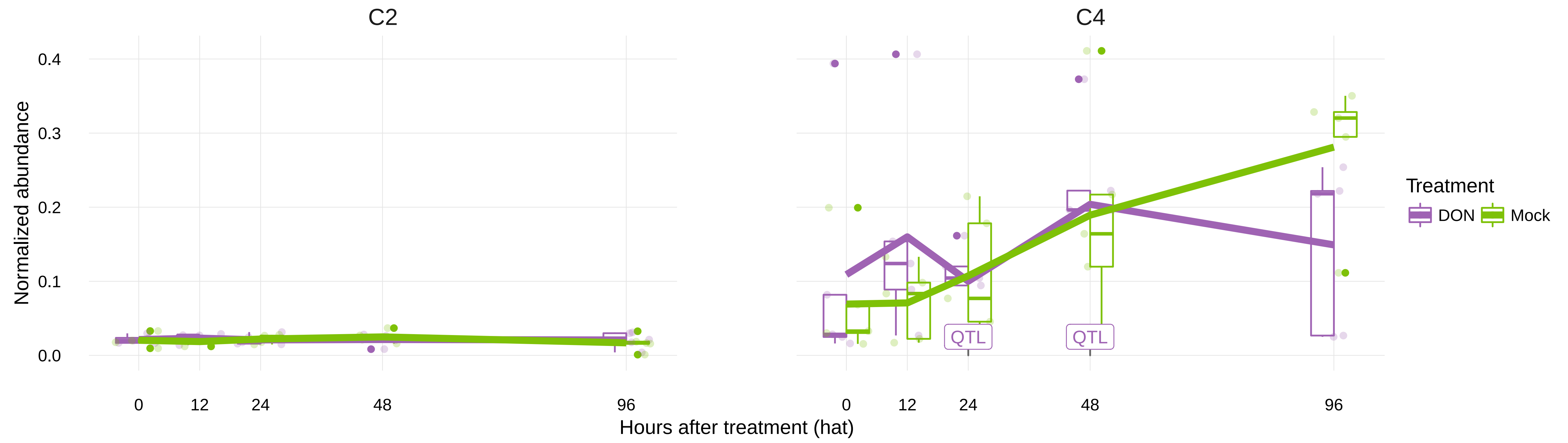

DON, Mock; different genotypes

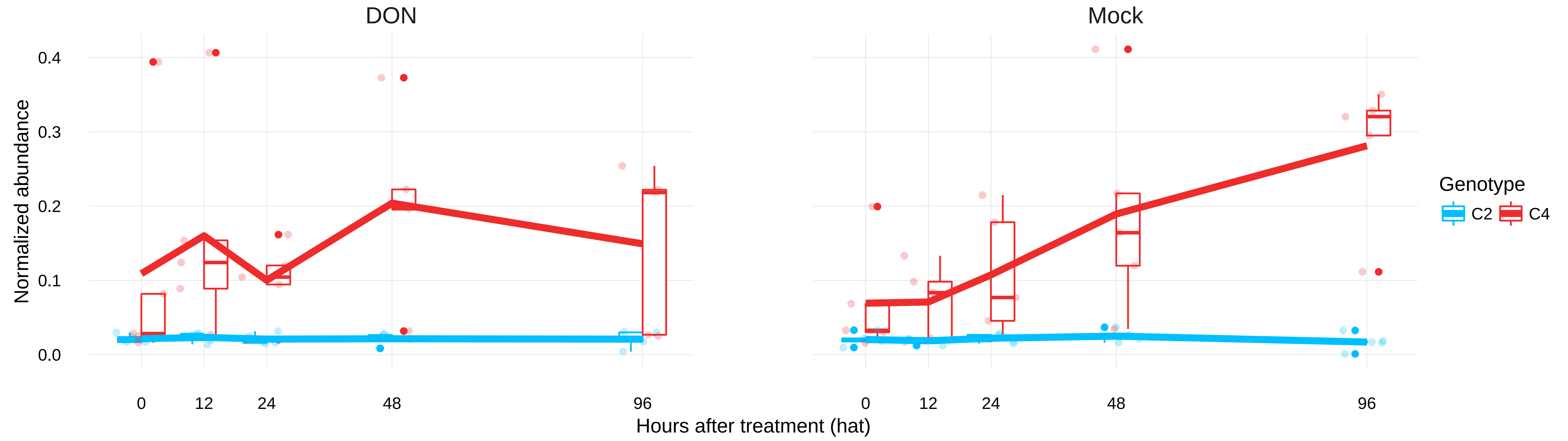

CM, Remus; different treatments

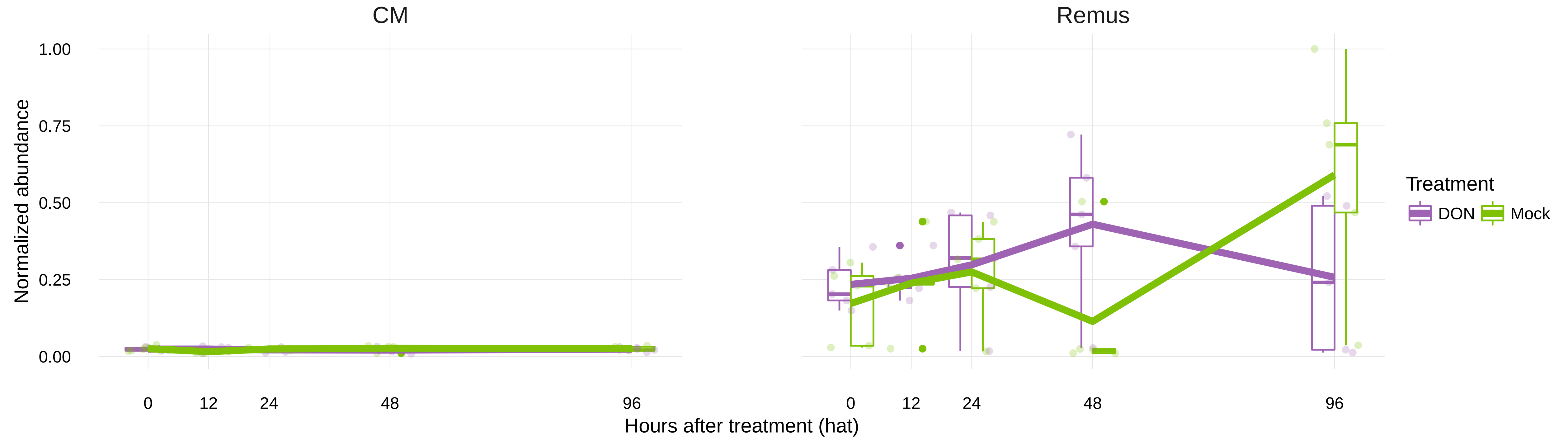

DON, Mock; all four genotypes

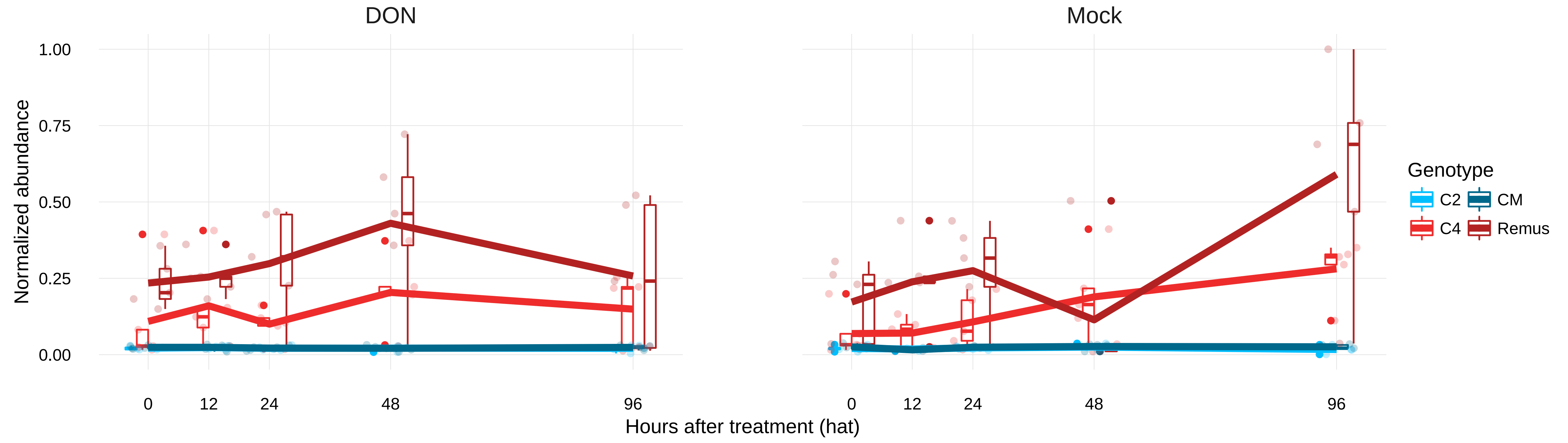

A.27

Annotated as HCA derivative
(1 database hit)

MZ	395.1315
RT	9.19 min
Normalization	Directly via KPX samples
Cluster	–
Cn total / Phe	17 /

C2, C4; different treatments

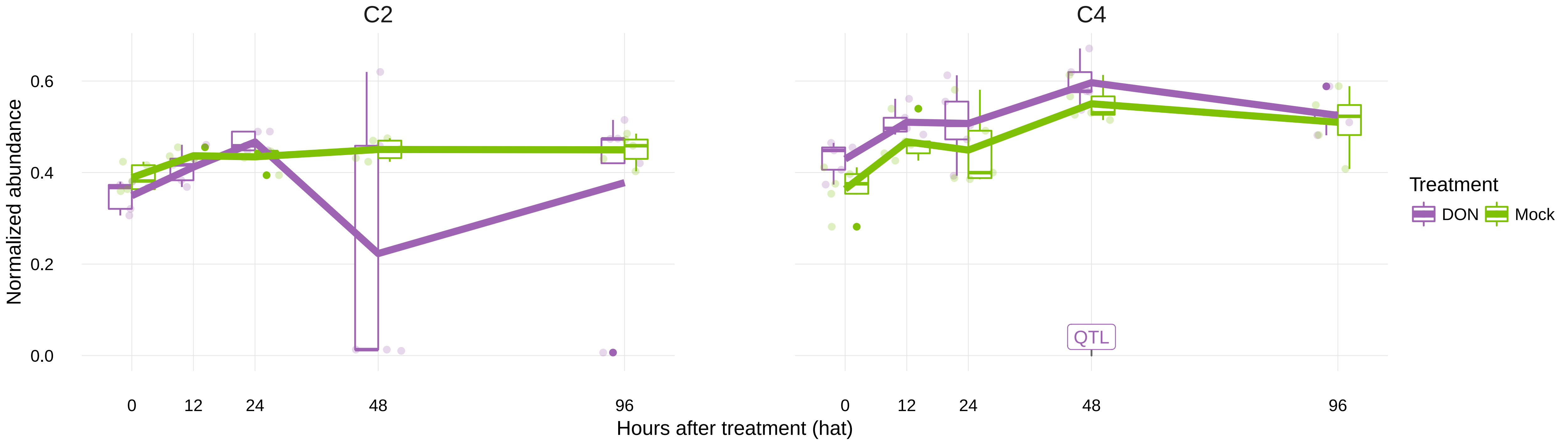

DON, Mock; different genotypes

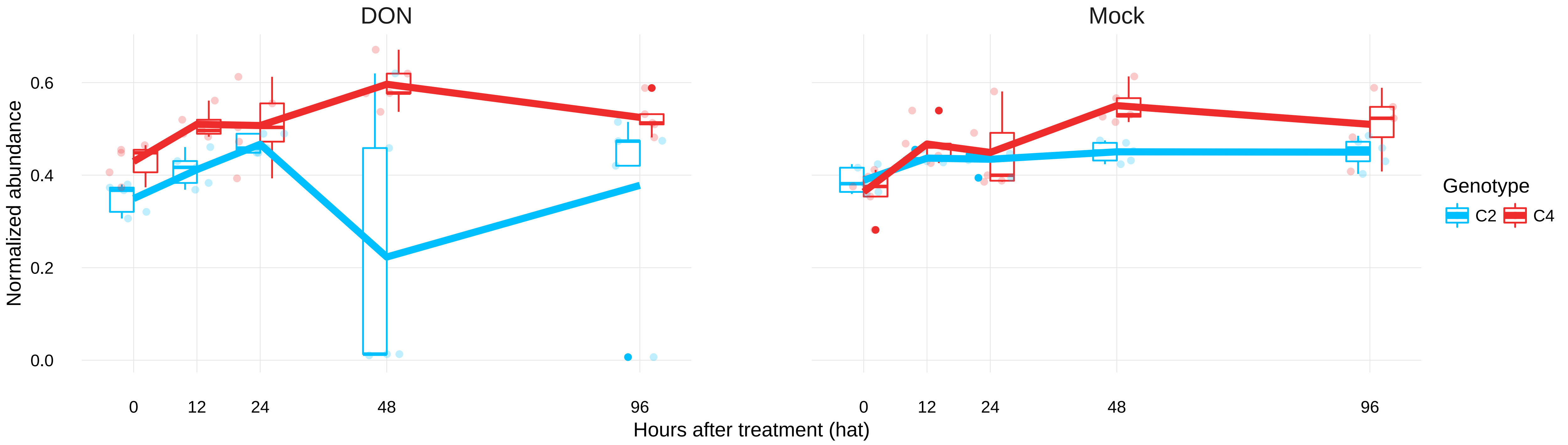

CM, Remus; different treatments

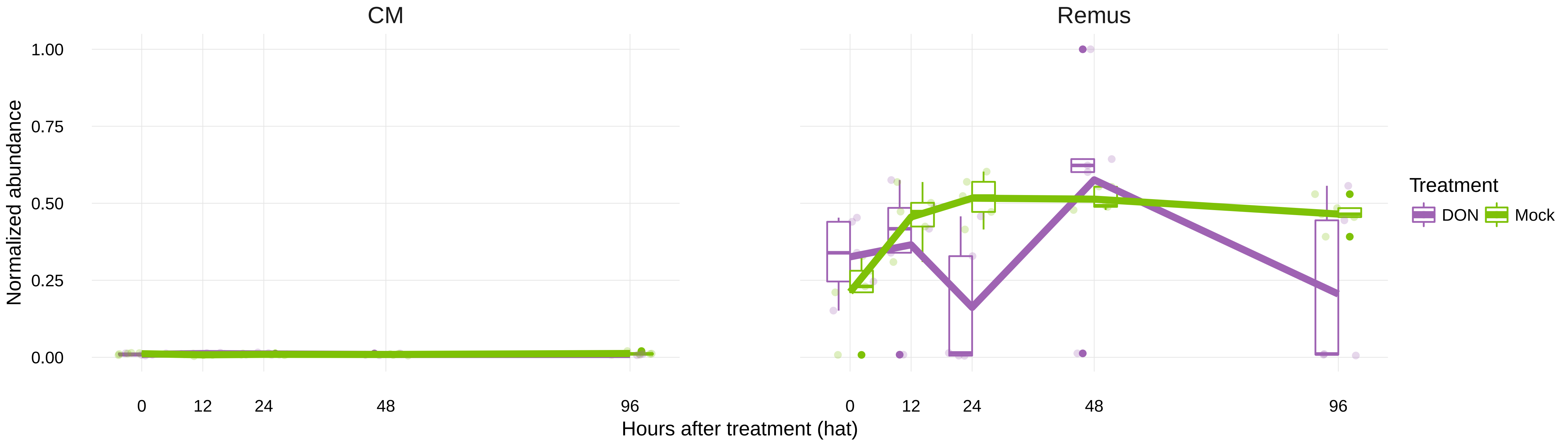

DON, Mock; all four genotypes

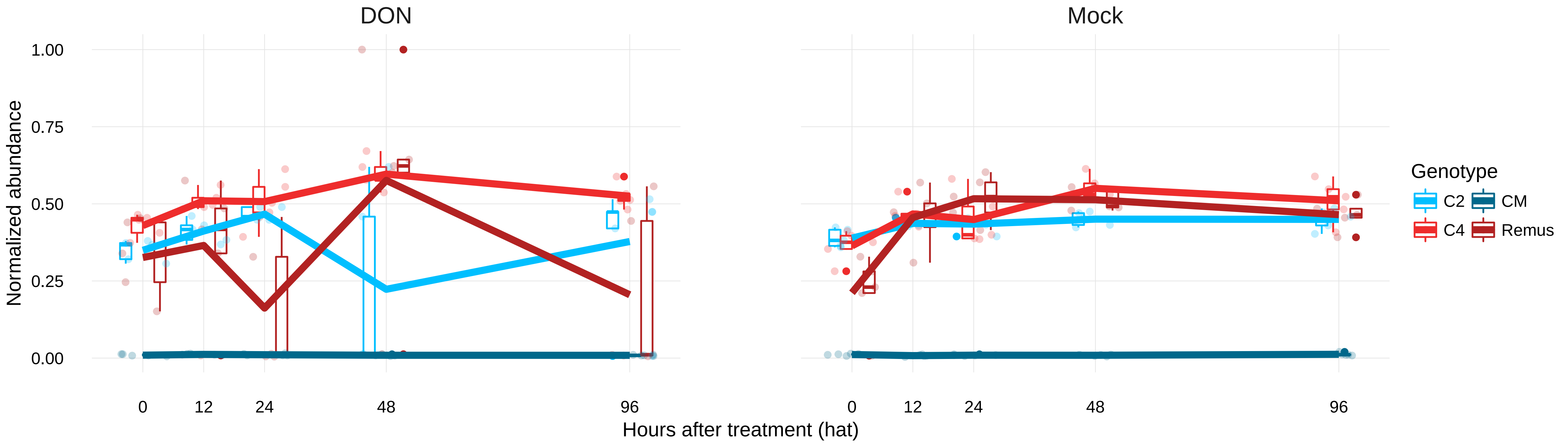

U.3

Unknown Phe−derived wheat−metabolite

MZ	768.2427
RT	9.6 min
Normalization	Directly via KPX samples
Cluster	−
Cn total / Phe	9 / 9

C2, C4; different treatments

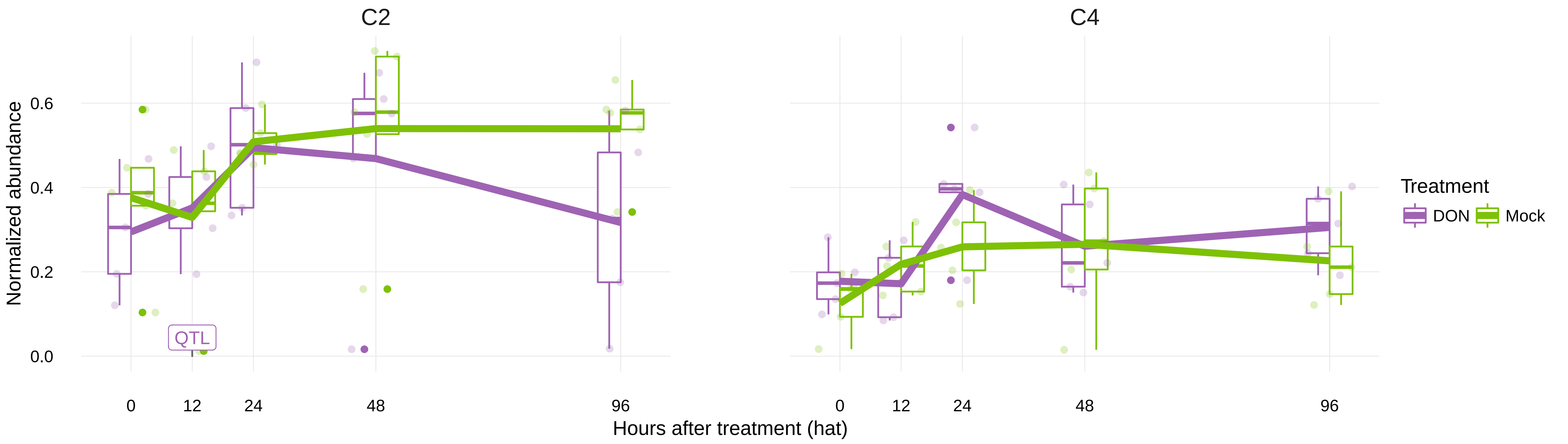

DON, Mock; different genotypes

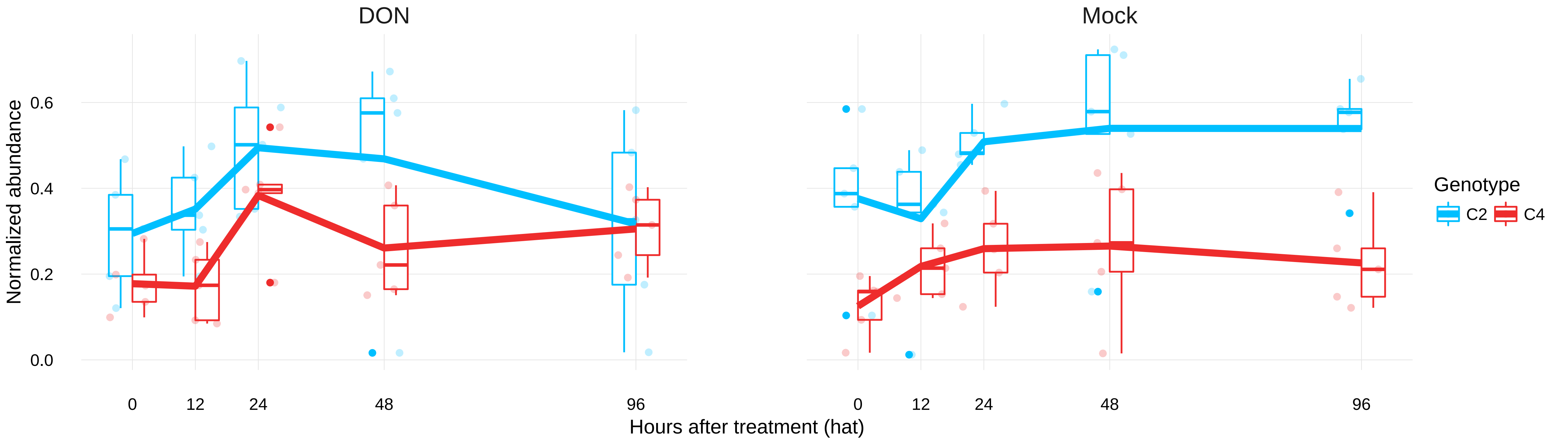

CM, Remus; different treatments

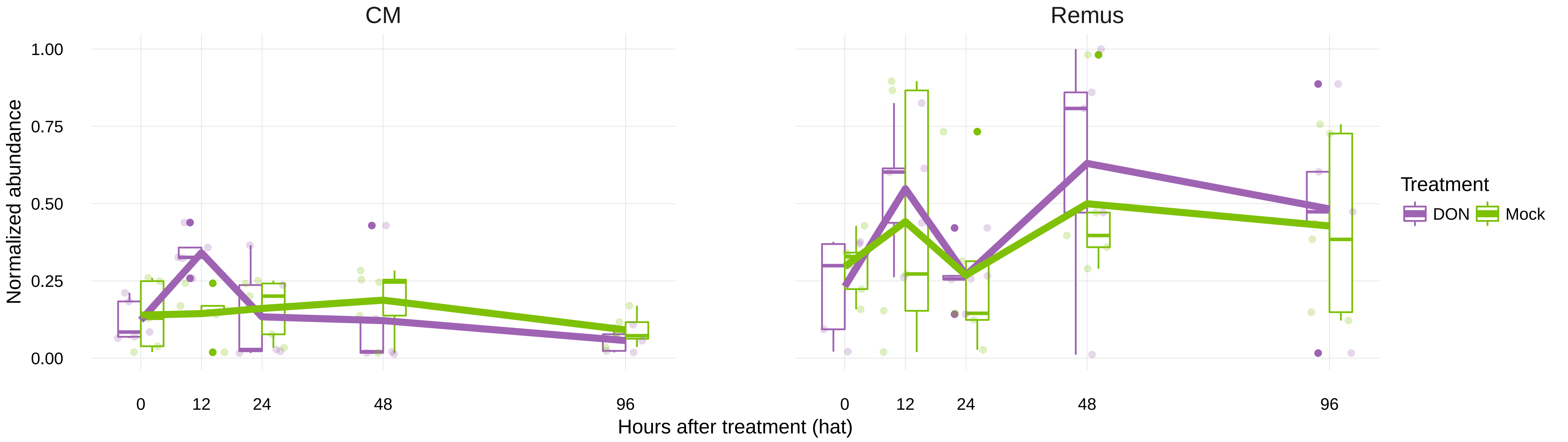

DON, Mock; all four genotypes

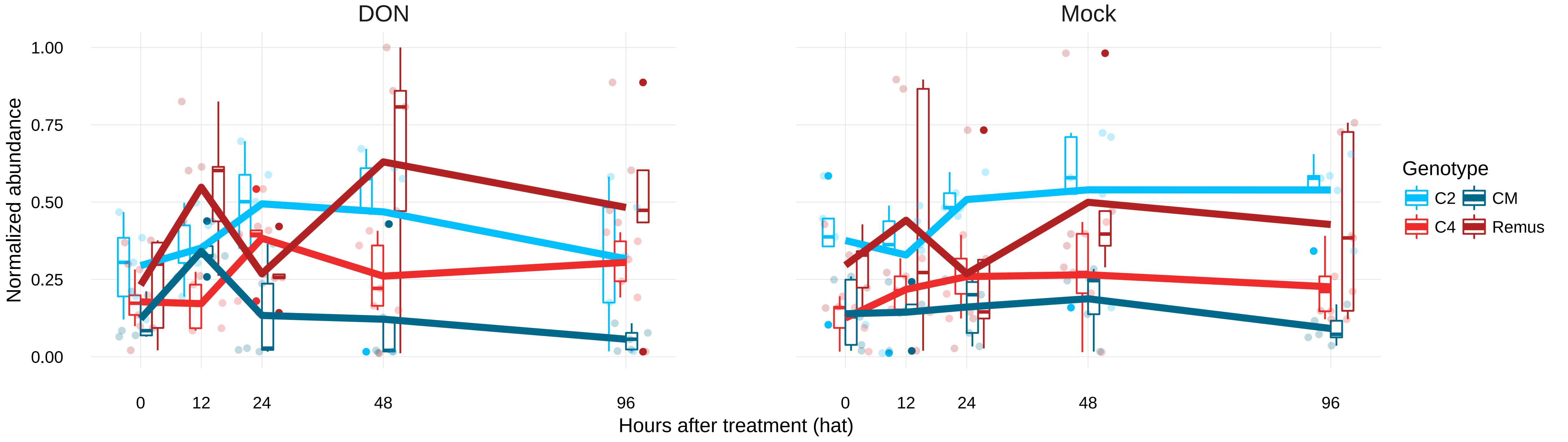

A.26

Annotated as HCA derivative
(7 database hits)

MZ	167.0703
RT	8.99 min
Normalization	Directly via KPX samples
Cluster	–
Cn total / Phe	9 /

C2, C4; different treatments

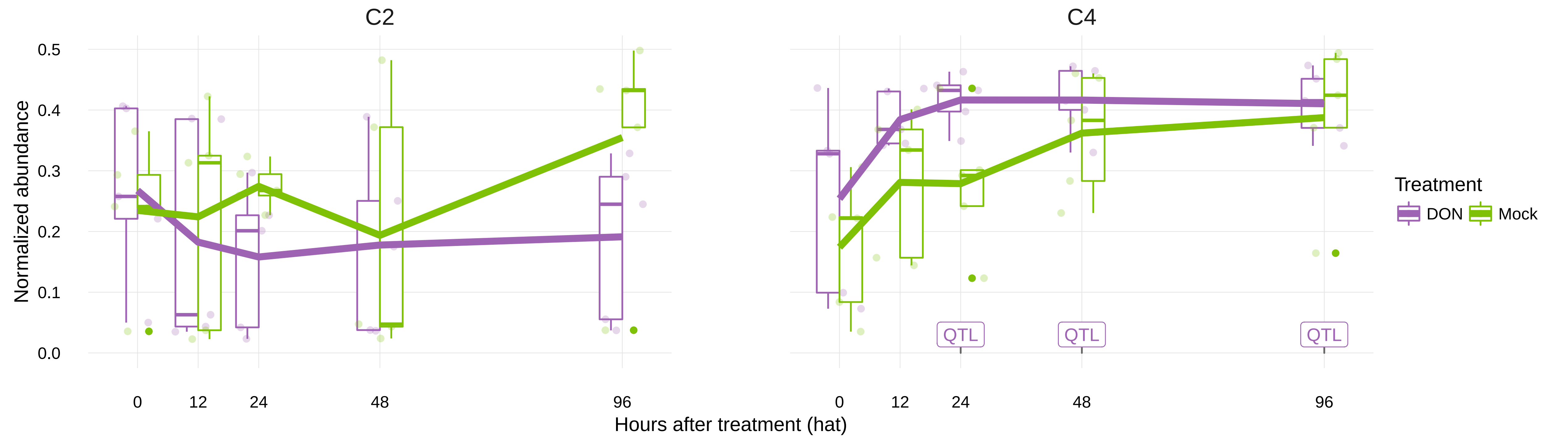

DON, Mock; different genotypes

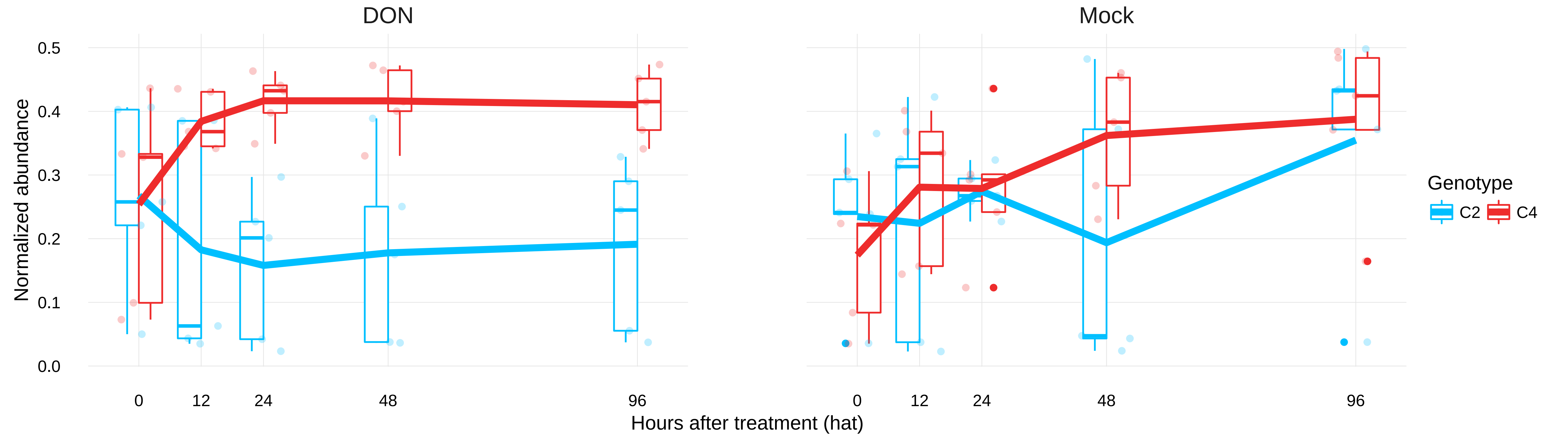

CM, Remus; different treatments

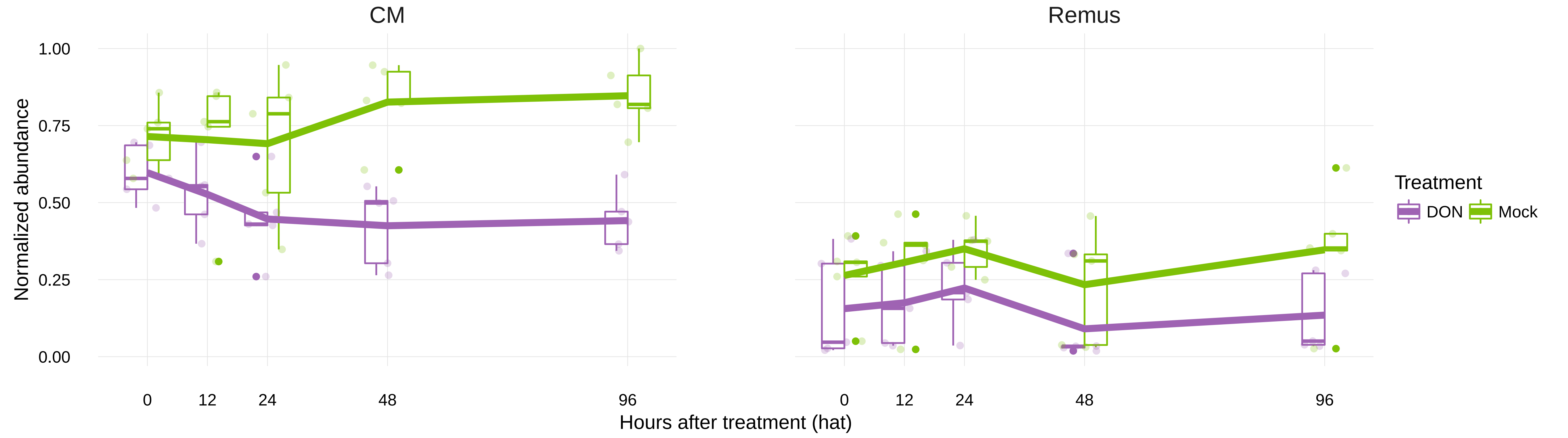

DON, Mock; all four genotypes

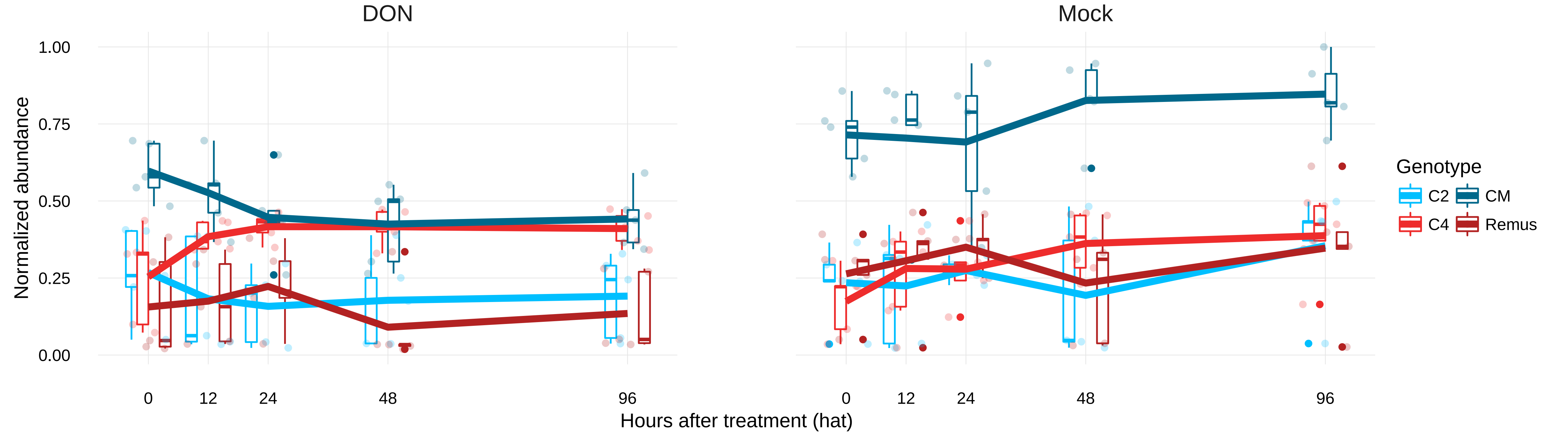

A.132

Annotated as Flavonoid or Lignan
(10 database hits)

MZ	415.1389
RT	21.22 min
Normalization	Indirectly via surrogate in the KPX samples
Cluster	–
Cn total / Phe	22 / 18

C2, C4; different treatments

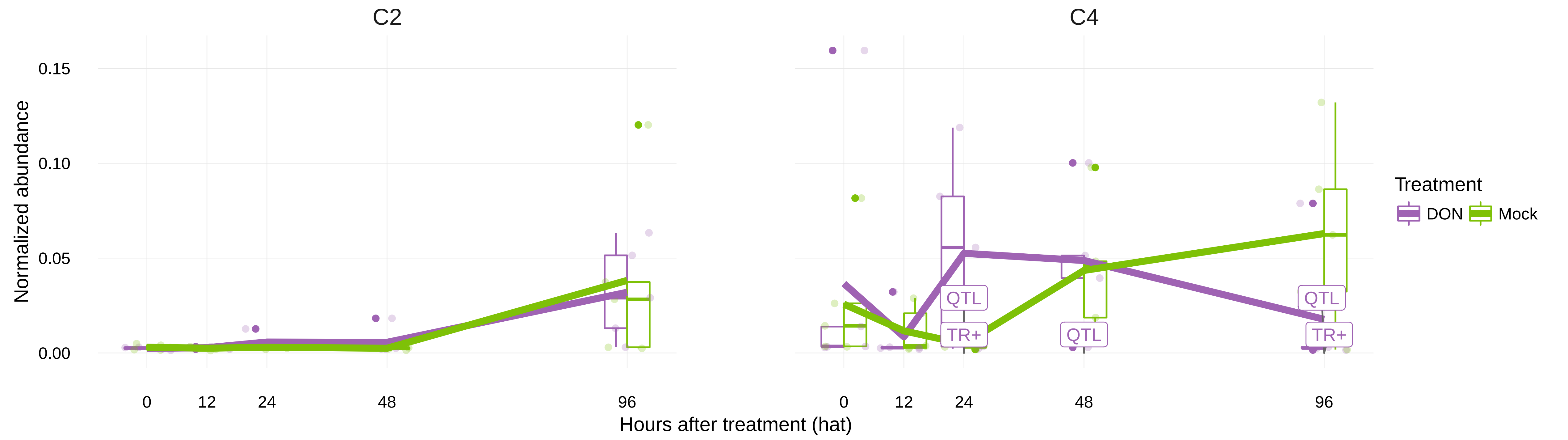

DON, Mock; different genotypes

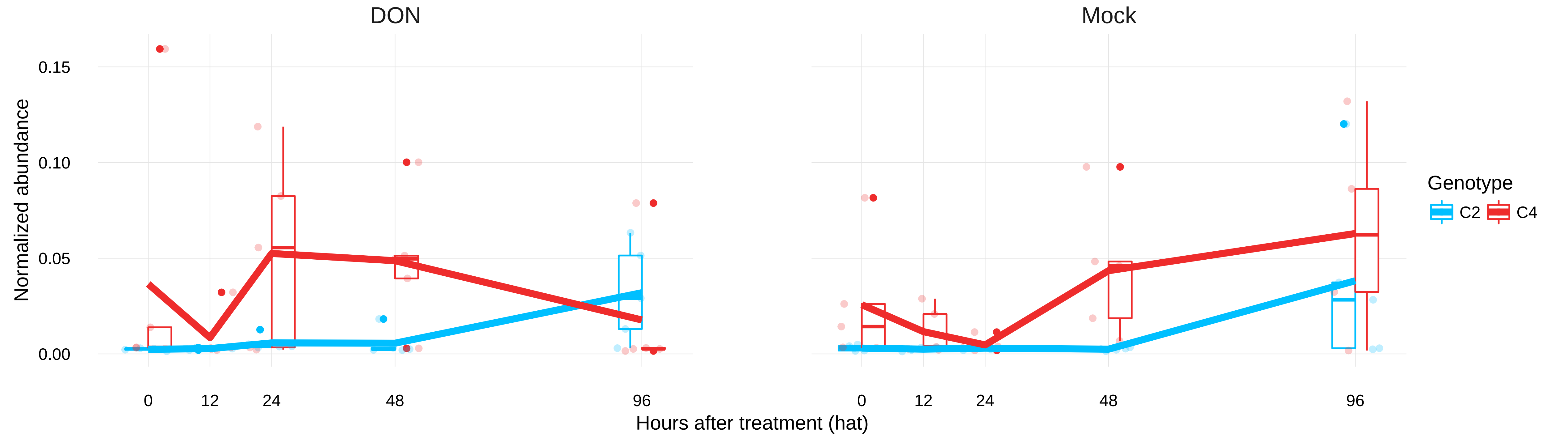

CM, Remus; different treatments

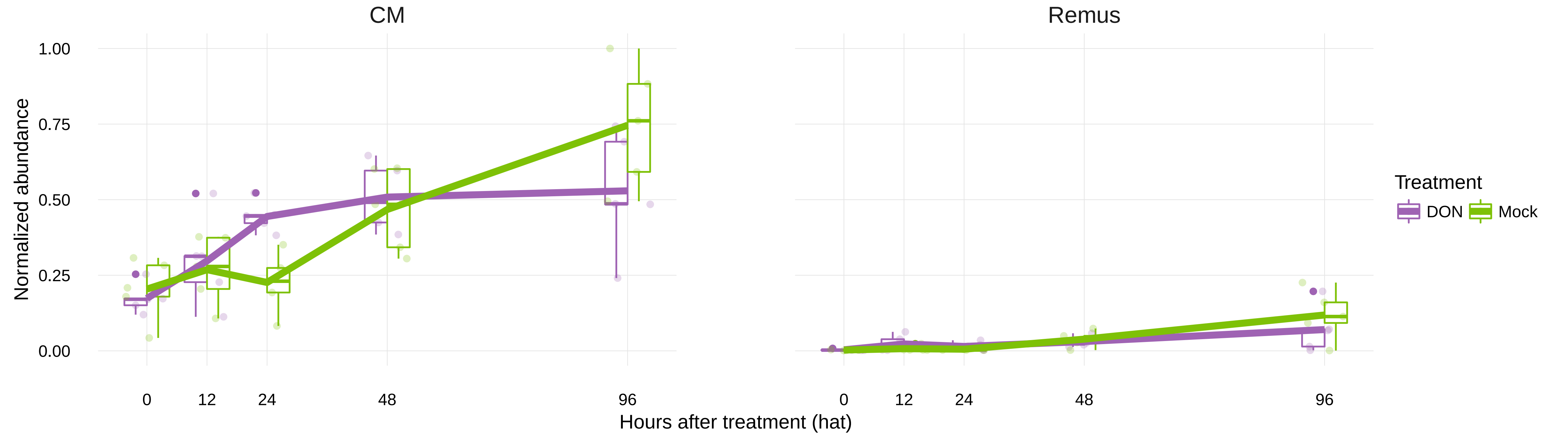

DON, Mock; all four genotypes

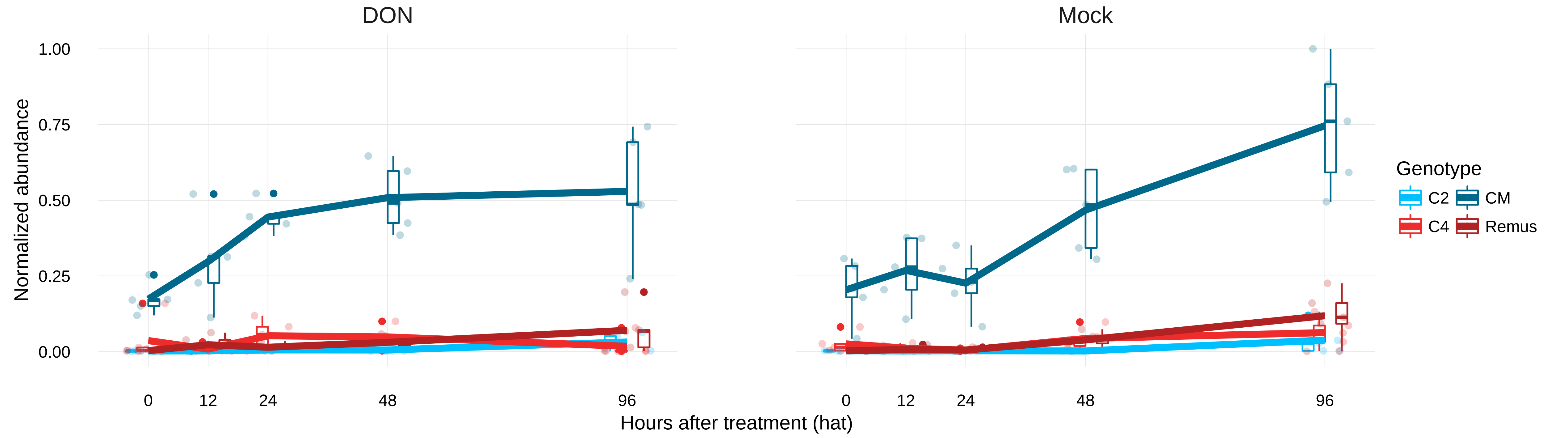

A.108

Annotated as Flavonoid
(30 database hits)

MZ	395.1102
RT	17.59 min
Normalization	Indirectly via surrogate in the KPX samples
Cluster	–
Cn total / Phe	20 /

C2, C4; different treatments

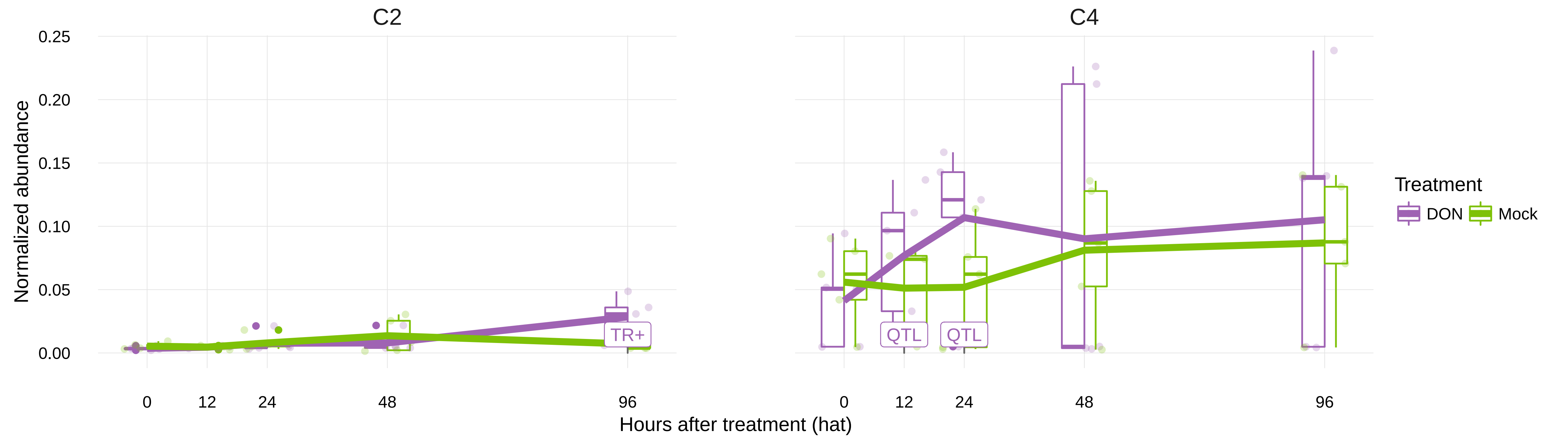

DON, Mock; different genotypes

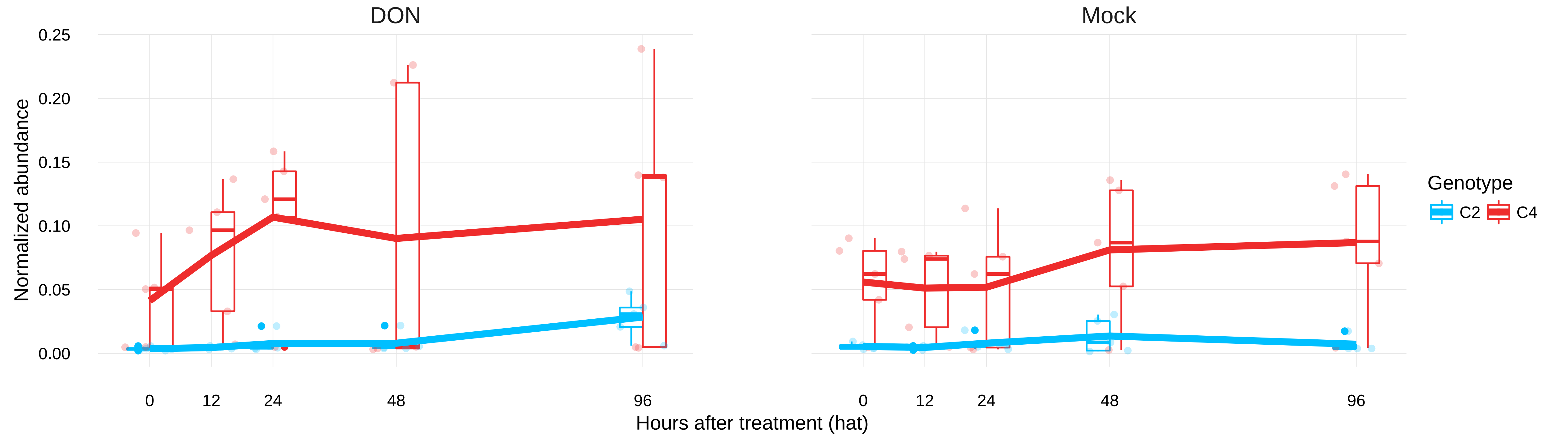

CM, Remus; different treatments

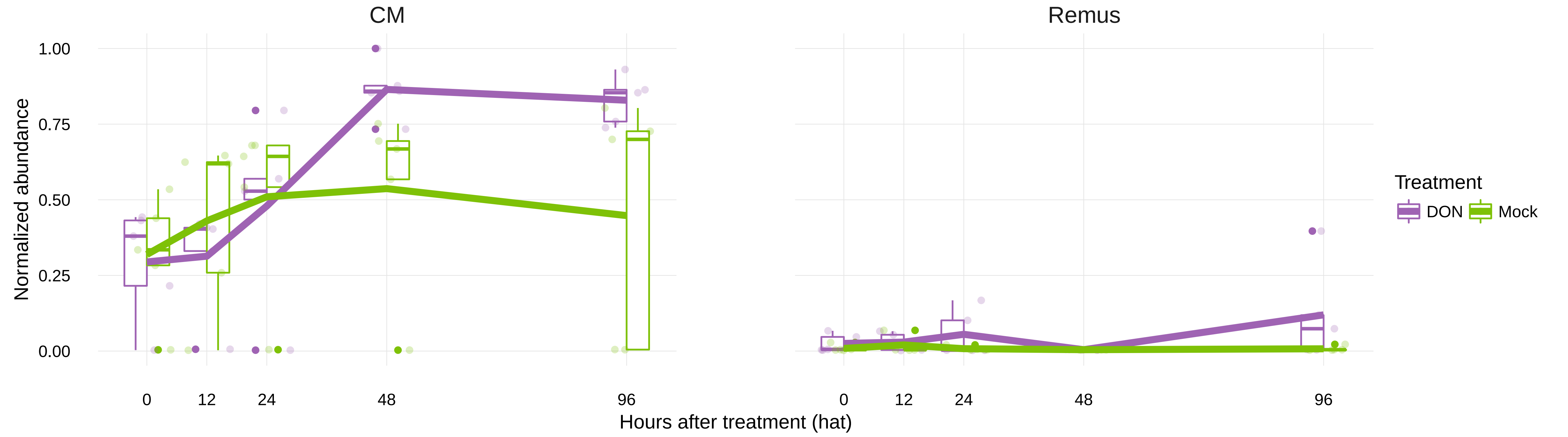

DON, Mock; all four genotypes

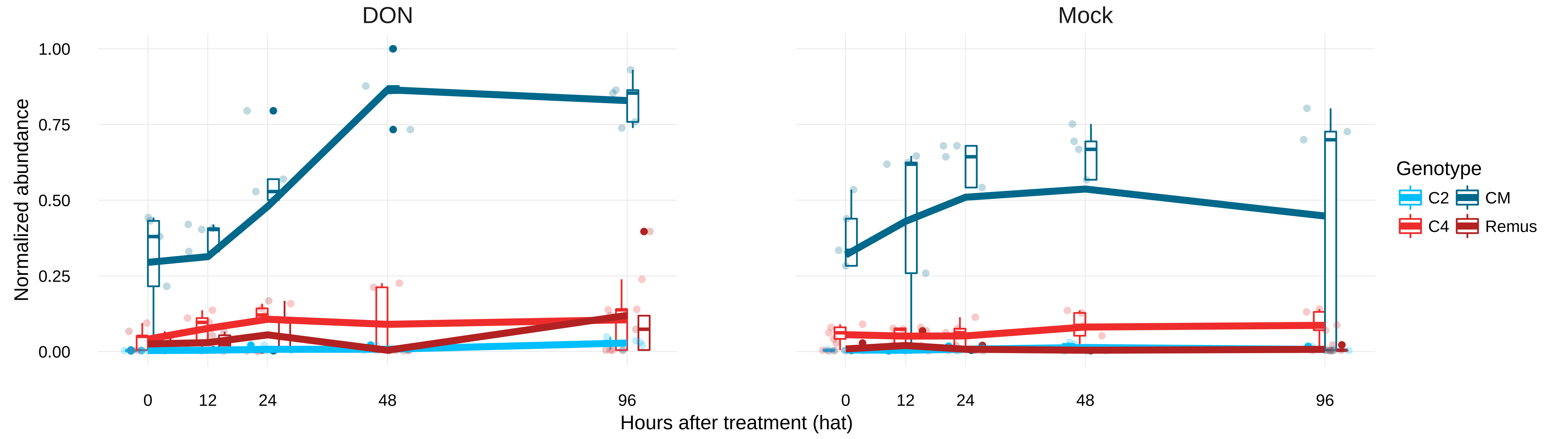

U.5

Unknown Phe−derived wheat−metabolite

MZ	450.1609
RT	12.78 min
Normalization	Indirectly via surrogate in the KPX samples
Cluster	−
Cn total / Phe	18 / 6

C2, C4; different treatments

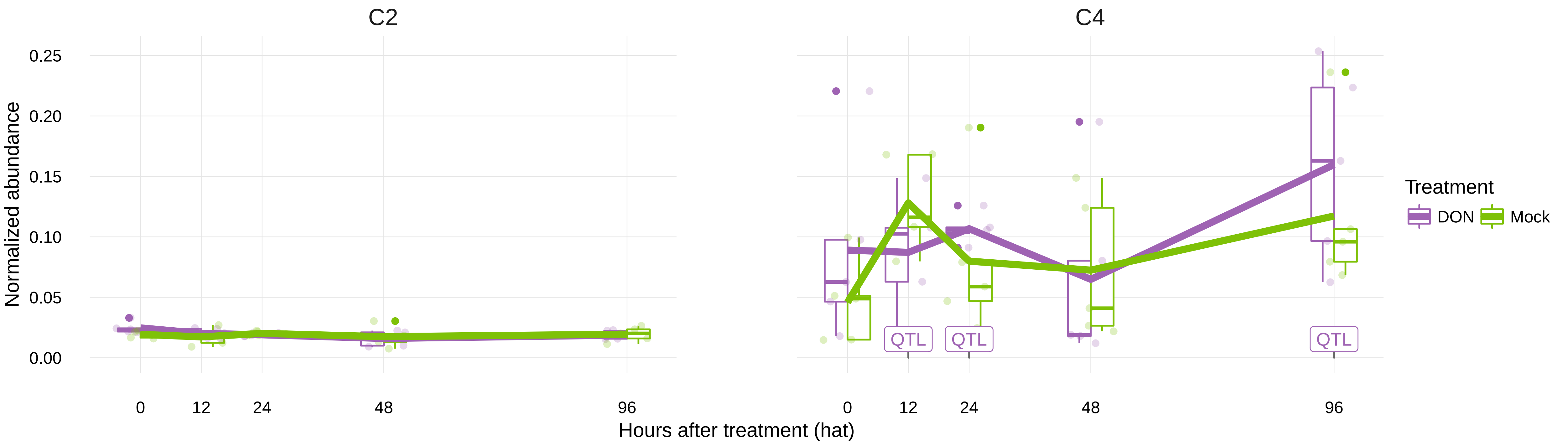

DON, Mock; different genotypes

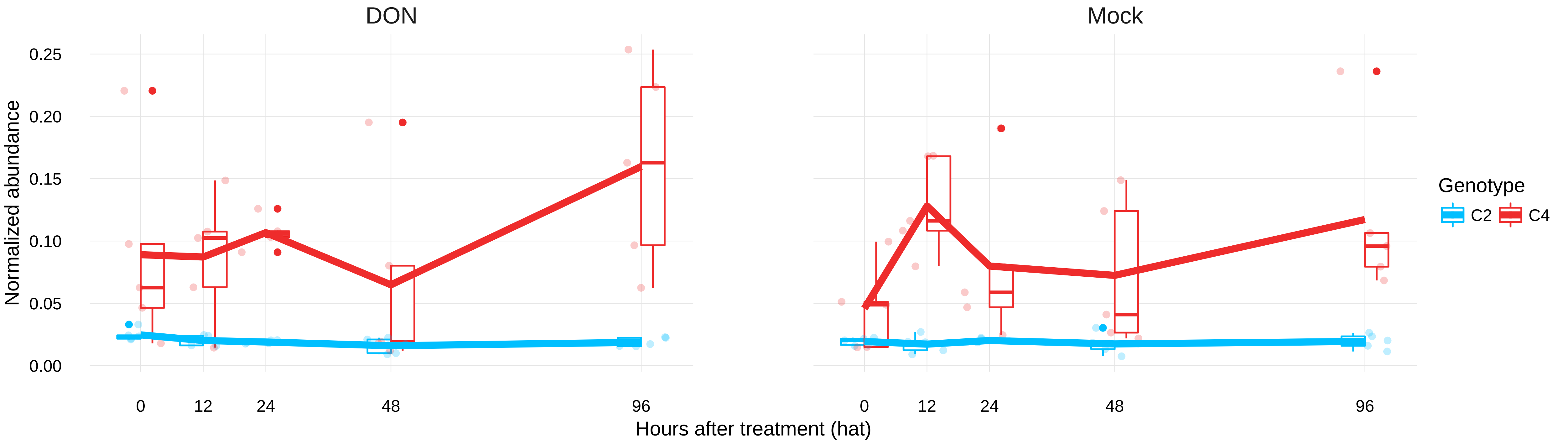

CM, Remus; different treatments

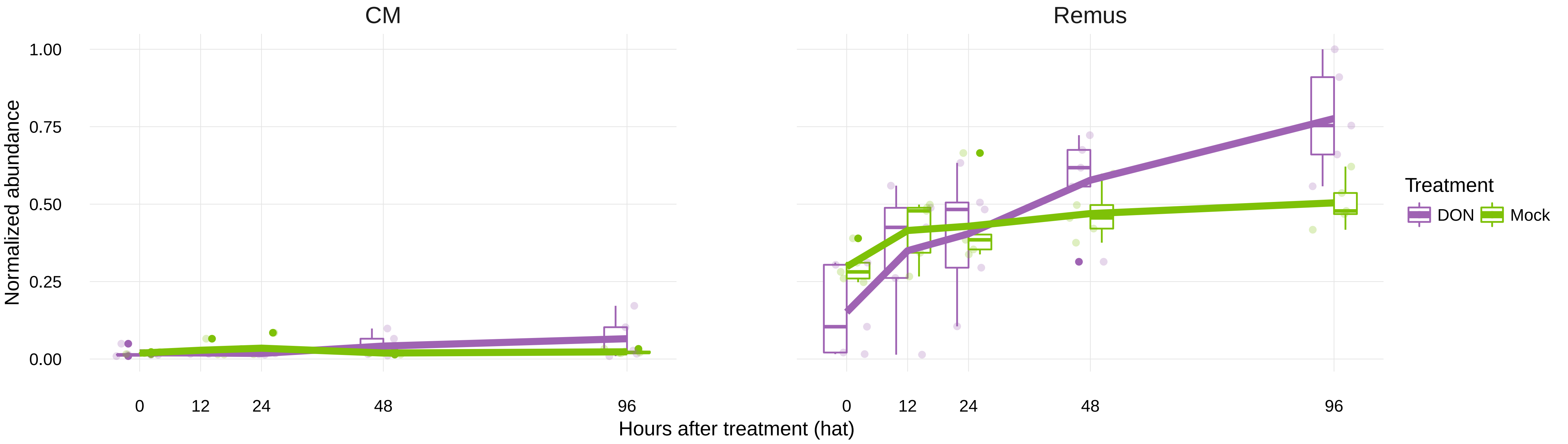

DON, Mock; all four genotypes

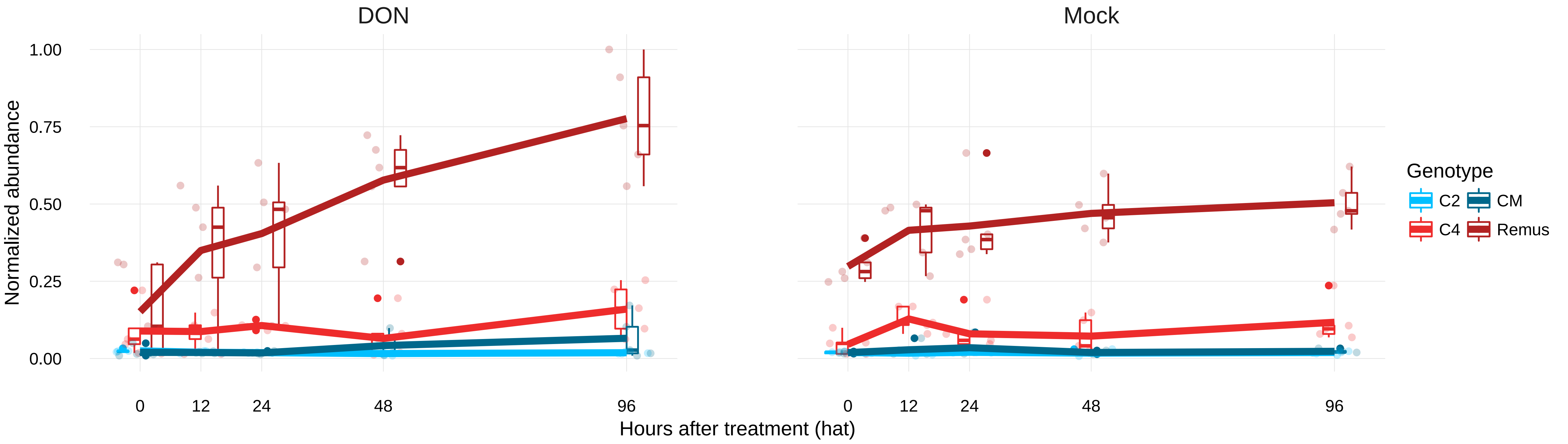

A.86

Annotated as Flavonoid
(30 database hits)

MZ	341.1385
RT	15.2 min
Normalization	Directly via KPX samples
Cluster	–
Cn total / Phe	20 /

C2, C4; different treatments

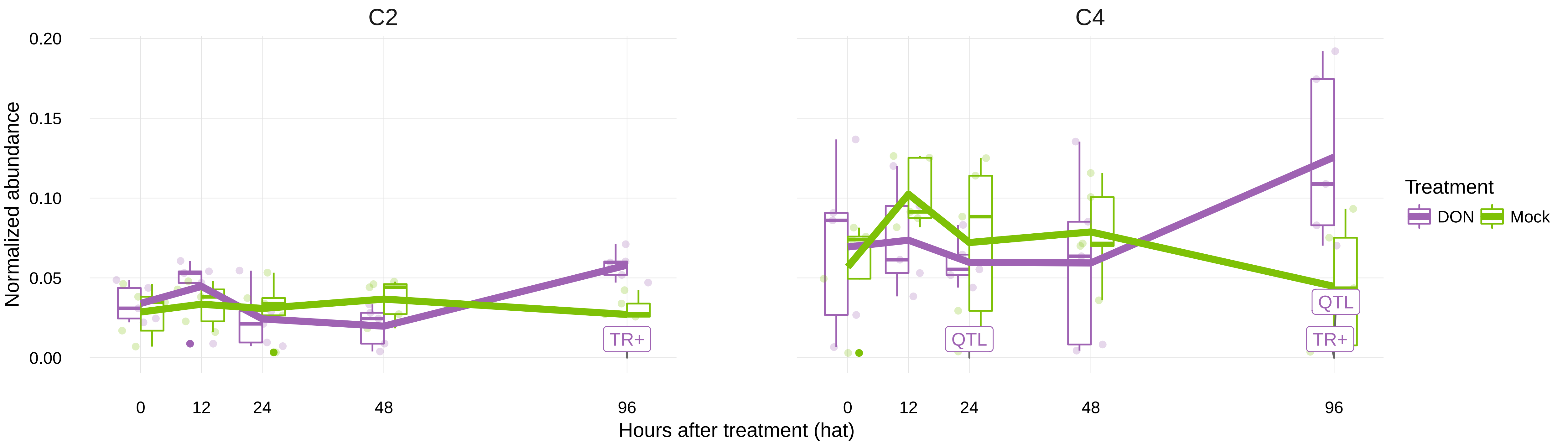

DON, Mock; different genotypes

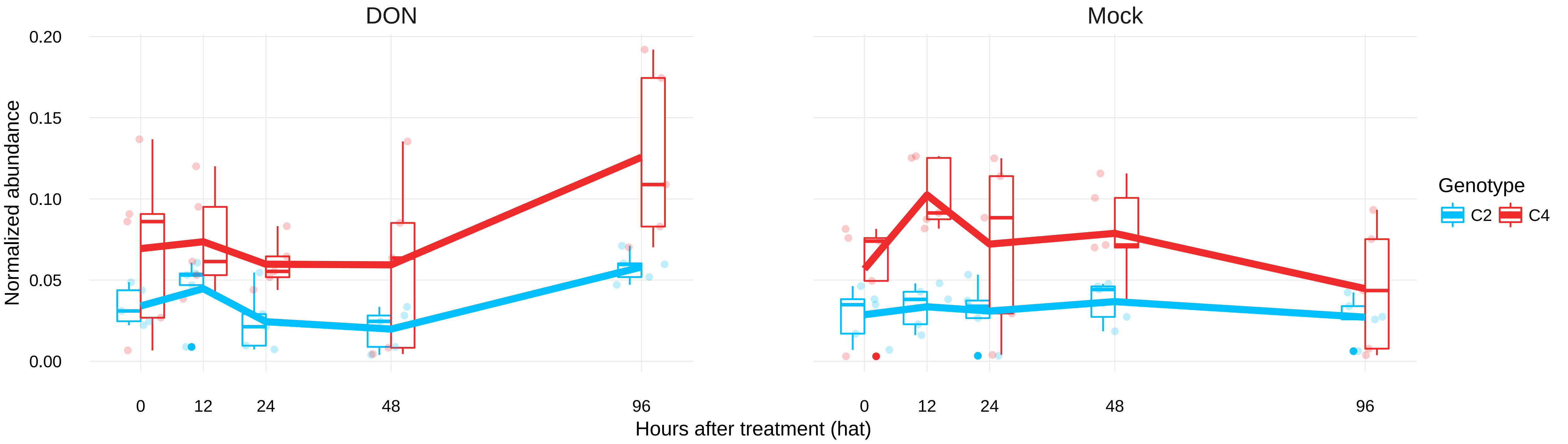

CM, Remus; different treatments

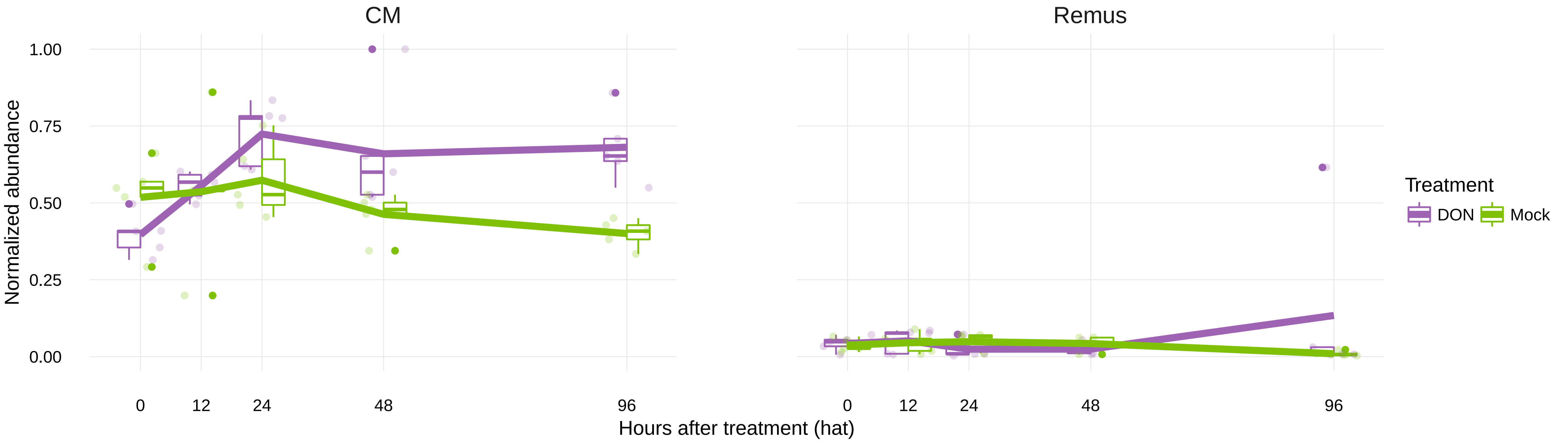

DON, Mock; all four genotypes

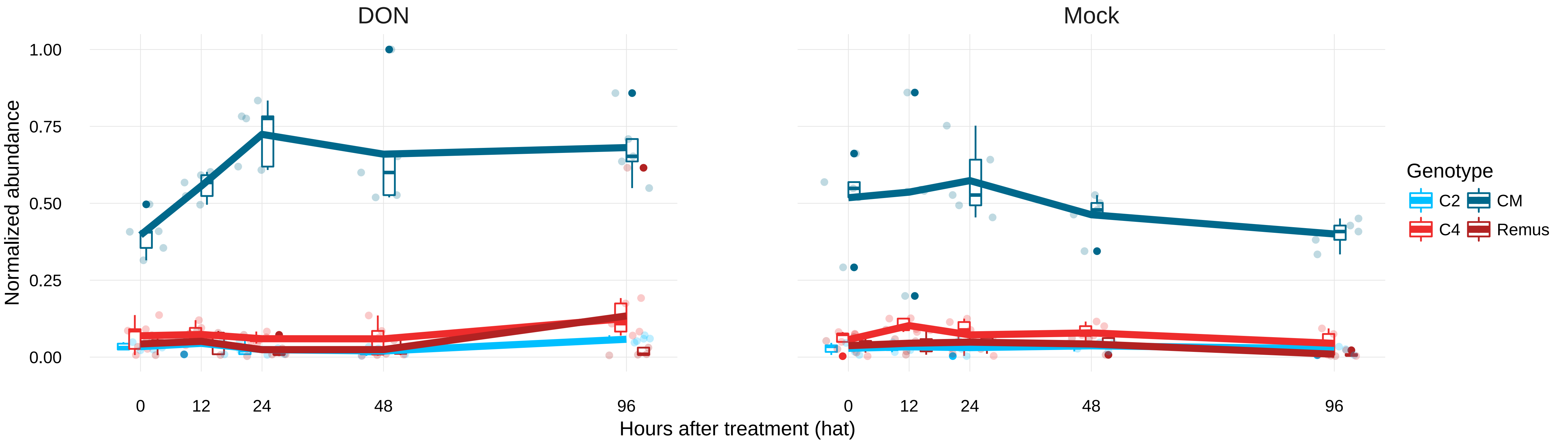

A.73

Annotated as Putative phenylacetic acid derivative
(1 database hit)

MZ	434.2024
RT	13.89 min
Normalization	Directly via KPX samples
Cluster	–
Cn total / Phe	19 / 8

C2, C4; different treatments

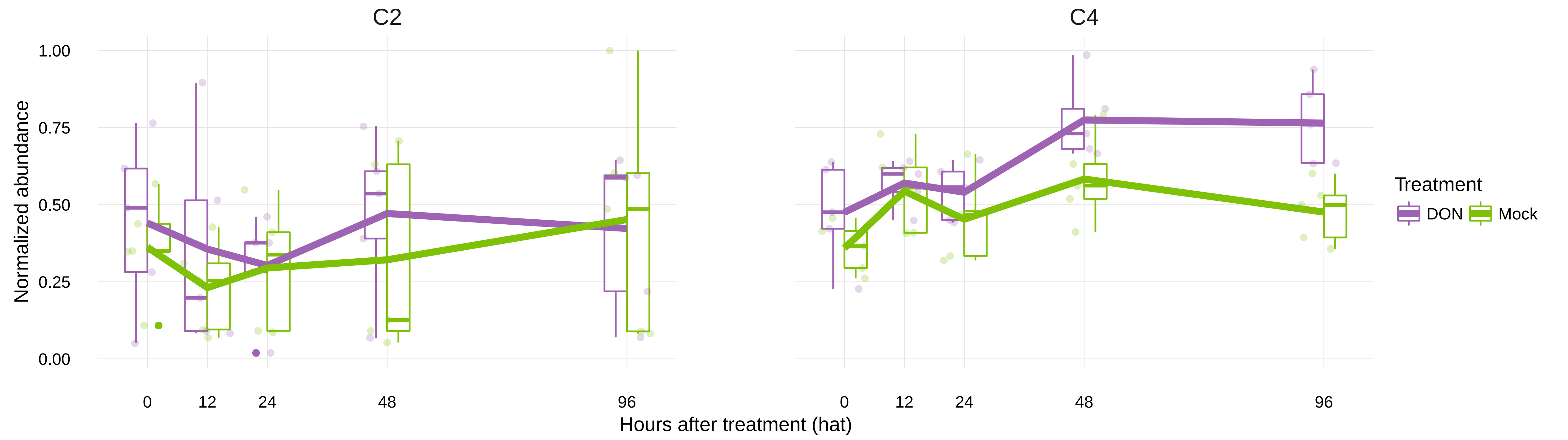

DON, Mock; different genotypes

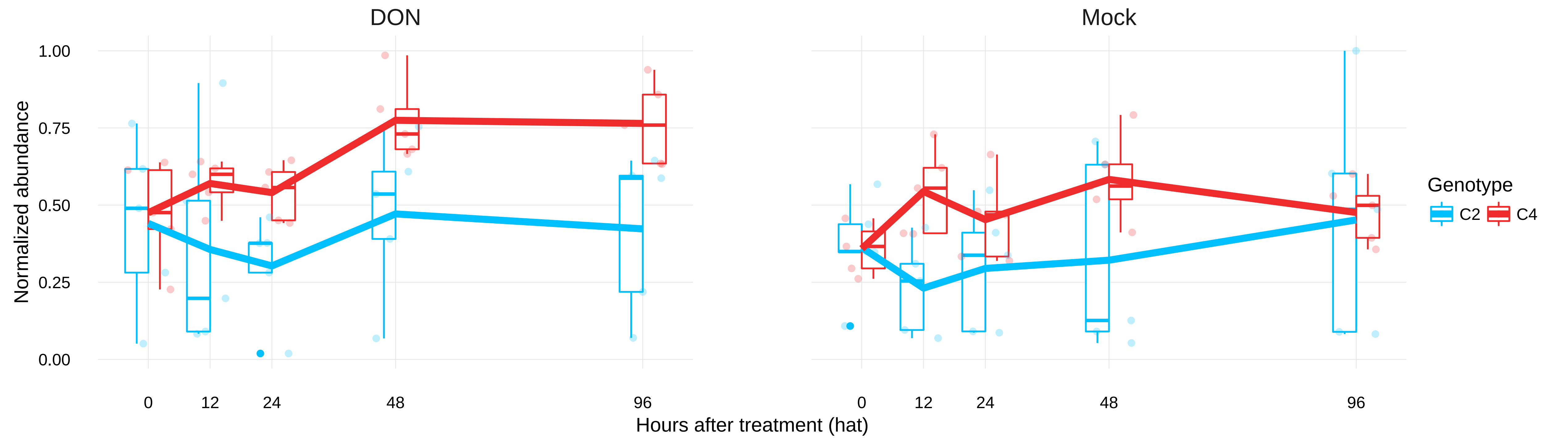

CM, Remus; different treatments

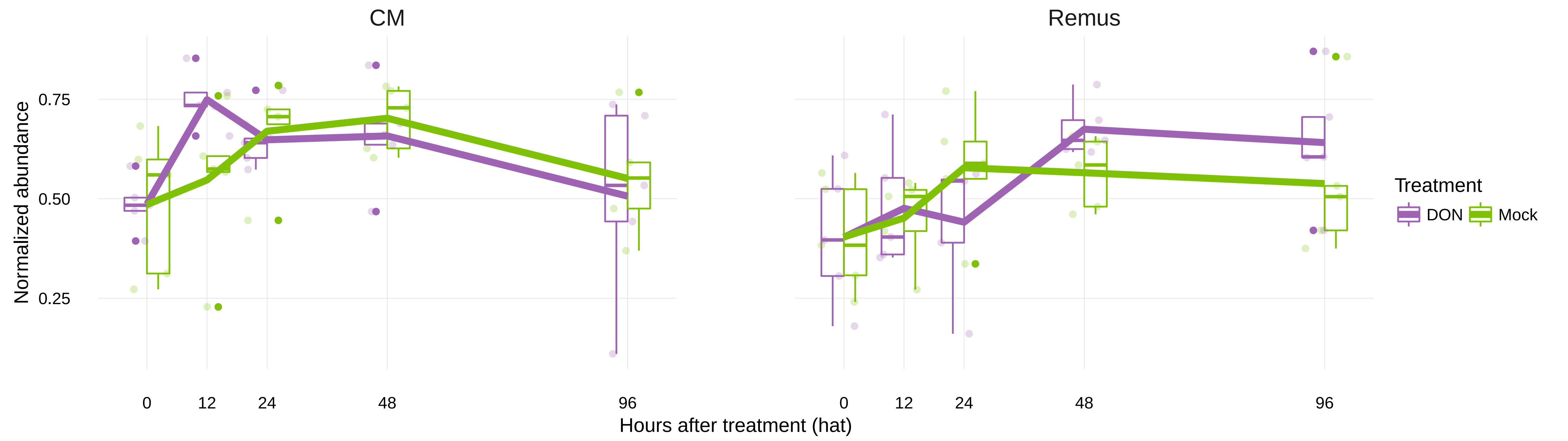

DON, Mock; all four genotypes

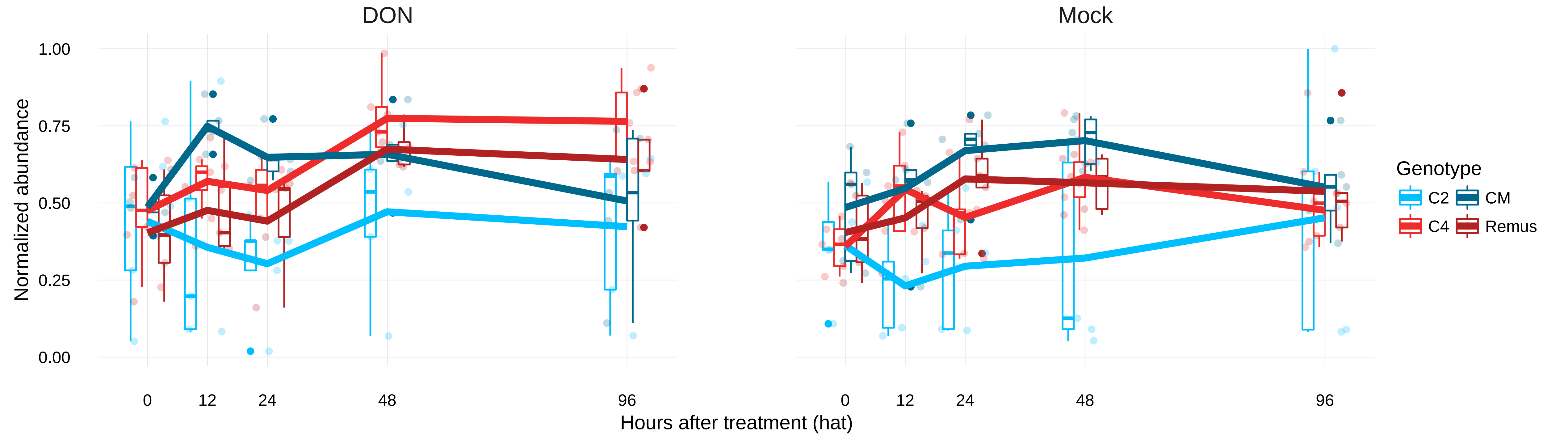

A.78

Annotated as HCAA (diHCAA)
(1 database hit)

MZ	438.2389
RT	14.22 min
Normalization	Indirectly via surrogate in the KPX samples
Cluster	–
Cn total / Phe	25 / 18

C2, C4; different treatments

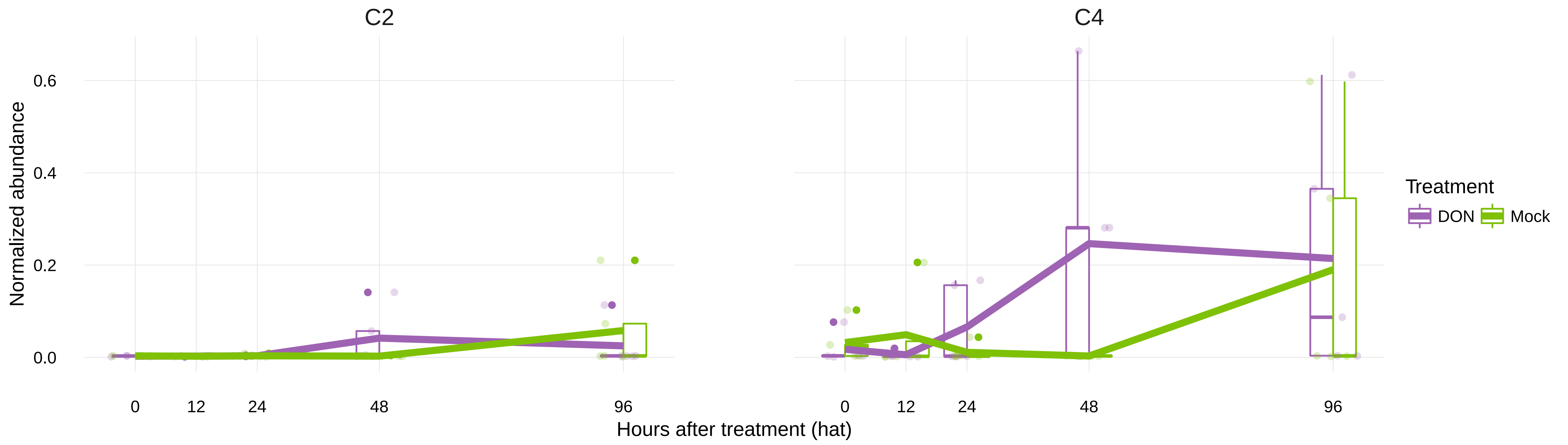

DON, Mock; different genotypes

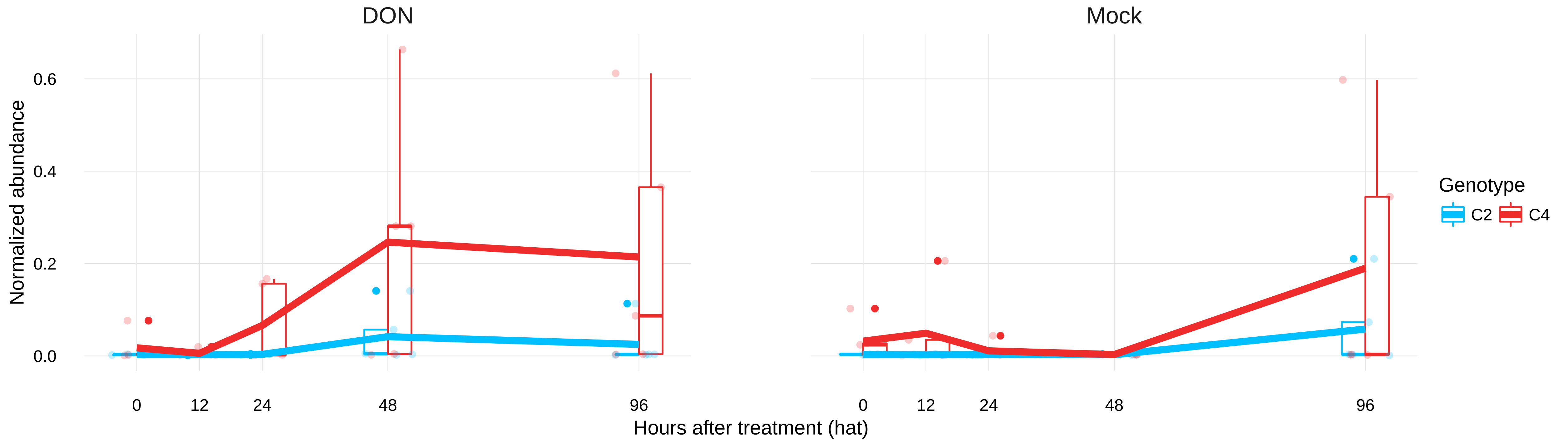

CM, Remus; different treatments

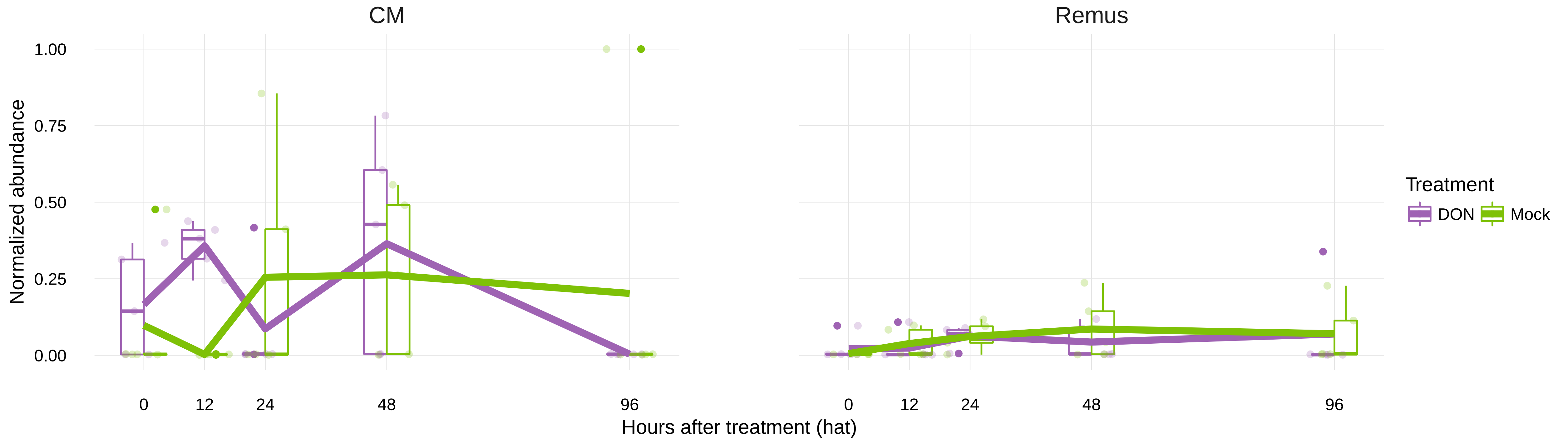

DON, Mock; all four genotypes

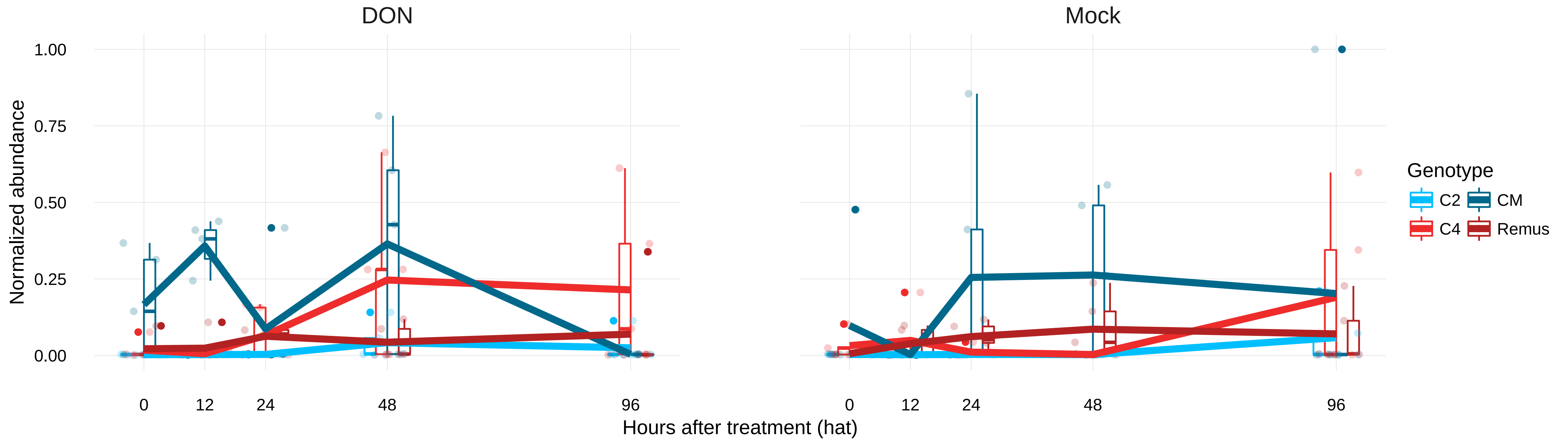

Chlorogenic acid

Identified metabolite (level 1)

MZ	377.0846
RT	9.54 min
Normalization	Directly via KPX samples
Cluster	–
Cn total / Phe	16 /

C2, C4; different treatments

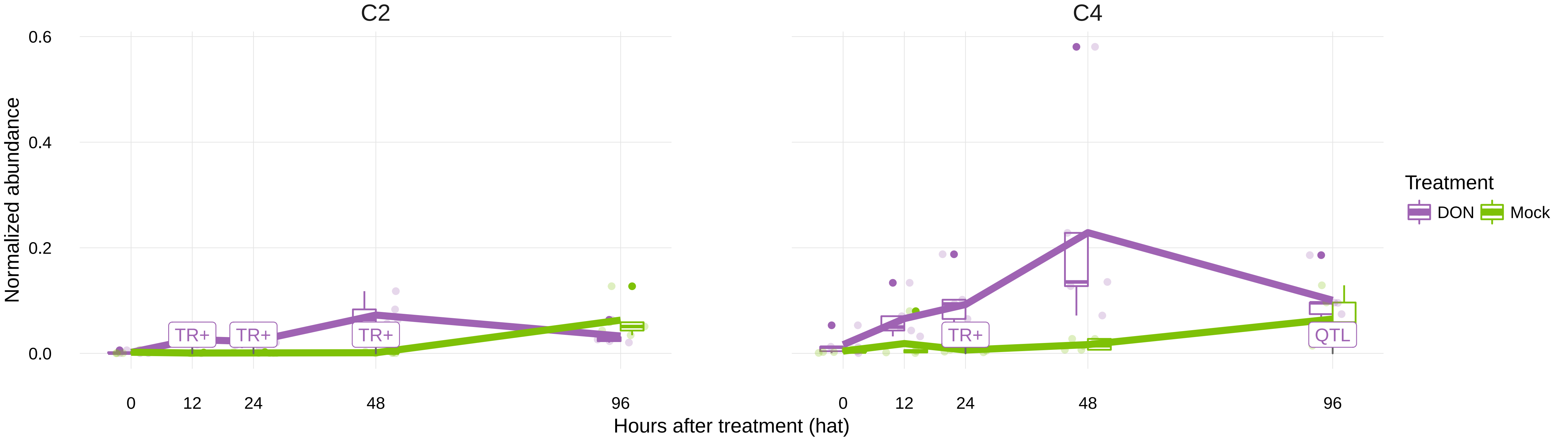

DON, Mock; different genotypes

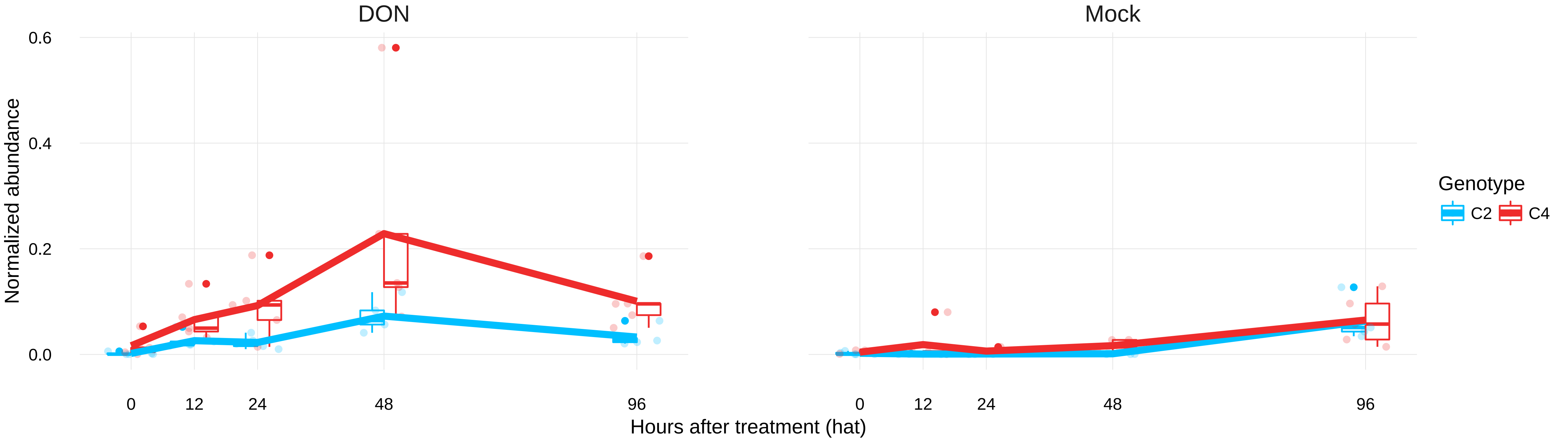

CM, Remus; different treatments

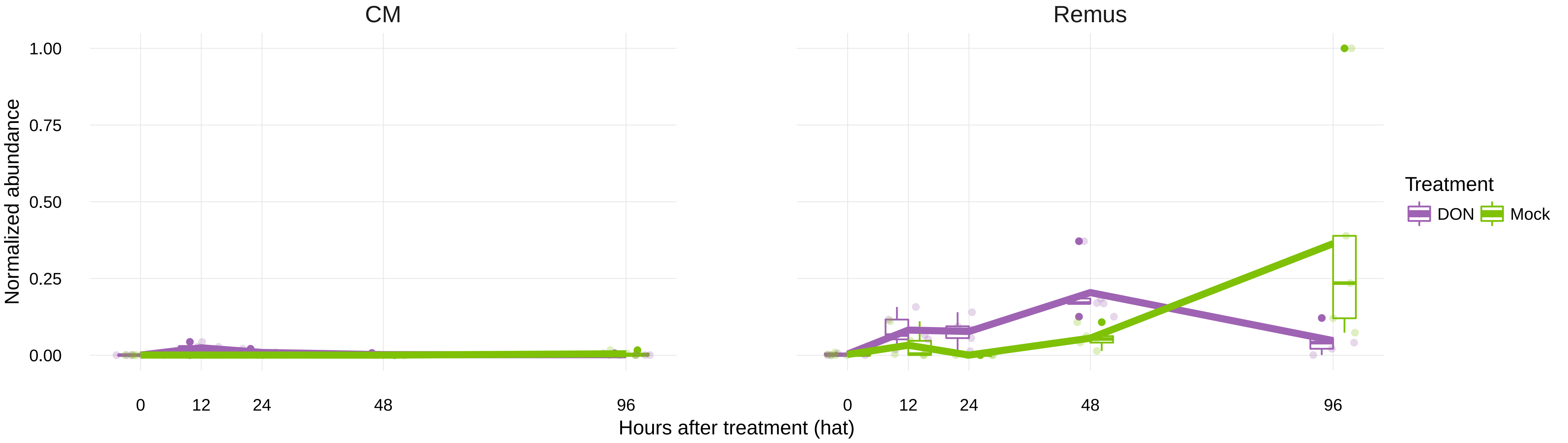

DON, Mock; all four genotypes

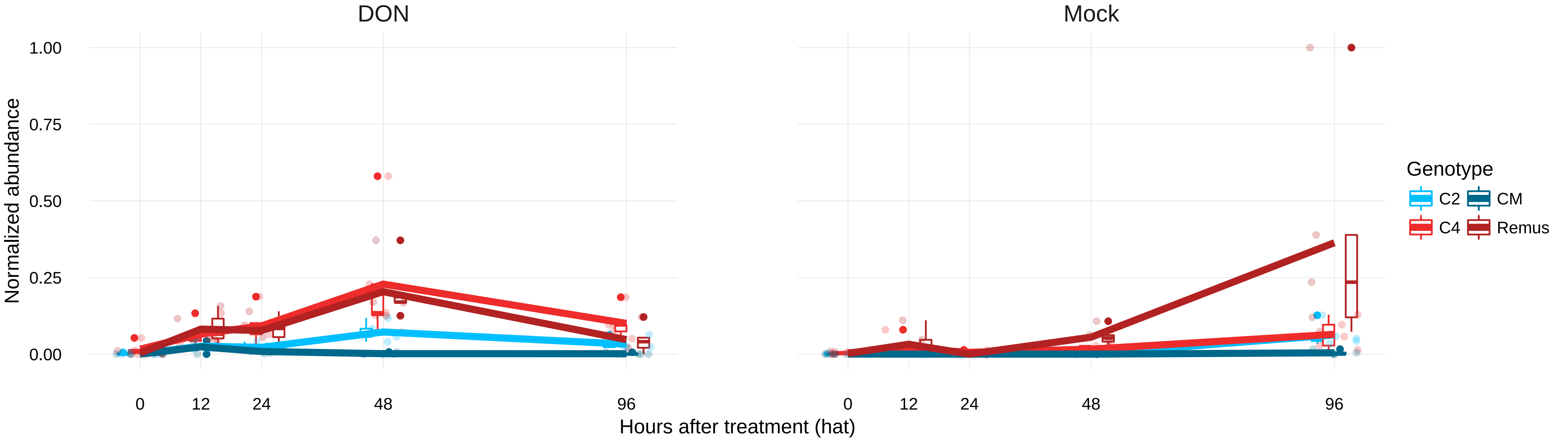

A.105

Annotated as HCA derivative
(1 database hit)

MZ	313.1282
RT	17.23 min
Normalization	Indirectly via surrogate in the KPX samples
Cluster	–
Cn total / Phe	15 /

C2, C4; different treatments

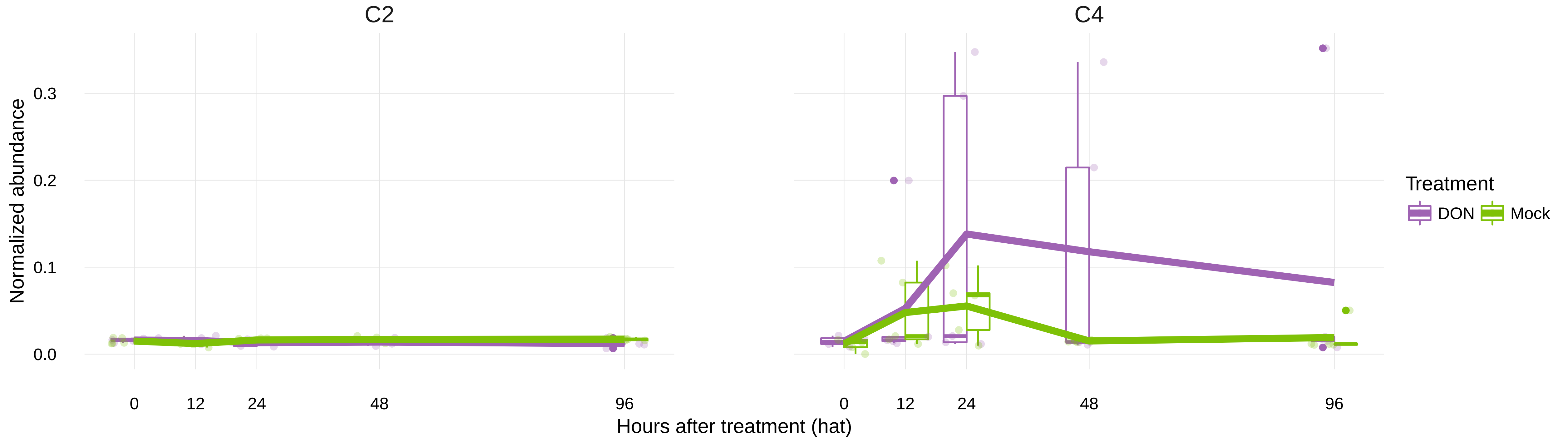

DON, Mock; different genotypes

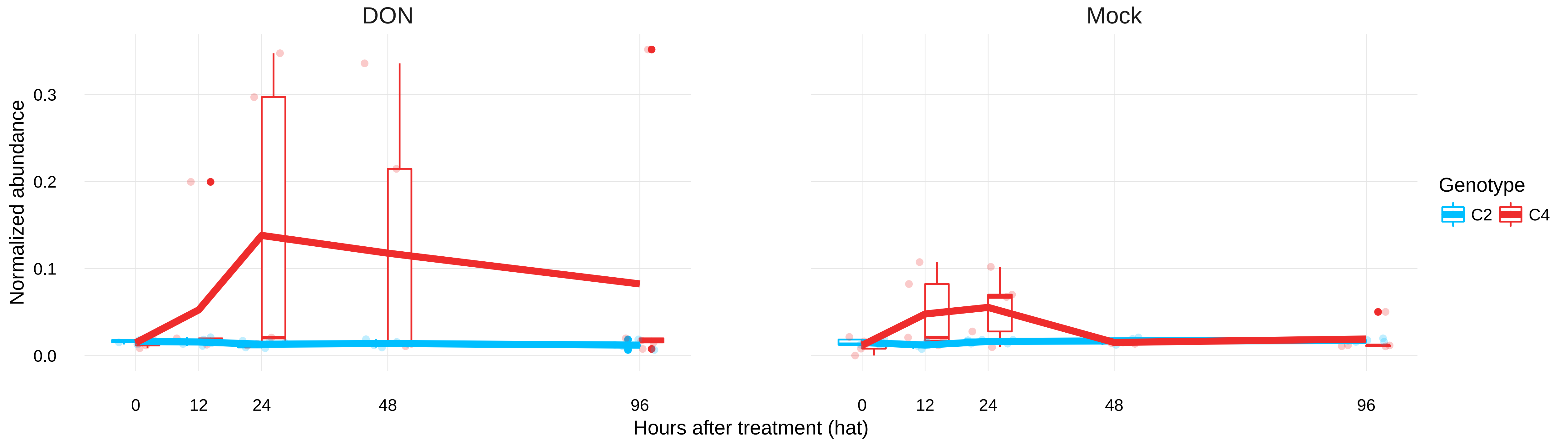

CM, Remus; different treatments

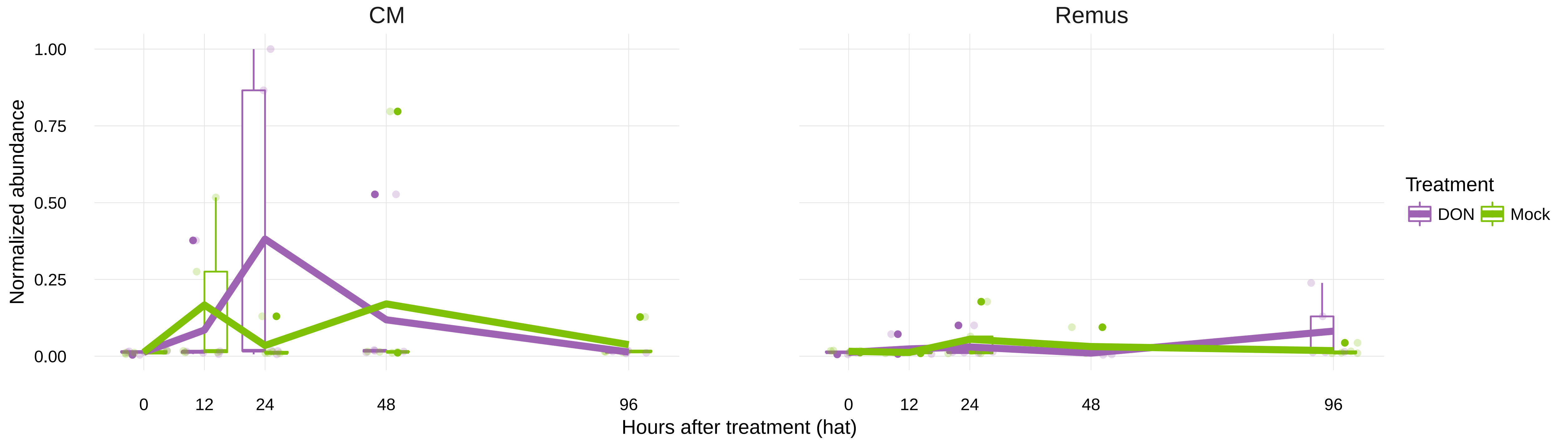

DON, Mock; all four genotypes

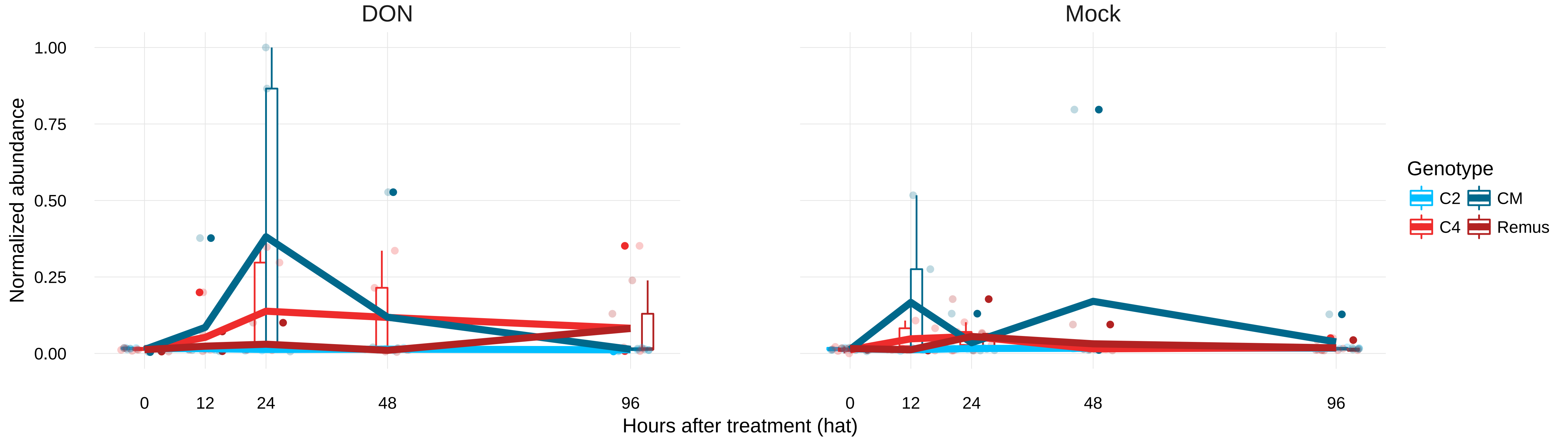

A.5

Annotated as others (Phenol)
(1 database hit)

MZ	127.039
RT	4.18 min
Normalization	Not normalized
Cluster	–
Cn total / Phe	6 /

C2, C4; different treatments

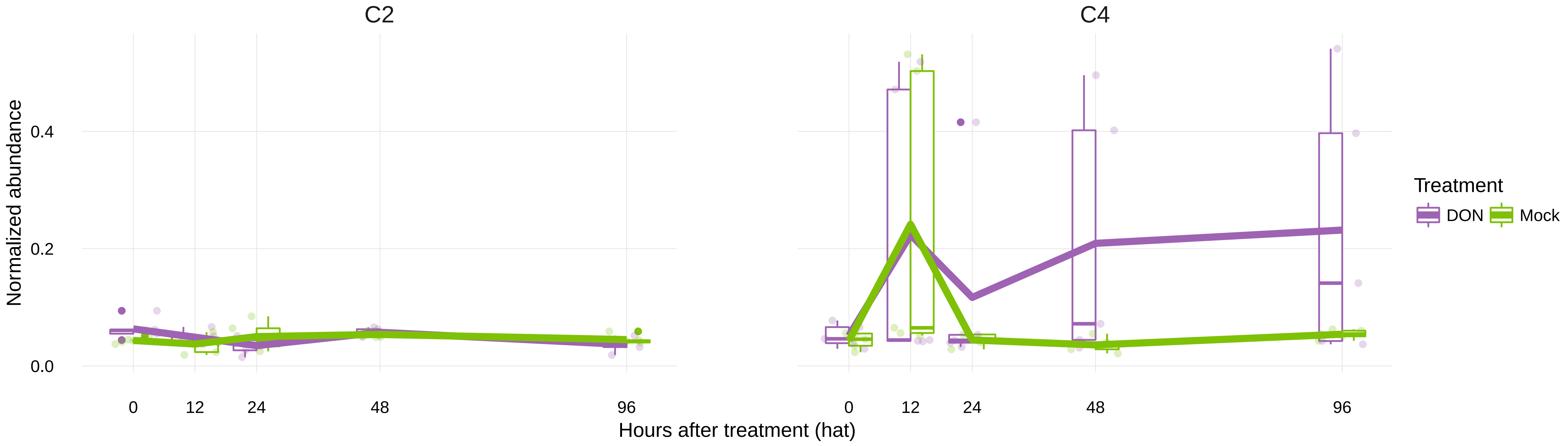

DON, Mock; different genotypes

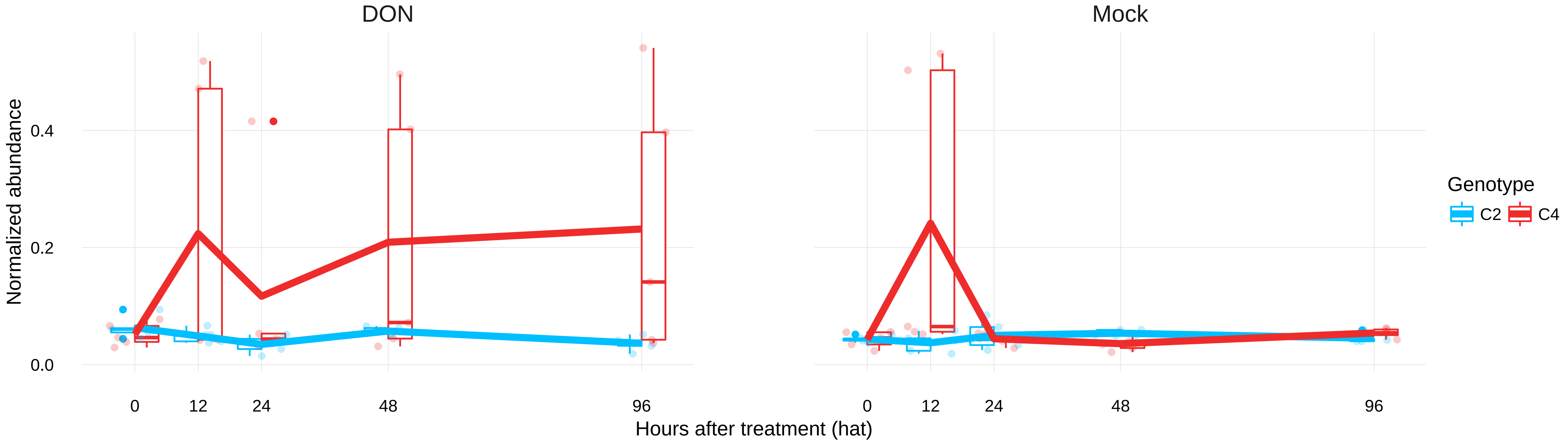

CM, Remus; different treatments

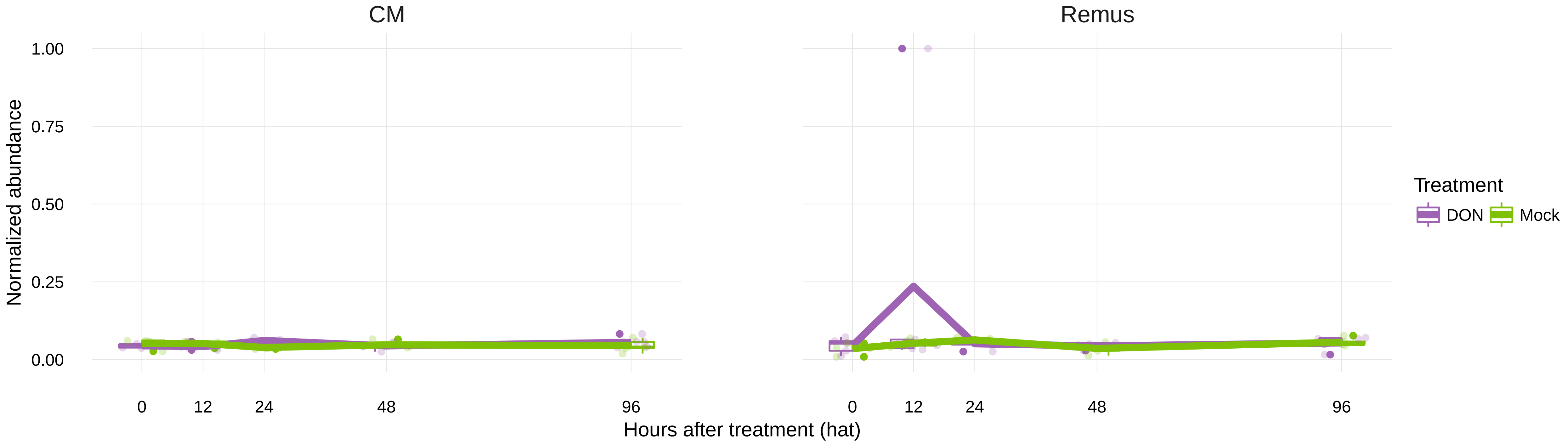

DON, Mock; all four genotypes

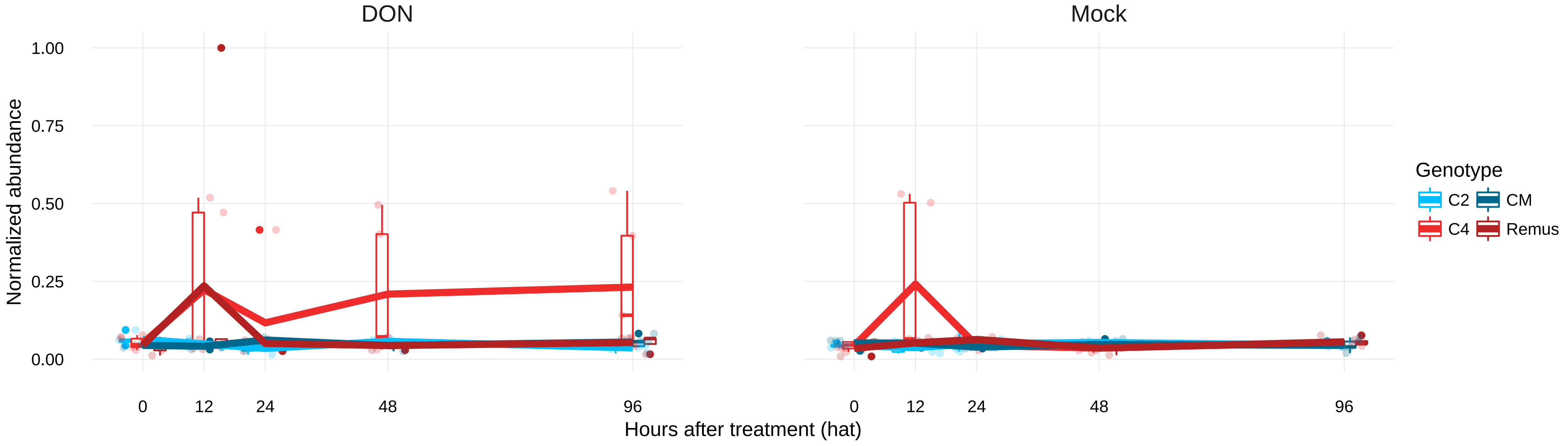

A.17

Annotated as HCA derivative
(1 database hit)

MZ	335.1103
RT	7.29 min
Normalization	Directly via KPX samples
Cluster	–
Cn total / Phe	15 /

C2, C4; different treatments

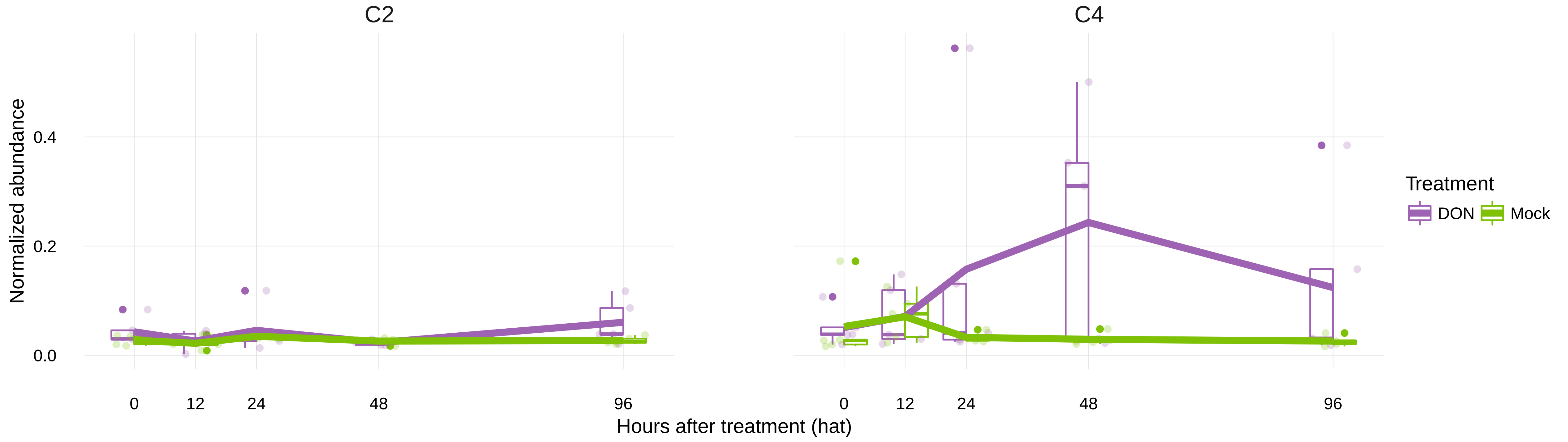

DON, Mock; different genotypes

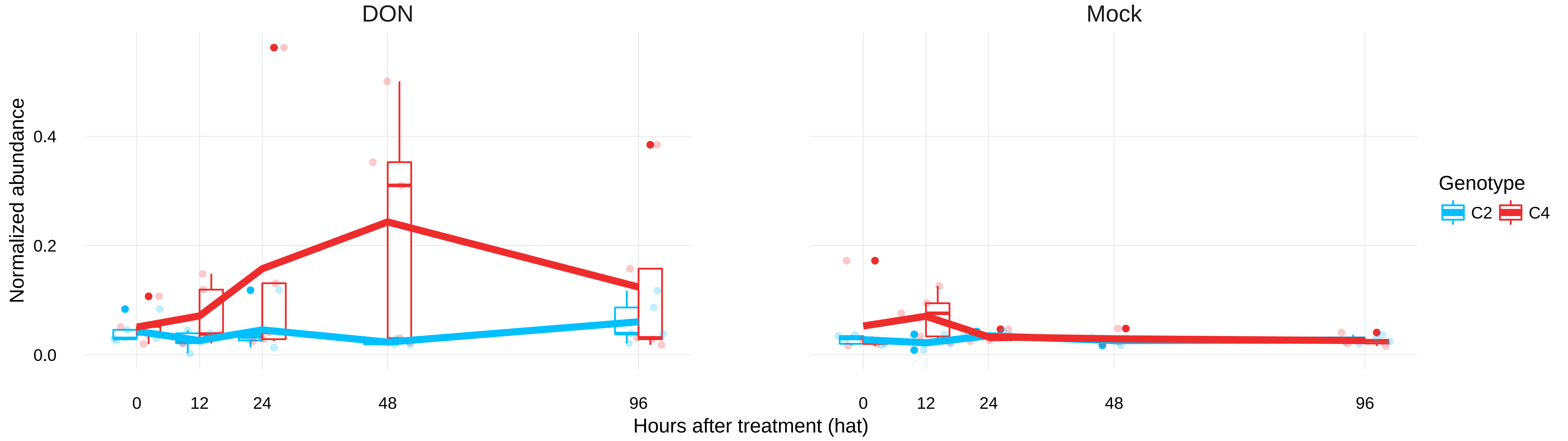

CM, Remus; different treatments

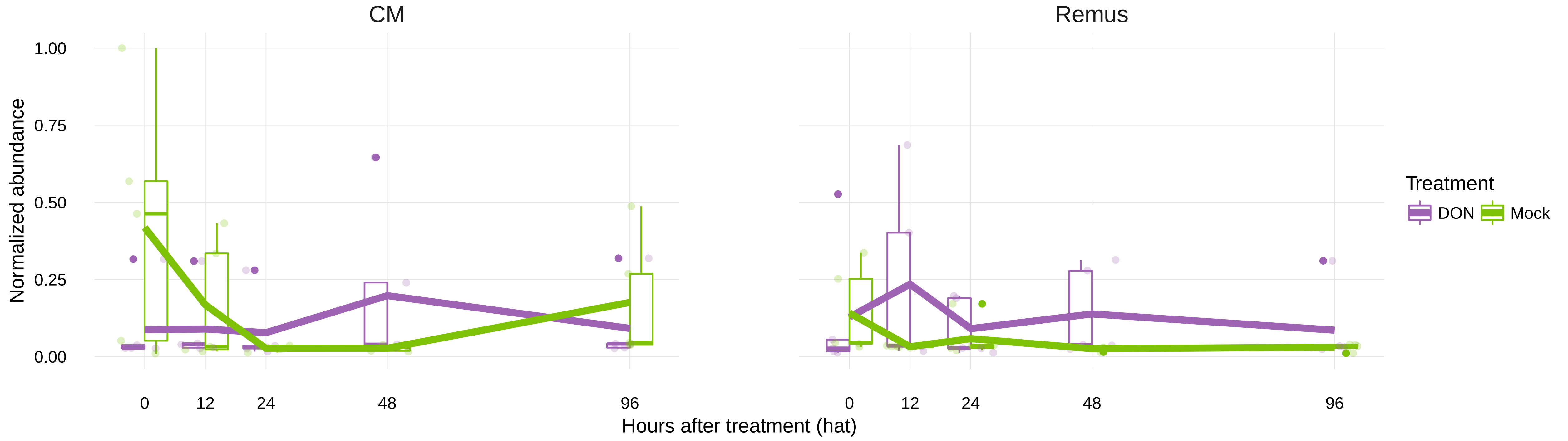

DON, Mock; all four genotypes

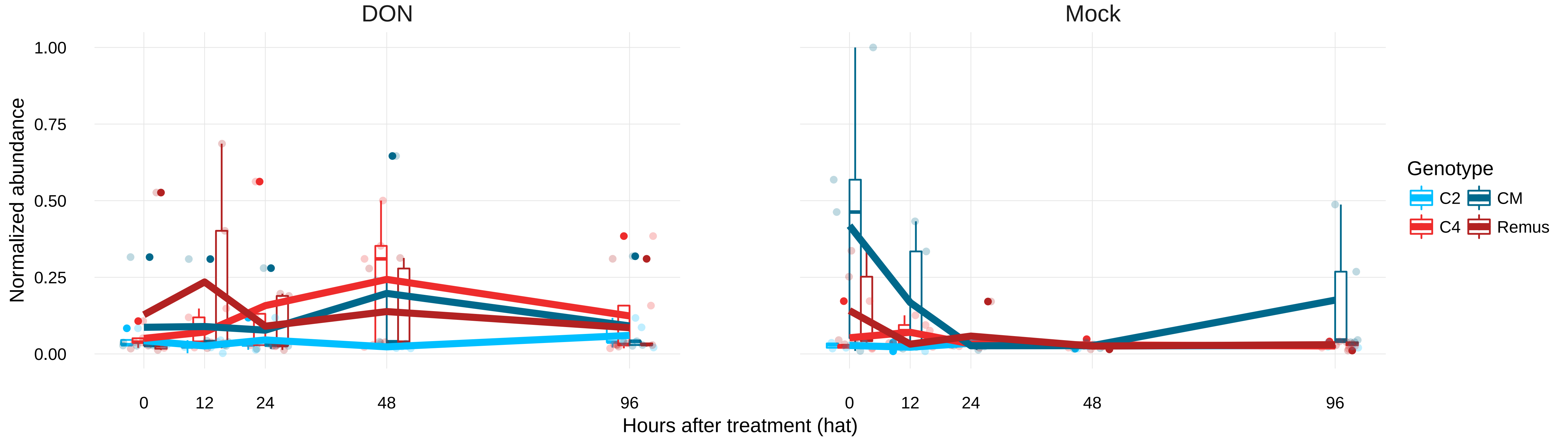

A.112

Annotated as Lignan
(1 database hit)

MZ	549.2118
RT	17.96 min
Normalization	Indirectly via surrogate in the KPX samples
Cluster	–
Cn total / Phe	31 / 18

C2, C4; different treatments

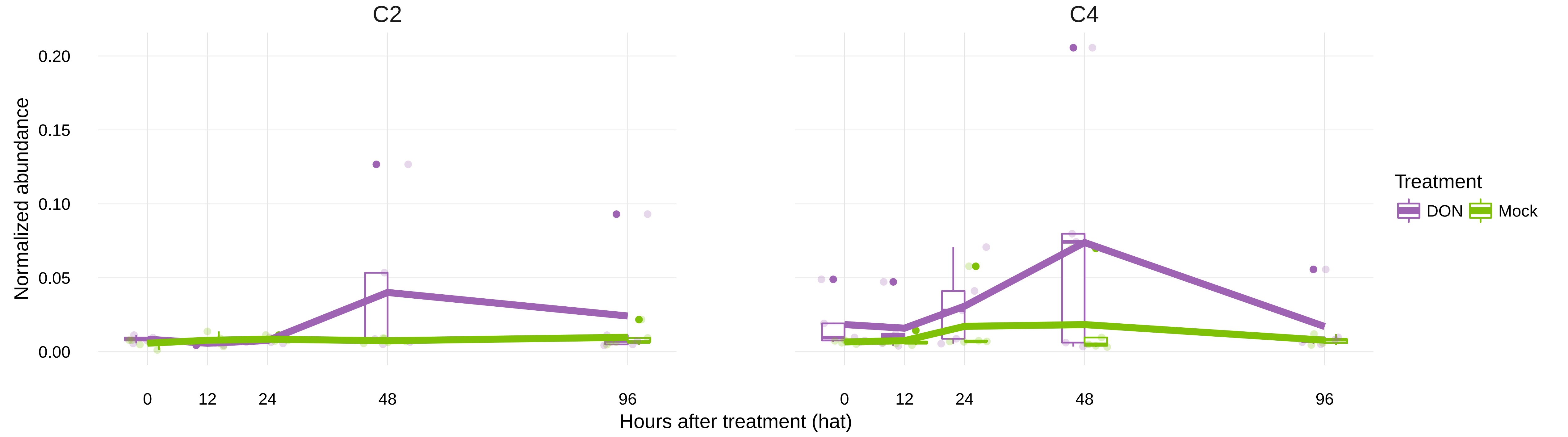

DON, Mock; different genotypes

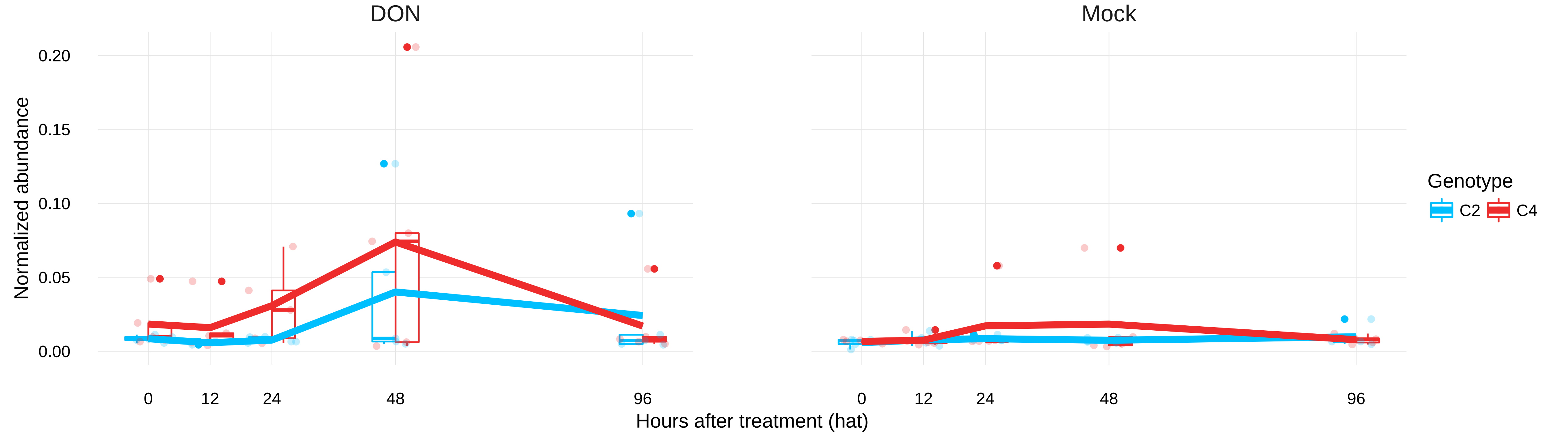

CM, Remus; different treatments

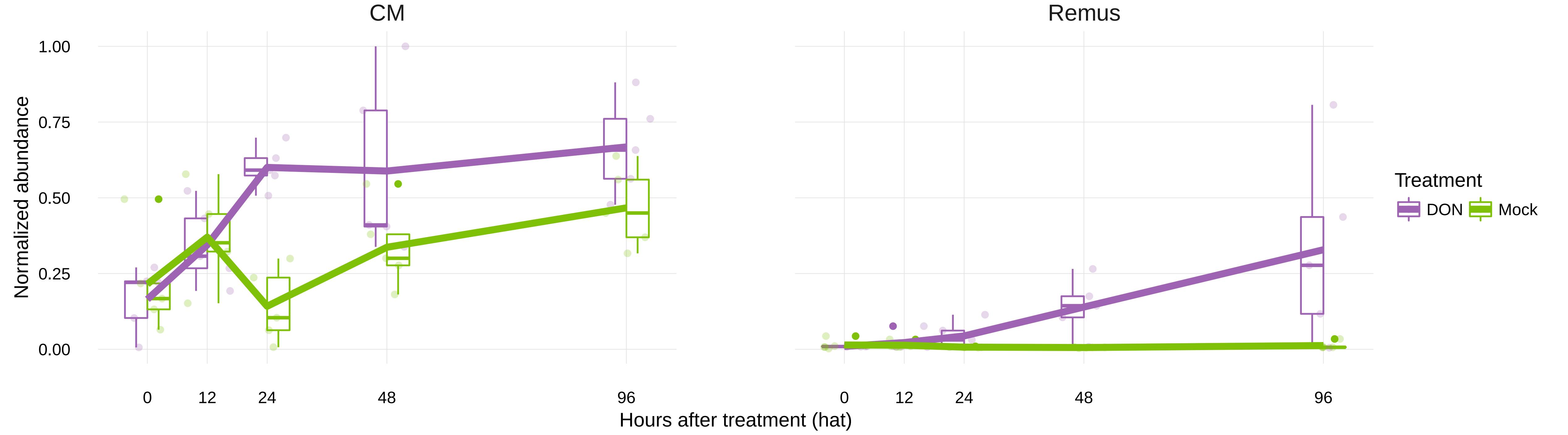

DON, Mock; all four genotypes

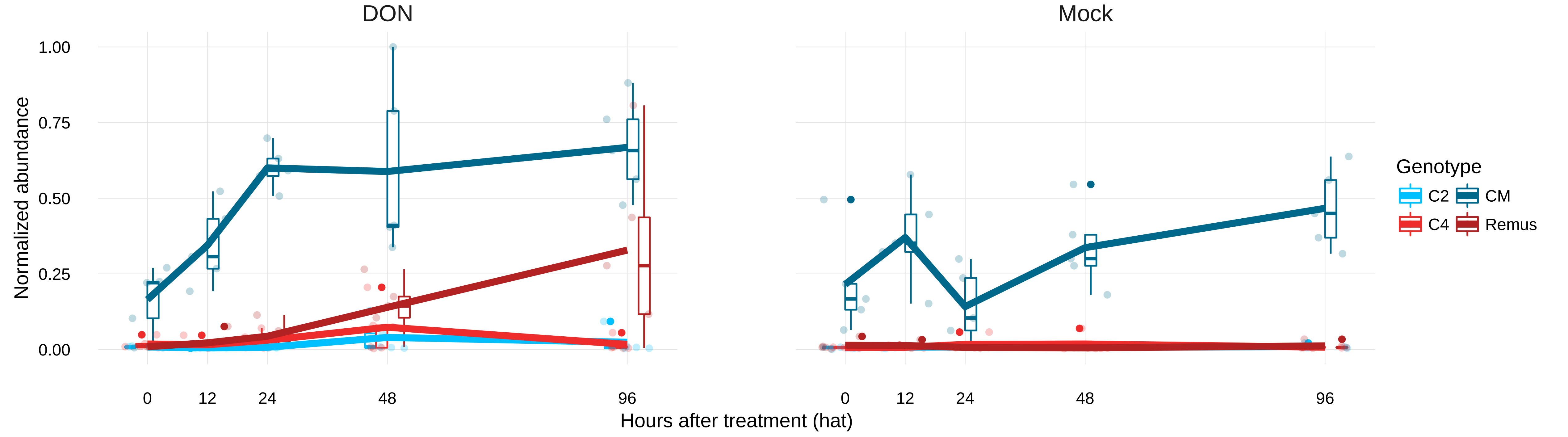

A.54

Annotated as HCA derivative
(1 database hit)

MZ	369.1181
RT	12.26 min
Normalization	Indirectly via surrogate in the KPX samples
Cluster	Cluster 3
Cn total / Phe	17 / 9

C2, C4; different treatments

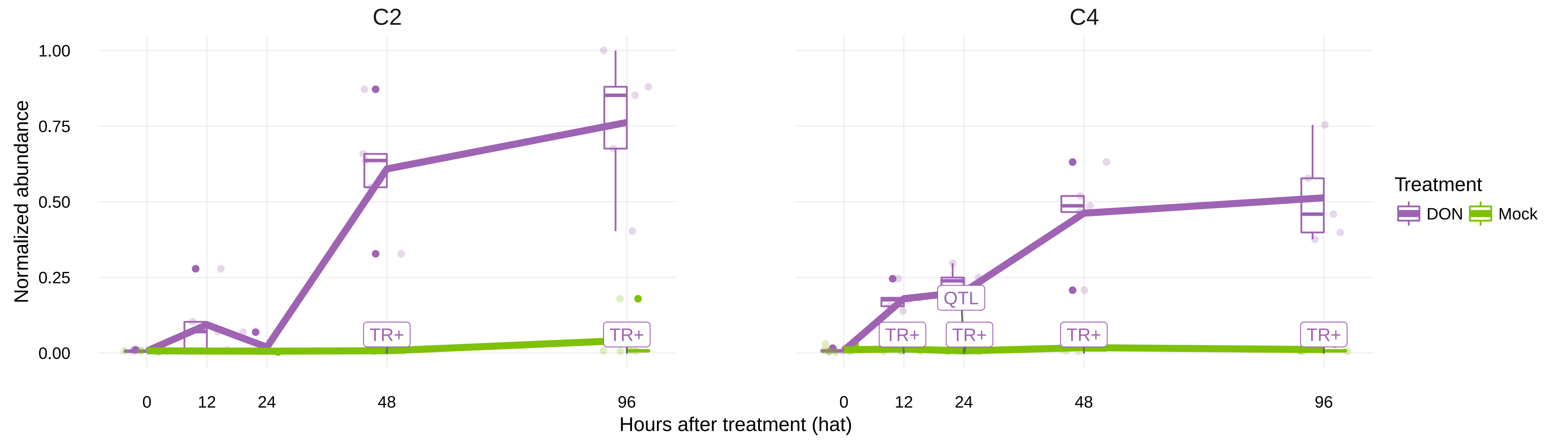

DON, Mock; different genotypes

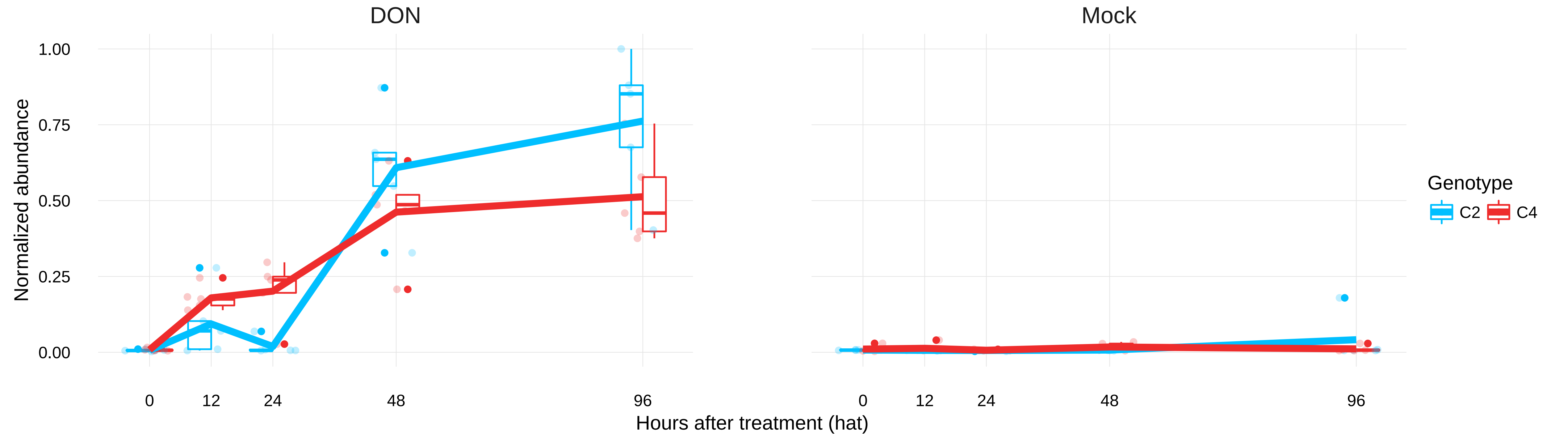

CM, Remus; different treatments

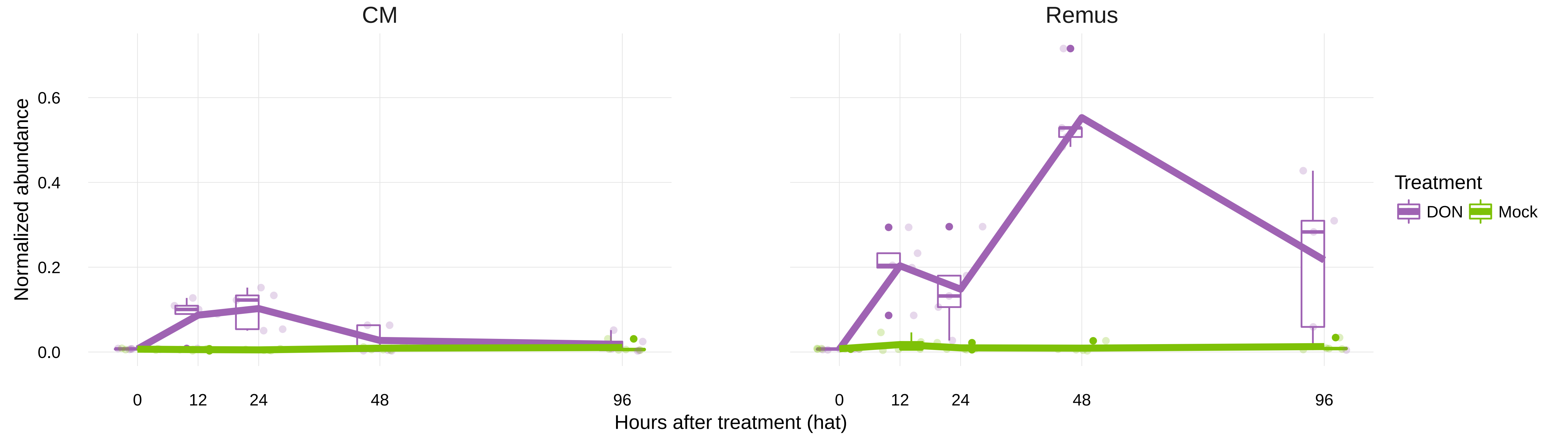

DON, Mock; all four genotypes

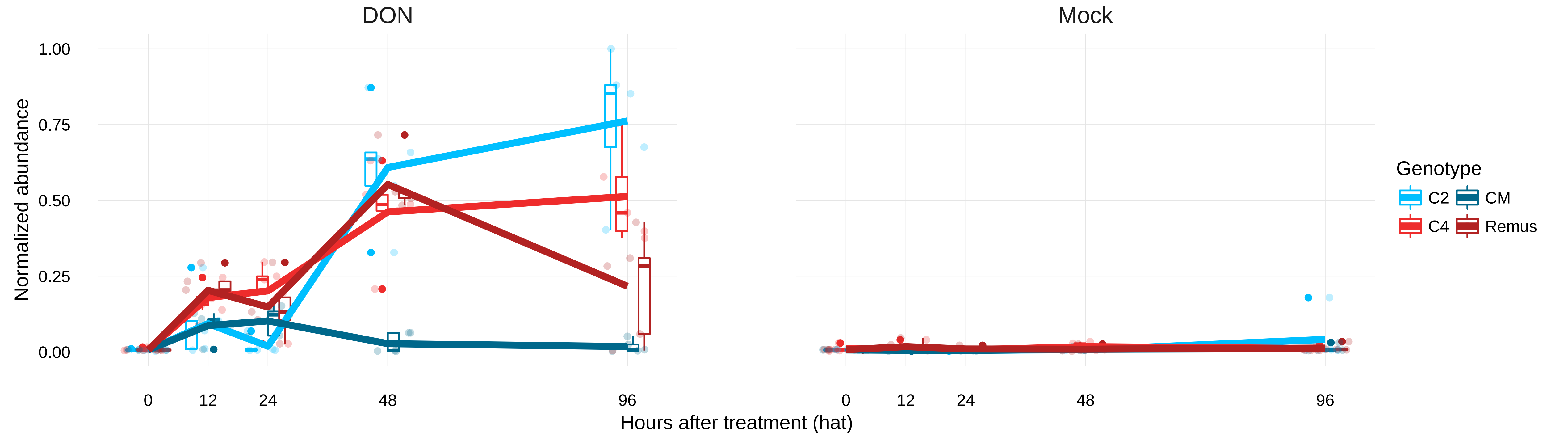

A.32

Annotated as HCA derivative

(1 database hit)

MZ	391.1005
RT	9.63 min
Normalization	Directly via KPX samples
Cluster	Cluster 3
Cn total / Phe	9 / 9

C2, C4; different treatments

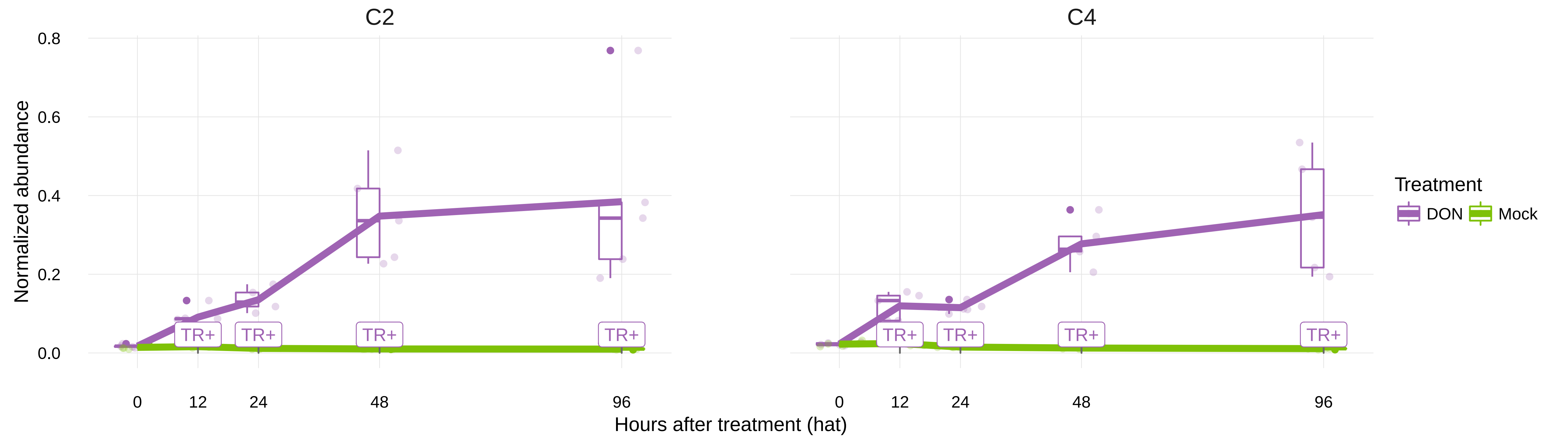

DON, Mock; different genotypes

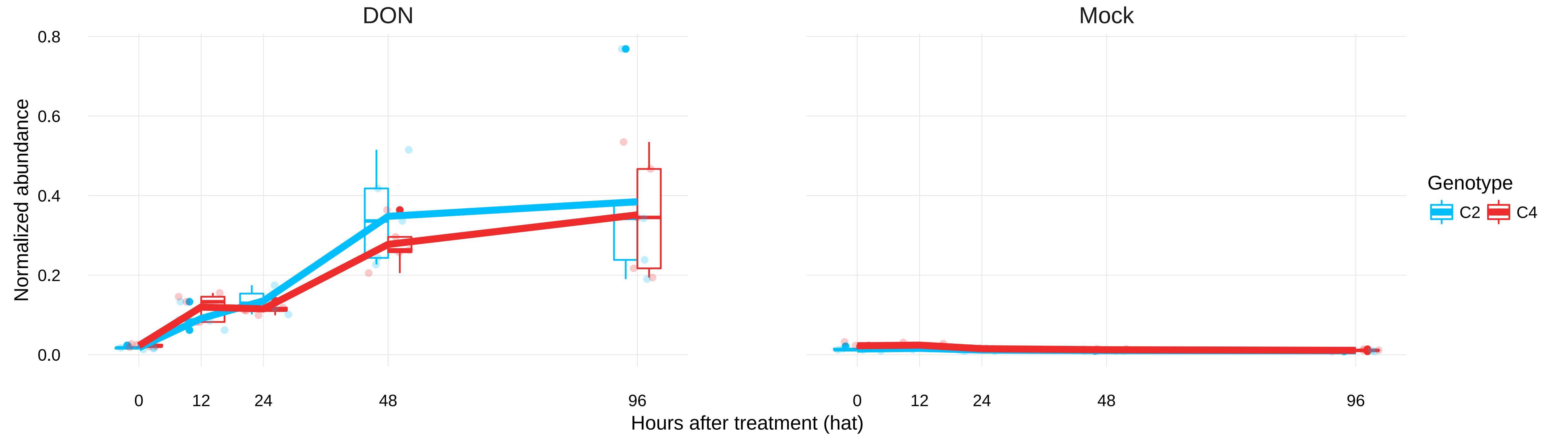

CM, Remus; different treatments

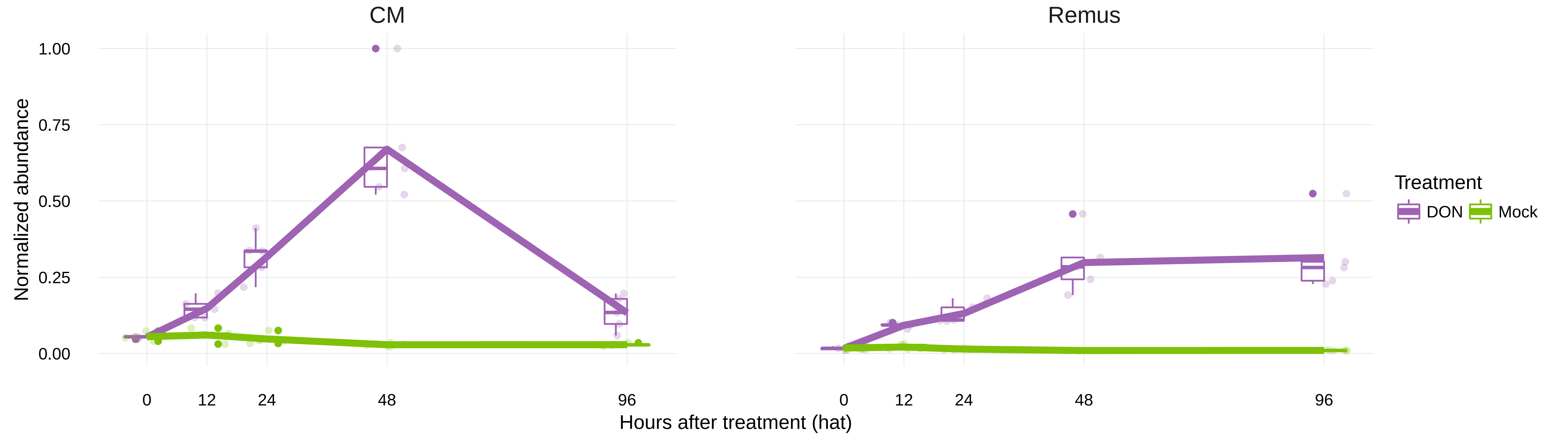

DON, Mock; all four genotypes

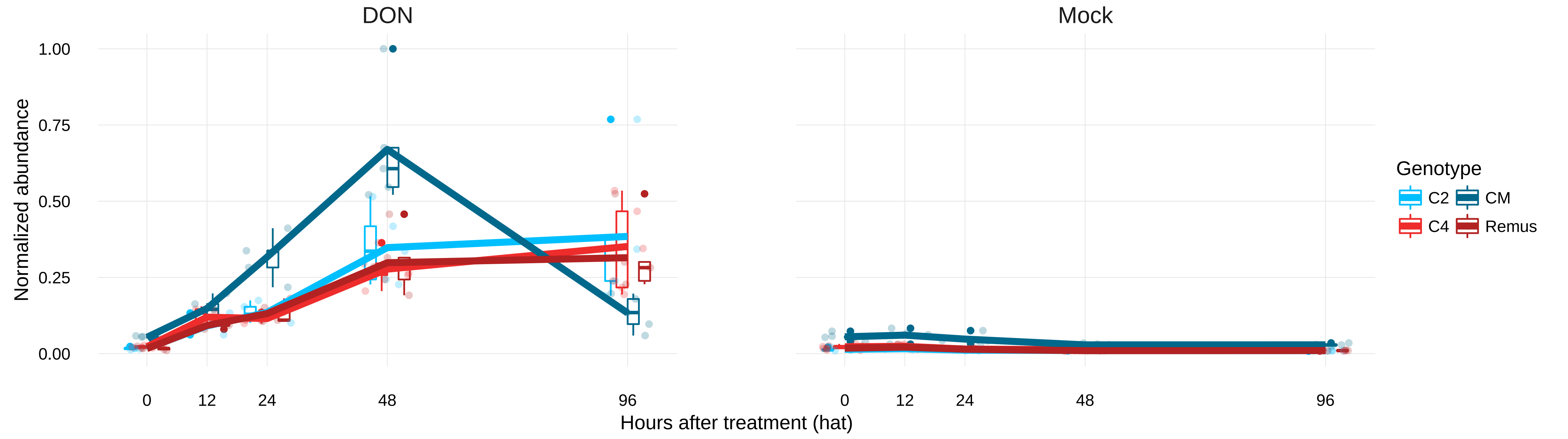

A.35

Annotated as others (dipeptide)
(1 database hit)

MZ	295.1289
RT	10.22 min
Normalization	Directly via KPX samples
Cluster	Cluster 3
Cn total / Phe	14 / 9

C2, C4; different treatments

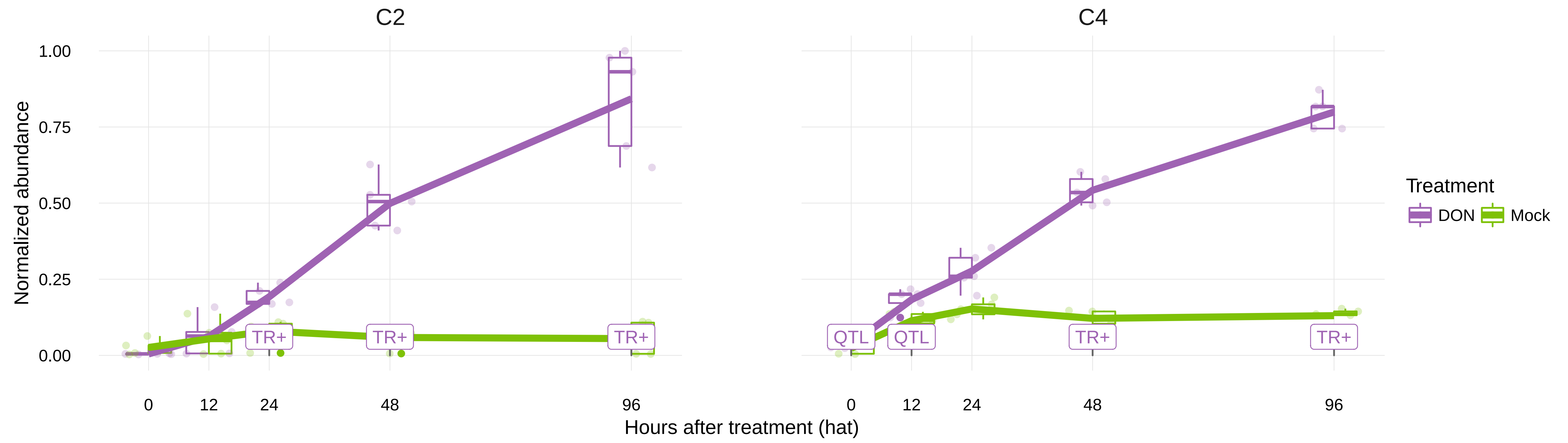

DON, Mock; different genotypes

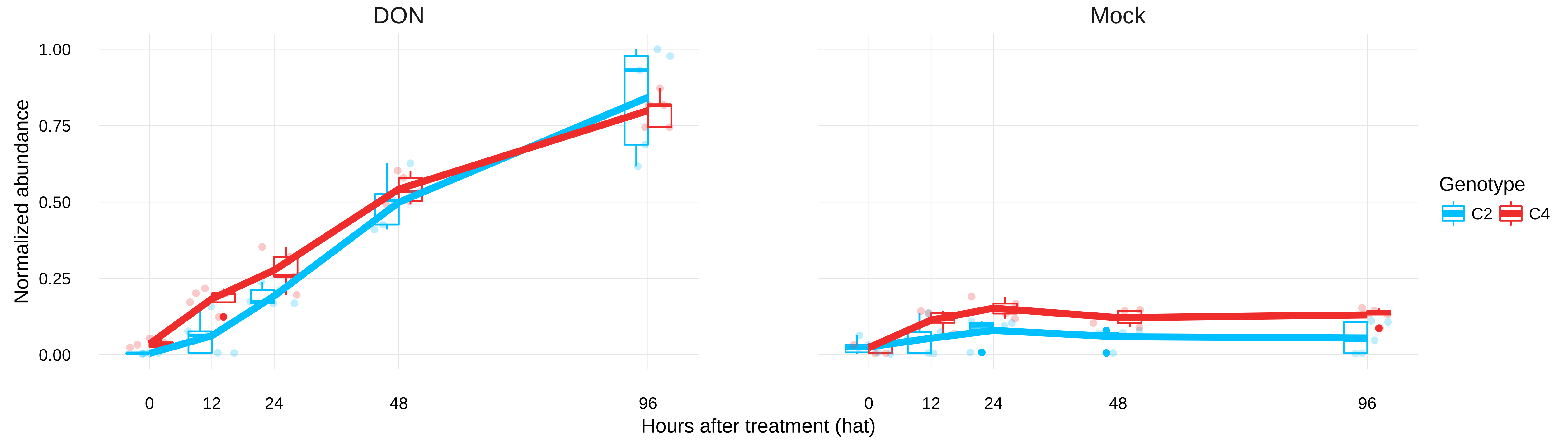

CM, Remus; different treatments

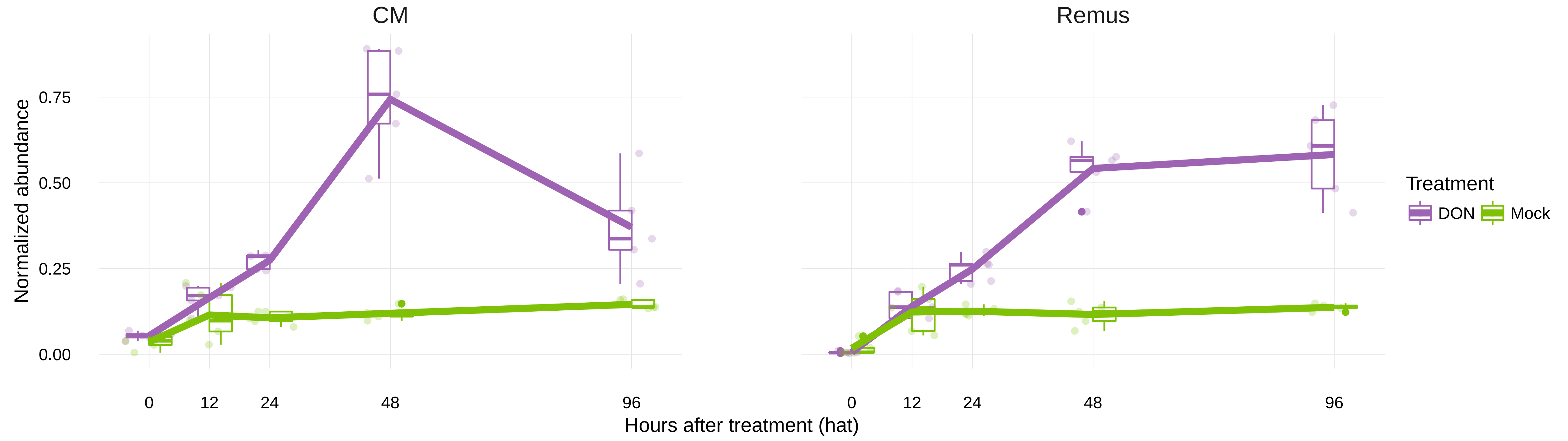

DON, Mock; all four genotypes

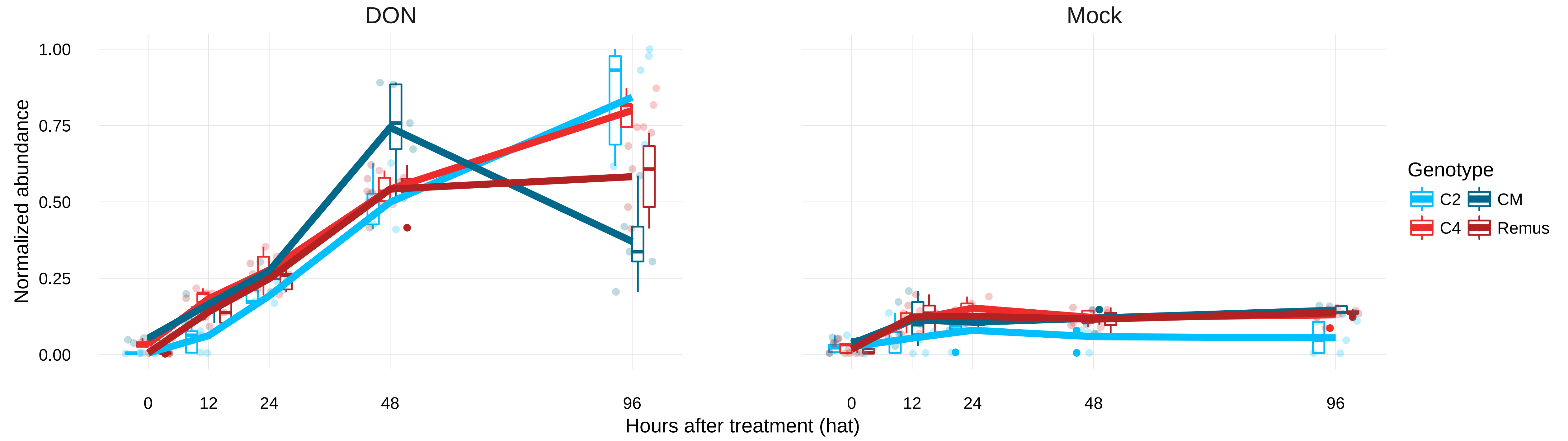

A.40

Annotated as HCA derivative
(1 database hit)

MZ	379.1009
RT	10.36 min
Normalization	Directly via KPX samples
Cluster	Cluster 3
Cn total / Phe	9 / 9

C2, C4; different treatments

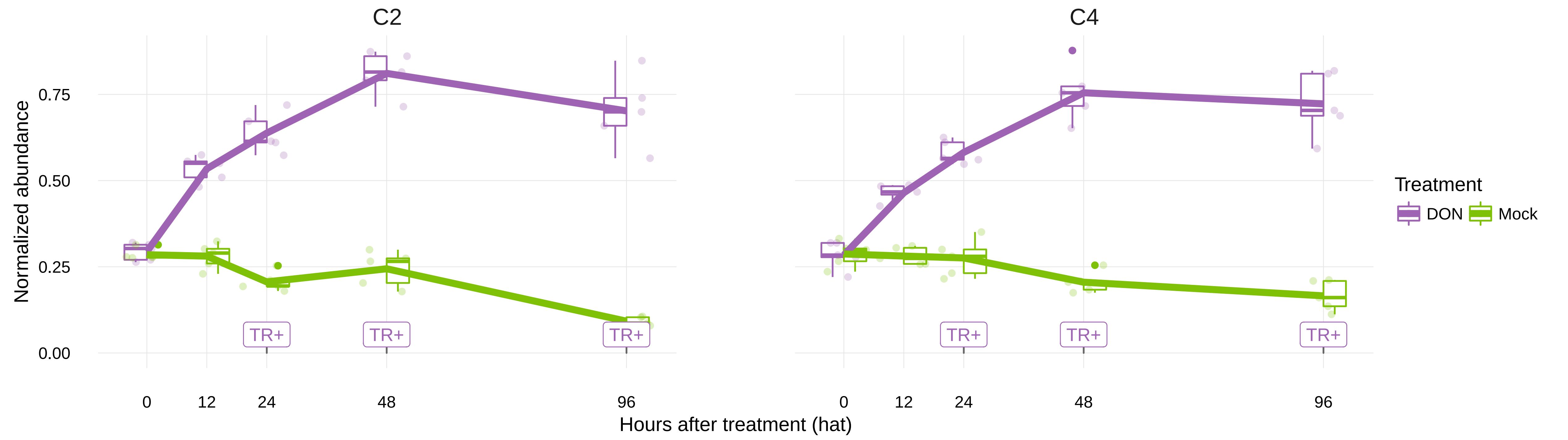

DON, Mock; different genotypes

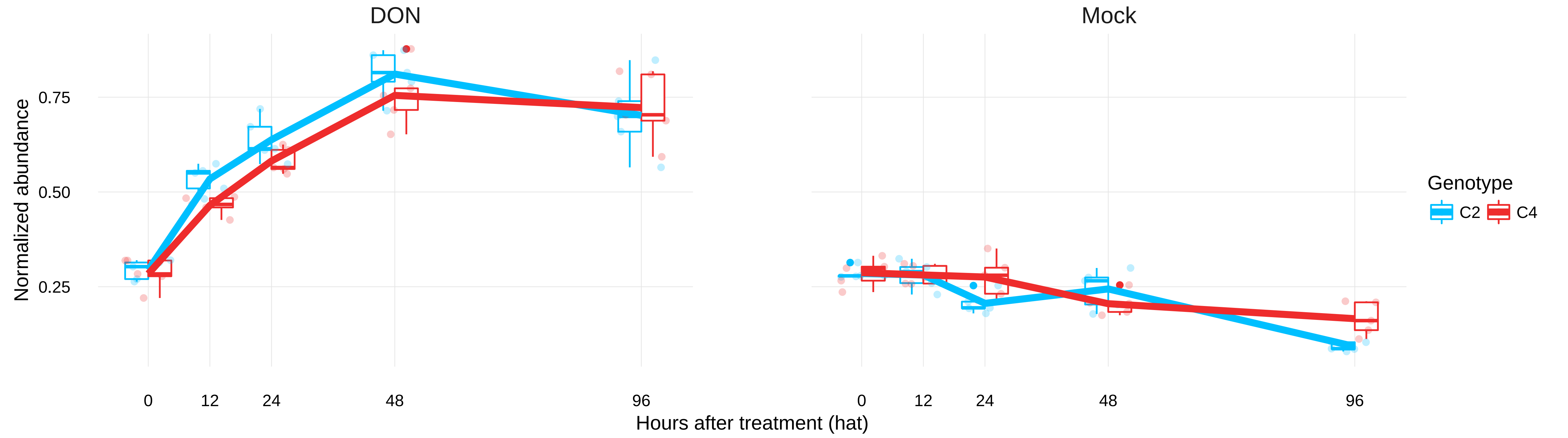

CM, Remus; different treatments

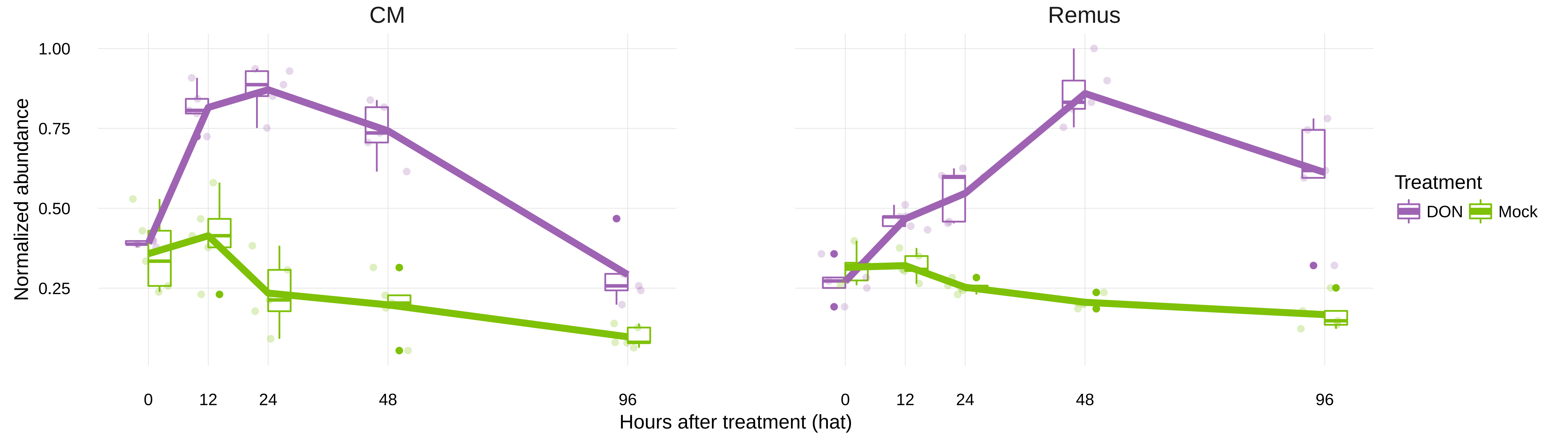

DON, Mock; all four genotypes

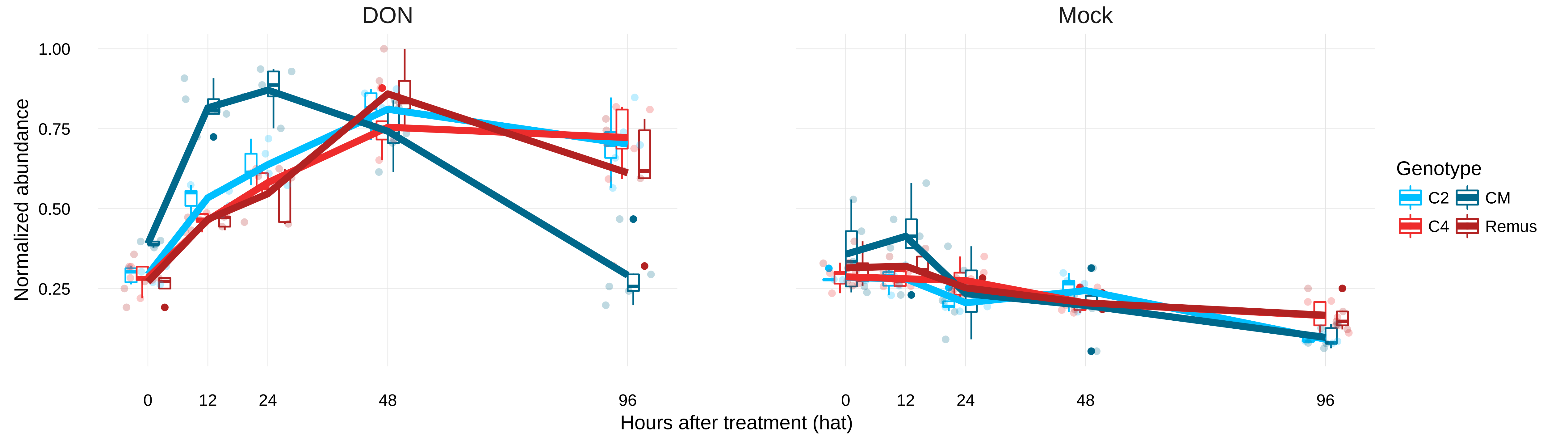

A.30

Annotated as HCA derivative
(1 database hit)

MZ	369.1183
RT	9.54 min
Normalization	Indirectly via surrogate in the KPX samples
Cluster	Cluster 3
Cn total / Phe	17 / 9

C2, C4; different treatments

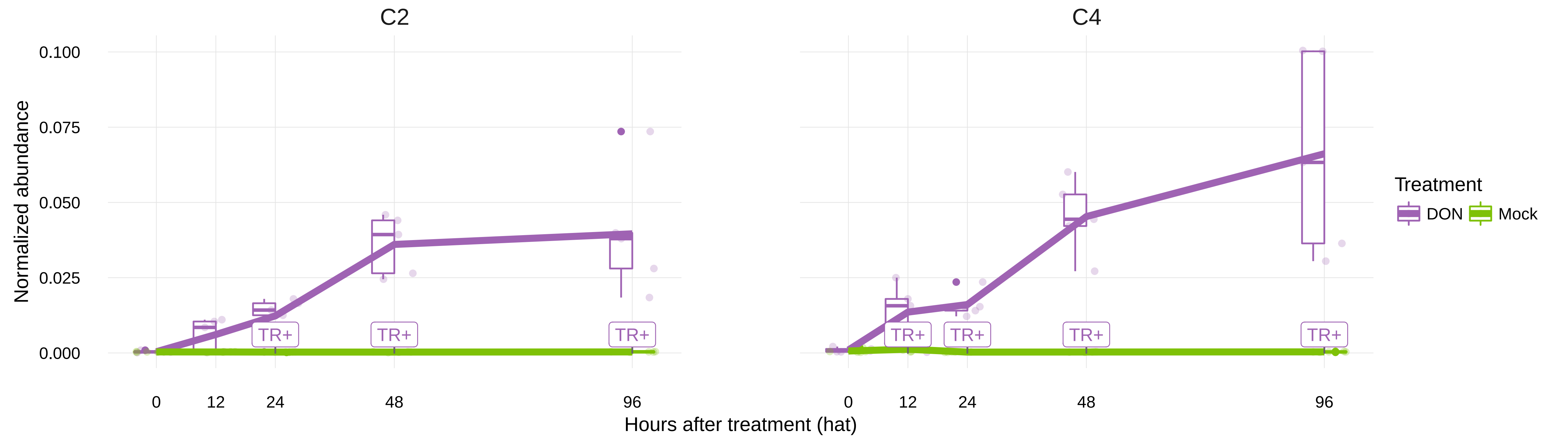

DON, Mock; different genotypes

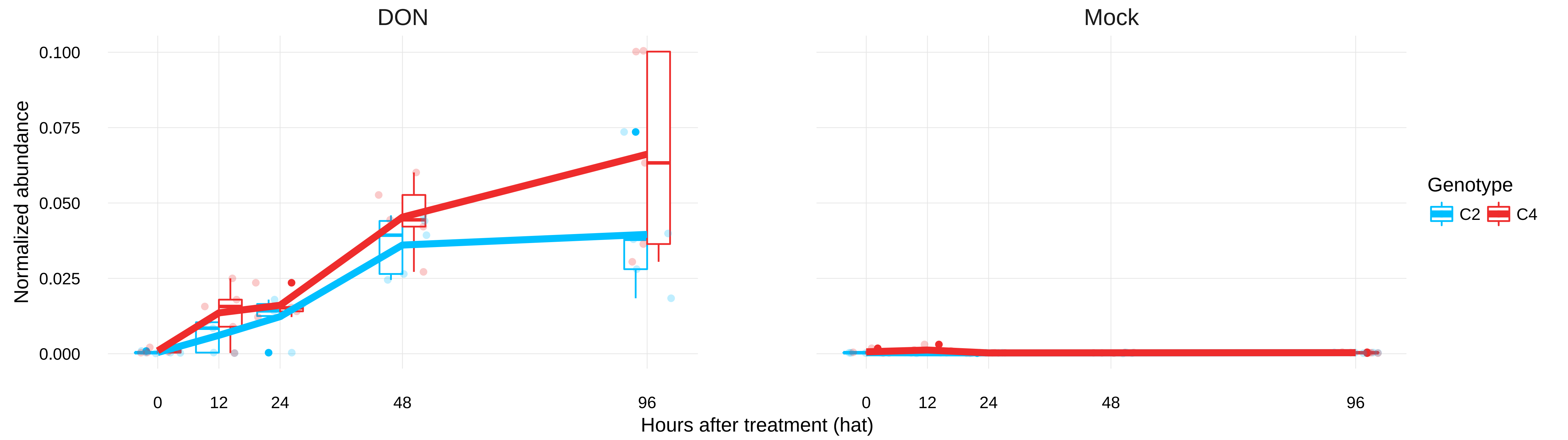

CM, Remus; different treatments

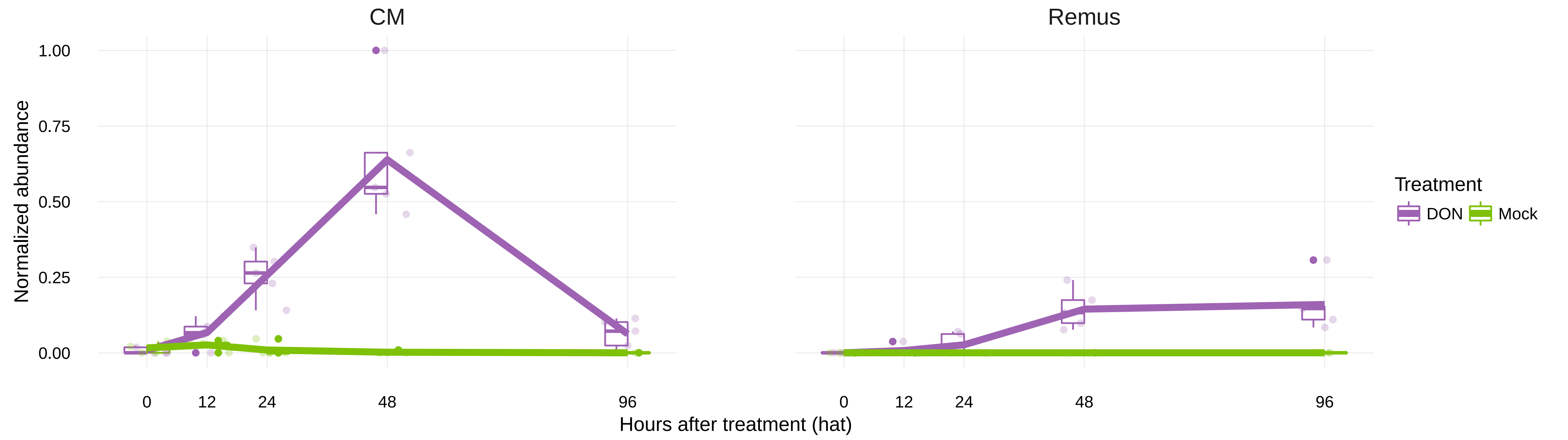

DON, Mock; all four genotypes

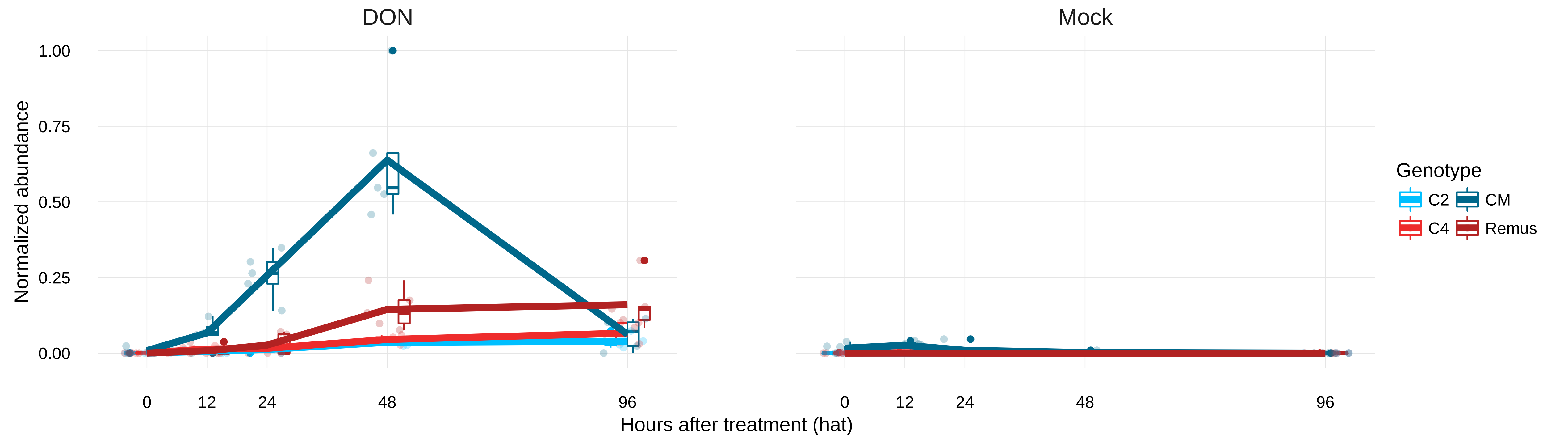

A.29

Annotated as HCA derivative

(3 database hits)

MZ	195.0653
RT	9.51 min
Normalization	Directly via KPX samples
Cluster	Cluster 3
Cn total / Phe	10 /

C2, C4; different treatments

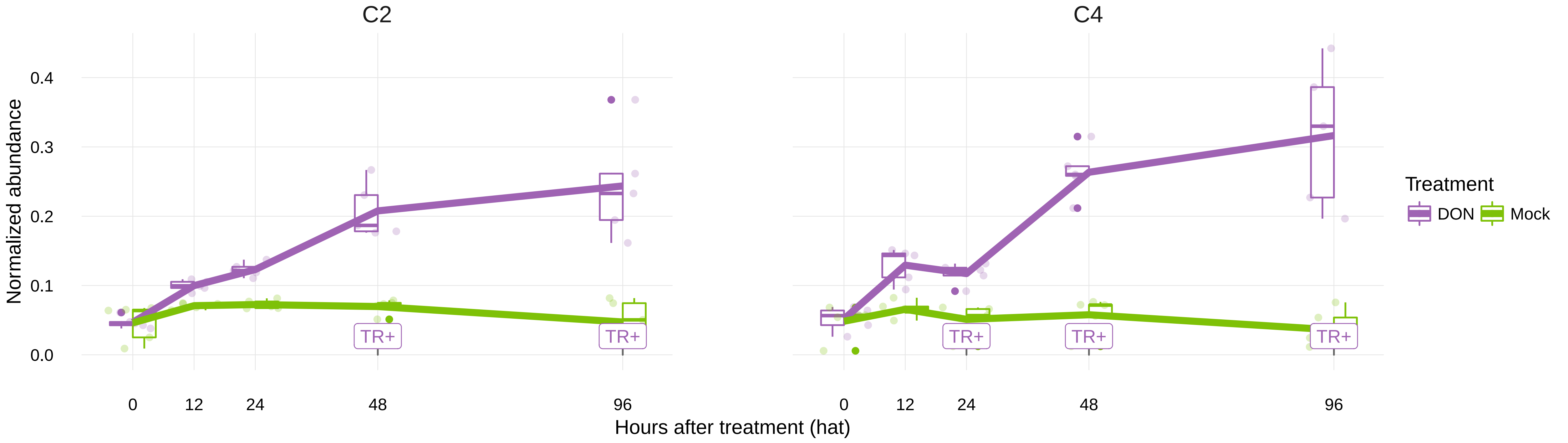

DON, Mock; different genotypes

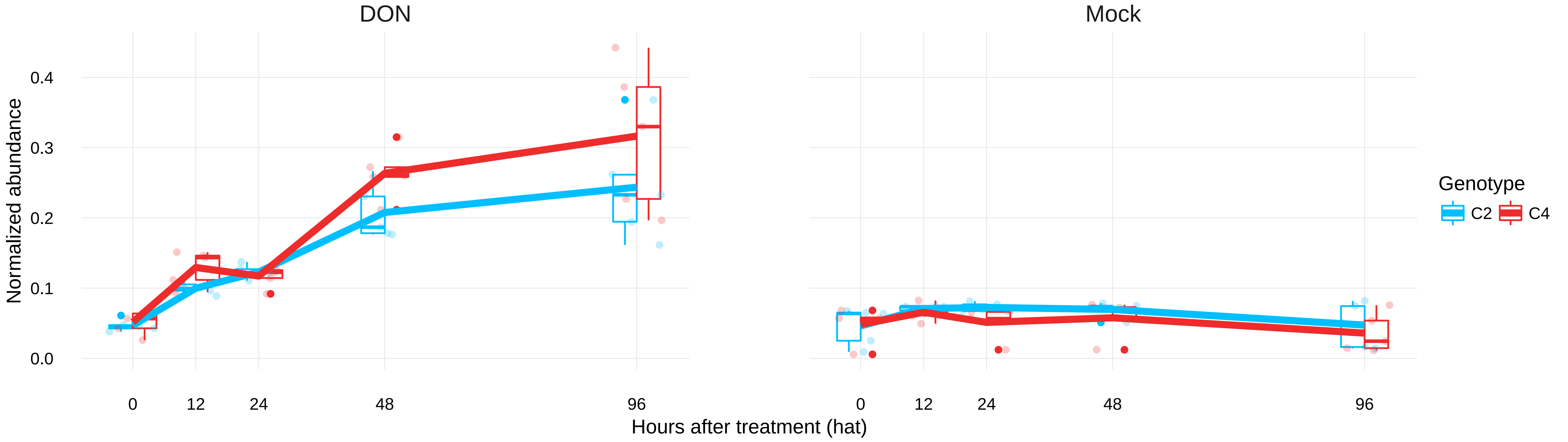

CM, Remus; different treatments

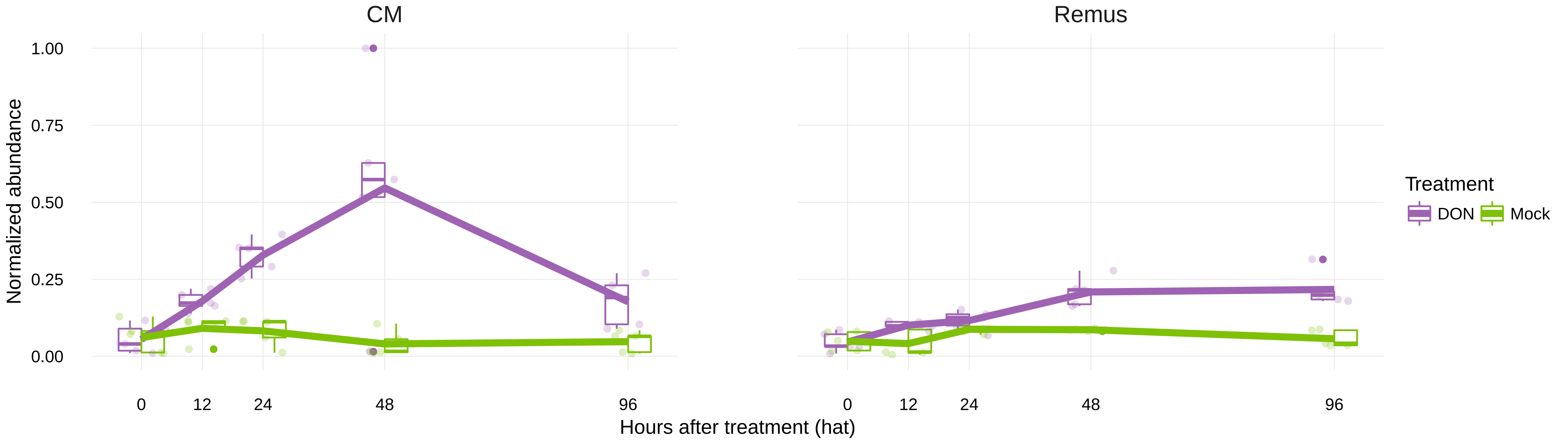

DON, Mock; all four genotypes

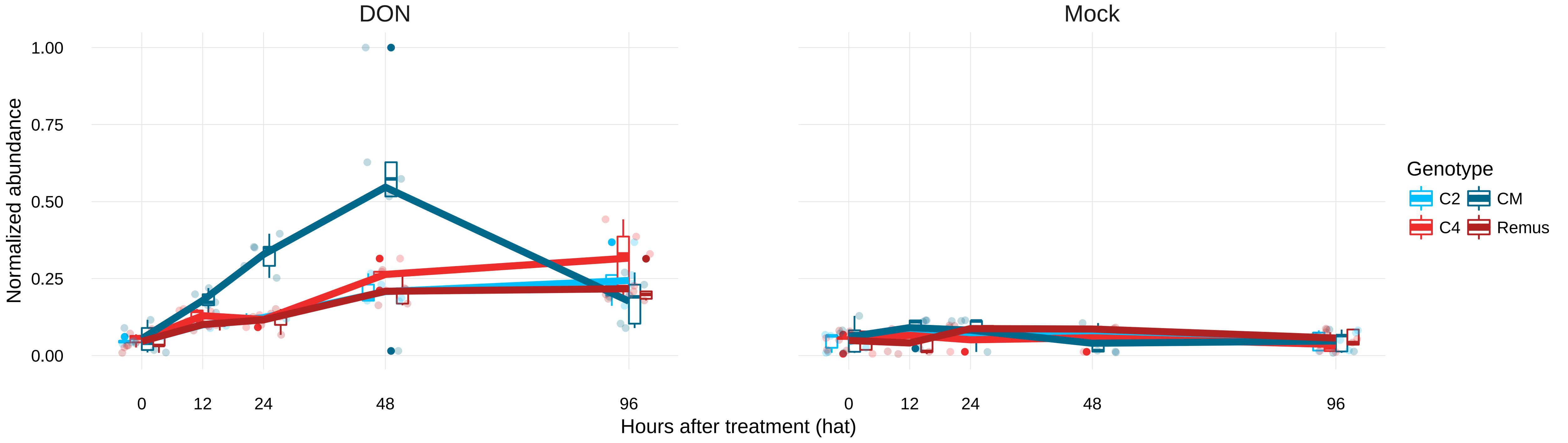

A.38

Annotated as HCA derivative
(3 database hits)

MZ	195.0653
RT	10.29 min
Normalization	Directly via KPX samples
Cluster	Cluster 3
Cn total / Phe	10 / 9

C2, C4; different treatments

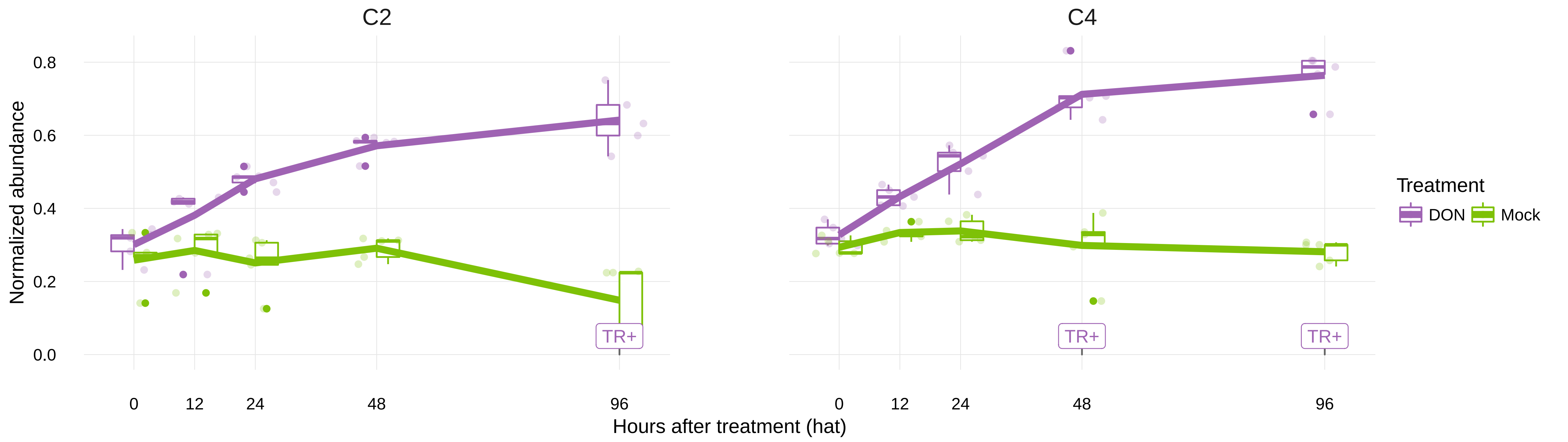

DON, Mock; different genotypes

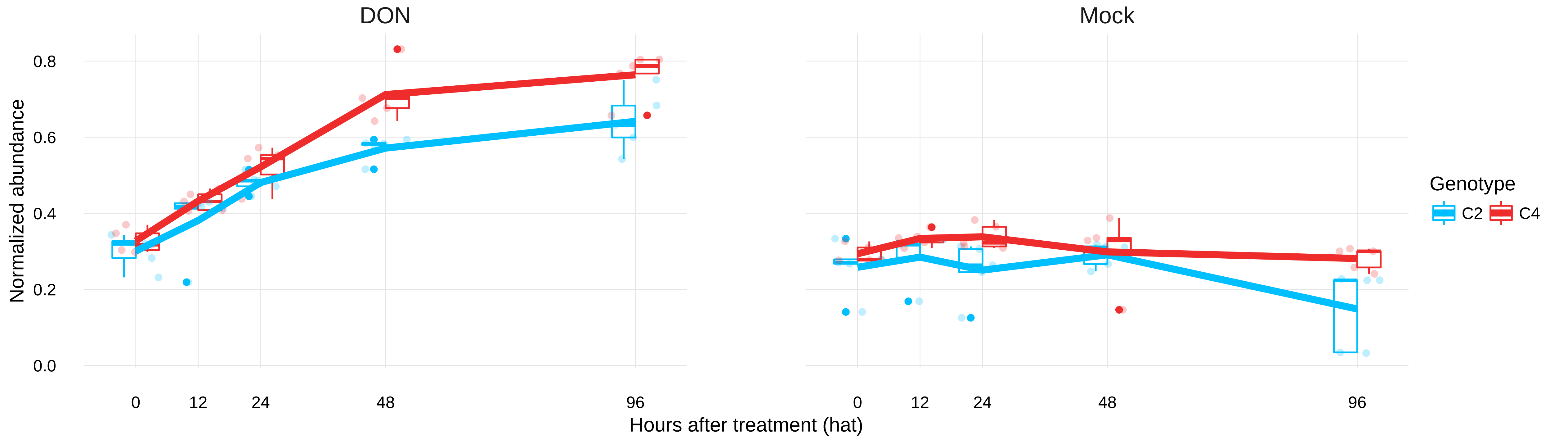

CM, Remus; different treatments

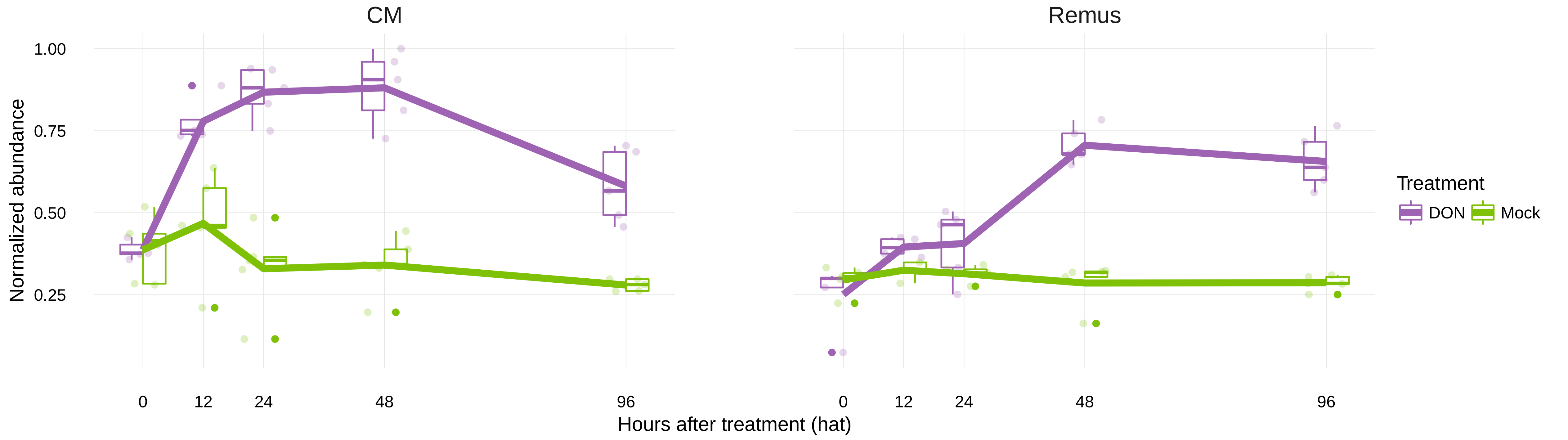

DON, Mock; all four genotypes

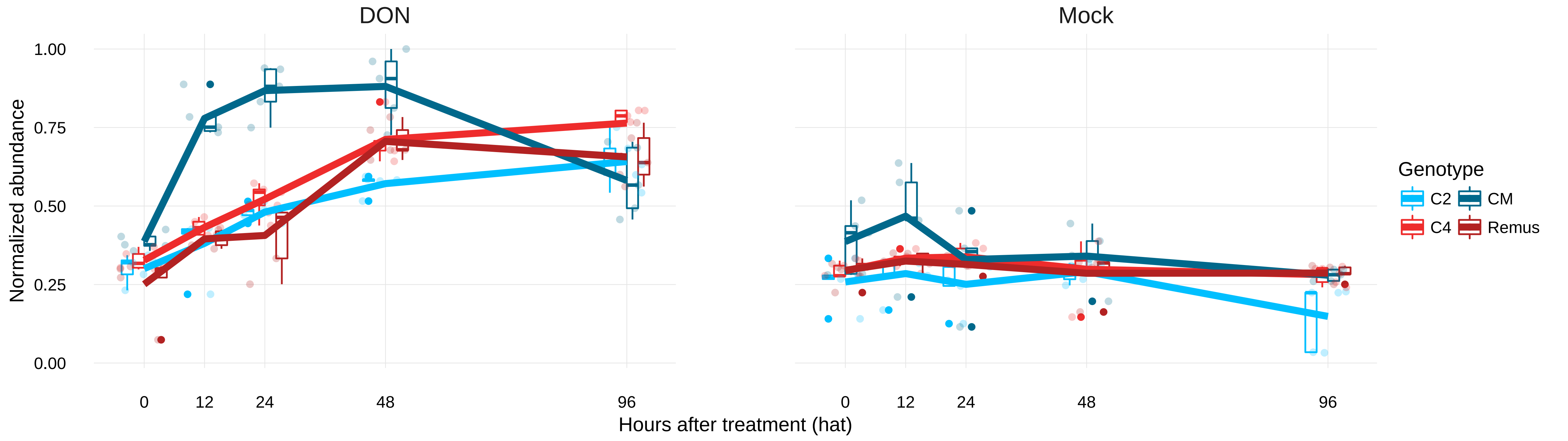

A.60

Annotated as Flavonoid or Lignan
(10 database hits)

MZ	359.1489
RT	12.8 min
Normalization	Directly via KPX samples
Cluster	Cluster 3
Cn total / Phe	20 /

C2, C4; different treatments

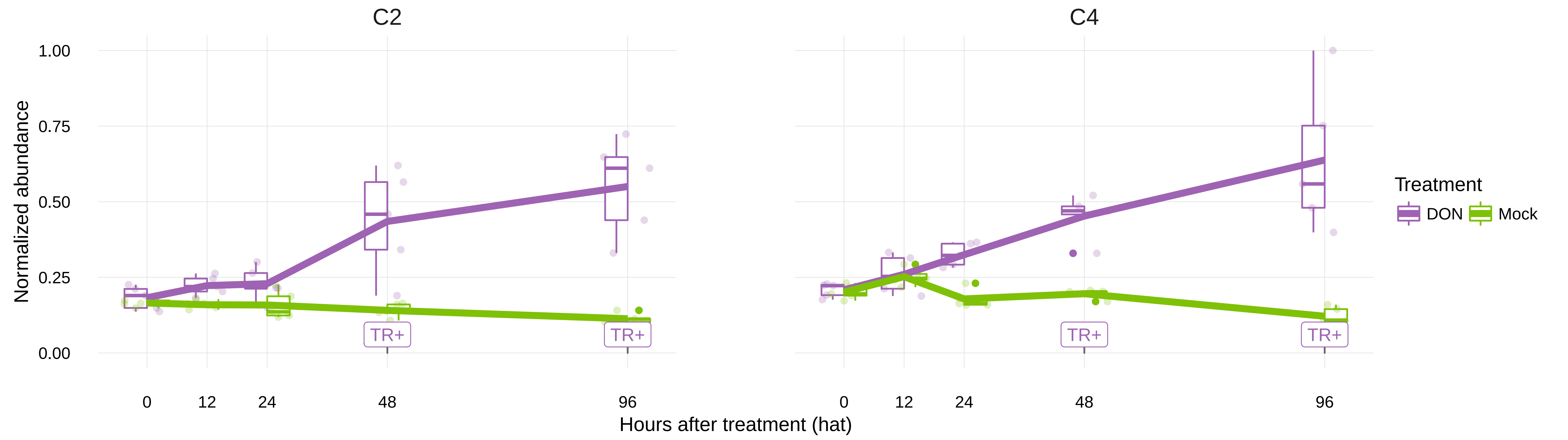

DON, Mock; different genotypes

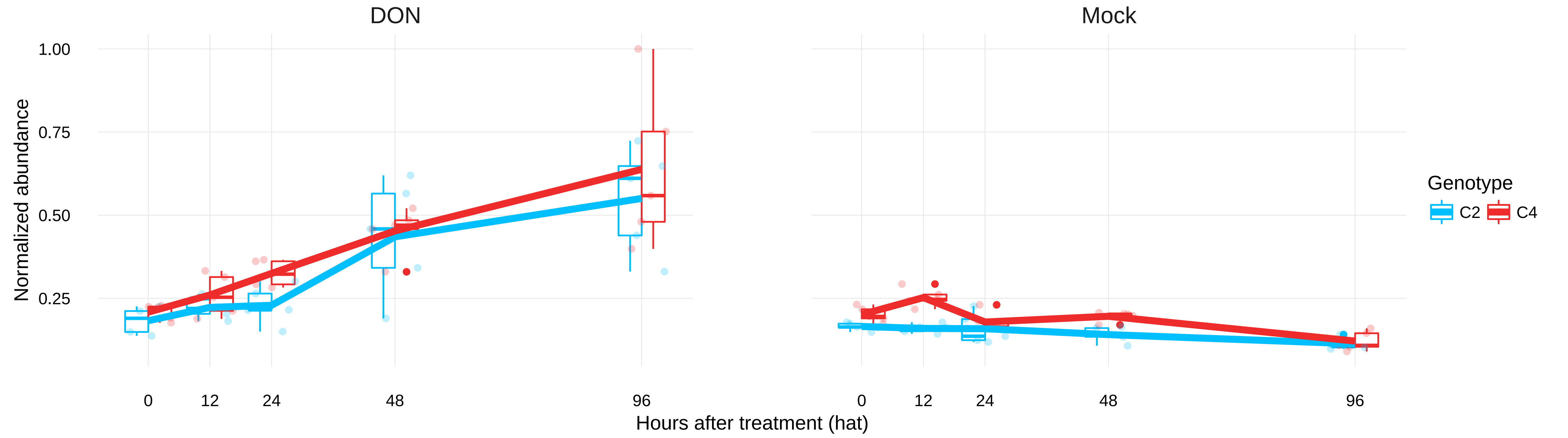

CM, Remus; different treatments

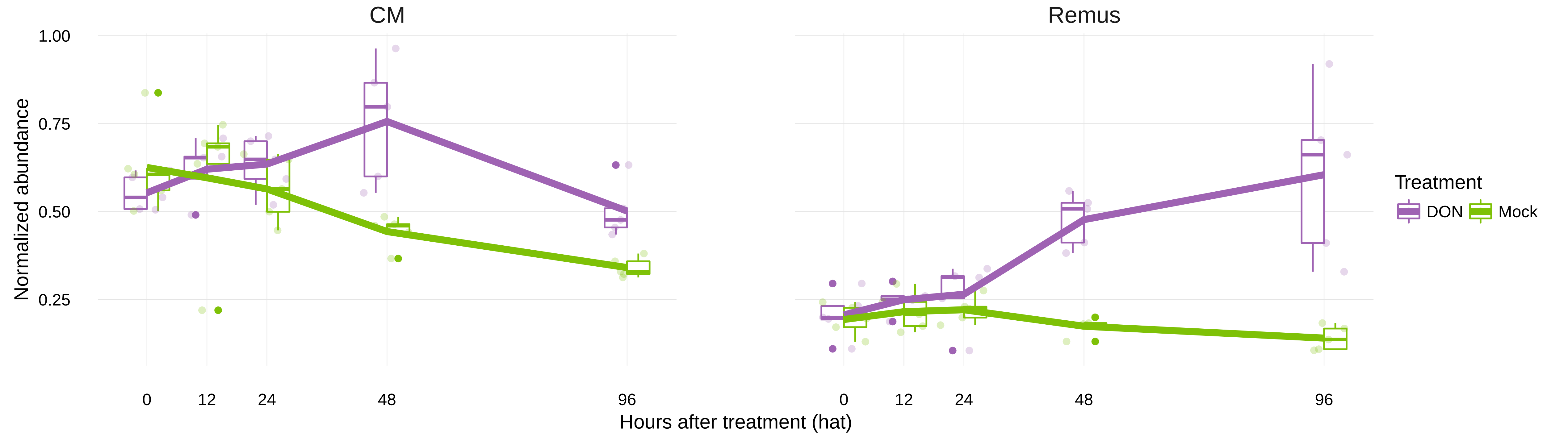

DON, Mock; all four genotypes

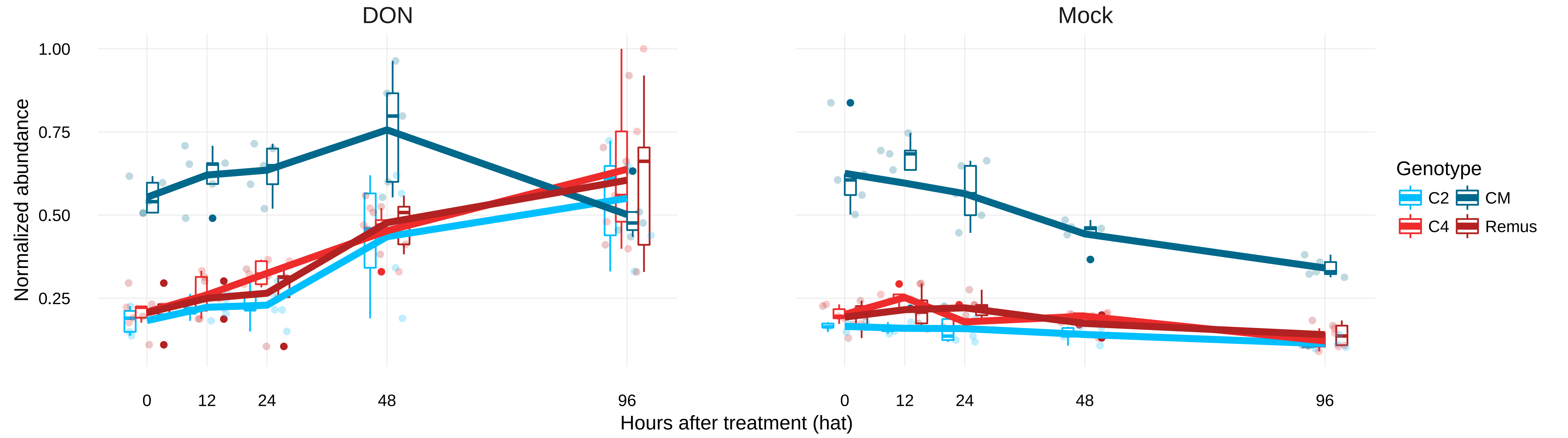

A.23

Annotated as HCA derivative
(1 database hit)

MZ	365.1209
RT	8.71 min
Normalization	Directly via KPX samples
Cluster	Cluster 3
Cn total / Phe	16 /

C2, C4; different treatments

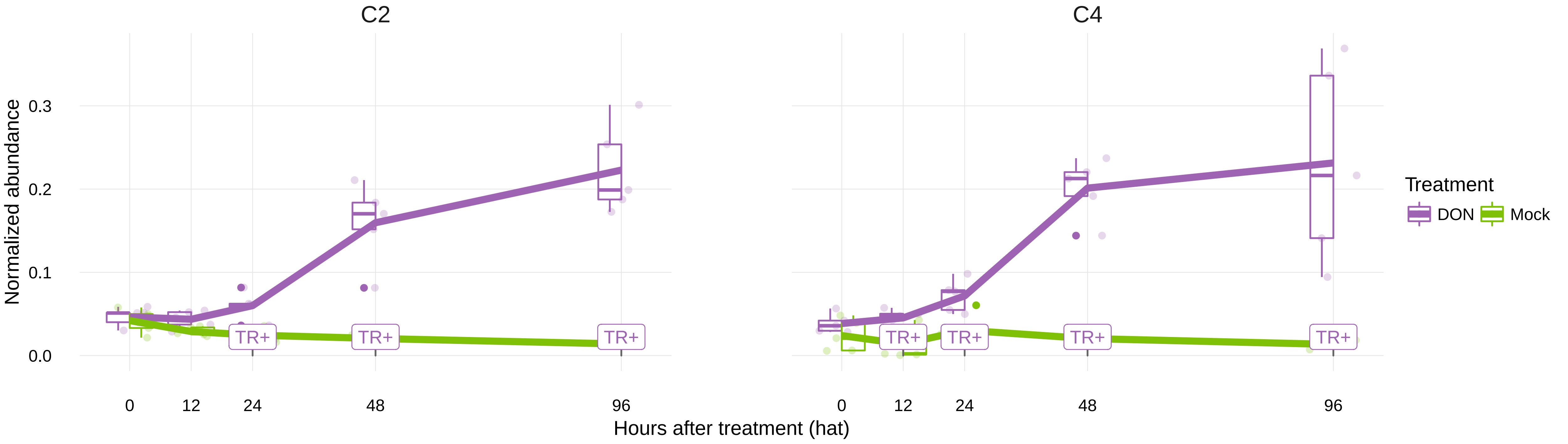

DON, Mock; different genotypes

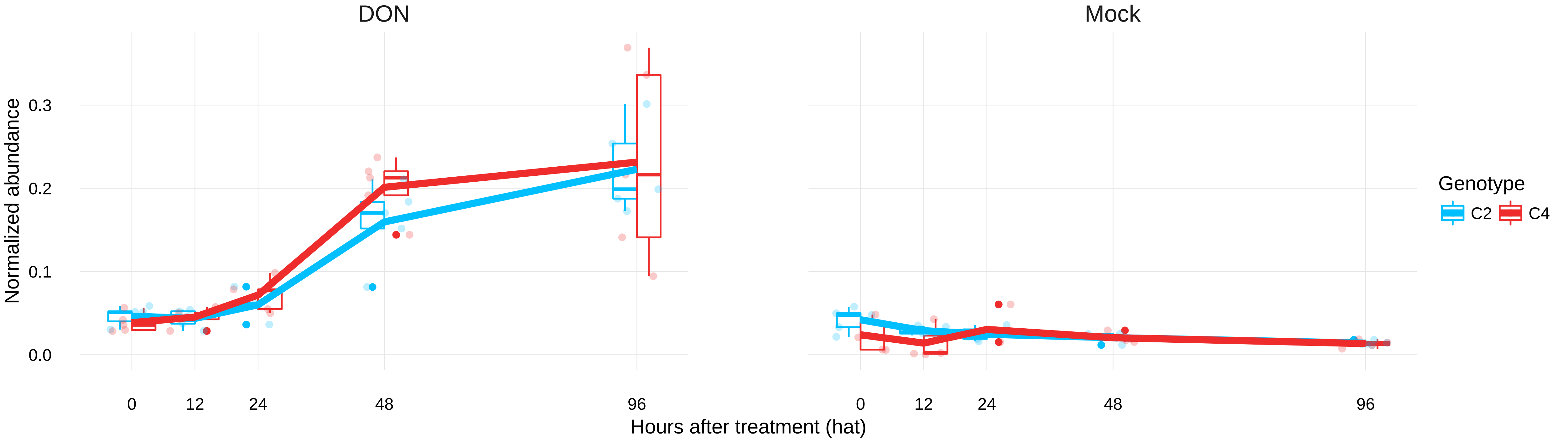

CM, Remus; different treatments

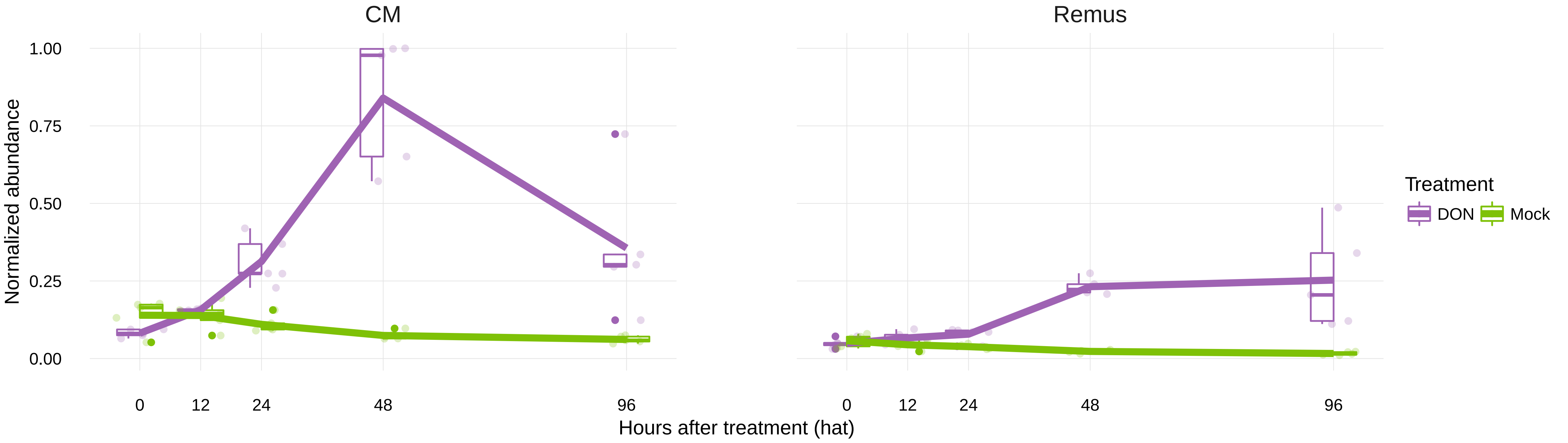

DON, Mock; all four genotypes

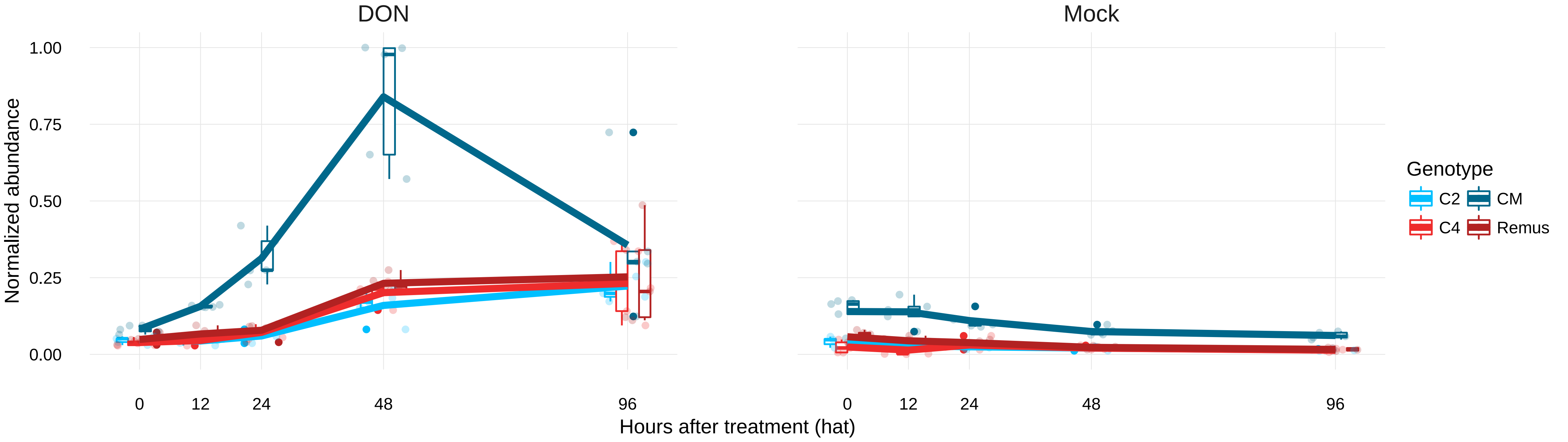

A.122

Annotated as HCA derivative
(2 database hits)

MZ	838.2766
RT	19.76 min
Normalization	Indirectly via surrogate in the KPX samples
Cluster	Cluster 3
Cn total / Phe	38 / 9

C2, C4; different treatments

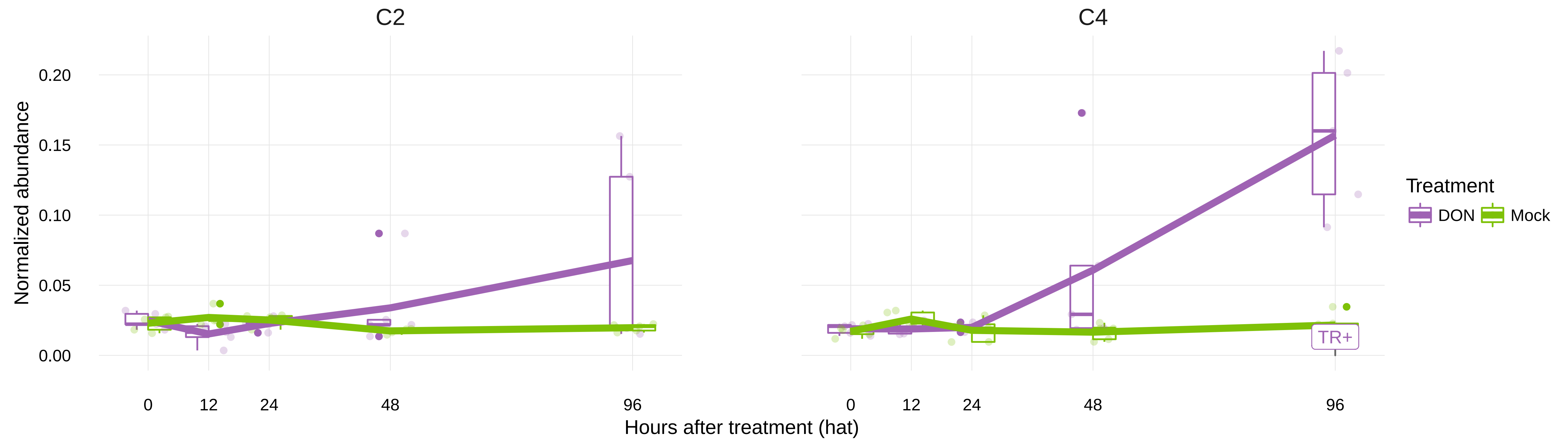

DON, Mock; different genotypes

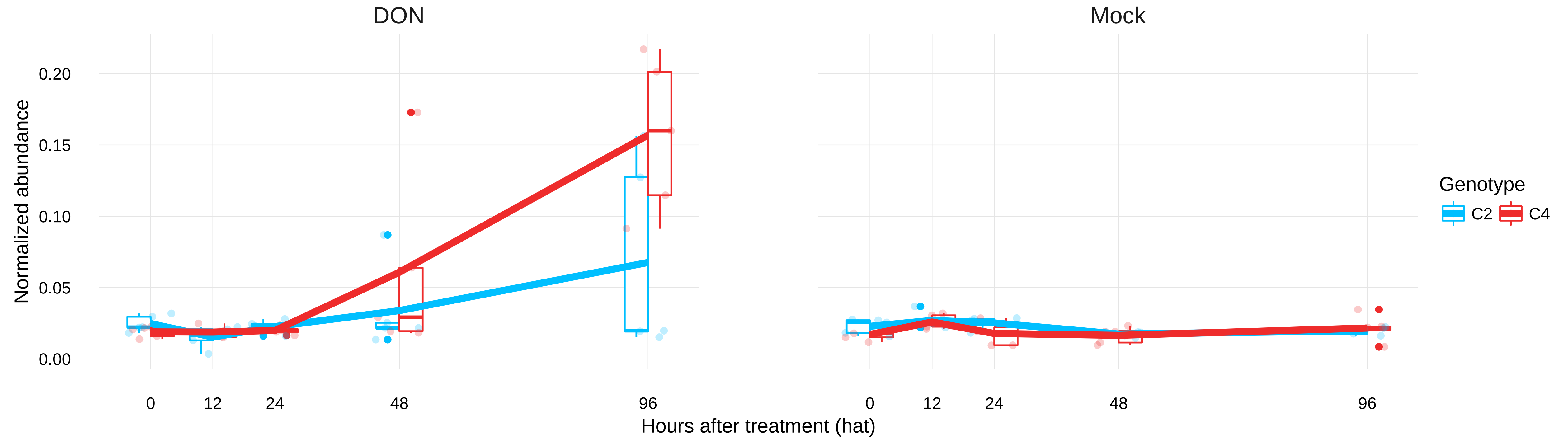

CM, Remus; different treatments

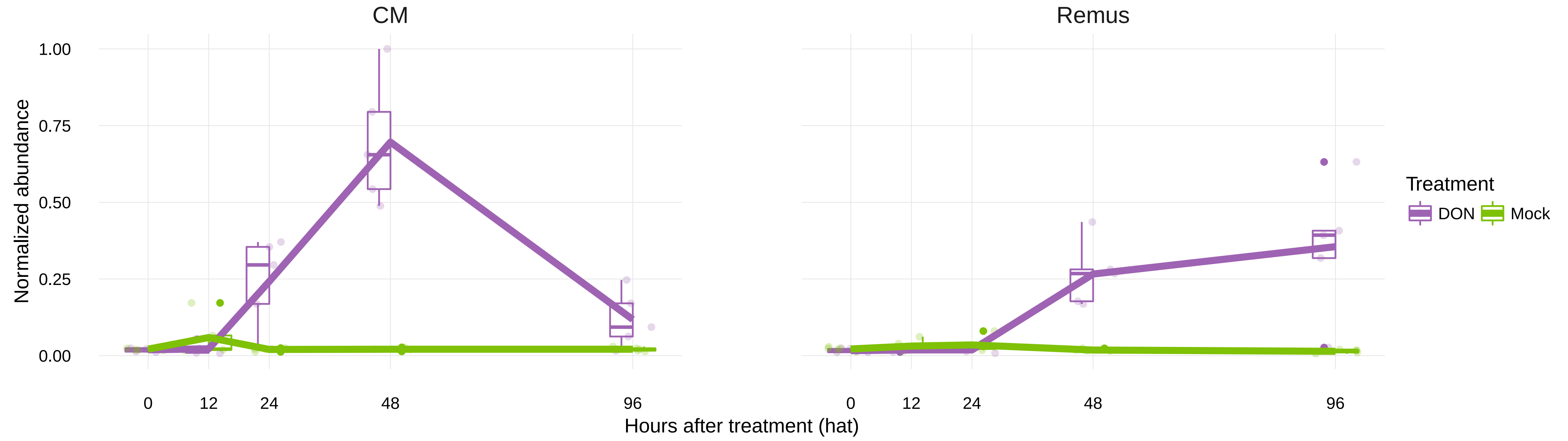

DON, Mock; all four genotypes

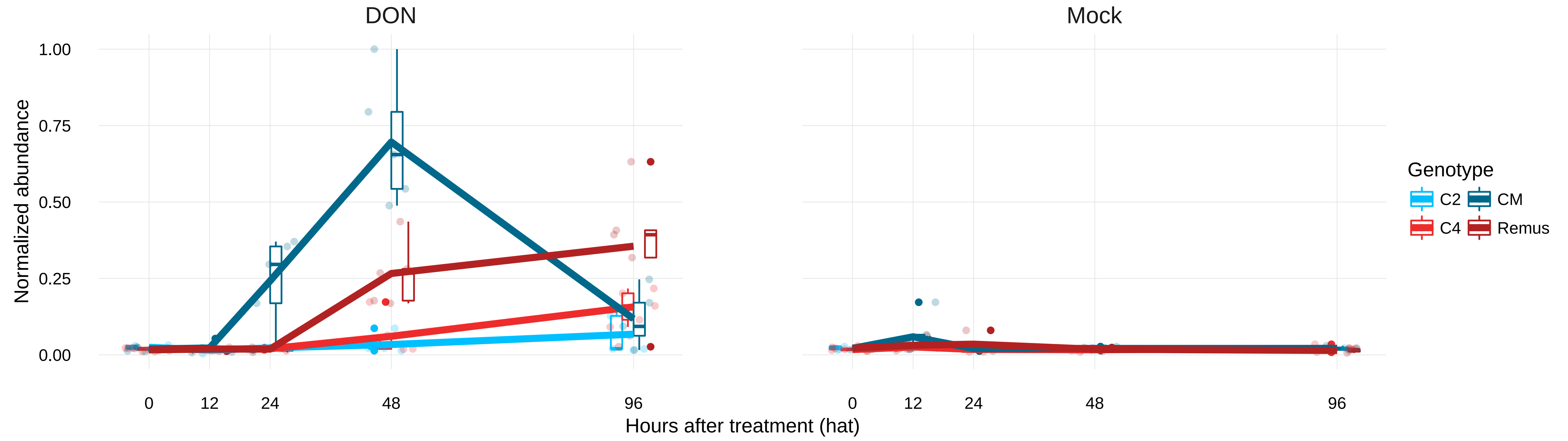

A.49

Annotated as HCA derivative
(1 database hit)

MZ	339.1076
RT	11.79 min
Normalization	Indirectly via surrogate in the KPX samples
Cluster	Cluster 3
Cn total / Phe	16 /

C2, C4; different treatments

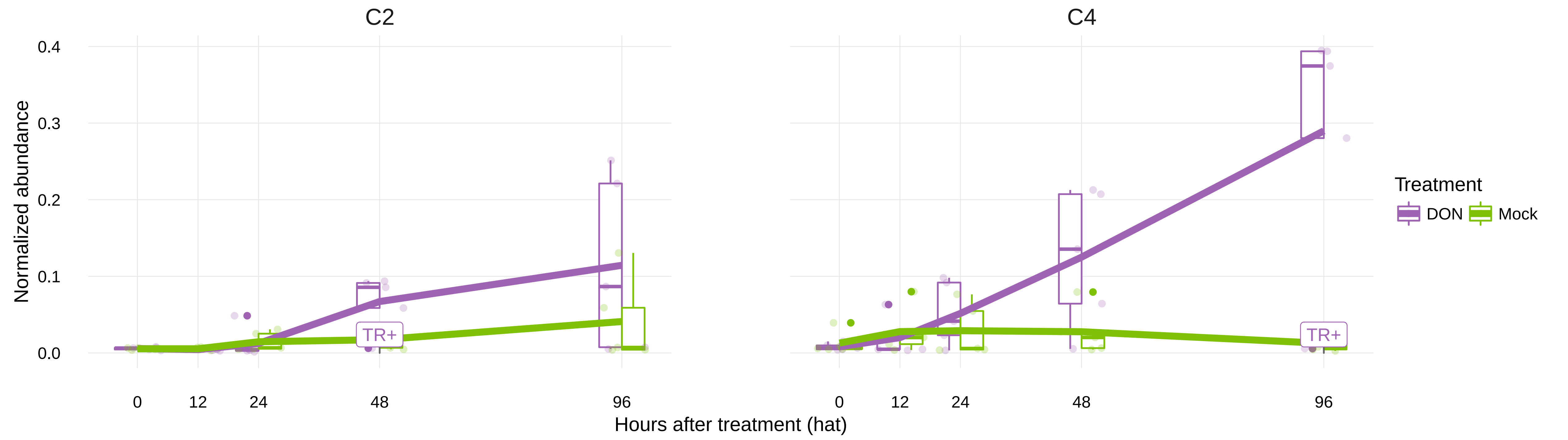

DON, Mock; different genotypes

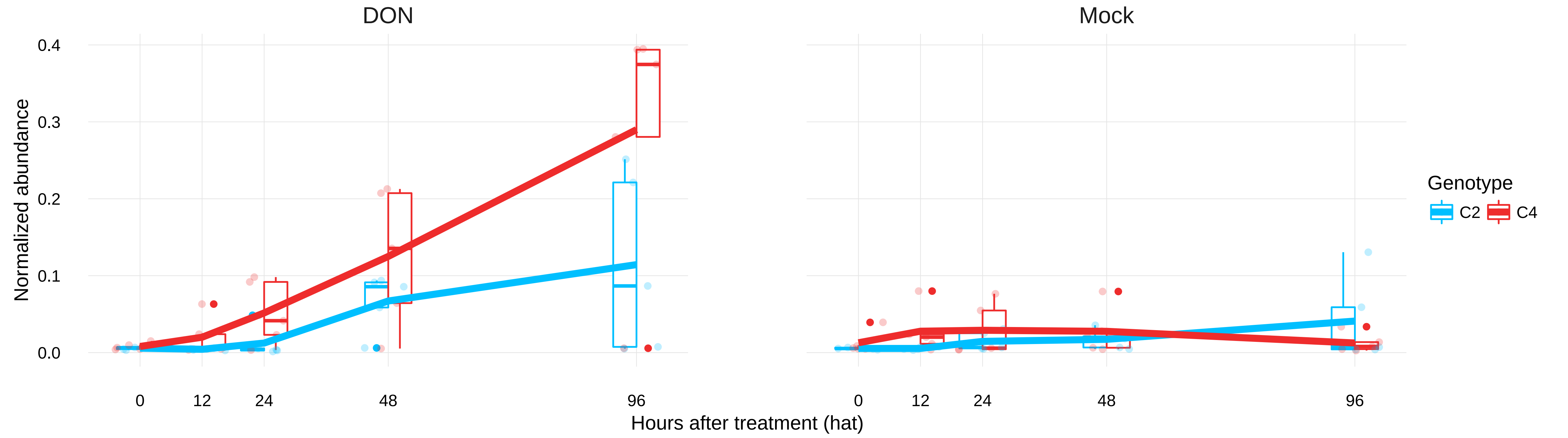

CM, Remus; different treatments

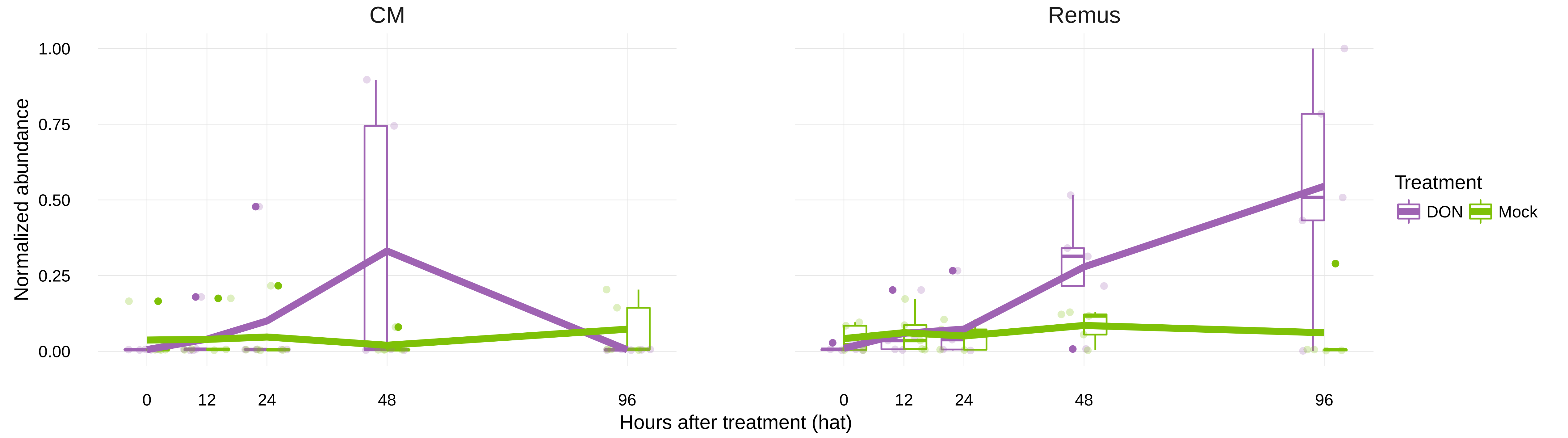

DON, Mock; all four genotypes

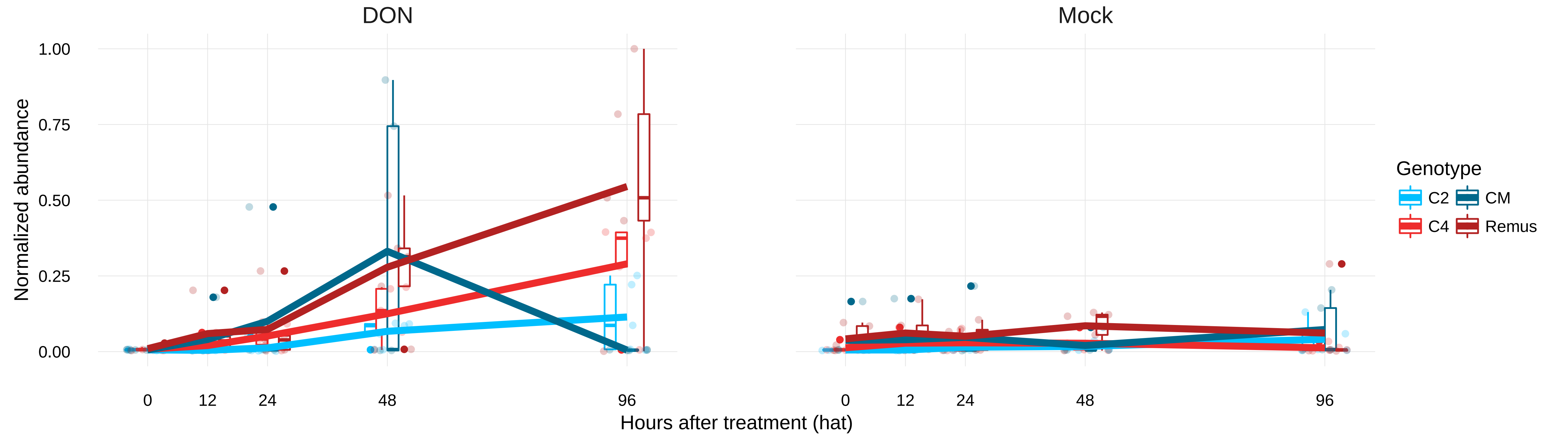

U.7

Unknown Phe−derived wheat−metabolite

MZ	193.086
RT	12.96 min
Normalization	Directly via KPX samples
Cluster	Cluster 3
Cn total / Phe	11 / 8

C2, C4; different treatments

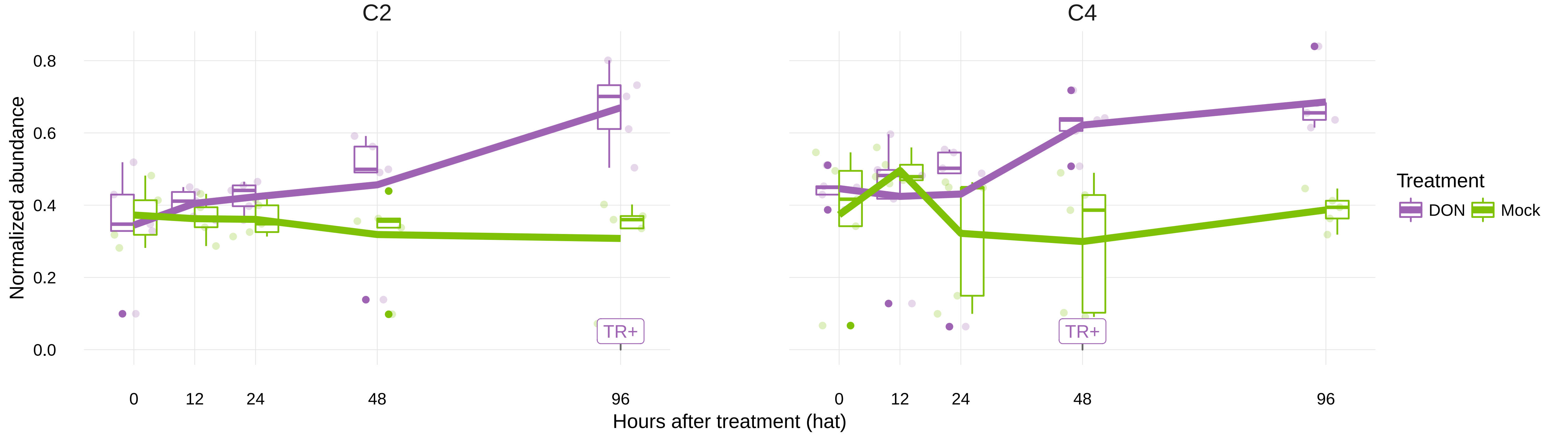

DON, Mock; different genotypes

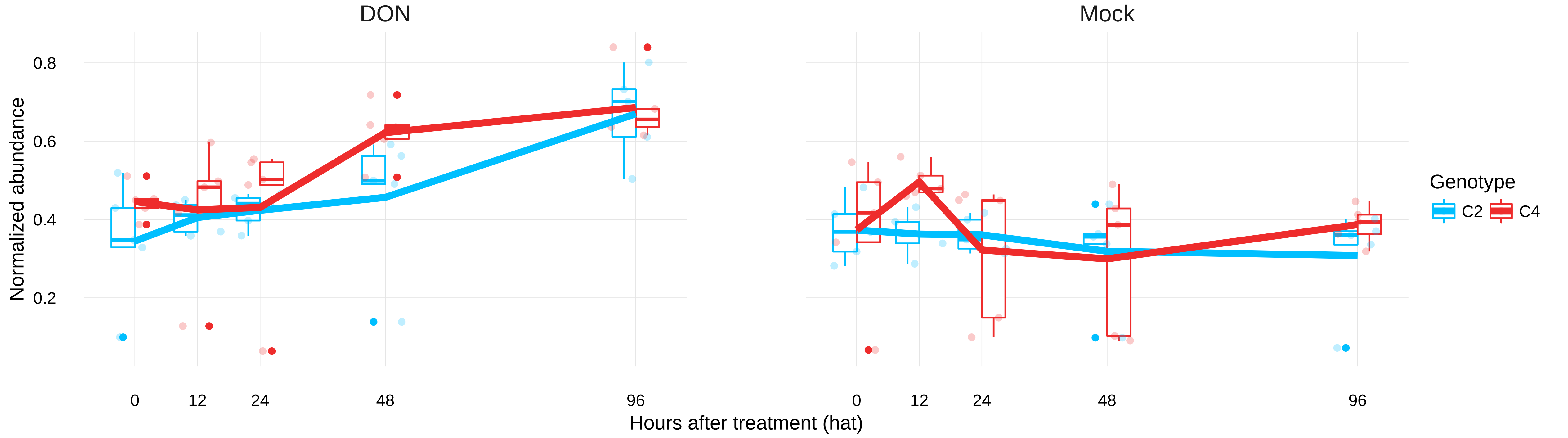

CM, Remus; different treatments

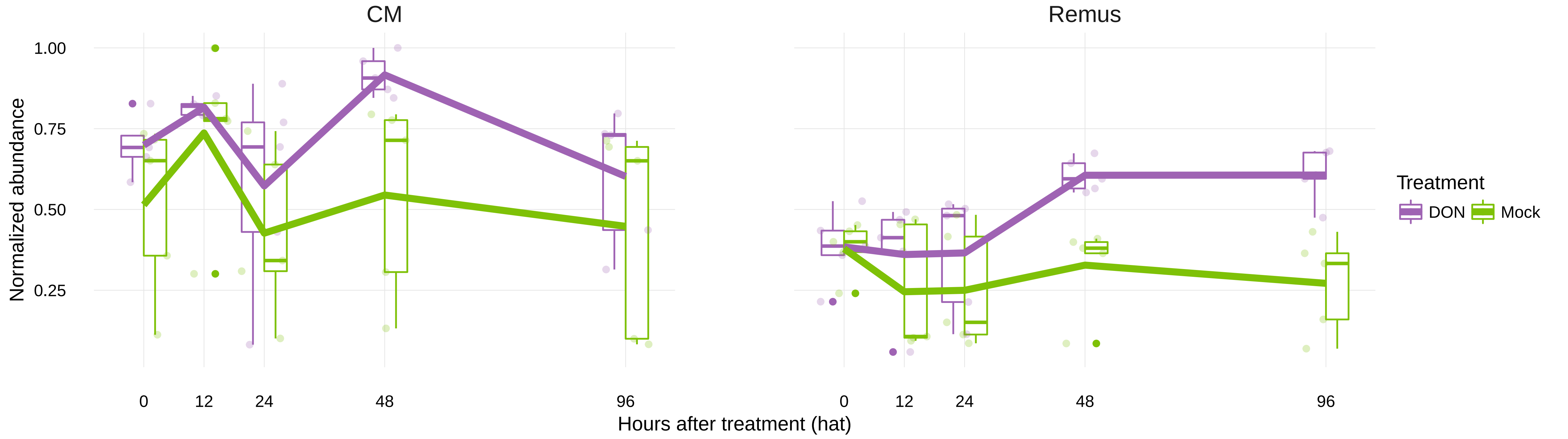

DON, Mock; all four genotypes

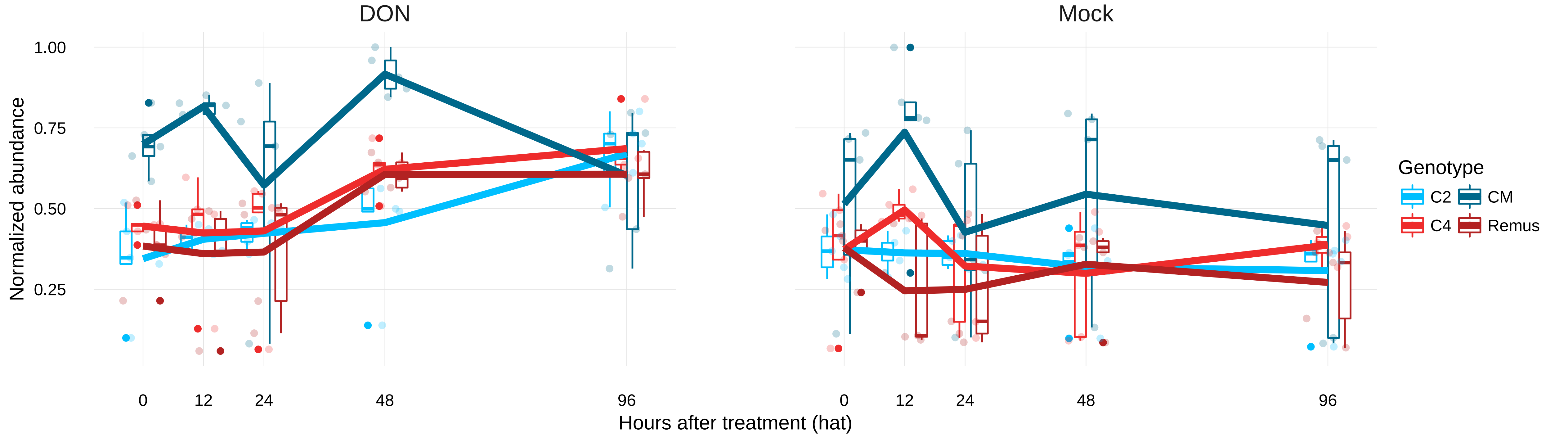

A.46

Annotated as Putative phenylacetic acid derivative
(1 database hit)

MZ	409.1107
RT	11.61 min
Normalization	Directly via KPX samples
Cluster	Cluster 3
Cn total / Phe	17 /

C2, C4; different treatments

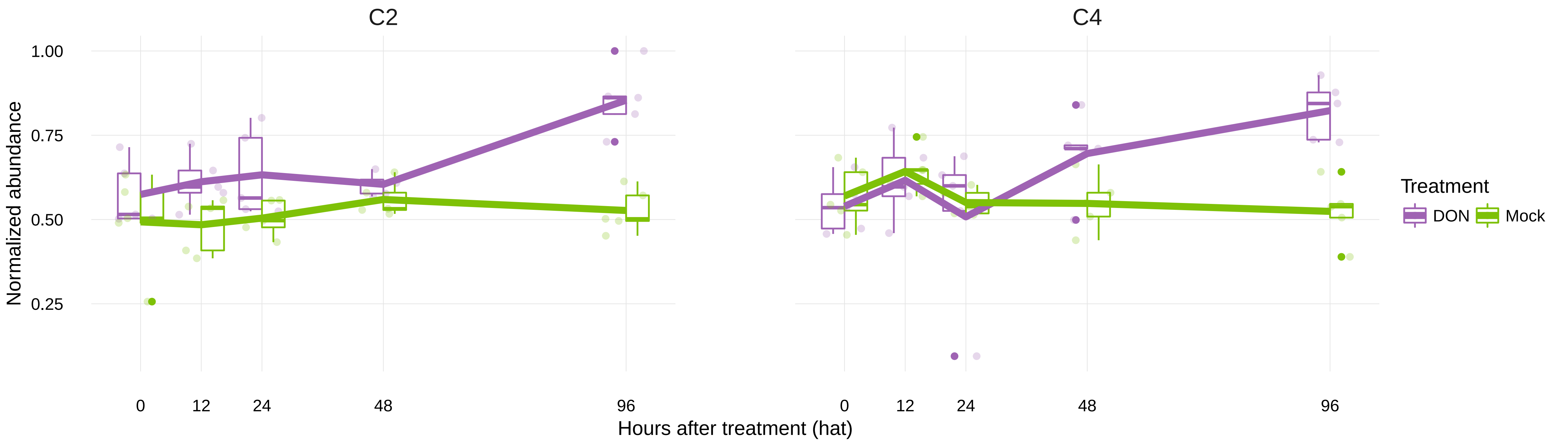

DON, Mock; different genotypes

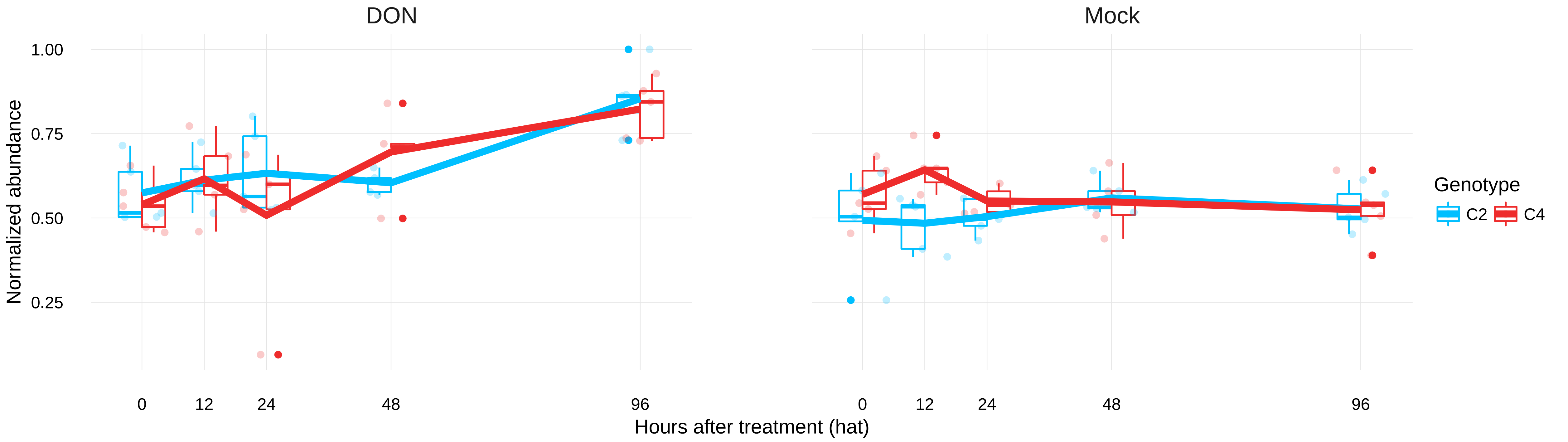

CM, Remus; different treatments

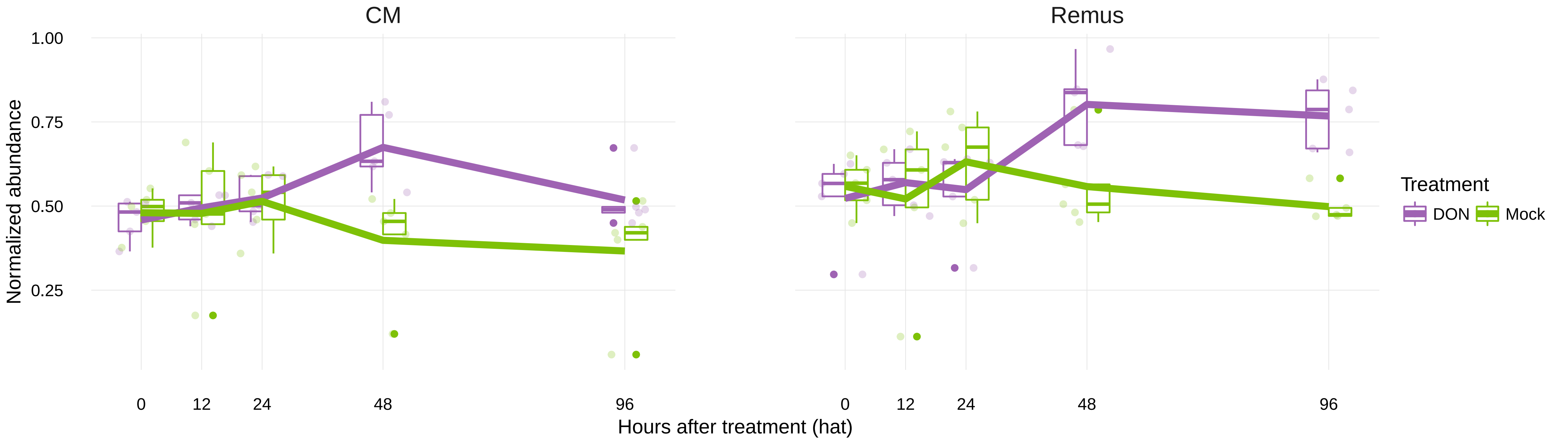

DON, Mock; all four genotypes

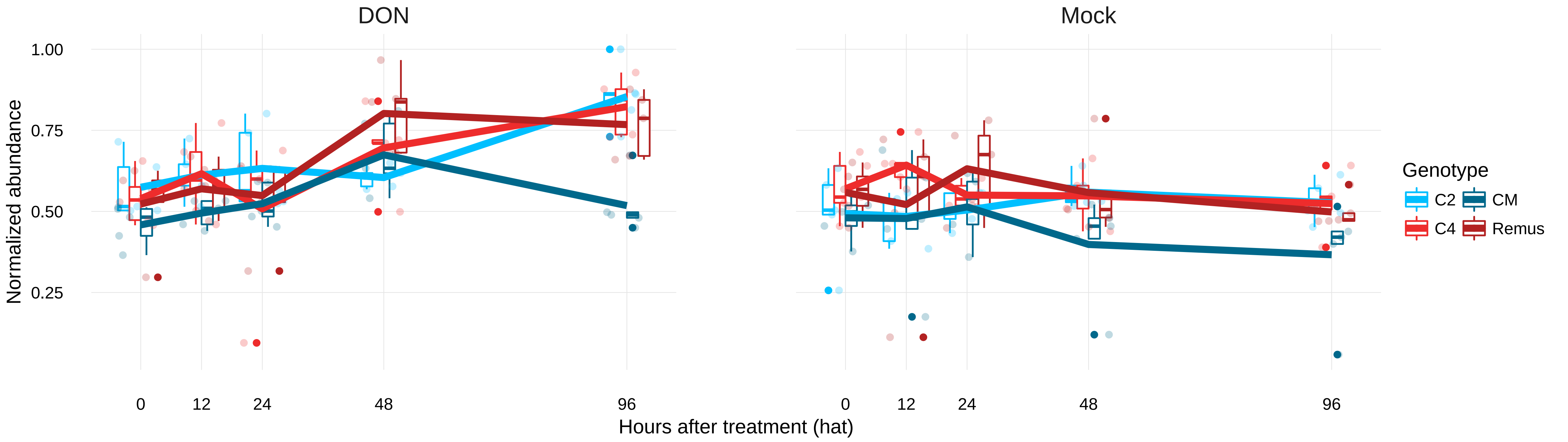

U.8

Unknown Phe−derived wheat−metabolite

MZ	451.1216
RT	13.19 min
Normalization	Directly via KPX samples
Cluster	Cluster 3
Cn total / Phe	9 / 9

C2, C4; different treatments

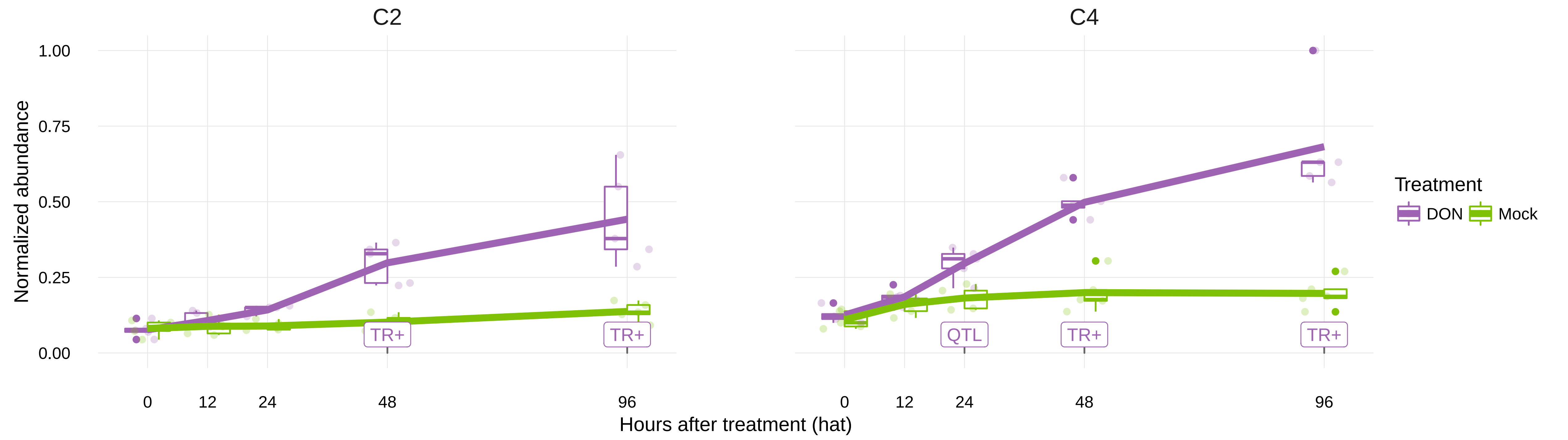

DON, Mock; different genotypes

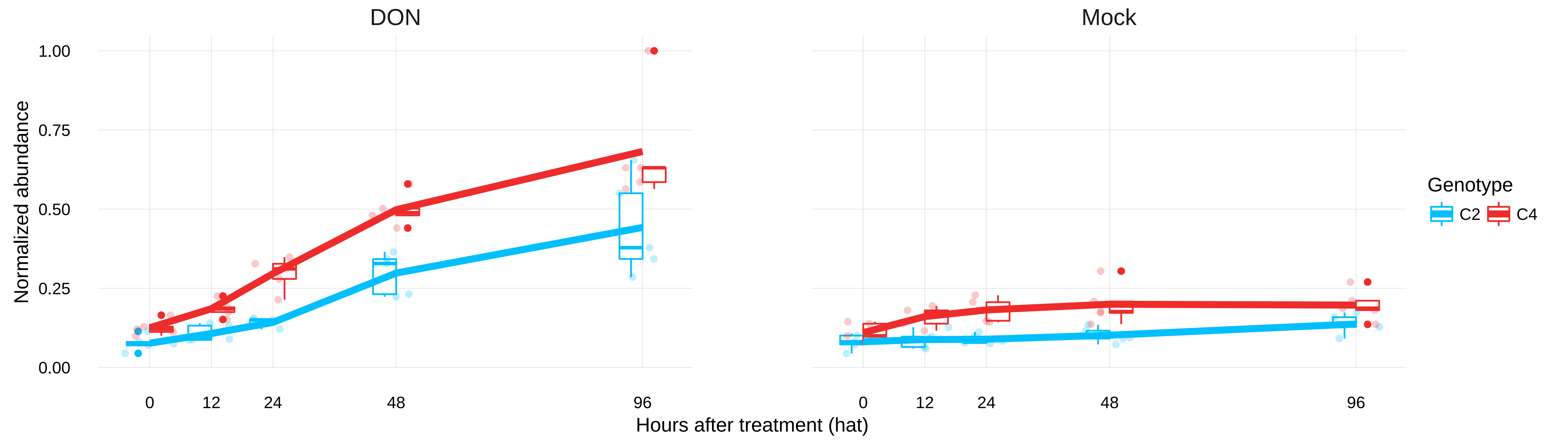

CM, Remus; different treatments

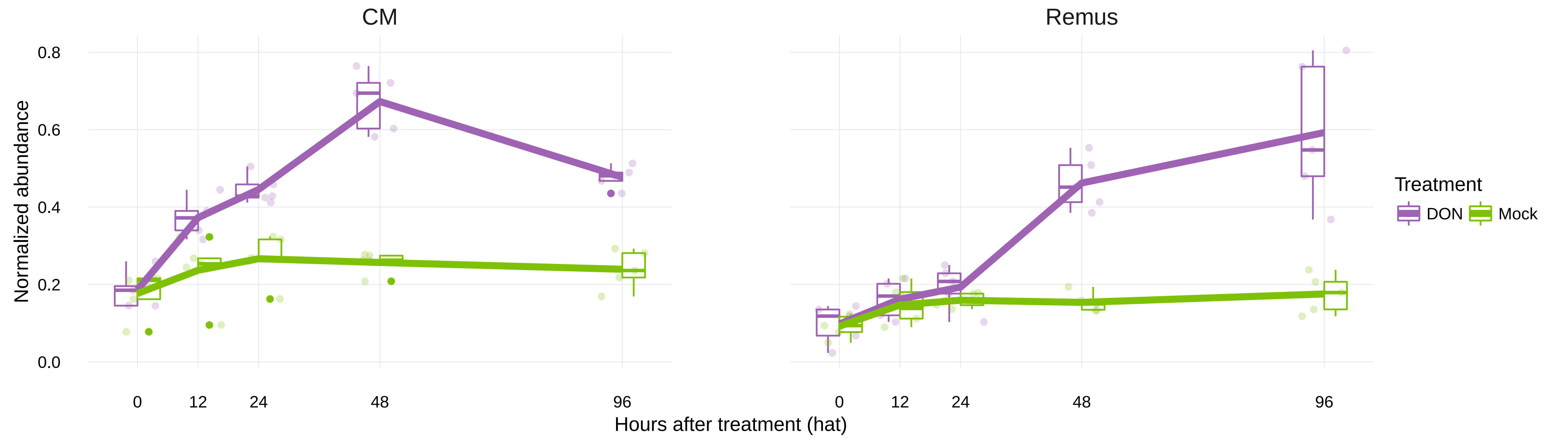

DON, Mock; all four genotypes

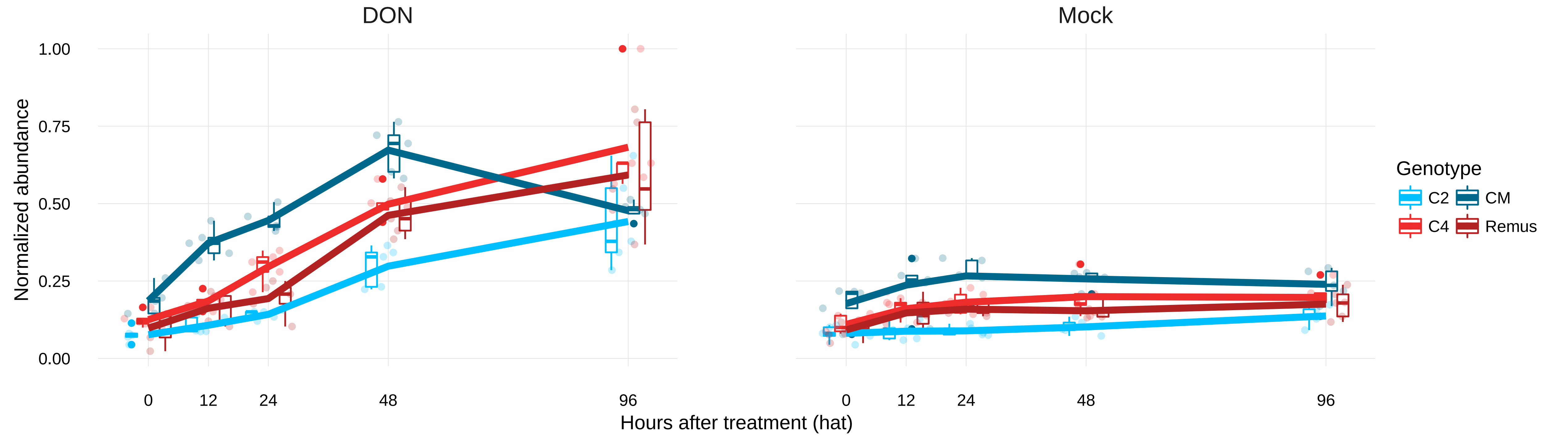

A.66

Annotated as HCA derivative

(2 database hits)

MZ	239.0915
RT	13.16 min
Normalization	Directly via KPX samples
Cluster	Cluster 3
Cn total / Phe	12 / 9

C2, C4; different treatments

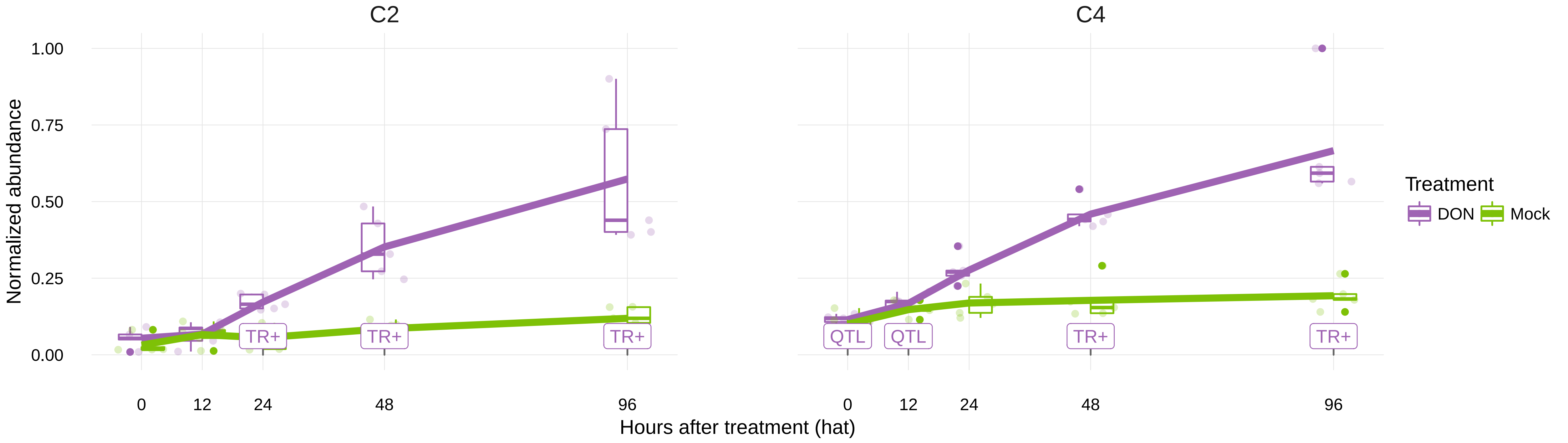

DON, Mock; different genotypes

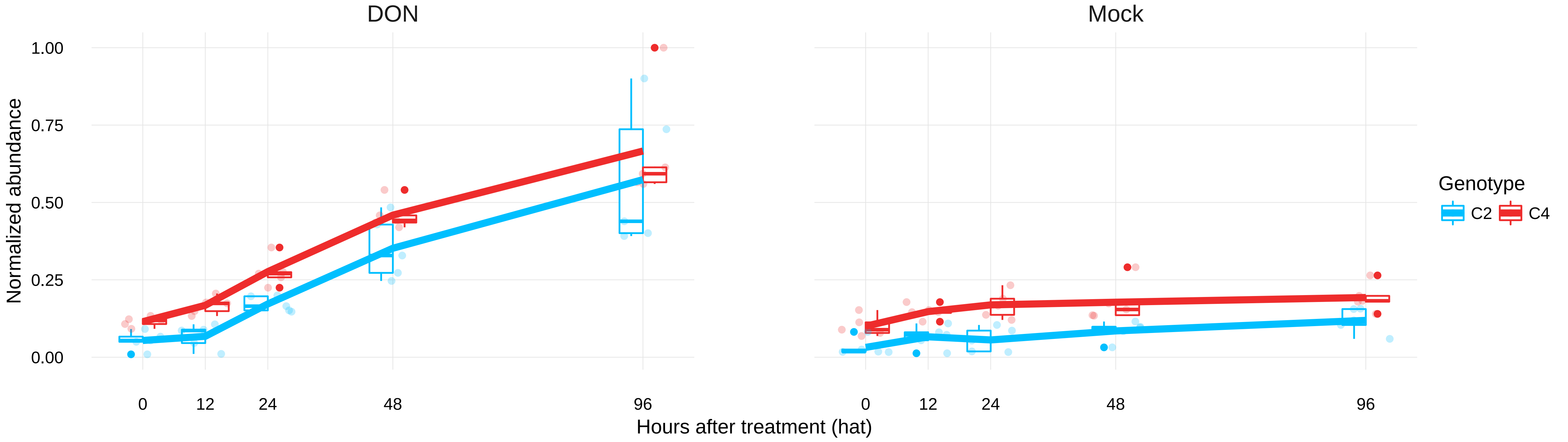

CM, Remus; different treatments

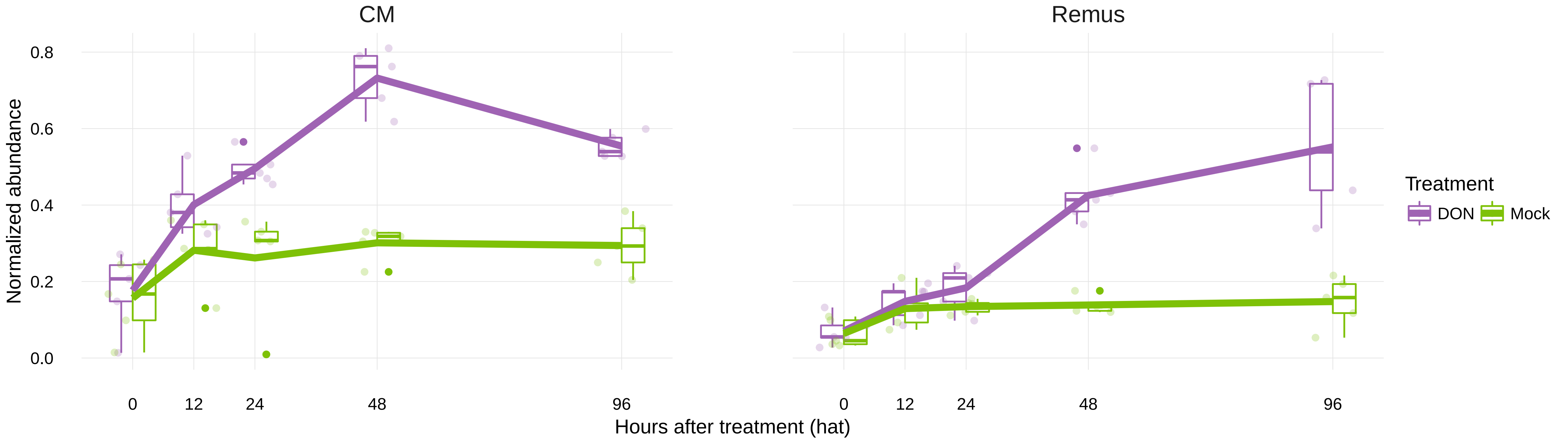

DON, Mock; all four genotypes

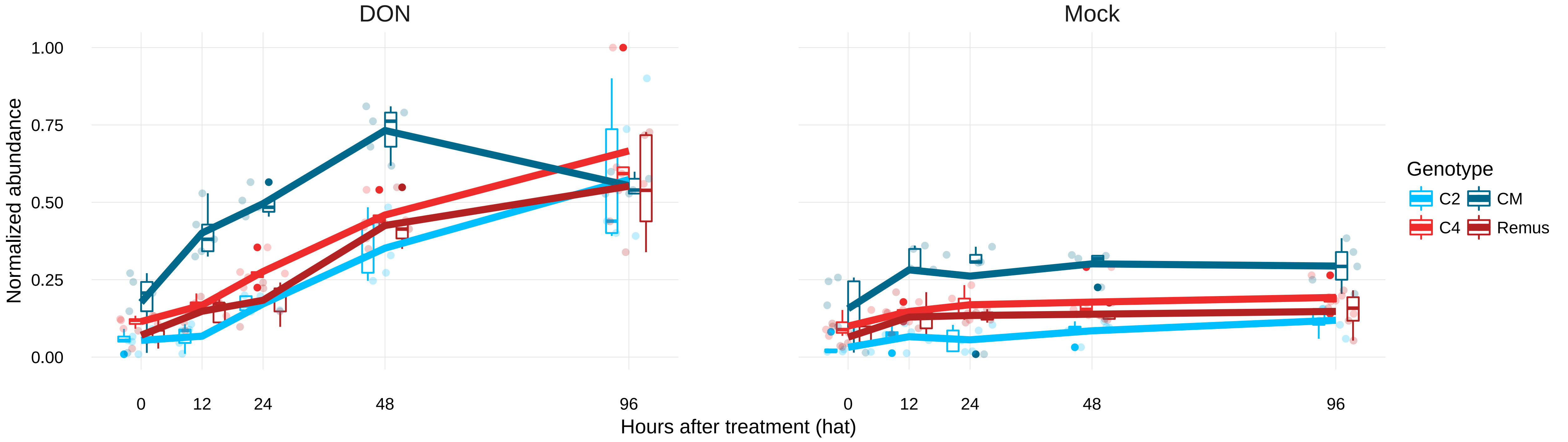

A.57

Annotated as others (Phenol)

(1 database hit)

MZ	185.0808
RT	12.76 min
Normalization	Directly via KPX samples
Cluster	Cluster 3
Cn total / Phe	9 / 6

C2, C4; different treatments

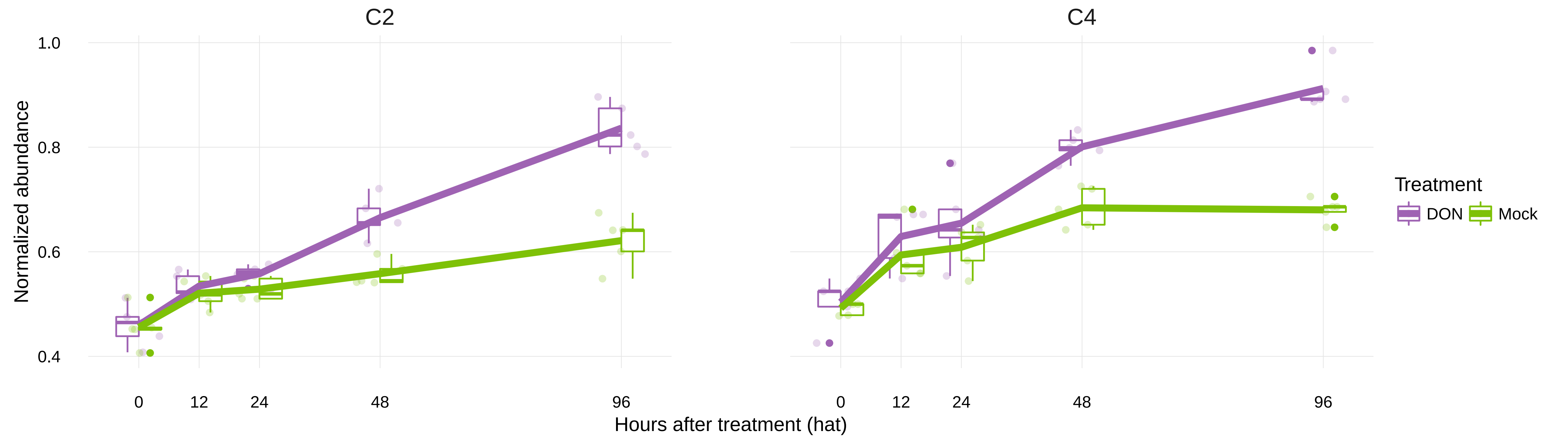

DON, Mock; different genotypes

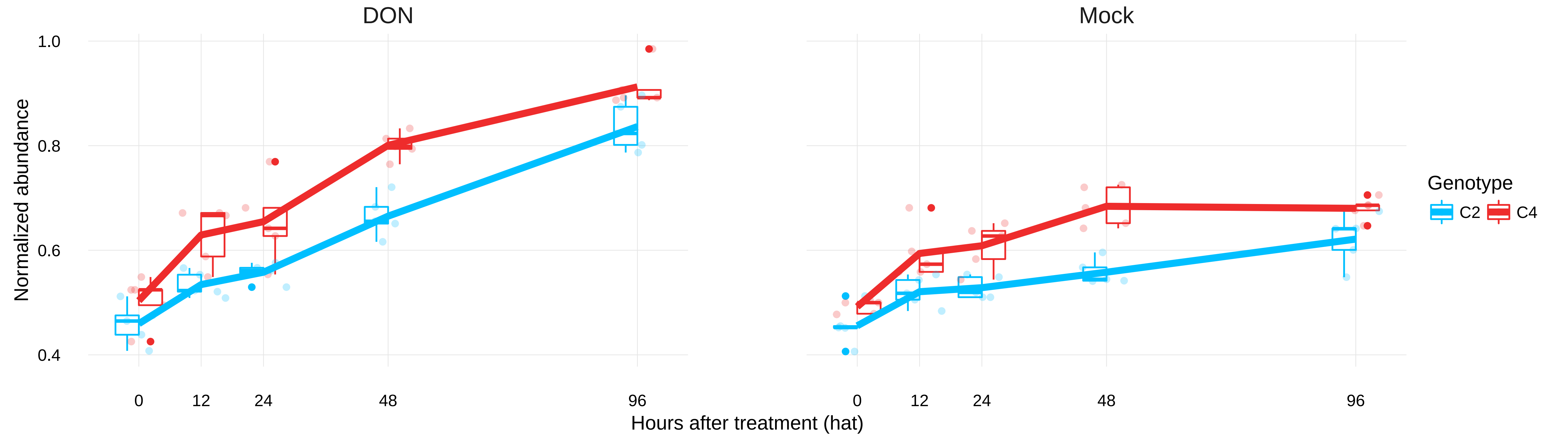

CM, Remus; different treatments

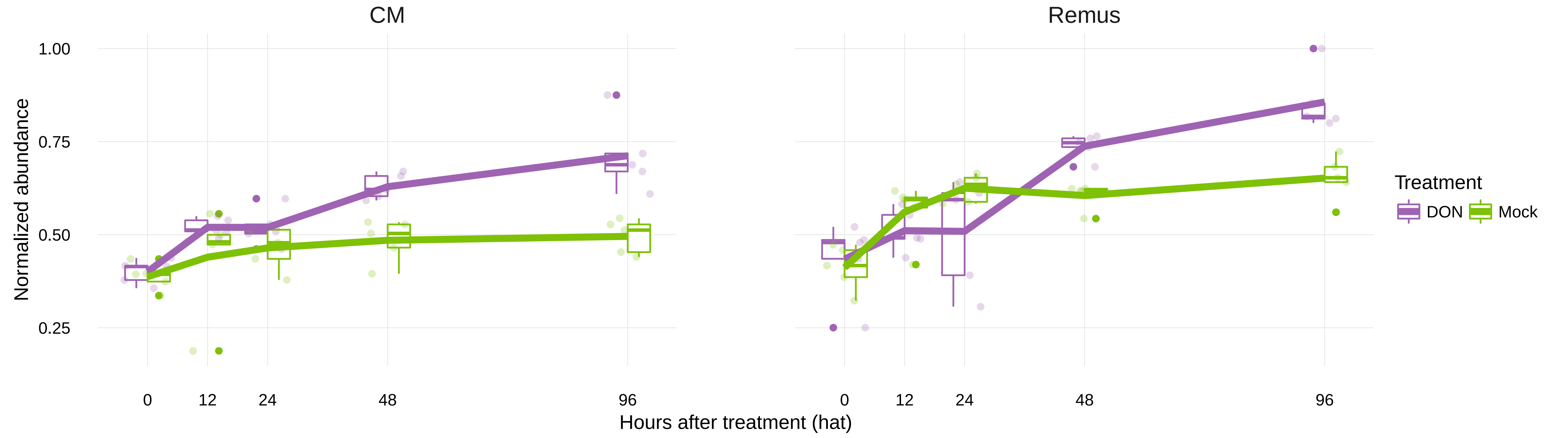

DON, Mock; all four genotypes

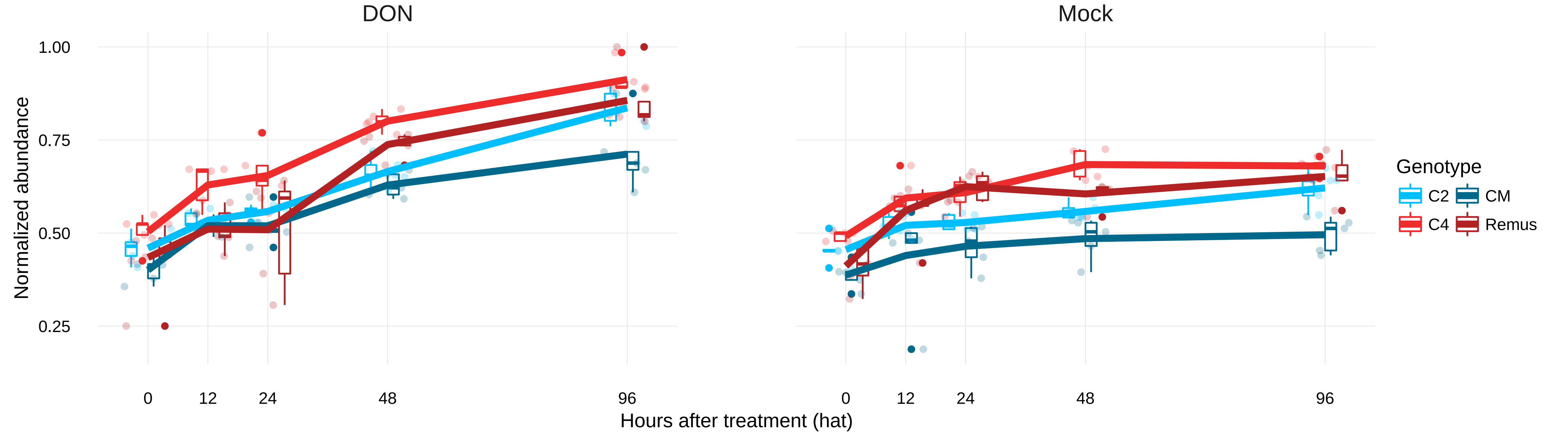

A.87

Annotated as Lignan

(1 database hit)

MZ	603.205
RT	15.28 min
Normalization	Directly via KPX samples
Cluster	Cluster 3
Cn total / Phe	28 /

C2, C4; different treatments

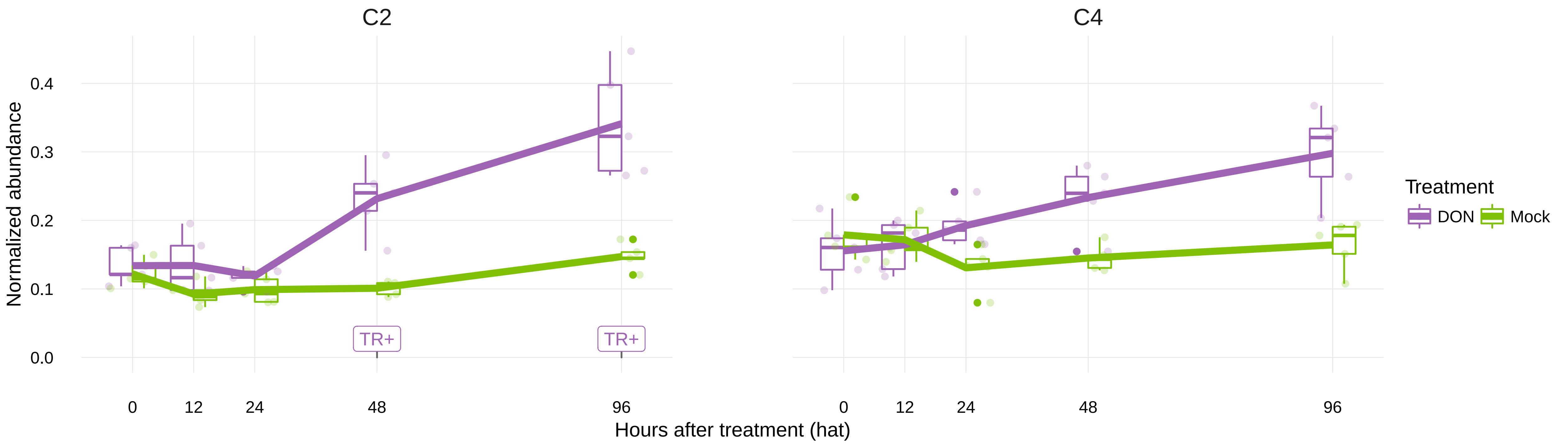

DON, Mock; different genotypes

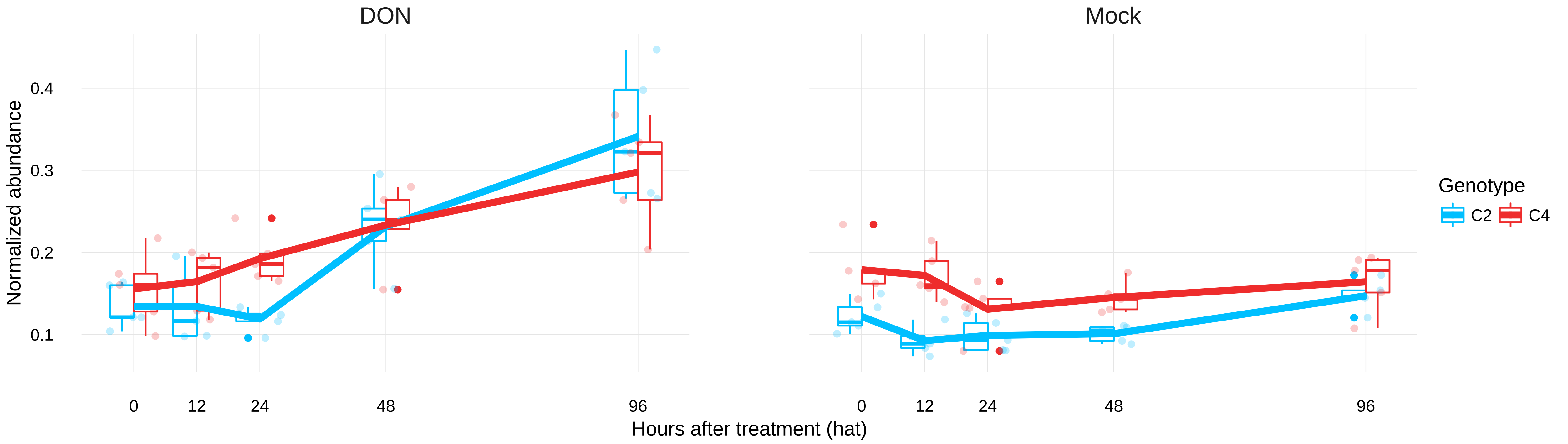

CM, Remus; different treatments

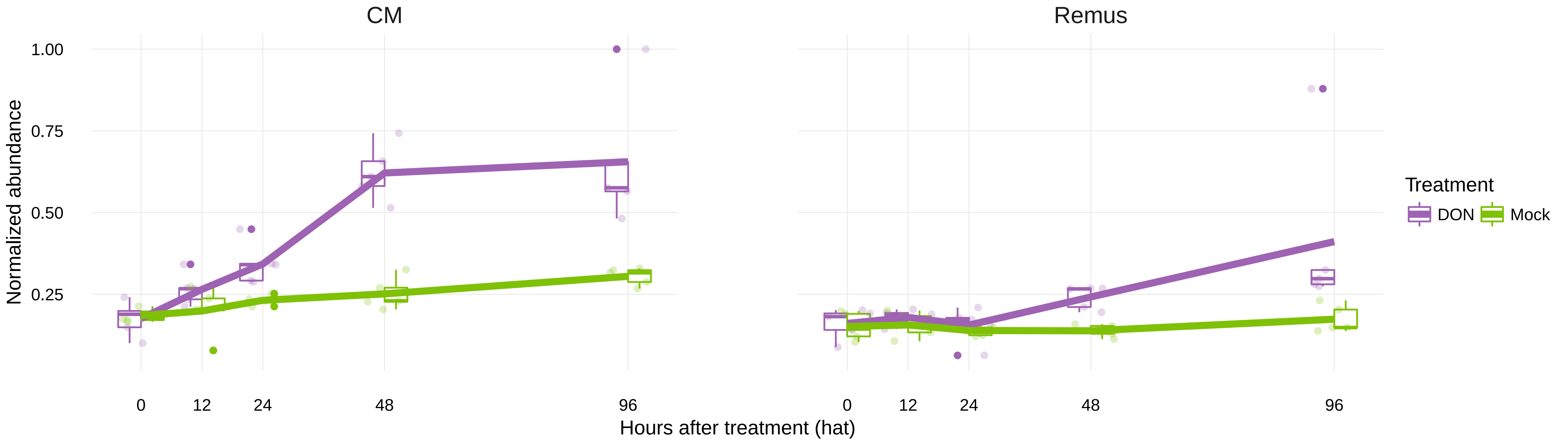

DON, Mock; all four genotypes

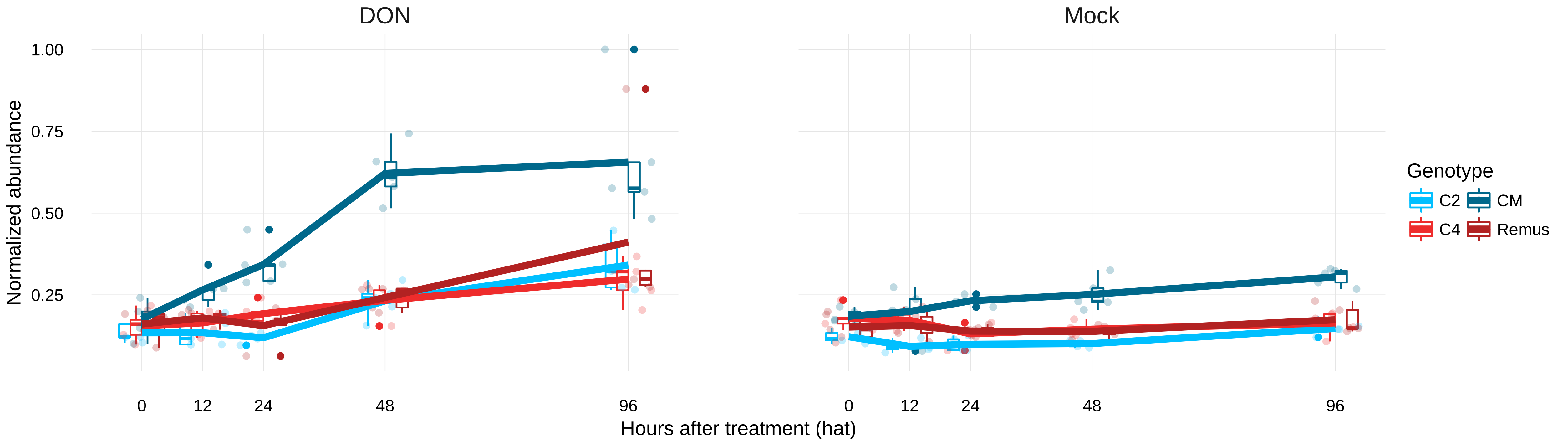

A.111

Annotated as Flavonoid
(21 database hits)

MZ	371.149
RT	17.93 min
Normalization	Directly via KPX samples
Cluster	Cluster 3
Cn total / Phe	21 /

C2, C4; different treatments

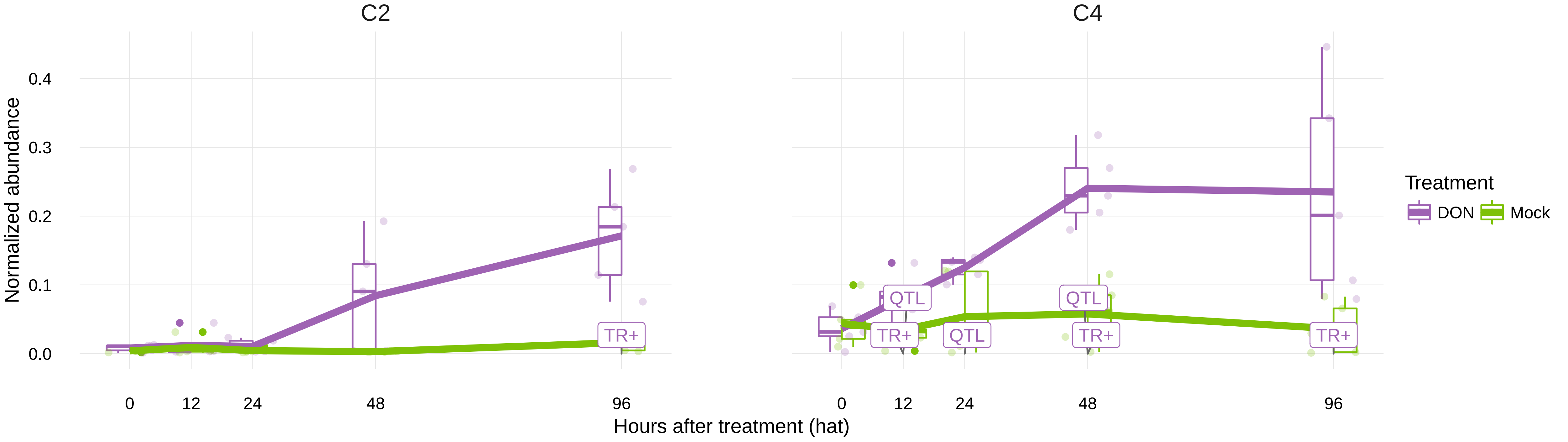

DON, Mock; different genotypes

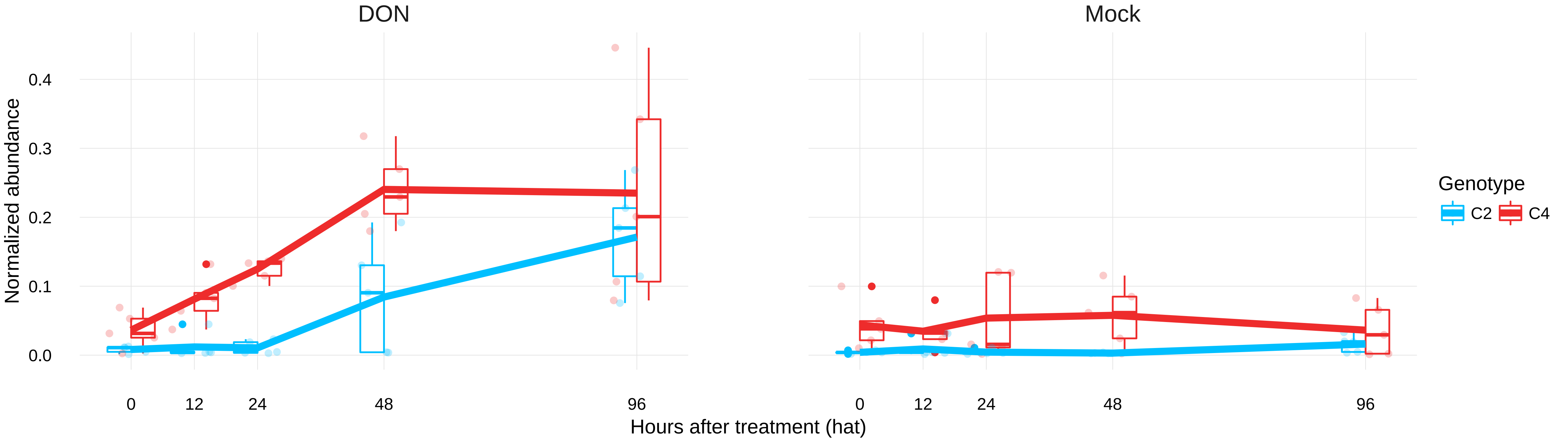

CM, Remus; different treatments

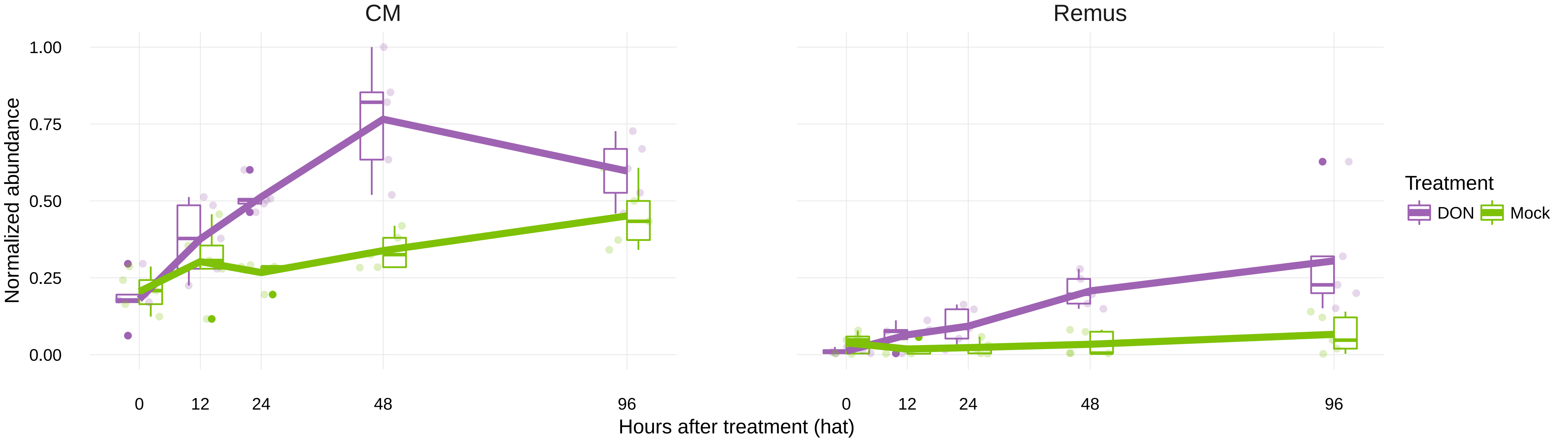

DON, Mock; all four genotypes

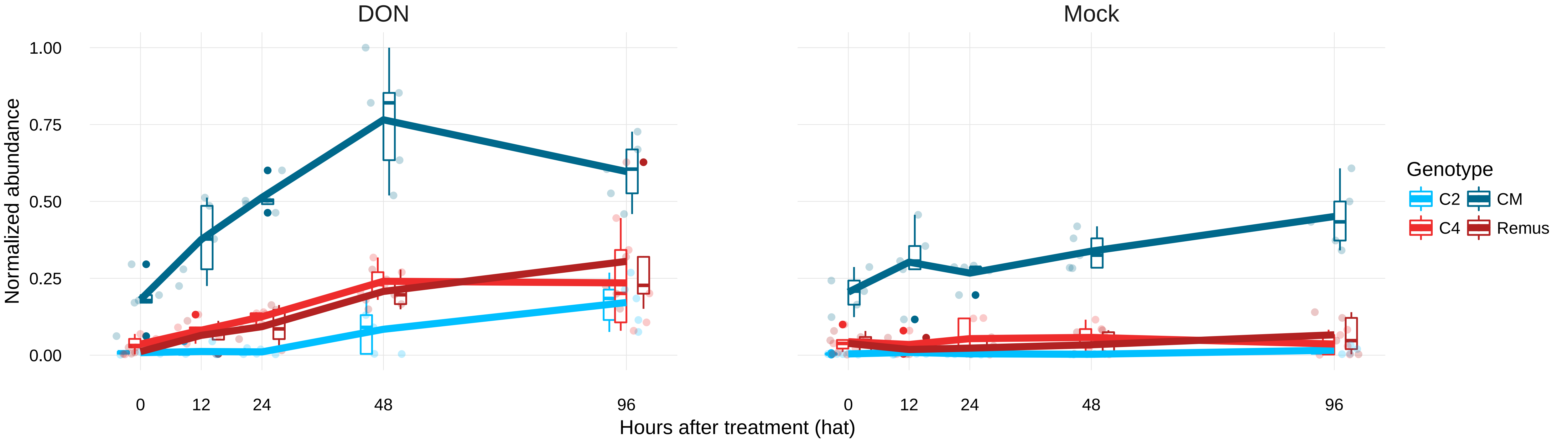

A.99

Annotated as others (Coumarins)
(1 database hit)

MZ	399.1413
RT	16.5 min
Normalization	Directly via KPX samples
Cluster	Cluster 3
Cn total / Phe	20 /

C2, C4; different treatments

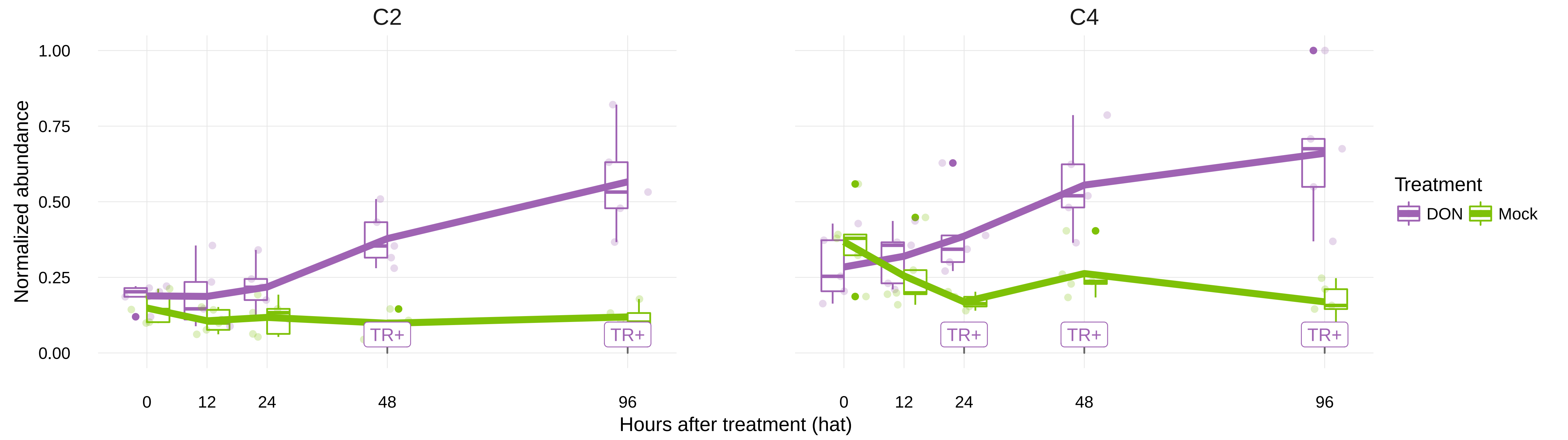

DON, Mock; different genotypes

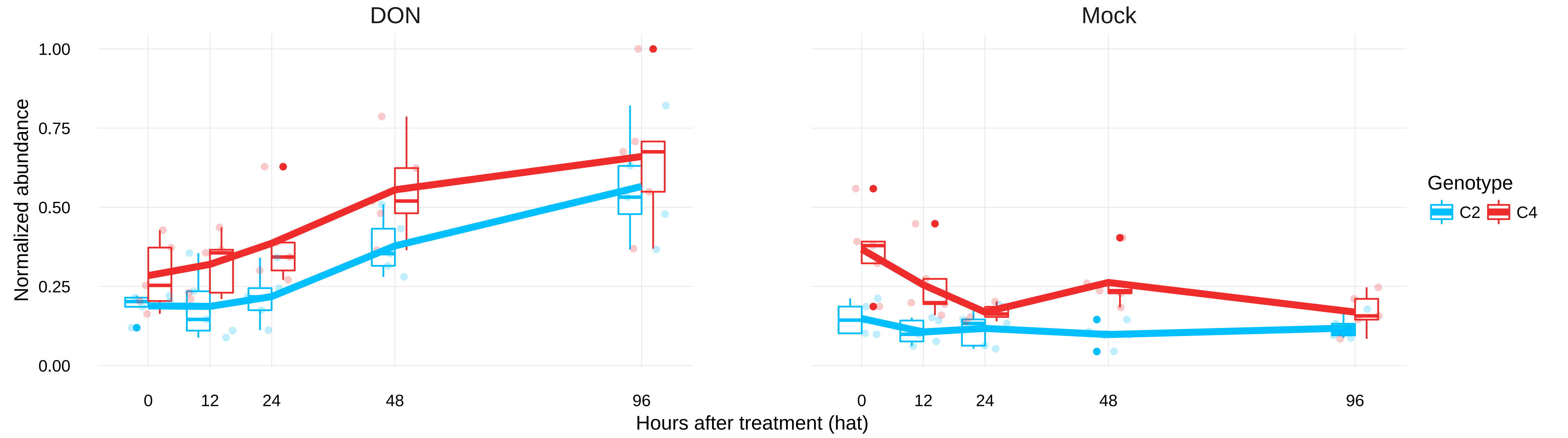

CM, Remus; different treatments

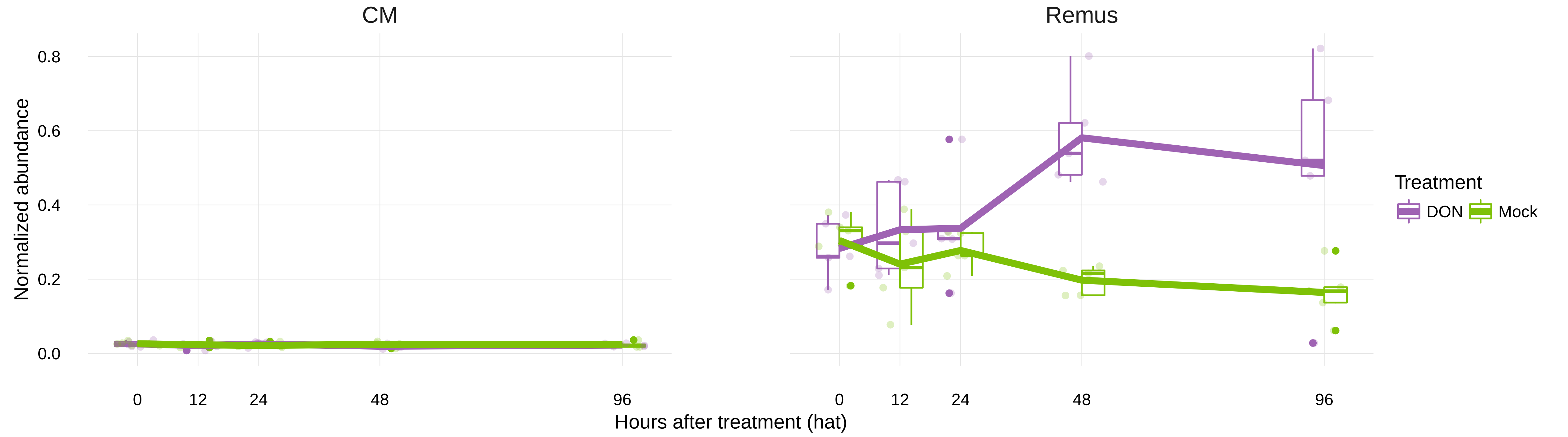

DON, Mock; all four genotypes

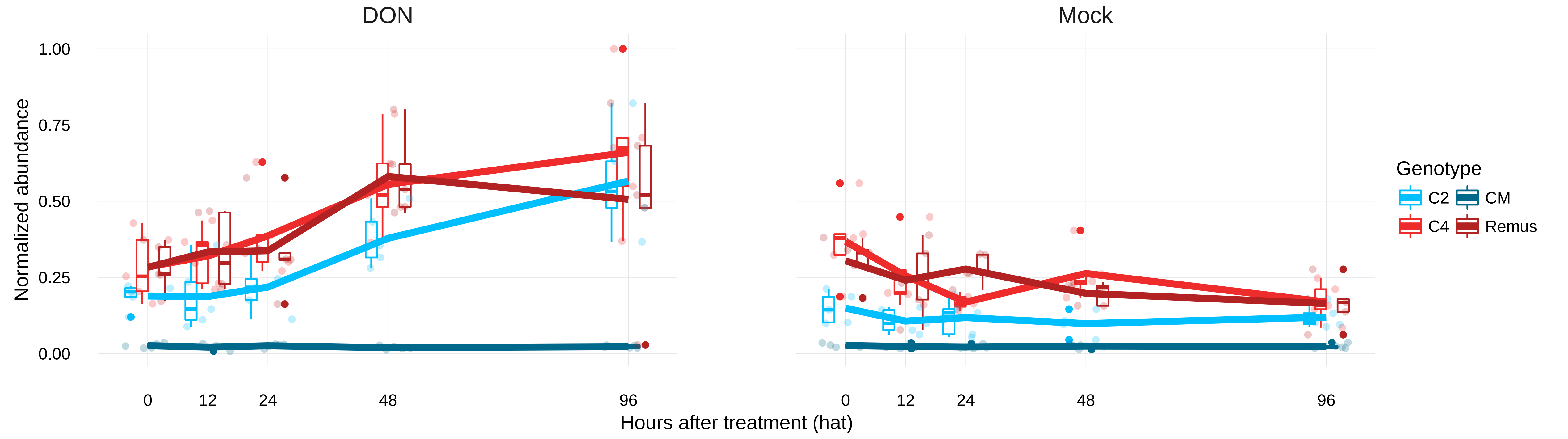

A.75

Annotated as Flavonoid

(30 database hits)

MZ	341.1386
RT	13.98 min
Normalization	Directly via KPX samples
Cluster	Cluster 3
Cn total / Phe	20 / 9

C2, C4; different treatments

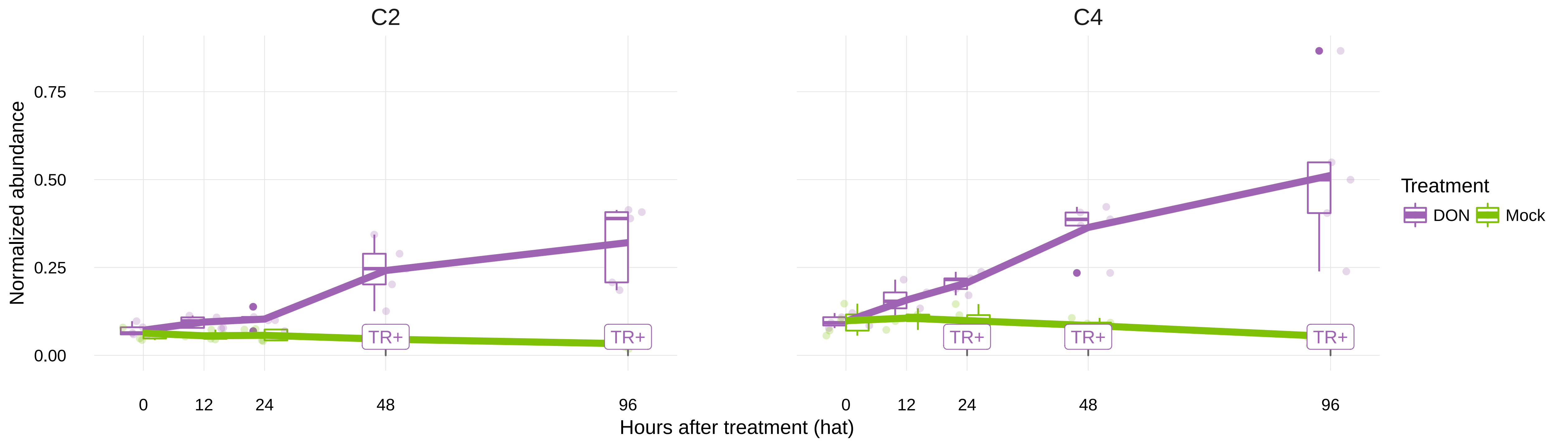

DON, Mock; different genotypes

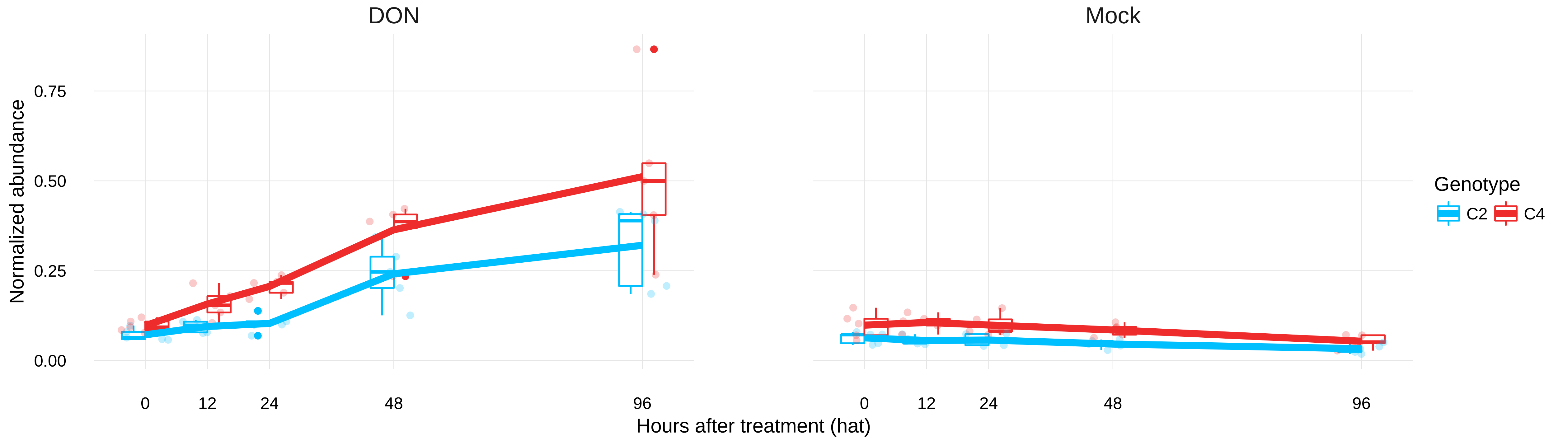

CM, Remus; different treatments

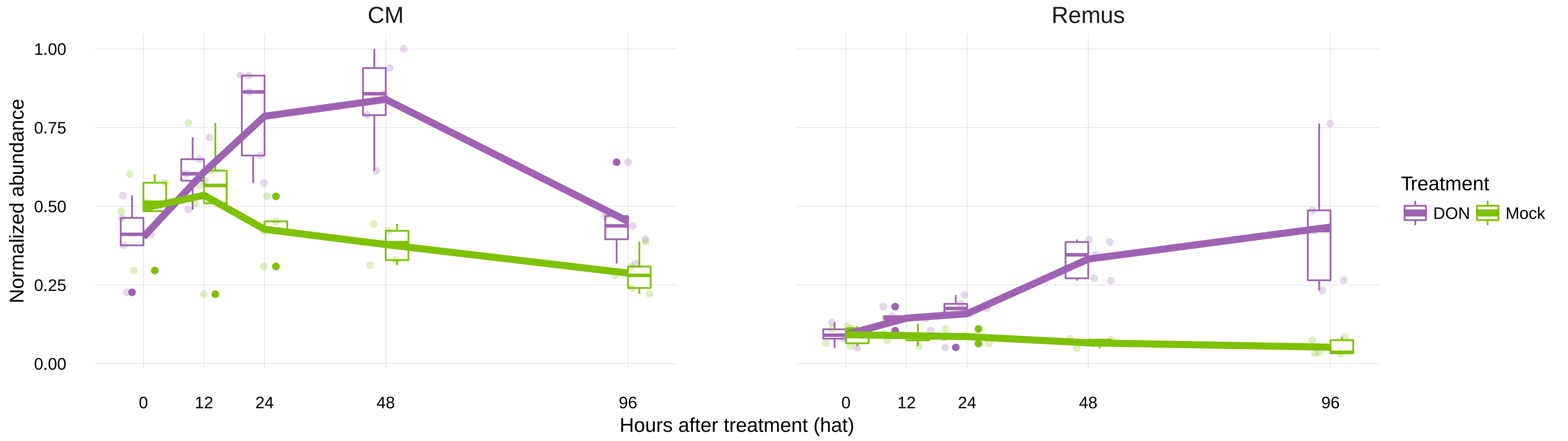

DON, Mock; all four genotypes

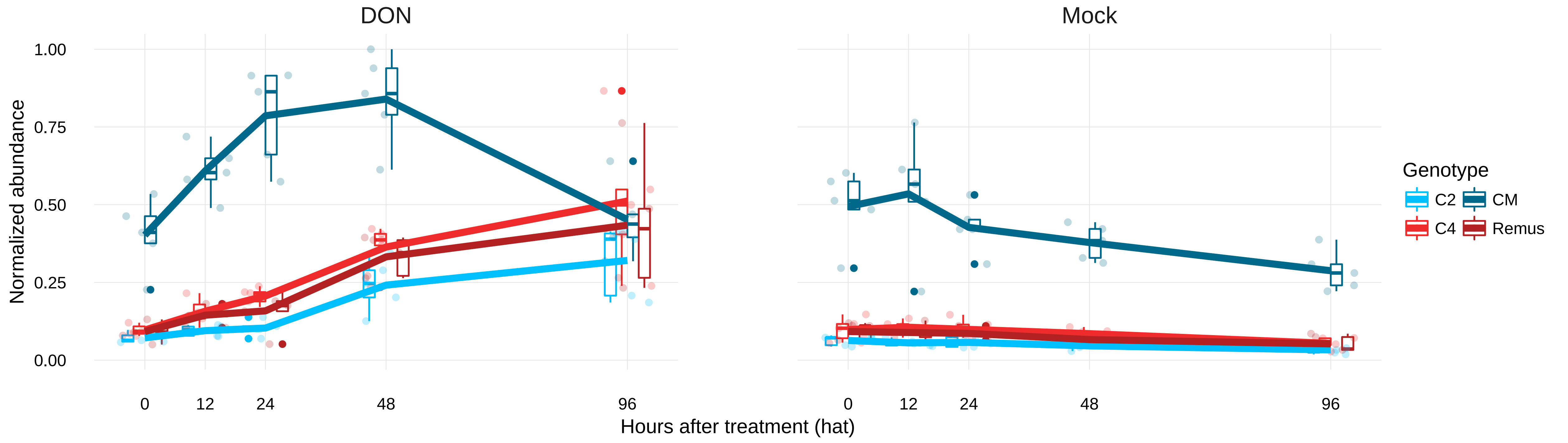

A.44

Annotated as Lignan
(2 database hits)

MZ	521.202
RT	11.2 min
Normalization	Directly via KPX samples
Cluster	Cluster 3
Cn total / Phe	26 / 9

C2, C4; different treatments

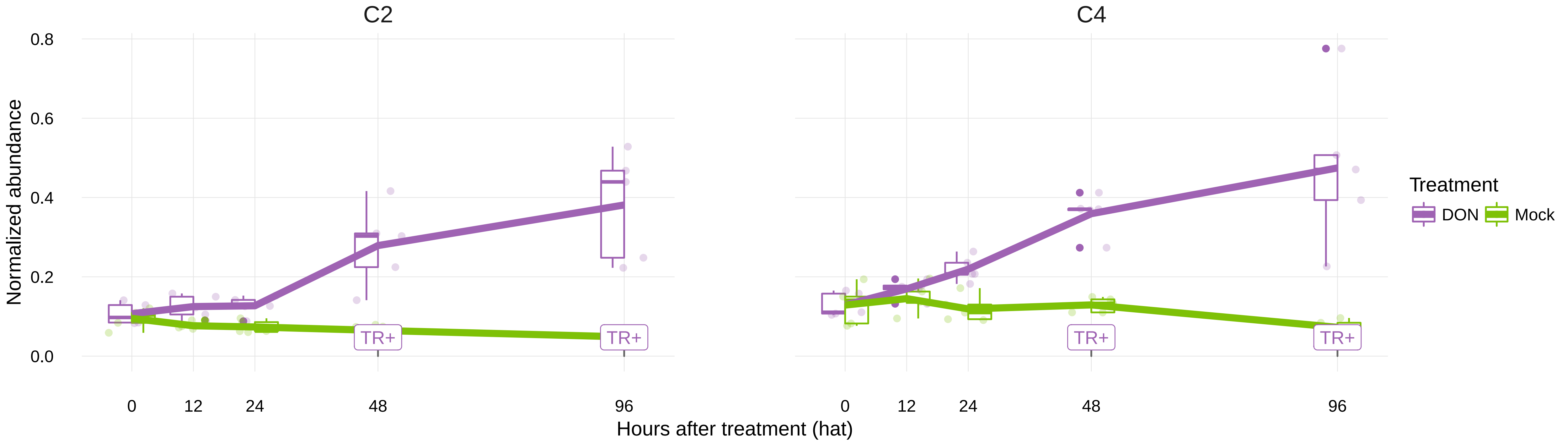

DON, Mock; different genotypes

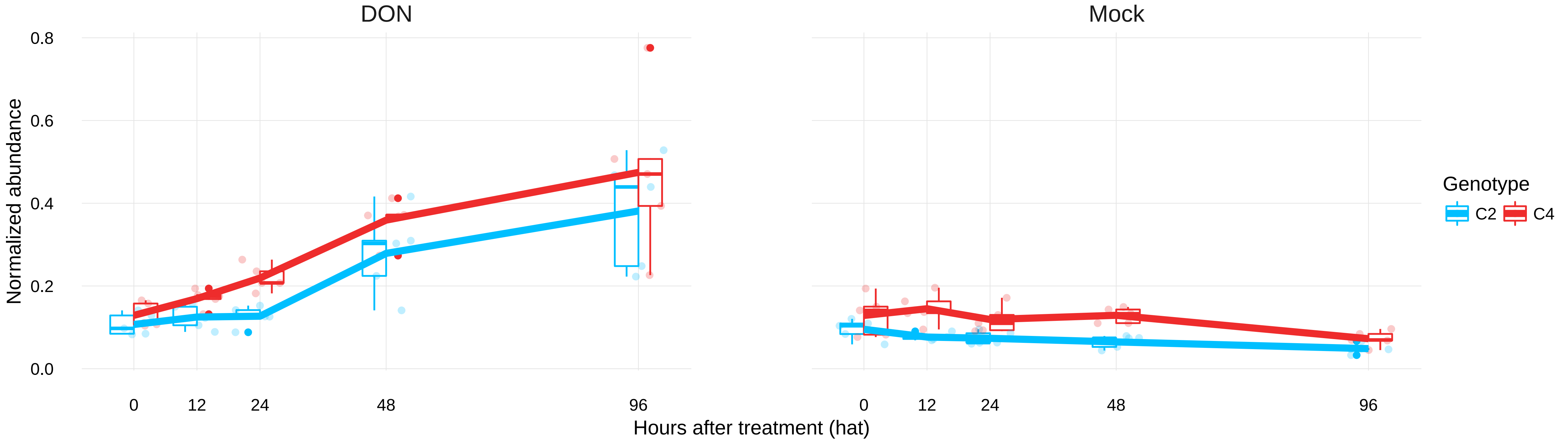

CM, Remus; different treatments

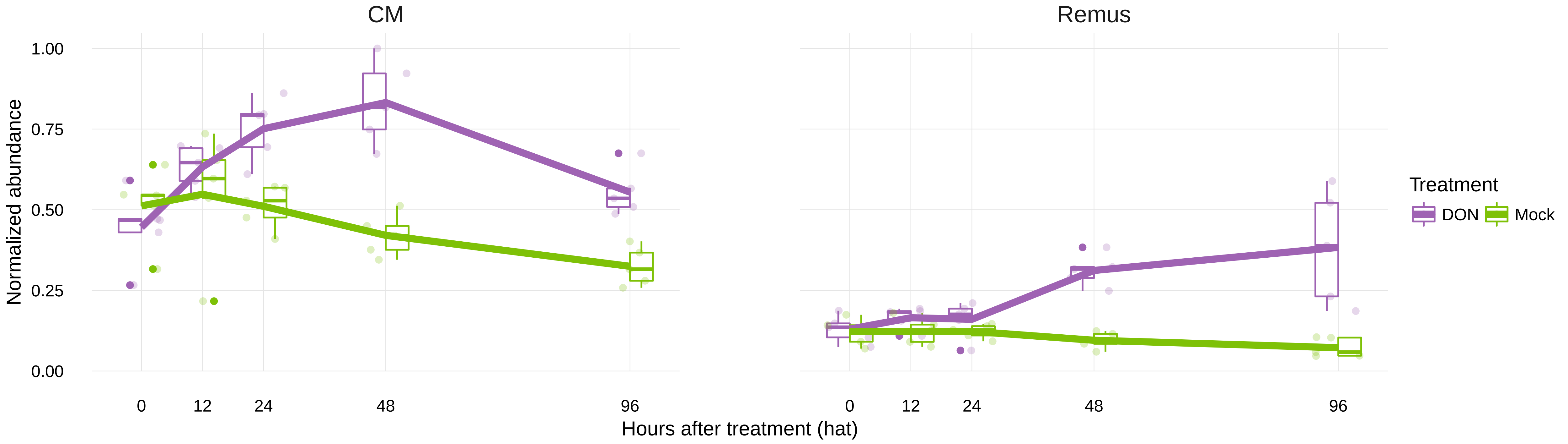

DON, Mock; all four genotypes

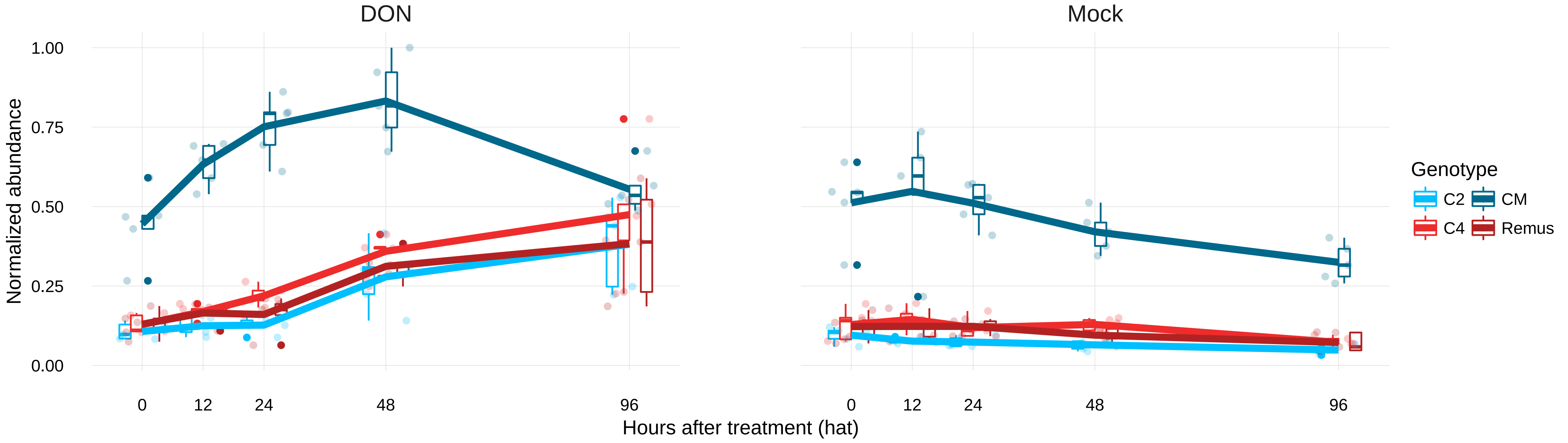

A.102

Annotated as Putative benzoic acid derivative
(1 database hit)

MZ	137.0597
RT	16.81 min
Normalization	Directly via KPX samples
Cluster	Cluster 3
Cn total / Phe	8 / 7

C2, C4; different treatments

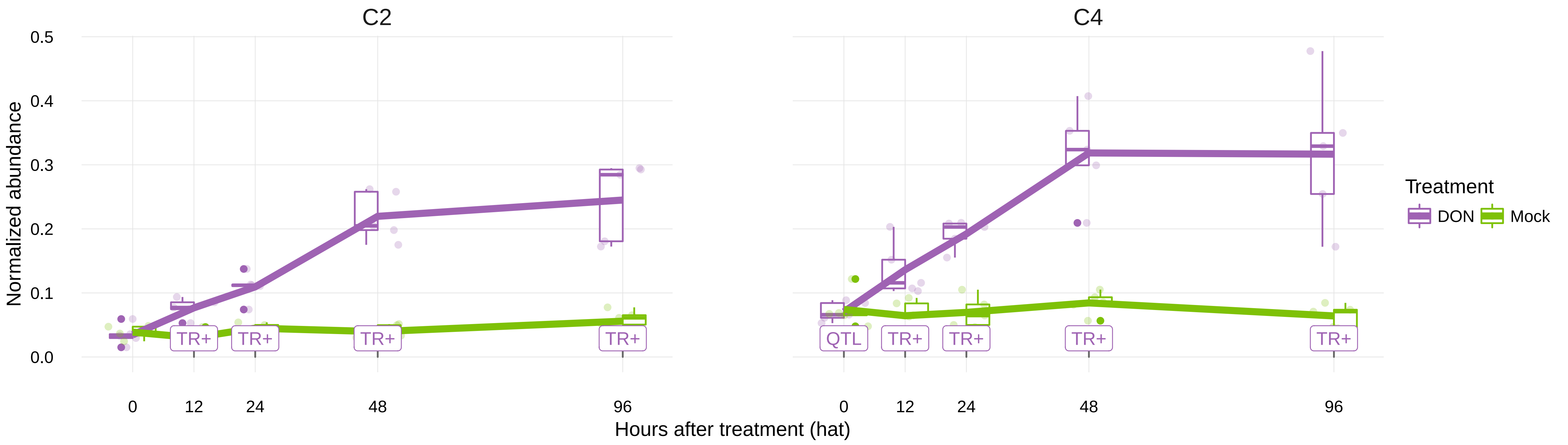

DON, Mock; different genotypes

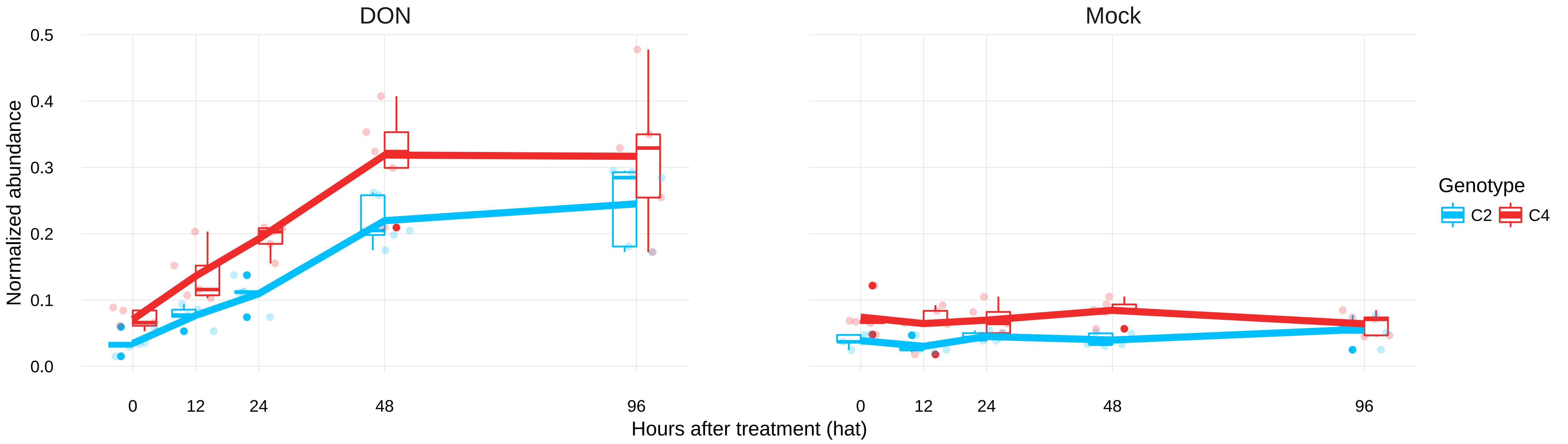

CM, Remus; different treatments

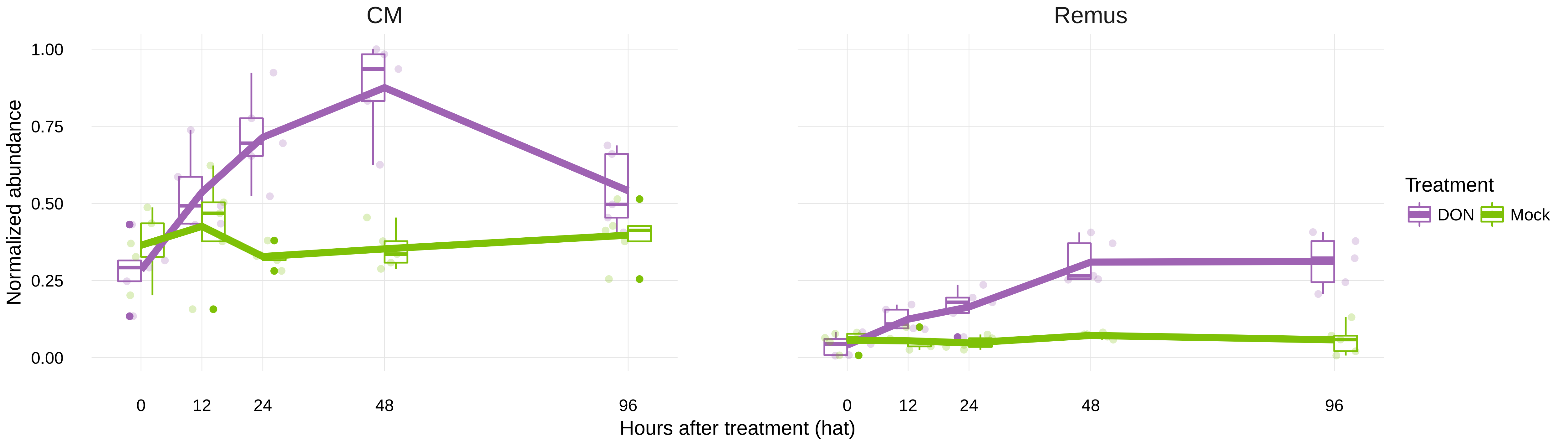

DON, Mock; all four genotypes

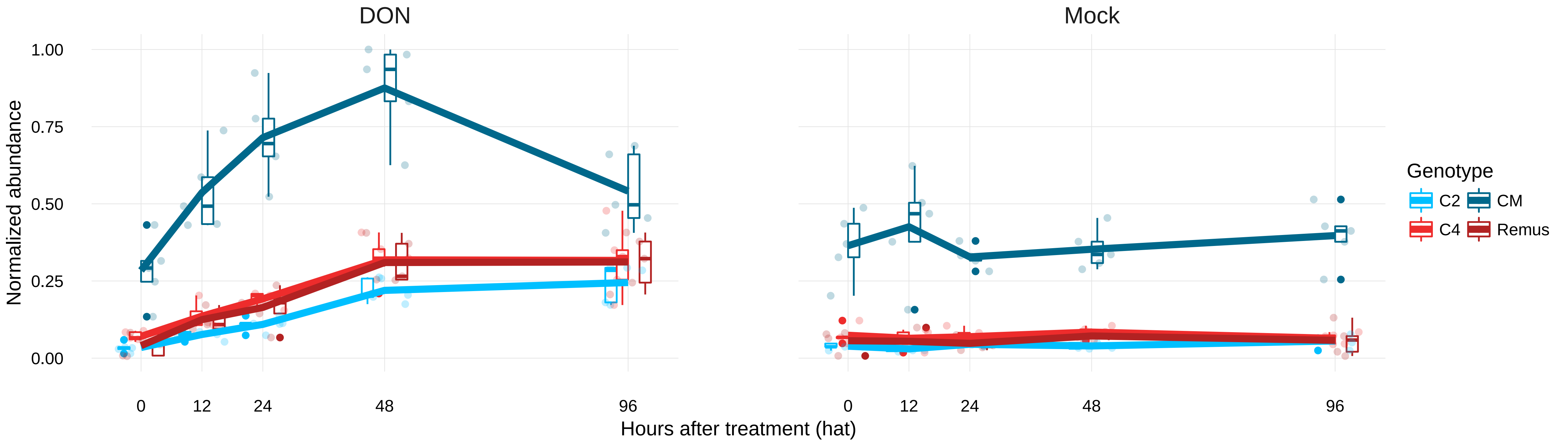

Supplement: Supplementary file 1 [file DataSheet_1.pdf]
