# Supplementary material for: Stable Isotope-Assisted Plant Metabolomics: Investigation of Phenylalanine-Related Metabolic Response in Wheat Upon Treatment With the Fusarium Virulence Factor Deoxynivalenol
Source: Front Plant Sci. 2019 Oct 30;10:1137. doi: 10.3389/fpls.2019.01137 (PMC6831647; doi:10.3389/fpls.2019.01137)

Supplementary Information 2:

The two scores plots of the two Principal Component Analysis show the samples of the experiment before (A) and after (B) normalization with the KPX samples. While the two genotypes (C2, C4) are well separated by the first principal component before KPX sample normalization, this separation is reduced after the normalization step. Moreover, the strength of this separation is also drastically reduced as can be seen by the explained variance for the scores plot before (PC1 in A) and after (PC2 in B) normalization.

(A) KPX samples before normalization

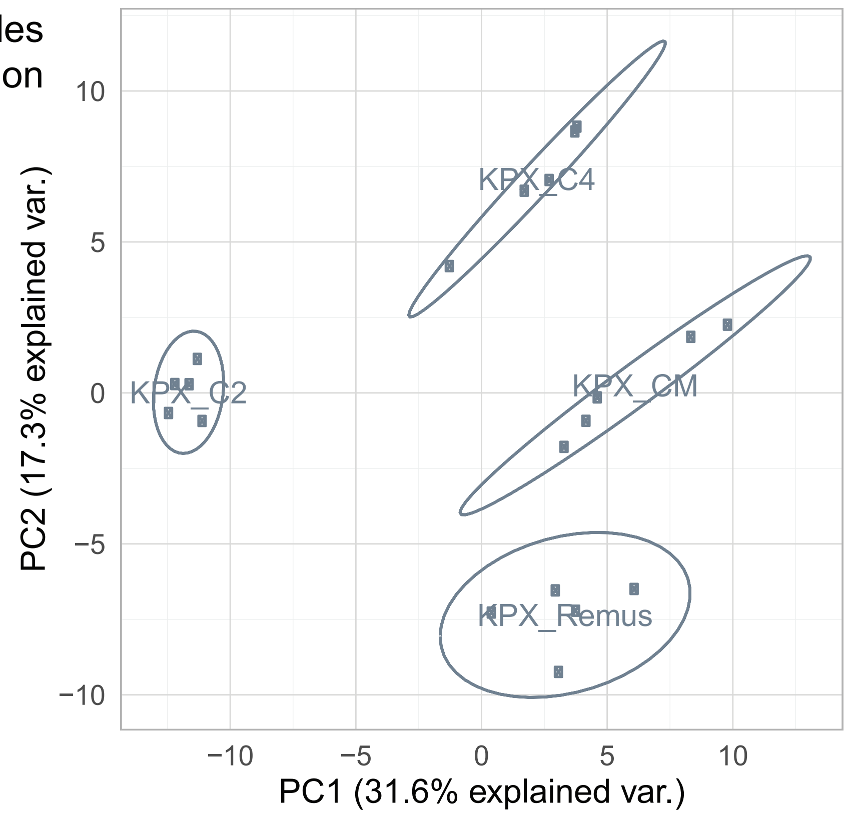

(B) KPX samples after normalization

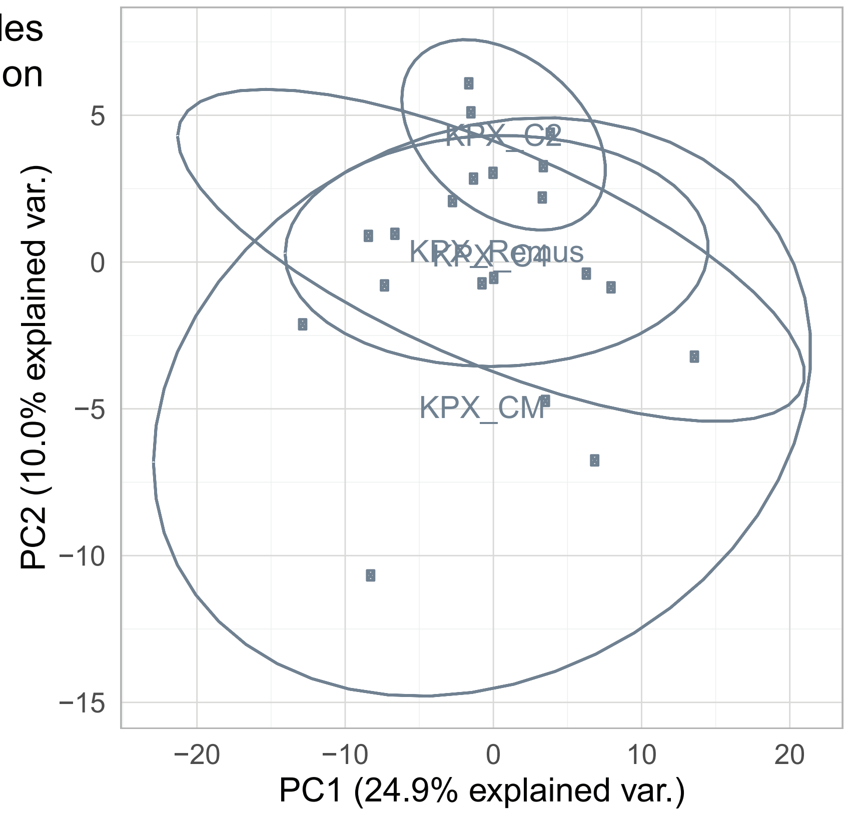

Supplement: Supplementary file 2 [file DataSheet_2.pdf]
